# Capturing Nature's Diversity

**DOI:** 10.1371/journal.pone.0120942

**Published:** 2015-04-22

**Authors:** Mauro Pascolutti, Marc Campitelli, Bao Nguyen, Ngoc Pham, Alain-Dominique Gorse, Ronald J. Quinn

**Affiliations:** 1 Eskitis Institute for Drug Discovery, Griffith University, Brisbane, Queensland, Australia; 2 QFAB Bioinformatics, The University of Queensland, Brisbane, Queensland, Australia; Broad Institute of Harvard and MIT, UNITED STATES

## Abstract

Natural products are universally recognized to contribute valuable chemical diversity to the design of molecular screening libraries. The analysis undertaken in this work, provides a foundation for the generation of fragment screening libraries that capture the diverse range of molecular recognition building blocks embedded within natural products. Physicochemical properties were used to select fragment-sized natural products from a database of known natural products (Dictionary of Natural Products). PCA analysis was used to illustrate the positioning of the fragment subset within the property space of the non-fragment sized natural products in the dataset. Structural diversity was analysed by three distinct methods: atom function analysis, using pharmacophore fingerprints, atom type analysis, using radial fingerprints, and scaffold analysis. Small pharmacophore triplets, representing the range of chemical features present in natural products that are capable of engaging in molecular interactions with small, contiguous areas of protein binding surfaces, were analysed. We demonstrate that fragment-sized natural products capture more than half of the small pharmacophore triplet diversity observed in non fragment-sized natural product datasets. Atom type analysis using radial fingerprints was represented by a self-organizing map. We examined the structural diversity of non-flat fragment-sized natural product scaffolds, rich in *sp3* configured centres. From these results we demonstrate that 2-ring fragment-sized natural products effectively balance the opposing characteristics of minimal complexity and broad structural diversity when compared to the larger, more complex fragment-like natural products. These naturally-derived fragments could be used as the starting point for the generation of a highly diverse library with the scope for further medicinal chemistry elaboration due to their minimal structural complexity. This study highlights the possibility to capture a high proportion of the individual molecular interaction motifs embedded within natural products using a fragment screening library spanning 422 structural clusters and comprised of approximately 2800 natural products.

## Introduction

Natural products (NPs) have played a pivotal role in the discovery and development of therapeutic drugs.[1] Historically, natural products from plants have been the basis for traditional medicine for thousands of years and according to the World Health Organisation (WHO) are still relied upon for primary health care by approximately 80% of the residents of developing countries.[2] Investigation of structural differences between natural products, drug substances and other chemicals, found that natural products interrogate a different and wider chemical space than synthetic derivatives.[3–6] Furthermore, it has been showed that 83% of core ring scaffolds (12977) present in NPs were absent from commercially available molecules and screening libraries.[7] The authors also suggested that including molecules containing scaffolds present in natural products would provide better opportunities to find both screening hits and chemical biology probes.[7] However, creating a high quality library incorporating unique natural product scaffolds would present a synthetic challenge due to the enormous number of scaffolds.[8] This challenge becomes less if fragment-based drug discovery (FBDD) is considered. In contrast to normal screening libraries, FBDD screens smaller libraries of relatively simple compounds with low-range molecular weight (150–300 Da).[9, 10] The exploitation of fragment combinatorics leads to greater coverage of chemical space.[11]

Here, we propose a novel approach to capture the structural diversity of nature using exclusively natural products with fragment-like physicochemical properties. This approach first identifies fragment-sized natural products from the Dictionary of Natural Products (DNP), followed by investigation of the structural diversity which was carried out by three distinct methods: atom type and atom function analyses and scaffold analysis. We demonstrate that fragment-sized natural products cover ~66% of small pharmacophore features found in natural products. Further structural diversity analysis using radial fingerprint of fragment-sized molecules suggested that natural products with non-flat 2-ring scaffolds show the greatest diversity with a potential to be used as biology probes.

## Results and Discussion

### Property space analysis to identify fragment-sized natural products

The Dictionary of Natural Products (DNP) was used as the natural product database source for this work. Although the composition of the database is a reflection of numerous biases related to the collection, isolation or identification of biota and their chemical constituents, the DNP is regarded as a good representative collection of all known natural products. The identification of fragment-sized natural products from the DNP (sdf version 211.9) was carried out through a two-step filtering process. The first part involved the identification of “clean” natural products. This was accomplished by removing duplicates, stripping salts, structure normalization and standardization. The remaining entities were then ionized at pH 7.4 and inorganic molecules were removed. A further complexity filter (molecular weight ≥ 100 Da or heavy atom count ≥ 7 and sulphur atoms counts ≤ 3) yielded 165281 clean natural products. Throughout the scientific literature,[12, 13] fragment-like properties have not been defined using standardized or even universally recognized criteria. Initially, fragment-like structures were identified by the empirical rule of three Ro3 in which molecular weight (MW) is < 300 Da, ClogP is ≤ 3, hydrogen bond donors (HBD) is ≤ 3 and hydrogen bond acceptors (HBA) is ≤ 3.[14] Later, experimental evidence suggested a lower molecular weight cutoff would be more appropriate in fragment screening collections.[15] Thus, the identification of fragment-sized natural products in this study was carried out according to the following in-house filtering criteria: MW ≤ 250 Da, ClogP < 4, rotatable bonds (RTB) ≤ 6, HBD ≤ 4, HBA ≤ 5 and polar surface area (PSA) < 45%, number of rings (RNG) ≥ 1, which resulted in 20185 fragment-sized natural products (and 145096 non fragment-sized natural products).

**Fig 1:**
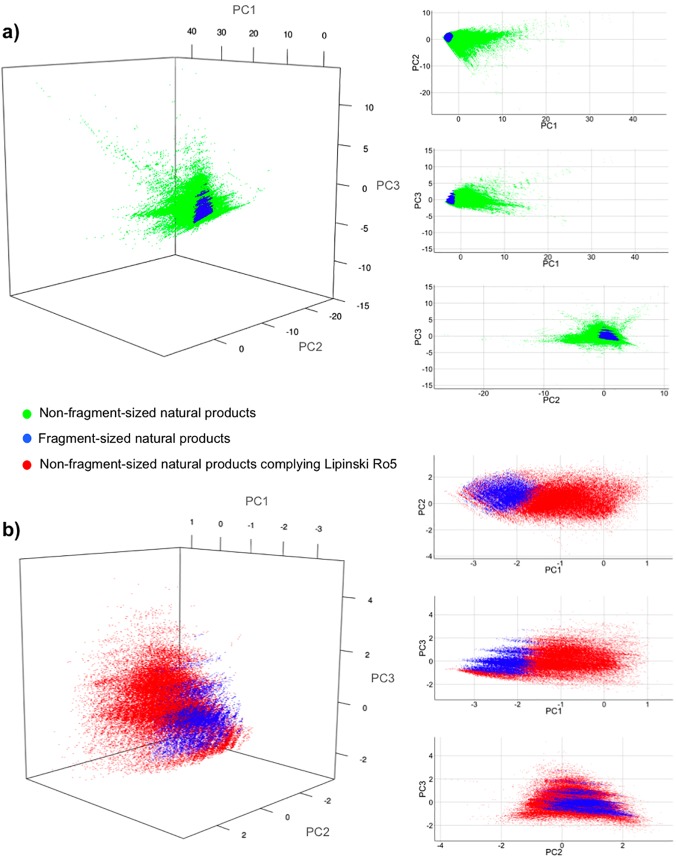
PCA analysis of the DNP. Comparison of fragment-sized natural products to all known natural products (DNP) in physicochemical property space as defined by principal component analysis of 11 physicochemical descriptors. The first three principal components account for 89.0% of the variance in the data (57.6%, 21.2%, and 10.2%, respectively). **(a)** PCA plot of 165281 clean natural products (145096 non-fragment-sized NPs (green) and 20185 fragment-sized NPs (blue). **(b)** PCA plot of 94929 natural products (74744 non-fragment sized natural products which satisfy Lipinski’s Ro5 (red) and 20185 fragment-sized NPs (blue)).

To investigate the coverage of physiochemical property space of fragment-sized natural products versus non-fragment-sized natural products, a principal component analysis (PCA) was performed using 11 physicochemical descriptors (ClogP, MW, HBA, HBD, RTB, Number of Atoms, RNG, Number of Aromatic Rings, Molecular Solubility, Molecular Surface Area, PSA). Visual illustration of the first three principal components for both datasets is shown in a 3D-plot in Fig 1. As expected, due to their vast property diversity, non-fragment-sized natural products (green—Fig 1A) cover a much larger area of the property space when compared to the fragment subset. (blue—Fig 1A).

Lipinski’s Ro5[16] has been developed to flag molecules which could cause pharmacokinetic problems during drug-development. By applying the Ro5 bioavailability filter to non-fragment natural products, we identified 74744 molecules which passed the Lipinski rules filter (*i*.*e*. MW < 500 Da, ClogP < 5, HBD < 5, HBA < 10). A significant difference was observed between the fragment-sized natural products (blue) and Lipinski-compliant natural product (red) in PC1 (Fig 1B). The corresponding PCA loadings data suggests that descriptors primarily related to reduced molecular complexity, such as lower HBD, HBA and ring counts, are responsible for the observed difference (S1 Table). This result is not unexpected since the criteria for defining the fragment-size subset of natural products includes property filters specifically designed to reduce complexity in line with fundamental concepts. This analysis ultimately provided a visual representation of the physicochemical property space of non-fragment and fragment-sized natural products. Even though fragments occupy a smaller physicochemical space, we believe that this is desirable from a molecular complexity and synthetic expansion standpoint since potential “hits” will have more room to be modified before violating Lipinski’s Ro5.

### Chemical space analysis

Fragment-sized natural products where compared to non-fragment sized natural products using atom function and atom type analyses to determine if fragment-sized natural products could potentially represent a significant proportion of natural product chemical space.

**Fig 2:**
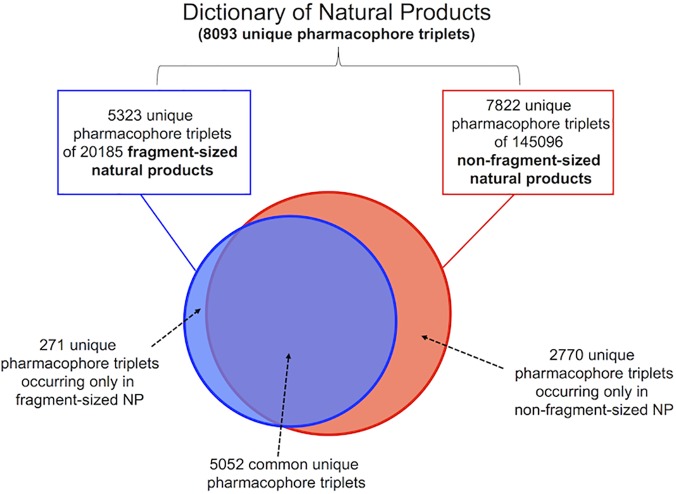
Pharmacophore analysis of natural products. Based on the number of unique pharmacophore triplets (1–6 bonds), generated using eight features, we identified that fragment-sized natural products cover ~66% of the unique pharmacophore of the whole DNP.

2D Topological pharmacophore triplets,[17] were used to compare the DNP datasets. The pharmacophore concept has been widely used in molecular recognition and virtual screening and consists in the spatial arrangement of a set of functional group types (features). Pharmacophore triplets were generated for all the possible combinations of three pharmacophore points in the molecule within a topological distance between 1–6 bonds. The pharmacophore triplet size restriction was used for the analysis to identify small regions of complex natural products capable molecular recognition with small, contiguous areas of a protein binding surface. Using a set of 8 features (HBA, HBD, positive charge, negative charge, positive ionisable atom, negative ionisable atom, aromatic ring, hydrophobic), we identified 8093 unique triplets for the DNP (165281 compounds), while the 20185 fragment-sized natural products had 5323 unique triplets and the 145096 non-fragment-sized natural products had 7822 unique triplets. Interestingly, there were 5052 common pharmacophore triplets between both datasets. This indicates that a relatively small number of fragment-sized natural products capture ~66% of the unique pharmacophore triplets of the DNP (Fig 2). Furthermore, we also identify 271 unique pharmacophore triplets belonging exclusively to the fragment-sized dataset.

From this analysis, we believe that fragment-sized natural products could be potentially used as a starting point for the generation of a highly diverse library based on small molecules from natural sources. An opportunity exists to access the remaining 2770 pharmacophore triplets (~34%) not identified within fragment-sized natural products via chemical synthesis of small portions of complex natural products that present these pharmacophoric features.

### Structural diversity analysis of fragment-sized scaffolds

The structural diversity of fragment-sized natural products was analysed using radial fingerprints encoding 2D topological atom environment (ECFP_4).[18, 19] This type of fingerprint is one of the most widely used, and represents the environment of atoms in the neighbourhood of each heavy atom in the molecule within a four-bond diameter.

**Fig 3:**
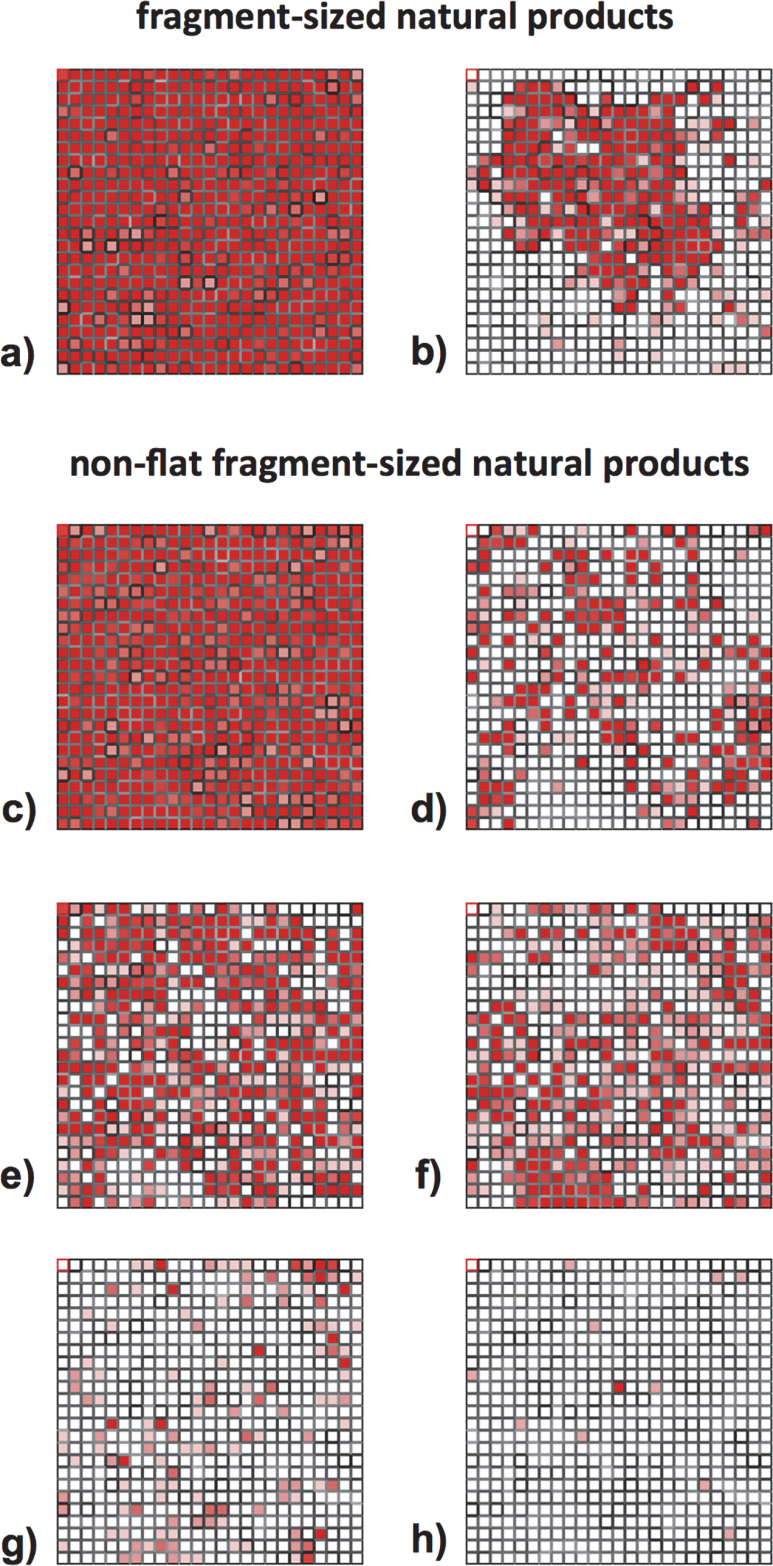
Distribution of compounds within the SOMs trained using ECPF_4 fingerprints of 20185 fragment-sized natural products (Fig 3A and 3B) and 7365 non-flat fragment-sized natural products (Fig 3C–3H). **(a)** 20185 fragment-sized natural products. **(b)** 7365 non-flat fragment sized natural products (F*sp* ^3^* > 0.45). **(c)** 7365 non-flat fragment-sized natural products. **(d)** 1-ring molecules; 37% coverage of non-flat fragments. **(e)** 2-ring molecules; 68% coverage of non-flat fragments. **(f)** 3-ring molecules; 56% coverage of non-flat fragments. **(g)** 4-ring molecules; 21% coverage of non-flat fragments. **(h)** 5-ring molecules; 2% coverage of non-flat fragments. Each cell represents a cluster of fragments and the distance between cells (i.e. nearby cell are structurally related compounds) is indicated by the shading of the cell borders; darker borders indicate larger distance. Cells are coloured by population, with white for empty cells, and red for cell containing more than 5 compounds. The trained SOM is characterized by a toroidal architecture, which means that the top edge is connected to the lower edge and the left edge with the right edge.

The total diversity of fragment sized natural products was then represented by training a self-organizing map 25x25 (SOM)[20] using radial fingerprints of the 20185 natural products meeting our fragment criteria (Fig 3).

A recent report by Lovering[21] pointed out that increasing the complexity of molecules in terms of carbon bond saturation, expressed as F*sp* ^3^, and with the presence of chiral centres in the molecule, could potentially translate to greater success in HTS screening. In our opinion, since flat and aromatic fragment-sized natural products are over-represented in existing fragment libraries, a higher degree of three dimensionality is necessary for accessing new areas of chemical space. We therefore decided to focus our studies on non-flat systems.

For our analysis, because of the importance of the overall shape and substituent positioning, the flatness of the molecules was defined by the F*sp* ^3^ content of Murcko scaffolds[22] (F*sp* ^3^*) rather than the F*sp* ^3^ values of the whole structure. (S2 Table provides some examples of F*sp* ^3^* and F*sp* ^3^ calculated values of the selected molecule).

Based on some calculated mean F*sp* ^3^ values reported for drug datasets[21, 23, 24], and also based on our previous report[25], we defined flat fragment-sized natural product with F*sp* ^3^* ≤ 0.45 and non-flat fragment with F*sp* ^3^* > 0.45. Using this cut off, from 20185 natural product fragments, 7365 were identified as non-flat molecules. Interestingly, projection of the non-flat fragments into the trained SOM revealed that the central area of the map was predominantly occupied by three-dimensional structures, covering 45% of the SOM cells (Fig 3B).

A new rectangular 25x25 SOM was generated training 7365 ECFP_4 fingerprints of non-flat fragment-size natural products (Fig 3C). The structural diversity of this dataset was investigated by clustering the molecules by the number of rings. Hence, we identified: 2636 1-ring fragments, 2822 2-ring fragments, 1621 3-ring fragments, 273 4-ring fragments and 13 5-ring fragments. A visual representation of the diversity coverage for each dataset is shown in Fig 3C–3H. Although 1-ring and 2 ring fragment-sized natural products have almost similar number of structures, clearly, the 2-ring projection gave the highest diversity covering 68% of the SOM cells.

**Fig 4:**
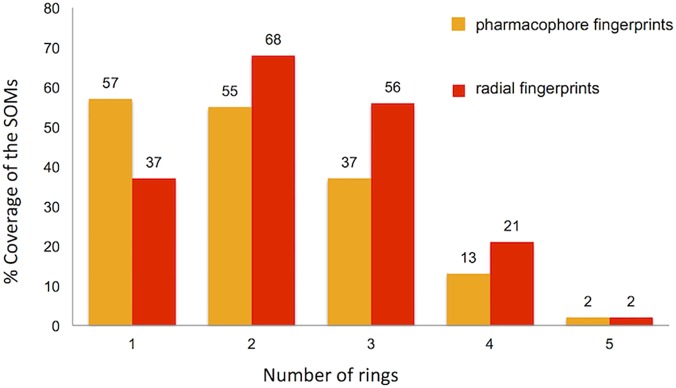
Structural diversity analysis of 7365 non-flat fragment-sized natural products based on pharmacophore (orange) and radial (red) fingerprints. Using SOMs (generated by training non-flat structures subset) we analyzed the distribution and therefore the coverage of the total diversity of non-flat fragments by the subsets clustered on the number of rings present in the structure.

A second approach to evaluate the diversity of non-flat fragment-sized natural products was based on pharmacophore analysis. From 7365 non-flat fragment-sized natural products we identified 2602 unique triplets using the set of 8 features with topological distance of 1–6 bonds. In parallel, unique triplets were identified for each of the five fragment-sized natural product subsets of 1- to 5- ring molecules: 1658 unique triplets for 1-ring subset (2636 molecules); 1394 for 2-ring subset (2822 molecules); 963 for 3-ring subset (1621 molecules); 348 for 4-ring (273 molecules); and 37 for 5-ring subset (13 molecules). Similarly to the previous approach, the diversity of fragment-sized natural products was displayed by a 25x25 SOM using pharmacophore fingerprints of the 20185 fragment-sized natural products. Projection of the non-flat structures into the SOM, revealed 54% coverage of the SOM cells by the non-flat natural products dataset (S1A Fig). The diversity of non-flat fragment-sized natural products and its subsets of 1, 2, 3, 4 and 5-ring molecules was also projected onto to a 25x25 SOM to calculate the percentage coverage of each subsets (S1B Fig). As outlined in Fig 4, the analysis based on pharmacophore fingerprints, one- and two-ring structures showed the highest diversity with coverage of 57% and 55% respectively whereas as shown previously when radial fingerprints were used, two- and three-ring structures provide the highest diversity. As expected, because the two methods employed capture different aspect of the molecules, the two analyses provided different outcomes, however the 2-ring subset, in both cases, showed the best coverage of the total diversity of non-flat fragment-sized natural products.

A further evaluation of the diversity of non-flat fragment-sized natural products was assessed investigating the diversity of the structures at the core level by ring scaffold analysis. Scaffold abstraction based on the Bemis and Murcko approach,[22] was performed generating 2473 scaffolds from the 7365 non-flat fragment-sized natural products. In addition, an analysis of the five datasets (clustered by the number of rings) was performed. From 1-ring fragment-sized dataset (2663 molecules), 303 scaffolds were identified. Among these scaffolds, 140 were singletons (molecules that occur just once). This suggests that the diversity of 1-ring fragments is mainly dictated by the substituents on the core ring structure. Scaffold analysis of 2-ring fragment-sized dataset (2822 molecules) revealed the presence of 929 scaffolds, which suggests a high level of diversity within the group. Moreover, from these 929 scaffolds, 737 were fused ring systems (2527 molecules) and 192 non-fused ring systems (295 molecules). It was also found that 61% (569 scaffolds) of the abstracted scaffolds from this dataset were singletons. This indicates that the molecular diversity of 2-ring fragment-sized natural products is mainly dictated by the variation of the core ring scaffold. Analysis of the 3-ring fragment-sized dataset (1621 molecules) led to the identification of 1008 scaffolds in which, 942 scaffolds featured three-fused rings system where only 66 scaffolds showed non-fused ring structures. Not surprisingly, 76% (766 singleton scaffolds) of the 1008 scaffolds were identified as singletons. When more complex systems, such as 4-ring and 5-ring system datasets where analyzed, 220 and 13 scaffolds respectively were detected. In the 4-ring group, 87% of the scaffolds revealed to be singletons (191 scaffolds) whereas in the 5-ring scaffold only singletons were observed.

### Generation of highly diverse fragment-based libraries

The structural diversity of non-flat fragment-sized natural products was assessed by two approaches using 2D topological fingerprints. In the first case, when the analysis was carried out using radial fingerprints, we observed that the 2-ring fragments dataset showed the highest diversity with a coverage of 68% of the SOM cells of non-flat fragment-sized natural products. The pharmacophore fingerprints approach revealed that 1-ring and 2-ring datasets have the best coverage of the non-flat natural products SOM (57% and 55% respectively). Interestingly, when the analysis was carried out considering just the underlying core structure, 2- and 3-ring datasets presented the highest number of unique scaffolds, suggesting that the diversity of these two datasets is predominantly due to the molecular framework.

Returning to our design of a fragment-based library consisting of naturally occurring small molecules, the data shows that 2-ring non-flat fragment-sized natural products potentially represents a highly diverse library. Although the term diversity can often lead to completely different outcomes depending on the method of analysis used (*i*.*e* atom type or atom function), our results are also in accordance with Langdon[26] and Tian[27] reports in which they identified level 1 of the scaffold tree[28] (2-ring scaffolds) as the best choice for scaffold diversity analysis.

**Fig 5:**
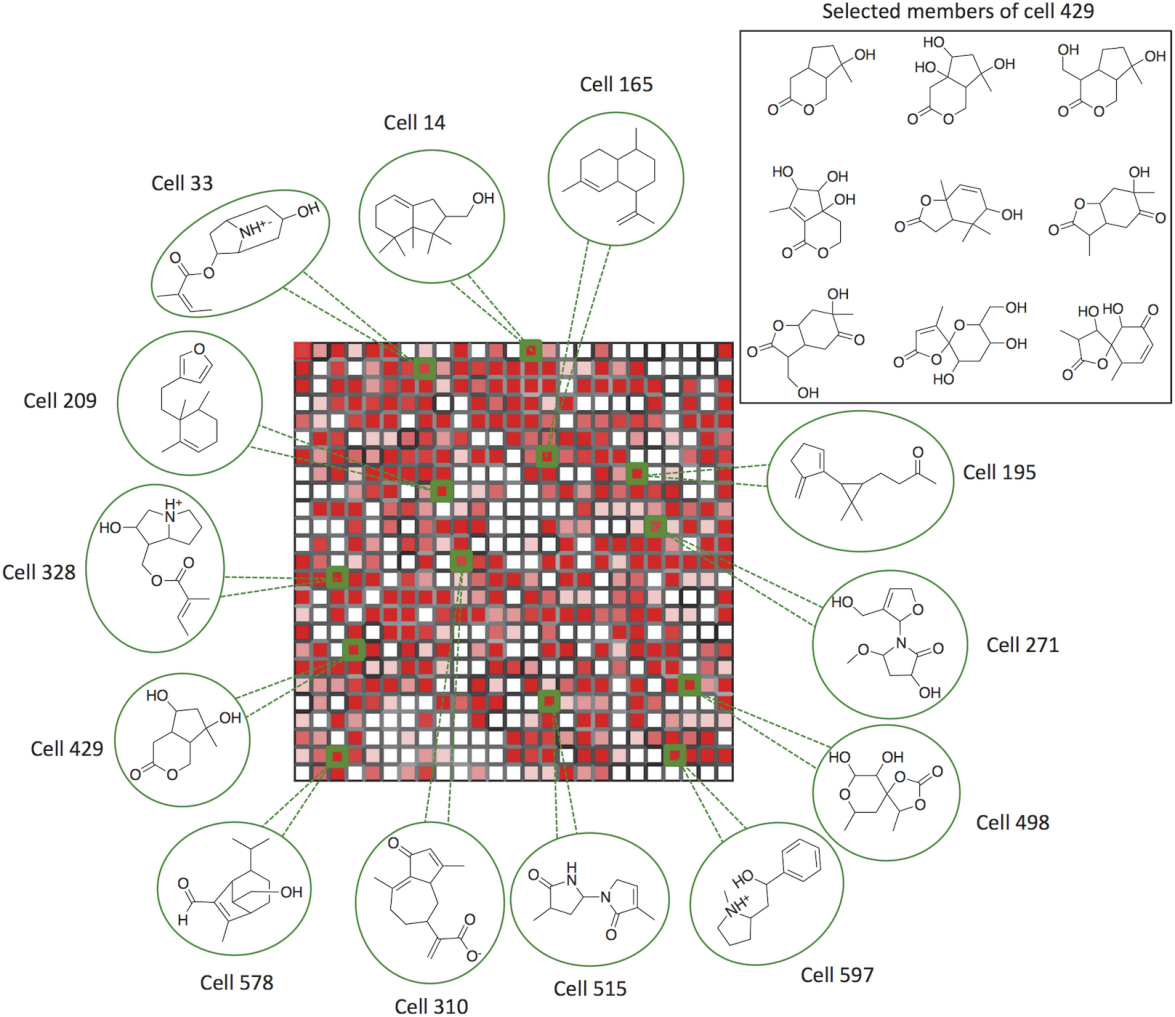
Distribution of 2-ring fragments dataset within the non-flat fragment-sized natural products SOM. For a few selected cells are, the most representative structures (green circle) is shown. In the black square box is reported one example of the molecular similarity within the same cell. The entire list of seed compounds can be found in S3 Table.

With the goal to explore a large chemical space using a minimal number of simple molecules (400–500 compounds), we provide additional examination of the 2-ring fragments dataset. For this analysis we employed the widely used radial fingerprints (ECFP_4), although this can also be performed with a different type of fingerprints (*i*.*e* pharmacophore fingerprints). As discussed previously, 2822 fragment-sized natural products (2-ring dataset) were clustered using a self-organizing map, which showed a coverage of 68% of the diversity of fragment-sized NPs. From the 422 occupied cells, 62 were singletons, suggesting that the 62 molecules are the most unique and cover 10% of the non-flat fragment-sized NPs diversity. In addition, based on Tanimoto similarity, we identified the most representative structure for each cell as the molecule for which the average distance to the other molecules within the same cell was minimal (S3 Table). Fig 5 shows a few examples of the most representative non-flat 2-ring fragment-sized natural products.

Ultimately this analysis provides a tool that can be used by the natural product and medicinal chemistry community for the generation of libraries featuring a high degree of diversity. The analysis also identifies that a minimal number of compounds provide an extensive coverage of natural product chemical space.

## Conclusion

The chemical space of all known natural products was analysed using pharmacophore triplets and 2D topological fingerprints. Herein we demonstrated that 20185 fragment-sized NP captured ~66% of the small pharmacophore features of the DNP (~165 thousand compounds).

Given the importance of non-flat structures in drug discovery,[12, 29] we examined the diversity of fragment-sized natural products rich in *sp* ^3^- configured centres and identified that those molecules based on 2-ring scaffolds effectively balance the opposing characteristics of minimal complexity and broad structural diversity when compared to the larger, more complex fragment-like natural products.

The task to deliver 12977 unique ring scaffolds, identified by the Shoichet’s group as unrepresented natural product scaffolds among commercial molecules, through synthetic methodologies followed by library enumeration, we believe to be out of reach of current methodologies. Here, we have presented 422 structural clusters, comprised of approximately 2800 natural products, for application in chemical biology and drug discovery with a particular potential in fragment based drug discovery.

## Methods

### Identification of fragment-sized natural products

Fragment-sized natural products were obtained from Dictionary of Natural Product (DNP sdf version 211.9) through a filtering process using SciTegic Pipeline Pilot 8.5.0.200. The first step involved the generation of “clean natural products” which were further filtered to yield fragment-sized natural products. *First step*: salts were stripped and the largest fragments were kept; all atoms that were not part of the largest connected structure fragment in each input molecule were identified and removed. The structures were then normalized and invalid structures were removed. The molecules were modified so that the protonation state of pKa sites was set according to pH 7.4. Molecules that did not contain atom types such as H, C, N, O, S, and halogen atoms were considered as inorganic and removed. A complexity filter was then applied (MW ≥ 100 Da or heavy atom count ≥ 7 and sulphur atoms ≤ 3) producing 165281 natural products. The identification of fragment-sized natural products was carried out using the following fragment criteria filter (MW ≤ 250 Da, ClogP < 4, HBA ≤ 5, HBD ≤ 4, PSA < 45, RTB ≤ 6, RNG ≥ 1) yielding 20185 molecules. The remaining 145096 were therefore non-fragments NPs.

### Property space analysis

Based on physicochemical properties, principal components analysis (PCA) was carried out using Pipeline Pilot. The 11 variables (ClogP, MW, HBD, HBA, RTB, Number of Atoms, RNG, Number of Aromatic Rings, Molecular Solubility, Molecular Surface Area, PSA) were mean, centred and scaled to the unit variance. The first three principal components which explain 89.0% of the variance, were illustrated in a 3D-plot using RStudio 0.97.551 (Fig 1). S1 Table summarizes the corresponding loadings for the first three PCs.

### Pharmacophore triplet calculation

The number of unique pharmacophore triplets was determined based on pharmacophore fingerprints (1024 bit length) calculated using Pipeline Pilot. The procedure includes the following steps: generation of pharmacophore triplets for each molecule, canonicalization of triplets using sorting features by type (for example, HA not AH), removal of duplicates. Molecules, which did not contain any pharmacophore triangles or triplets, were removed.

### Structural diversity analysis

The diversity analysis of fragment-sized natural products (20185 structures) based on radial fingerprints (ECFP_4, 1024 bit length) was carried out using Canvas by Schrodinger (version 1.5.518). At first a 25x25 SOM was trained using ECFP_4 of 20185 fragment-sized natural products. As mentioned in the text, the need to work with *sp* ^3^ rich structures led us to focus our work on exclusively non-flat systems. To provide a better analysis a new 25x25 SOM was trained using 7365 non-flat fragment-sized natural products.

The diversity analysis based on pharmacophore fingerprints was accomplished using Pipeline Pilot. Also in this case, an initial 25x25 SOM was trained using 20185 structures. Furthermore the analysis was carried out on a new trained SOM (25x25) based exclusively on 7365 non-flat fragment-sized natural products. (S1 Fig)

### Scaffold generation and analysis

The identification of ring scaffolds was accomplished with Canvas software. Using a build-in function, based on Bemis-Murcko approach, from 7365 non-flat fragment-sized natural products, 2473 scaffolds were identified. In this process, contiguous ring systems were extracted with retention of exocyclic double bonds to terminal atoms in order to preserve important chemical functionality.

## Supporting Information

S1 Table Loadings for the first three principal components of the property space of natural products database. (PDF)

S2 Table Some examples showing a clear difference between F*sp* ^3^ and F*sp* ^3^* calculated values. (PDF)

S3 Table Identification of the most representative 2-ring fragment-sized natural products. (PDF)

S1 Fig Self-organizing maps of the DNP and fragment-sized natural products using pharmacophore fingerprint. (PDF)
