# Supplementary material for: Capturing Nature's Diversity
Source: PLoS One. 2015 Apr 22;10(4):e0120942. doi: 10.1371/journal.pone.0120942 (PMC4406718; doi:10.1371/journal.pone.0120942)
Supplement: S1 Table — (PDF) [file pone.0120942.s001.pdf]

S1 Table. PCA analysis. Loadings for the first three principal components of the property space of natural products database. (PCA plots in Fig. 1)

	**PC1**	**PC2**	**PC3**
Cumulative variance	0.576	0.788	0.89
ClogP	-0.02158	-0.16983	0.07724
Molecular weight	0.00151	-0.00033	0.00002
Hydrogen bond acceptors	0.09325	0.06010	-0.03076
Hydrogen bond donors	0.05943	0.02901	-0.00713
Rotatable bonds	0.03399	-0.05029	-0.04794
Number of atoms	0.02154	-0.00510	0.00178
Number of rings	0.11769	0.04387	0.16825
Number of aromatic rings	0.07699	0.05688	0.63688
Molecular solubility	-0.03850	0.18205	-0.01040
Molecular surface area	0.00149	-0.00066	-0.00026
Polar surface area	0.00357	0.00192	-0.00081
