# Supplementary material for: Capturing Nature's Diversity
Source: PLoS One. 2015 Apr 22;10(4):e0120942. doi: 10.1371/journal.pone.0120942 (PMC4406718; doi:10.1371/journal.pone.0120942)
Supplement: S2 Table — (PDF) [file pone.0120942.s002.pdf]

S2 Table. Flatness identification. Some examples showing a clear difference between Fsp^3 and Fsp^{3*} calculated values.

Structure	Fsp^3	Extracted scaffold	Fsp^{3*}
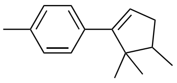	0.47	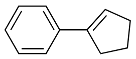	0.27
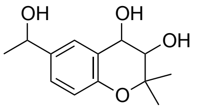	0.54	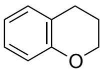	0.33
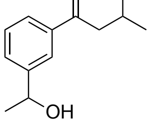	0.46	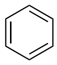	0
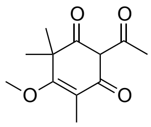	0.50	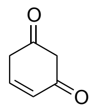	0.33
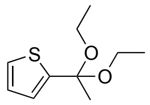	0.60	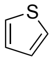	0
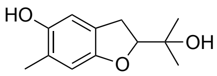	0.50	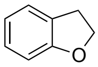	0.25
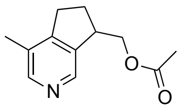	0.50	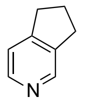	0.38
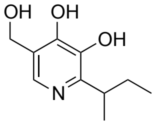	0.50	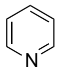	0
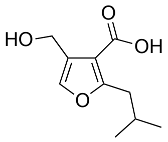	0.50	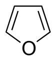	0
