# Supplementary material for: Capturing Nature's Diversity
Source: PLoS One. 2015 Apr 22;10(4):e0120942. doi: 10.1371/journal.pone.0120942 (PMC4406718; doi:10.1371/journal.pone.0120942)
Supplement: S3 Table — (PDF) [file pone.0120942.s003.pdf]

S4 Table. Calculation of the most representative structures based on Tanimoto similarity. 422 Cluster sets of 2-ring non-flat fragment-sized natural products. The molecules shown are ionized at pH=7.4.

Cell ID, number of the cell; the numbering start from the top left corner (cell 1) and increases sequentially from the left to the right.

Cluster Center = 1 indicates the representative molecule of the cluster cell.

Cluster Center = 0 indicates molecules as cluster members.

Number of Compounds, indicates the number of cell members.

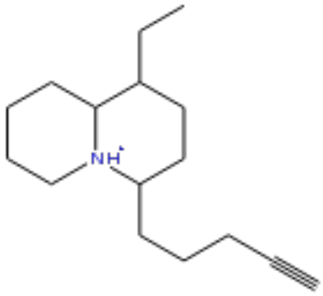

Cell ID	Cluster Center	Number of Compounds
1	1	4

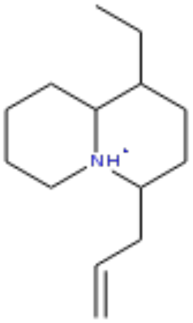

Cell ID	Cluster Center	Number of Compounds
1	0	4

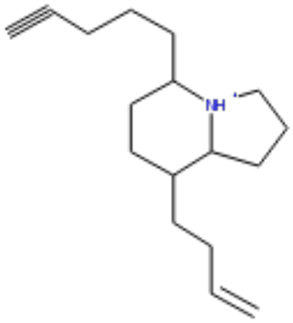

Cell ID	Cluster Center	Number of Compounds
1	0	4

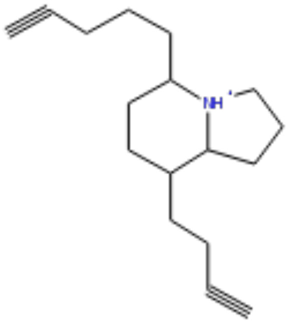

Cell ID	Cluster Center	Number of Compounds
1	0	4

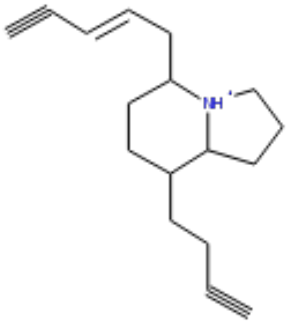

Cell ID	Cluster Center	Number of Compounds
2	1	2

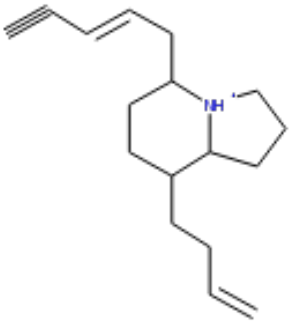

Cell ID	Cluster Center	Number of Compounds
2	0	2

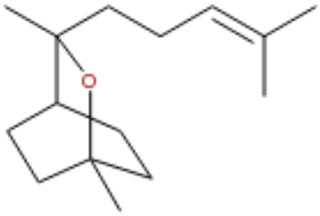

Cell ID	Cluster Center	Number of Compounds
3	1	5

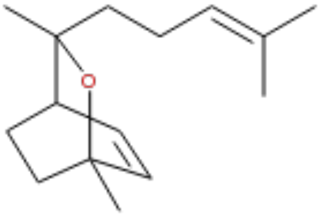

Cell ID	Cluster Center	Number of Compounds
3	0	5

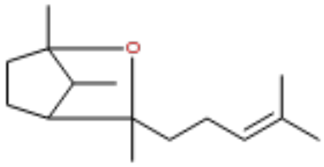

Cell ID	Cluster Center	Number of Compounds
3	0	5

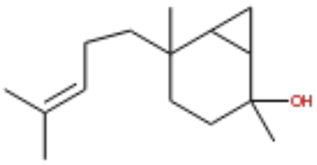

Cell ID	Cluster Center	Number of Compounds
3	0	5

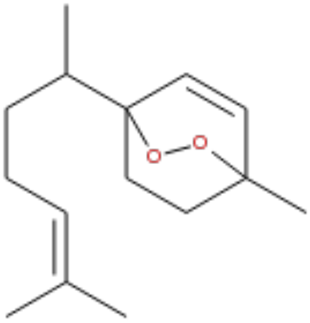

Cell ID	Cluster Center	Number of Compounds
3	0	5

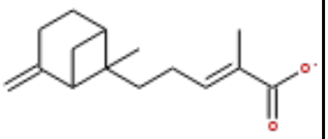

Cell ID	Cluster Center	Number of Compounds
4	1	1

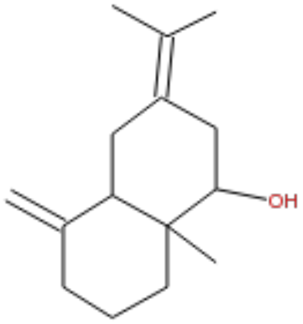

Cell ID	Cluster Center	Number of Compounds
5	1	5

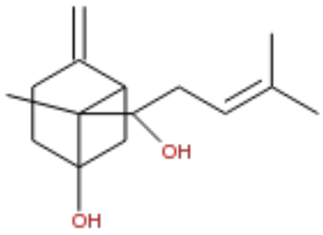

Cell ID	Cluster Center	Number of Compounds
5	0	5

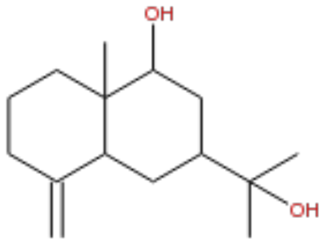

Cell ID	Cluster Center	Number of Compounds
5	0	5

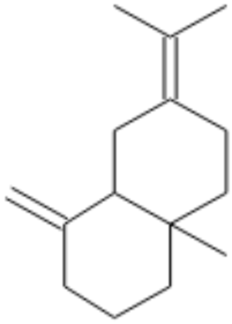

Cell ID	Cluster Center	Number of Compounds
5	0	5

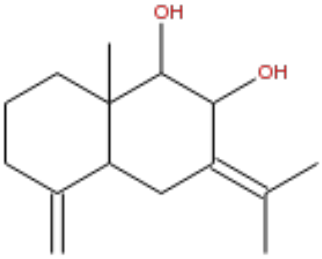

Cell ID	Cluster Center	Number of Compounds
5	0	5

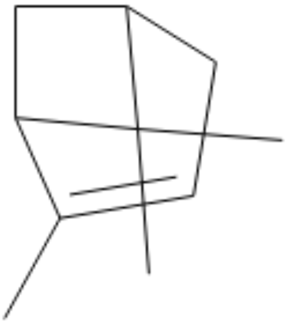

Cell ID	Cluster Center	Number of Compounds
6	1	22

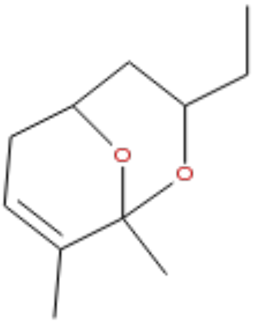

Cell ID	Cluster Center	Number of Compounds
6	0	22

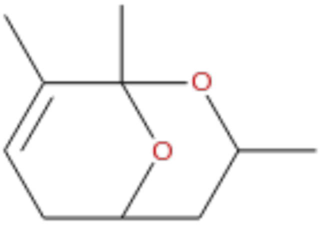

Cell ID	Cluster Center	Number of Compounds
6	0	22

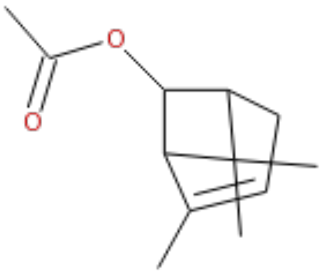

Cell ID	Cluster Center	Number of Compounds
6	0	22

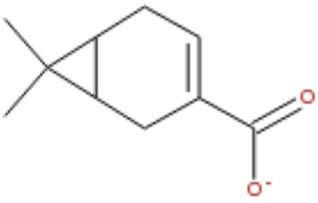

Cell ID	Cluster Center	Number of Compounds
6	0	22

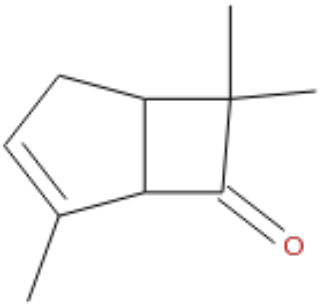

Cell ID	Cluster Center	Number of Compounds
6	0	22

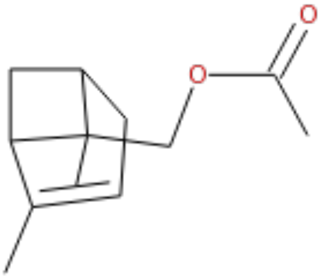

Cell ID	Cluster Center	Number of Compounds
6	0	22

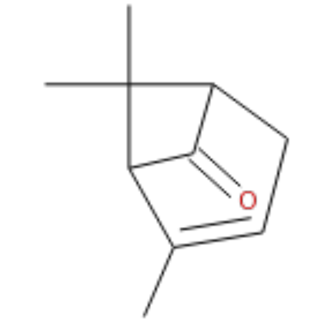

Cell ID	Cluster Center	Number of Compounds
6	0	22

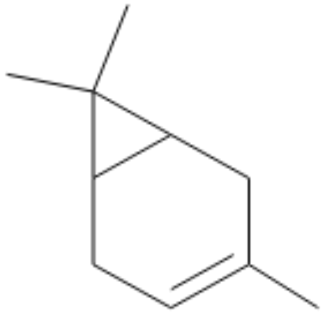

Cell ID	Cluster Center	Number of Compounds
6	0	22

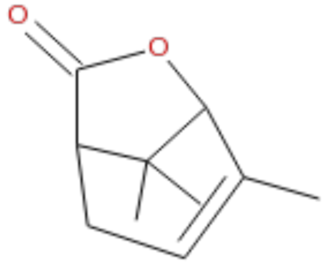

Cell ID	Cluster Center	Number of Compounds
6	0	22

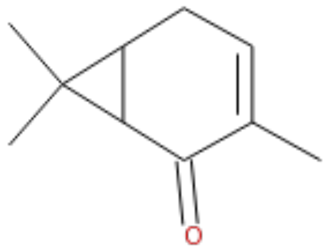

Cell ID	Cluster Center	Number of Compounds
6	0	22

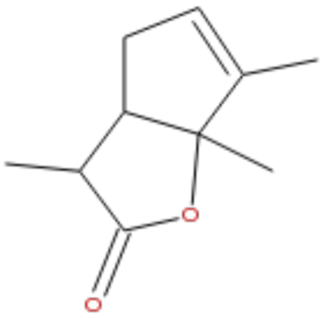

Cell ID	Cluster Center	Number of Compounds
6	0	22

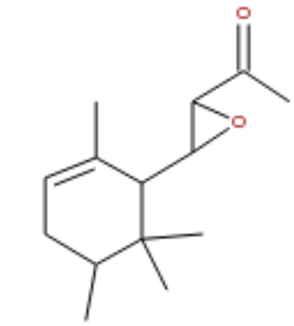

Cell ID	Cluster Center	Number of Compounds
6	0	22

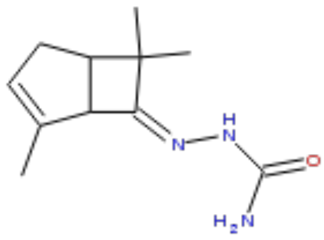

Cell ID	Cluster Center	Number of Compounds
6	0	22

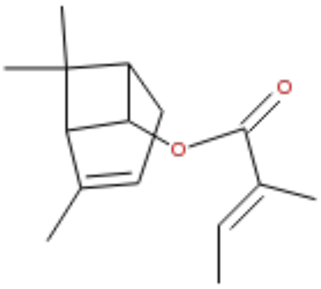

Cell ID	Cluster Center	Number of Compounds
6	0	22

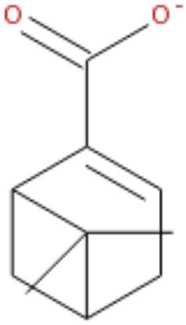

Cell ID	Cluster Center	Number of Compounds
6	0	22

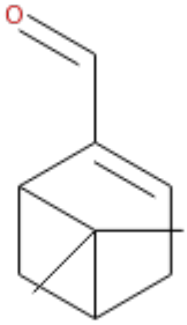

Cell ID	Cluster Center	Number of Compounds
6	0	22

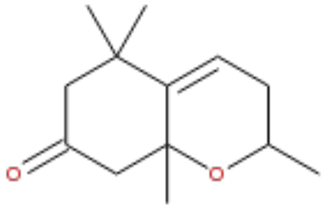

Cell ID	Cluster Center	Number of Compounds
6	0	22

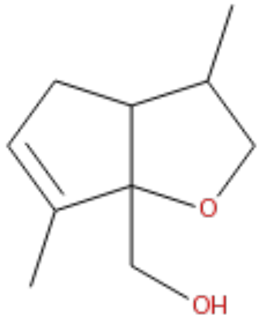

Cell ID	Cluster Center	Number of Compounds
6	0	22

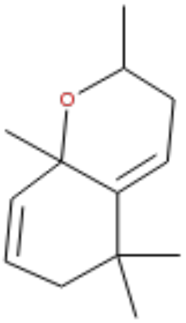

Cell ID	Cluster Center	Number of Compounds
6	0	22

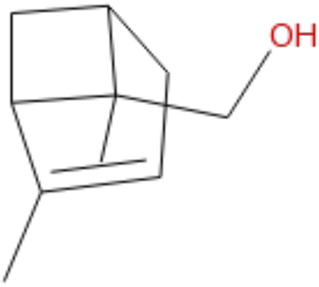

Cell ID	Cluster Center	Number of Compounds
6	0	22

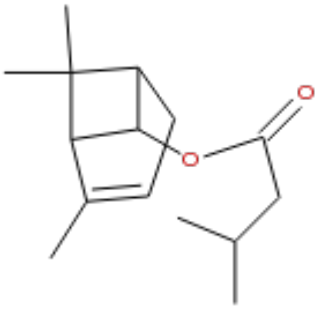

Cell ID	Cluster Center	Number of Compounds
6	0	22

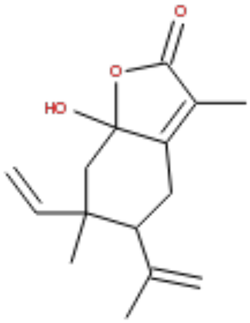

Cell ID	Cluster Center	Number of Compounds
8	1	1

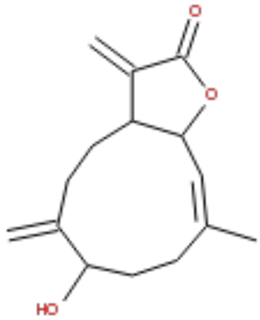

Cell ID	Cluster Center	Number of Compounds
10	1	14

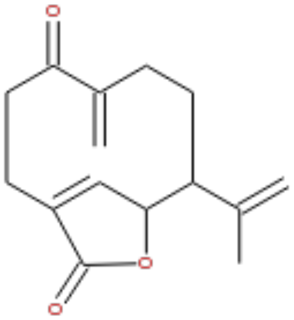

Cell ID	Cluster Center	Number of Compounds
10	0	14

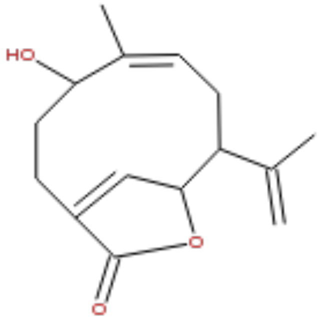

Cell ID	Cluster Center	Number of Compounds
10	0	14

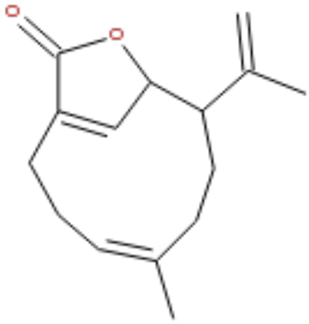

Cell ID	Cluster Center	Number of Compounds
10	0	14

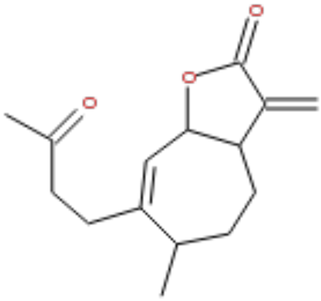

Cell ID	Cluster Center	Number of Compounds
10	0	14

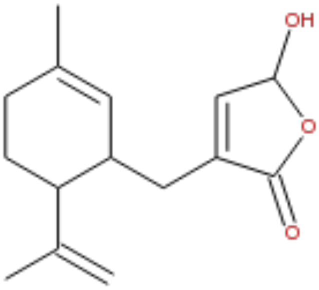

Cell ID	Cluster Center	Number of Compounds
10	0	14

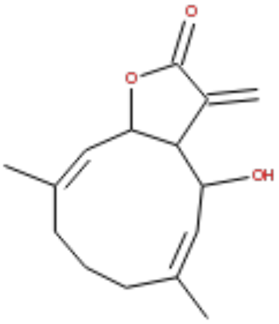

Cell ID	Cluster Center	Number of Compounds
10	0	14

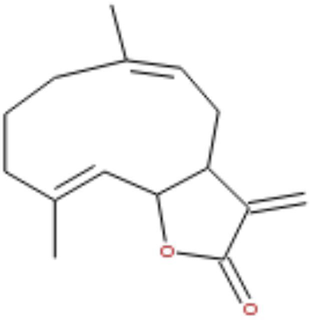

Cell ID	Cluster Center	Number of Compounds
10	0	14

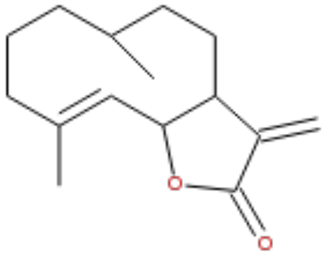

Cell ID	Cluster Center	Number of Compounds
10	0	14

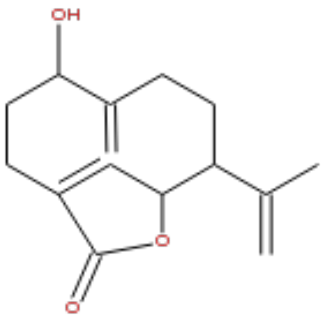

Cell ID	Cluster Center	Number of Compounds
10	0	14

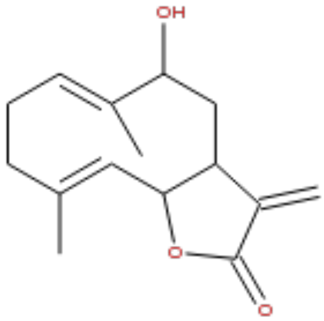

Cell ID	Cluster Center	Number of Compounds
10	0	14

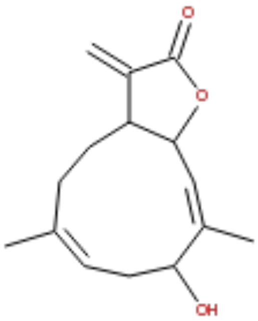

Cell ID	Cluster Center	Number of Compounds
10	0	14

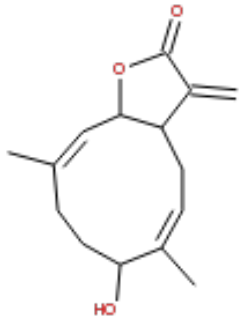

Cell ID	Cluster Center	Number of Compounds
10	0	14

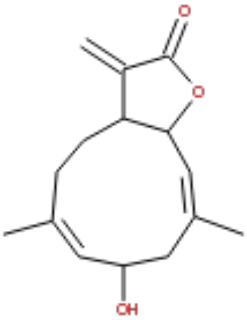

Cell ID	Cluster Center	Number of Compounds
10	0	14

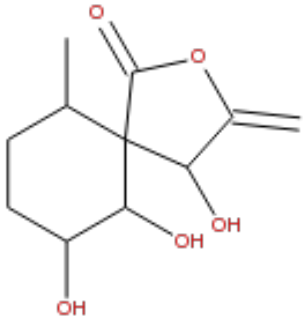

Cell ID	Cluster Center	Number of Compounds
12	1	3

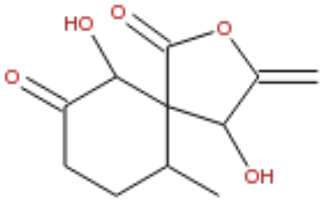

Cell ID	Cluster Center	Number of Compounds
12	0	3

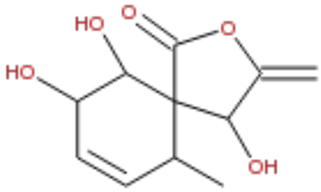

Cell ID	Cluster Center	Number of Compounds
12	0	3

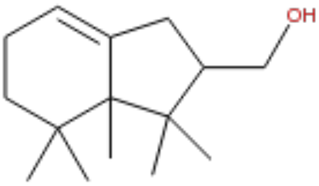

Cell ID	Cluster Center	Number of Compounds
14	1	13

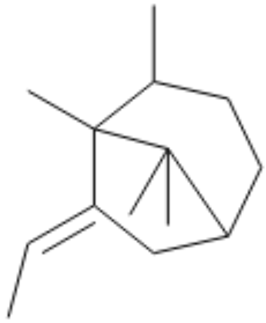

Cell ID	Cluster Center	Number of Compounds
14	0	13

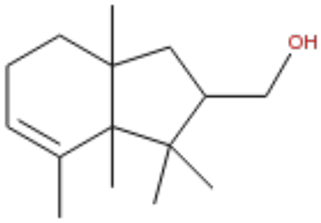

Cell ID	Cluster Center	Number of Compounds
14	0	13

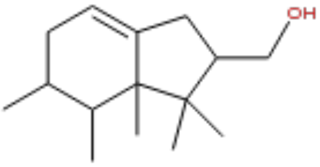

Cell ID	Cluster Center	Number of Compounds
14	0	13

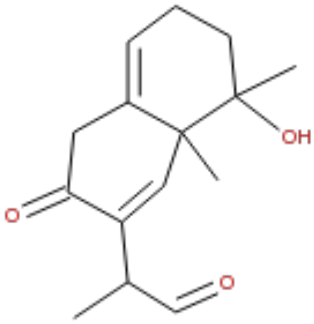

Cell ID	Cluster Center	Number of Compounds
14	0	13

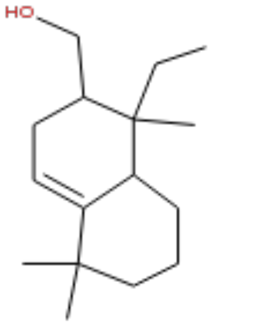

Cell ID	Cluster Center	Number of Compounds
14	0	13

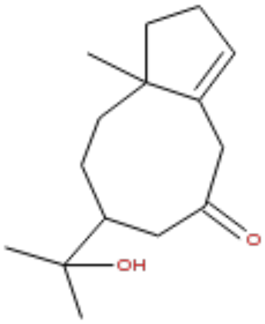

Cell ID	Cluster Center	Number of Compounds
14	0	13

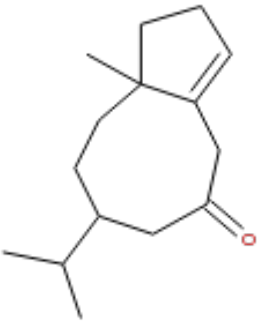

Cell ID	Cluster Center	Number of Compounds
14	0	13

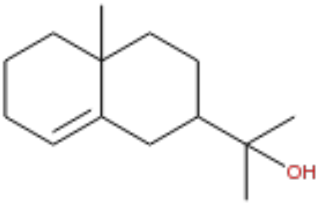

Cell ID	Cluster Center	Number of Compounds
14	0	13

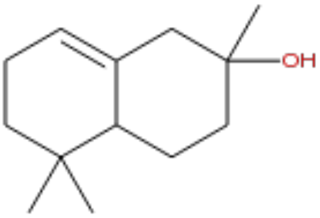

Cell ID	Cluster Center	Number of Compounds
14	0	13

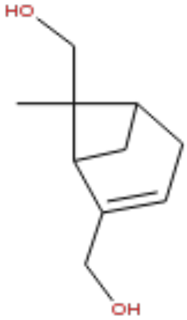

Cell ID	Cluster Center	Number of Compounds
14	0	13

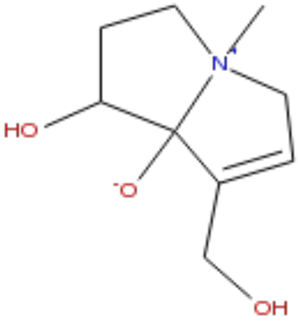

Cell ID	Cluster Center	Number of Compounds
14	0	13

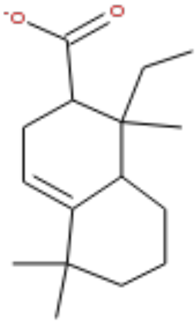

Cell ID	Cluster Center	Number of Compounds
14	0	13

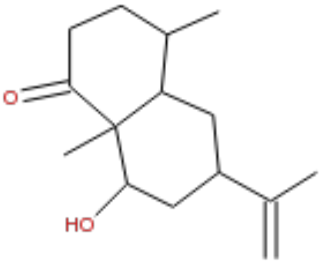

Cell ID	Cluster Center	Number of Compounds
15	1	2

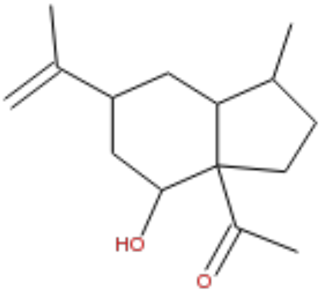

Cell ID	Cluster Center	Number of Compounds
15	0	2

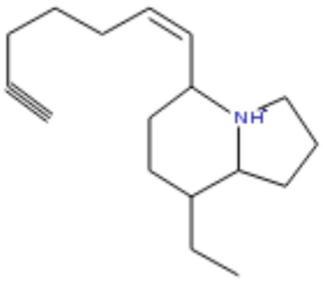

Cell ID	Cluster Center	Number of Compounds
18	1	3

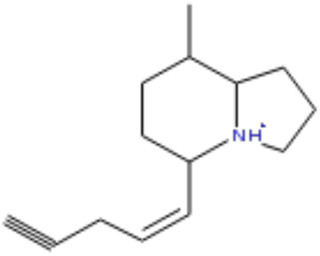

Cell ID	Cluster Center	Number of Compounds
18	0	3

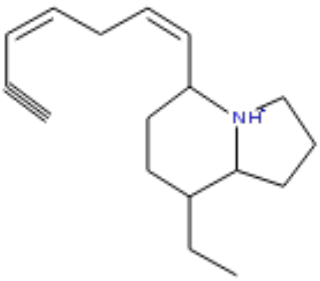

Cell ID	Cluster Center	Number of Compounds
18	0	3

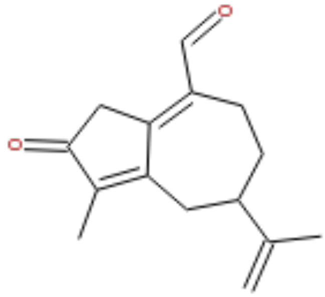

Cell ID	Cluster Center	Number of Compounds
25	1	7

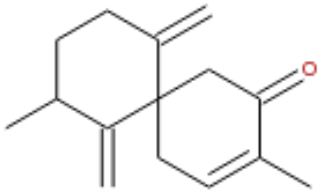

Cell ID	Cluster Center	Number of Compounds
25	0	7

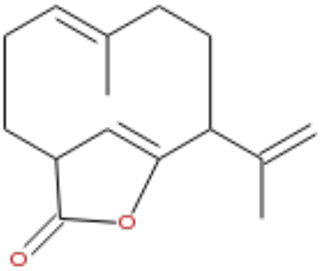

Cell ID	Cluster Center	Number of Compounds
25	0	7

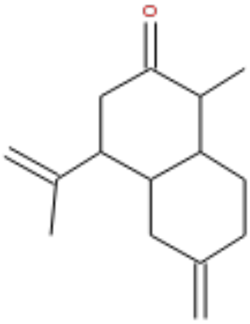

Cell ID	Cluster Center	Number of Compounds
25	0	7

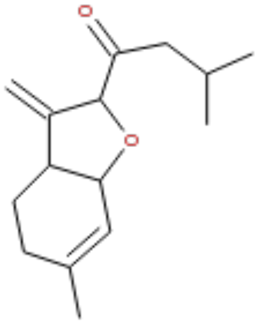

Cell ID	Cluster Center	Number of Compounds
25	0	7

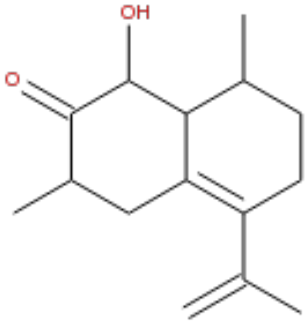

Cell ID	Cluster Center	Number of Compounds
25	0	7

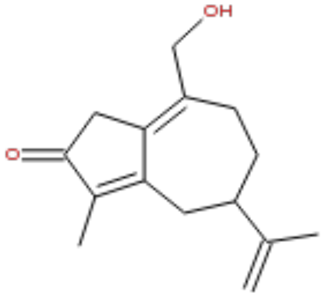

Cell ID	Cluster Center	Number of Compounds
25	0	7

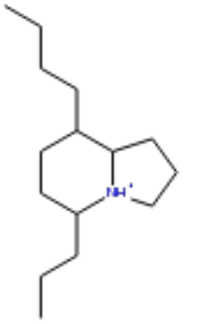

Cell ID	Cluster Center	Number of Compounds
26	1	5

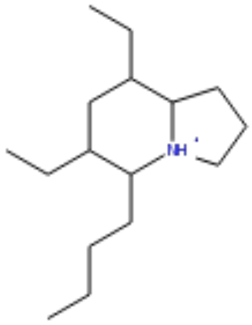

Cell ID	Cluster Center	Number of Compounds
26	0	5

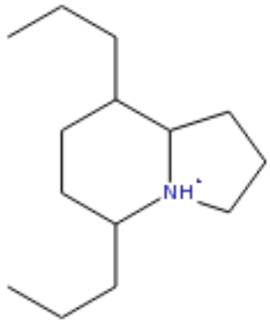

Cell ID	Cluster Center	Number of Compounds
26	0	5

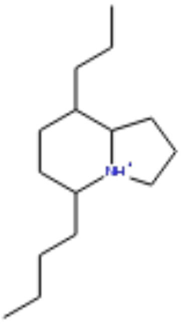

Cell ID	Cluster Center	Number of Compounds
26	0	5

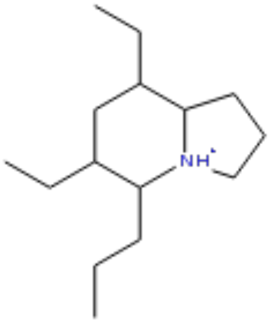

Cell ID	Cluster Center	Number of Compounds
26	0	5

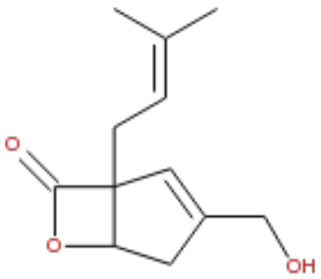

Cell ID	Cluster Center	Number of Compounds
28	1	4

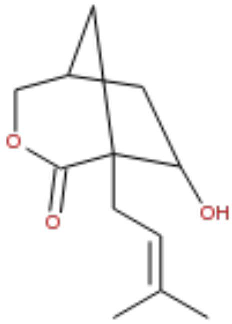

Cell ID	Cluster Center	Number of Compounds
28	0	4

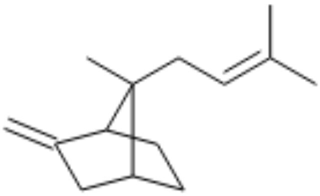

Cell ID	Cluster Center	Number of Compounds
28	0	4

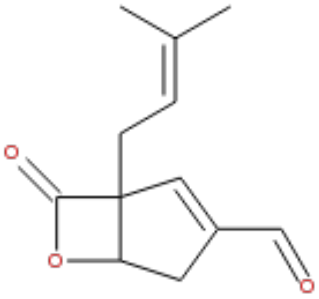

Cell ID	Cluster Center	Number of Compounds
28	0	4

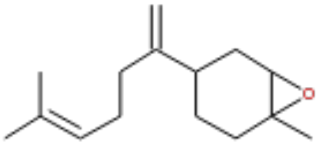

Cell ID	Cluster Center	Number of Compounds
30	1	2

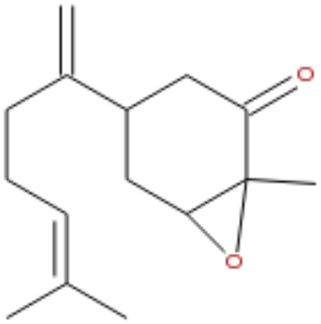

Cell ID	Cluster Center	Number of Compounds
30	0	2

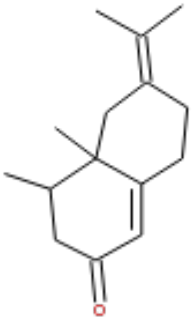

Cell ID	Cluster Center	Number of Compounds
31	1	5

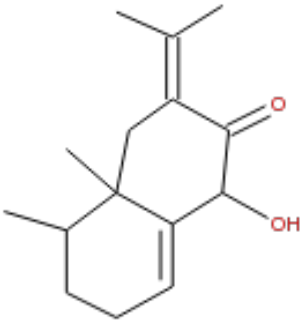

Cell ID	Cluster Center	Number of Compounds
31	0	5

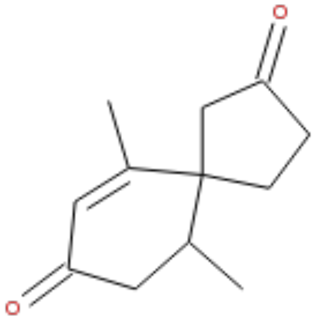

Cell ID	Cluster Center	Number of Compounds
31	0	5

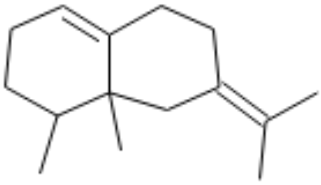

Cell ID	Cluster Center	Number of Compounds
31	0	5

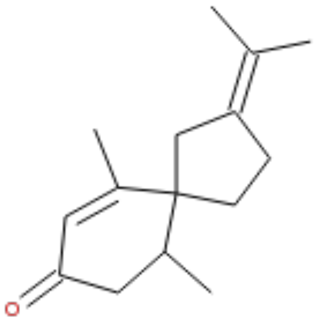

Cell ID	Cluster Center	Number of Compounds
31	0	5

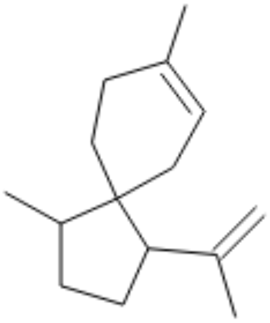

Cell ID	Cluster Center	Number of Compounds
32	1	4

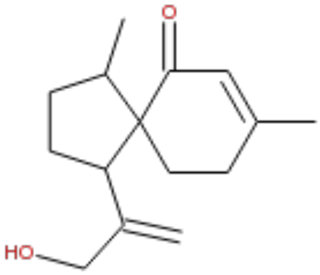

Cell ID	Cluster Center	Number of Compounds
32	0	4

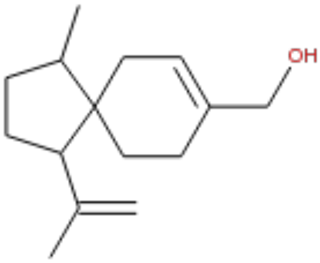

Cell ID	Cluster Center	Number of Compounds
32	0	4

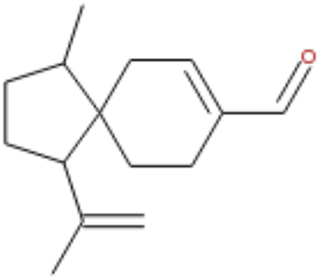

Cell ID	Cluster Center	Number of Compounds
32	0	4

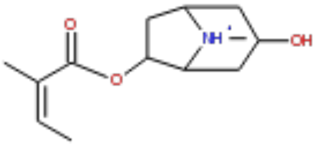

Cell ID	Cluster Center	Number of Compounds
33	1	4

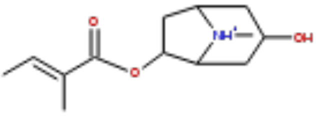

Cell ID	Cluster Center	Number of Compounds
33	0	4

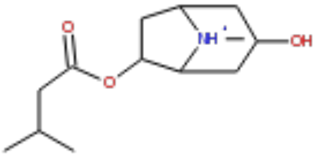

Cell ID	Cluster Center	Number of Compounds
33	0	4

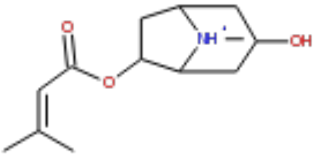

Cell ID	Cluster Center	Number of Compounds
33	0	4

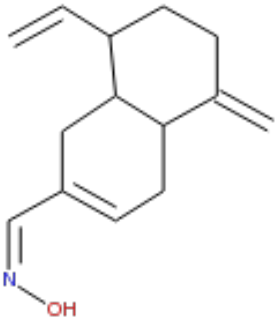

Cell ID	Cluster Center	Number of Compounds
34	1	2

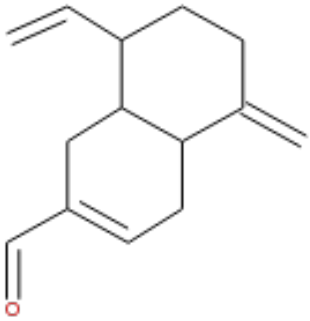

Cell ID	Cluster Center	Number of Compounds
34	0	2

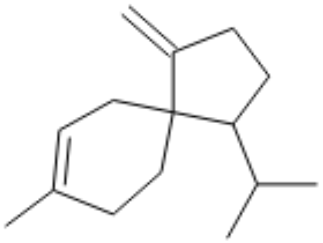

Cell ID	Cluster Center	Number of Compounds
35	1	7

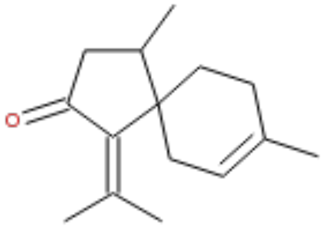

Cell ID	Cluster Center	Number of Compounds
35	0	7

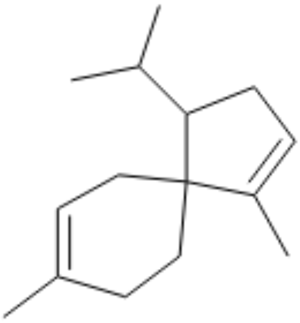

Cell ID	Cluster Center	Number of Compounds
35	0	7

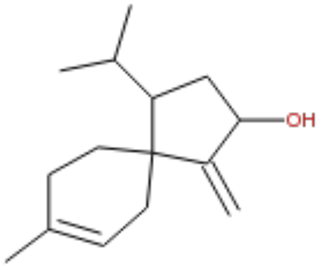

Cell ID	Cluster Center	Number of Compounds
35	0	7

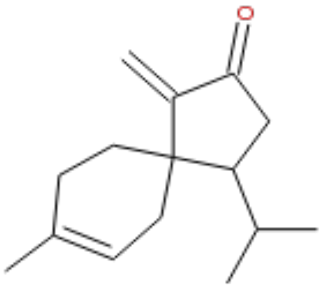

Cell ID	Cluster Center	Number of Compounds
35	0	7

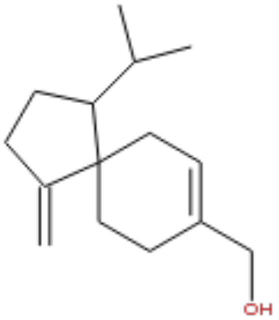

Cell ID	Cluster Center	Number of Compounds
35	0	7

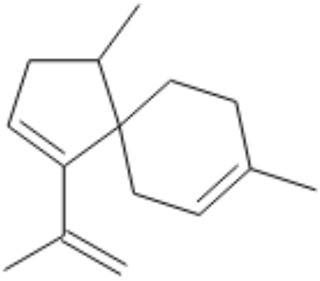

Cell ID	Cluster Center	Number of Compounds
35	0	7

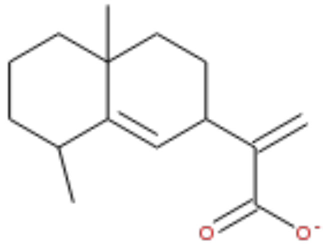

Cell ID	Cluster Center	Number of Compounds
36	1	4

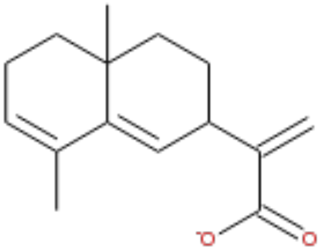

Cell ID	Cluster Center	Number of Compounds
36	0	4

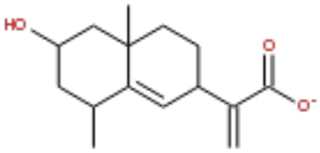

Cell ID	Cluster Center	Number of Compounds
36	0	4

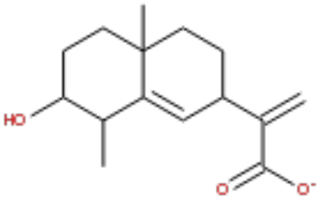

Cell ID	Cluster Center	Number of Compounds
36	0	4

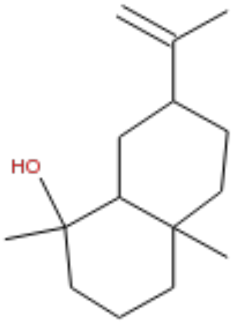

Cell ID	Cluster Center	Number of Compounds
37	1	15

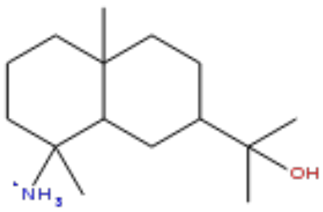

Cell ID	Cluster Center	Number of Compounds
37	0	15

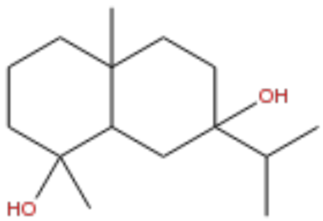

Cell ID	Cluster Center	Number of Compounds
37	0	15

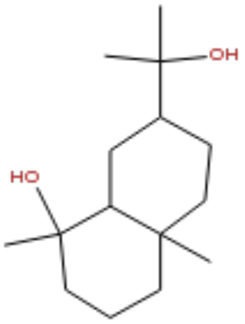

Cell ID	Cluster Center	Number of Compounds
37	0	15

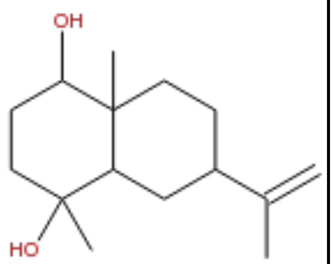

Cell ID	Cluster Center	Number of Compounds
37	0	15

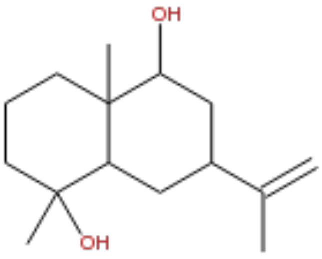

Cell ID	Cluster Center	Number of Compounds
37	0	15

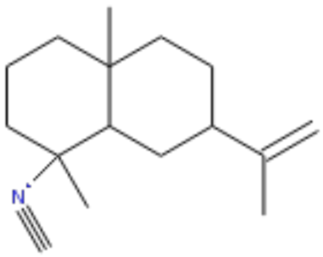

Cell ID	Cluster Center	Number of Compounds
37	0	15

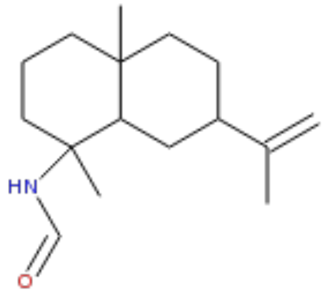

Cell ID	Cluster Center	Number of Compounds
37	0	15

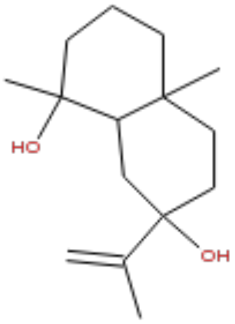

Cell ID	Cluster Center	Number of Compounds
37	0	15

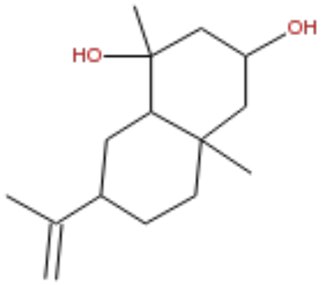

Cell ID	Cluster Center	Number of Compounds
37	0	15

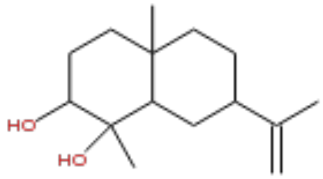

Cell ID	Cluster Center	Number of Compounds
37	0	15

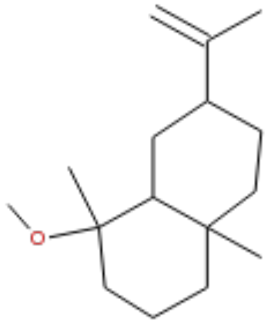

Cell ID	Cluster Center	Number of Compounds
37	0	15

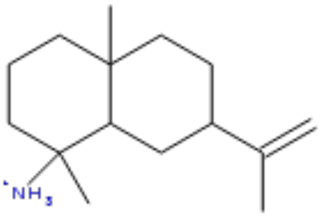

Cell ID	Cluster Center	Number of Compounds
37	0	15

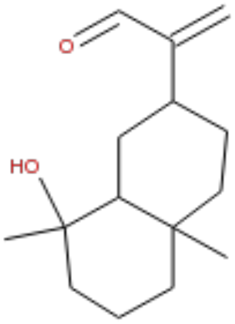

Cell ID	Cluster Center	Number of Compounds
37	0	15

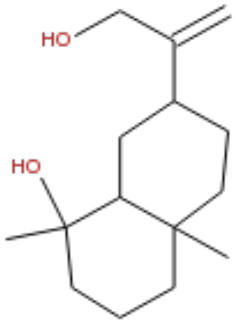

Cell ID	Cluster Center	Number of Compounds
37	0	15

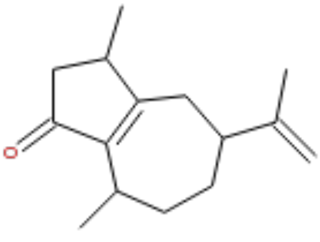

Cell ID	Cluster Center	Number of Compounds
38	1	7

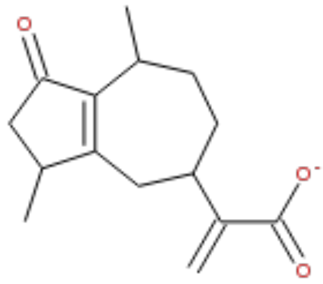

Cell ID	Cluster Center	Number of Compounds
38	0	7

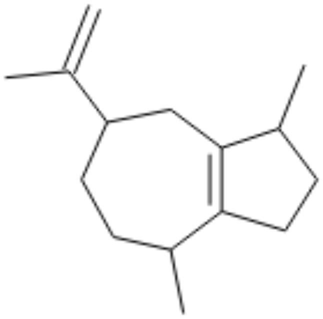

Cell ID	Cluster Center	Number of Compounds
38	0	7

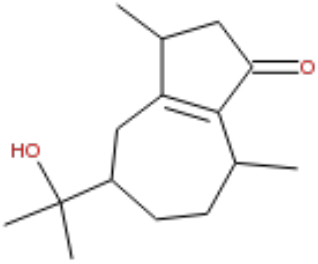

Cell ID	Cluster Center	Number of Compounds
38	0	7

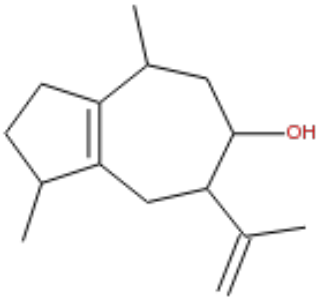

Cell ID	Cluster Center	Number of Compounds
38	0	7

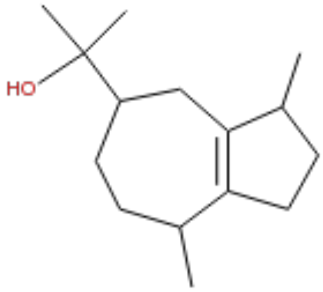

Cell ID	Cluster Center	Number of Compounds
38	0	7

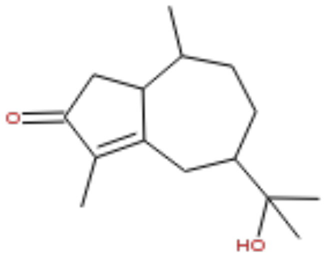

Cell ID	Cluster Center	Number of Compounds
38	0	7

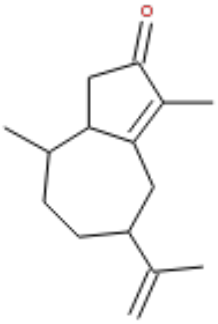

Cell ID	Cluster Center	Number of Compounds
39	1	11

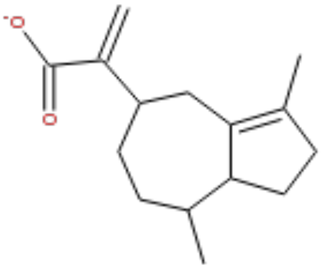

Cell ID	Cluster Center	Number of Compounds
39	0	11

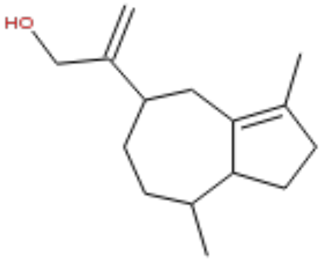

Cell ID	Cluster Center	Number of Compounds
39	0	11

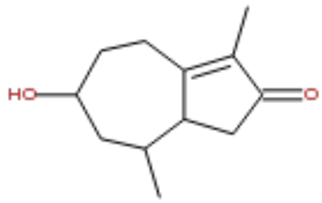

Cell ID	Cluster Center	Number of Compounds
39	0	11

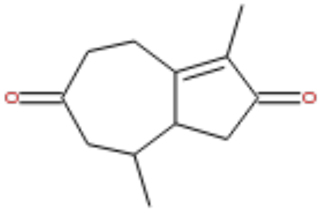

Cell ID	Cluster Center	Number of Compounds
39	0	11

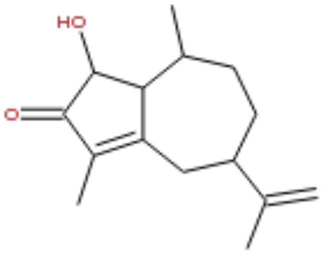

Cell ID	Cluster Center	Number of Compounds
39	0	11

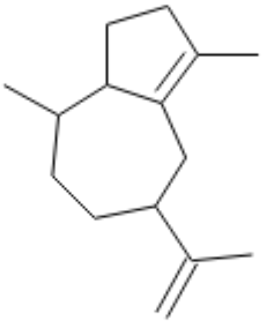

Cell ID	Cluster Center	Number of Compounds
39	0	11

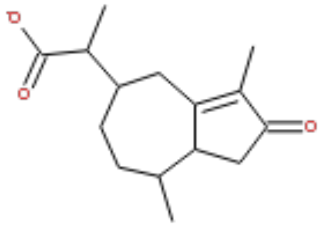

Cell ID	Cluster Center	Number of Compounds
39	0	11

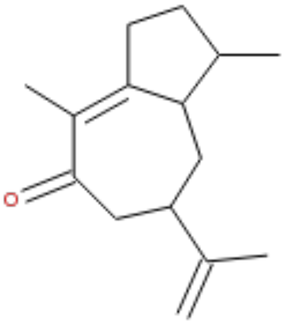

Cell ID	Cluster Center	Number of Compounds
39	0	11

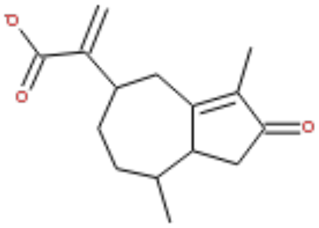

Cell ID	Cluster Center	Number of Compounds
39	0	11

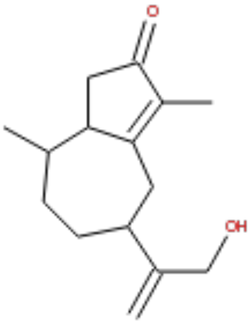

Cell ID	Cluster Center	Number of Compounds
39	0	11

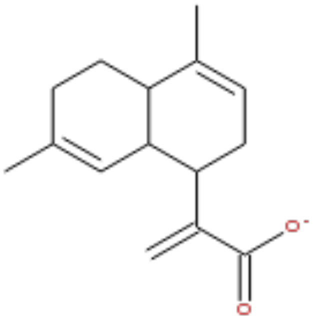

Cell ID	Cluster Center	Number of Compounds
40	1	5

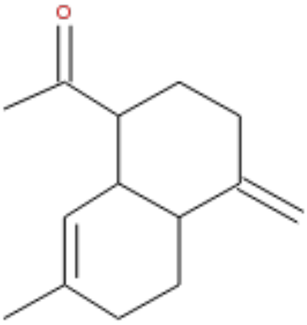

Cell ID	Cluster Center	Number of Compounds
40	0	5

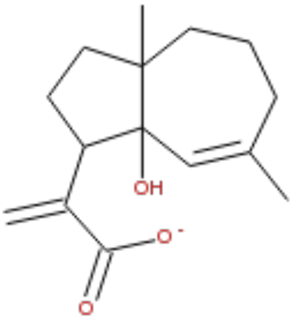

Cell ID	Cluster Center	Number of Compounds
40	0	5

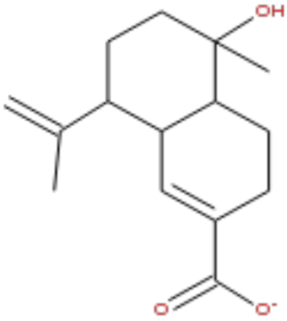

Cell ID	Cluster Center	Number of Compounds
40	0	5

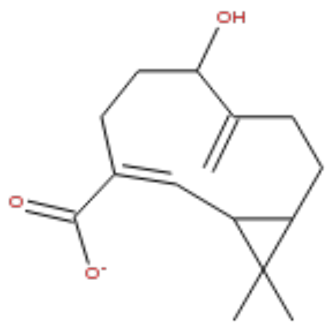

Cell ID	Cluster Center	Number of Compounds
40	0	5

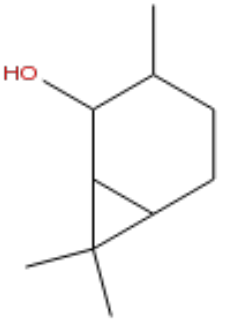

Cell ID	Cluster Center	Number of Compounds
41	1	1

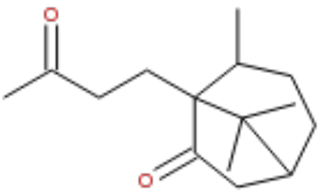

Cell ID	Cluster Center	Number of Compounds
42	1	1

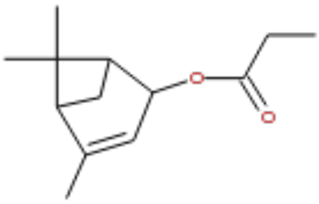

Cell ID	Cluster Center	Number of Compounds
43	1	18

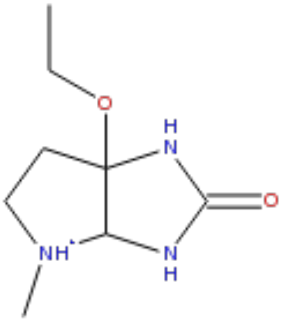

Cell ID	Cluster Center	Number of Compounds
43	0	18

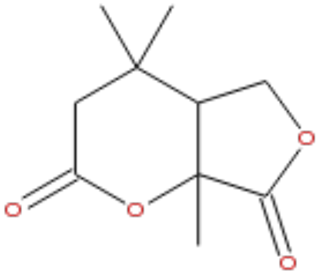

Cell ID	Cluster Center	Number of Compounds
43	0	18

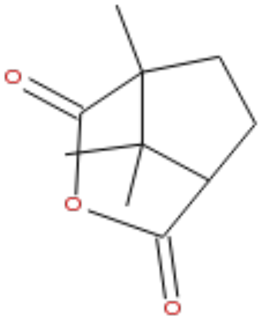

Cell ID	Cluster Center	Number of Compounds
43	0	18

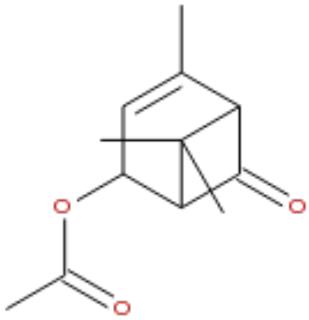

Cell ID	Cluster Center	Number of Compounds
43	0	18

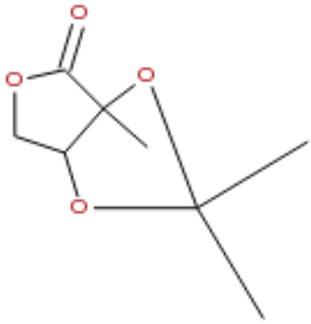

Cell ID	Cluster Center	Number of Compounds
43	0	18

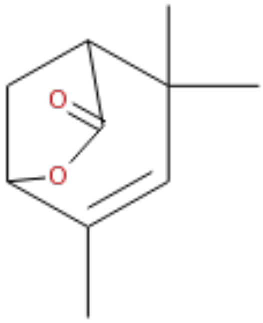

Cell ID	Cluster Center	Number of Compounds
43	0	18

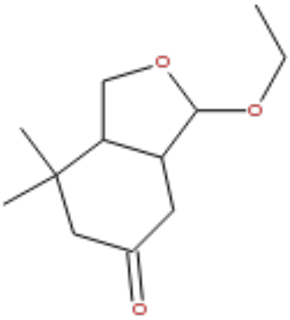

Cell ID	Cluster Center	Number of Compounds
43	0	18

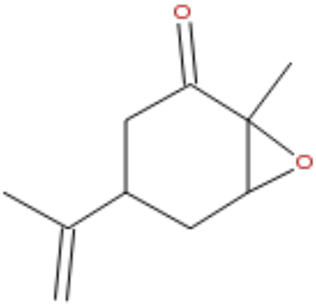

Cell ID	Cluster Center	Number of Compounds
43	0	18

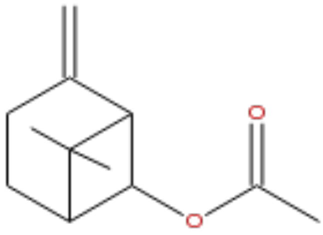

Cell ID	Cluster Center	Number of Compounds
43	0	18

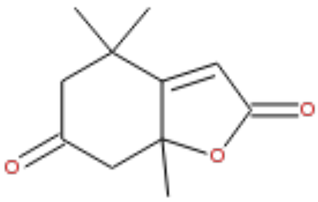

Cell ID	Cluster Center	Number of Compounds
43	0	18

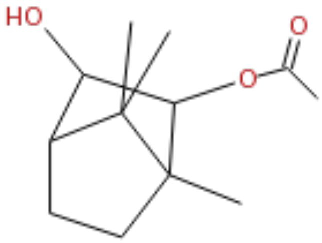

Cell ID	Cluster Center	Number of Compounds
43	0	18

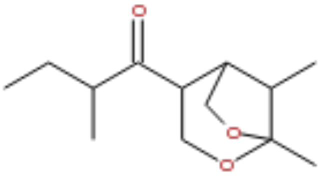

Cell ID	Cluster Center	Number of Compounds
43	0	18

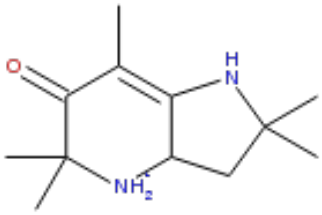

Cell ID	Cluster Center	Number of Compounds
43	0	18

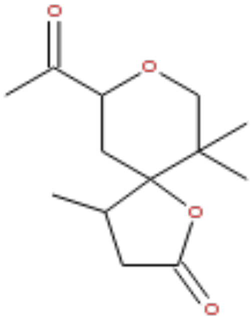

Cell ID	Cluster Center	Number of Compounds
43	0	18

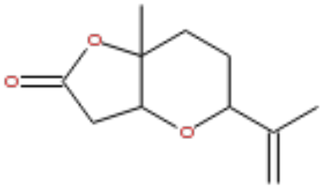

Cell ID	Cluster Center	Number of Compounds
43	0	18

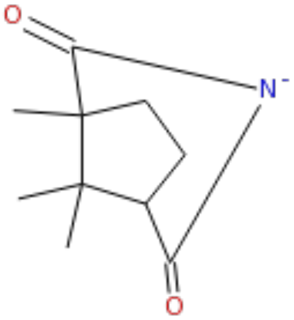

Cell ID	Cluster Center	Number of Compounds
43	0	18

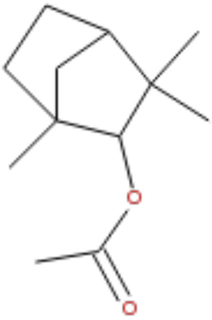

Cell ID	Cluster Center	Number of Compounds
43	0	18

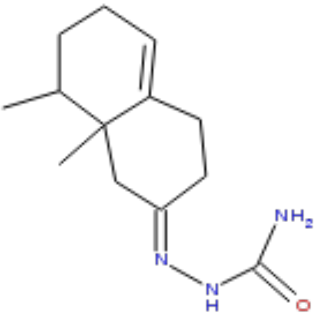

Cell ID	Cluster Center	Number of Compounds
44	1	2

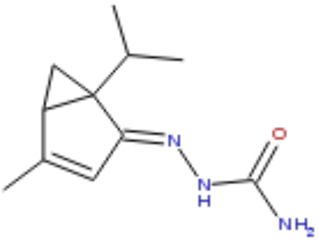

Cell ID	Cluster Center	Number of Compounds
44	0	2

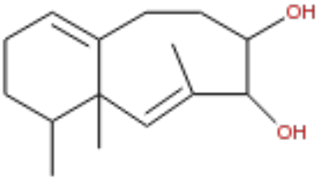

Cell ID	Cluster Center	Number of Compounds
45	1	5

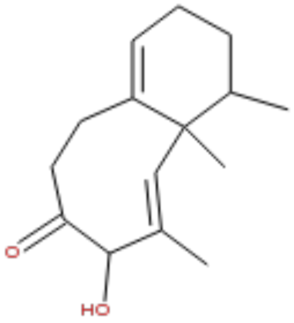

Cell ID	Cluster Center	Number of Compounds
45	0	5

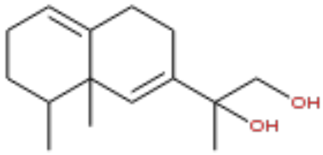

Cell ID	Cluster Center	Number of Compounds
45	0	5

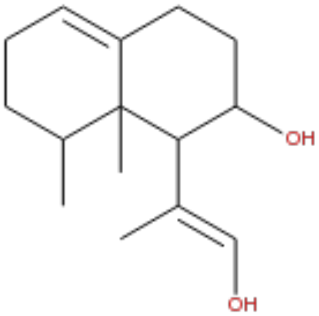

Cell ID	Cluster Center	Number of Compounds
45	0	5

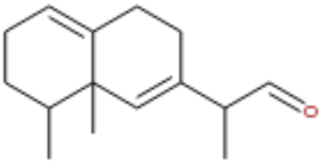

Cell ID	Cluster Center	Number of Compounds
45	0	5

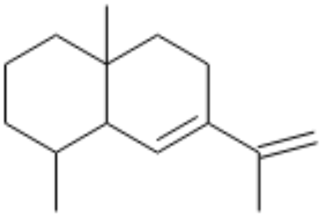

Cell ID	Cluster Center	Number of Compounds
51	1	6

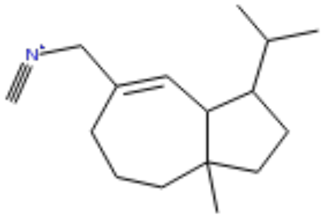

Cell ID	Cluster Center	Number of Compounds
51	0	6

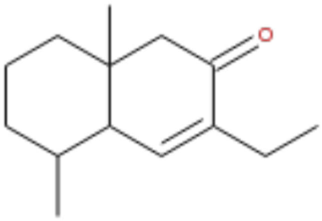

Cell ID	Cluster Center	Number of Compounds
51	0	6

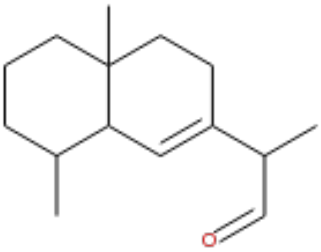

Cell ID	Cluster Center	Number of Compounds
51	0	6

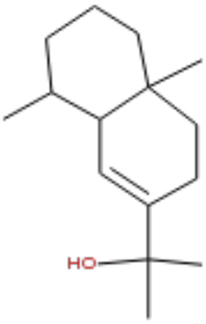

Cell ID	Cluster Center	Number of Compounds
51	0	6

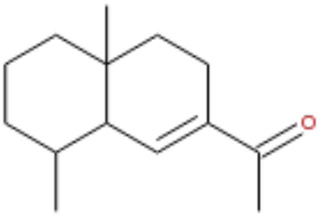

Cell ID	Cluster Center	Number of Compounds
51	0	6

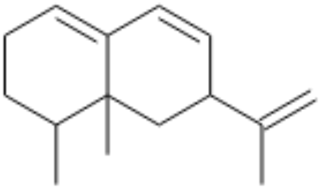

Cell ID	Cluster Center	Number of Compounds
53	1	5

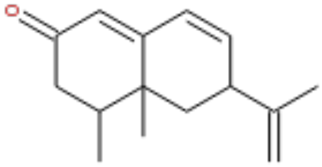

Cell ID	Cluster Center	Number of Compounds
53	0	5

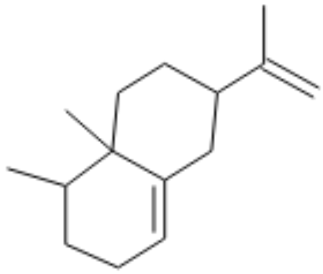

Cell ID	Cluster Center	Number of Compounds
53	0	5

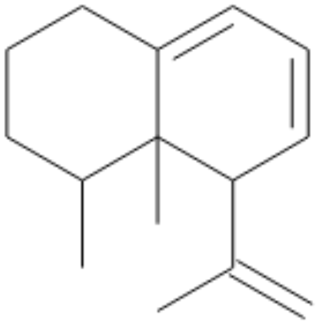

Cell ID	Cluster Center	Number of Compounds
53	0	5

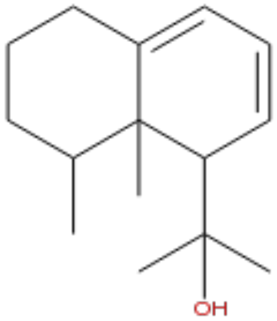

Cell ID	Cluster Center	Number of Compounds
53	0	5

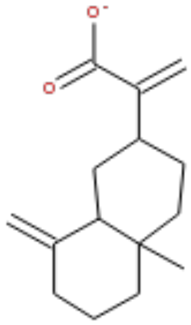

Cell ID	Cluster Center	Number of Compounds
54	1	14

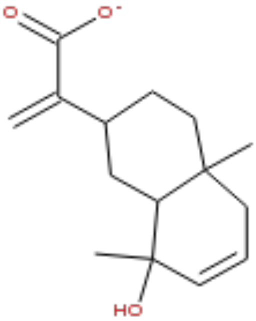

Cell ID	Cluster Center	Number of Compounds
54	0	14

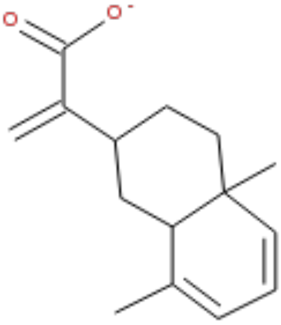

Cell ID	Cluster Center	Number of Compounds
54	0	14

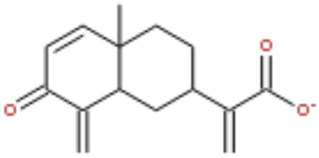

Cell ID	Cluster Center	Number of Compounds
54	0	14

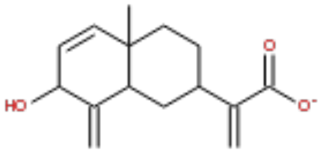

Cell ID	Cluster Center	Number of Compounds
54	0	14

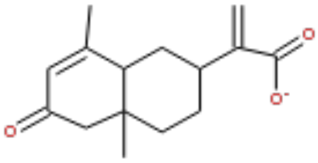

Cell ID	Cluster Center	Number of Compounds
54	0	14

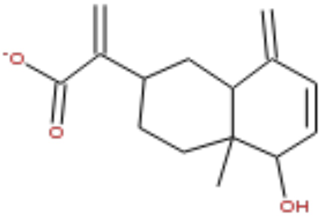

Cell ID	Cluster Center	Number of Compounds
54	0	14

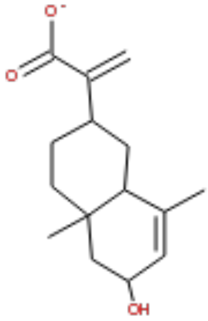

Cell ID	Cluster Center	Number of Compounds
54	0	14

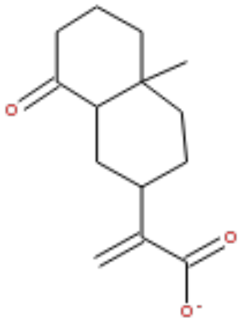

Cell ID	Cluster Center	Number of Compounds
54	0	14

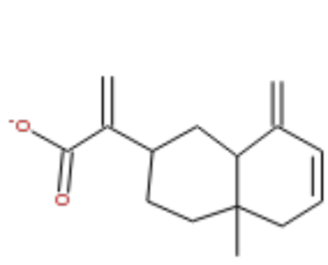

Cell ID	Cluster Center	Number of Compounds
54	0	14

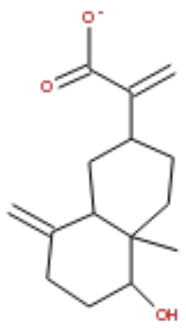

Cell ID	Cluster Center	Number of Compounds
54	0	14

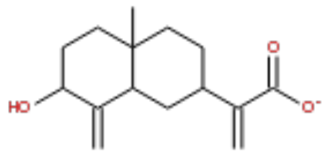

Cell ID	Cluster Center	Number of Compounds
54	0	14

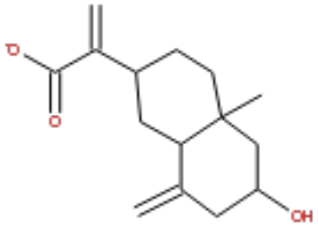

Cell ID	Cluster Center	Number of Compounds
54	0	14

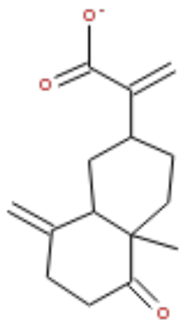

Cell ID	Cluster Center	Number of Compounds
54	0	14

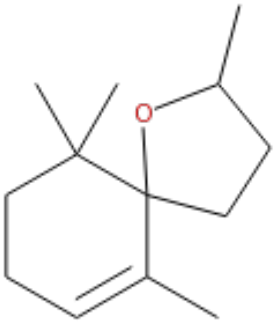

Cell ID	Cluster Center	Number of Compounds
55	1	2

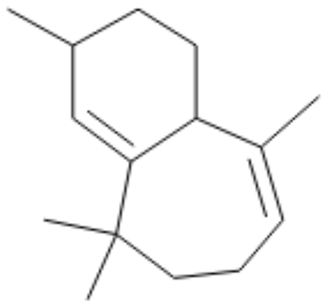

Cell ID	Cluster Center	Number of Compounds
55	0	2

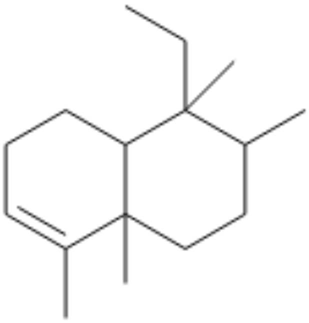

Cell ID	Cluster Center	Number of Compounds
56	1	4

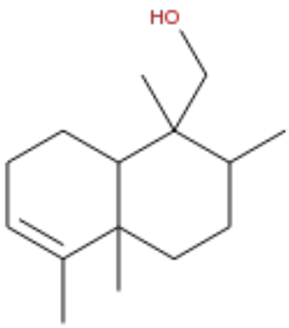

Cell ID	Cluster Center	Number of Compounds
56	0	4

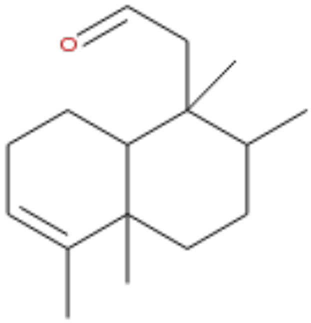

Cell ID	Cluster Center	Number of Compounds
56	0	4

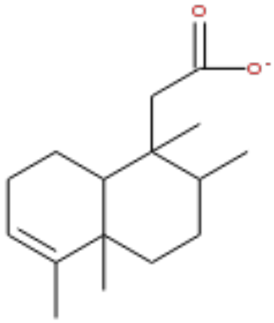

Cell ID	Cluster Center	Number of Compounds
56	0	4

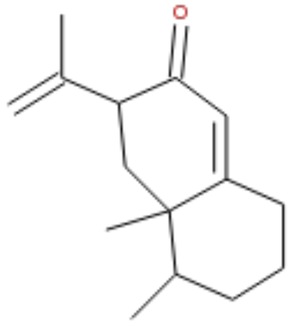

Cell ID	Cluster Center	Number of Compounds
57	1	11

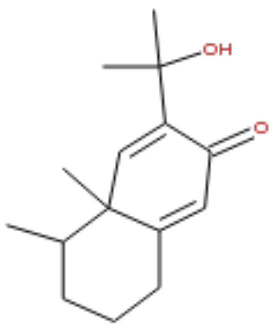

Cell ID	Cluster Center	Number of Compounds
57	0	11

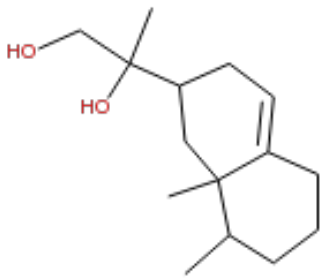

Cell ID	Cluster Center	Number of Compounds
57	0	11

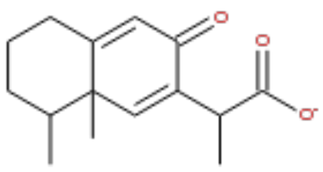

Cell ID	Cluster Center	Number of Compounds
57	0	11

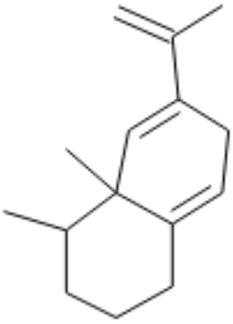

Cell ID	Cluster Center	Number of Compounds
57	0	11

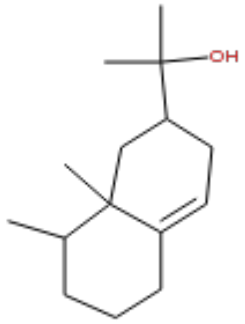

Cell ID	Cluster Center	Number of Compounds
57	0	11

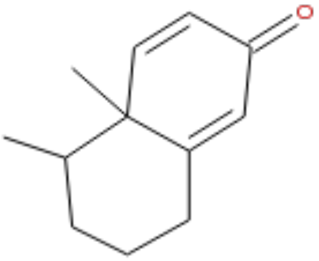

Cell ID	Cluster Center	Number of Compounds
57	0	11

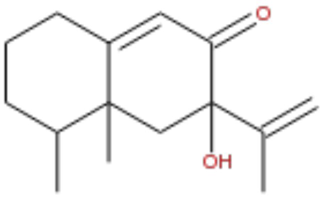

Cell ID	Cluster Center	Number of Compounds
57	0	11

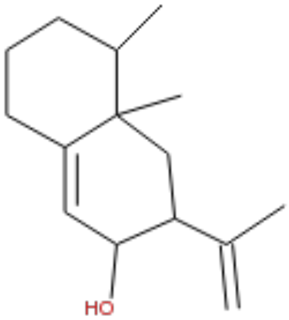

Cell ID	Cluster Center	Number of Compounds
57	0	11

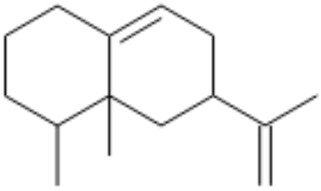

Cell ID	Cluster Center	Number of Compounds
57	0	11

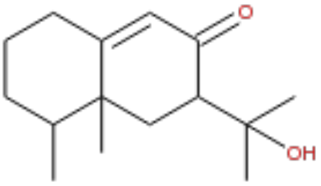

Cell ID	Cluster Center	Number of Compounds
57	0	11

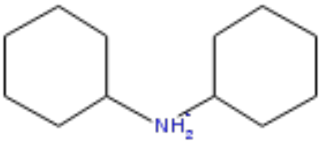

Cell ID	Cluster Center	Number of Compounds
58	1	10

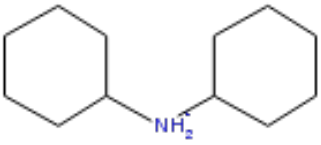

Cell ID	Cluster Center	Number of Compounds
58	0	10

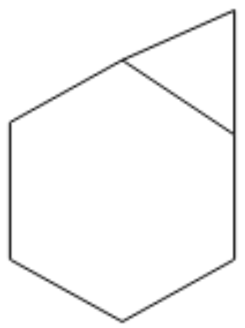

Cell ID	Cluster Center	Number of Compounds
58	0	10

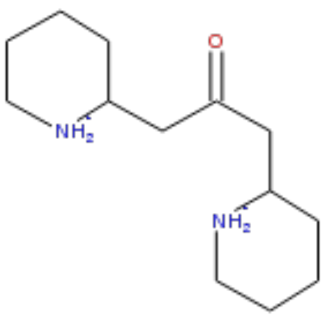

Cell ID	Cluster Center	Number of Compounds
58	0	10

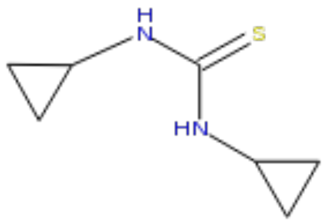

Cell ID	Cluster Center	Number of Compounds
58	0	10

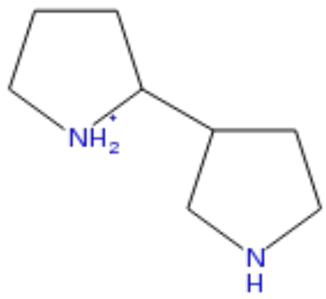

Cell ID	Cluster Center	Number of Compounds
58	0	10

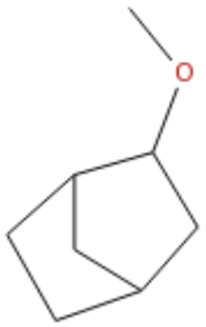

Cell ID	Cluster Center	Number of Compounds
58	0	10

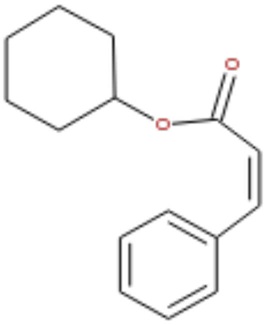

Cell ID	Cluster Center	Number of Compounds
58	0	10

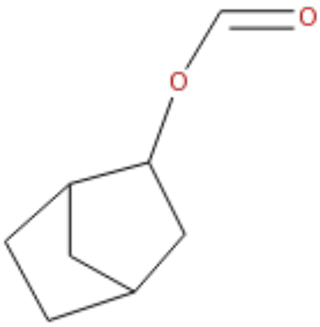

Cell ID	Cluster Center	Number of Compounds
58	0	10

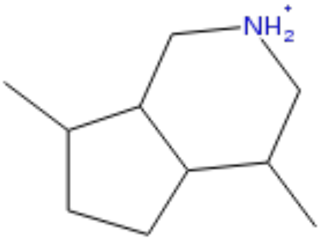

Cell ID	Cluster Center	Number of Compounds
58	0	10

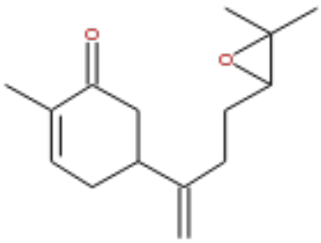

Cell ID	Cluster Center	Number of Compounds
59	1	1

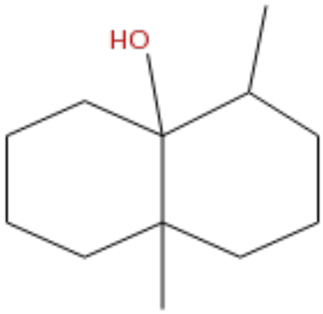

Cell ID	Cluster Center	Number of Compounds
60	1	21

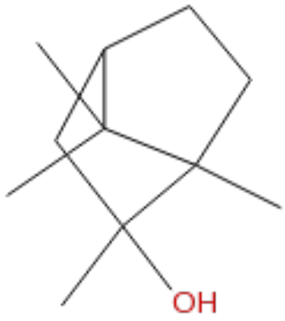

Cell ID	Cluster Center	Number of Compounds
60	0	21

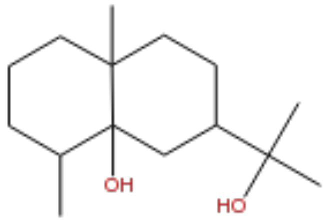

Cell ID	Cluster Center	Number of Compounds
60	0	21

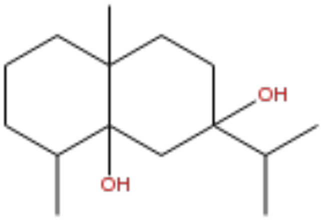

Cell ID	Cluster Center	Number of Compounds
60	0	21

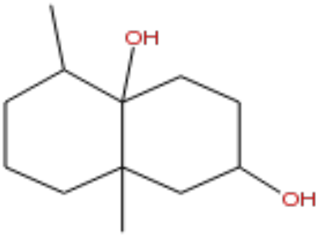

Cell ID	Cluster Center	Number of Compounds
60	0	21

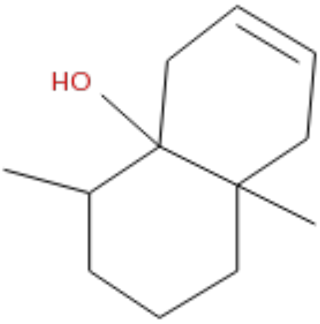

Cell ID	Cluster Center	Number of Compounds
60	0	21

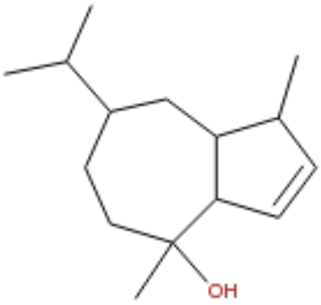

Cell ID	Cluster Center	Number of Compounds
60	0	21

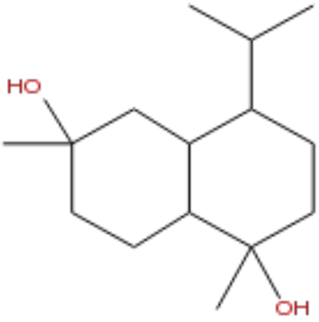

Cell ID	Cluster Center	Number of Compounds
60	0	21

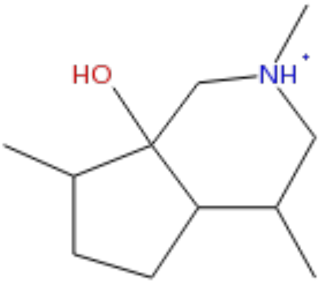

Cell ID	Cluster Center	Number of Compounds
60	0	21

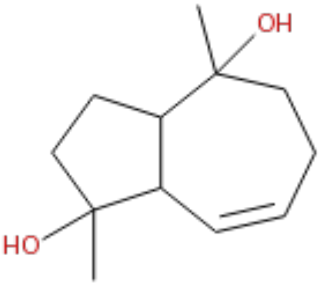

Cell ID	Cluster Center	Number of Compounds
60	0	21

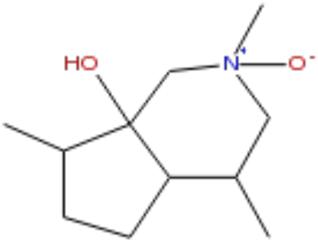

Cell ID	Cluster Center	Number of Compounds
60	0	21

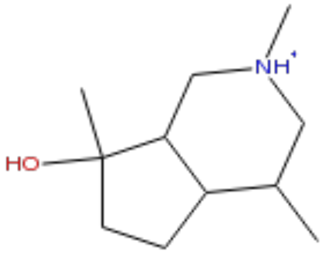

Cell ID	Cluster Center	Number of Compounds
60	0	21

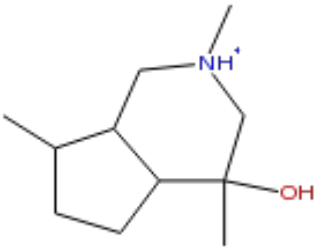

Cell ID	Cluster Center	Number of Compounds
60	0	21

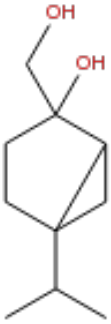

Cell ID	Cluster Center	Number of Compounds
60	0	21

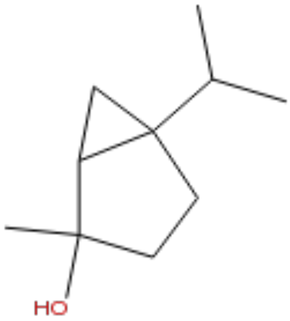

Cell ID	Cluster Center	Number of Compounds
60	0	21

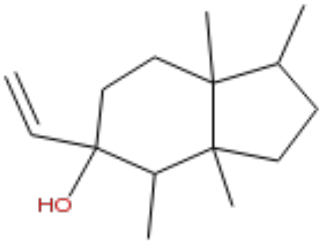

Cell ID	Cluster Center	Number of Compounds
60	0	21

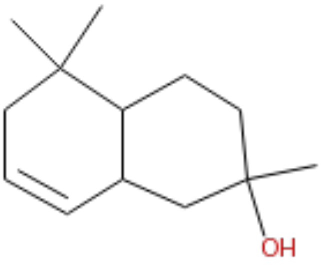

Cell ID	Cluster Center	Number of Compounds
60	0	21

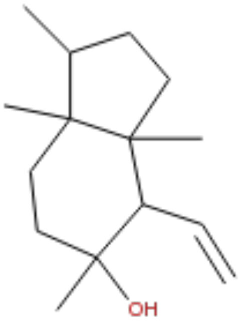

Cell ID	Cluster Center	Number of Compounds
60	0	21

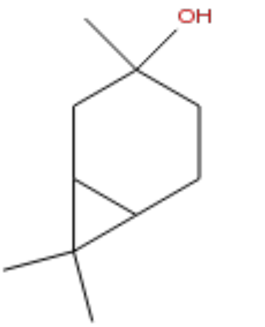

Cell ID	Cluster Center	Number of Compounds
60	0	21

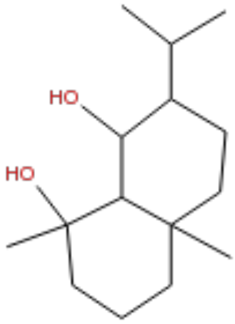

Cell ID	Cluster Center	Number of Compounds
60	0	21

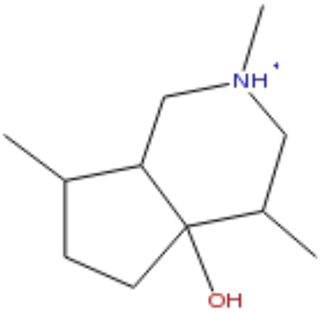

Cell ID	Cluster Center	Number of Compounds
60	0	21

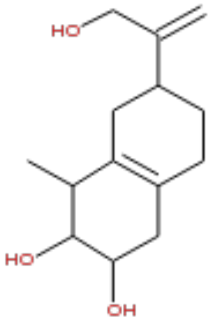

Cell ID	Cluster Center	Number of Compounds
61	1	3

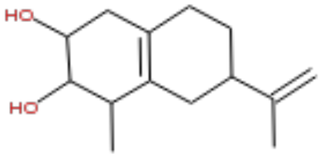

Cell ID	Cluster Center	Number of Compounds
61	0	3

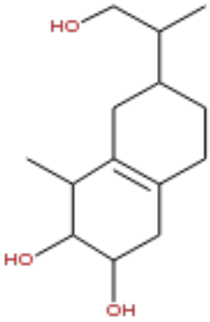

Cell ID	Cluster Center	Number of Compounds
61	0	3

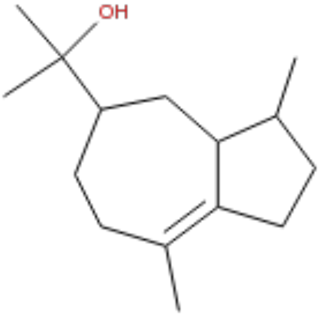

Cell ID	Cluster Center	Number of Compounds
62	1	14

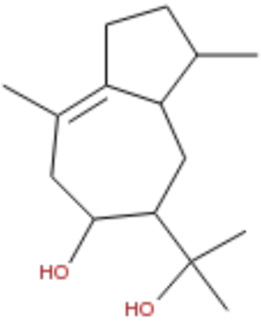

Cell ID	Cluster Center	Number of Compounds
62	0	14

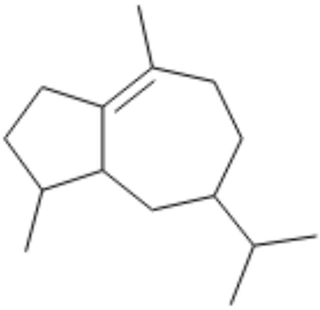

Cell ID	Cluster Center	Number of Compounds
62	0	14

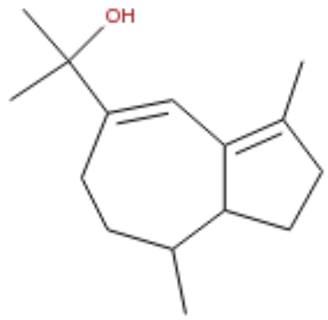

Cell ID	Cluster Center	Number of Compounds
62	0	14

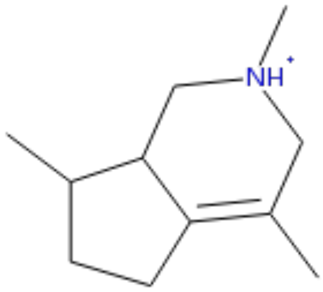

Cell ID	Cluster Center	Number of Compounds
62	0	14

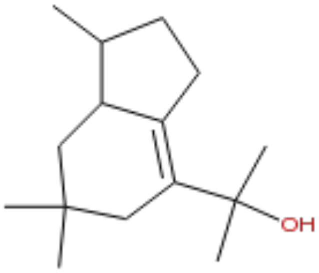

Cell ID	Cluster Center	Number of Compounds
62	0	14

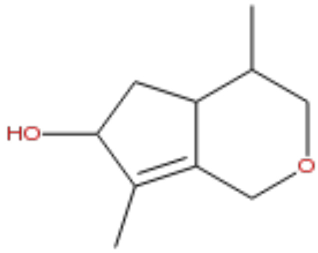

Cell ID	Cluster Center	Number of Compounds
62	0	14

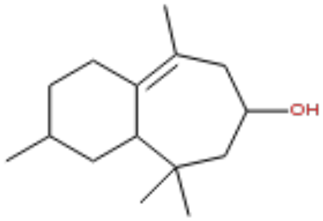

Cell ID	Cluster Center	Number of Compounds
62	0	14

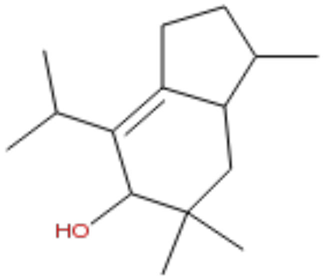

Cell ID	Cluster Center	Number of Compounds
62	0	14

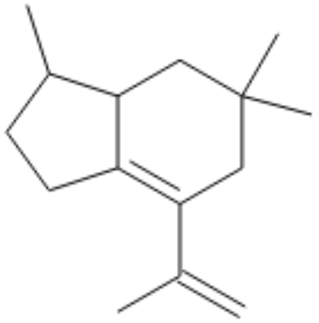

Cell ID	Cluster Center	Number of Compounds
62	0	14

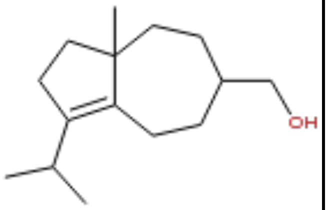

Cell ID	Cluster Center	Number of Compounds
62	0	14

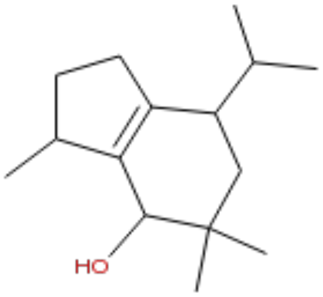

Cell ID	Cluster Center	Number of Compounds
62	0	14

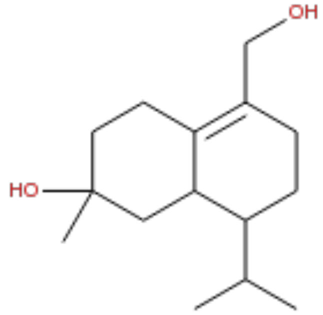

Cell ID	Cluster Center	Number of Compounds
62	0	14

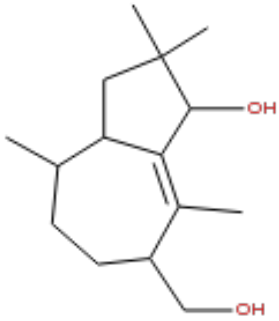

Cell ID	Cluster Center	Number of Compounds
62	0	14

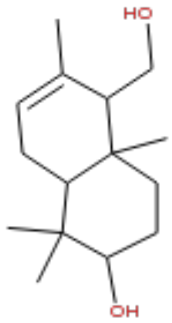

Cell ID	Cluster Center	Number of Compounds
63	1	6

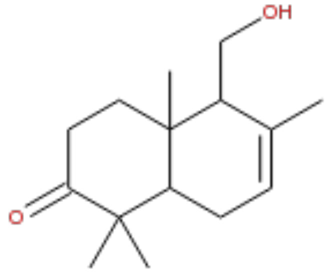

Cell ID	Cluster Center	Number of Compounds
63	0	6

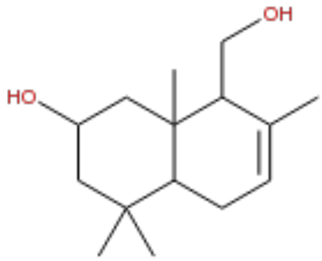

Cell ID	Cluster Center	Number of Compounds
63	0	6

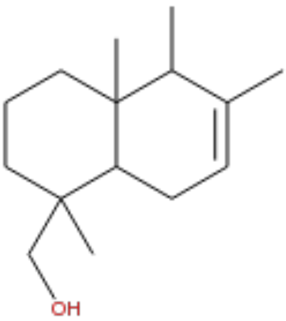

Cell ID	Cluster Center	Number of Compounds
63	0	6

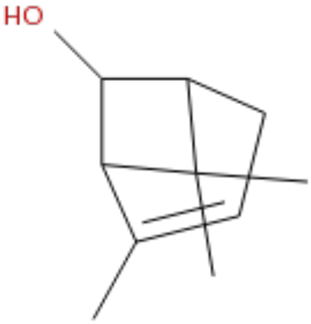

Cell ID	Cluster Center	Number of Compounds
63	0	6

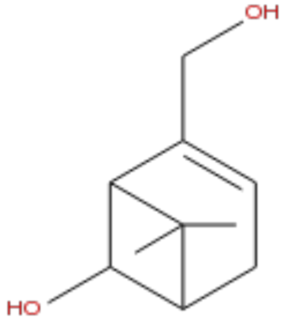

Cell ID	Cluster Center	Number of Compounds
63	0	6

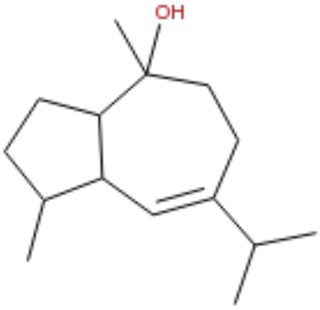

Cell ID	Cluster Center	Number of Compounds
64	1	32

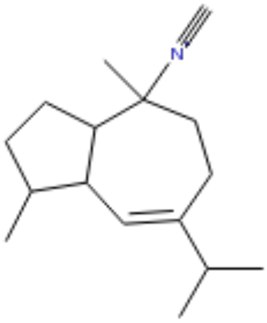

Cell ID	Cluster Center	Number of Compounds
64	0	32

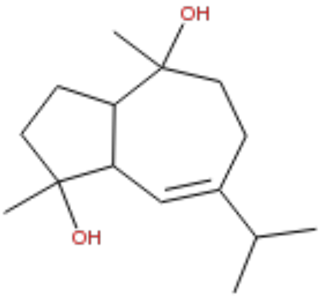

Cell ID	Cluster Center	Number of Compounds
64	0	32

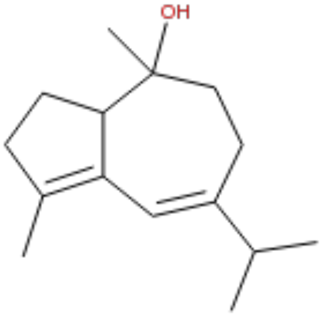

Cell ID	Cluster Center	Number of Compounds
64	0	32

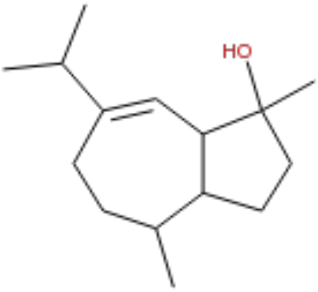

Cell ID	Cluster Center	Number of Compounds
64	0	32

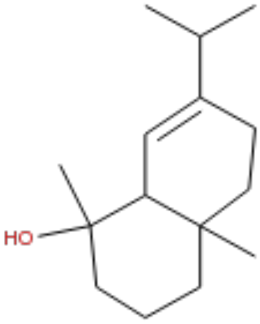

Cell ID	Cluster Center	Number of Compounds
64	0	32

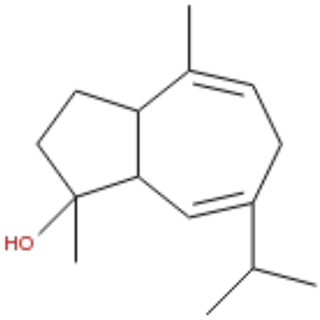

Cell ID	Cluster Center	Number of Compounds
64	0	32

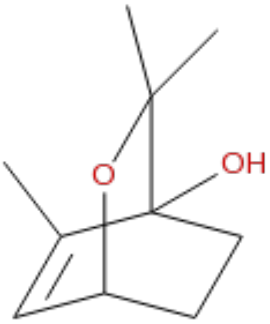

Cell ID	Cluster Center	Number of Compounds
64	0	32

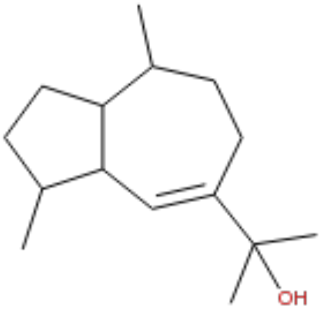

Cell ID	Cluster Center	Number of Compounds
64	0	32

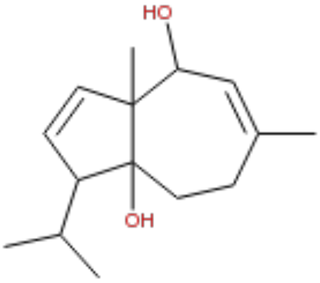

Cell ID	Cluster Center	Number of Compounds
64	0	32

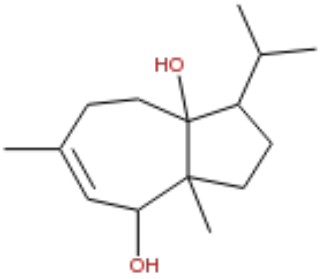

Cell ID	Cluster Center	Number of Compounds
64	0	32

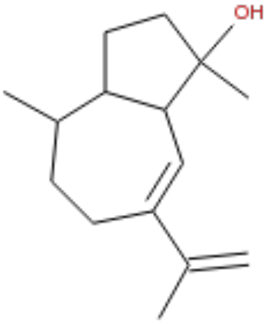

Cell ID	Cluster Center	Number of Compounds
64	0	32

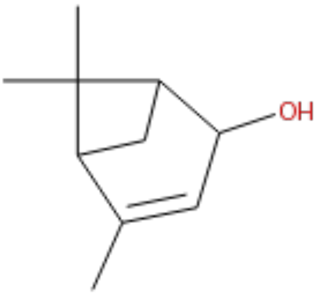

Cell ID	Cluster Center	Number of Compounds
64	0	32

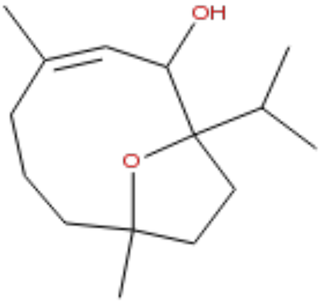

Cell ID	Cluster Center	Number of Compounds
64	0	32

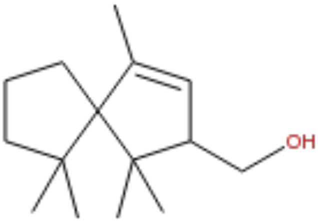

Cell ID	Cluster Center	Number of Compounds
64	0	32

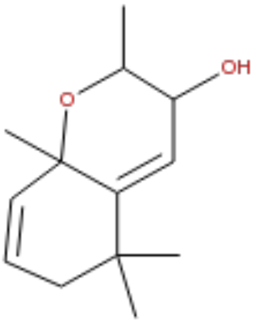

Cell ID	Cluster Center	Number of Compounds
64	0	32

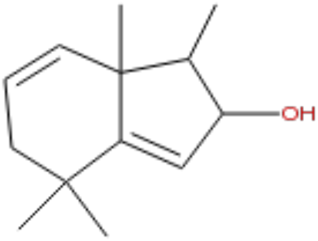

Cell ID	Cluster Center	Number of Compounds
64	0	32

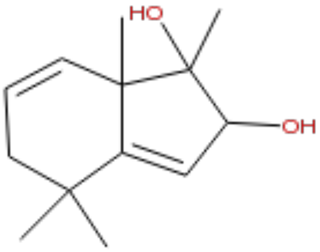

Cell ID	Cluster Center	Number of Compounds
64	0	32

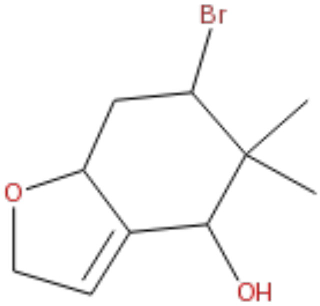

Cell ID	Cluster Center	Number of Compounds
64	0	32

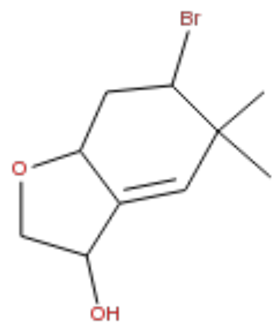

Cell ID	Cluster Center	Number of Compounds
64	0	32

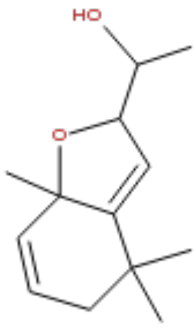

Cell ID	Cluster Center	Number of Compounds
64	0	32

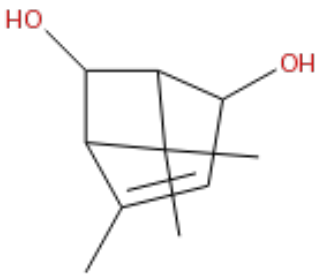

Cell ID	Cluster Center	Number of Compounds
64	0	32

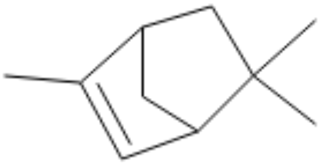

Cell ID	Cluster Center	Number of Compounds
64	0	32

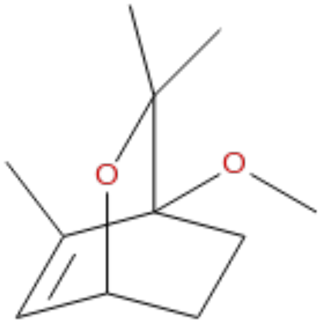

Cell ID	Cluster Center	Number of Compounds
64	0	32

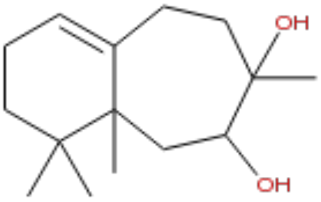

Cell ID	Cluster Center	Number of Compounds
64	0	32

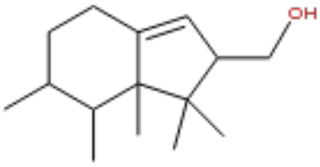

Cell ID	Cluster Center	Number of Compounds
64	0	32

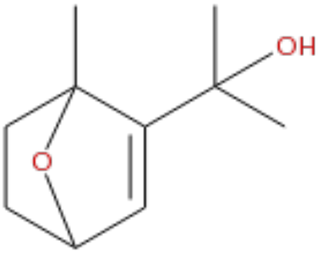

Cell ID	Cluster Center	Number of Compounds
64	0	32

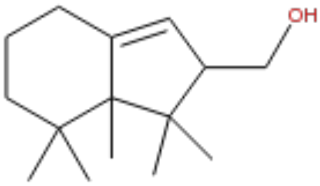

Cell ID	Cluster Center	Number of Compounds
64	0	32

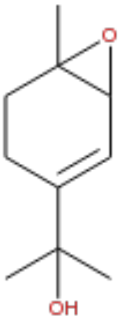

Cell ID	Cluster Center	Number of Compounds
64	0	32

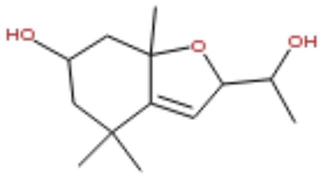

Cell ID	Cluster Center	Number of Compounds
64	0	32

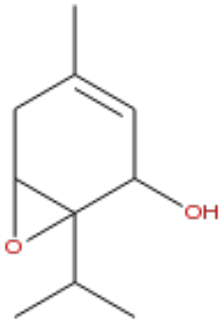

Cell ID	Cluster Center	Number of Compounds
64	0	32

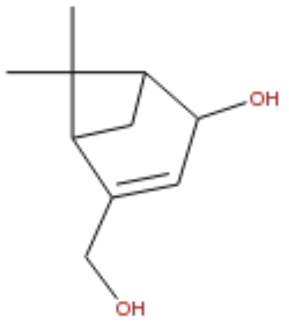

Cell ID	Cluster Center	Number of Compounds
64	0	32

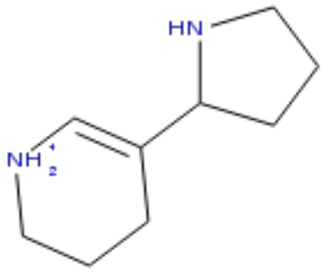

Cell ID	Cluster Center	Number of Compounds
65	1	7

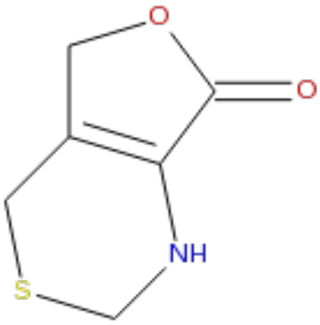

Cell ID	Cluster Center	Number of Compounds
65	0	7

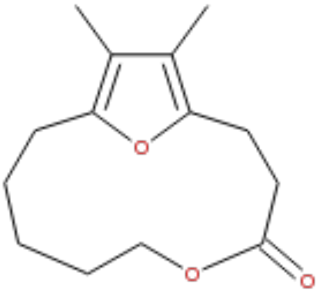

Cell ID	Cluster Center	Number of Compounds
65	0	7

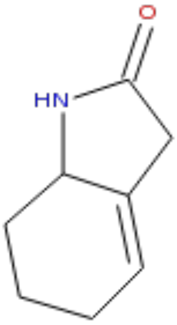

Cell ID	Cluster Center	Number of Compounds
65	0	7

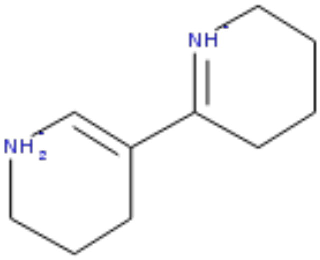

Cell ID	Cluster Center	Number of Compounds
65	0	7

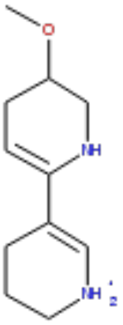

Cell ID	Cluster Center	Number of Compounds
65	0	7

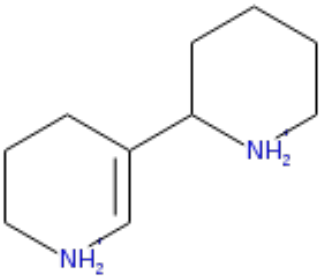

Cell ID	Cluster Center	Number of Compounds
65	0	7

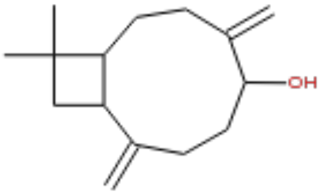

Cell ID	Cluster Center	Number of Compounds
67	1	5

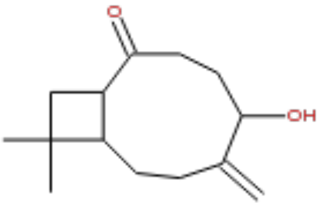

Cell ID	Cluster Center	Number of Compounds
67	0	5

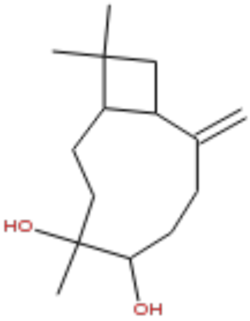

Cell ID	Cluster Center	Number of Compounds
67	0	5

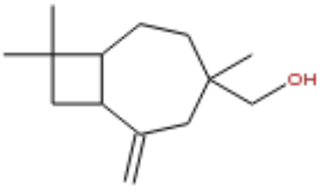

Cell ID	Cluster Center	Number of Compounds
67	0	5

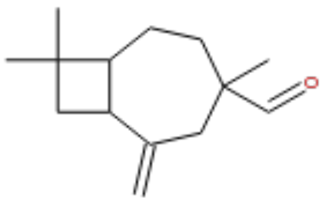

Cell ID	Cluster Center	Number of Compounds
67	0	5

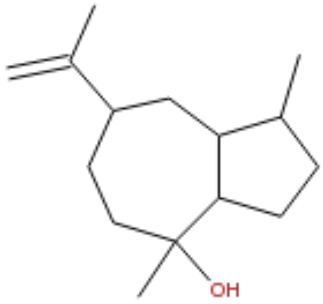

Cell ID	Cluster Center	Number of Compounds
68	1	5

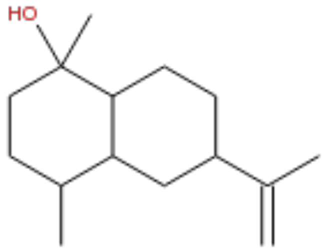

Cell ID	Cluster Center	Number of Compounds
68	0	5

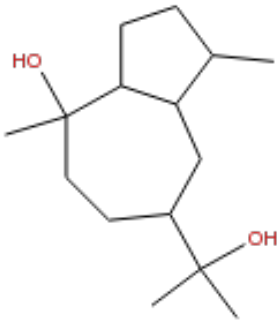

Cell ID	Cluster Center	Number of Compounds
68	0	5

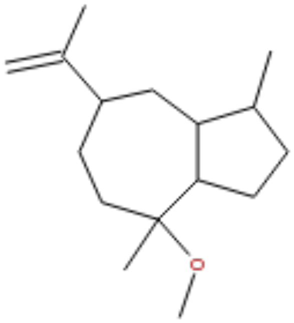

Cell ID	Cluster Center	Number of Compounds
68	0	5

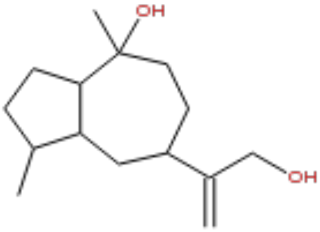

Cell ID	Cluster Center	Number of Compounds
68	0	5

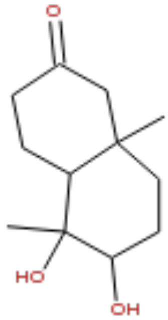

Cell ID	Cluster Center	Number of Compounds
69	1	25

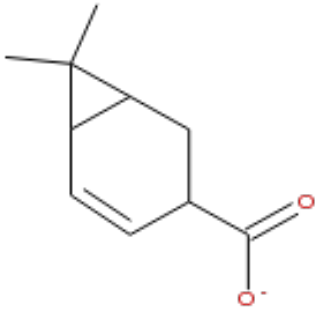

Cell ID	Cluster Center	Number of Compounds
69	0	25

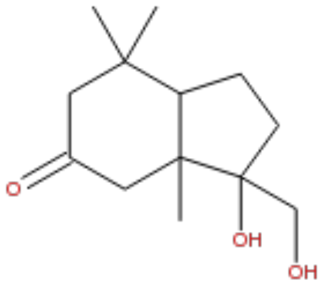

Cell ID	Cluster Center	Number of Compounds
69	0	25

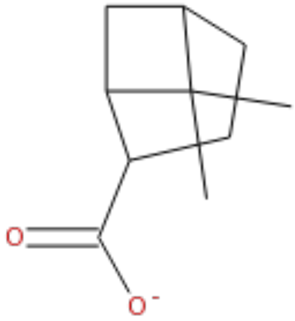

Cell ID	Cluster Center	Number of Compounds
69	0	25

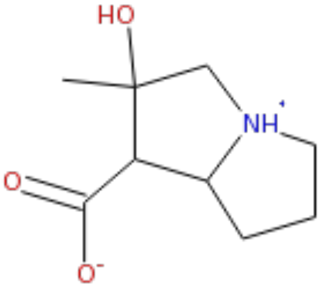

Cell ID	Cluster Center	Number of Compounds
69	0	25

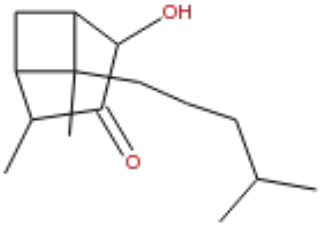

Cell ID	Cluster Center	Number of Compounds
69	0	25

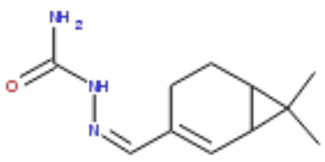

Cell ID	Cluster Center	Number of Compounds
69	0	25

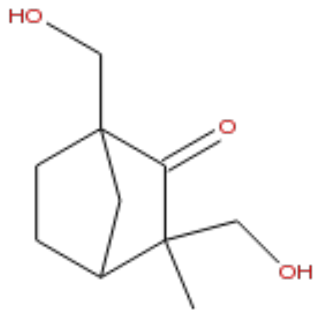

Cell ID	Cluster Center	Number of Compounds
69	0	25

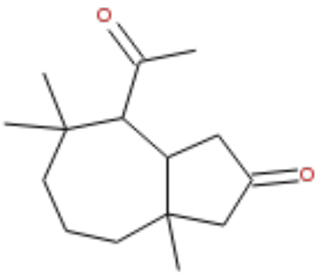

Cell ID	Cluster Center	Number of Compounds
69	0	25

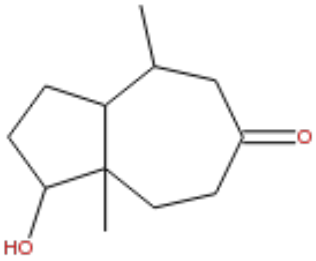

Cell ID	Cluster Center	Number of Compounds
69	0	25

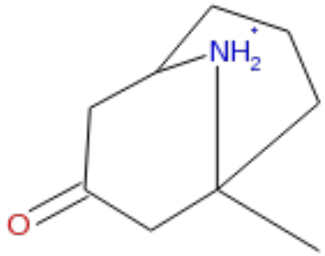

Cell ID	Cluster Center	Number of Compounds
69	0	25

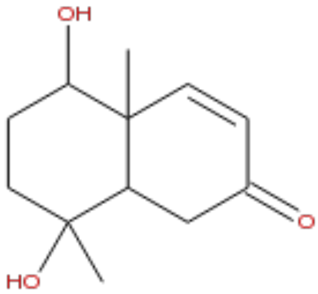

Cell ID	Cluster Center	Number of Compounds
69	0	25

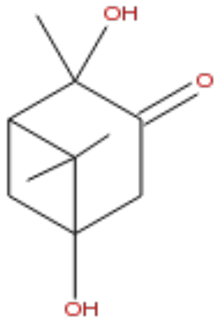

Cell ID	Cluster Center	Number of Compounds
69	0	25

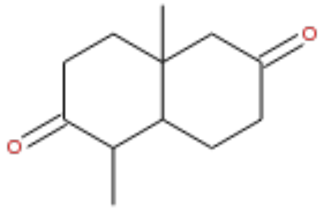

Cell ID	Cluster Center	Number of Compounds
69	0	25

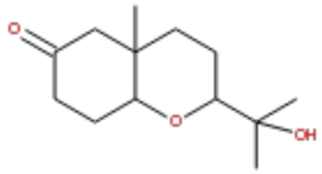

Cell ID	Cluster Center	Number of Compounds
69	0	25

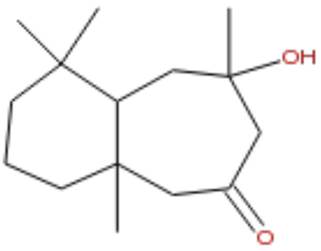

Cell ID	Cluster Center	Number of Compounds
69	0	25

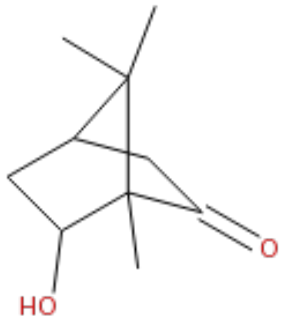

Cell ID	Cluster Center	Number of Compounds
69	0	25

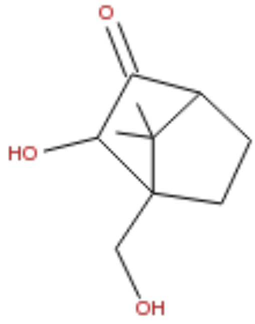

Cell ID	Cluster Center	Number of Compounds
69	0	25

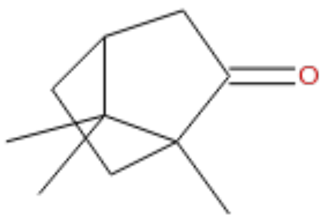

Cell ID	Cluster Center	Number of Compounds
69	0	25

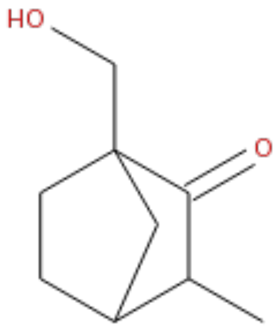

Cell ID	Cluster Center	Number of Compounds
69	0	25

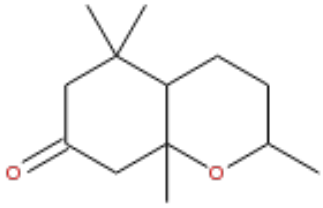

Cell ID	Cluster Center	Number of Compounds
69	0	25

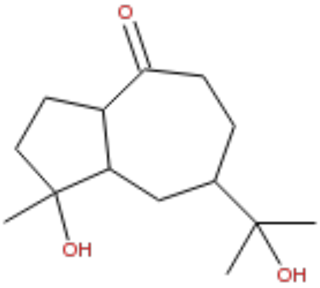

Cell ID	Cluster Center	Number of Compounds
69	0	25

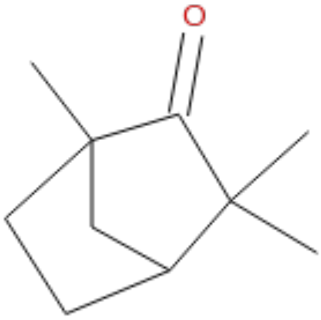

Cell ID	Cluster Center	Number of Compounds
69	0	25

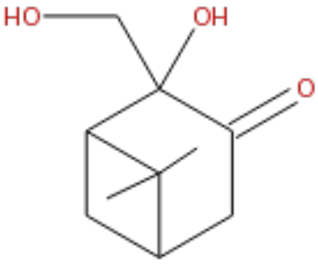

Cell ID	Cluster Center	Number of Compounds
69	0	25

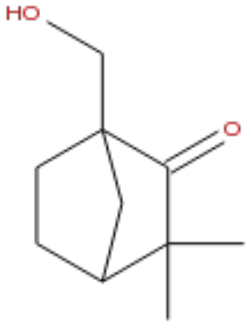

Cell ID	Cluster Center	Number of Compounds
69	0	25

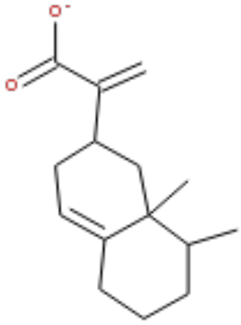

Cell ID	Cluster Center	Number of Compounds
70	1	7

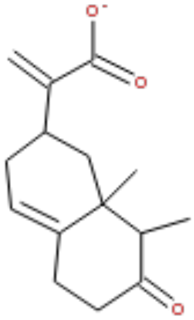

Cell ID	Cluster Center	Number of Compounds
70	0	7

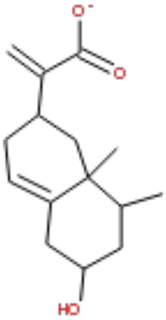

Cell ID	Cluster Center	Number of Compounds
70	0	7

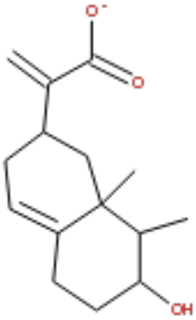

Cell ID	Cluster Center	Number of Compounds
70	0	7

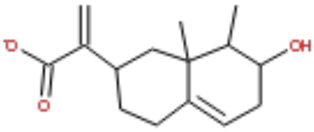

Cell ID	Cluster Center	Number of Compounds
70	0	7

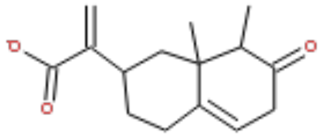

Cell ID	Cluster Center	Number of Compounds
70	0	7

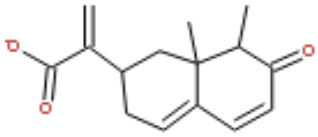

Cell ID	Cluster Center	Number of Compounds
70	0	7

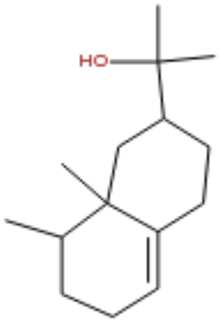

Cell ID	Cluster Center	Number of Compounds
71	1	12

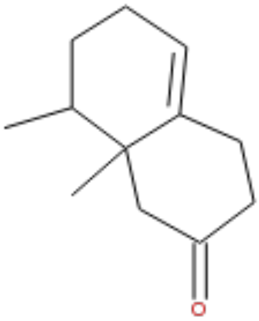

Cell ID	Cluster Center	Number of Compounds
71	0	12

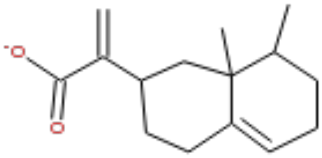

Cell ID	Cluster Center	Number of Compounds
71	0	12

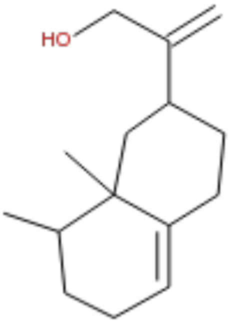

Cell ID	Cluster Center	Number of Compounds
71	0	12

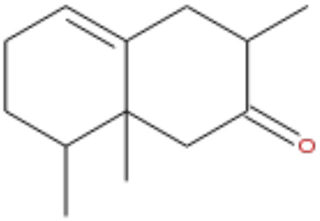

Cell ID	Cluster Center	Number of Compounds
71	0	12

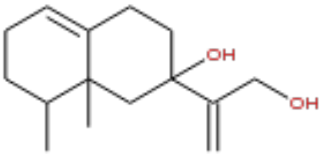

Cell ID	Cluster Center	Number of Compounds
71	0	12

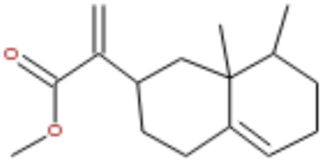

Cell ID	Cluster Center	Number of Compounds
71	0	12

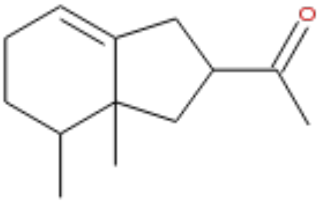

Cell ID	Cluster Center	Number of Compounds
71	0	12

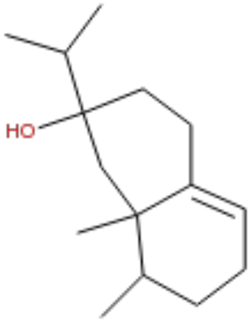

Cell ID	Cluster Center	Number of Compounds
71	0	12

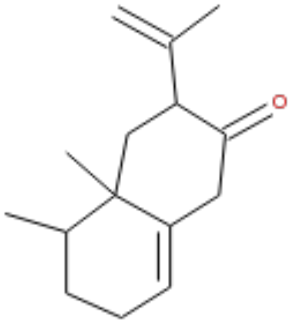

Cell ID	Cluster Center	Number of Compounds
71	0	12

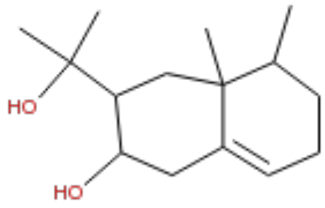

Cell ID	Cluster Center	Number of Compounds
71	0	12

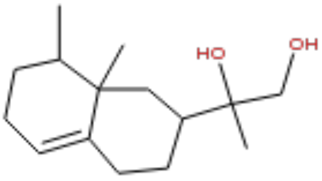

Cell ID	Cluster Center	Number of Compounds
71	0	12

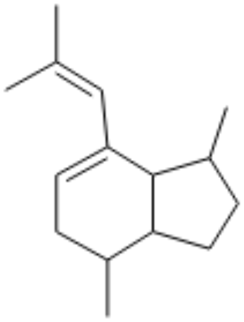

Cell ID	Cluster Center	Number of Compounds
73	1	4

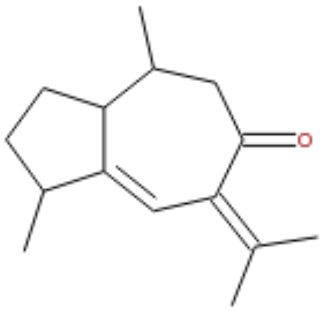

Cell ID	Cluster Center	Number of Compounds
73	0	4

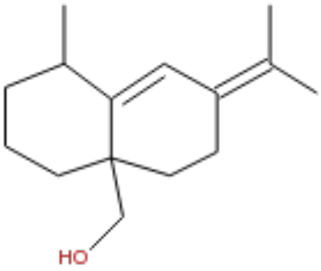

Cell ID	Cluster Center	Number of Compounds
73	0	4

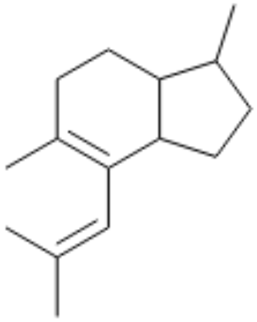

Cell ID	Cluster Center	Number of Compounds
73	0	4

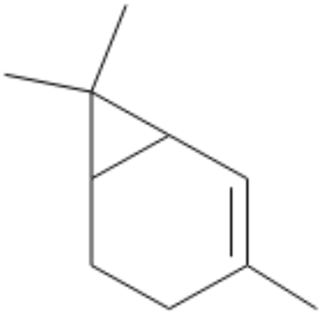

Cell ID	Cluster Center	Number of Compounds
75	1	5

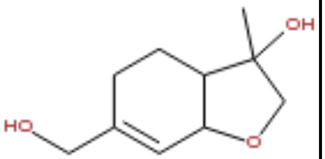

Cell ID	Cluster Center	Number of Compounds
75	0	5

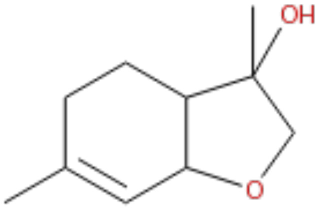

Cell ID	Cluster Center	Number of Compounds
75	0	5

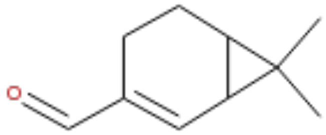

Cell ID	Cluster Center	Number of Compounds
75	0	5

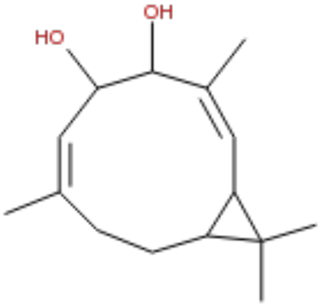

Cell ID	Cluster Center	Number of Compounds
75	0	5

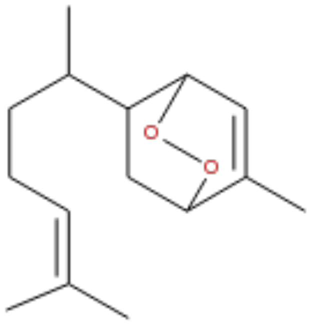

Cell ID	Cluster Center	Number of Compounds
77	1	1

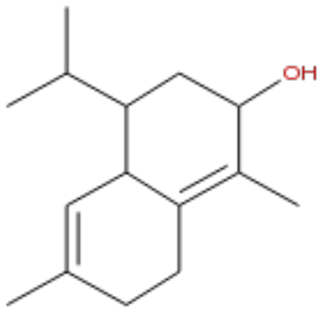

Cell ID	Cluster Center	Number of Compounds
78	1	4

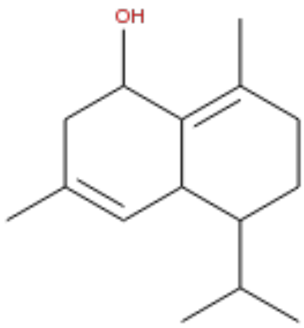

Cell ID	Cluster Center	Number of Compounds
78	0	4

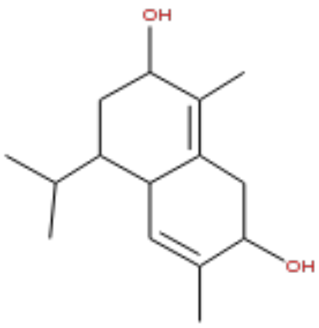

Cell ID	Cluster Center	Number of Compounds
78	0	4

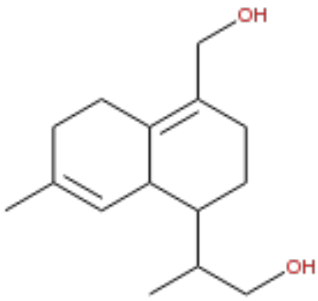

Cell ID	Cluster Center	Number of Compounds
78	0	4

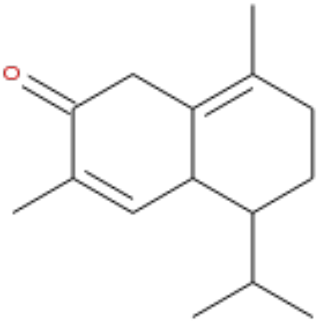

Cell ID	Cluster Center	Number of Compounds
79	1	6

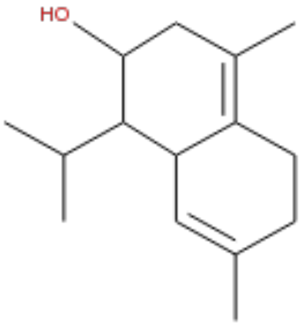

Cell ID	Cluster Center	Number of Compounds
79	0	6

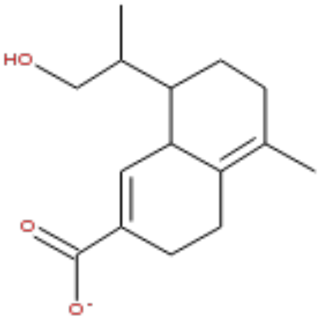

Cell ID	Cluster Center	Number of Compounds
79	0	6

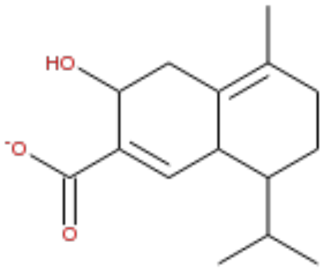

Cell ID	Cluster Center	Number of Compounds
79	0	6

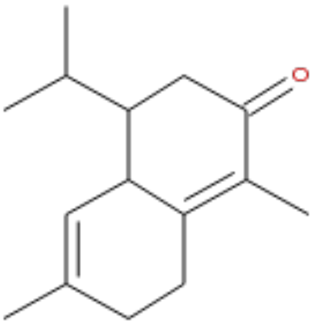

Cell ID	Cluster Center	Number of Compounds
79	0	6

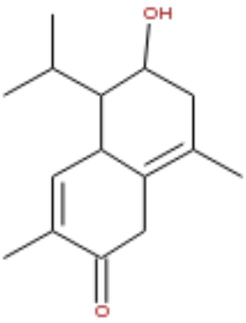

Cell ID	Cluster Center	Number of Compounds
79	0	6

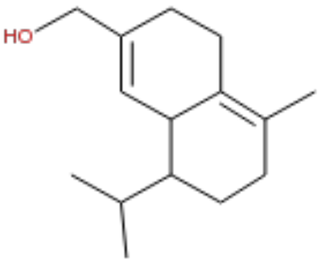

Cell ID	Cluster Center	Number of Compounds
80	1	4

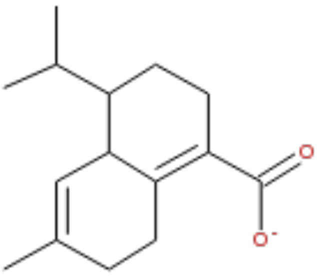

Cell ID	Cluster Center	Number of Compounds
80	0	4

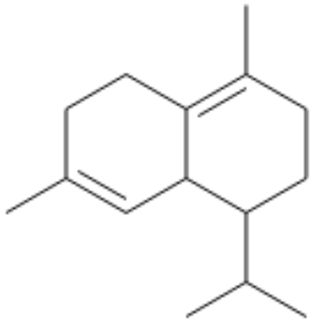

Cell ID	Cluster Center	Number of Compounds
80	0	4

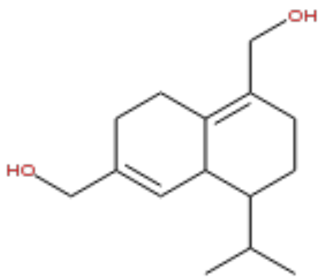

Cell ID	Cluster Center	Number of Compounds
80	0	4

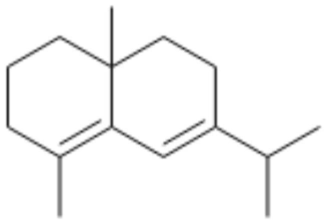

Cell ID	Cluster Center	Number of Compounds
81	1	7

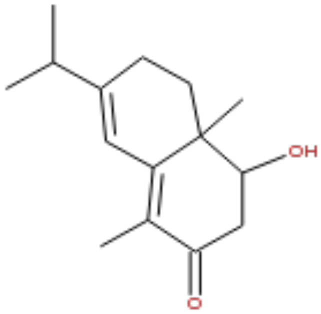

Cell ID	Cluster Center	Number of Compounds
81	0	7

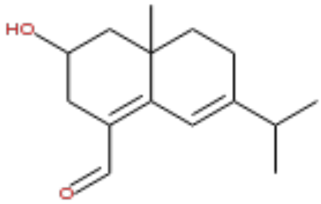

Cell ID	Cluster Center	Number of Compounds
81	0	7

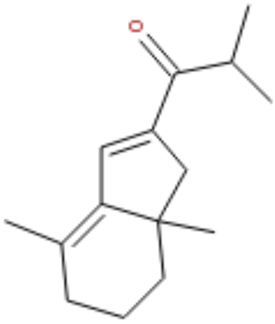

Cell ID	Cluster Center	Number of Compounds
81	0	7

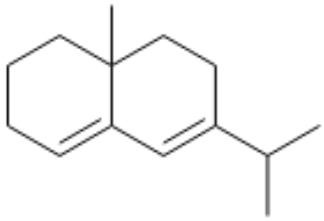

Cell ID	Cluster Center	Number of Compounds
81	0	7

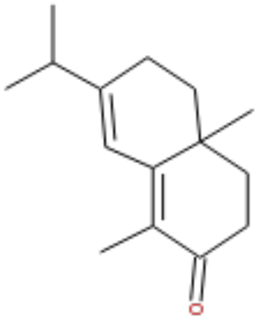

Cell ID	Cluster Center	Number of Compounds
81	0	7

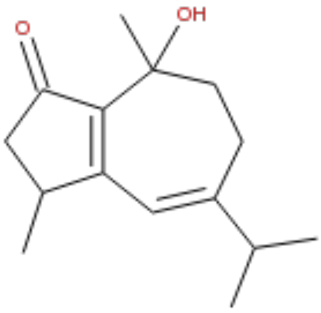

Cell ID	Cluster Center	Number of Compounds
81	0	7

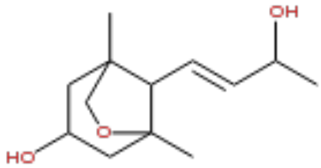

Cell ID	Cluster Center	Number of Compounds
82	1	4

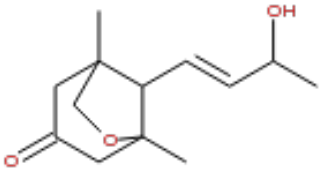

Cell ID	Cluster Center	Number of Compounds
82	0	4

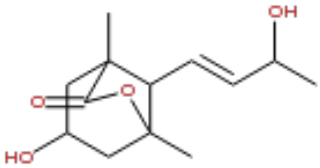

Cell ID	Cluster Center	Number of Compounds
82	0	4

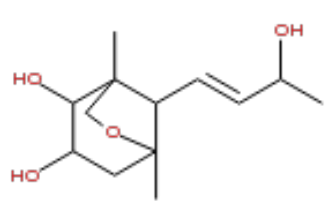

Cell ID	Cluster Center	Number of Compounds
82	0	4

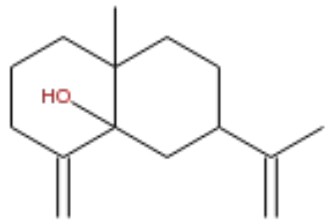

Cell ID	Cluster Center	Number of Compounds
83	1	5

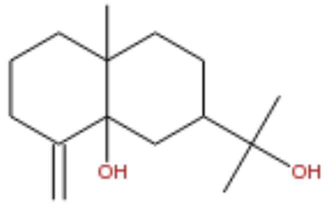

Cell ID	Cluster Center	Number of Compounds
83	0	5

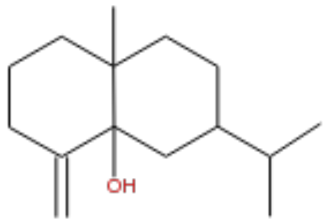

Cell ID	Cluster Center	Number of Compounds
83	0	5

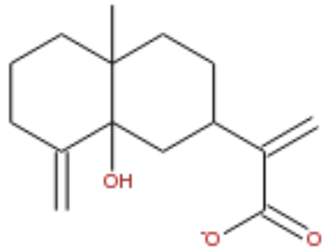

Cell ID	Cluster Center	Number of Compounds
83	0	5

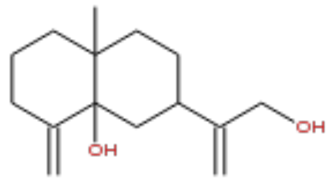

Cell ID	Cluster Center	Number of Compounds
83	0	5

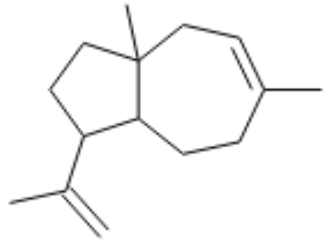

Cell ID	Cluster Center	Number of Compounds
86	1	8

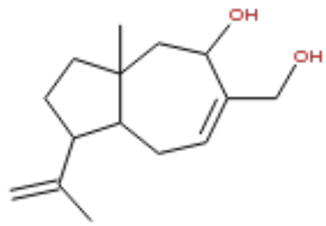

Cell ID	Cluster Center	Number of Compounds
86	0	8

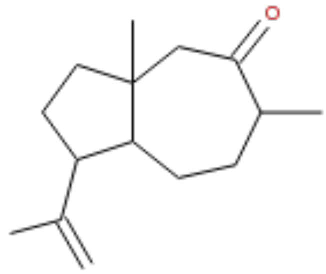

Cell ID	Cluster Center	Number of Compounds
86	0	8

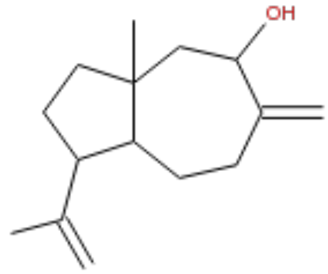

Cell ID	Cluster Center	Number of Compounds
86	0	8

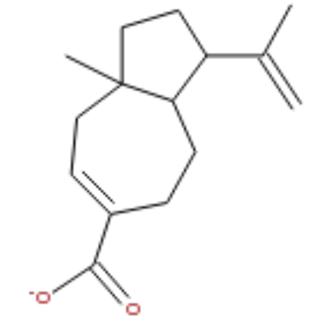

Cell ID	Cluster Center	Number of Compounds
86	0	8

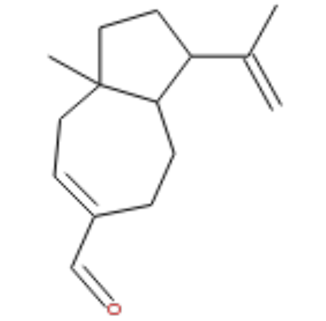

Cell ID	Cluster Center	Number of Compounds
86	0	8

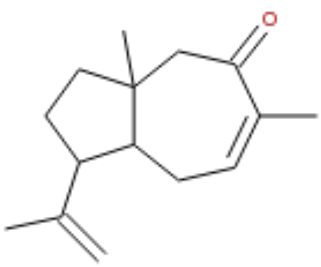

Cell ID	Cluster Center	Number of Compounds
86	0	8

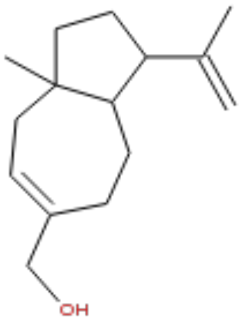

Cell ID	Cluster Center	Number of Compounds
86	0	8

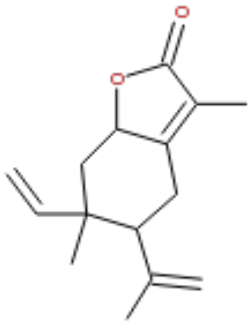

Cell ID	Cluster Center	Number of Compounds
87	1	3

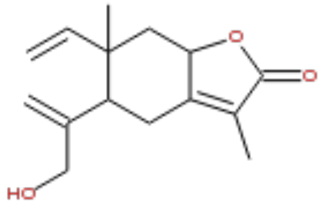

Cell ID	Cluster Center	Number of Compounds
87	0	3

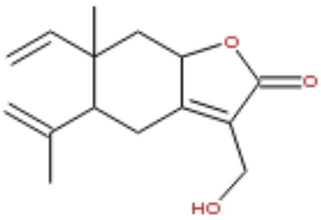

Cell ID	Cluster Center	Number of Compounds
87	0	3

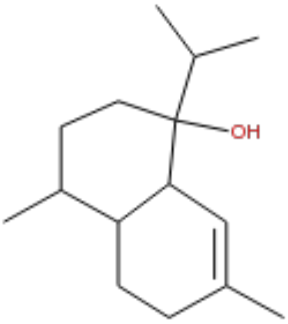

Cell ID	Cluster Center	Number of Compounds
88	1	7

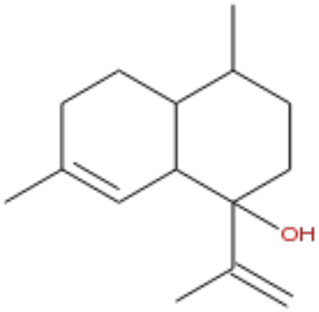

Cell ID	Cluster Center	Number of Compounds
88	0	7

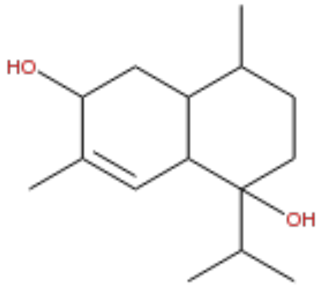

Cell ID	Cluster Center	Number of Compounds
88	0	7

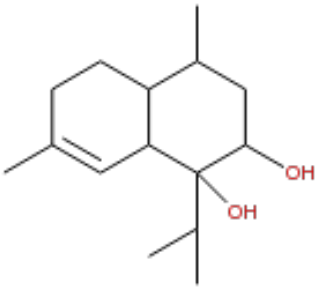

Cell ID	Cluster Center	Number of Compounds
88	0	7

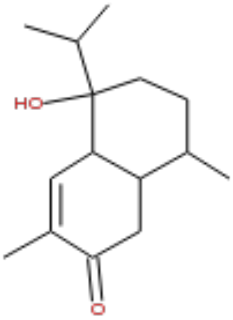

Cell ID	Cluster Center	Number of Compounds
88	0	7

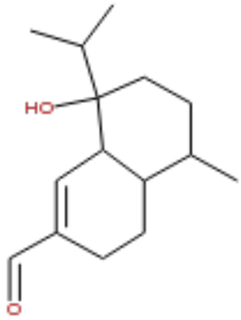

Cell ID	Cluster Center	Number of Compounds
88	0	7

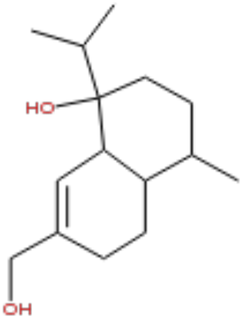

Cell ID	Cluster Center	Number of Compounds
88	0	7

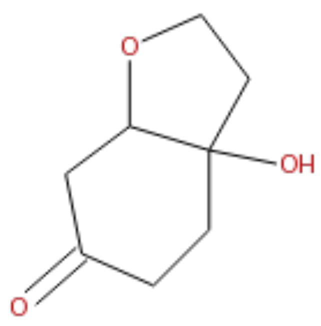

Cell ID	Cluster Center	Number of Compounds
89	1	19

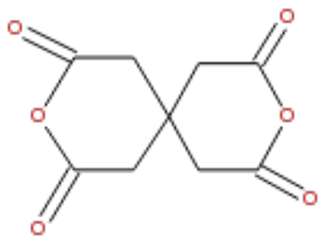

Cell ID	Cluster Center	Number of Compounds
89	0	19

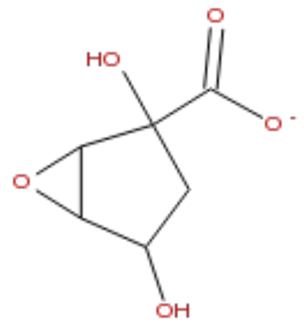

Cell ID	Cluster Center	Number of Compounds
89	0	19

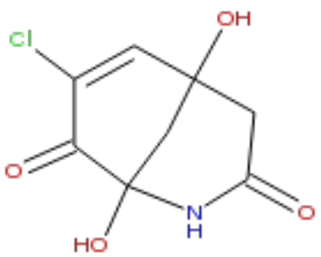

Cell ID	Cluster Center	Number of Compounds
89	0	19

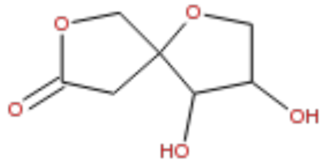

Cell ID	Cluster Center	Number of Compounds
89	0	19

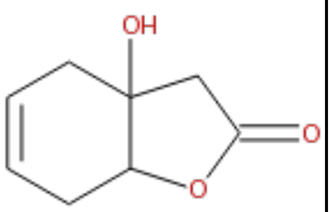

Cell ID	Cluster Center	Number of Compounds
89	0	19

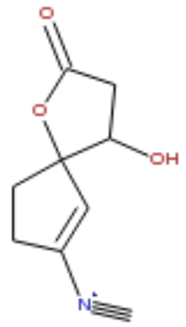

Cell ID	Cluster Center	Number of Compounds
89	0	19

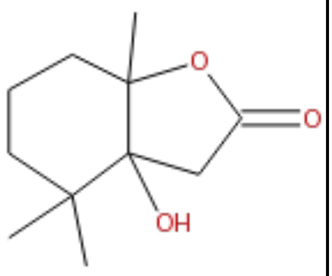

Cell ID	Cluster Center	Number of Compounds
89	0	19

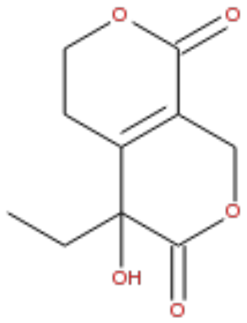

Cell ID	Cluster Center	Number of Compounds
89	0	19

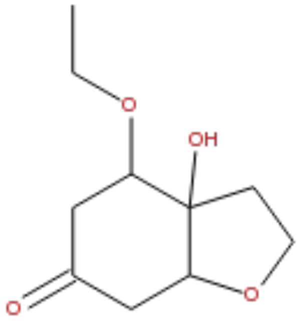

Cell ID	Cluster Center	Number of Compounds
89	0	19

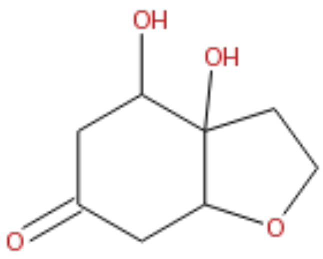

Cell ID	Cluster Center	Number of Compounds
89	0	19

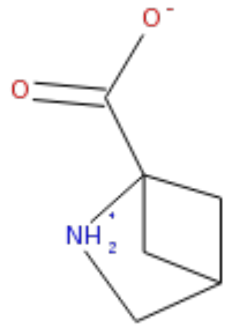

Cell ID	Cluster Center	Number of Compounds
89	0	19

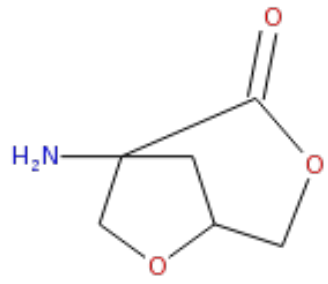

Cell ID	Cluster Center	Number of Compounds
89	0	19

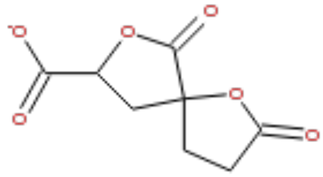

Cell ID	Cluster Center	Number of Compounds
89	0	19

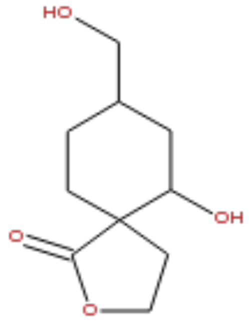

Cell ID	Cluster Center	Number of Compounds
89	0	19

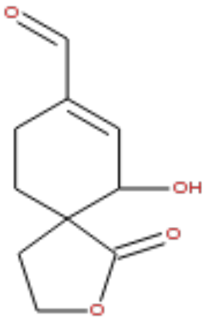

Cell ID	Cluster Center	Number of Compounds
89	0	19

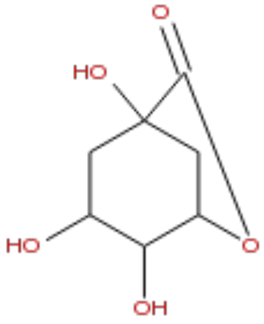

Cell ID	Cluster Center	Number of Compounds
89	0	19

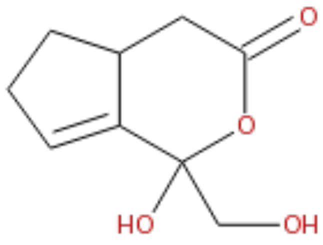

Cell ID	Cluster Center	Number of Compounds
89	0	19

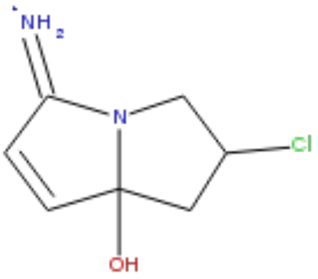

Cell ID	Cluster Center	Number of Compounds
89	0	19

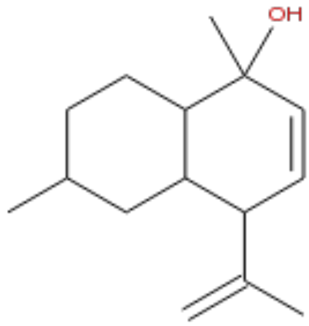

Cell ID	Cluster Center	Number of Compounds
90	1	1

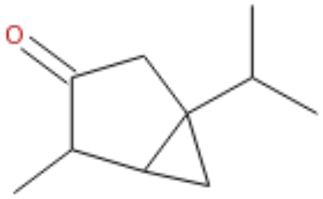

Cell ID	Cluster Center	Number of Compounds
91	1	14

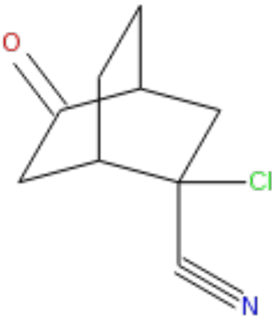

Cell ID	Cluster Center	Number of Compounds
91	0	14

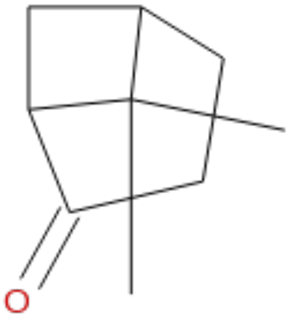

Cell ID	Cluster Center	Number of Compounds
91	0	14

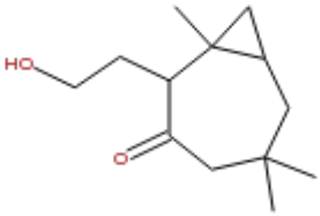

Cell ID	Cluster Center	Number of Compounds
91	0	14

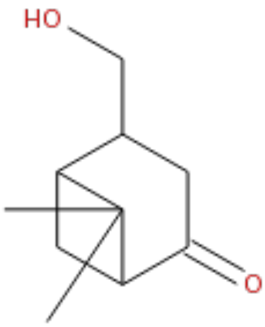

Cell ID	Cluster Center	Number of Compounds
91	0	14

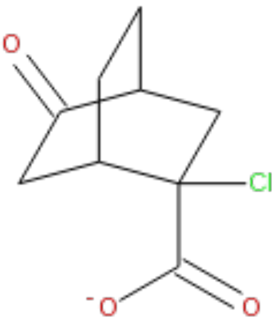

Cell ID	Cluster Center	Number of Compounds
91	0	14

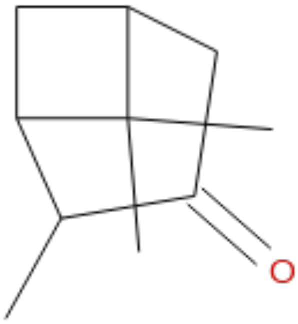

Cell ID	Cluster Center	Number of Compounds
91	0	14

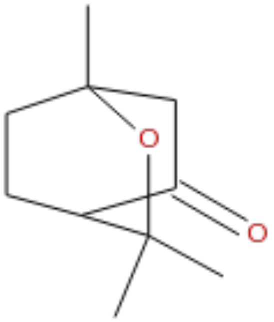

Cell ID	Cluster Center	Number of Compounds
91	0	14

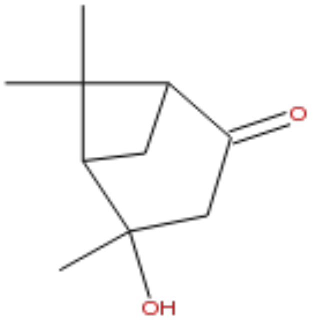

Cell ID	Cluster Center	Number of Compounds
91	0	14

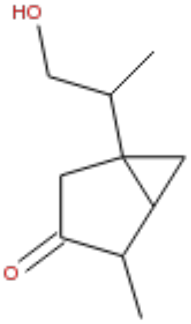

Cell ID	Cluster Center	Number of Compounds
91	0	14

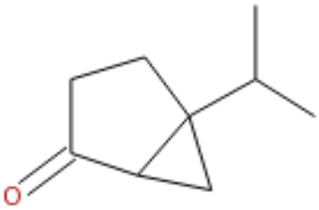

Cell ID	Cluster Center	Number of Compounds
91	0	14

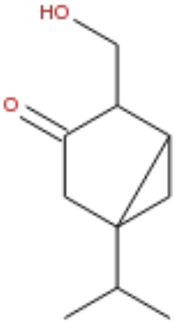

Cell ID	Cluster Center	Number of Compounds
91	0	14

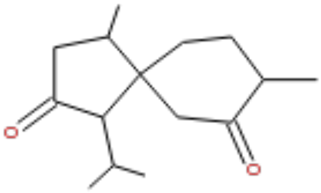

Cell ID	Cluster Center	Number of Compounds
91	0	14

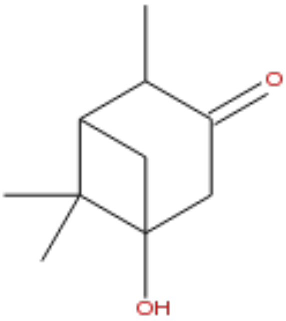

Cell ID	Cluster Center	Number of Compounds
91	0	14

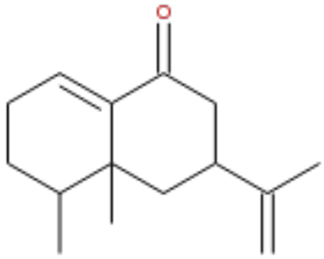

Cell ID	Cluster Center	Number of Compounds
96	1	7

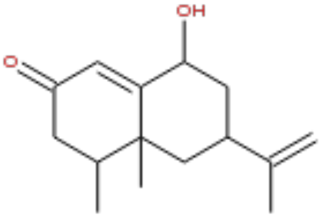

Cell ID	Cluster Center	Number of Compounds
96	0	7

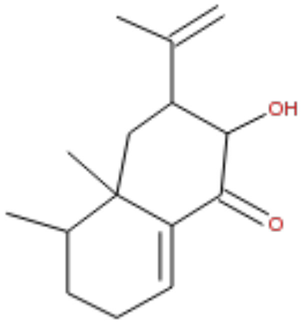

Cell ID	Cluster Center	Number of Compounds
96	0	7

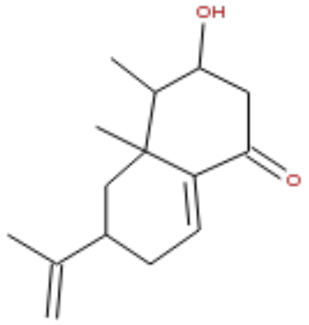

Cell ID	Cluster Center	Number of Compounds
96	0	7

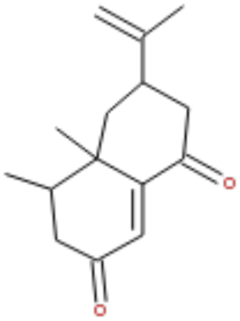

Cell ID	Cluster Center	Number of Compounds
96	0	7

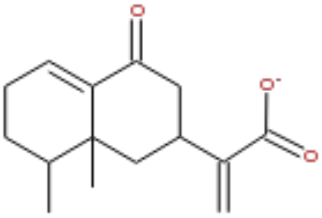

Cell ID	Cluster Center	Number of Compounds
96	0	7

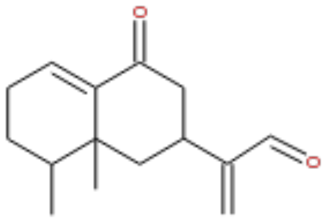

Cell ID	Cluster Center	Number of Compounds
96	0	7

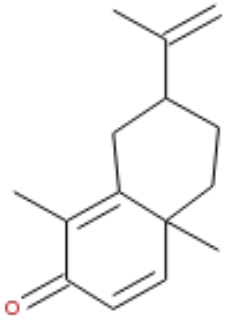

Cell ID	Cluster Center	Number of Compounds
98	1	6

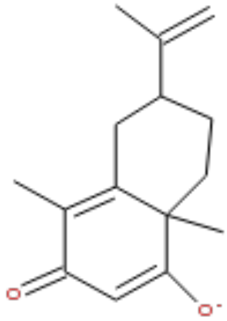

Cell ID	Cluster Center	Number of Compounds
98	0	6

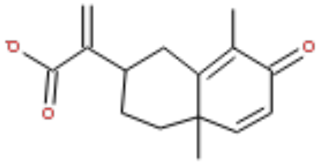

Cell ID	Cluster Center	Number of Compounds
98	0	6

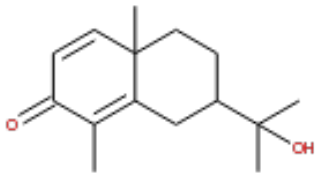

Cell ID	Cluster Center	Number of Compounds
98	0	6

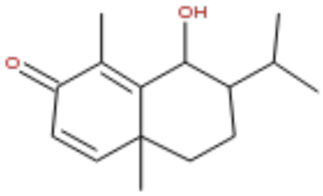

Cell ID	Cluster Center	Number of Compounds
98	0	6

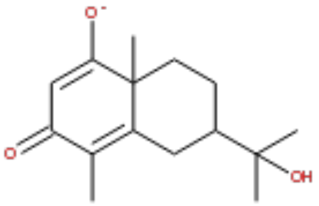

Cell ID	Cluster Center	Number of Compounds
98	0	6

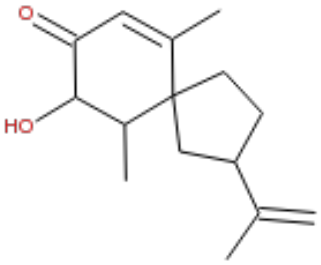

Cell ID	Cluster Center	Number of Compounds
100	1	7

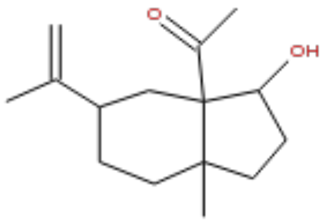

Cell ID	Cluster Center	Number of Compounds
100	0	7

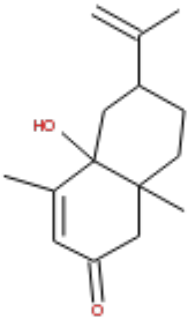

Cell ID	Cluster Center	Number of Compounds
100	0	7

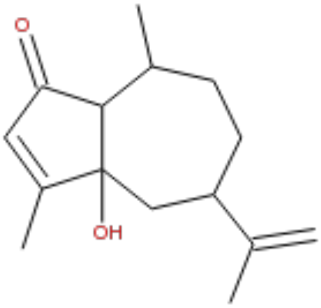

Cell ID	Cluster Center	Number of Compounds
100	0	7

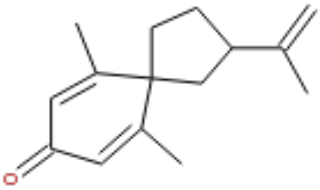

Cell ID	Cluster Center	Number of Compounds
100	0	7

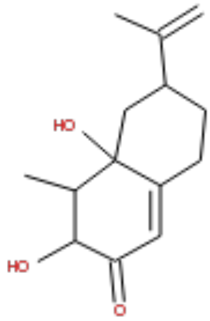

Cell ID	Cluster Center	Number of Compounds
100	0	7

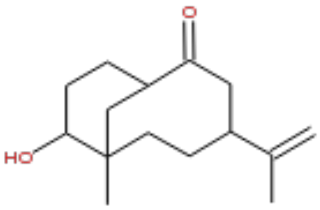

Cell ID	Cluster Center	Number of Compounds
100	0	7

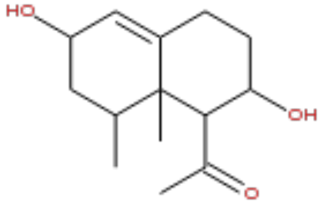

Cell ID	Cluster Center	Number of Compounds
101	1	3

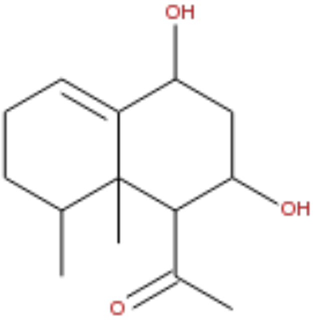

Cell ID	Cluster Center	Number of Compounds
101	0	3

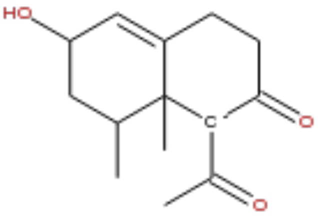

Cell ID	Cluster Center	Number of Compounds
101	0	3

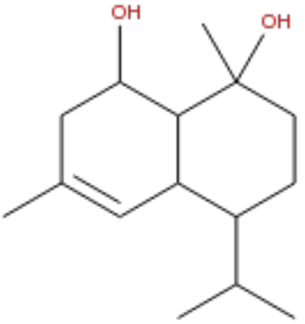

Cell ID	Cluster Center	Number of Compounds
102	1	1

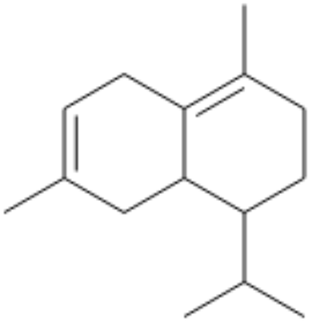

Cell ID	Cluster Center	Number of Compounds
104	1	4

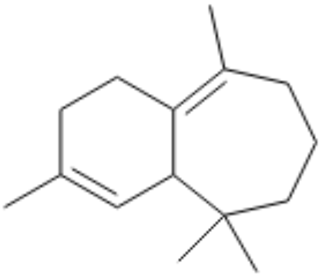

Cell ID	Cluster Center	Number of Compounds
104	0	4

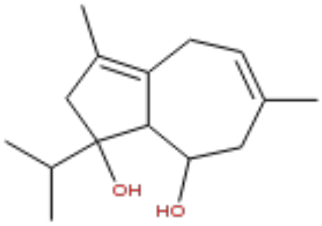

Cell ID	Cluster Center	Number of Compounds
104	0	4

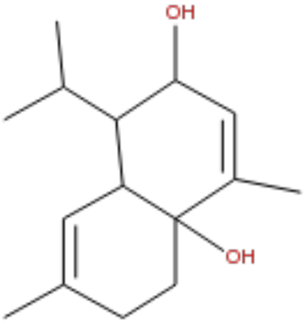

Cell ID	Cluster Center	Number of Compounds
104	0	4

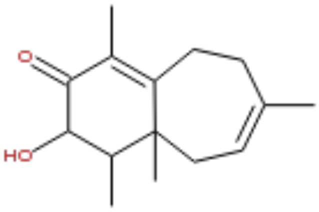

Cell ID	Cluster Center	Number of Compounds
105	1	5

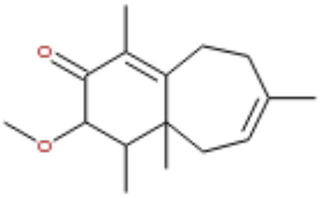

Cell ID	Cluster Center	Number of Compounds
105	0	5

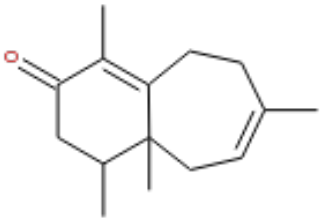

Cell ID	Cluster Center	Number of Compounds
105	0	5

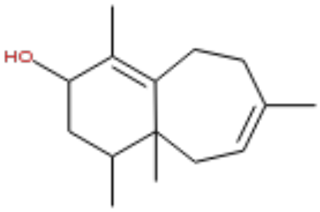

Cell ID	Cluster Center	Number of Compounds
105	0	5

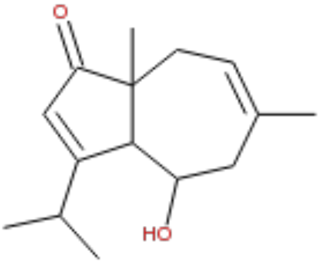

Cell ID	Cluster Center	Number of Compounds
105	0	5

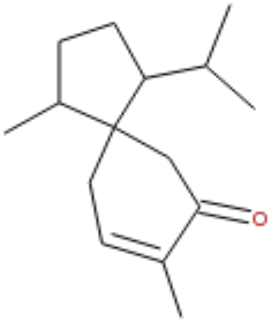

Cell ID	Cluster Center	Number of Compounds
106	1	8

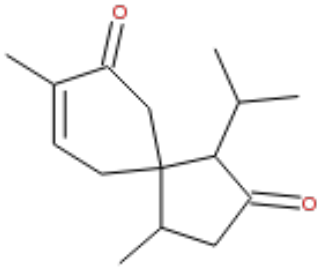

Cell ID	Cluster Center	Number of Compounds
106	0	8

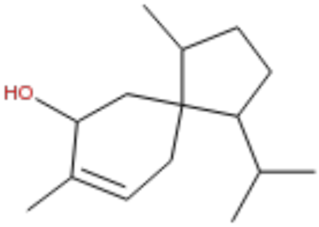

Cell ID	Cluster Center	Number of Compounds
106	0	8

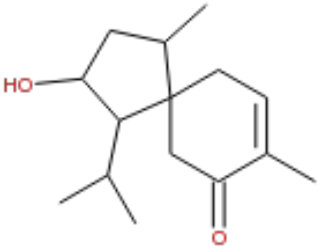

Cell ID	Cluster Center	Number of Compounds
106	0	8

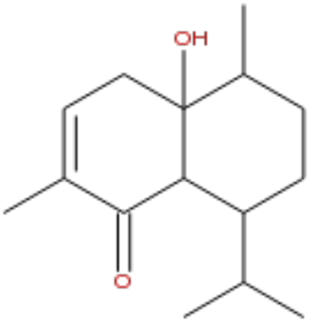

Cell ID	Cluster Center	Number of Compounds
106	0	8

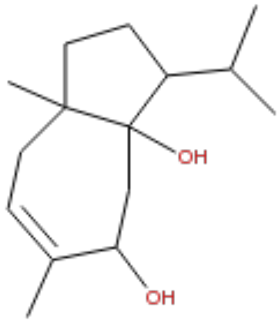

Cell ID	Cluster Center	Number of Compounds
106	0	8

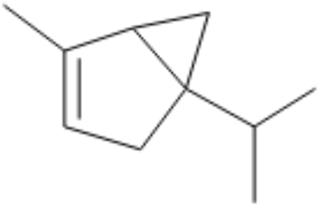

Cell ID	Cluster Center	Number of Compounds
106	0	8

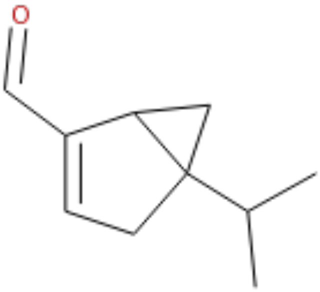

Cell ID	Cluster Center	Number of Compounds
106	0	8

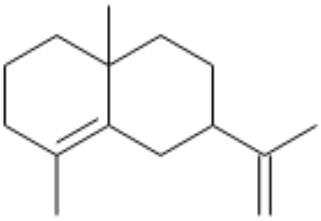

Cell ID	Cluster Center	Number of Compounds
108	1	12

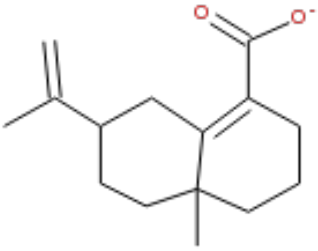

Cell ID	Cluster Center	Number of Compounds
108	0	12

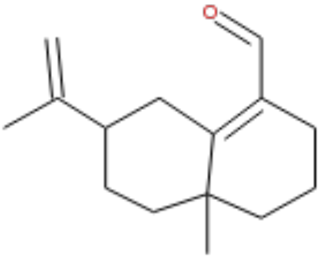

Cell ID	Cluster Center	Number of Compounds
108	0	12

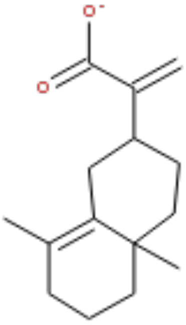

Cell ID	Cluster Center	Number of Compounds
108	0	12

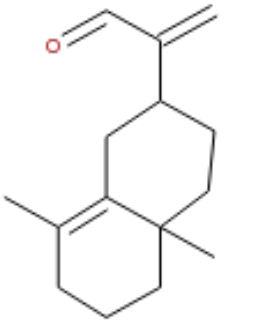

Cell ID	Cluster Center	Number of Compounds
108	0	12

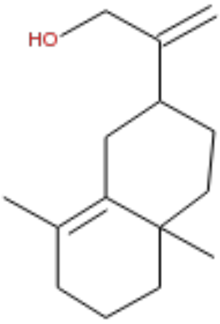

Cell ID	Cluster Center	Number of Compounds
108	0	12

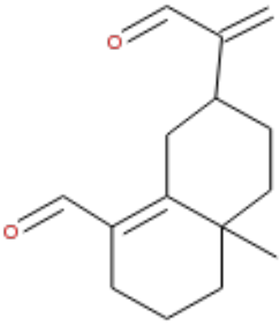

Cell ID	Cluster Center	Number of Compounds
108	0	12

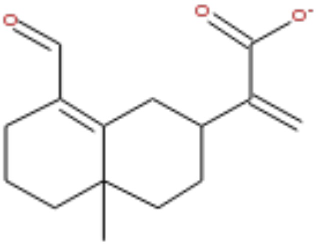

Cell ID	Cluster Center	Number of Compounds
108	0	12

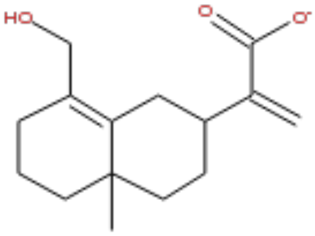

Cell ID	Cluster Center	Number of Compounds
108	0	12

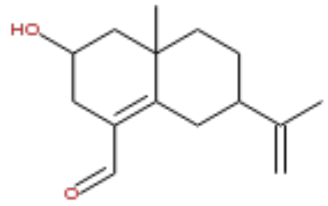

Cell ID	Cluster Center	Number of Compounds
108	0	12

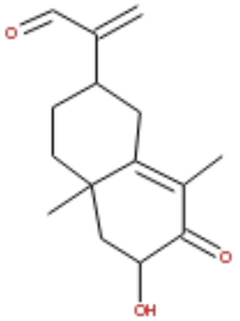

Cell ID	Cluster Center	Number of Compounds
108	0	12

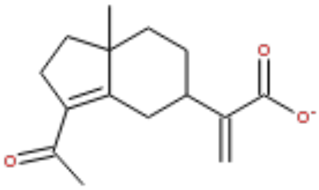

Cell ID	Cluster Center	Number of Compounds
108	0	12

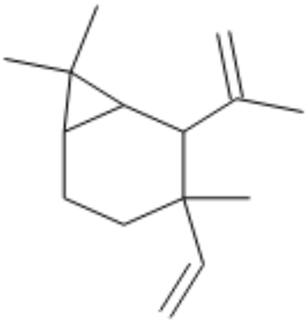

Cell ID	Cluster Center	Number of Compounds
109	1	5

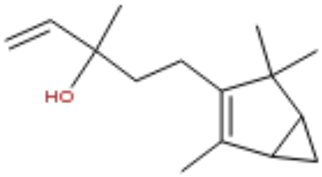

Cell ID	Cluster Center	Number of Compounds
109	0	5

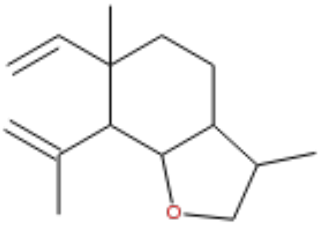

Cell ID	Cluster Center	Number of Compounds
109	0	5

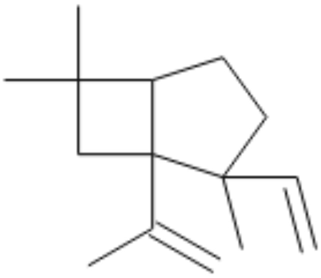

Cell ID	Cluster Center	Number of Compounds
109	0	5

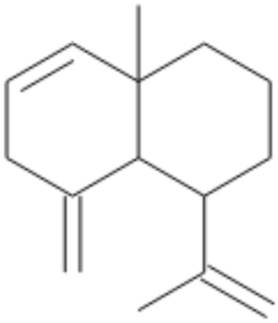

Cell ID	Cluster Center	Number of Compounds
109	0	5

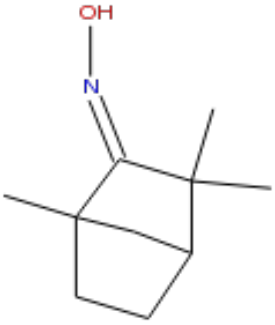

Cell ID	Cluster Center	Number of Compounds
111	1	9

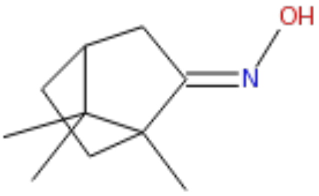

Cell ID	Cluster Center	Number of Compounds
111	0	9

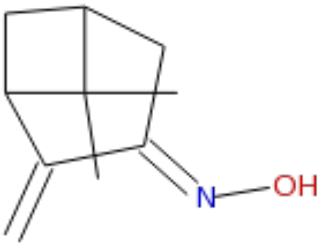

Cell ID	Cluster Center	Number of Compounds
111	0	9

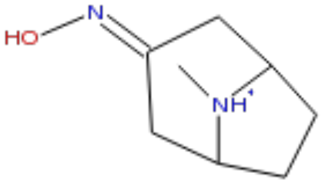

Cell ID	Cluster Center	Number of Compounds
111	0	9

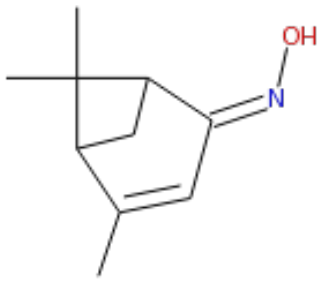

Cell ID	Cluster Center	Number of Compounds
111	0	9

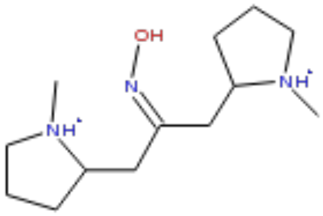

Cell ID	Cluster Center	Number of Compounds
111	0	9

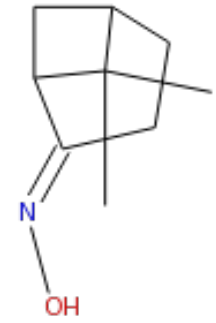

Cell ID	Cluster Center	Number of Compounds
111	0	9

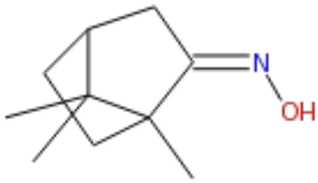

Cell ID	Cluster Center	Number of Compounds
111	0	9

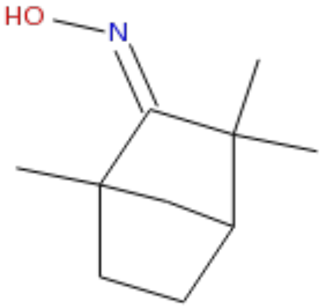

Cell ID	Cluster Center	Number of Compounds
111	0	9

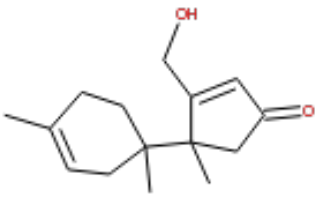

Cell ID	Cluster Center	Number of Compounds
112	1	5

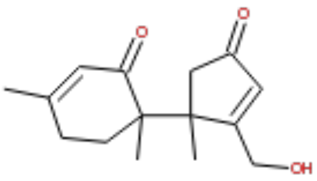

Cell ID	Cluster Center	Number of Compounds
112	0	5

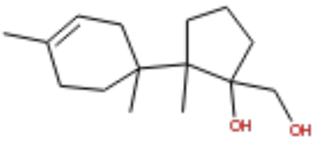

Cell ID	Cluster Center	Number of Compounds
112	0	5

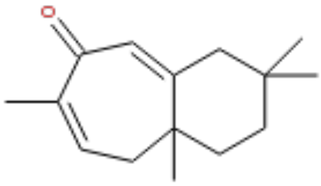

Cell ID	Cluster Center	Number of Compounds
112	0	5

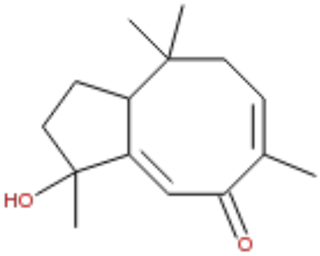

Cell ID	Cluster Center	Number of Compounds
112	0	5

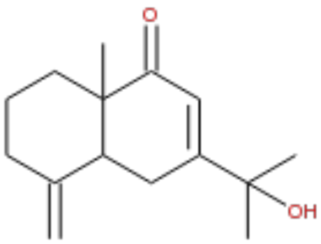

Cell ID	Cluster Center	Number of Compounds
113	1	5

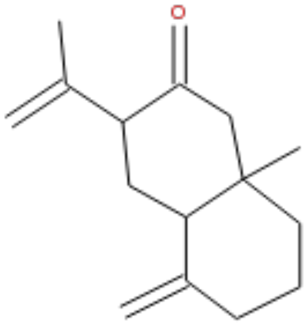

Cell ID	Cluster Center	Number of Compounds
113	0	5

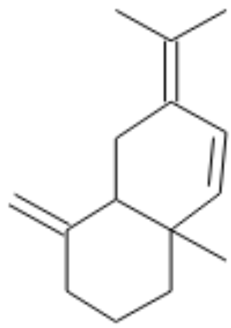

Cell ID	Cluster Center	Number of Compounds
113	0	5

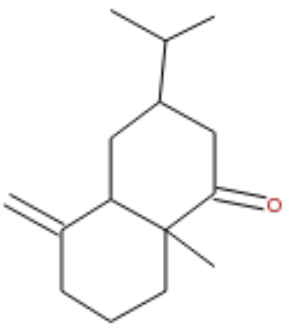

Cell ID	Cluster Center	Number of Compounds
113	0	5

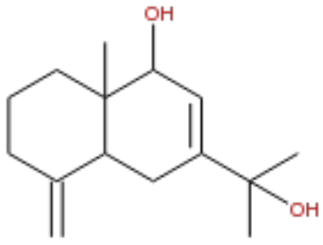

Cell ID	Cluster Center	Number of Compounds
113	0	5

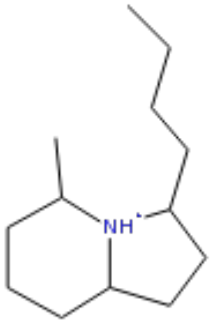

Cell ID	Cluster Center	Number of Compounds
114	1	26

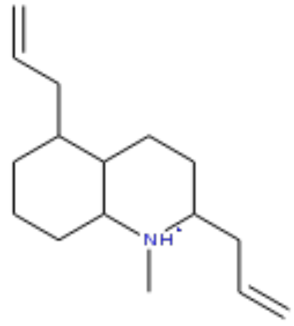

Cell ID	Cluster Center	Number of Compounds
114	0	26

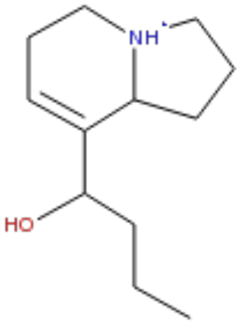

Cell ID	Cluster Center	Number of Compounds
114	0	26

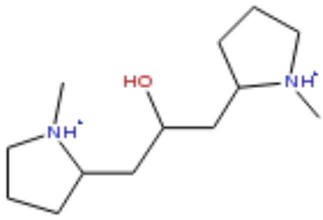

Cell ID	Cluster Center	Number of Compounds
114	0	26

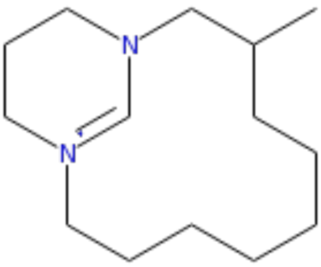

Cell ID	Cluster Center	Number of Compounds
114	0	26

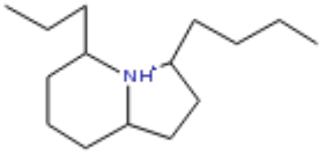

Cell ID	Cluster Center	Number of Compounds
114	0	26

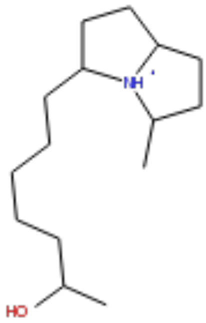

Cell ID	Cluster Center	Number of Compounds
114	0	26

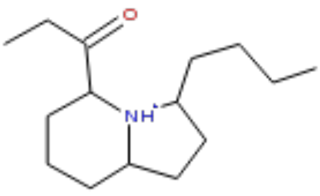

Cell ID	Cluster Center	Number of Compounds
114	0	26

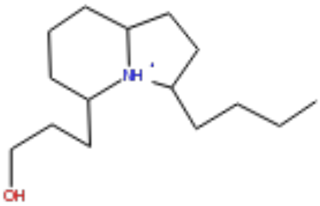

Cell ID	Cluster Center	Number of Compounds
114	0	26

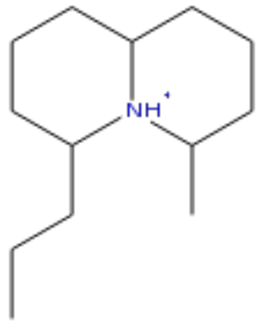

Cell ID	Cluster Center	Number of Compounds
114	0	26

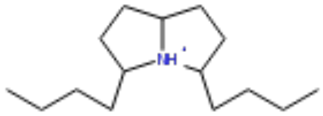

Cell ID	Cluster Center	Number of Compounds
114	0	26

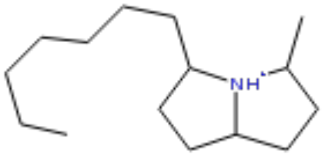

Cell ID	Cluster Center	Number of Compounds
114	0	26

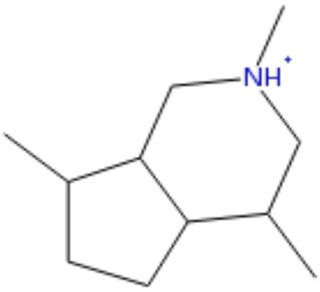

Cell ID	Cluster Center	Number of Compounds
114	0	26

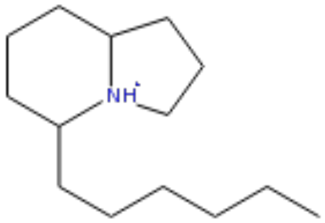

Cell ID	Cluster Center	Number of Compounds
114	0	26

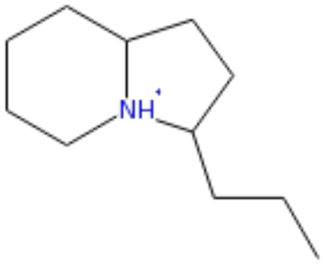

Cell ID	Cluster Center	Number of Compounds
114	0	26

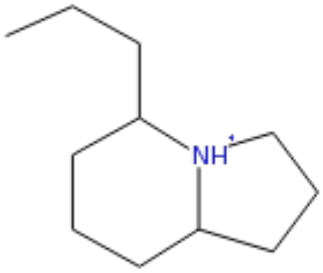

Cell ID	Cluster Center	Number of Compounds
114	0	26

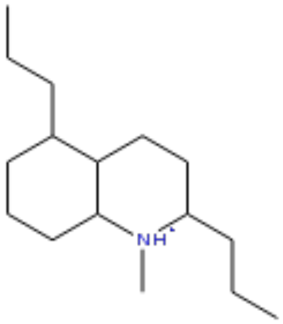

Cell ID	Cluster Center	Number of Compounds
114	0	26

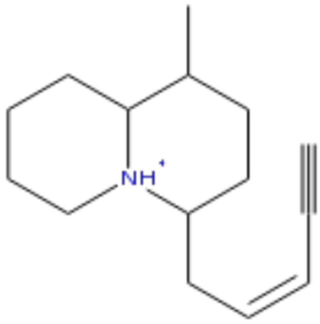

Cell ID	Cluster Center	Number of Compounds
114	0	26

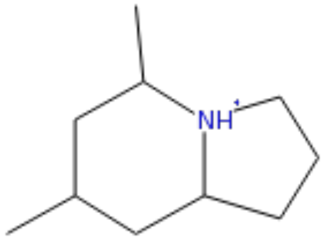

Cell ID	Cluster Center	Number of Compounds
114	0	26

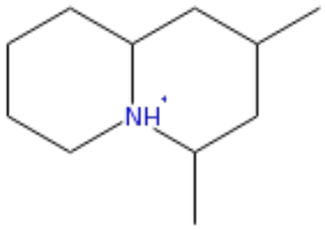

Cell ID	Cluster Center	Number of Compounds
114	0	26

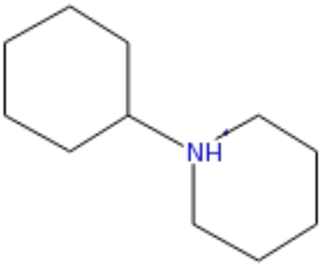

Cell ID	Cluster Center	Number of Compounds
114	0	26

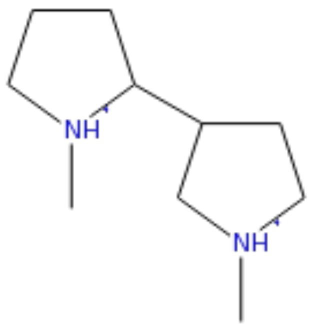

Cell ID	Cluster Center	Number of Compounds
114	0	26

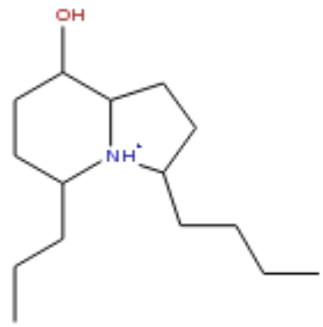

Cell ID	Cluster Center	Number of Compounds
114	0	26

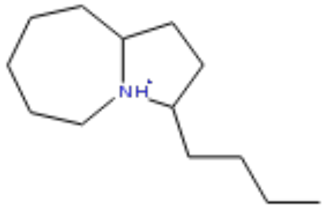

Cell ID	Cluster Center	Number of Compounds
114	0	26

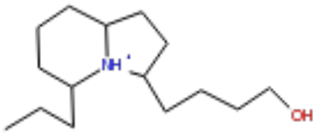

Cell ID	Cluster Center	Number of Compounds
114	0	26

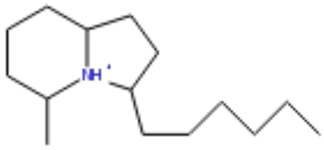

Cell ID	Cluster Center	Number of Compounds
114	0	26

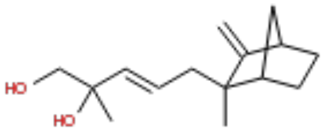

Cell ID	Cluster Center	Number of Compounds
115	1	3

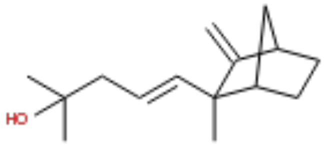

Cell ID	Cluster Center	Number of Compounds
115	0	3

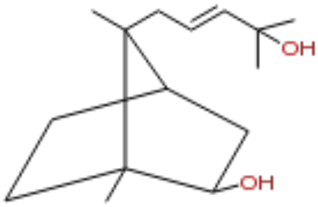

Cell ID	Cluster Center	Number of Compounds
115	0	3

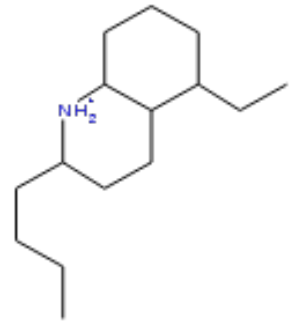

Cell ID	Cluster Center	Number of Compounds
117	1	3

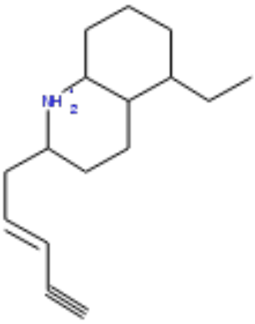

Cell ID	Cluster Center	Number of Compounds
117	0	3

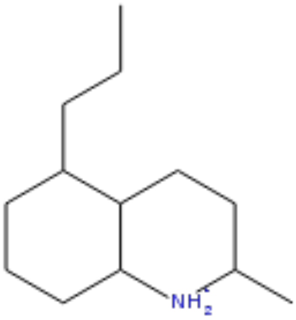

Cell ID	Cluster Center	Number of Compounds
117	0	3

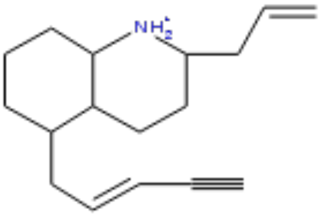

Cell ID	Cluster Center	Number of Compounds
118	1	3

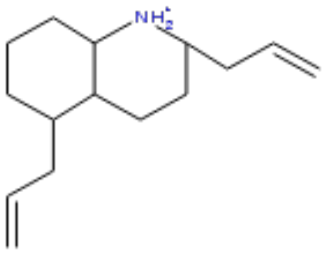

Cell ID	Cluster Center	Number of Compounds
118	0	3

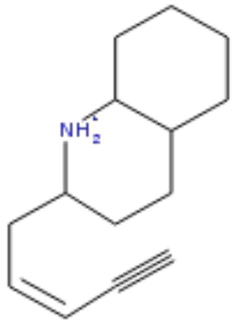

Cell ID	Cluster Center	Number of Compounds
118	0	3

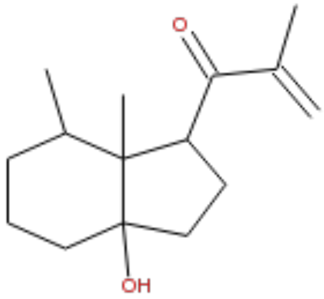

Cell ID	Cluster Center	Number of Compounds
119	1	4

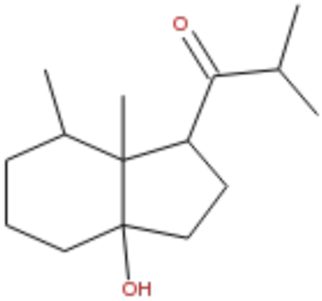

Cell ID	Cluster Center	Number of Compounds
119	0	4

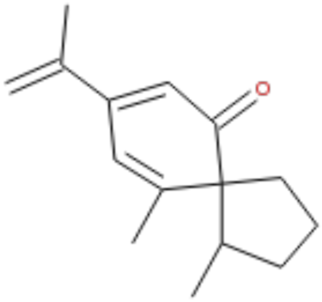

Cell ID	Cluster Center	Number of Compounds
119	0	4

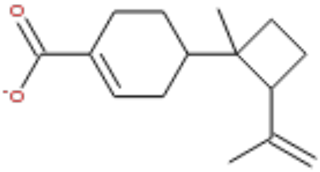

Cell ID	Cluster Center	Number of Compounds
119	0	4

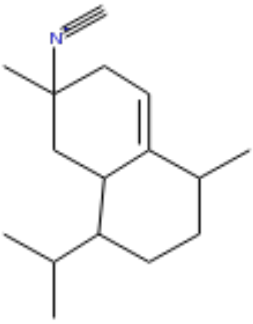

Cell ID	Cluster Center	Number of Compounds
120	1	4

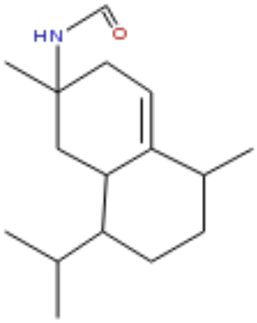

Cell ID	Cluster Center	Number of Compounds
120	0	4

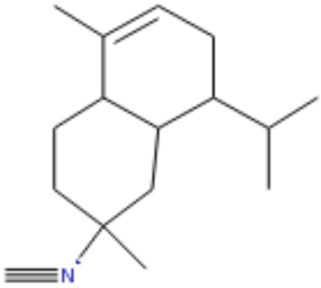

Cell ID	Cluster Center	Number of Compounds
120	0	4

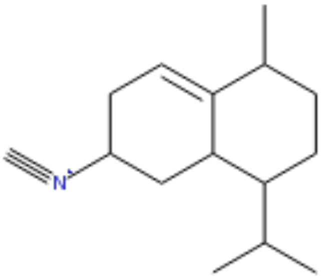

Cell ID	Cluster Center	Number of Compounds
120	0	4

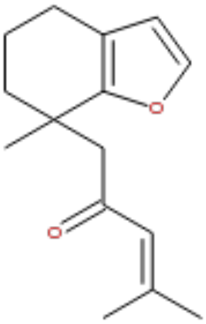

Cell ID	Cluster Center	Number of Compounds
121	1	3

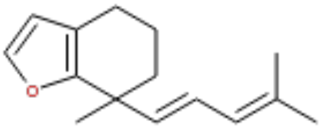

Cell ID	Cluster Center	Number of Compounds
121	0	3

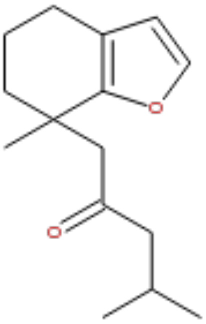

Cell ID	Cluster Center	Number of Compounds
121	0	3

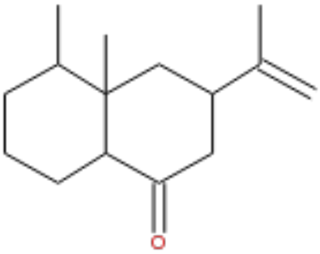

Cell ID	Cluster Center	Number of Compounds
123	1	12

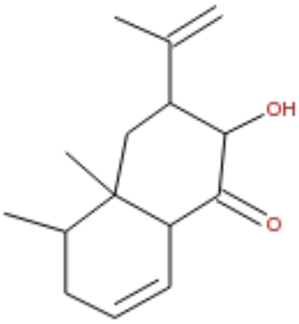

Cell ID	Cluster Center	Number of Compounds
123	0	12

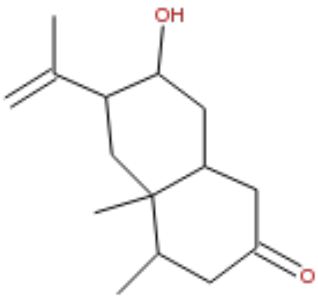

Cell ID	Cluster Center	Number of Compounds
123	0	12

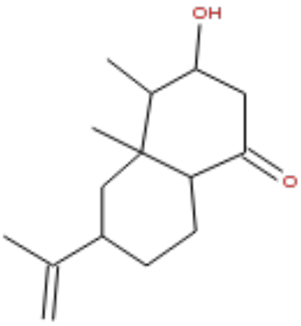

Cell ID	Cluster Center	Number of Compounds
123	0	12

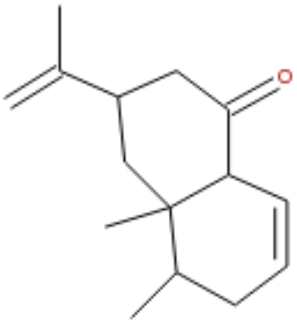

Cell ID	Cluster Center	Number of Compounds
123	0	12

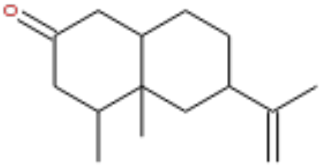

Cell ID	Cluster Center	Number of Compounds
123	0	12

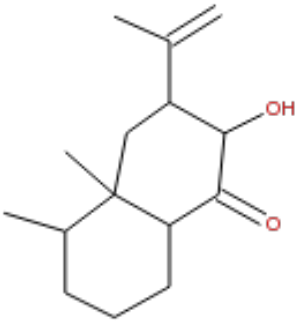

Cell ID	Cluster Center	Number of Compounds
123	0	12

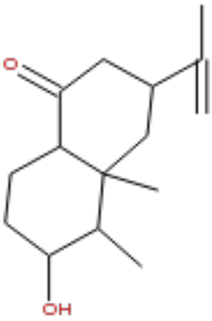

Cell ID	Cluster Center	Number of Compounds
123	0	12

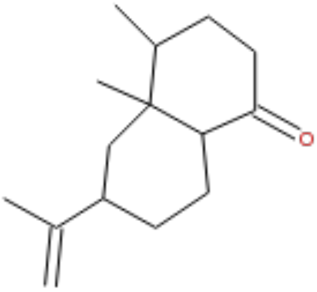

Cell ID	Cluster Center	Number of Compounds
123	0	12

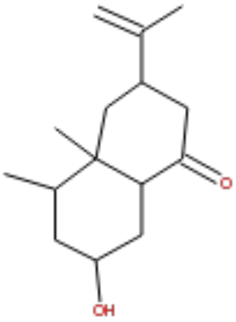

Cell ID	Cluster Center	Number of Compounds
123	0	12

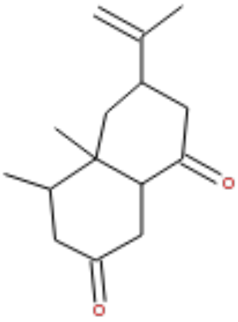

Cell ID	Cluster Center	Number of Compounds
123	0	12

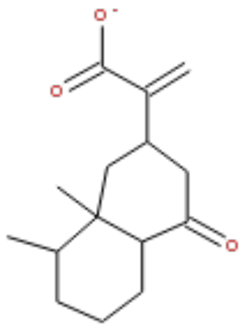

Cell ID	Cluster Center	Number of Compounds
123	0	12

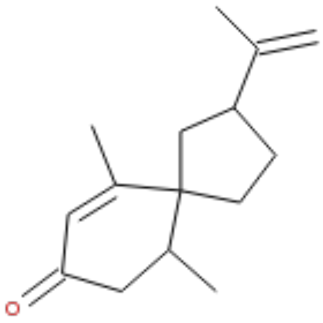

Cell ID	Cluster Center	Number of Compounds
124	1	10

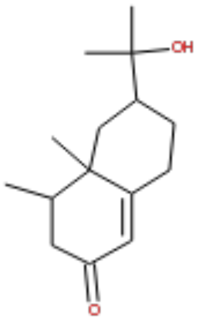

Cell ID	Cluster Center	Number of Compounds
124	0	10

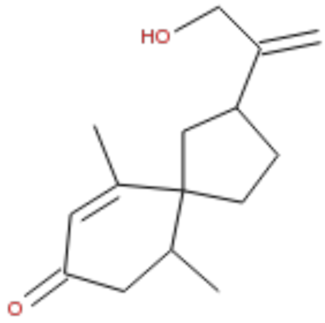

Cell ID	Cluster Center	Number of Compounds
124	0	10

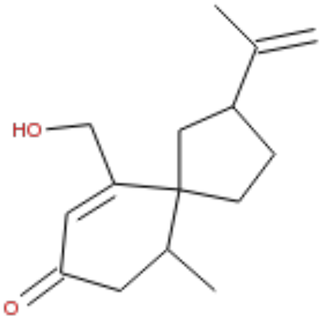

Cell ID	Cluster Center	Number of Compounds
124	0	10

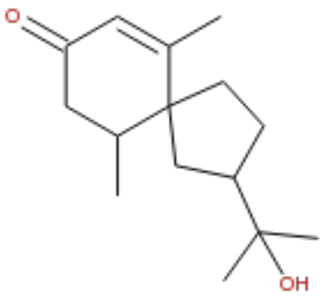

Cell ID	Cluster Center	Number of Compounds
124	0	10

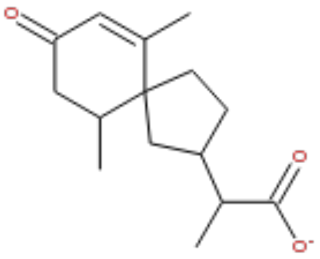

Cell ID	Cluster Center	Number of Compounds
124	0	10

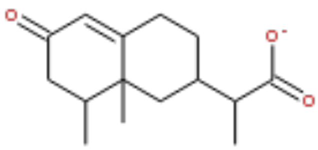

Cell ID	Cluster Center	Number of Compounds
124	0	10

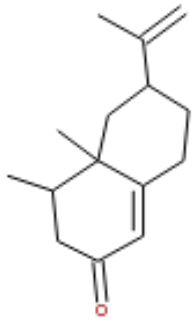

Cell ID	Cluster Center	Number of Compounds
124	0	10

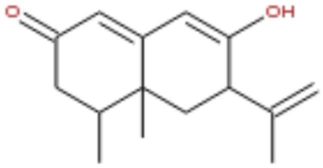

Cell ID	Cluster Center	Number of Compounds
124	0	10

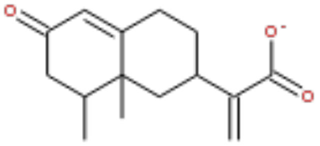

Cell ID	Cluster Center	Number of Compounds
124	0	10

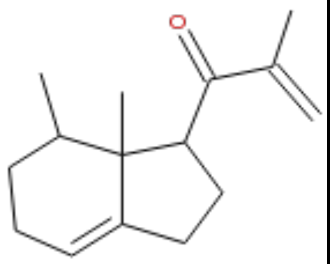

Cell ID	Cluster Center	Number of Compounds
125	1	7

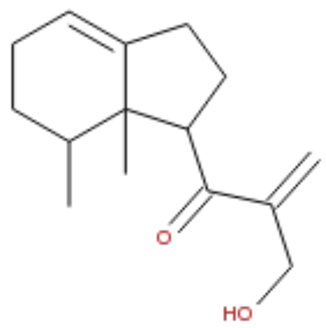

Cell ID	Cluster Center	Number of Compounds
125	0	7

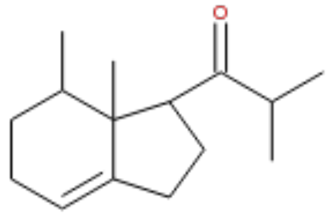

Cell ID	Cluster Center	Number of Compounds
125	0	7

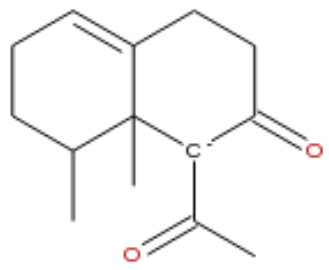

Cell ID	Cluster Center	Number of Compounds
125	0	7

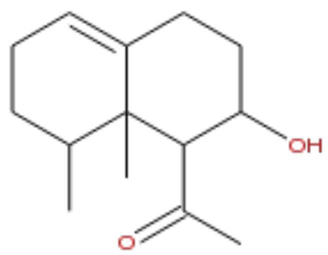

Cell ID	Cluster Center	Number of Compounds
125	0	7

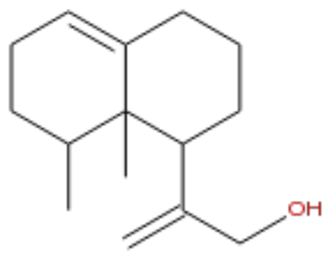

Cell ID	Cluster Center	Number of Compounds
125	0	7

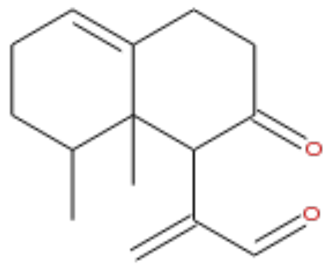

Cell ID	Cluster Center	Number of Compounds
125	0	7

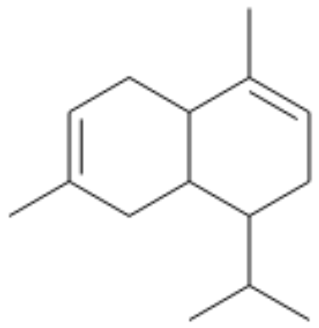

Cell ID	Cluster Center	Number of Compounds
127	1	13

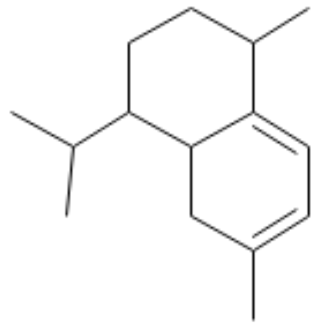

Cell ID	Cluster Center	Number of Compounds
127	0	13

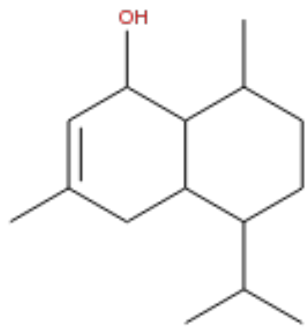

Cell ID	Cluster Center	Number of Compounds
127	0	13

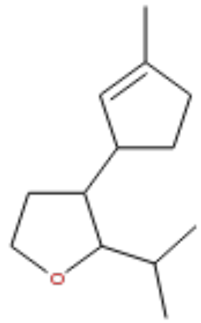

Cell ID	Cluster Center	Number of Compounds
127	0	13

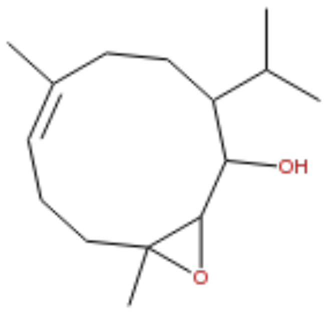

Cell ID	Cluster Center	Number of Compounds
127	0	13

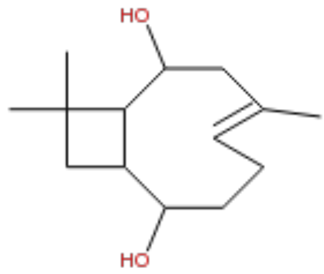

Cell ID	Cluster Center	Number of Compounds
127	0	13

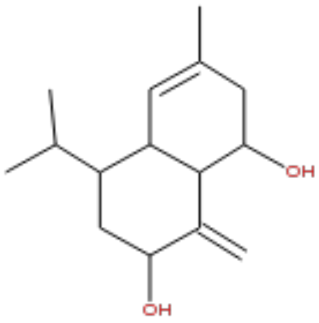

Cell ID	Cluster Center	Number of Compounds
127	0	13

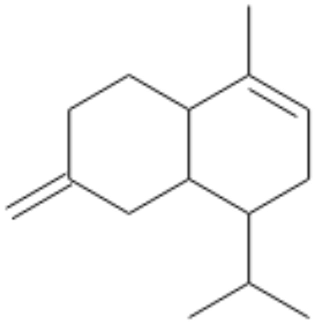

Cell ID	Cluster Center	Number of Compounds
127	0	13

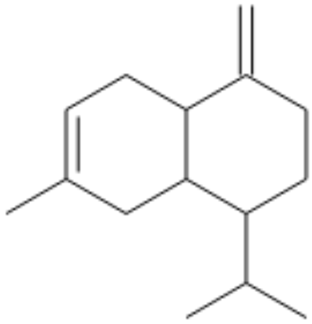

Cell ID	Cluster Center	Number of Compounds
127	0	13

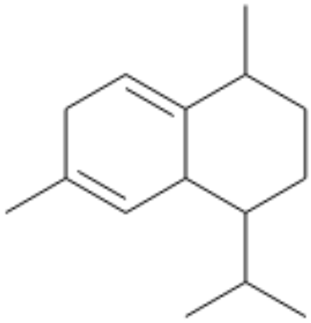

Cell ID	Cluster Center	Number of Compounds
127	0	13

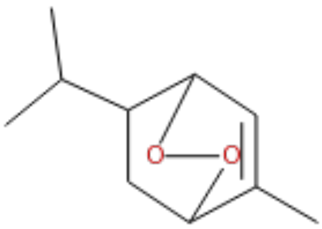

Cell ID	Cluster Center	Number of Compounds
127	0	13

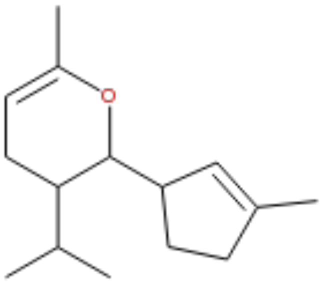

Cell ID	Cluster Center	Number of Compounds
127	0	13

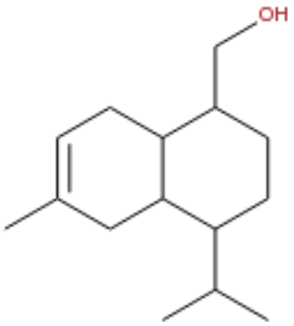

Cell ID	Cluster Center	Number of Compounds
127	0	13

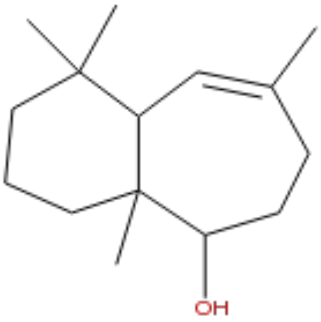

Cell ID	Cluster Center	Number of Compounds
128	1	4

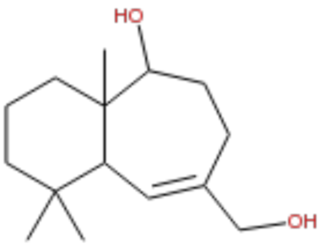

Cell ID	Cluster Center	Number of Compounds
128	0	4

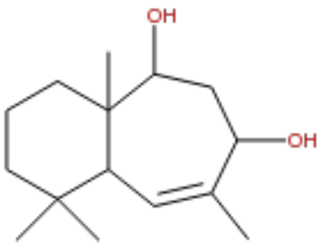

Cell ID	Cluster Center	Number of Compounds
128	0	4

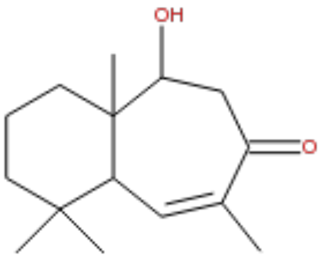

Cell ID	Cluster Center	Number of Compounds
128	0	4

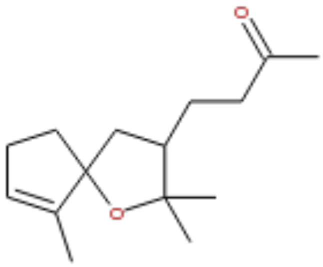

Cell ID	Cluster Center	Number of Compounds
130	1	1

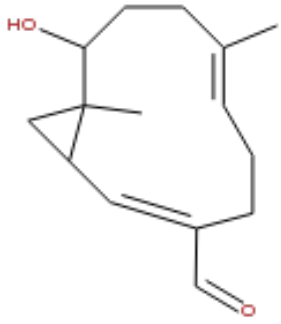

Cell ID	Cluster Center	Number of Compounds
131	1	1

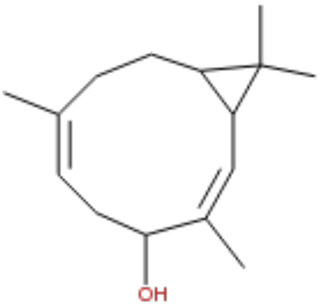

Cell ID	Cluster Center	Number of Compounds
132	1	3

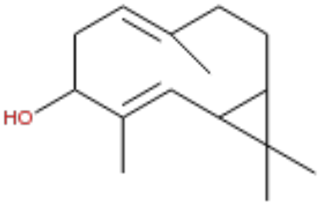

Cell ID	Cluster Center	Number of Compounds
132	0	3

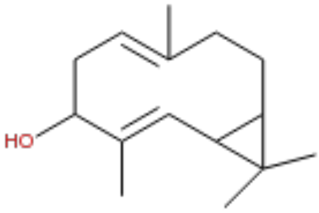

Cell ID	Cluster Center	Number of Compounds
132	0	3

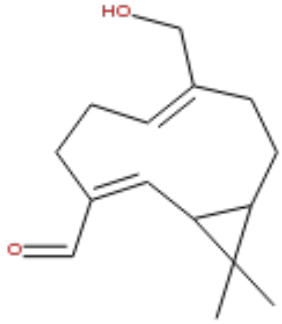

Cell ID	Cluster Center	Number of Compounds
133	1	4

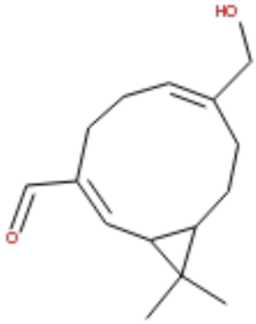

Cell ID	Cluster Center	Number of Compounds
133	0	4

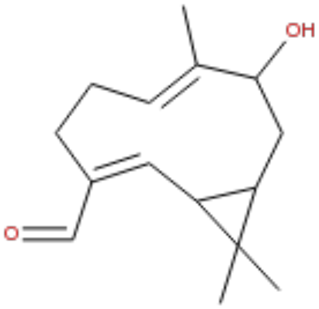

Cell ID	Cluster Center	Number of Compounds
133	0	4

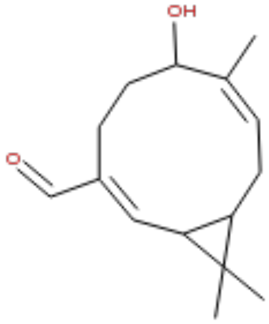

Cell ID	Cluster Center	Number of Compounds
133	0	4

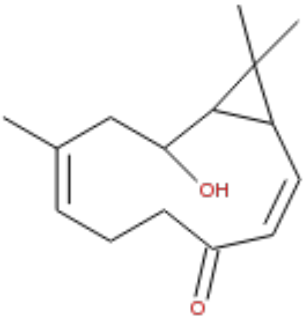

Cell ID	Cluster Center	Number of Compounds
134	1	2

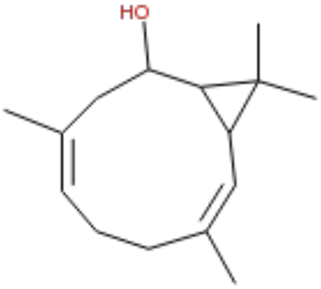

Cell ID	Cluster Center	Number of Compounds
134	0	2

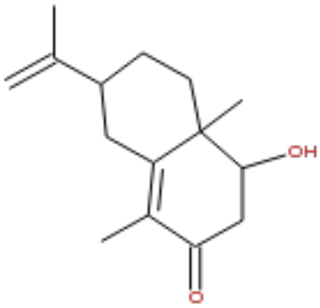

Cell ID	Cluster Center	Number of Compounds
135	1	4

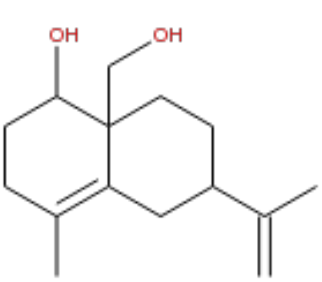

Cell ID	Cluster Center	Number of Compounds
135	0	4

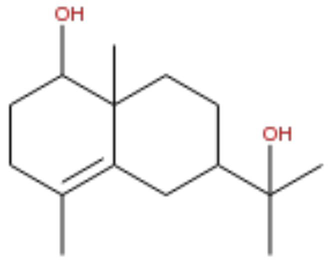

Cell ID	Cluster Center	Number of Compounds
135	0	4

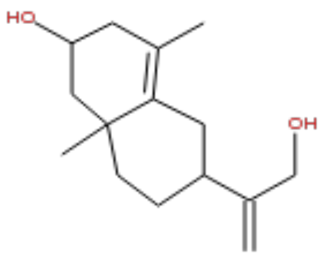

Cell ID	Cluster Center	Number of Compounds
135	0	4

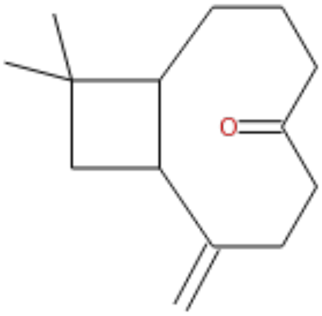

Cell ID	Cluster Center	Number of Compounds
138	1	11

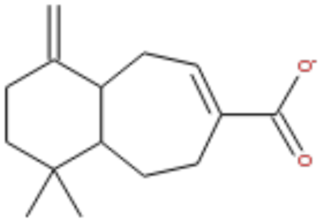

Cell ID	Cluster Center	Number of Compounds
138	0	11

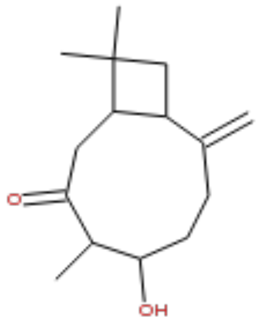

Cell ID	Cluster Center	Number of Compounds
138	0	11

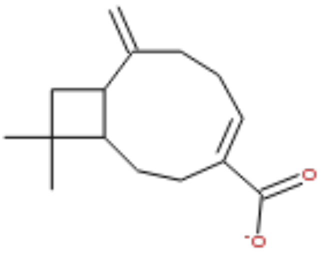

Cell ID	Cluster Center	Number of Compounds
138	0	11

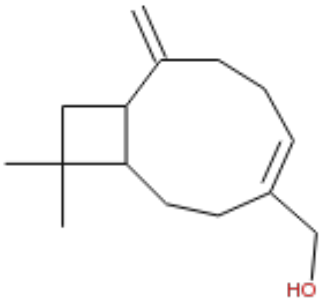

Cell ID	Cluster Center	Number of Compounds
138	0	11

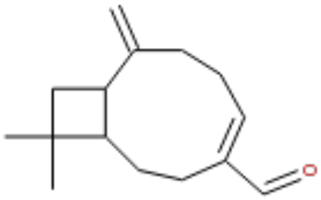

Cell ID	Cluster Center	Number of Compounds
138	0	11

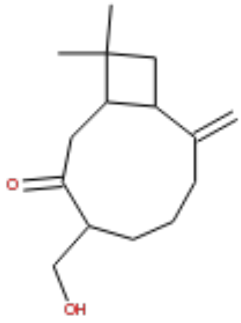

Cell ID	Cluster Center	Number of Compounds
138	0	11

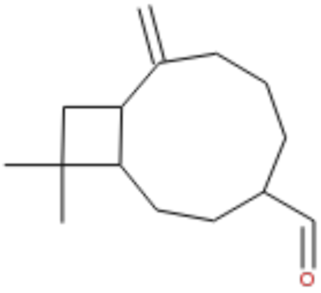

Cell ID	Cluster Center	Number of Compounds
138	0	11

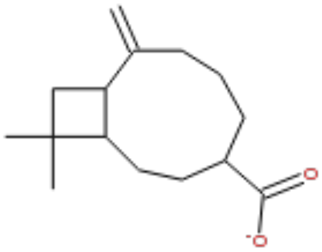

Cell ID	Cluster Center	Number of Compounds
138	0	11

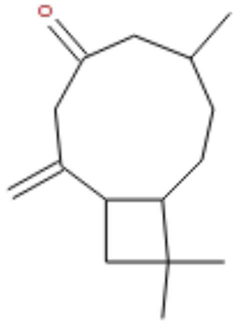

Cell ID	Cluster Center	Number of Compounds
138	0	11

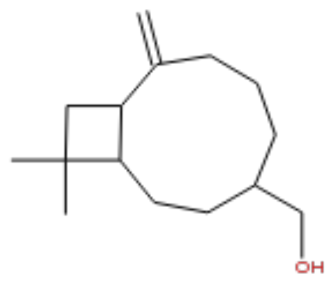

Cell ID	Cluster Center	Number of Compounds
138	0	11

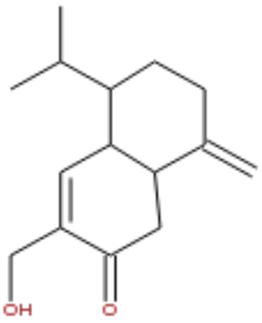

Cell ID	Cluster Center	Number of Compounds
139	1	3

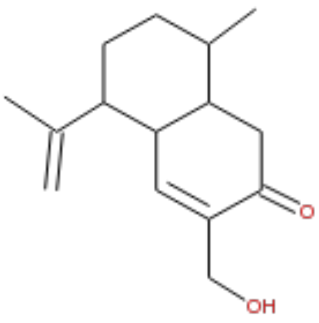

Cell ID	Cluster Center	Number of Compounds
139	0	3

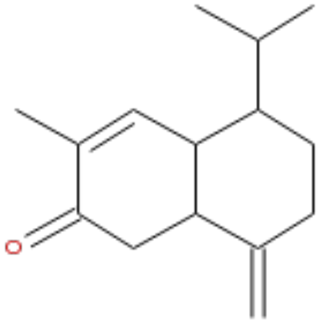

Cell ID	Cluster Center	Number of Compounds
139	0	3

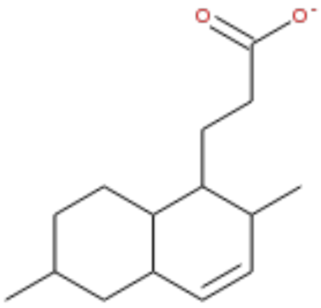

Cell ID	Cluster Center	Number of Compounds
140	1	3

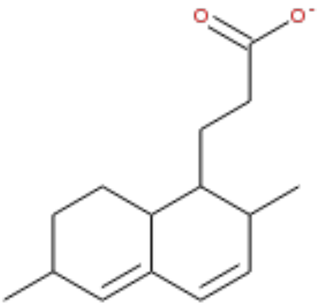

Cell ID	Cluster Center	Number of Compounds
140	0	3

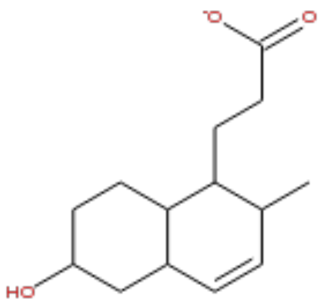

Cell ID	Cluster Center	Number of Compounds
140	0	3

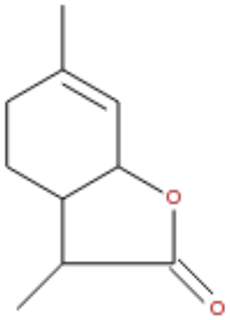

Cell ID	Cluster Center	Number of Compounds
141	1	6

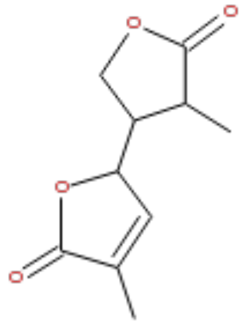

Cell ID	Cluster Center	Number of Compounds
141	0	6

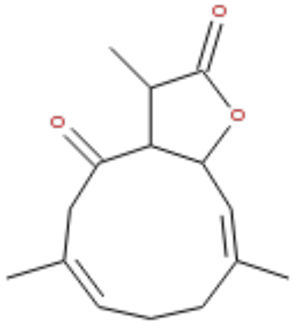

Cell ID	Cluster Center	Number of Compounds
141	0	6

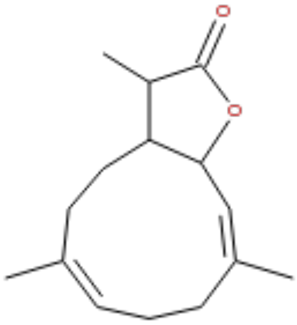

Cell ID	Cluster Center	Number of Compounds
141	0	6

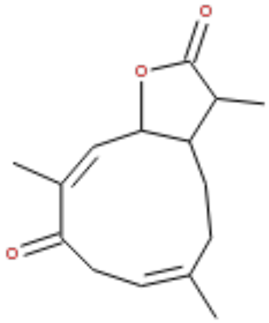

Cell ID	Cluster Center	Number of Compounds
141	0	6

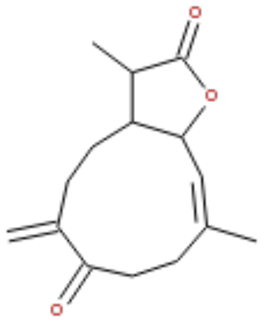

Cell ID	Cluster Center	Number of Compounds
141	0	6

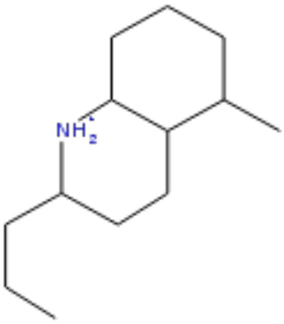

Cell ID	Cluster Center	Number of Compounds
142	1	6

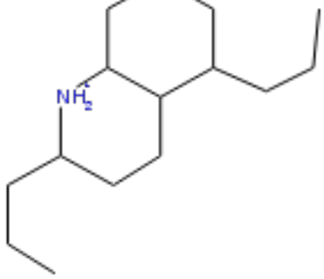

Cell ID	Cluster Center	Number of Compounds
142	0	6

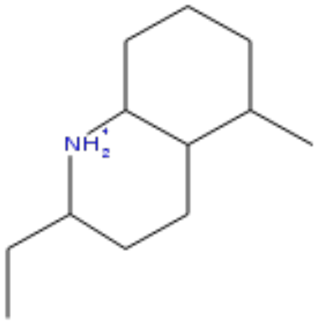

Cell ID	Cluster Center	Number of Compounds
142	0	6

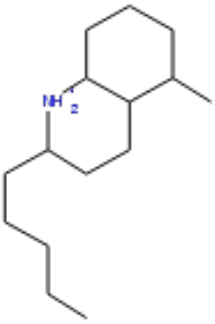

Cell ID	Cluster Center	Number of Compounds
142	0	6

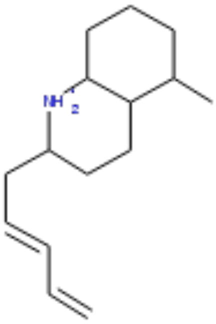

Cell ID	Cluster Center	Number of Compounds
142	0	6

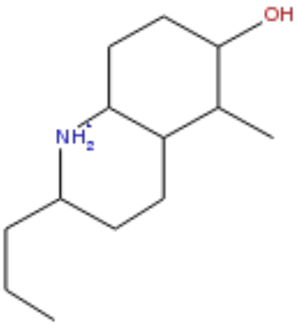

Cell ID	Cluster Center	Number of Compounds
142	0	6

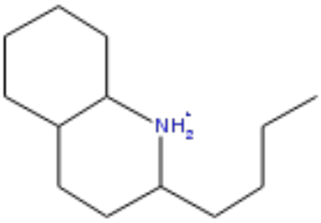

Cell ID	Cluster Center	Number of Compounds
143	1	4

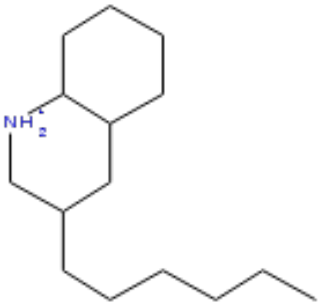

Cell ID	Cluster Center	Number of Compounds
143	0	4

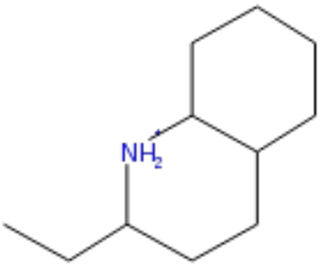

Cell ID	Cluster Center	Number of Compounds
143	0	4

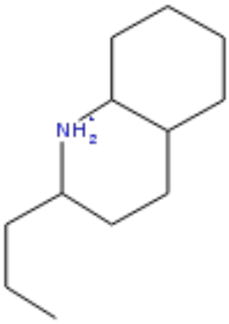

Cell ID	Cluster Center	Number of Compounds
143	0	4

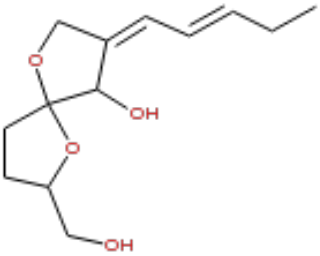

Cell ID	Cluster Center	Number of Compounds
145	1	2

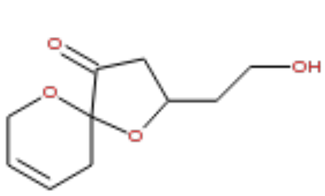

Cell ID	Cluster Center	Number of Compounds
145	0	2

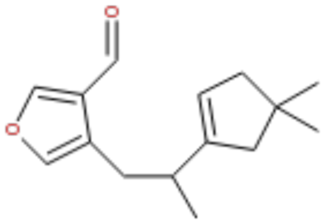

Cell ID	Cluster Center	Number of Compounds
147	1	1

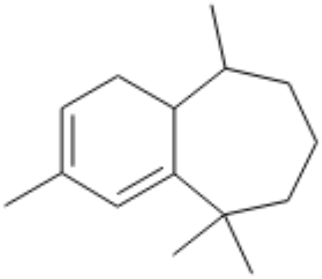

Cell ID	Cluster Center	Number of Compounds
149	1	9

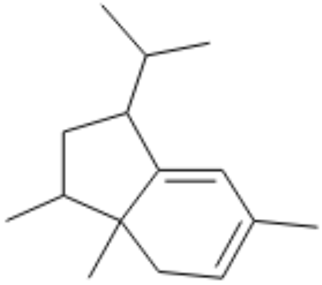

Cell ID	Cluster Center	Number of Compounds
149	0	9

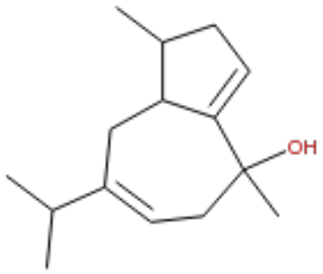

Cell ID	Cluster Center	Number of Compounds
149	0	9

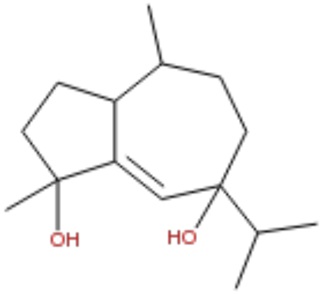

Cell ID	Cluster Center	Number of Compounds
149	0	9

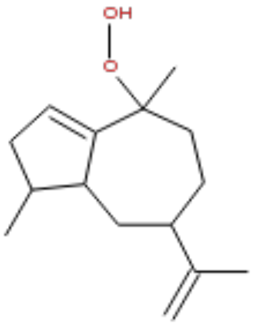

Cell ID	Cluster Center	Number of Compounds
149	0	9

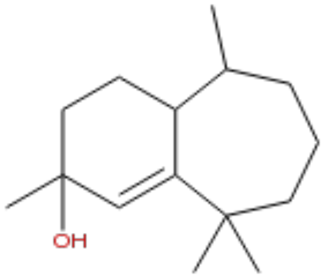

Cell ID	Cluster Center	Number of Compounds
149	0	9

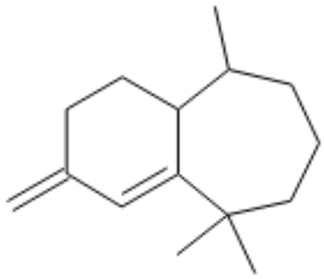

Cell ID	Cluster Center	Number of Compounds
149	0	9

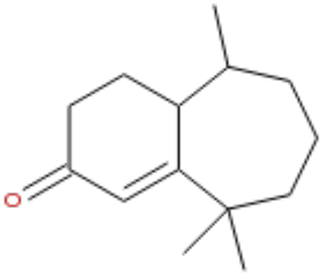

Cell ID	Cluster Center	Number of Compounds
149	0	9

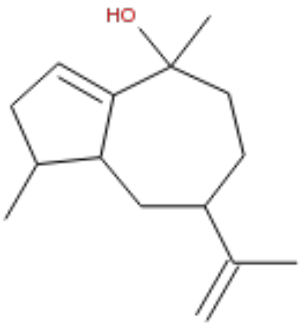

Cell ID	Cluster Center	Number of Compounds
149	0	9

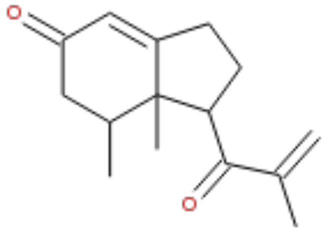

Cell ID	Cluster Center	Number of Compounds
150	1	3

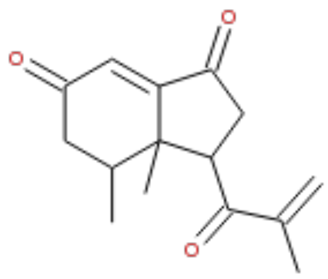

Cell ID	Cluster Center	Number of Compounds
150	0	3

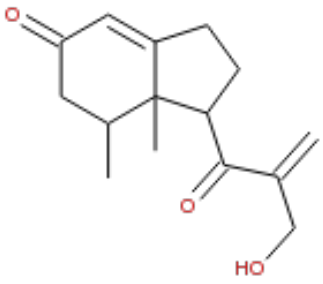

Cell ID	Cluster Center	Number of Compounds
150	0	3

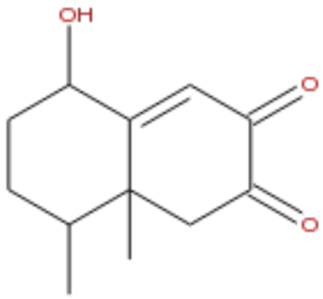

Cell ID	Cluster Center	Number of Compounds
151	1	11

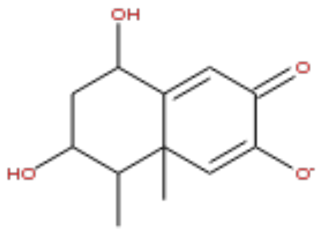

Cell ID	Cluster Center	Number of Compounds
151	0	11

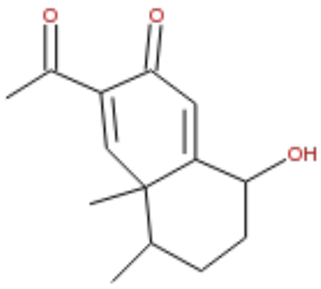

Cell ID	Cluster Center	Number of Compounds
151	0	11

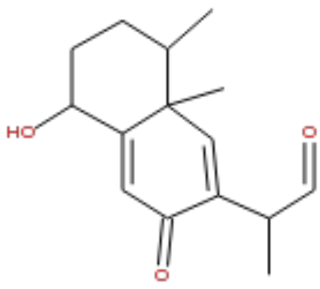

Cell ID	Cluster Center	Number of Compounds
151	0	11

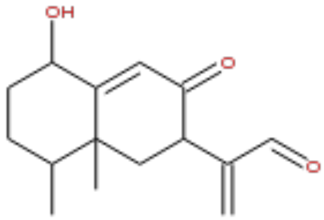

Cell ID	Cluster Center	Number of Compounds
151	0	11

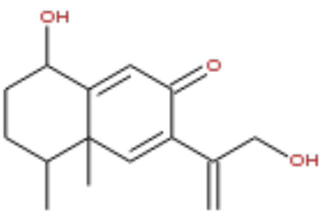

Cell ID	Cluster Center	Number of Compounds
151	0	11

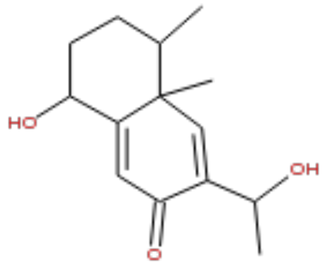

Cell ID	Cluster Center	Number of Compounds
151	0	11

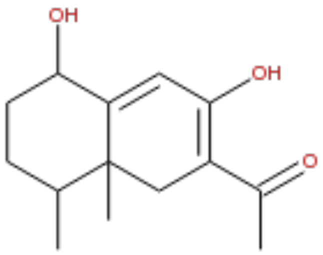

Cell ID	Cluster Center	Number of Compounds
151	0	11

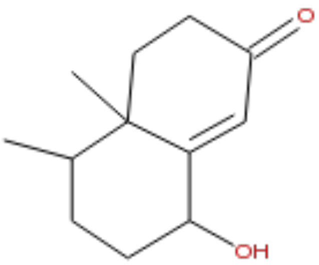

Cell ID	Cluster Center	Number of Compounds
151	0	11

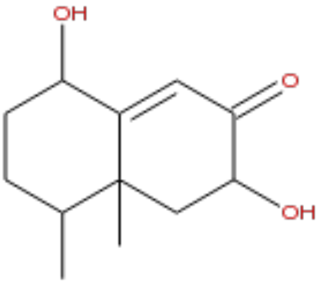

Cell ID	Cluster Center	Number of Compounds
151	0	11

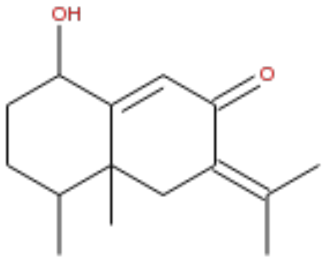

Cell ID	Cluster Center	Number of Compounds
151	0	11

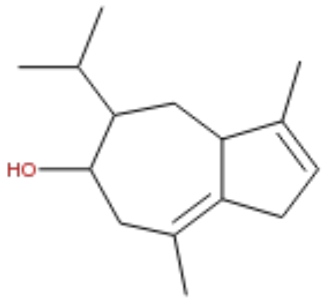

Cell ID	Cluster Center	Number of Compounds
152	1	2

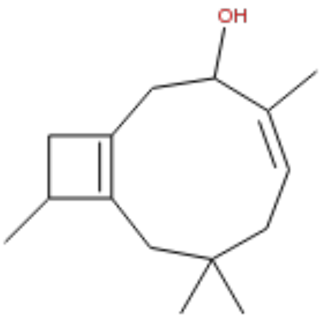

Cell ID	Cluster Center	Number of Compounds
152	0	2

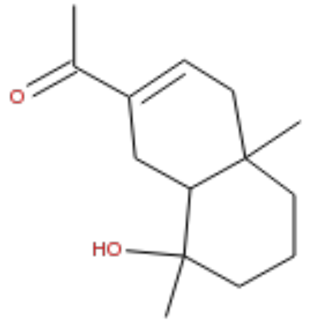

Cell ID	Cluster Center	Number of Compounds
153	1	6

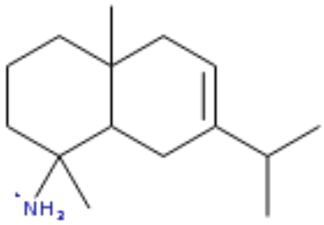

Cell ID	Cluster Center	Number of Compounds
153	0	6

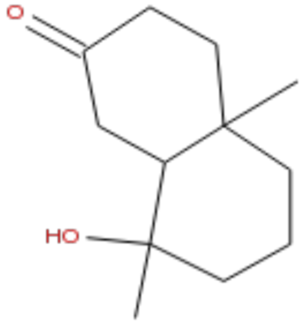

Cell ID	Cluster Center	Number of Compounds
153	0	6

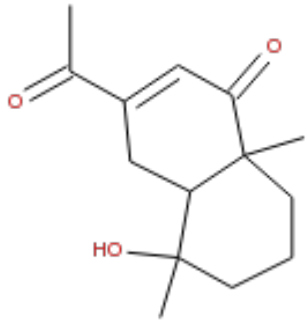

Cell ID	Cluster Center	Number of Compounds
153	0	6

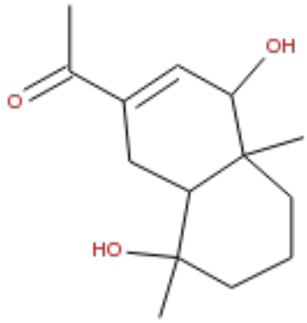

Cell ID	Cluster Center	Number of Compounds
153	0	6

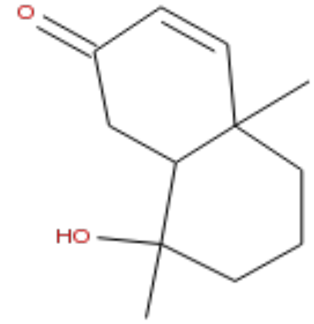

Cell ID	Cluster Center	Number of Compounds
153	0	6

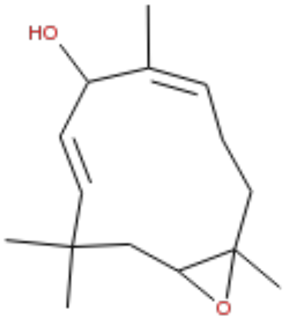

Cell ID	Cluster Center	Number of Compounds
154	1	3

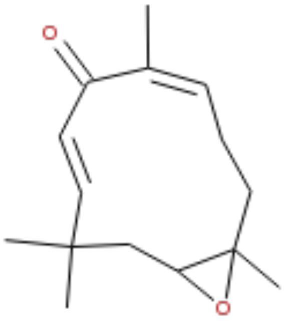

Cell ID	Cluster Center	Number of Compounds
154	0	3

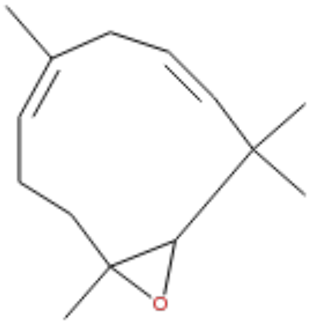

Cell ID	Cluster Center	Number of Compounds
154	0	3

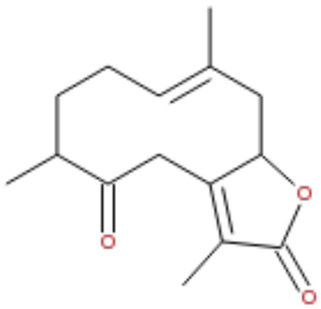

Cell ID	Cluster Center	Number of Compounds
155	1	2

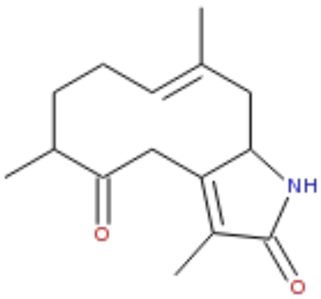

Cell ID	Cluster Center	Number of Compounds
155	0	2

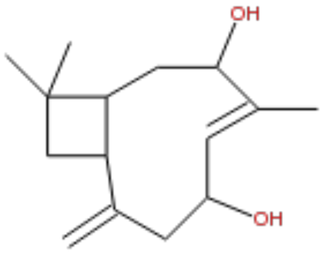

Cell ID	Cluster Center	Number of Compounds
156	1	7

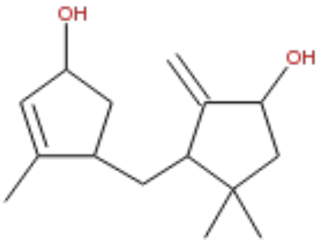

Cell ID	Cluster Center	Number of Compounds
156	0	7

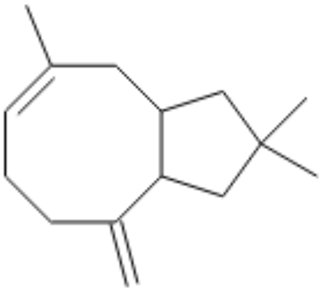

Cell ID	Cluster Center	Number of Compounds
156	0	7

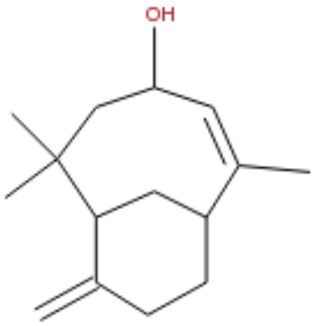

Cell ID	Cluster Center	Number of Compounds
156	0	7

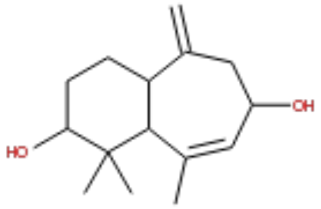

Cell ID	Cluster Center	Number of Compounds
156	0	7

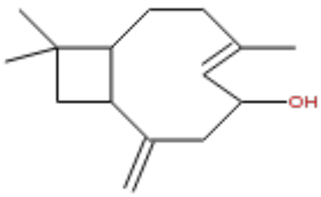

Cell ID	Cluster Center	Number of Compounds
156	0	7

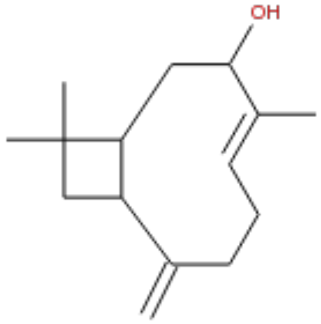

Cell ID	Cluster Center	Number of Compounds
156	0	7

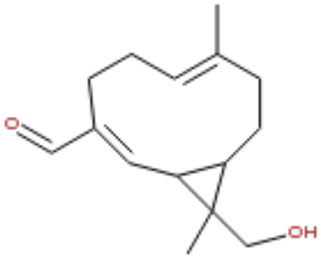

Cell ID	Cluster Center	Number of Compounds
157	1	4

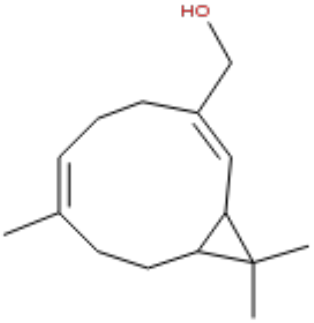

Cell ID	Cluster Center	Number of Compounds
157	0	4

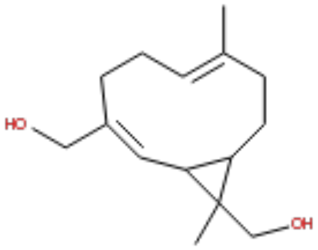

Cell ID	Cluster Center	Number of Compounds
157	0	4

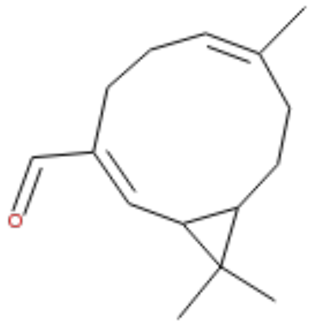

Cell ID	Cluster Center	Number of Compounds
157	0	4

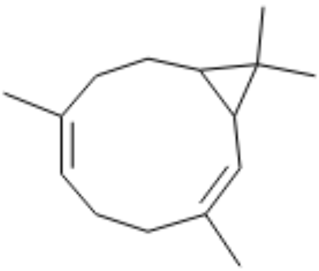

Cell ID	Cluster Center	Number of Compounds
158	1	4

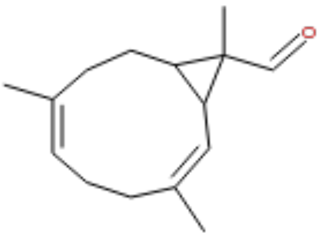

Cell ID	Cluster Center	Number of Compounds
158	0	4

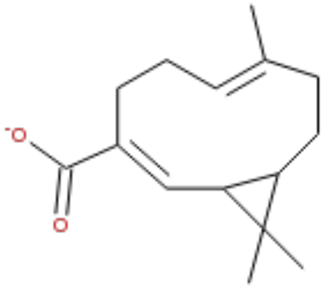

Cell ID	Cluster Center	Number of Compounds
158	0	4

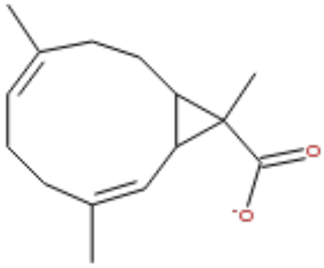

Cell ID	Cluster Center	Number of Compounds
158	0	4

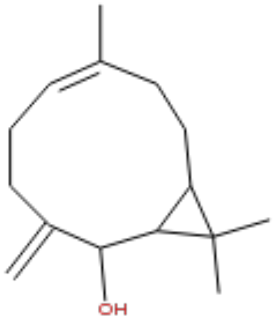

Cell ID	Cluster Center	Number of Compounds
159	1	9

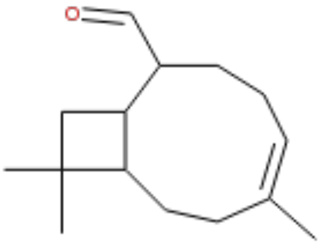

Cell ID	Cluster Center	Number of Compounds
159	0	9

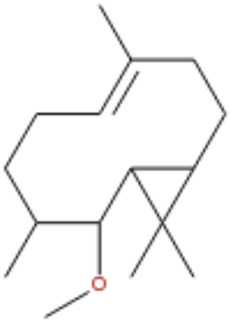

Cell ID	Cluster Center	Number of Compounds
159	0	9

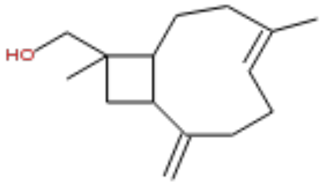

Cell ID	Cluster Center	Number of Compounds
159	0	9

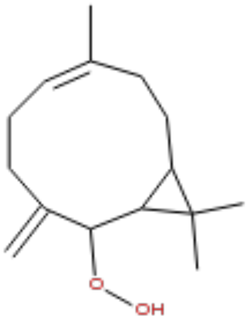

Cell ID	Cluster Center	Number of Compounds
159	0	9

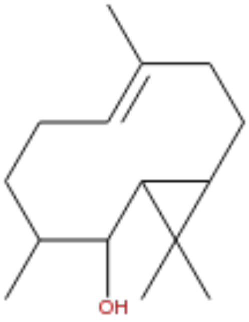

Cell ID	Cluster Center	Number of Compounds
159	0	9

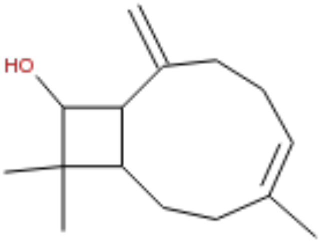

Cell ID	Cluster Center	Number of Compounds
159	0	9

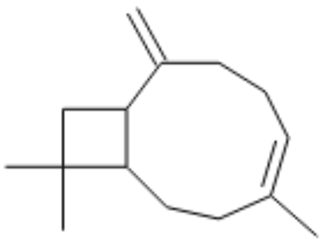

Cell ID	Cluster Center	Number of Compounds
159	0	9

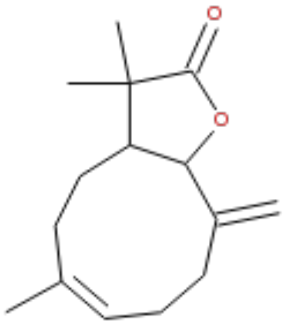

Cell ID	Cluster Center	Number of Compounds
159	0	9

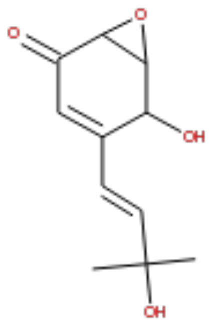

Cell ID	Cluster Center	Number of Compounds
160	1	1

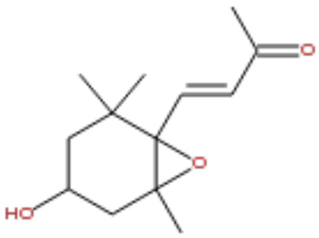

Cell ID	Cluster Center	Number of Compounds
161	1	6

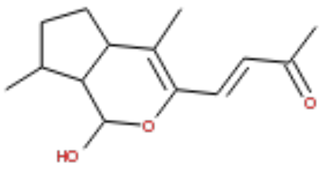

Cell ID	Cluster Center	Number of Compounds
161	0	6

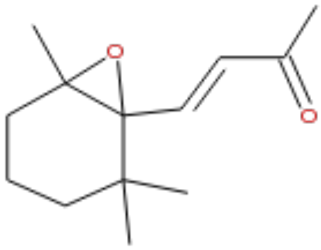

Cell ID	Cluster Center	Number of Compounds
161	0	6

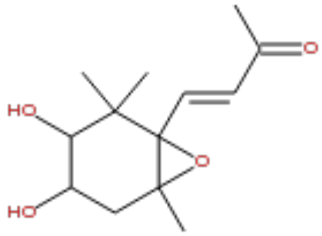

Cell ID	Cluster Center	Number of Compounds
161	0	6

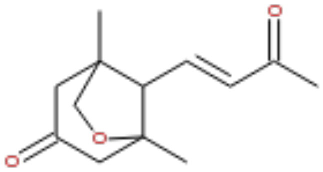

Cell ID	Cluster Center	Number of Compounds
161	0	6

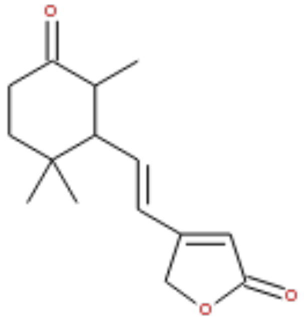

Cell ID	Cluster Center	Number of Compounds
161	0	6

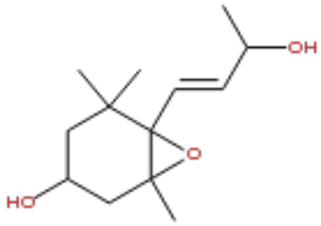

Cell ID	Cluster Center	Number of Compounds
162	1	6

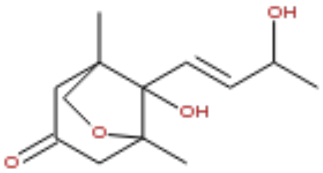

Cell ID	Cluster Center	Number of Compounds
162	0	6

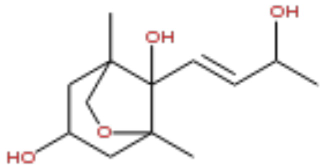

Cell ID	Cluster Center	Number of Compounds
162	0	6

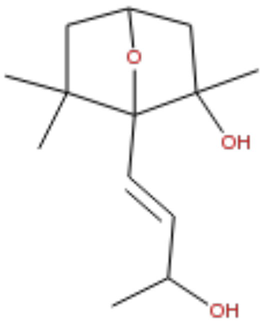

Cell ID	Cluster Center	Number of Compounds
162	0	6

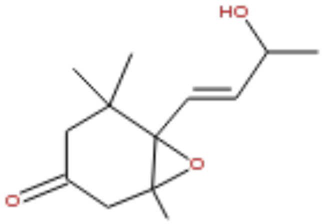

Cell ID	Cluster Center	Number of Compounds
162	0	6

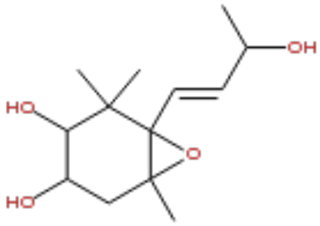

Cell ID	Cluster Center	Number of Compounds
162	0	6

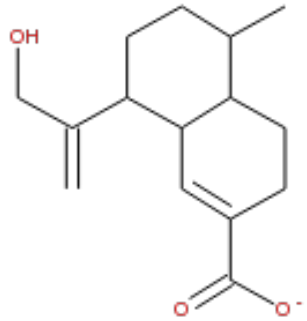

Cell ID	Cluster Center	Number of Compounds
164	1	3

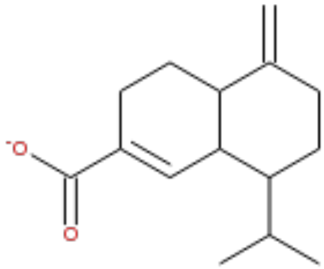

Cell ID	Cluster Center	Number of Compounds
164	0	3

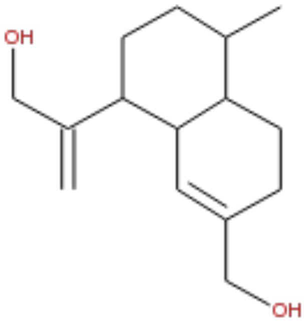

Cell ID	Cluster Center	Number of Compounds
164	0	3

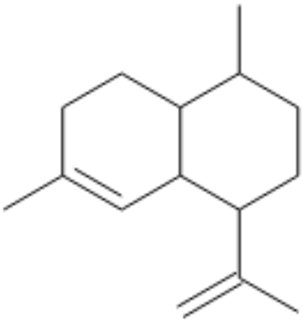

Cell ID	Cluster Center	Number of Compounds
165	1	15

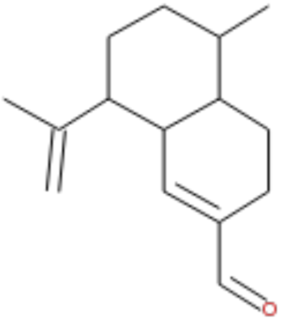

Cell ID	Cluster Center	Number of Compounds
165	0	15

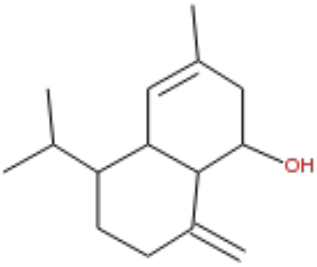

Cell ID	Cluster Center	Number of Compounds
165	0	15

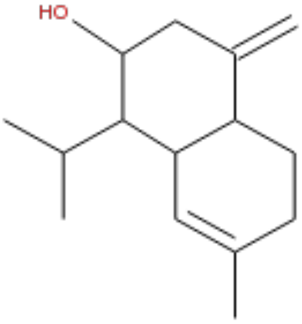

Cell ID	Cluster Center	Number of Compounds
165	0	15

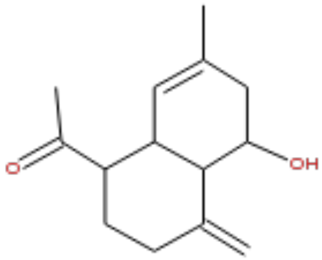

Cell ID	Cluster Center	Number of Compounds
165	0	15

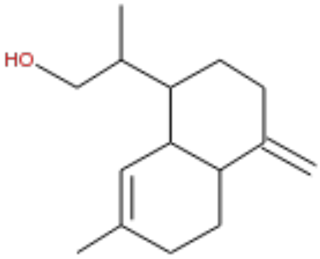

Cell ID	Cluster Center	Number of Compounds
165	0	15

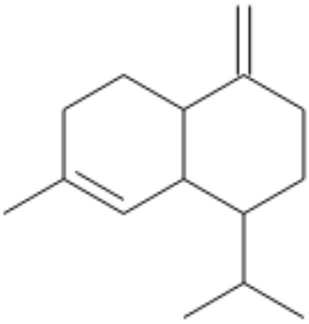

Cell ID	Cluster Center	Number of Compounds
165	0	15

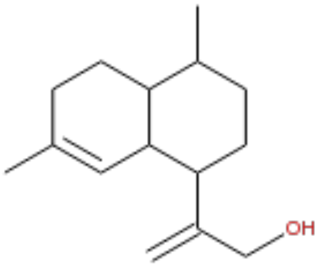

Cell ID	Cluster Center	Number of Compounds
165	0	15

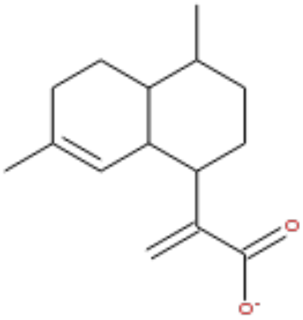

Cell ID	Cluster Center	Number of Compounds
165	0	15

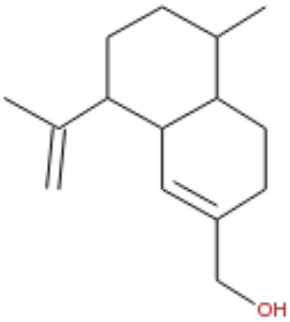

Cell ID	Cluster Center	Number of Compounds
165	0	15

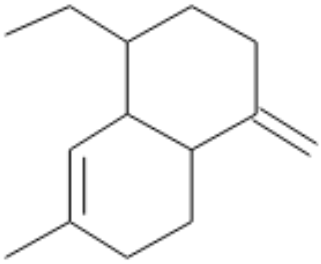

Cell ID	Cluster Center	Number of Compounds
165	0	15

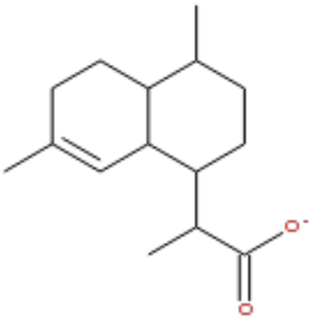

Cell ID	Cluster Center	Number of Compounds
165	0	15

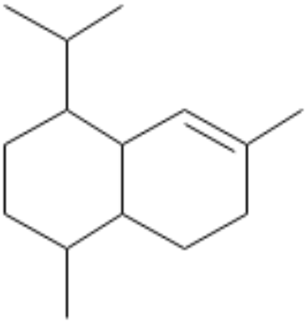

Cell ID	Cluster Center	Number of Compounds
165	0	15

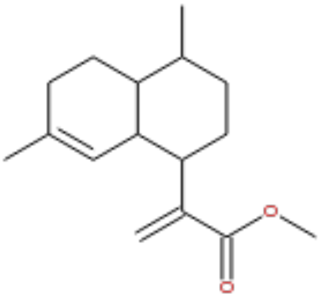

Cell ID	Cluster Center	Number of Compounds
165	0	15

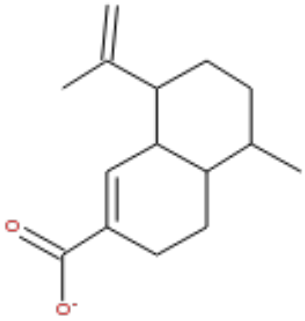

Cell ID	Cluster Center	Number of Compounds
165	0	15

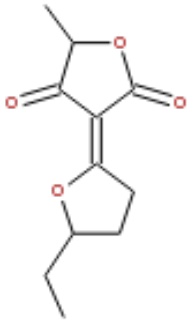

Cell ID	Cluster Center	Number of Compounds
166	1	3

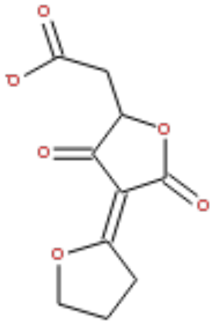

Cell ID	Cluster Center	Number of Compounds
166	0	3

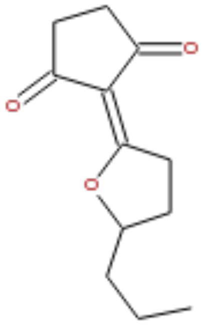

Cell ID	Cluster Center	Number of Compounds
166	0	3

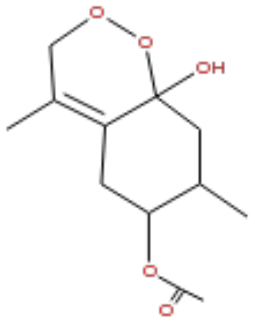

Cell ID	Cluster Center	Number of Compounds
167	1	4

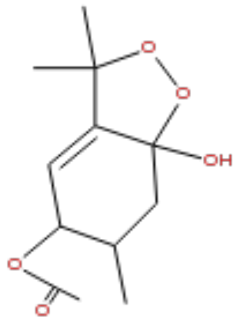

Cell ID	Cluster Center	Number of Compounds
167	0	4

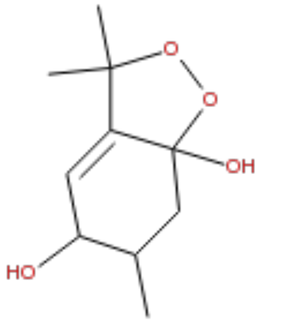

Cell ID	Cluster Center	Number of Compounds
167	0	4

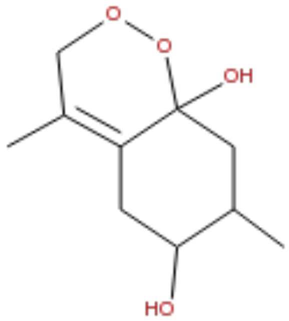

Cell ID	Cluster Center	Number of Compounds
167	0	4

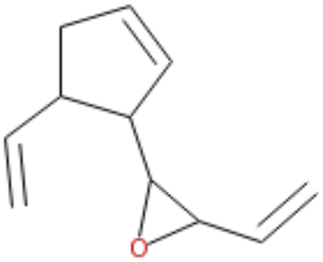

Cell ID	Cluster Center	Number of Compounds
168	1	6

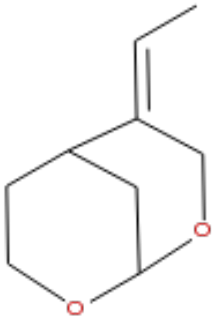

Cell ID	Cluster Center	Number of Compounds
168	0	6

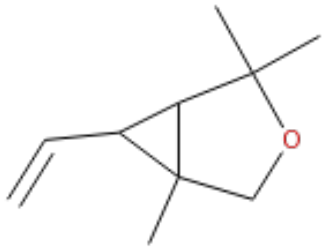

Cell ID	Cluster Center	Number of Compounds
168	0	6

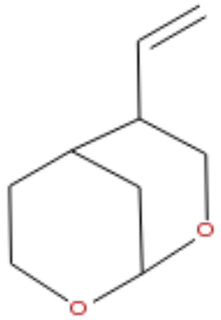

Cell ID	Cluster Center	Number of Compounds
168	0	6

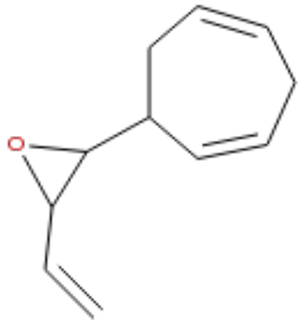

Cell ID	Cluster Center	Number of Compounds
168	0	6

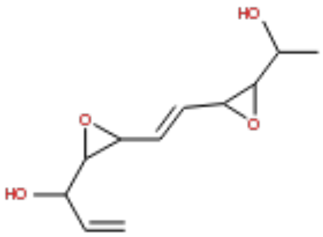

Cell ID	Cluster Center	Number of Compounds
168	0	6

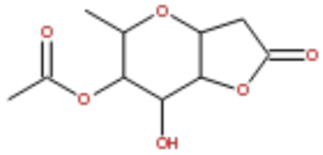

Cell ID	Cluster Center	Number of Compounds
169	1	6

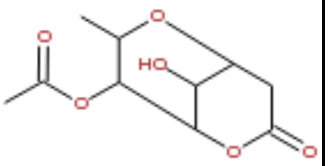

Cell ID	Cluster Center	Number of Compounds
169	0	6

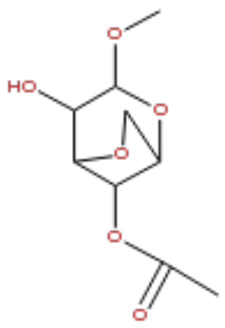

Cell ID	Cluster Center	Number of Compounds
169	0	6

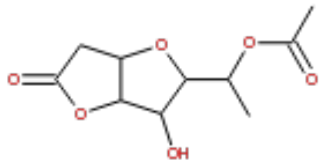

Cell ID	Cluster Center	Number of Compounds
169	0	6

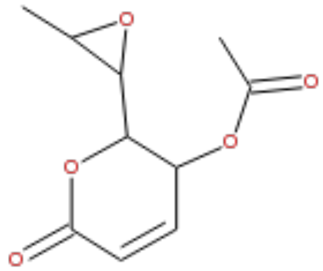

Cell ID	Cluster Center	Number of Compounds
169	0	6

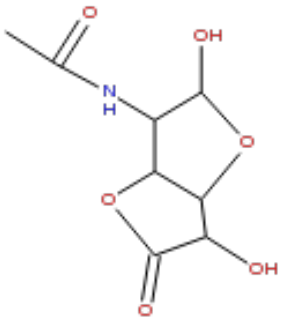

Cell ID	Cluster Center	Number of Compounds
169	0	6

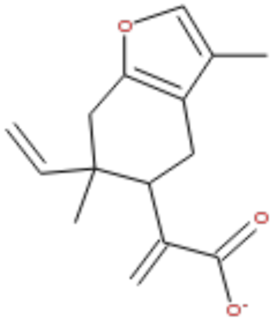

Cell ID	Cluster Center	Number of Compounds
173	1	3

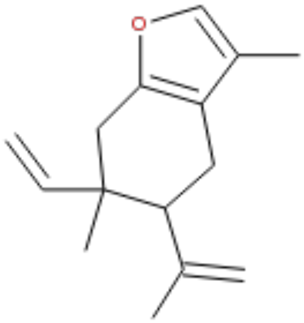

Cell ID	Cluster Center	Number of Compounds
173	0	3

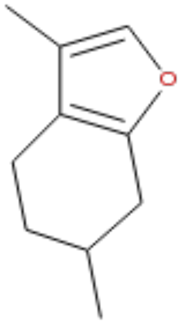

Cell ID	Cluster Center	Number of Compounds
173	0	3

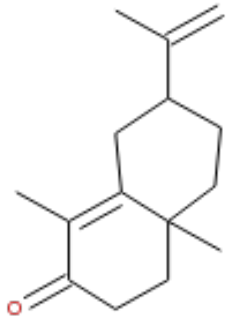

Cell ID	Cluster Center	Number of Compounds
174	1	6

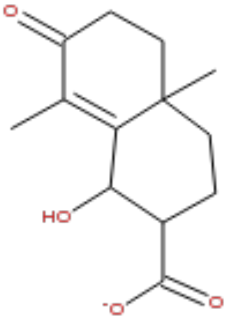

Cell ID	Cluster Center	Number of Compounds
174	0	6

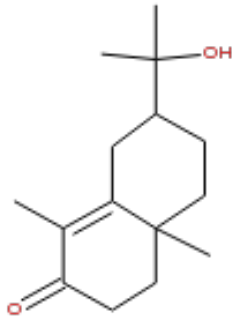

Cell ID	Cluster Center	Number of Compounds
174	0	6

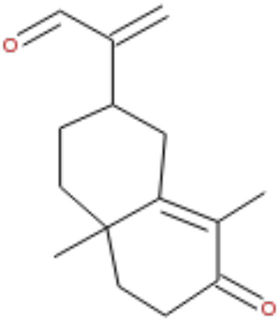

Cell ID	Cluster Center	Number of Compounds
174	0	6

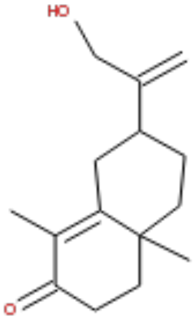

Cell ID	Cluster Center	Number of Compounds
174	0	6

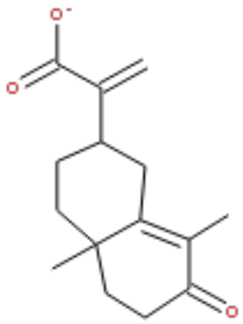

Cell ID	Cluster Center	Number of Compounds
174	0	6

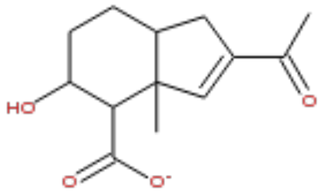

Cell ID	Cluster Center	Number of Compounds
175	1	4

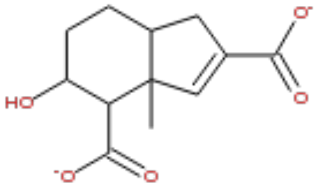

Cell ID	Cluster Center	Number of Compounds
175	0	4

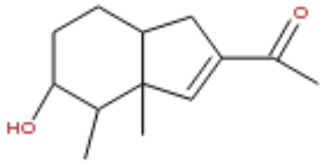

Cell ID	Cluster Center	Number of Compounds
175	0	4

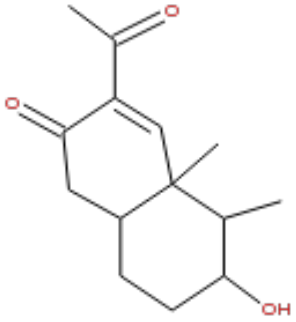

Cell ID	Cluster Center	Number of Compounds
175	0	4

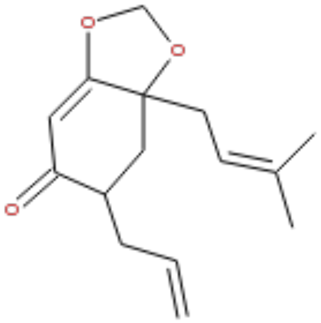

Cell ID	Cluster Center	Number of Compounds
177	1	2

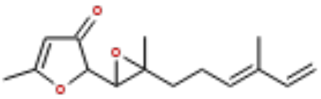

Cell ID	Cluster Center	Number of Compounds
177	0	2

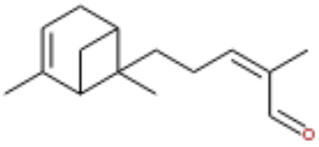

Cell ID	Cluster Center	Number of Compounds
178	1	5

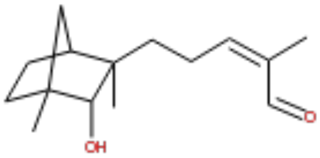

Cell ID	Cluster Center	Number of Compounds
178	0	5

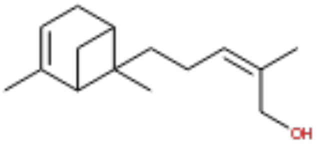

Cell ID	Cluster Center	Number of Compounds
178	0	5

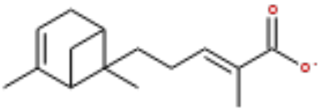

Cell ID	Cluster Center	Number of Compounds
178	0	5

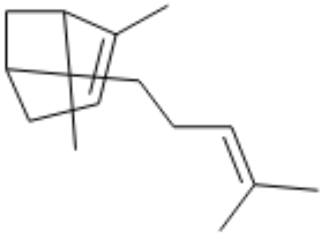

Cell ID	Cluster Center	Number of Compounds
178	0	5

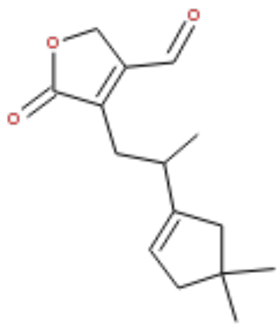

Cell ID	Cluster Center	Number of Compounds
179	1	1

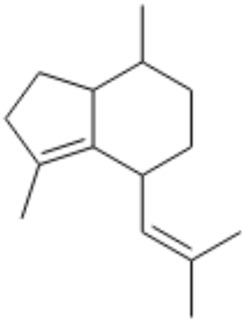

Cell ID	Cluster Center	Number of Compounds
180	1	9

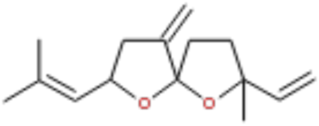

Cell ID	Cluster Center	Number of Compounds
180	0	9

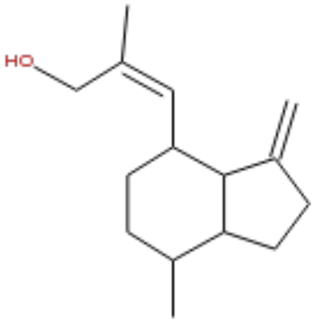

Cell ID	Cluster Center	Number of Compounds
180	0	9

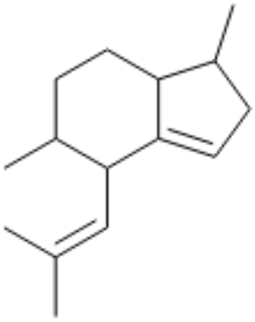

Cell ID	Cluster Center	Number of Compounds
180	0	9

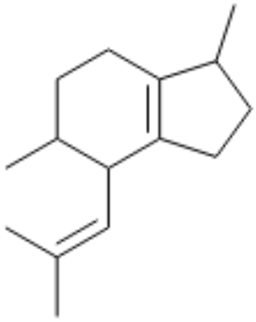

Cell ID	Cluster Center	Number of Compounds
180	0	9

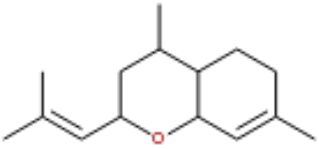

Cell ID	Cluster Center	Number of Compounds
180	0	9

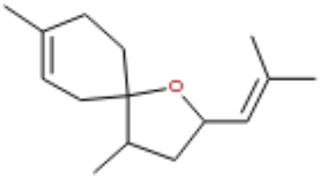

Cell ID	Cluster Center	Number of Compounds
180	0	9

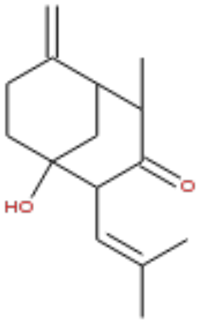

Cell ID	Cluster Center	Number of Compounds
180	0	9

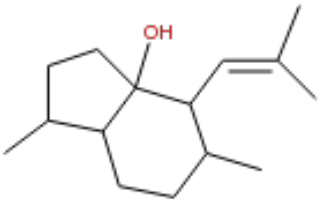

Cell ID	Cluster Center	Number of Compounds
180	0	9

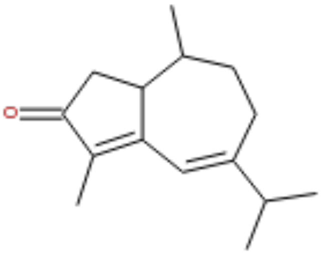

Cell ID	Cluster Center	Number of Compounds
181	1	5

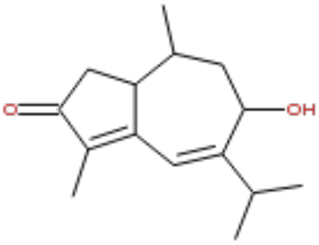

Cell ID	Cluster Center	Number of Compounds
181	0	5

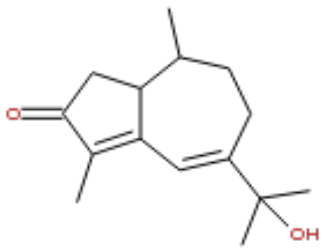

Cell ID	Cluster Center	Number of Compounds
181	0	5

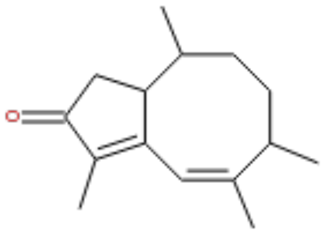

Cell ID	Cluster Center	Number of Compounds
181	0	5

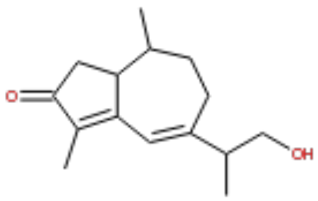

Cell ID	Cluster Center	Number of Compounds
181	0	5

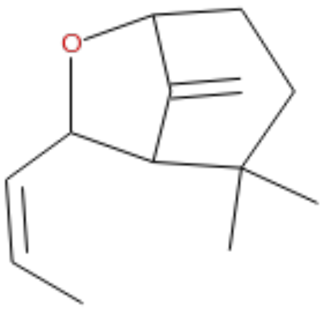

Cell ID	Cluster Center	Number of Compounds
182	1	6

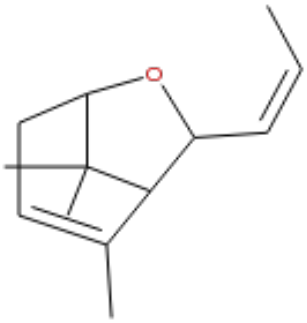

Cell ID	Cluster Center	Number of Compounds
182	0	6

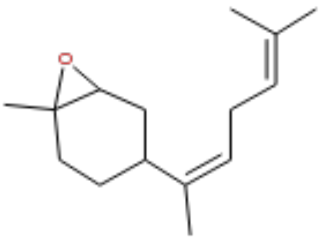

Cell ID	Cluster Center	Number of Compounds
182	0	6

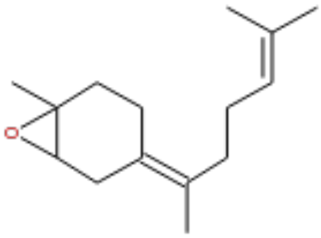

Cell ID	Cluster Center	Number of Compounds
182	0	6

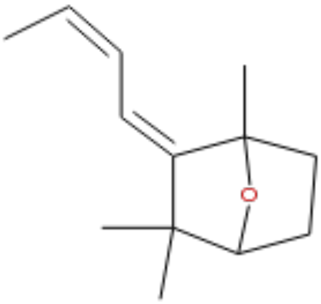

Cell ID	Cluster Center	Number of Compounds
182	0	6

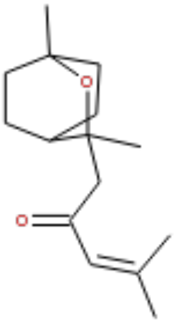

Cell ID	Cluster Center	Number of Compounds
182	0	6

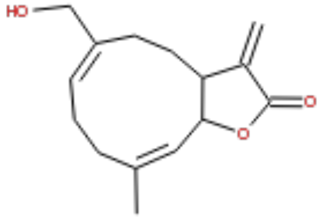

Cell ID	Cluster Center	Number of Compounds
183	1	6

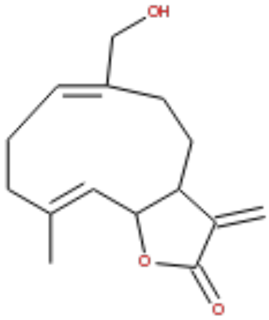

Cell ID	Cluster Center	Number of Compounds
183	0	6

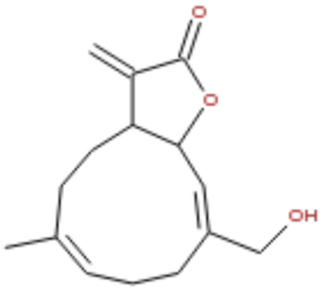

Cell ID	Cluster Center	Number of Compounds
183	0	6

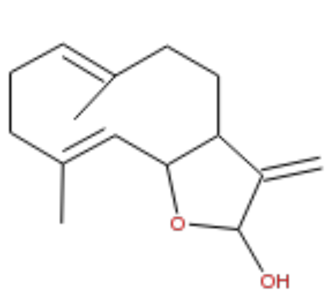

Cell ID	Cluster Center	Number of Compounds
183	0	6

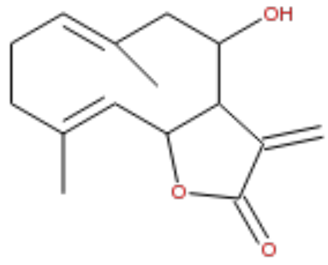

Cell ID	Cluster Center	Number of Compounds
183	0	6

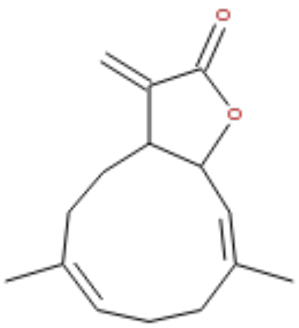

Cell ID	Cluster Center	Number of Compounds
183	0	6

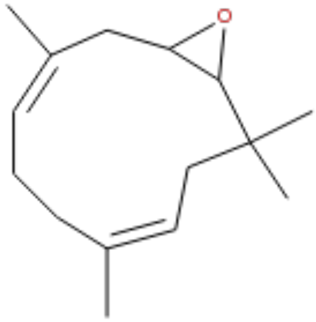

Cell ID	Cluster Center	Number of Compounds
184	1	5

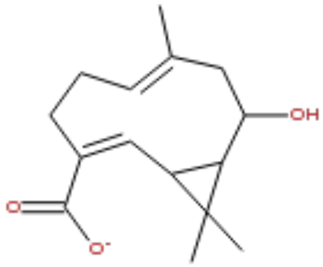

Cell ID	Cluster Center	Number of Compounds
184	0	5

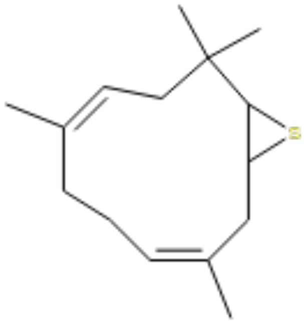

Cell ID	Cluster Center	Number of Compounds
184	0	5

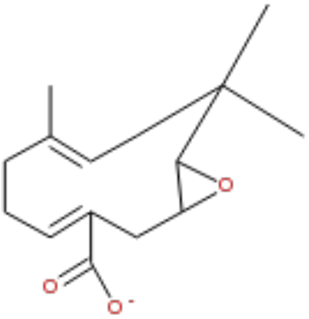

Cell ID	Cluster Center	Number of Compounds
184	0	5

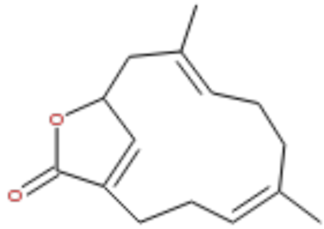

Cell ID	Cluster Center	Number of Compounds
184	0	5

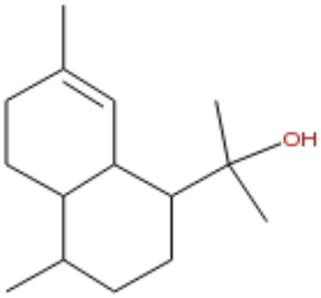

Cell ID	Cluster Center	Number of Compounds
189	1	5

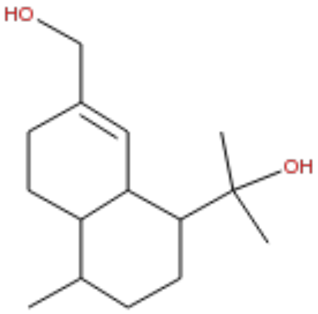

Cell ID	Cluster Center	Number of Compounds
189	0	5

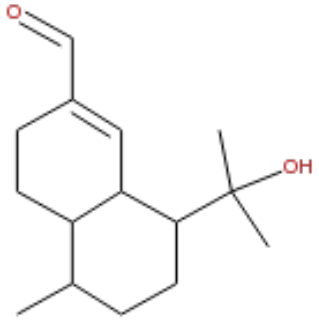

Cell ID	Cluster Center	Number of Compounds
189	0	5

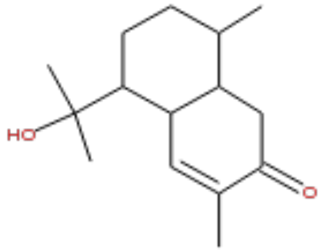

Cell ID	Cluster Center	Number of Compounds
189	0	5

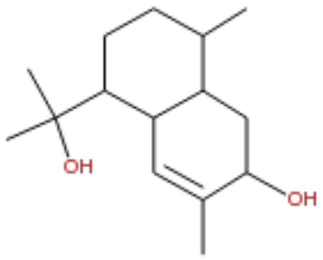

Cell ID	Cluster Center	Number of Compounds
189	0	5

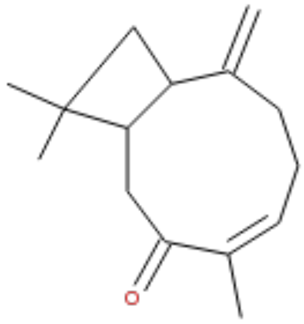

Cell ID	Cluster Center	Number of Compounds
190	1	7

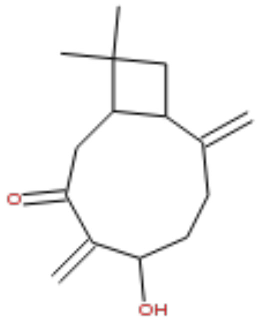

Cell ID	Cluster Center	Number of Compounds
190	0	7

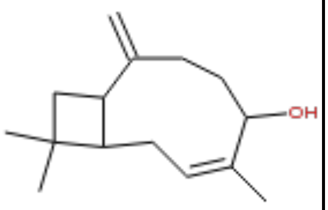

Cell ID	Cluster Center	Number of Compounds
190	0	7

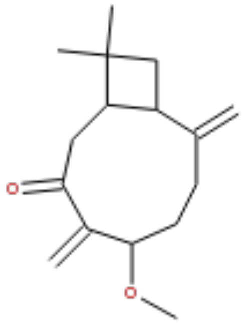

Cell ID	Cluster Center	Number of Compounds
190	0	7

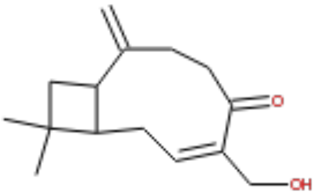

Cell ID	Cluster Center	Number of Compounds
190	0	7

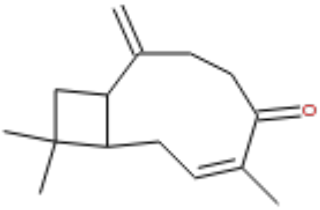

Cell ID	Cluster Center	Number of Compounds
190	0	7

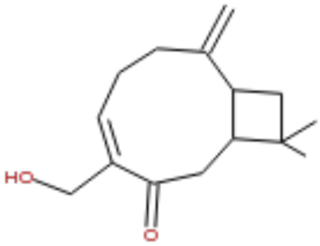

Cell ID	Cluster Center	Number of Compounds
190	0	7

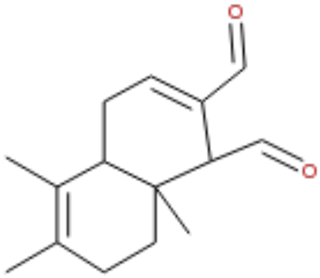

Cell ID	Cluster Center	Number of Compounds
193	1	5

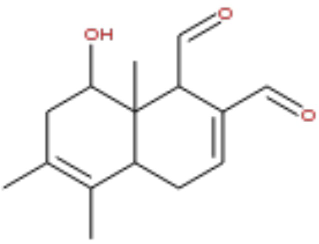

Cell ID	Cluster Center	Number of Compounds
193	0	5

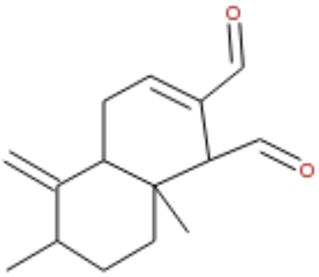

Cell ID	Cluster Center	Number of Compounds
193	0	5

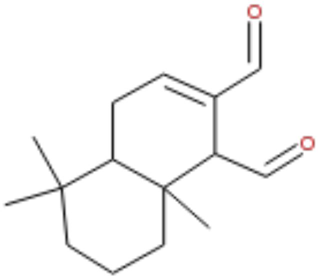

Cell ID	Cluster Center	Number of Compounds
193	0	5

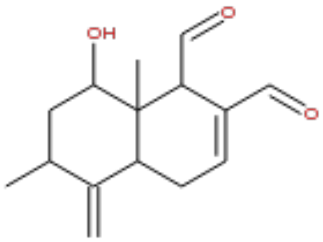

Cell ID	Cluster Center	Number of Compounds
193	0	5

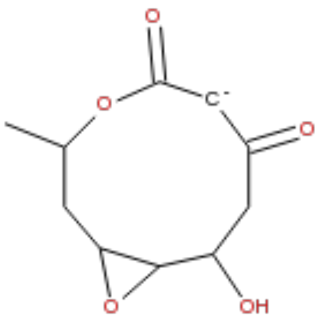

Cell ID	Cluster Center	Number of Compounds
194	1	2

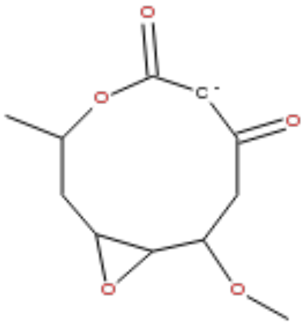

Cell ID	Cluster Center	Number of Compounds
194	0	2

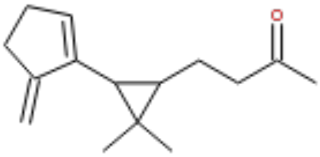

Cell ID	Cluster Center	Number of Compounds
195	1	8

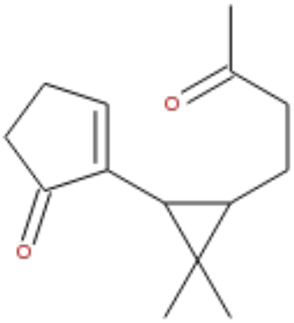

Cell ID	Cluster Center	Number of Compounds
195	0	8

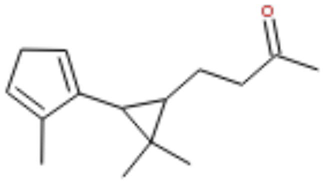

Cell ID	Cluster Center	Number of Compounds
195	0	8

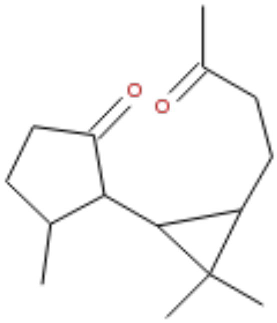

Cell ID	Cluster Center	Number of Compounds
195	0	8

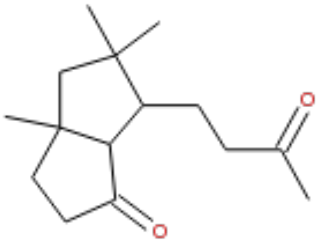

Cell ID	Cluster Center	Number of Compounds
195	0	8

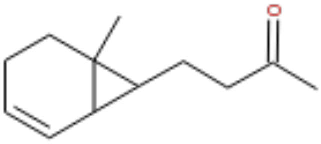

Cell ID	Cluster Center	Number of Compounds
195	0	8

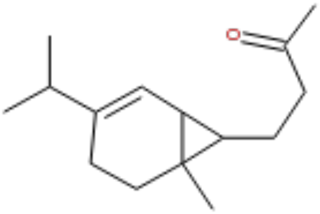

Cell ID	Cluster Center	Number of Compounds
195	0	8

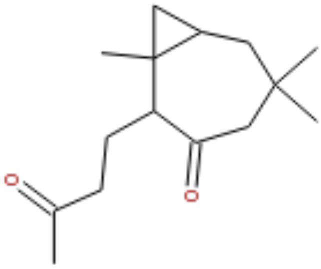

Cell ID	Cluster Center	Number of Compounds
195	0	8

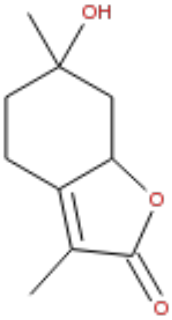

Cell ID	Cluster Center	Number of Compounds
197	1	1

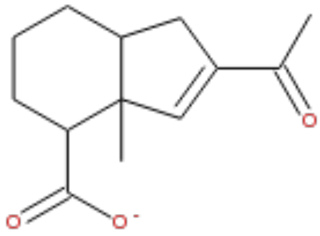

Cell ID	Cluster Center	Number of Compounds
200	1	5

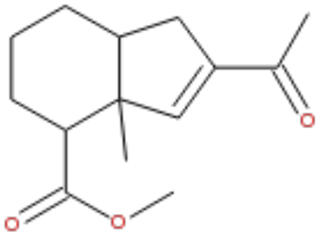

Cell ID	Cluster Center	Number of Compounds
200	0	5

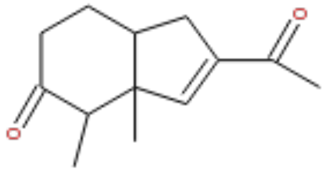

Cell ID	Cluster Center	Number of Compounds
200	0	5

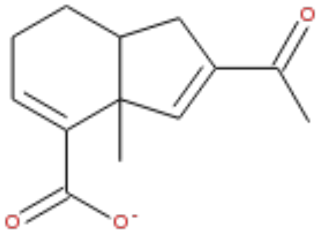

Cell ID	Cluster Center	Number of Compounds
200	0	5

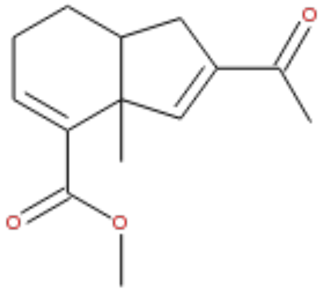

Cell ID	Cluster Center	Number of Compounds
200	0	5

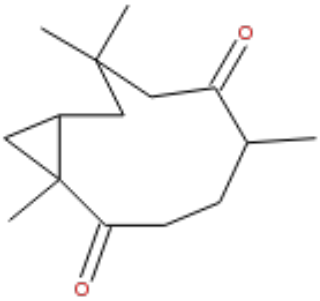

Cell ID	Cluster Center	Number of Compounds
204	1	2

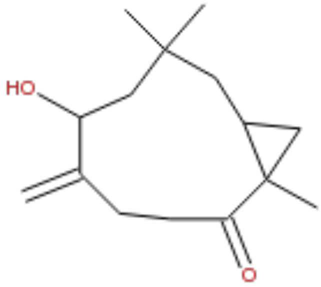

Cell ID	Cluster Center	Number of Compounds
204	0	2

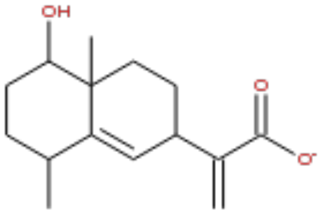

Cell ID	Cluster Center	Number of Compounds
205	1	4

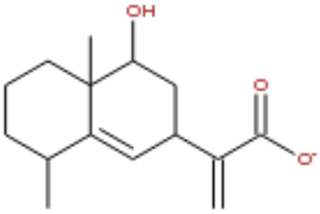

Cell ID	Cluster Center	Number of Compounds
205	0	4

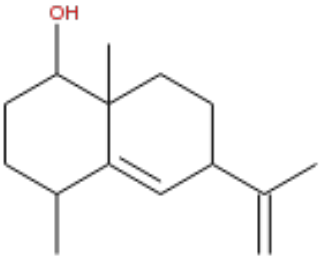

Cell ID	Cluster Center	Number of Compounds
205	0	4

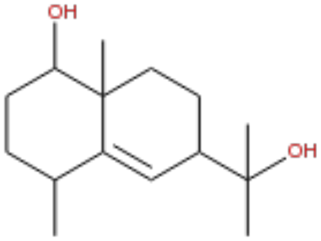

Cell ID	Cluster Center	Number of Compounds
205	0	4

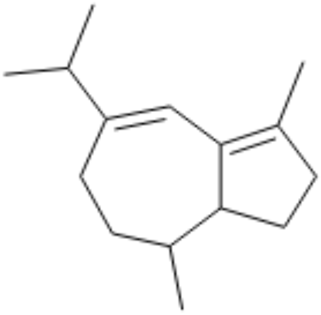

Cell ID	Cluster Center	Number of Compounds
206	1	36

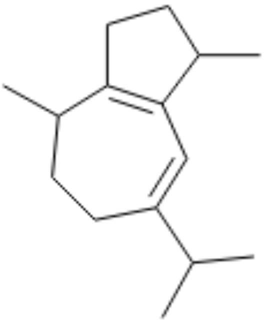

Cell ID	Cluster Center	Number of Compounds
206	0	36

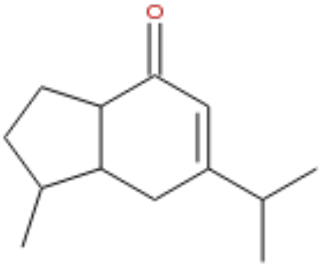

Cell ID	Cluster Center	Number of Compounds
206	0	36

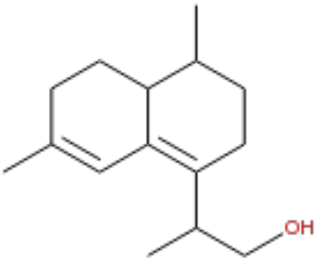

Cell ID	Cluster Center	Number of Compounds
206	0	36

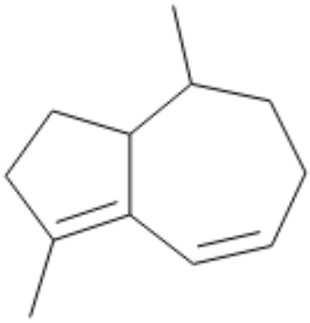

Cell ID	Cluster Center	Number of Compounds
206	0	36

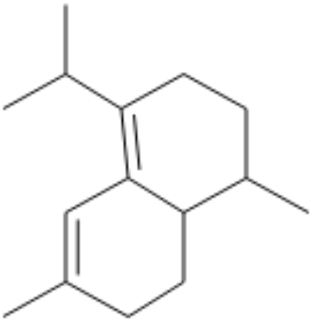

Cell ID	Cluster Center	Number of Compounds
206	0	36

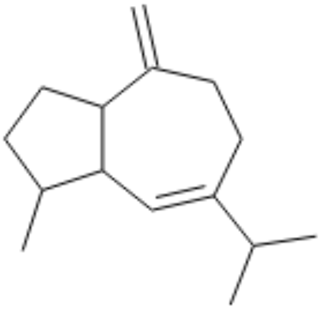

Cell ID	Cluster Center	Number of Compounds
206	0	36

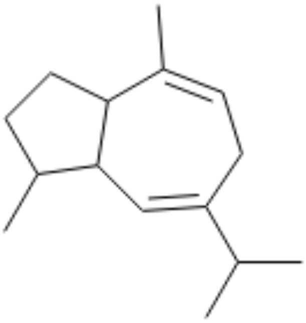

Cell ID	Cluster Center	Number of Compounds
206	0	36

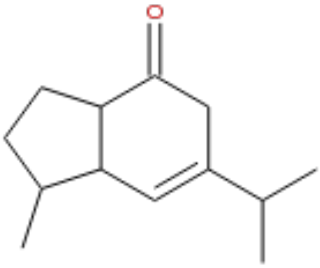

Cell ID	Cluster Center	Number of Compounds
206	0	36

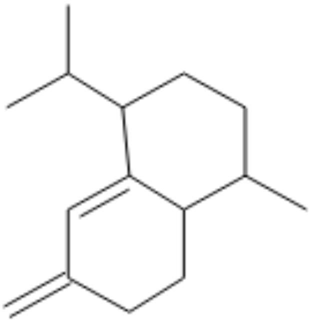

Cell ID	Cluster Center	Number of Compounds
206	0	36

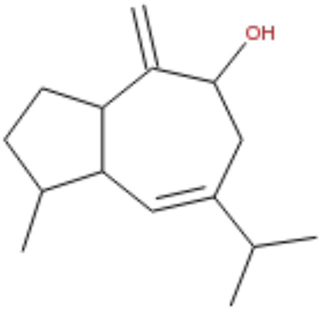

Cell ID	Cluster Center	Number of Compounds
206	0	36

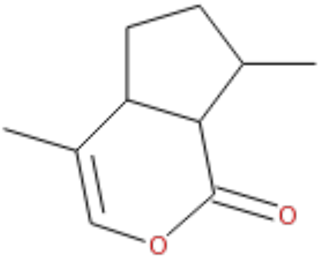

Cell ID	Cluster Center	Number of Compounds
206	0	36

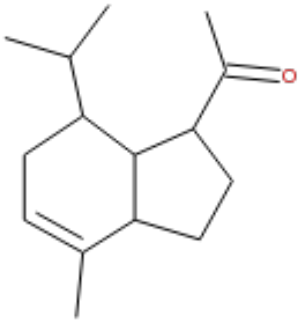

Cell ID	Cluster Center	Number of Compounds
206	0	36

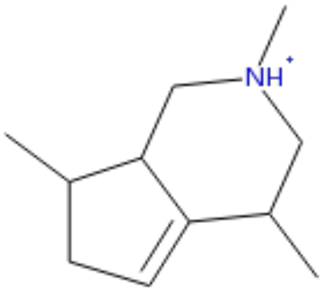

Cell ID	Cluster Center	Number of Compounds
206	0	36

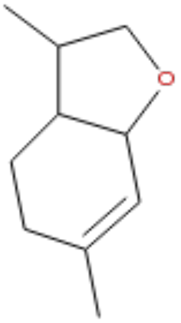

Cell ID	Cluster Center	Number of Compounds
206	0	36

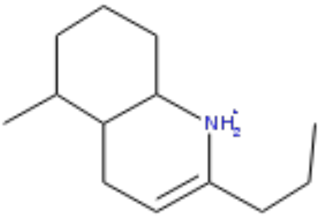

Cell ID	Cluster Center	Number of Compounds
206	0	36

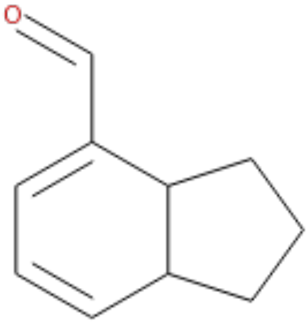

Cell ID	Cluster Center	Number of Compounds
206	0	36

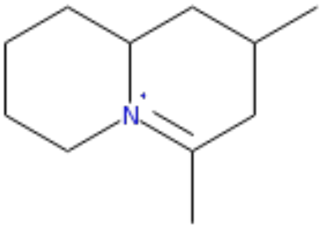

Cell ID	Cluster Center	Number of Compounds
206	0	36

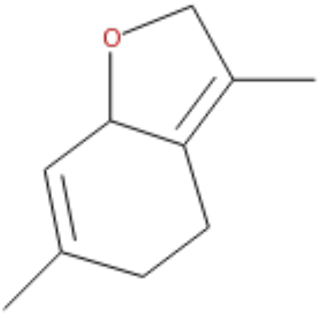

Cell ID	Cluster Center	Number of Compounds
206	0	36

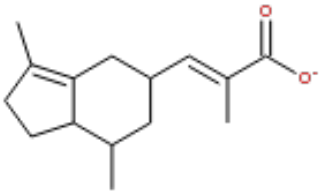

Cell ID	Cluster Center	Number of Compounds
206	0	36

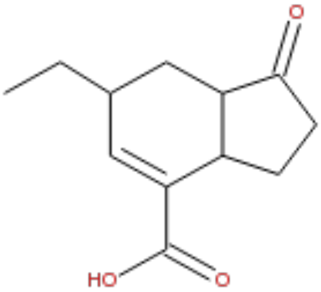

Cell ID	Cluster Center	Number of Compounds
206	0	36

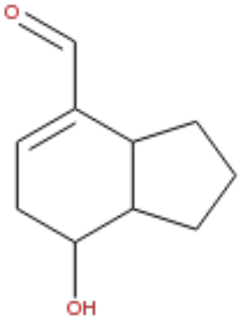

Cell ID	Cluster Center	Number of Compounds
206	0	36

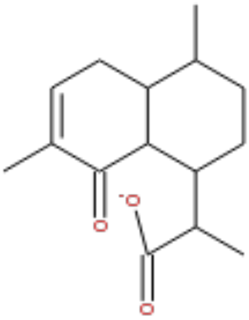

Cell ID	Cluster Center	Number of Compounds
206	0	36

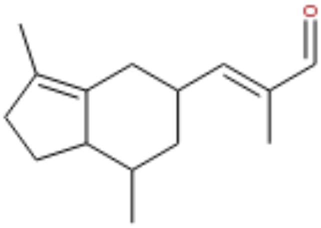

Cell ID	Cluster Center	Number of Compounds
206	0	36

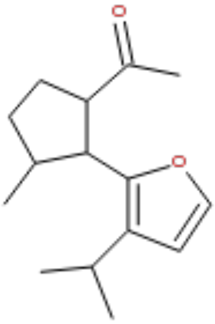

Cell ID	Cluster Center	Number of Compounds
206	0	36

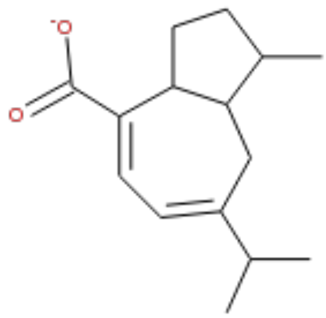

Cell ID	Cluster Center	Number of Compounds
206	0	36

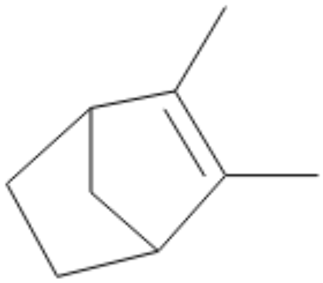

Cell ID	Cluster Center	Number of Compounds
206	0	36

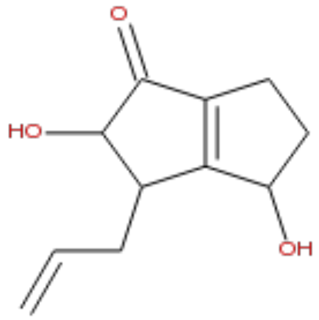

Cell ID	Cluster Center	Number of Compounds
206	0	36

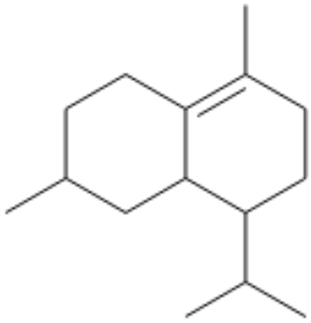

Cell ID	Cluster Center	Number of Compounds
206	0	36

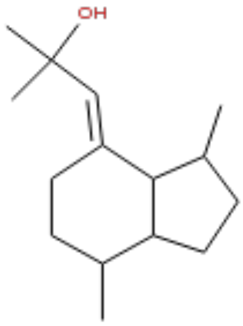

Cell ID	Cluster Center	Number of Compounds
206	0	36

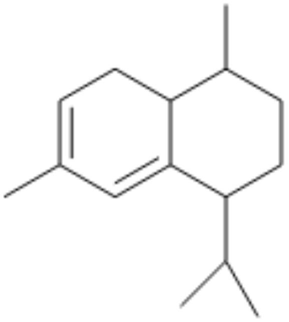

Cell ID	Cluster Center	Number of Compounds
206	0	36

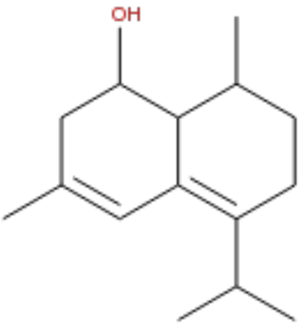

Cell ID	Cluster Center	Number of Compounds
206	0	36

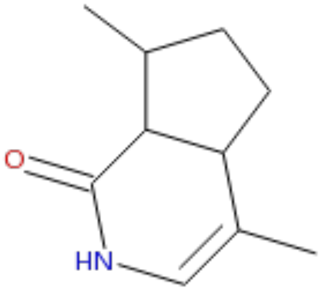

Cell ID	Cluster Center	Number of Compounds
206	0	36

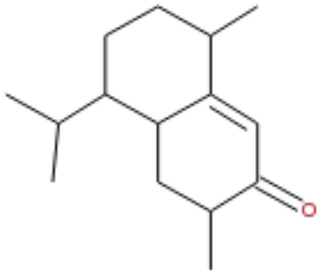

Cell ID	Cluster Center	Number of Compounds
206	0	36

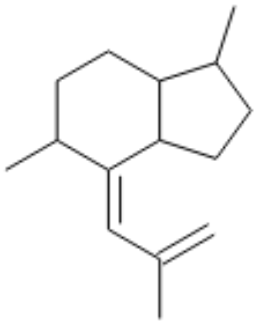

Cell ID	Cluster Center	Number of Compounds
206	0	36

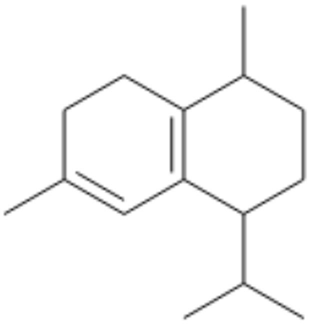

Cell ID	Cluster Center	Number of Compounds
206	0	36

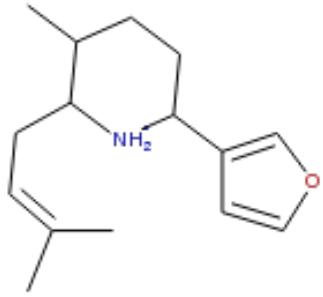

Cell ID	Cluster Center	Number of Compounds
208	1	2

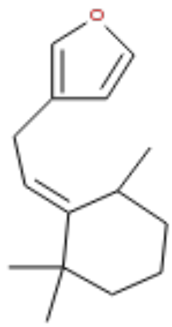

Cell ID	Cluster Center	Number of Compounds
208	0	2

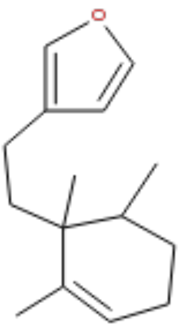

Cell ID	Cluster Center	Number of Compounds
209	1	6

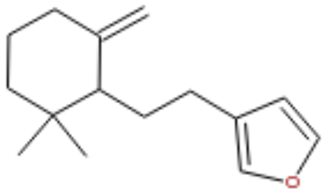

Cell ID	Cluster Center	Number of Compounds
209	0	6

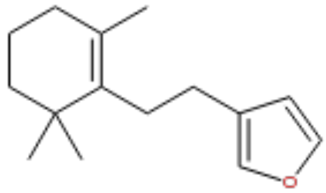

Cell ID	Cluster Center	Number of Compounds
209	0	6

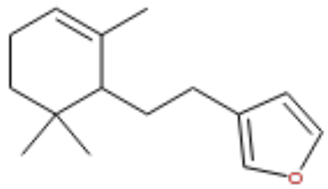

Cell ID	Cluster Center	Number of Compounds
209	0	6

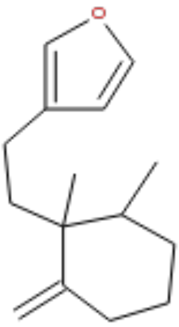

Cell ID	Cluster Center	Number of Compounds
209	0	6

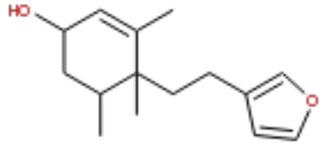

Cell ID	Cluster Center	Number of Compounds
209	0	6

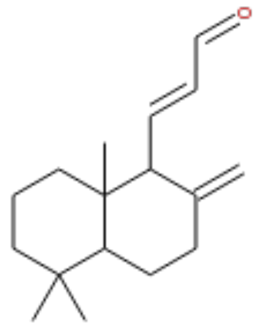

Cell ID	Cluster Center	Number of Compounds
211	1	1

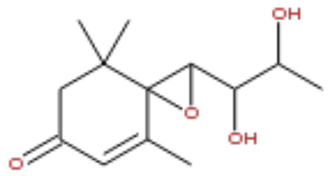

Cell ID	Cluster Center	Number of Compounds
212	1	3

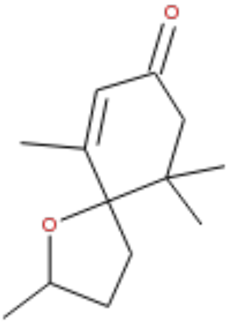

Cell ID	Cluster Center	Number of Compounds
212	0	3

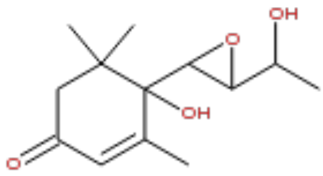

Cell ID	Cluster Center	Number of Compounds
212	0	3

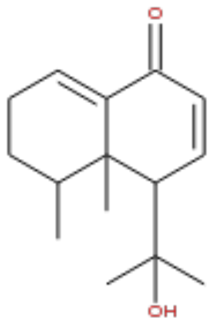

Cell ID	Cluster Center	Number of Compounds
215	1	3

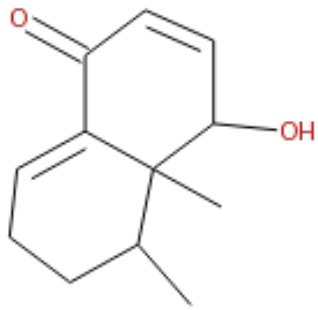

Cell ID	Cluster Center	Number of Compounds
215	0	3

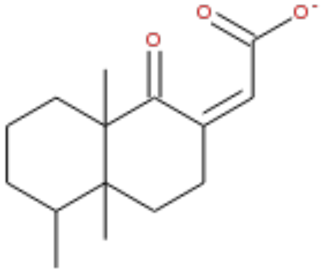

Cell ID	Cluster Center	Number of Compounds
215	0	3

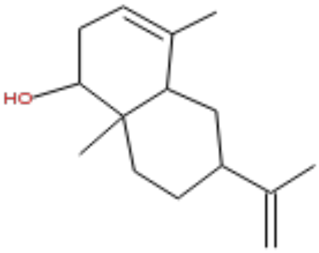

Cell ID	Cluster Center	Number of Compounds
216	1	9

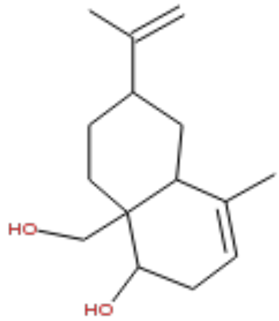

Cell ID	Cluster Center	Number of Compounds
216	0	9

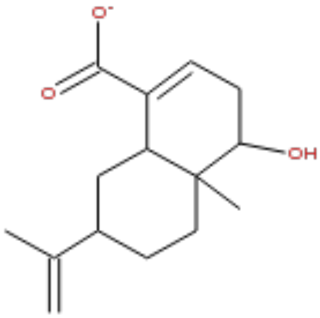

Cell ID	Cluster Center	Number of Compounds
216	0	9

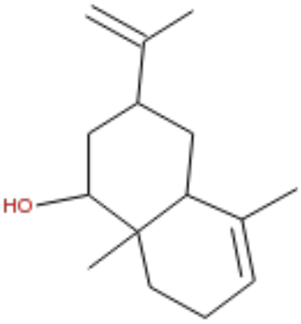

Cell ID	Cluster Center	Number of Compounds
216	0	9

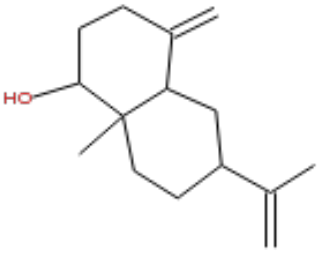

Cell ID	Cluster Center	Number of Compounds
216	0	9

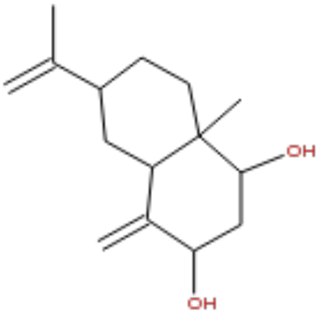

Cell ID	Cluster Center	Number of Compounds
216	0	9

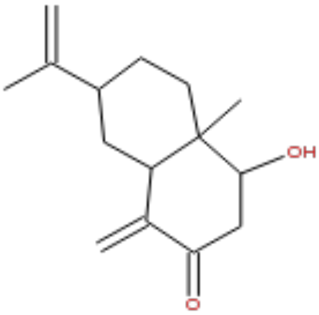

Cell ID	Cluster Center	Number of Compounds
216	0	9

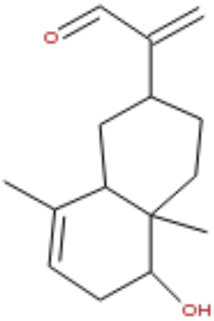

Cell ID	Cluster Center	Number of Compounds
216	0	9

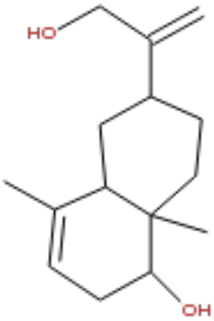

Cell ID	Cluster Center	Number of Compounds
216	0	9

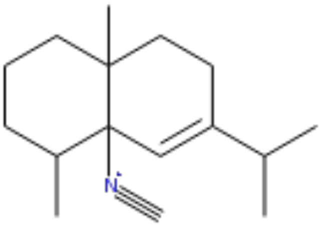

Cell ID	Cluster Center	Number of Compounds
217	1	2

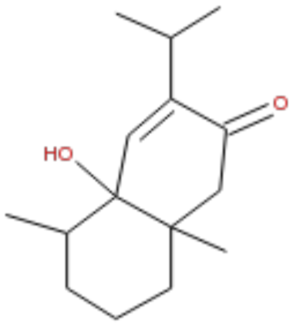

Cell ID	Cluster Center	Number of Compounds
217	0	2

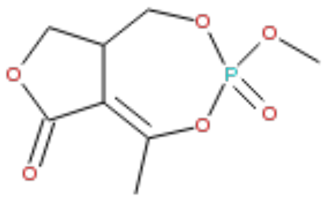

Cell ID	Cluster Center	Number of Compounds
218	1	6

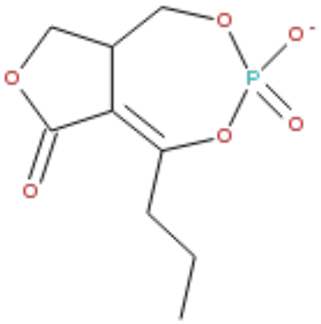

Cell ID	Cluster Center	Number of Compounds
218	0	6

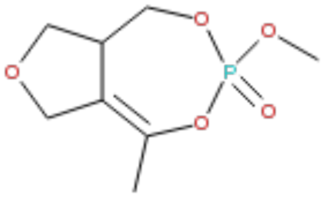

Cell ID	Cluster Center	Number of Compounds
218	0	6

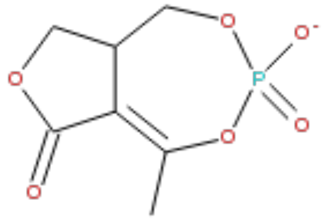

Cell ID	Cluster Center	Number of Compounds
218	0	6

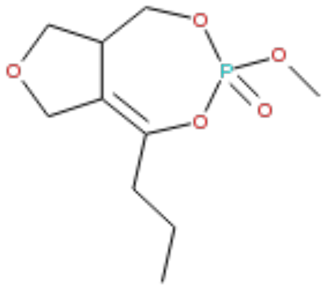

Cell ID	Cluster Center	Number of Compounds
218	0	6

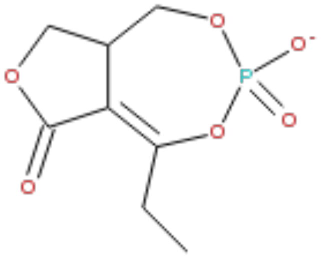

Cell ID	Cluster Center	Number of Compounds
218	0	6

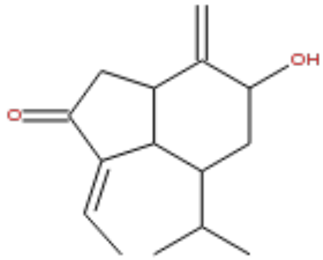

Cell ID	Cluster Center	Number of Compounds
219	1	3

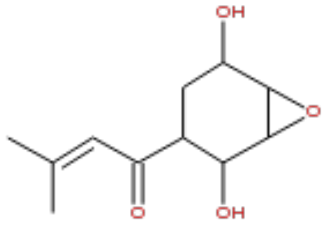

Cell ID	Cluster Center	Number of Compounds
219	0	3

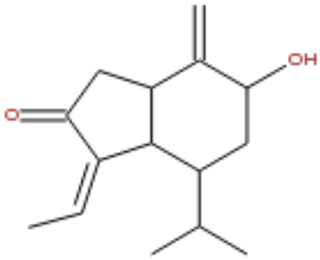

Cell ID	Cluster Center	Number of Compounds
219	0	3

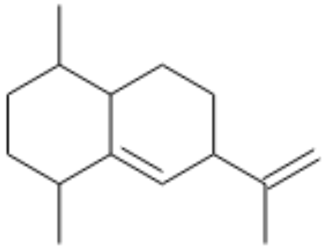

Cell ID	Cluster Center	Number of Compounds
220	1	6

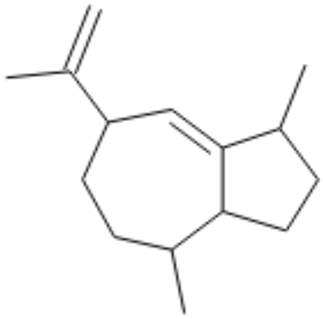

Cell ID	Cluster Center	Number of Compounds
220	0	6

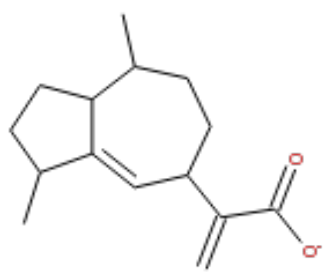

Cell ID	Cluster Center	Number of Compounds
220	0	6

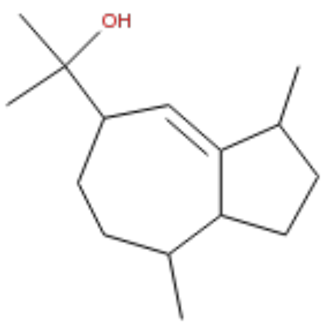

Cell ID	Cluster Center	Number of Compounds
220	0	6

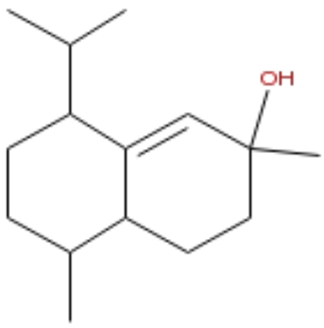

Cell ID	Cluster Center	Number of Compounds
220	0	6

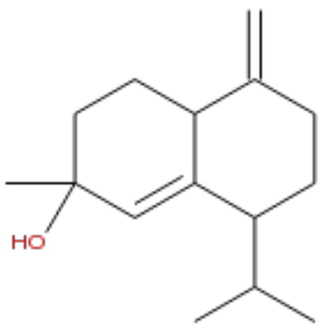

Cell ID	Cluster Center	Number of Compounds
220	0	6

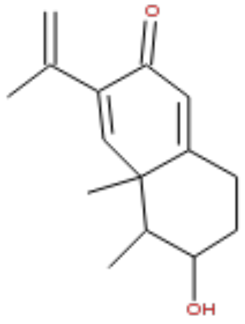

Cell ID	Cluster Center	Number of Compounds
221	1	4

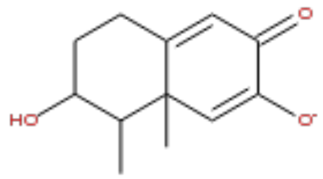

Cell ID	Cluster Center	Number of Compounds
221	0	4

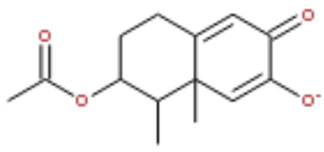

Cell ID	Cluster Center	Number of Compounds
221	0	4

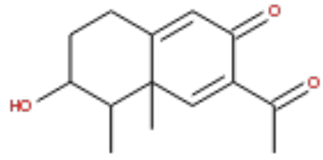

Cell ID	Cluster Center	Number of Compounds
221	0	4

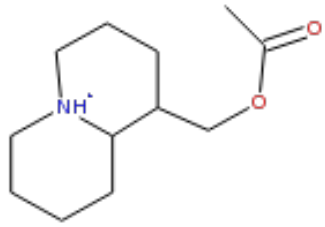

Cell ID	Cluster Center	Number of Compounds
222	1	5

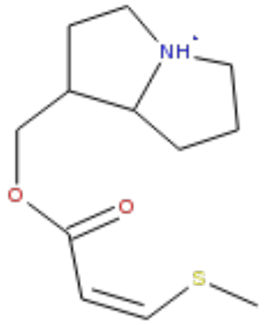

Cell ID	Cluster Center	Number of Compounds
222	0	5

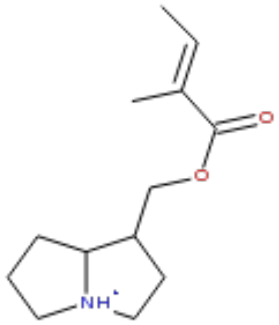

Cell ID	Cluster Center	Number of Compounds
222	0	5

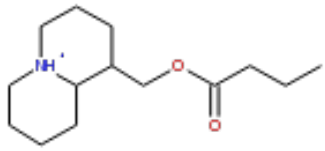

Cell ID	Cluster Center	Number of Compounds
222	0	5

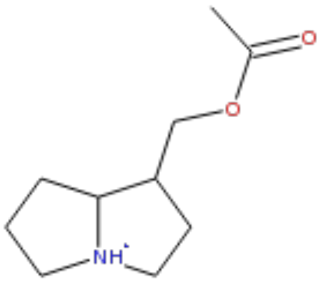

Cell ID	Cluster Center	Number of Compounds
222	0	5

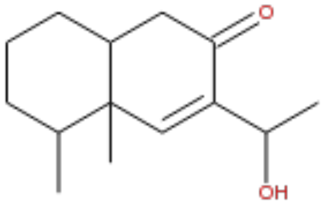

Cell ID	Cluster Center	Number of Compounds
225	1	7

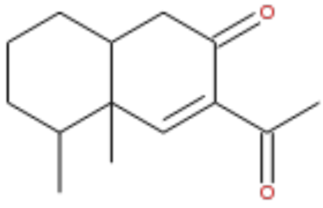

Cell ID	Cluster Center	Number of Compounds
225	0	7

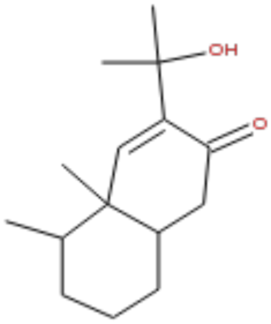

Cell ID	Cluster Center	Number of Compounds
225	0	7

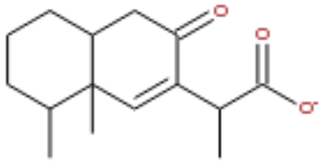

Cell ID	Cluster Center	Number of Compounds
225	0	7

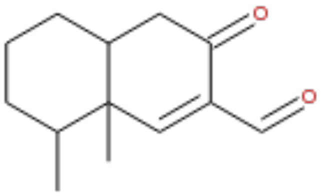

Cell ID	Cluster Center	Number of Compounds
225	0	7

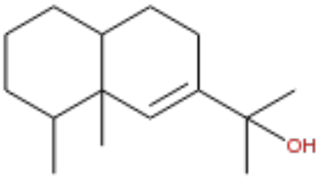

Cell ID	Cluster Center	Number of Compounds
225	0	7

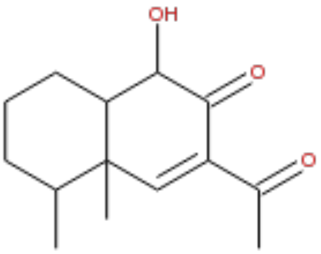

Cell ID	Cluster Center	Number of Compounds
225	0	7

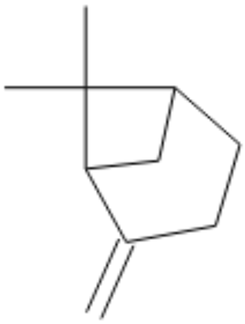

Cell ID	Cluster Center	Number of Compounds
227	1	24

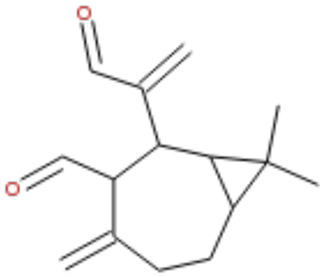

Cell ID	Cluster Center	Number of Compounds
227	0	24

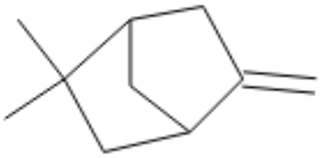

Cell ID	Cluster Center	Number of Compounds
227	0	24

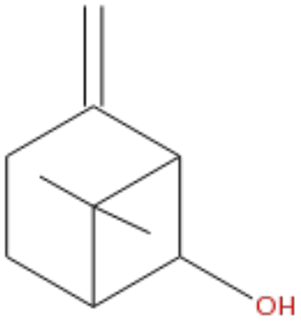

Cell ID	Cluster Center	Number of Compounds
227	0	24

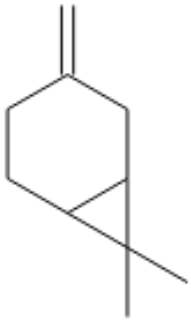

Cell ID	Cluster Center	Number of Compounds
227	0	24

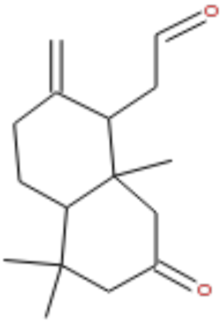

Cell ID	Cluster Center	Number of Compounds
227	0	24

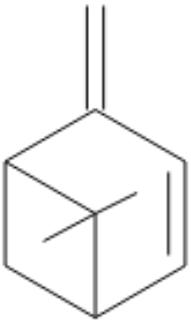

Cell ID	Cluster Center	Number of Compounds
227	0	24

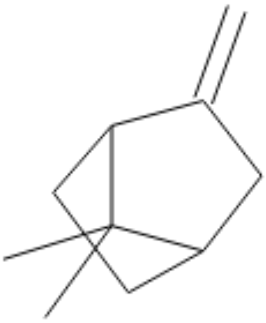

Cell ID	Cluster Center	Number of Compounds
227	0	24

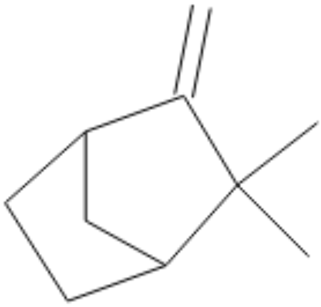

Cell ID	Cluster Center	Number of Compounds
227	0	24

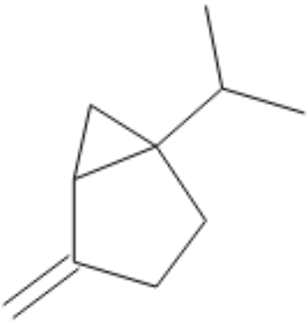

Cell ID	Cluster Center	Number of Compounds
227	0	24

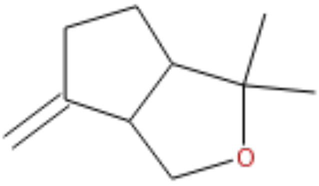

Cell ID	Cluster Center	Number of Compounds
227	0	24

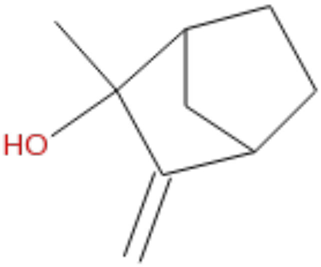

Cell ID	Cluster Center	Number of Compounds
227	0	24

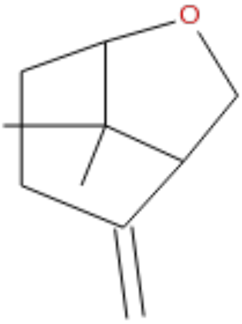

Cell ID	Cluster Center	Number of Compounds
227	0	24

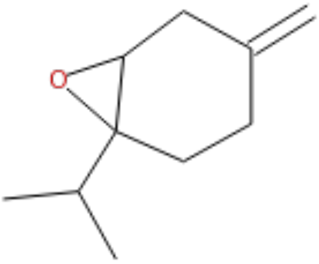

Cell ID	Cluster Center	Number of Compounds
227	0	24

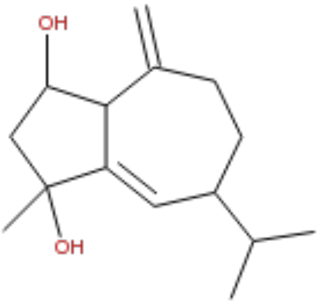

Cell ID	Cluster Center	Number of Compounds
227	0	24

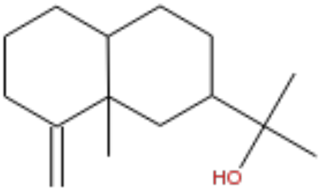

Cell ID	Cluster Center	Number of Compounds
227	0	24

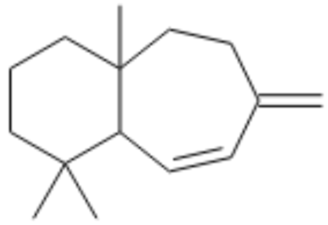

Cell ID	Cluster Center	Number of Compounds
227	0	24

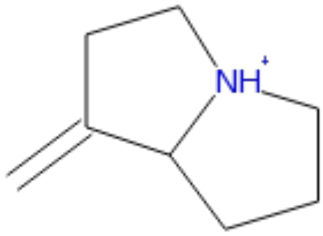

Cell ID	Cluster Center	Number of Compounds
227	0	24

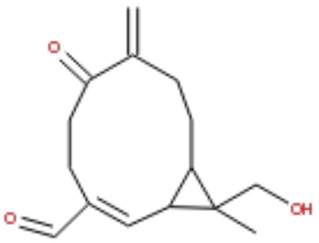

Cell ID	Cluster Center	Number of Compounds
227	0	24

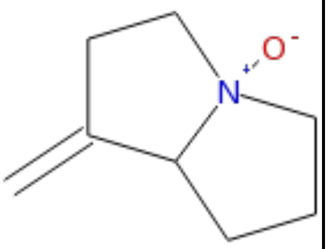

Cell ID	Cluster Center	Number of Compounds
227	0	24

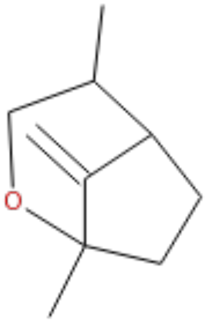

Cell ID	Cluster Center	Number of Compounds
227	0	24

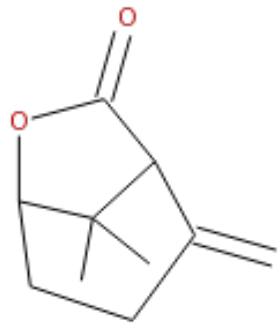

Cell ID	Cluster Center	Number of Compounds
227	0	24

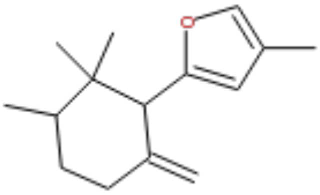

Cell ID	Cluster Center	Number of Compounds
227	0	24

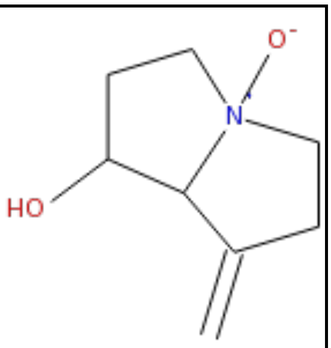

Cell ID	Cluster Center	Number of Compounds
227	0	24

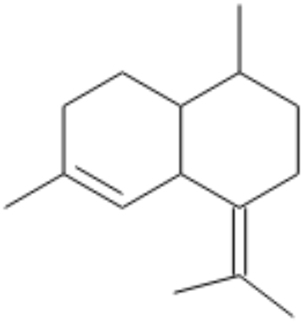

Cell ID	Cluster Center	Number of Compounds
228	1	7

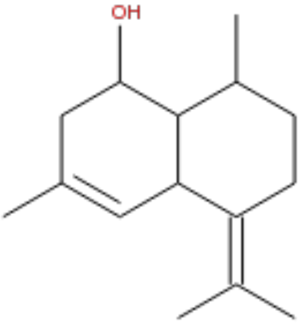

Cell ID	Cluster Center	Number of Compounds
228	0	7

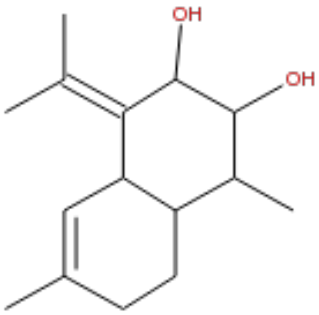

Cell ID	Cluster Center	Number of Compounds
228	0	7

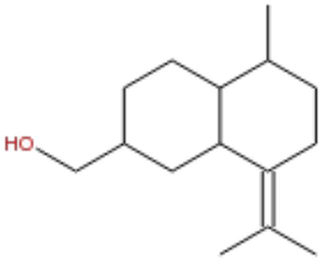

Cell ID	Cluster Center	Number of Compounds
228	0	7

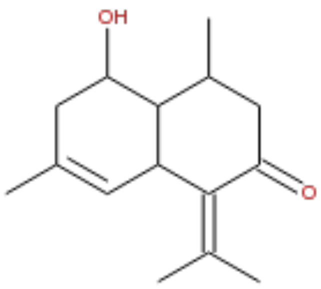

Cell ID	Cluster Center	Number of Compounds
228	0	7

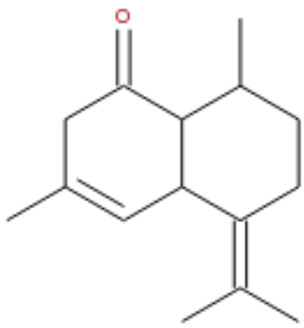

Cell ID	Cluster Center	Number of Compounds
228	0	7

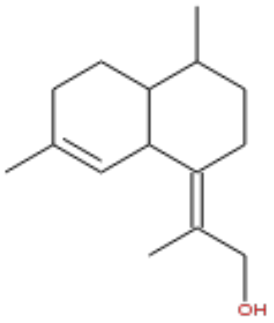

Cell ID	Cluster Center	Number of Compounds
228	0	7

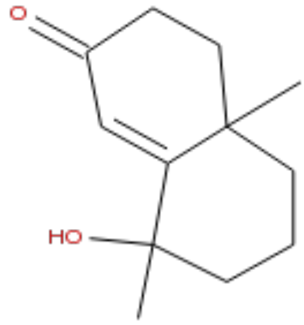

Cell ID	Cluster Center	Number of Compounds
229	1	17

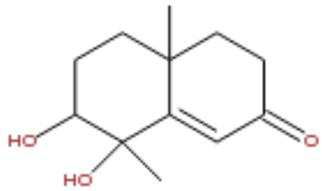

Cell ID	Cluster Center	Number of Compounds
229	0	17

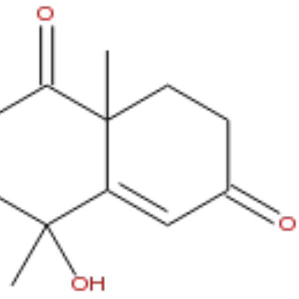

Cell ID	Cluster Center	Number of Compounds
229	0	17

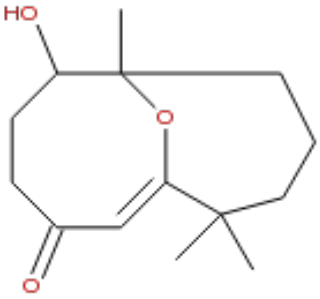

Cell ID	Cluster Center	Number of Compounds
229	0	17

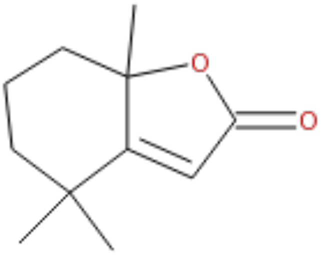

Cell ID	Cluster Center	Number of Compounds
229	0	17

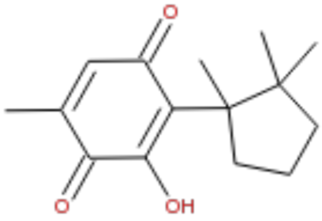

Cell ID	Cluster Center	Number of Compounds
229	0	17

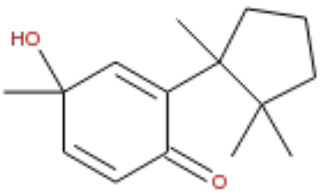

Cell ID	Cluster Center	Number of Compounds
229	0	17

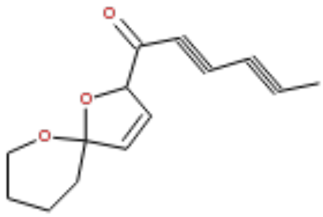

Cell ID	Cluster Center	Number of Compounds
229	0	17

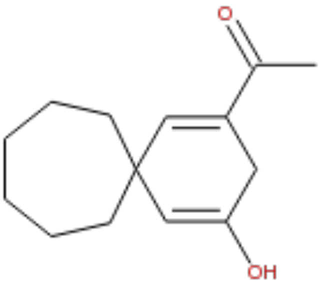

Cell ID	Cluster Center	Number of Compounds
229	0	17

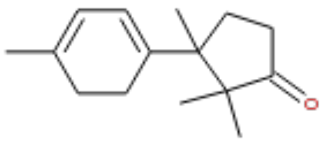

Cell ID	Cluster Center	Number of Compounds
229	0	17

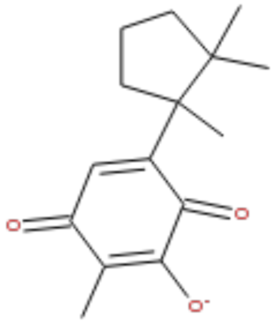

Cell ID	Cluster Center	Number of Compounds
229	0	17

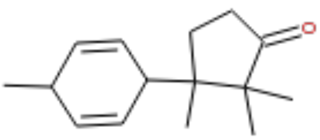

Cell ID	Cluster Center	Number of Compounds
229	0	17

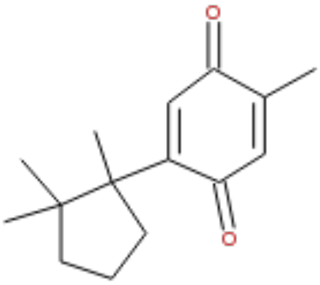

Cell ID	Cluster Center	Number of Compounds
229	0	17

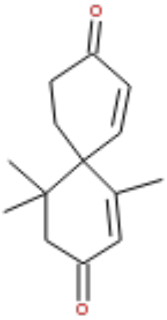

Cell ID	Cluster Center	Number of Compounds
229	0	17

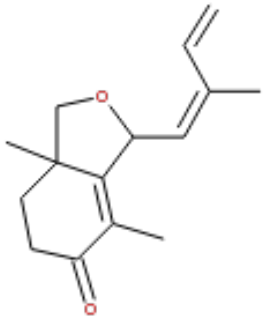

Cell ID	Cluster Center	Number of Compounds
229	0	17

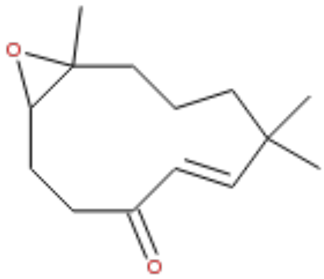

Cell ID	Cluster Center	Number of Compounds
229	0	17

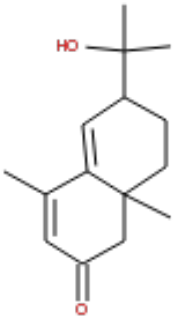

Cell ID	Cluster Center	Number of Compounds
229	0	17

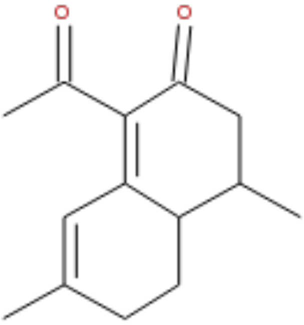

Cell ID	Cluster Center	Number of Compounds
231	1	3

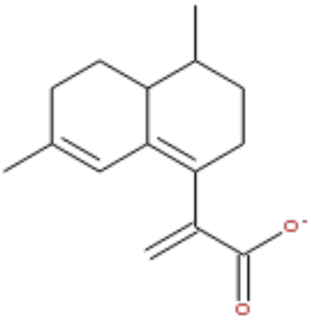

Cell ID	Cluster Center	Number of Compounds
231	0	3

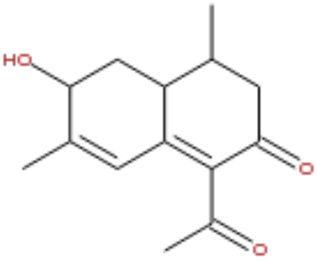

Cell ID	Cluster Center	Number of Compounds
231	0	3

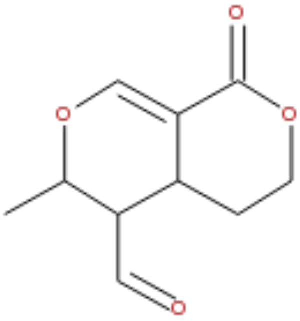

Cell ID	Cluster Center	Number of Compounds
232	1	4

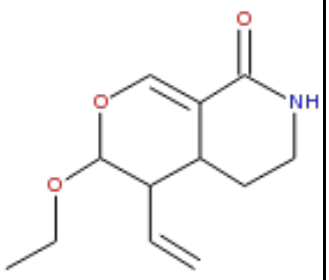

Cell ID	Cluster Center	Number of Compounds
232	0	4

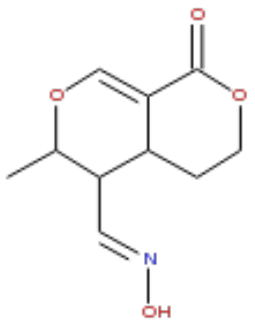

Cell ID	Cluster Center	Number of Compounds
232	0	4

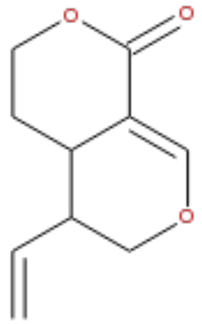

Cell ID	Cluster Center	Number of Compounds
232	0	4

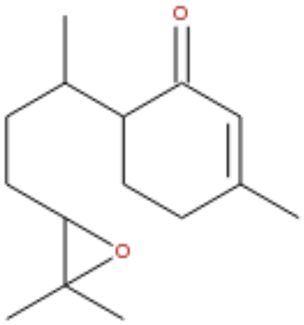

Cell ID	Cluster Center	Number of Compounds
233	1	2

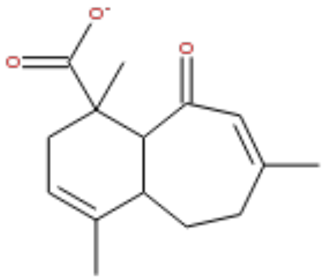

Cell ID	Cluster Center	Number of Compounds
233	0	2

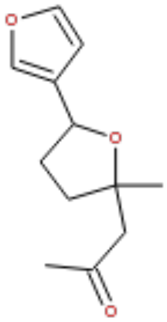

Cell ID	Cluster Center	Number of Compounds
235	1	1

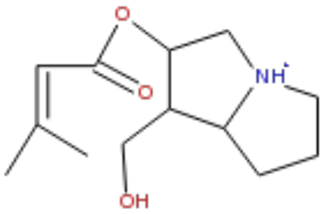

Cell ID	Cluster Center	Number of Compounds
236	1	3

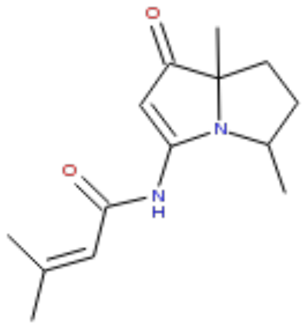

Cell ID	Cluster Center	Number of Compounds
236	0	3

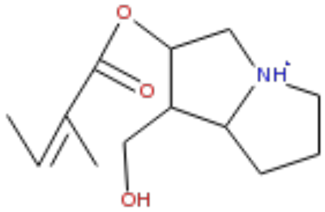

Cell ID	Cluster Center	Number of Compounds
236	0	3

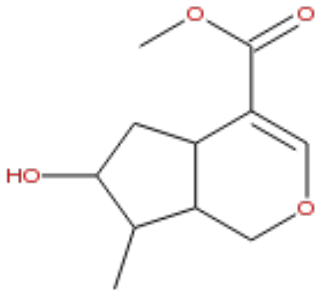

Cell ID	Cluster Center	Number of Compounds
242	1	4

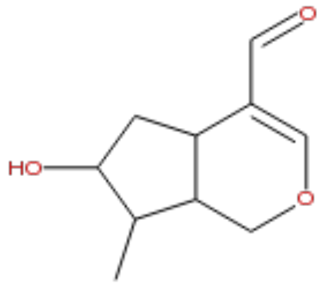

Cell ID	Cluster Center	Number of Compounds
242	0	4

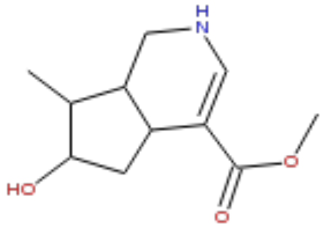

Cell ID	Cluster Center	Number of Compounds
242	0	4

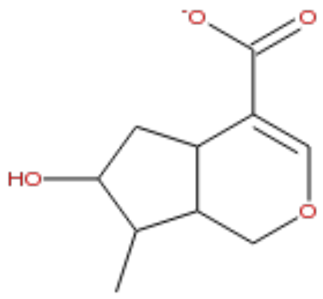

Cell ID	Cluster Center	Number of Compounds
242	0	4

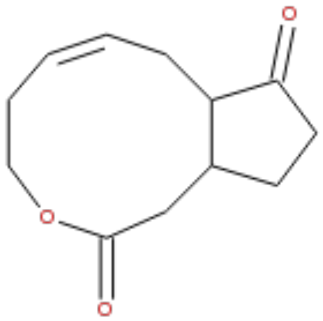

Cell ID	Cluster Center	Number of Compounds
243	1	1

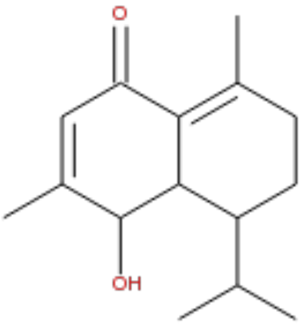

Cell ID	Cluster Center	Number of Compounds
244	1	1

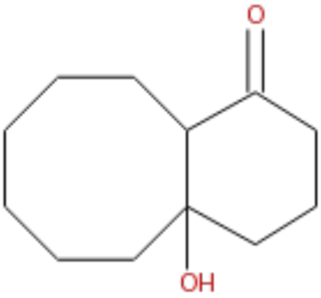

Cell ID	Cluster Center	Number of Compounds
245	1	1

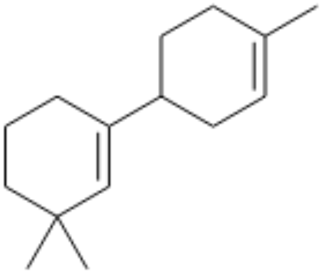

Cell ID	Cluster Center	Number of Compounds
246	1	2

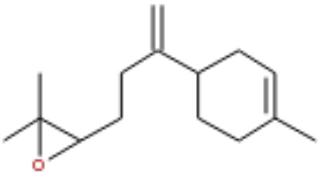

Cell ID	Cluster Center	Number of Compounds
246	0	2

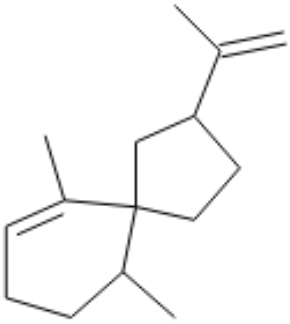

Cell ID	Cluster Center	Number of Compounds
247	1	6

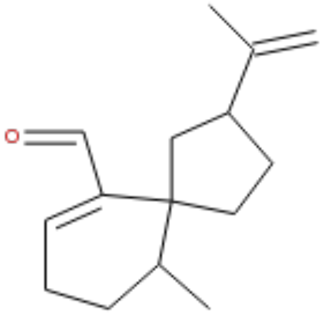

Cell ID	Cluster Center	Number of Compounds
247	0	6

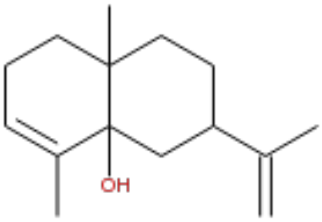

Cell ID	Cluster Center	Number of Compounds
247	0	6

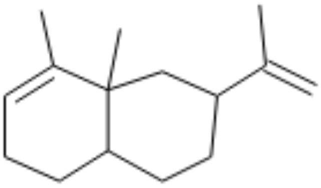

Cell ID	Cluster Center	Number of Compounds
247	0	6

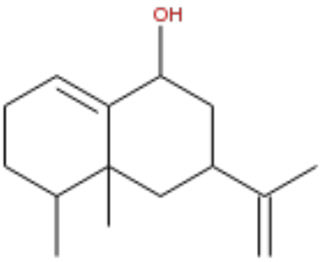

Cell ID	Cluster Center	Number of Compounds
247	0	6

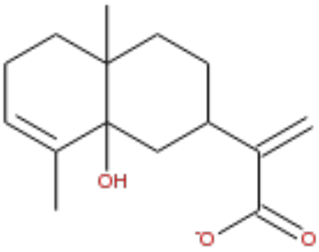

Cell ID	Cluster Center	Number of Compounds
247	0	6

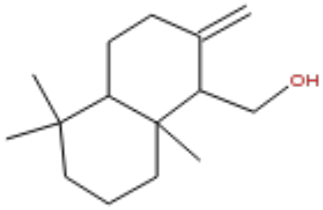

Cell ID	Cluster Center	Number of Compounds
248	1	3

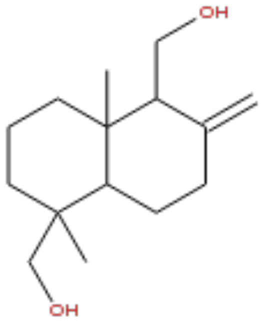

Cell ID	Cluster Center	Number of Compounds
248	0	3

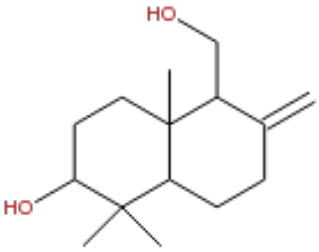

Cell ID	Cluster Center	Number of Compounds
248	0	3

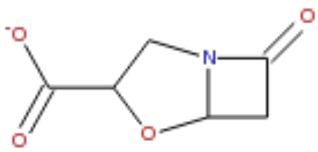

Cell ID	Cluster Center	Number of Compounds
249	1	24

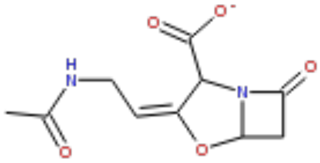

Cell ID	Cluster Center	Number of Compounds
249	0	24

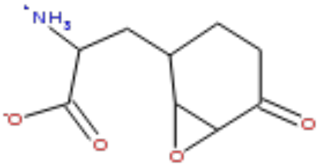

Cell ID	Cluster Center	Number of Compounds
249	0	24

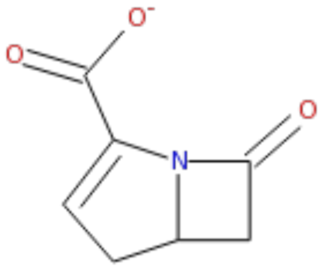

Cell ID	Cluster Center	Number of Compounds
249	0	24

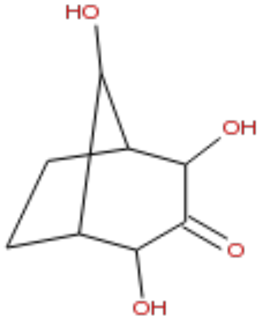

Cell ID	Cluster Center	Number of Compounds
249	0	24

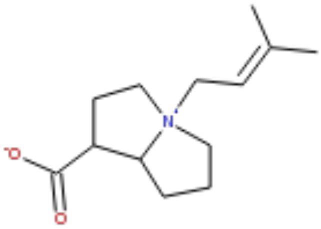

Cell ID	Cluster Center	Number of Compounds
249	0	24

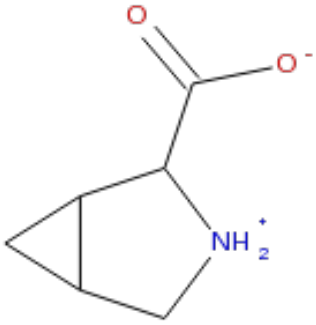

Cell ID	Cluster Center	Number of Compounds
249	0	24

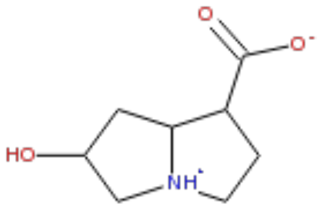

Cell ID	Cluster Center	Number of Compounds
249	0	24

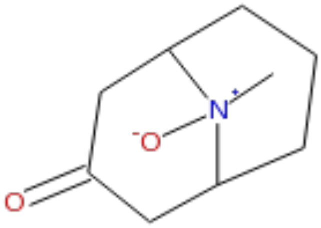

Cell ID	Cluster Center	Number of Compounds
249	0	24

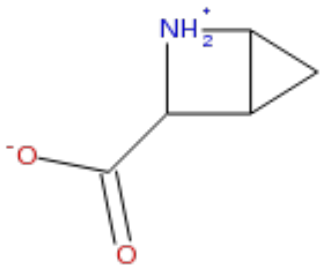

Cell ID	Cluster Center	Number of Compounds
249	0	24

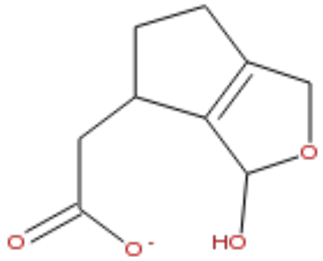

Cell ID	Cluster Center	Number of Compounds
249	0	24

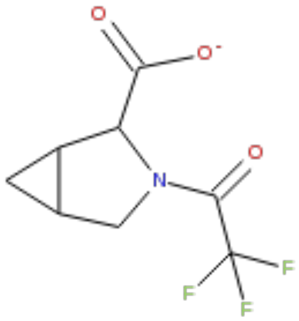

Cell ID	Cluster Center	Number of Compounds
249	0	24

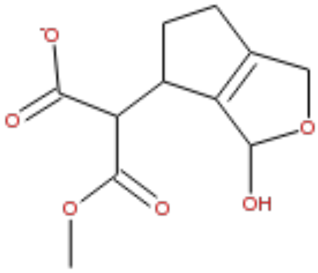

Cell ID	Cluster Center	Number of Compounds
249	0	24

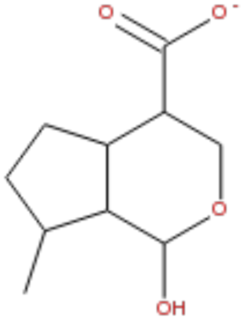

Cell ID	Cluster Center	Number of Compounds
249	0	24

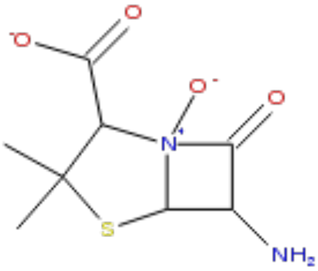

Cell ID	Cluster Center	Number of Compounds
249	0	24

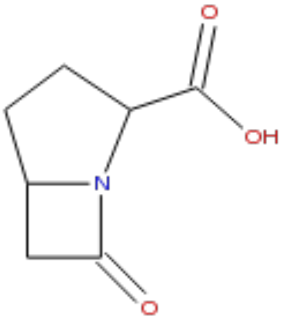

Cell ID	Cluster Center	Number of Compounds
249	0	24

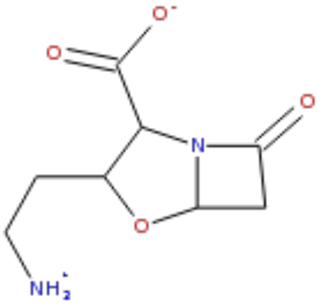

Cell ID	Cluster Center	Number of Compounds
249	0	24

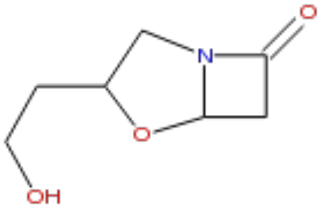

Cell ID	Cluster Center	Number of Compounds
249	0	24

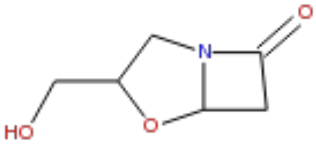

Cell ID	Cluster Center	Number of Compounds
249	0	24

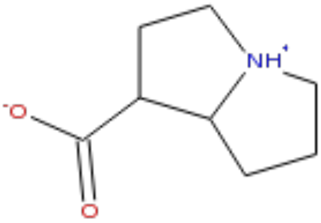

Cell ID	Cluster Center	Number of Compounds
249	0	24

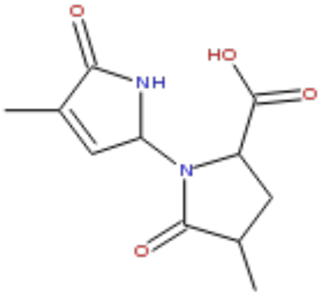

Cell ID	Cluster Center	Number of Compounds
249	0	24

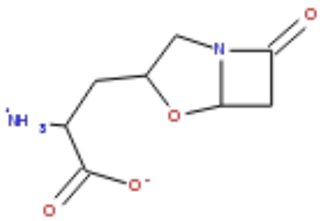

Cell ID	Cluster Center	Number of Compounds
249	0	24

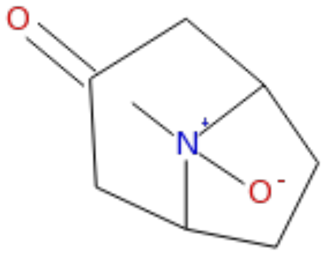

Cell ID	Cluster Center	Number of Compounds
249	0	24

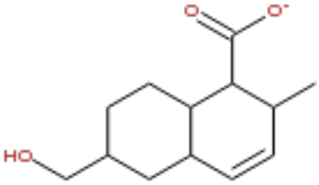

Cell ID	Cluster Center	Number of Compounds
249	0	24

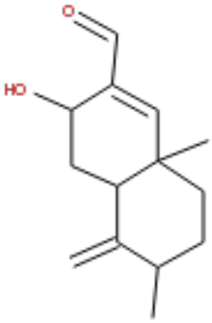

Cell ID	Cluster Center	Number of Compounds
250	1	2

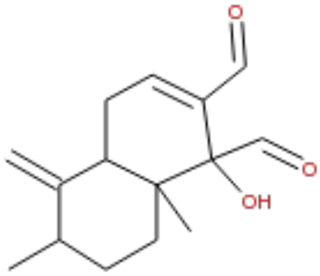

Cell ID	Cluster Center	Number of Compounds
250	0	2

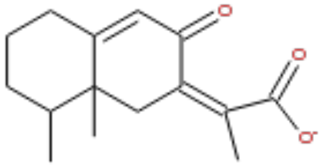

Cell ID	Cluster Center	Number of Compounds
254	1	4

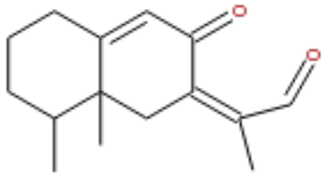

Cell ID	Cluster Center	Number of Compounds
254	0	4

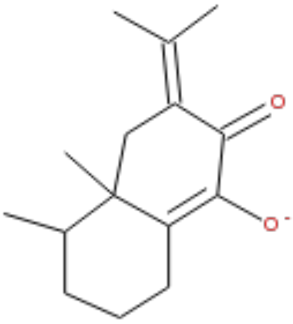

Cell ID	Cluster Center	Number of Compounds
254	0	4

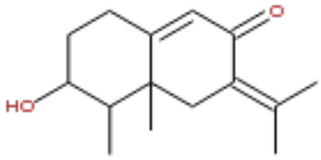

Cell ID	Cluster Center	Number of Compounds
254	0	4

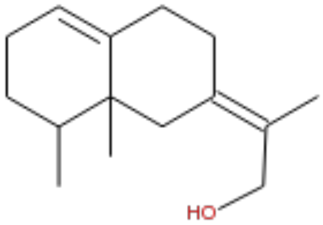

Cell ID	Cluster Center	Number of Compounds
256	1	3

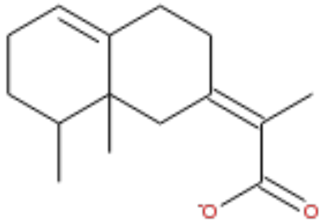

Cell ID	Cluster Center	Number of Compounds
256	0	3

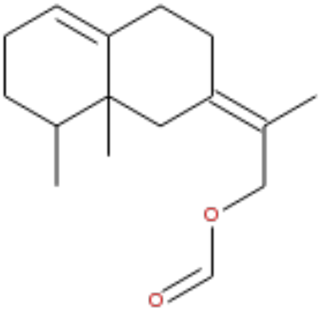

Cell ID	Cluster Center	Number of Compounds
256	0	3

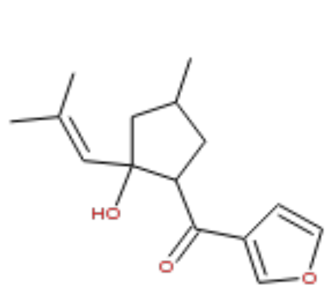

Cell ID	Cluster Center	Number of Compounds
258	1	3

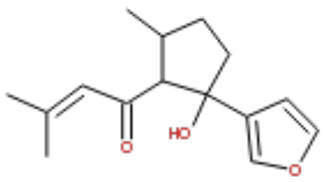

Cell ID	Cluster Center	Number of Compounds
258	0	3

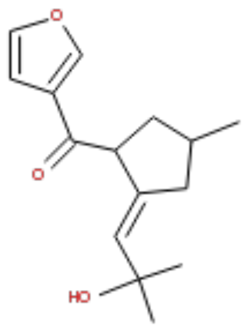

Cell ID	Cluster Center	Number of Compounds
258	0	3

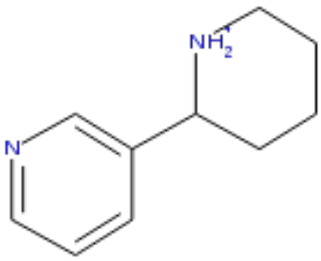

Cell ID	Cluster Center	Number of Compounds
259	1	5

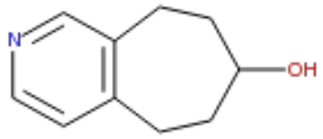

Cell ID	Cluster Center	Number of Compounds
259	0	5

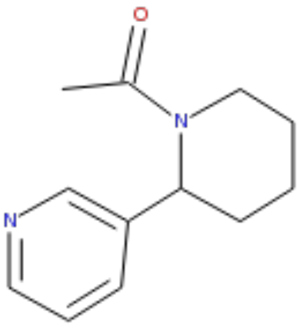

Cell ID	Cluster Center	Number of Compounds
259	0	5

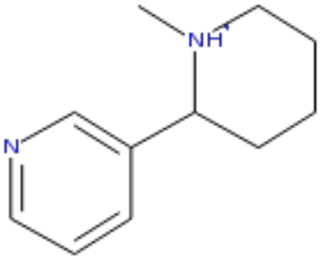

Cell ID	Cluster Center	Number of Compounds
259	0	5

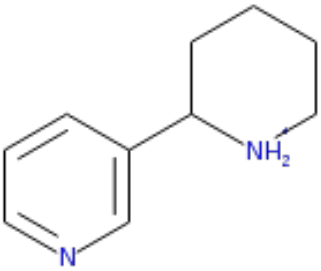

Cell ID	Cluster Center	Number of Compounds
259	0	5

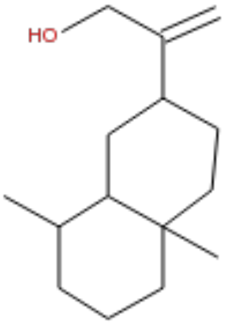

Cell ID	Cluster Center	Number of Compounds
260	1	6

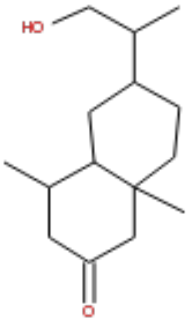

Cell ID	Cluster Center	Number of Compounds
260	0	6

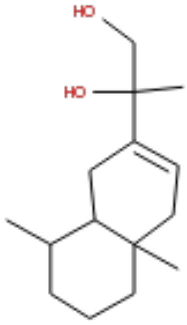

Cell ID	Cluster Center	Number of Compounds
260	0	6

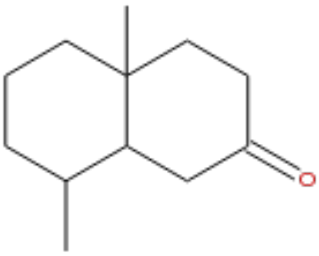

Cell ID	Cluster Center	Number of Compounds
260	0	6

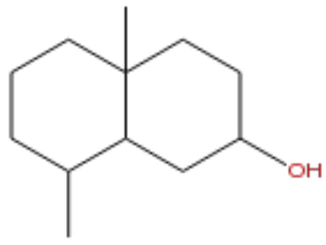

Cell ID	Cluster Center	Number of Compounds
260	0	6

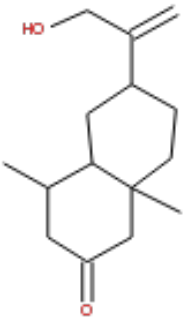

Cell ID	Cluster Center	Number of Compounds
260	0	6

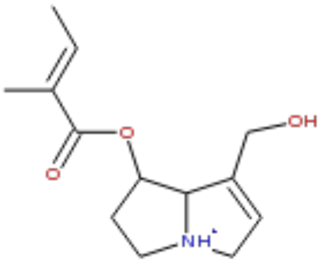

Cell ID	Cluster Center	Number of Compounds
261	1	6

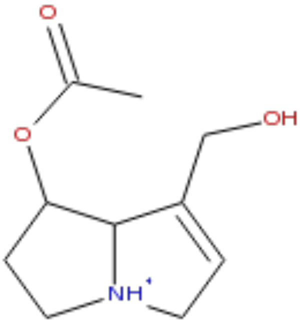

Cell ID	Cluster Center	Number of Compounds
261	0	6

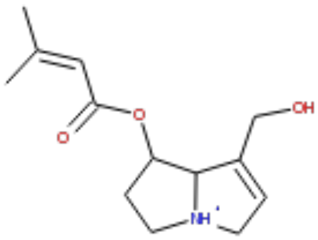

Cell ID	Cluster Center	Number of Compounds
261	0	6

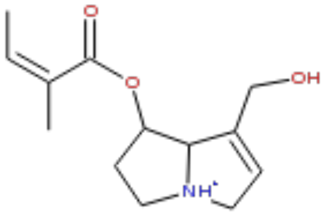

Cell ID	Cluster Center	Number of Compounds
261	0	6

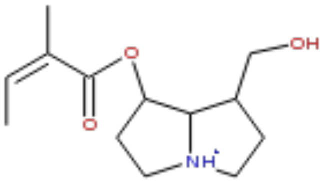

Cell ID	Cluster Center	Number of Compounds
261	0	6

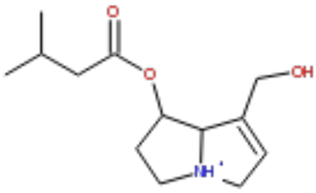

Cell ID	Cluster Center	Number of Compounds
261	0	6

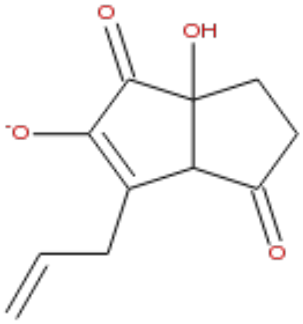

Cell ID	Cluster Center	Number of Compounds
265	1	5

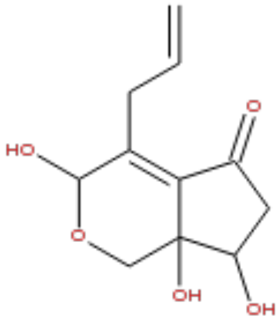

Cell ID	Cluster Center	Number of Compounds
265	0	5

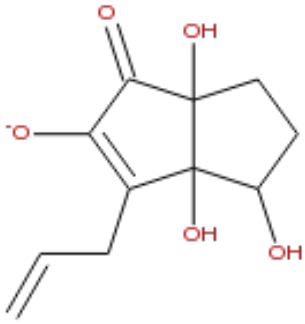

Cell ID	Cluster Center	Number of Compounds
265	0	5

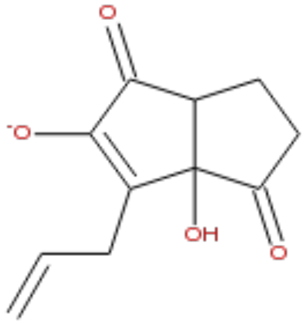

Cell ID	Cluster Center	Number of Compounds
265	0	5

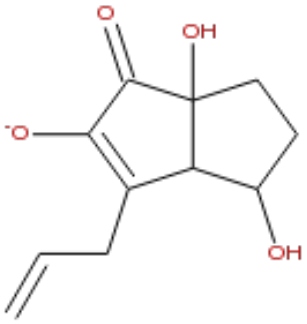

Cell ID	Cluster Center	Number of Compounds
265	0	5

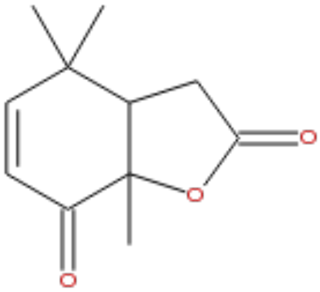

Cell ID	Cluster Center	Number of Compounds
266	1	2

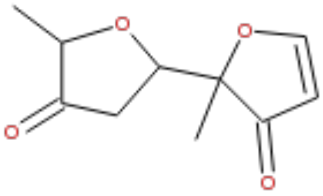

Cell ID	Cluster Center	Number of Compounds
266	0	2

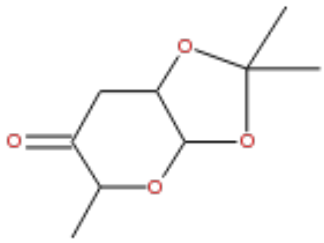

Cell ID	Cluster Center	Number of Compounds
267	1	2

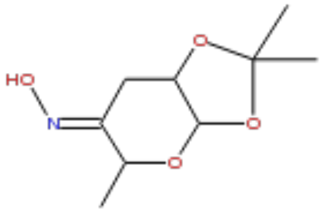

Cell ID	Cluster Center	Number of Compounds
267	0	2

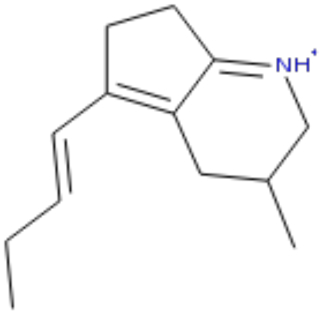

Cell ID	Cluster Center	Number of Compounds
270	1	1

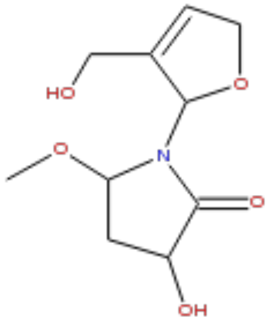

Cell ID	Cluster Center	Number of Compounds
271	1	4

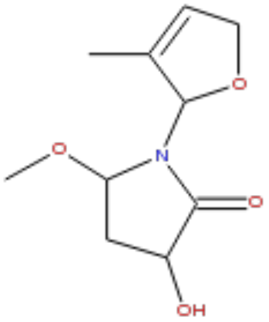

Cell ID	Cluster Center	Number of Compounds
271	0	4

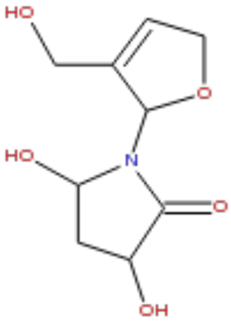

Cell ID	Cluster Center	Number of Compounds
271	0	4

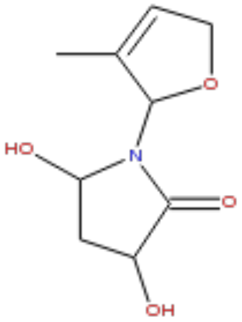

Cell ID	Cluster Center	Number of Compounds
271	0	4

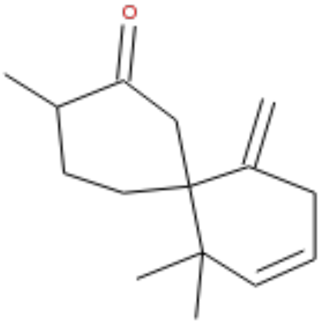

Cell ID	Cluster Center	Number of Compounds
272	1	1

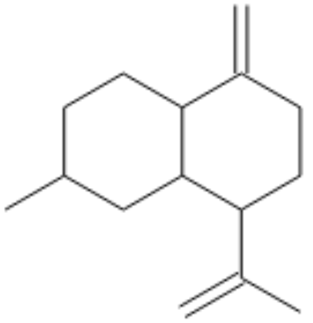

Cell ID	Cluster Center	Number of Compounds
273	1	12

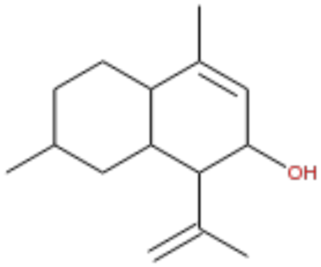

Cell ID	Cluster Center	Number of Compounds
273	0	12

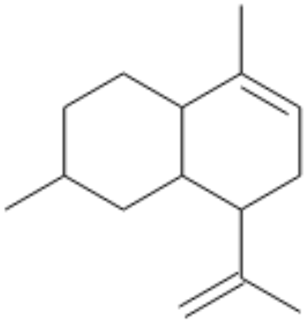

Cell ID	Cluster Center	Number of Compounds
273	0	12

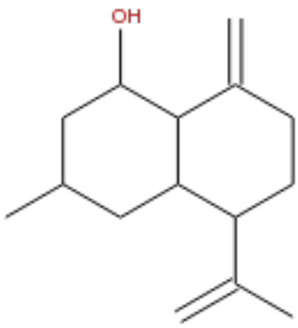

Cell ID	Cluster Center	Number of Compounds
273	0	12

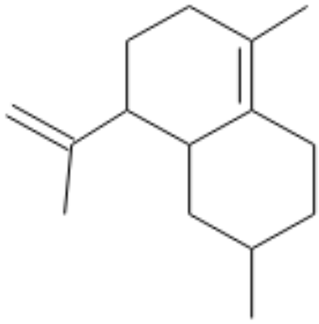

Cell ID	Cluster Center	Number of Compounds
273	0	12

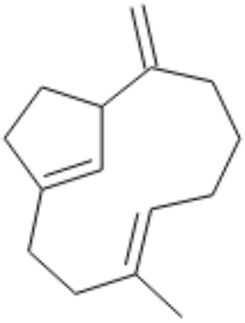

Cell ID	Cluster Center	Number of Compounds
273	0	12

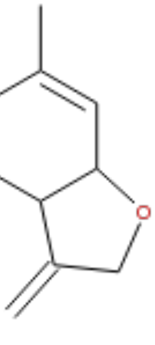

Cell ID	Cluster Center	Number of Compounds
273	0	12

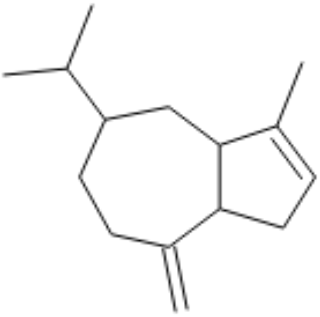

Cell ID	Cluster Center	Number of Compounds
273	0	12

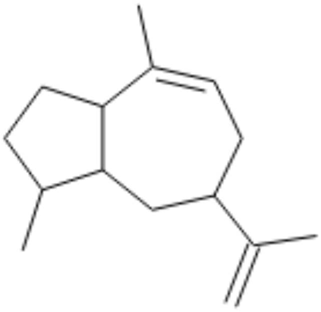

Cell ID	Cluster Center	Number of Compounds
273	0	12

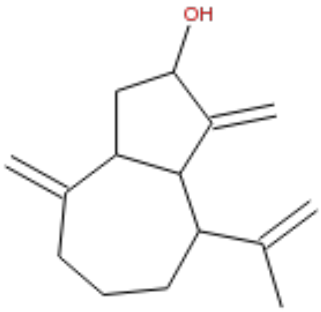

Cell ID	Cluster Center	Number of Compounds
273	0	12

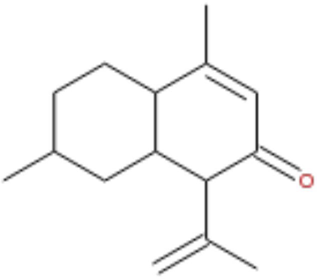

Cell ID	Cluster Center	Number of Compounds
273	0	12

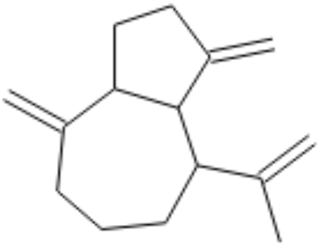

Cell ID	Cluster Center	Number of Compounds
273	0	12

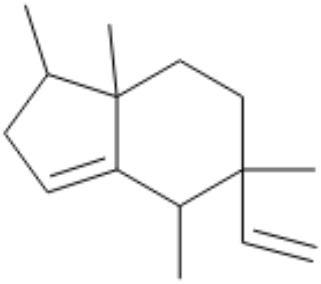

Cell ID	Cluster Center	Number of Compounds
274	1	1

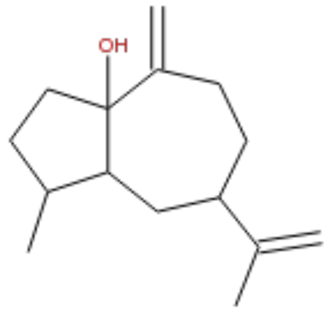

Cell ID	Cluster Center	Number of Compounds
275	1	8

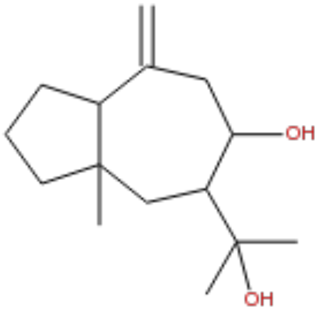

Cell ID	Cluster Center	Number of Compounds
275	0	8

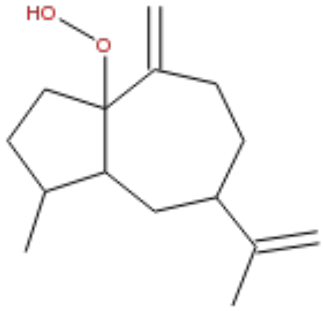

Cell ID	Cluster Center	Number of Compounds
275	0	8

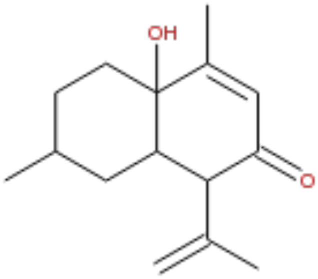

Cell ID	Cluster Center	Number of Compounds
275	0	8

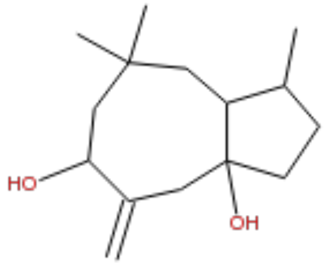

Cell ID	Cluster Center	Number of Compounds
275	0	8

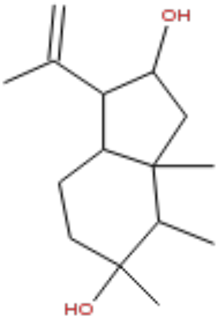

Cell ID	Cluster Center	Number of Compounds
275	0	8

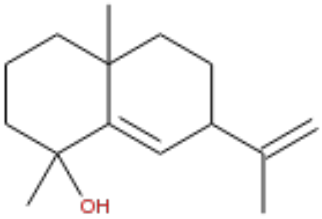

Cell ID	Cluster Center	Number of Compounds
275	0	8

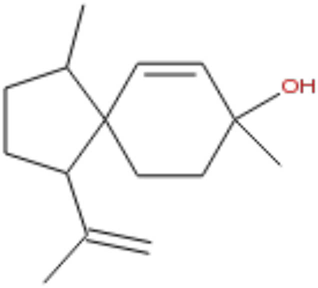

Cell ID	Cluster Center	Number of Compounds
275	0	8

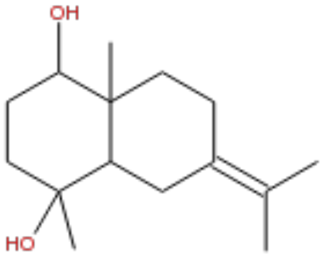

Cell ID	Cluster Center	Number of Compounds
277	1	4

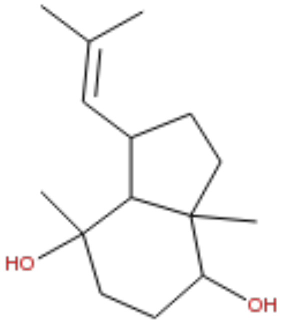

Cell ID	Cluster Center	Number of Compounds
277	0	4

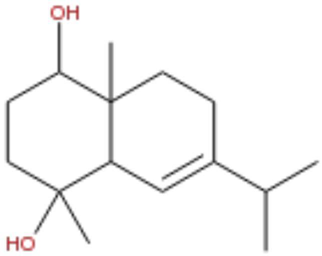

Cell ID	Cluster Center	Number of Compounds
277	0	4

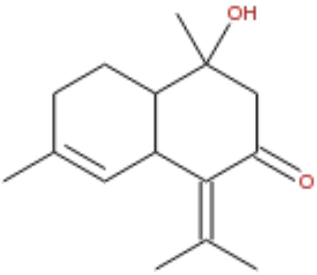

Cell ID	Cluster Center	Number of Compounds
277	0	4

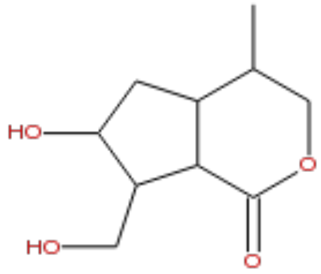

Cell ID	Cluster Center	Number of Compounds
279	1	29

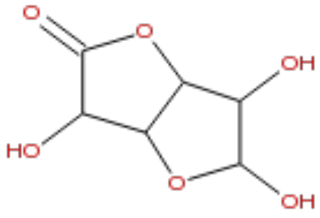

Cell ID	Cluster Center	Number of Compounds
279	0	29

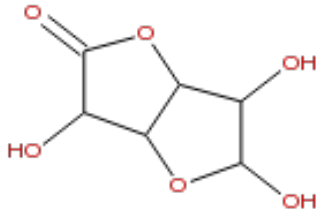

Cell ID	Cluster Center	Number of Compounds
279	0	29

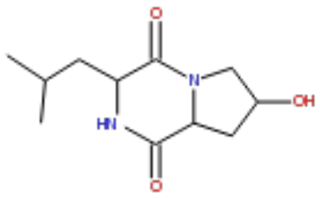

Cell ID	Cluster Center	Number of Compounds
279	0	29

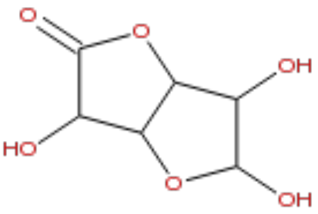

Cell ID	Cluster Center	Number of Compounds
279	0	29

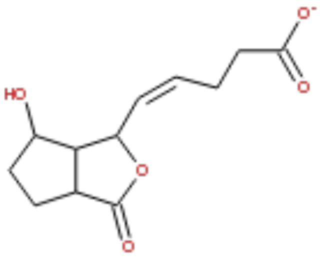

Cell ID	Cluster Center	Number of Compounds
279	0	29

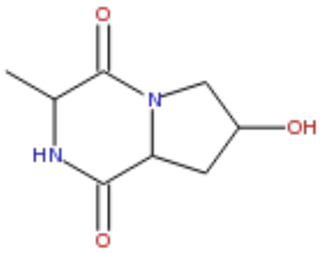

Cell ID	Cluster Center	Number of Compounds
279	0	29

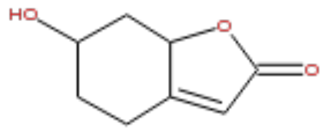

Cell ID	Cluster Center	Number of Compounds
279	0	29

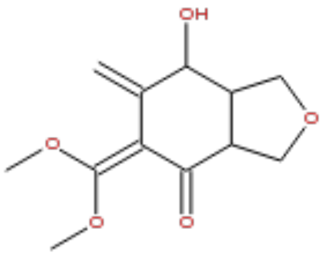

Cell ID	Cluster Center	Number of Compounds
279	0	29

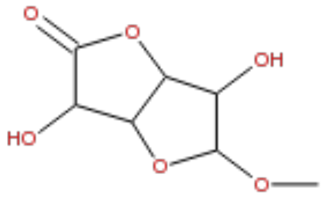

Cell ID	Cluster Center	Number of Compounds
279	0	29

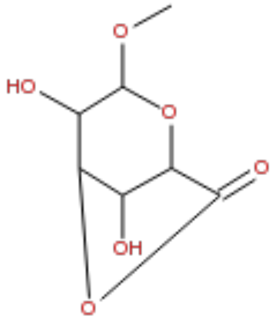

Cell ID	Cluster Center	Number of Compounds
279	0	29

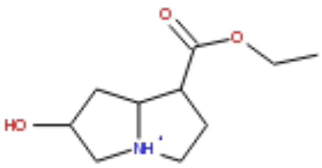

Cell ID	Cluster Center	Number of Compounds
279	0	29

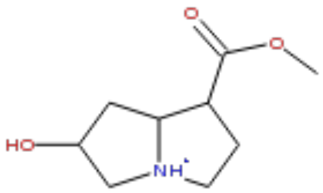

Cell ID	Cluster Center	Number of Compounds
279	0	29

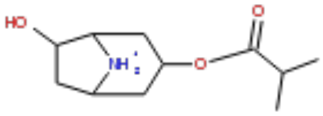

Cell ID	Cluster Center	Number of Compounds
279	0	29

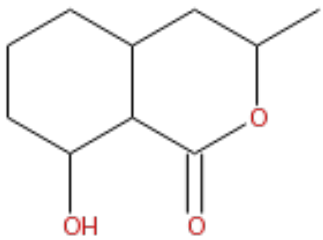

Cell ID	Cluster Center	Number of Compounds
279	0	29

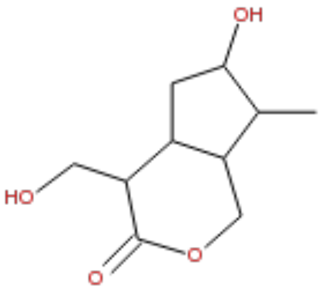

Cell ID	Cluster Center	Number of Compounds
279	0	29

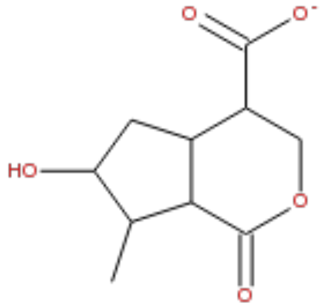

Cell ID	Cluster Center	Number of Compounds
279	0	29

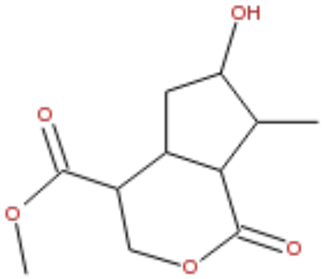

Cell ID	Cluster Center	Number of Compounds
279	0	29

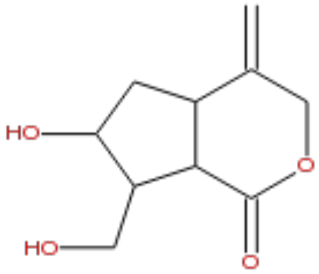

Cell ID	Cluster Center	Number of Compounds
279	0	29

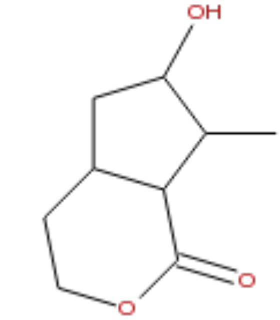

Cell ID	Cluster Center	Number of Compounds
279	0	29

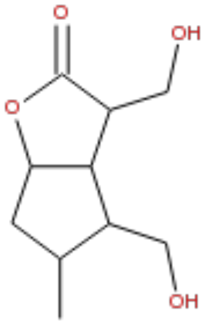

Cell ID	Cluster Center	Number of Compounds
279	0	29

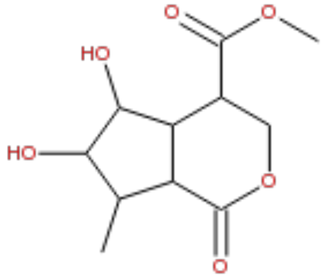

Cell ID	Cluster Center	Number of Compounds
279	0	29

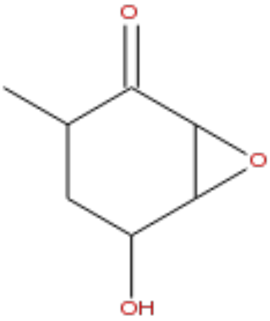

Cell ID	Cluster Center	Number of Compounds
279	0	29

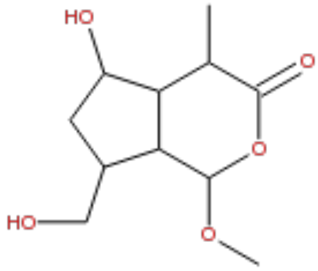

Cell ID	Cluster Center	Number of Compounds
279	0	29

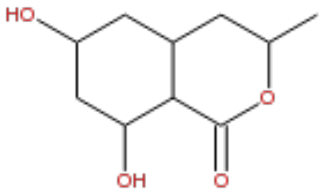

Cell ID	Cluster Center	Number of Compounds
279	0	29

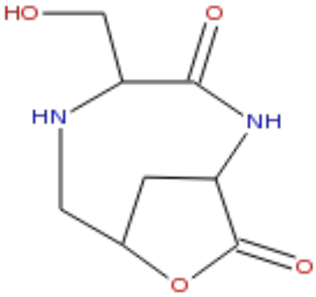

Cell ID	Cluster Center	Number of Compounds
279	0	29

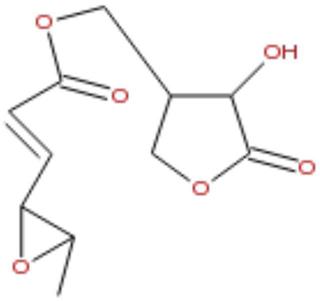

Cell ID	Cluster Center	Number of Compounds
279	0	29

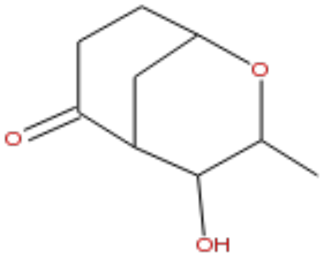

Cell ID	Cluster Center	Number of Compounds
279	0	29

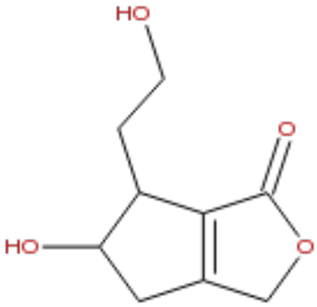

Cell ID	Cluster Center	Number of Compounds
279	0	29

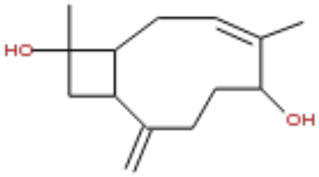

Cell ID	Cluster Center	Number of Compounds
280	1	2

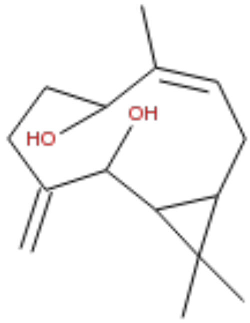

Cell ID	Cluster Center	Number of Compounds
280	0	2

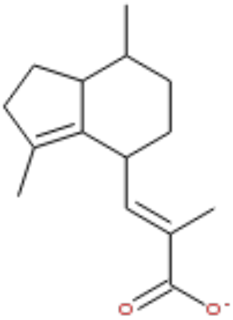

Cell ID	Cluster Center	Number of Compounds
282	1	4

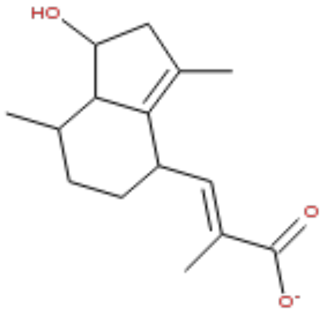

Cell ID	Cluster Center	Number of Compounds
282	0	4

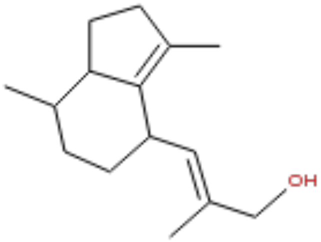

Cell ID	Cluster Center	Number of Compounds
282	0	4

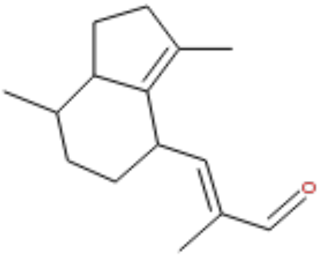

Cell ID	Cluster Center	Number of Compounds
282	0	4

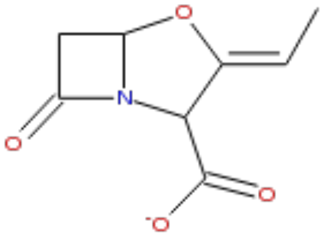

Cell ID	Cluster Center	Number of Compounds
283	1	3

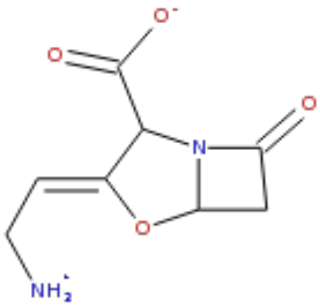

Cell ID	Cluster Center	Number of Compounds
283	0	3

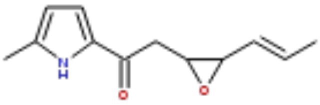

Cell ID	Cluster Center	Number of Compounds
283	0	3

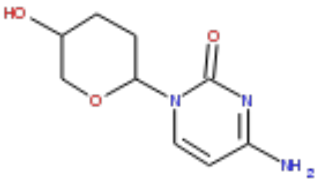

Cell ID	Cluster Center	Number of Compounds
284	1	1

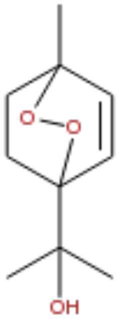

Cell ID	Cluster Center	Number of Compounds
285	1	18

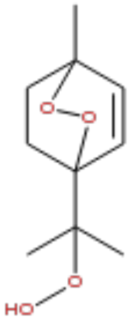

Cell ID	Cluster Center	Number of Compounds
285	0	18

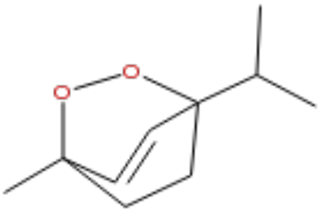

Cell ID	Cluster Center	Number of Compounds
285	0	18

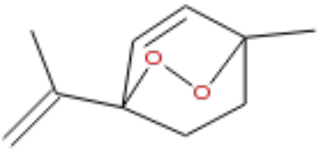

Cell ID	Cluster Center	Number of Compounds
285	0	18

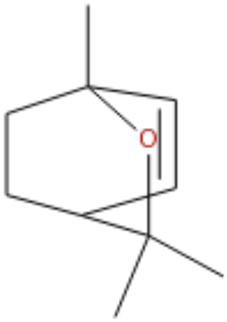

Cell ID	Cluster Center	Number of Compounds
285	0	18

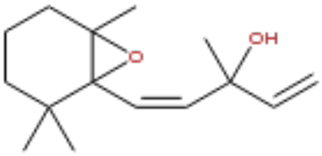

Cell ID	Cluster Center	Number of Compounds
285	0	18

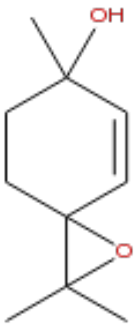

Cell ID	Cluster Center	Number of Compounds
285	0	18

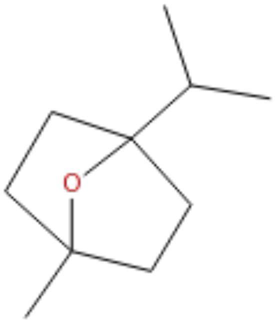

Cell ID	Cluster Center	Number of Compounds
285	0	18

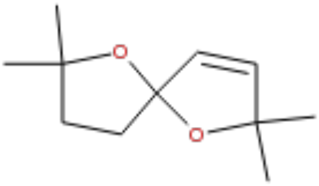

Cell ID	Cluster Center	Number of Compounds
285	0	18

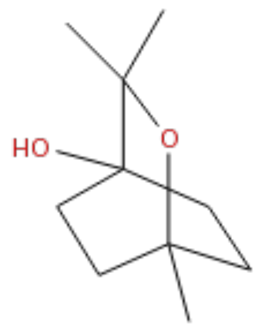

Cell ID	Cluster Center	Number of Compounds
285	0	18

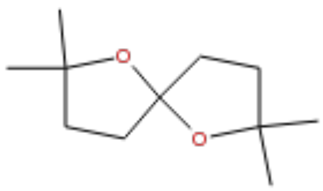

Cell ID	Cluster Center	Number of Compounds
285	0	18

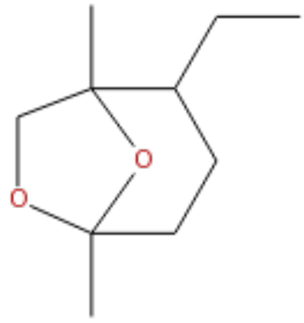

Cell ID	Cluster Center	Number of Compounds
285	0	18

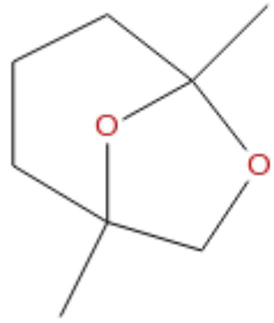

Cell ID	Cluster Center	Number of Compounds
285	0	18

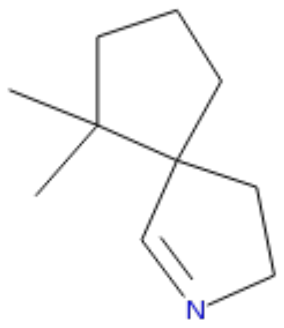

Cell ID	Cluster Center	Number of Compounds
285	0	18

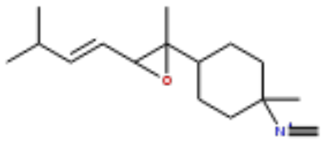

Cell ID	Cluster Center	Number of Compounds
285	0	18

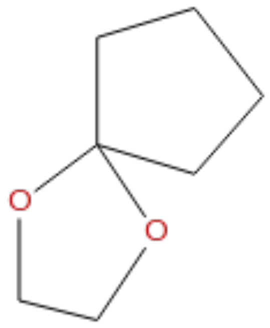

Cell ID	Cluster Center	Number of Compounds
285	0	18

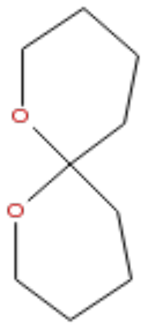

Cell ID	Cluster Center	Number of Compounds
285	0	18

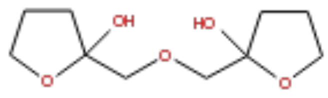

Cell ID	Cluster Center	Number of Compounds
285	0	18

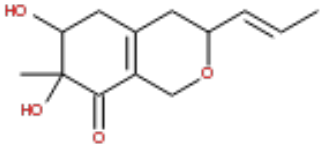

Cell ID	Cluster Center	Number of Compounds
289	1	1

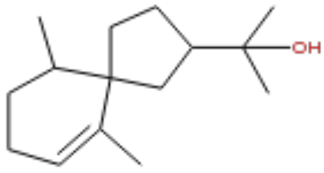

Cell ID	Cluster Center	Number of Compounds
291	1	8

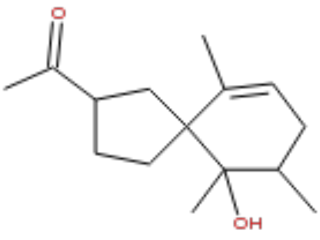

Cell ID	Cluster Center	Number of Compounds
291	0	8

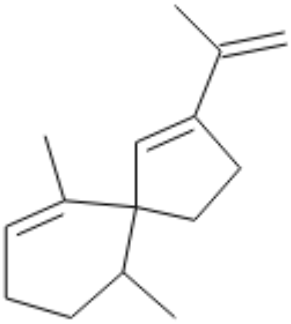

Cell ID	Cluster Center	Number of Compounds
291	0	8

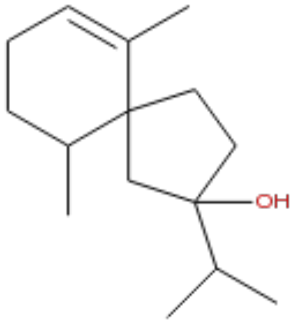

Cell ID	Cluster Center	Number of Compounds
291	0	8

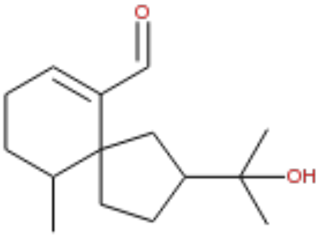

Cell ID	Cluster Center	Number of Compounds
291	0	8

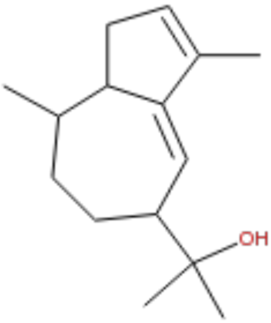

Cell ID	Cluster Center	Number of Compounds
291	0	8

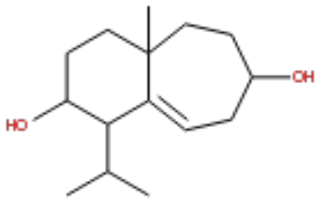

Cell ID	Cluster Center	Number of Compounds
291	0	8

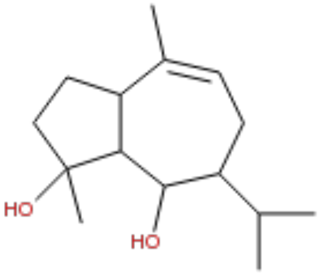

Cell ID	Cluster Center	Number of Compounds
291	0	8

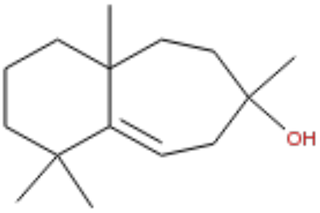

Cell ID	Cluster Center	Number of Compounds
293	1	7

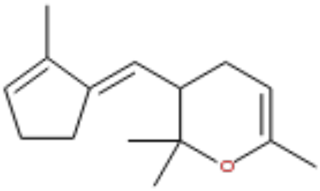

Cell ID	Cluster Center	Number of Compounds
293	0	7

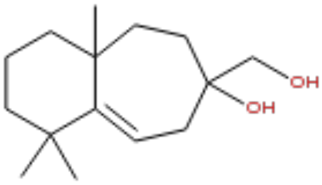

Cell ID	Cluster Center	Number of Compounds
293	0	7

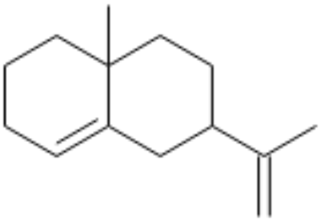

Cell ID	Cluster Center	Number of Compounds
293	0	7

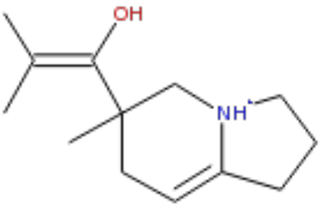

Cell ID	Cluster Center	Number of Compounds
293	0	7

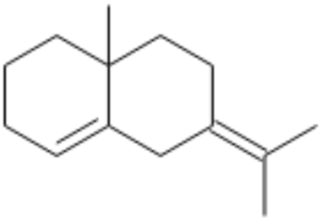

Cell ID	Cluster Center	Number of Compounds
293	0	7

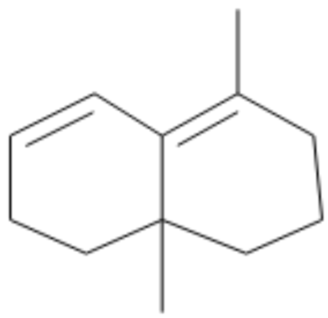

Cell ID	Cluster Center	Number of Compounds
293	0	7

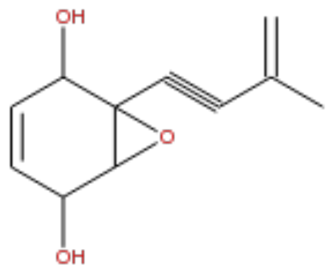

Cell ID	Cluster Center	Number of Compounds
294	1	5

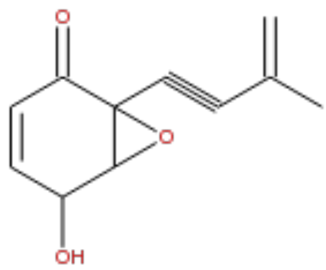

Cell ID	Cluster Center	Number of Compounds
294	0	5

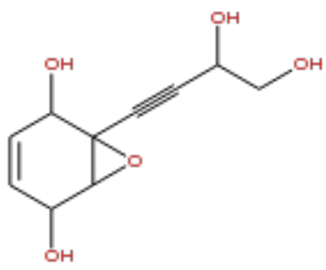

Cell ID	Cluster Center	Number of Compounds
294	0	5

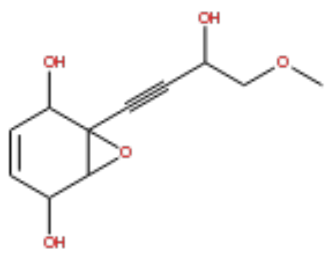

Cell ID	Cluster Center	Number of Compounds
294	0	5

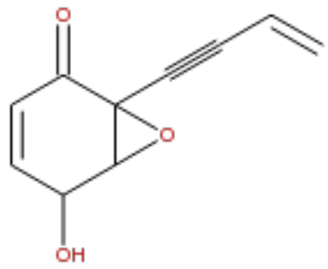

Cell ID	Cluster Center	Number of Compounds
294	0	5

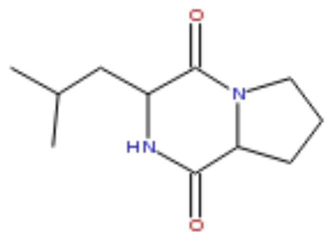

Cell ID	Cluster Center	Number of Compounds
295	1	13

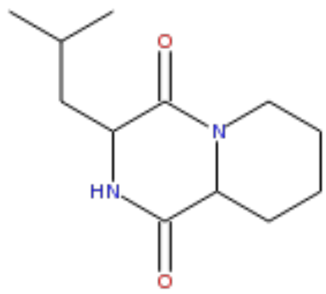

Cell ID	Cluster Center	Number of Compounds
295	0	13

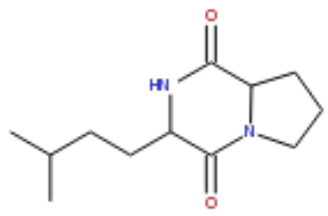

Cell ID	Cluster Center	Number of Compounds
295	0	13

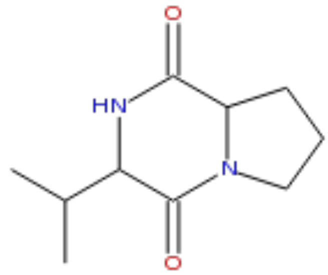

Cell ID	Cluster Center	Number of Compounds
295	0	13

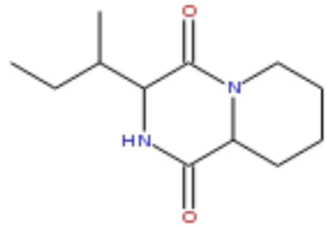

Cell ID	Cluster Center	Number of Compounds
295	0	13

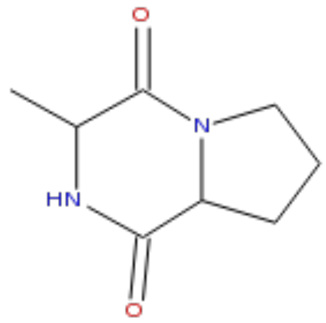

Cell ID	Cluster Center	Number of Compounds
295	0	13

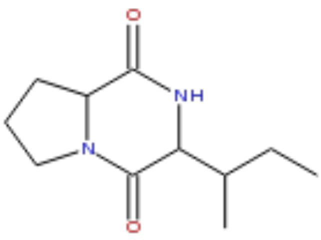

Cell ID	Cluster Center	Number of Compounds
295	0	13

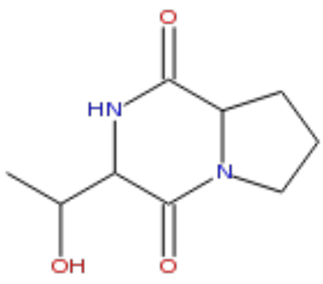

Cell ID	Cluster Center	Number of Compounds
295	0	13

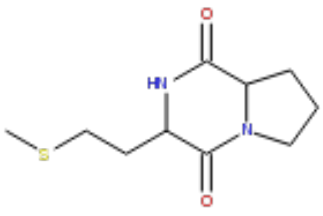

Cell ID	Cluster Center	Number of Compounds
295	0	13

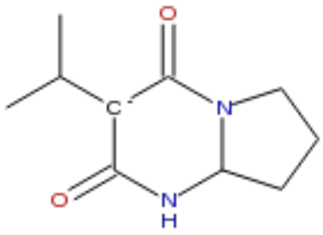

Cell ID	Cluster Center	Number of Compounds
295	0	13

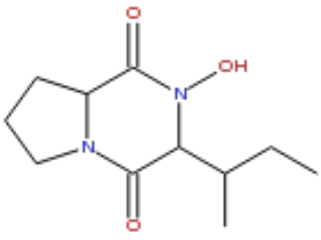

Cell ID	Cluster Center	Number of Compounds
295	0	13

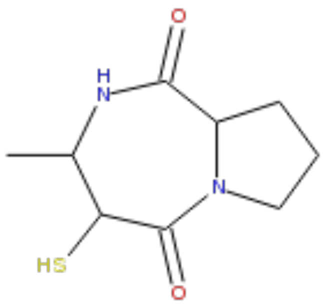

Cell ID	Cluster Center	Number of Compounds
295	0	13

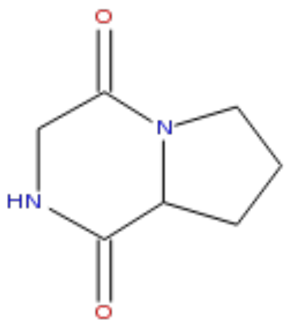

Cell ID	Cluster Center	Number of Compounds
295	0	13

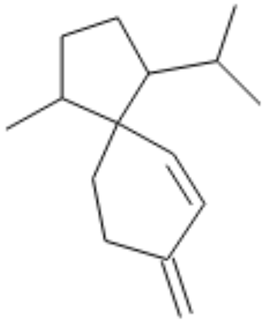

Cell ID	Cluster Center	Number of Compounds
296	1	5

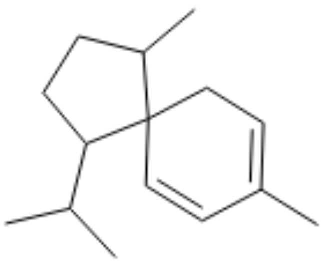

Cell ID	Cluster Center	Number of Compounds
296	0	5

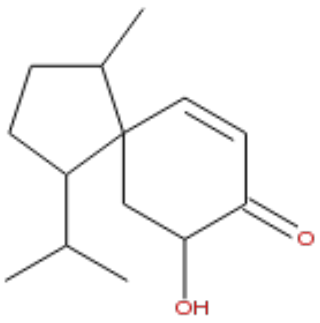

Cell ID	Cluster Center	Number of Compounds
296	0	5

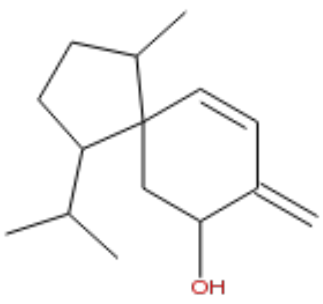

Cell ID	Cluster Center	Number of Compounds
296	0	5

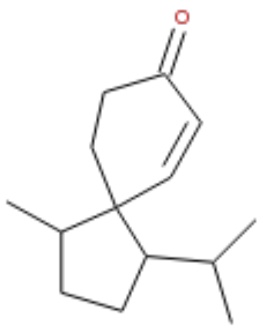

Cell ID	Cluster Center	Number of Compounds
296	0	5

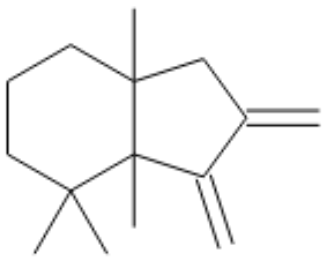

Cell ID	Cluster Center	Number of Compounds
297	1	2

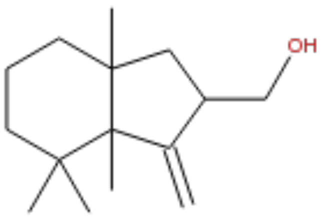

Cell ID	Cluster Center	Number of Compounds
297	0	2

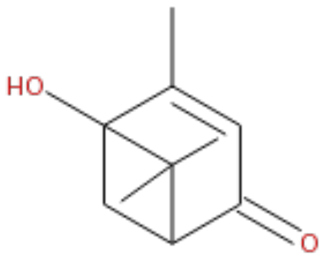

Cell ID	Cluster Center	Number of Compounds
299	1	15

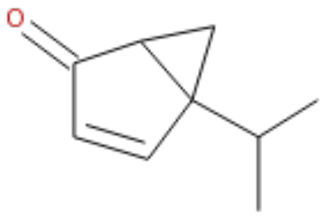

Cell ID	Cluster Center	Number of Compounds
299	0	15

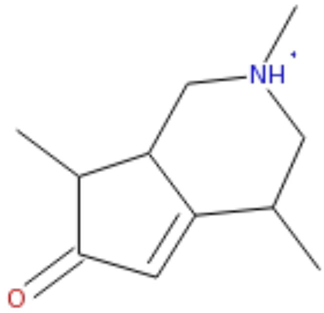

Cell ID	Cluster Center	Number of Compounds
299	0	15

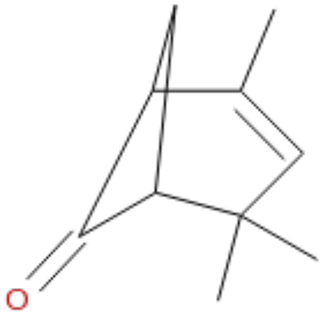

Cell ID	Cluster Center	Number of Compounds
299	0	15

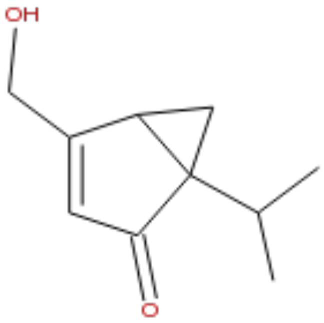

Cell ID	Cluster Center	Number of Compounds
299	0	15

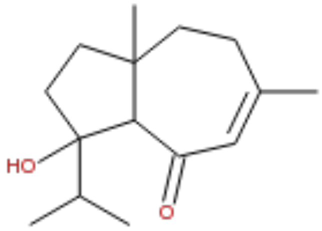

Cell ID	Cluster Center	Number of Compounds
299	0	15

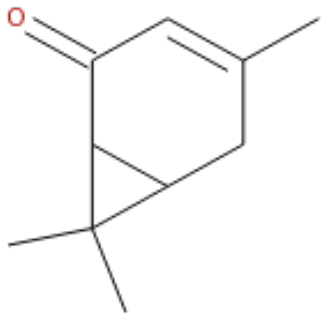

Cell ID	Cluster Center	Number of Compounds
299	0	15

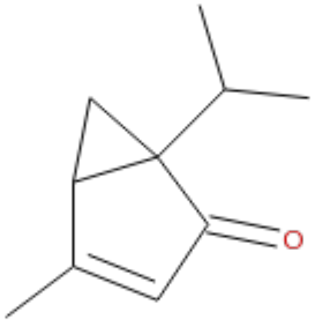

Cell ID	Cluster Center	Number of Compounds
299	0	15

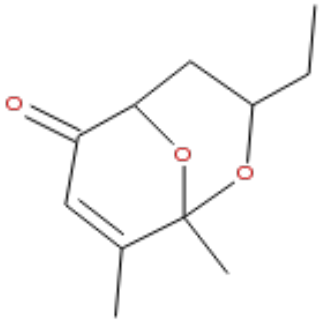

Cell ID	Cluster Center	Number of Compounds
299	0	15

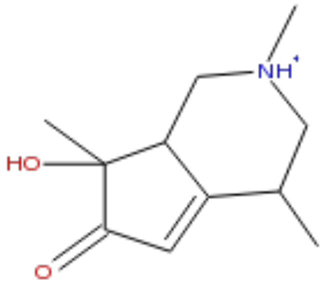

Cell ID	Cluster Center	Number of Compounds
299	0	15

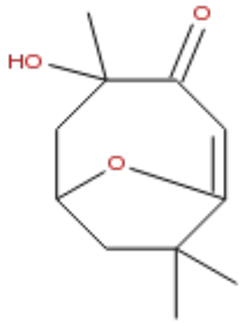

Cell ID	Cluster Center	Number of Compounds
299	0	15

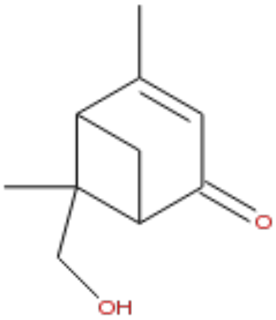

Cell ID	Cluster Center	Number of Compounds
299	0	15

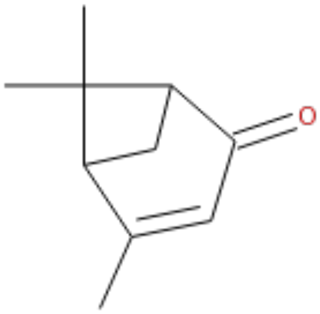

Cell ID	Cluster Center	Number of Compounds
299	0	15

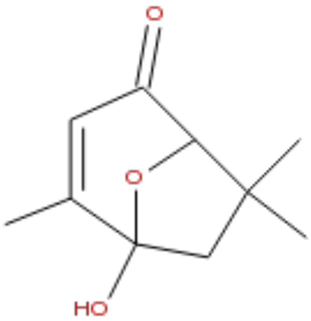

Cell ID	Cluster Center	Number of Compounds
299	0	15

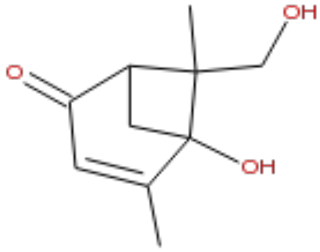

Cell ID	Cluster Center	Number of Compounds
299	0	15

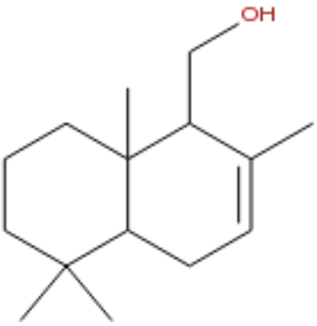

Cell ID	Cluster Center	Number of Compounds
300	1	5

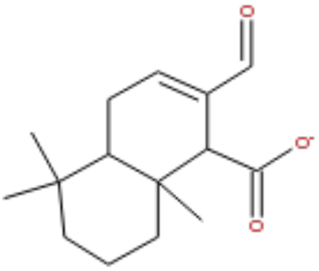

Cell ID	Cluster Center	Number of Compounds
300	0	5

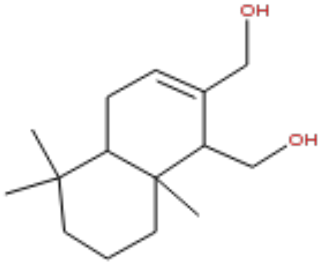

Cell ID	Cluster Center	Number of Compounds
300	0	5

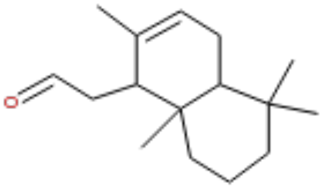

Cell ID	Cluster Center	Number of Compounds
300	0	5

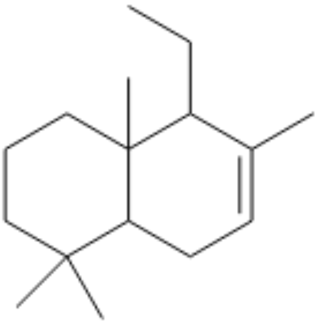

Cell ID	Cluster Center	Number of Compounds
300	0	5

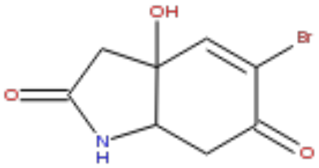

Cell ID	Cluster Center	Number of Compounds
301	1	5

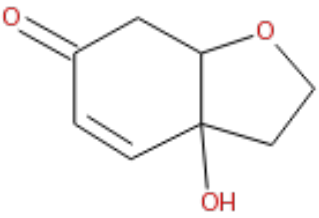

Cell ID	Cluster Center	Number of Compounds
301	0	5

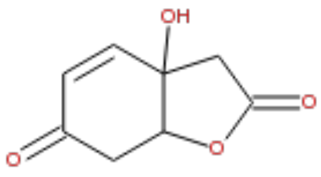

Cell ID	Cluster Center	Number of Compounds
301	0	5

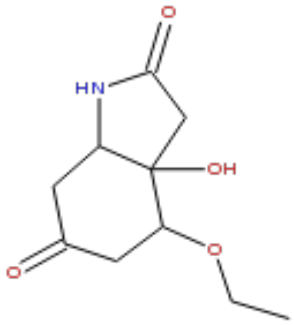

Cell ID	Cluster Center	Number of Compounds
301	0	5

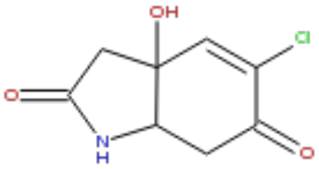

Cell ID	Cluster Center	Number of Compounds
301	0	5

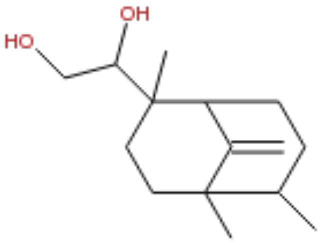

Cell ID	Cluster Center	Number of Compounds
302	1	3

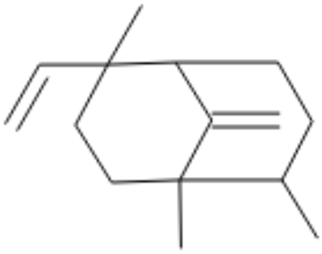

Cell ID	Cluster Center	Number of Compounds
302	0	3

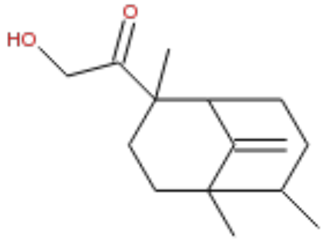

Cell ID	Cluster Center	Number of Compounds
302	0	3

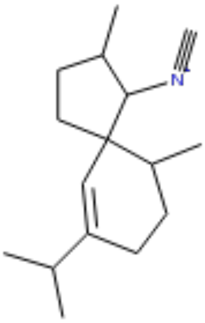

Cell ID	Cluster Center	Number of Compounds
304	1	1

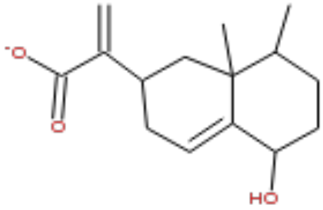

Cell ID	Cluster Center	Number of Compounds
305	1	3

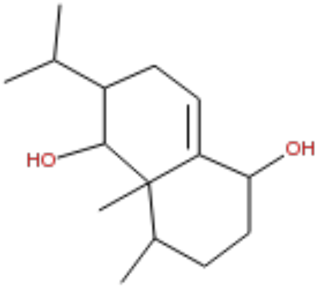

Cell ID	Cluster Center	Number of Compounds
305	0	3

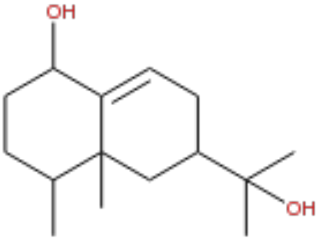

Cell ID	Cluster Center	Number of Compounds
305	0	3

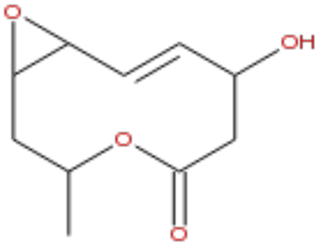

Cell ID	Cluster Center	Number of Compounds
308	1	15

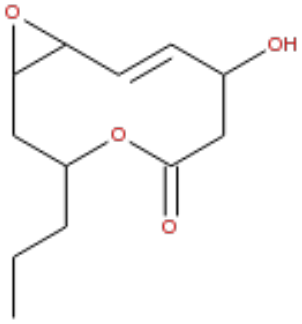

Cell ID	Cluster Center	Number of Compounds
308	0	15

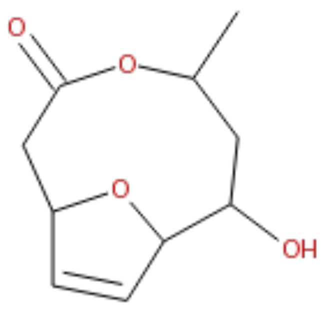

Cell ID	Cluster Center	Number of Compounds
308	0	15

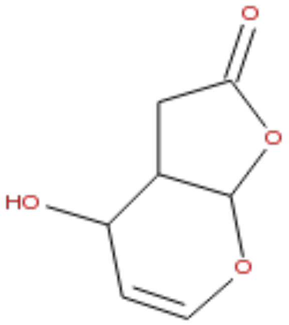

Cell ID	Cluster Center	Number of Compounds
308	0	15

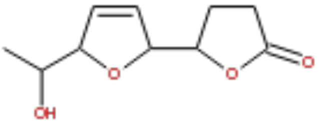

Cell ID	Cluster Center	Number of Compounds
308	0	15

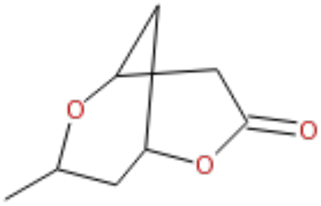

Cell ID	Cluster Center	Number of Compounds
308	0	15

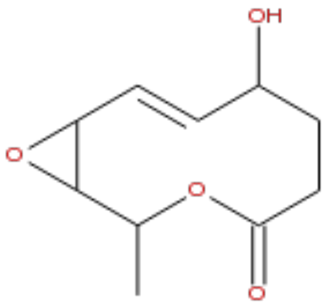

Cell ID	Cluster Center	Number of Compounds
308	0	15

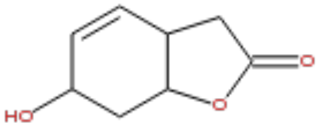

Cell ID	Cluster Center	Number of Compounds
308	0	15

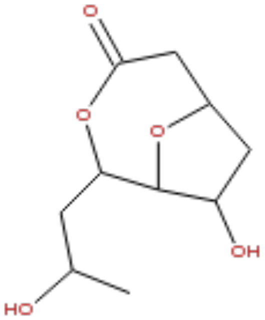

Cell ID	Cluster Center	Number of Compounds
308	0	15

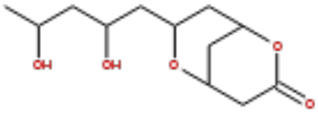

Cell ID	Cluster Center	Number of Compounds
308	0	15

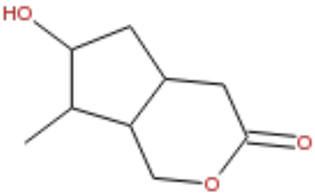

Cell ID	Cluster Center	Number of Compounds
308	0	15

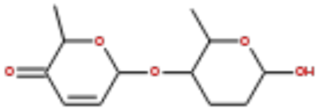

Cell ID	Cluster Center	Number of Compounds
308	0	15

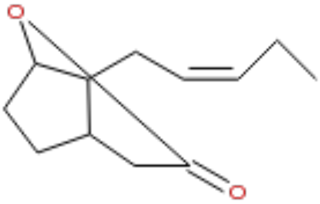

Cell ID	Cluster Center	Number of Compounds
308	0	15

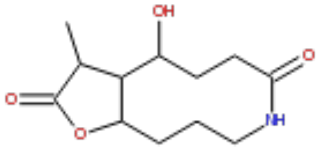

Cell ID	Cluster Center	Number of Compounds
308	0	15

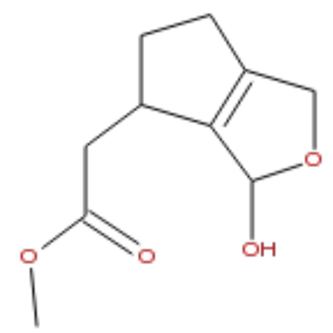

Cell ID	Cluster Center	Number of Compounds
308	0	15

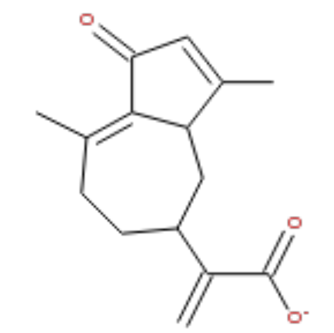

Cell ID	Cluster Center	Number of Compounds
310	1	4

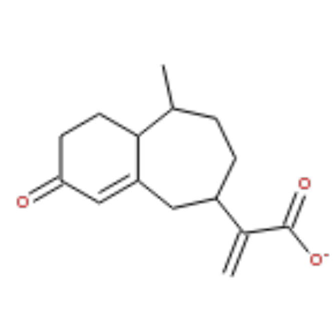

Cell ID	Cluster Center	Number of Compounds
310	0	4

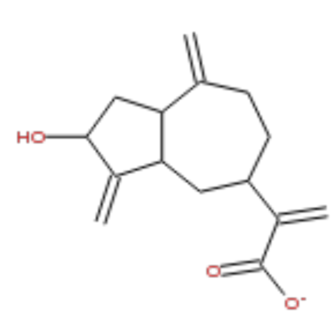

Cell ID	Cluster Center	Number of Compounds
310	0	4

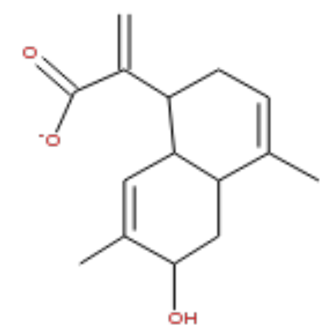

Cell ID	Cluster Center	Number of Compounds
310	0	4

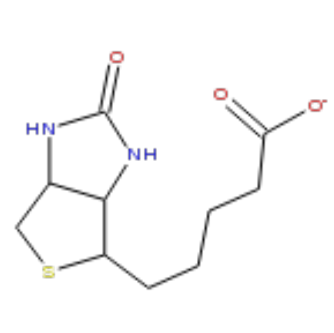

Cell ID	Cluster Center	Number of Compounds
311	1	4

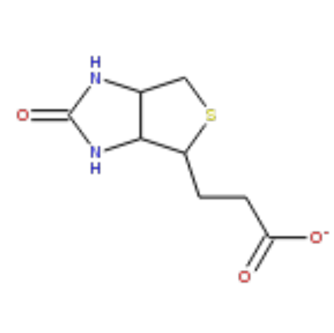

Cell ID	Cluster Center	Number of Compounds
311	0	4

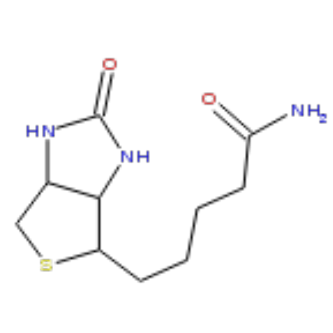

Cell ID	Cluster Center	Number of Compounds
311	0	4

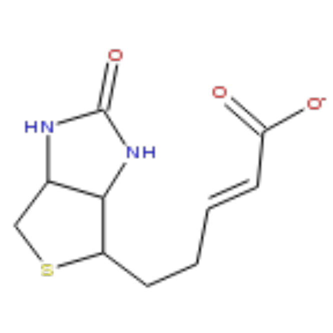

Cell ID	Cluster Center	Number of Compounds
311	0	4

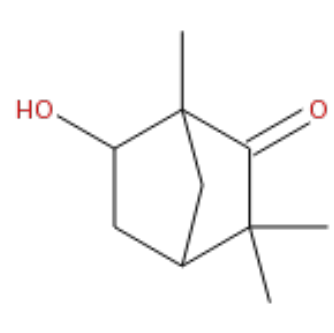

Cell ID	Cluster Center	Number of Compounds
312	1	12

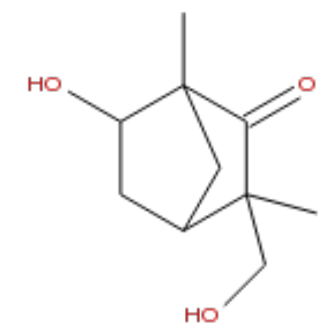

Cell ID	Cluster Center	Number of Compounds
312	0	12

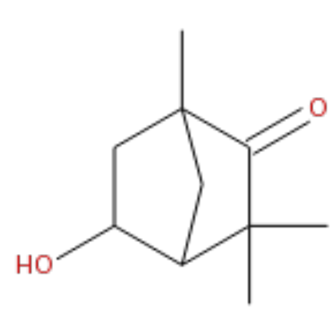

Cell ID	Cluster Center	Number of Compounds
312	0	12

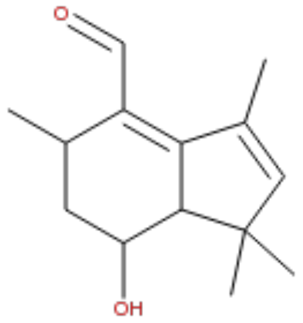

Cell ID	Cluster Center	Number of Compounds
312	0	12

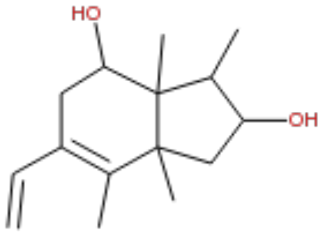

Cell ID	Cluster Center	Number of Compounds
312	0	12

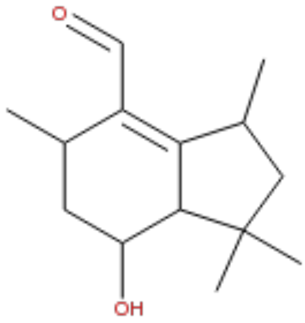

Cell ID	Cluster Center	Number of Compounds
312	0	12

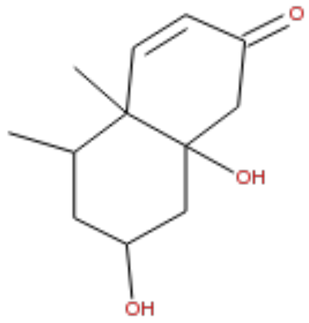

Cell ID	Cluster Center	Number of Compounds
312	0	12

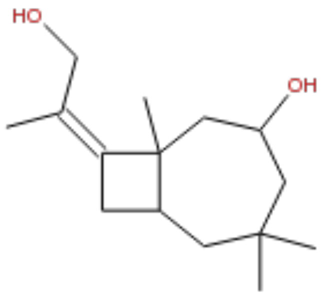

Cell ID	Cluster Center	Number of Compounds
312	0	12

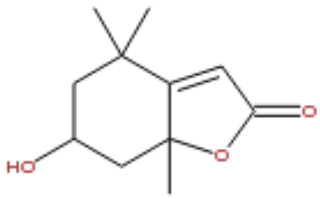

Cell ID	Cluster Center	Number of Compounds
312	0	12

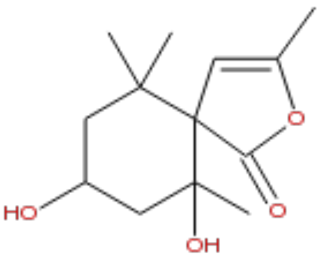

Cell ID	Cluster Center	Number of Compounds
312	0	12

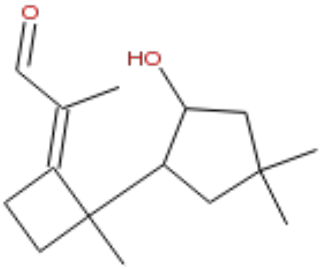

Cell ID	Cluster Center	Number of Compounds
312	0	12

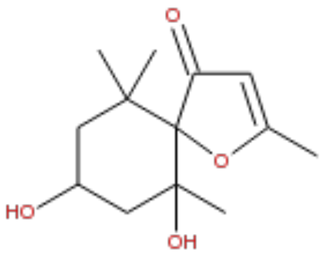

Cell ID	Cluster Center	Number of Compounds
312	0	12

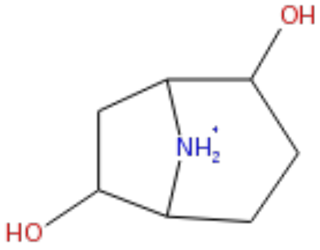

Cell ID	Cluster Center	Number of Compounds
315	1	14

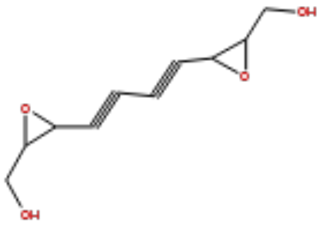

Cell ID	Cluster Center	Number of Compounds
315	0	14

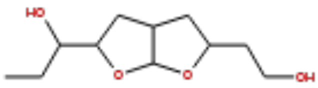

Cell ID	Cluster Center	Number of Compounds
315	0	14

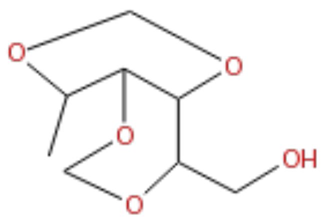

Cell ID	Cluster Center	Number of Compounds
315	0	14

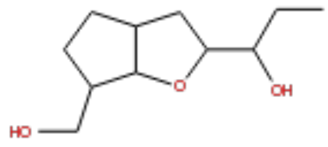

Cell ID	Cluster Center	Number of Compounds
315	0	14

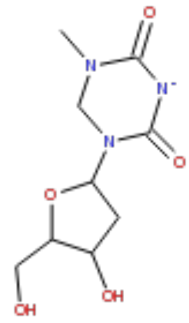

Cell ID	Cluster Center	Number of Compounds
315	0	14

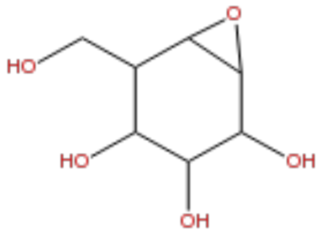

Cell ID	Cluster Center	Number of Compounds
315	0	14

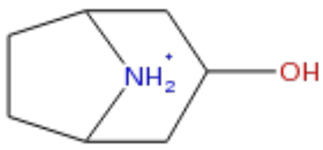

Cell ID	Cluster Center	Number of Compounds
315	0	14

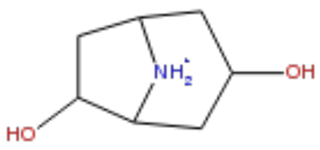

Cell ID	Cluster Center	Number of Compounds
315	0	14

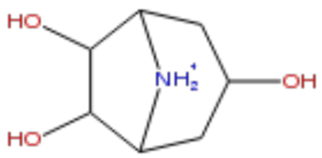

Cell ID	Cluster Center	Number of Compounds
315	0	14

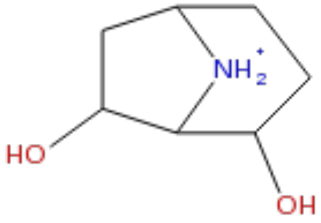

Cell ID	Cluster Center	Number of Compounds
315	0	14

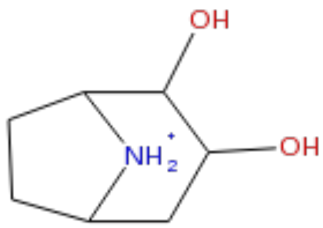

Cell ID	Cluster Center	Number of Compounds
315	0	14

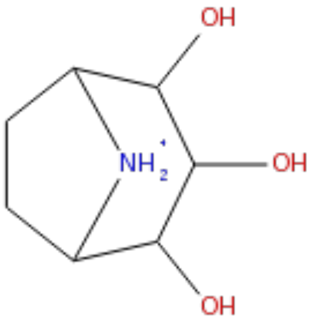

Cell ID	Cluster Center	Number of Compounds
315	0	14

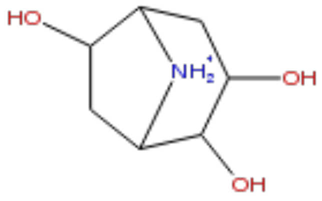

Cell ID	Cluster Center	Number of Compounds
315	0	14

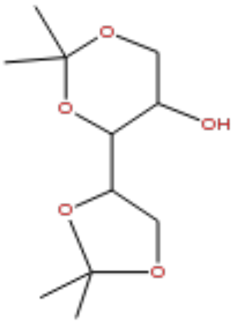

Cell ID	Cluster Center	Number of Compounds
316	1	29

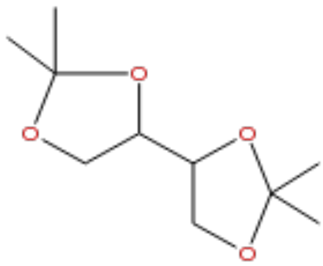

Cell ID	Cluster Center	Number of Compounds
316	0	29

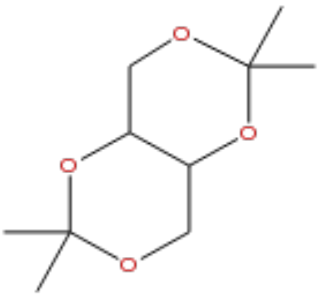

Cell ID	Cluster Center	Number of Compounds
316	0	29

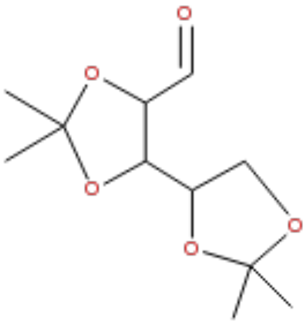

Cell ID	Cluster Center	Number of Compounds
316	0	29

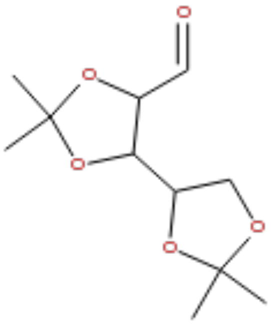

Cell ID	Cluster Center	Number of Compounds
316	0	29

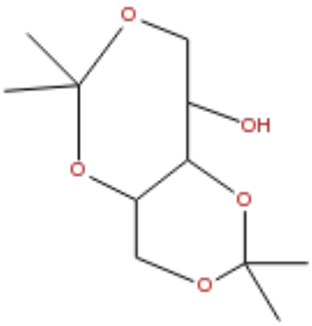

Cell ID	Cluster Center	Number of Compounds
316	0	29

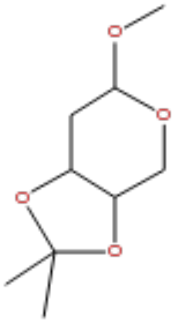

Cell ID	Cluster Center	Number of Compounds
316	0	29

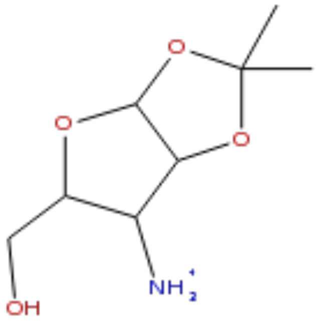

Cell ID	Cluster Center	Number of Compounds
316	0	29

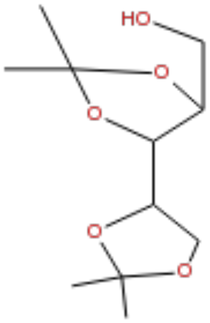

Cell ID	Cluster Center	Number of Compounds
316	0	29

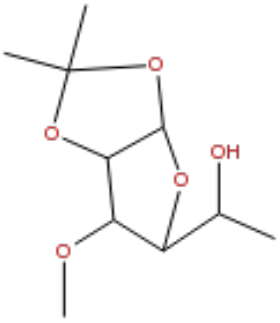

Cell ID	Cluster Center	Number of Compounds
316	0	29

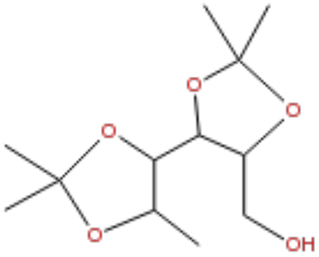

Cell ID	Cluster Center	Number of Compounds
316	0	29

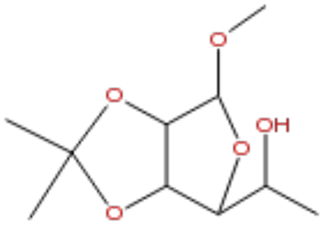

Cell ID	Cluster Center	Number of Compounds
316	0	29

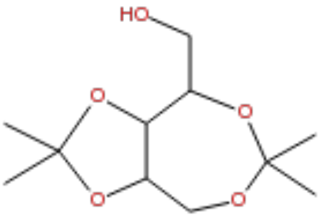

Cell ID	Cluster Center	Number of Compounds
316	0	29

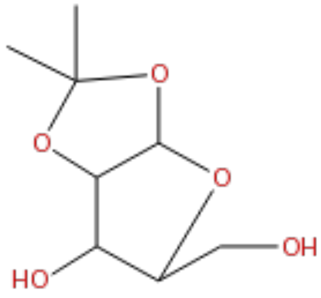

Cell ID	Cluster Center	Number of Compounds
316	0	29

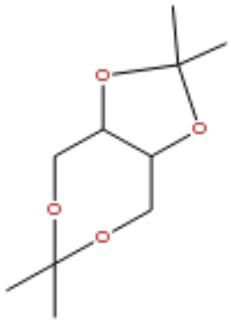

Cell ID	Cluster Center	Number of Compounds
316	0	29

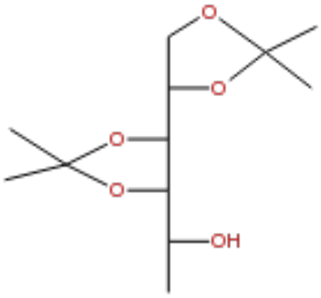

Cell ID	Cluster Center	Number of Compounds
316	0	29

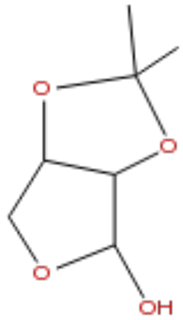

Cell ID	Cluster Center	Number of Compounds
316	0	29

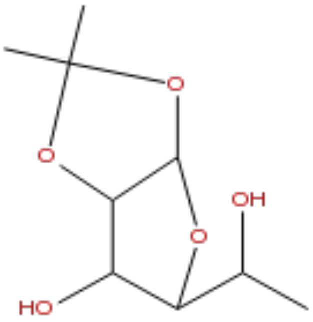

Cell ID	Cluster Center	Number of Compounds
316	0	29

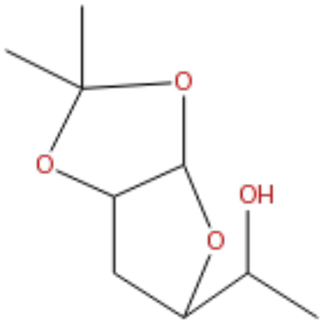

Cell ID	Cluster Center	Number of Compounds
316	0	29

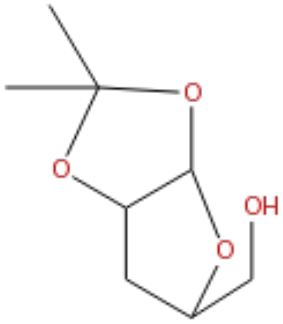

Cell ID	Cluster Center	Number of Compounds
316	0	29

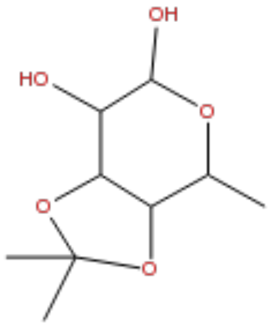

Cell ID	Cluster Center	Number of Compounds
316	0	29

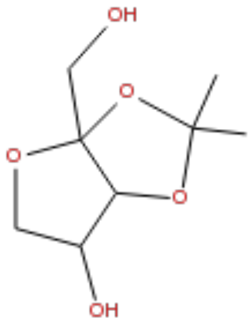

Cell ID	Cluster Center	Number of Compounds
316	0	29

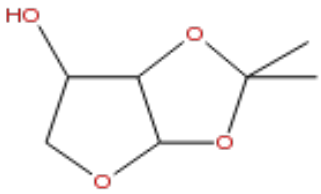

Cell ID	Cluster Center	Number of Compounds
316	0	29

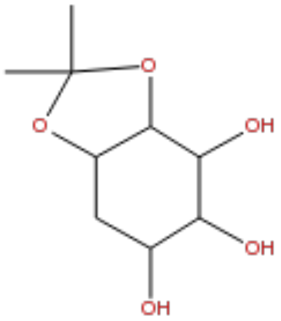

Cell ID	Cluster Center	Number of Compounds
316	0	29

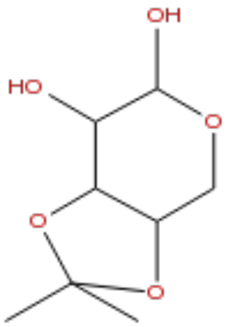

Cell ID	Cluster Center	Number of Compounds
316	0	29

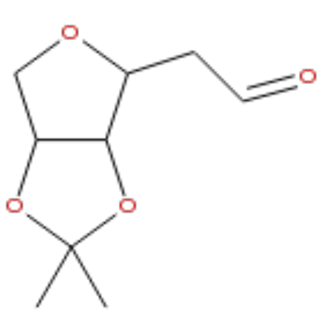

Cell ID	Cluster Center	Number of Compounds
316	0	29

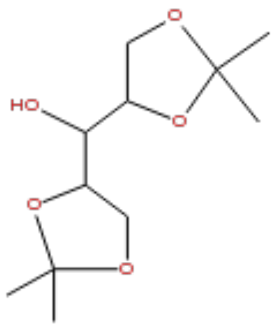

Cell ID	Cluster Center	Number of Compounds
316	0	29

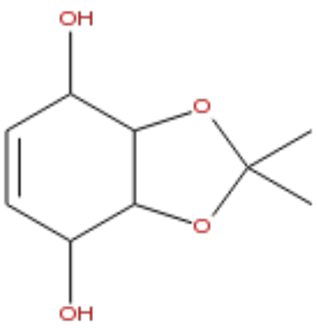

Cell ID	Cluster Center	Number of Compounds
316	0	29

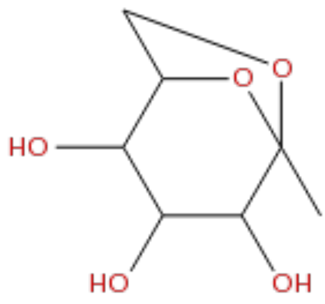

Cell ID	Cluster Center	Number of Compounds
316	0	29

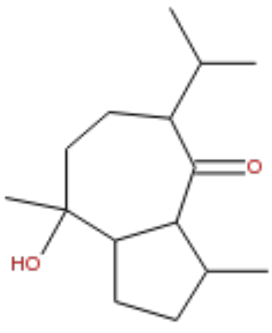

Cell ID	Cluster Center	Number of Compounds
318	1	18

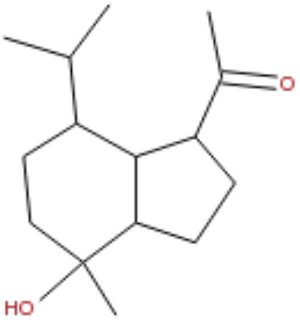

Cell ID	Cluster Center	Number of Compounds
318	0	18

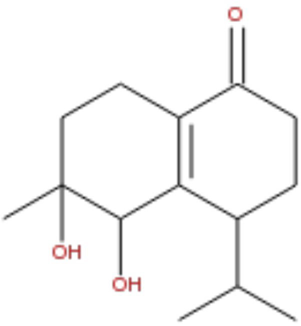

Cell ID	Cluster Center	Number of Compounds
318	0	18

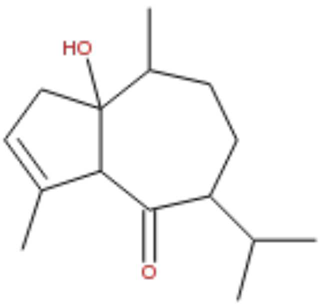

Cell ID	Cluster Center	Number of Compounds
318	0	18

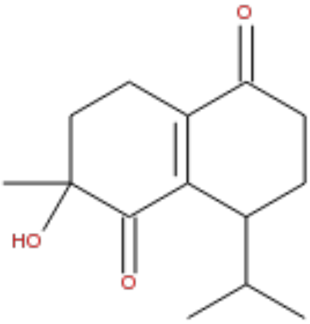

Cell ID	Cluster Center	Number of Compounds
318	0	18

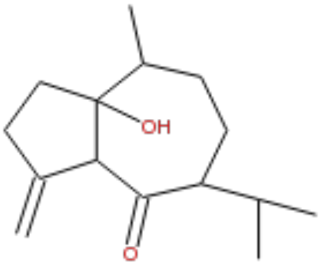

Cell ID	Cluster Center	Number of Compounds
318	0	18

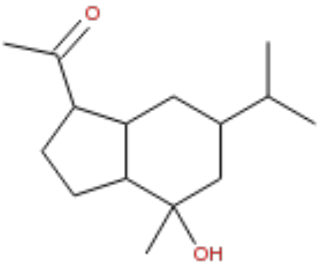

Cell ID	Cluster Center	Number of Compounds
318	0	18

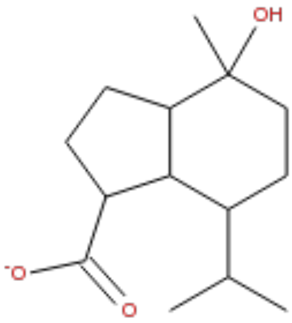

Cell ID	Cluster Center	Number of Compounds
318	0	18

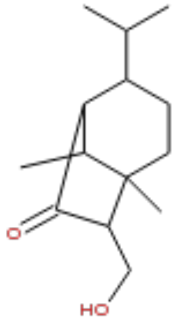

Cell ID	Cluster Center	Number of Compounds
318	0	18

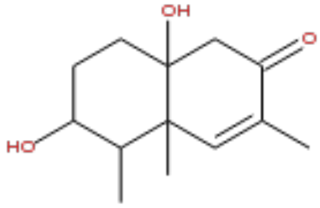

Cell ID	Cluster Center	Number of Compounds
318	0	18

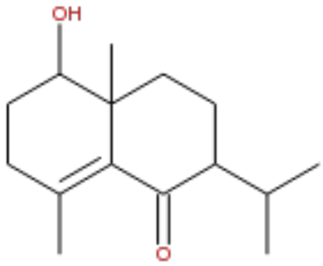

Cell ID	Cluster Center	Number of Compounds
318	0	18

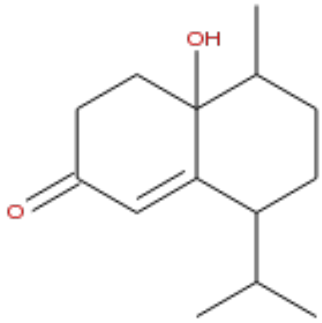

Cell ID	Cluster Center	Number of Compounds
318	0	18

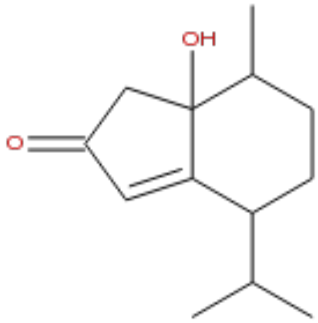

Cell ID	Cluster Center	Number of Compounds
318	0	18

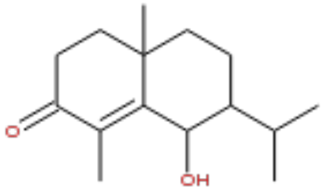

Cell ID	Cluster Center	Number of Compounds
318	0	18

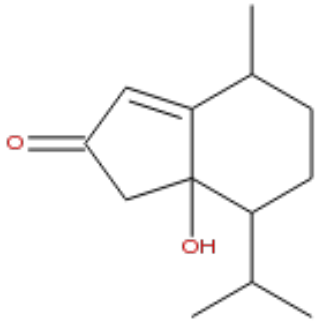

Cell ID	Cluster Center	Number of Compounds
318	0	18

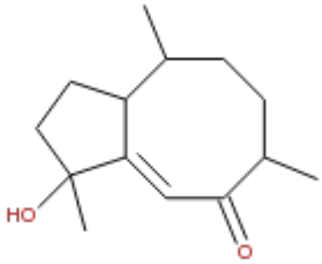

Cell ID	Cluster Center	Number of Compounds
318	0	18

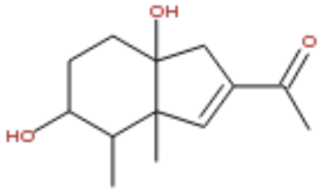

Cell ID	Cluster Center	Number of Compounds
318	0	18

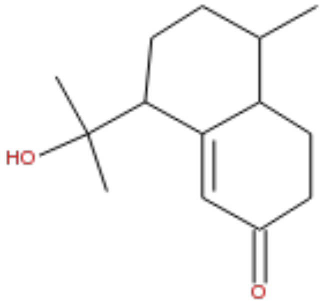

Cell ID	Cluster Center	Number of Compounds
318	0	18

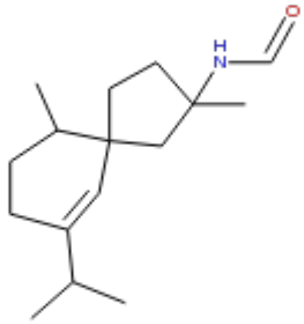

Cell ID	Cluster Center	Number of Compounds
319	1	1

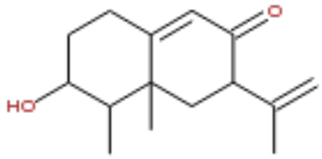

Cell ID	Cluster Center	Number of Compounds
320	1	7

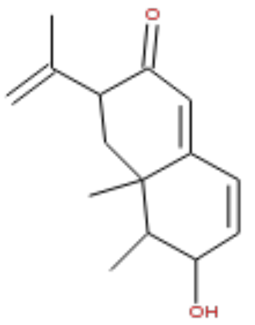

Cell ID	Cluster Center	Number of Compounds
320	0	7

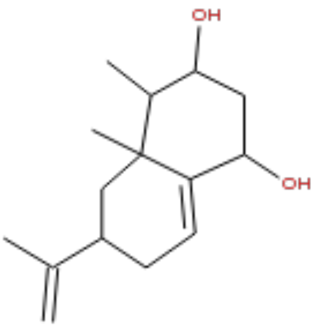

Cell ID	Cluster Center	Number of Compounds
320	0	7

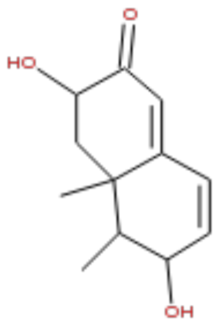

Cell ID	Cluster Center	Number of Compounds
320	0	7

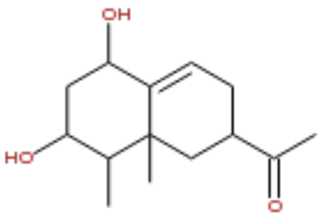

Cell ID	Cluster Center	Number of Compounds
320	0	7

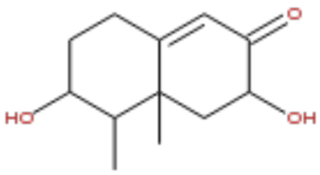

Cell ID	Cluster Center	Number of Compounds
320	0	7

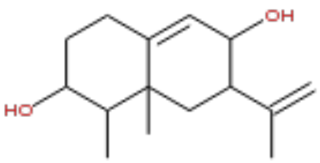

Cell ID	Cluster Center	Number of Compounds
320	0	7

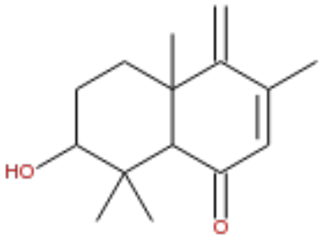

Cell ID	Cluster Center	Number of Compounds
321	1	5

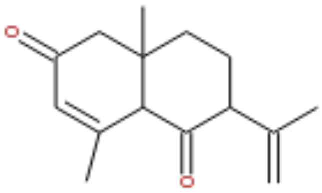

Cell ID	Cluster Center	Number of Compounds
321	0	5

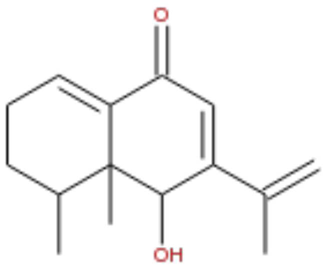

Cell ID	Cluster Center	Number of Compounds
321	0	5

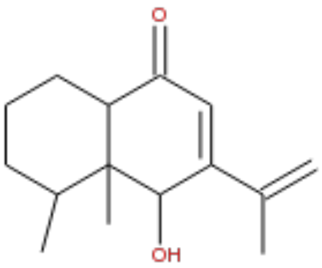

Cell ID	Cluster Center	Number of Compounds
321	0	5

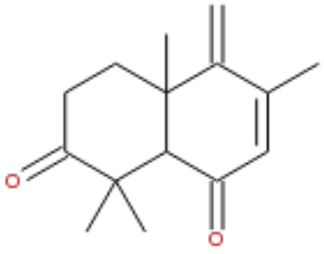

Cell ID	Cluster Center	Number of Compounds
321	0	5

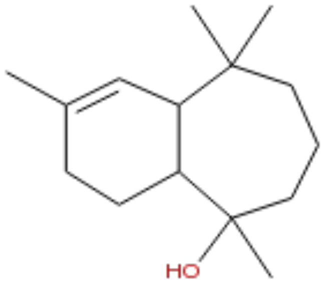

Cell ID	Cluster Center	Number of Compounds
322	1	8

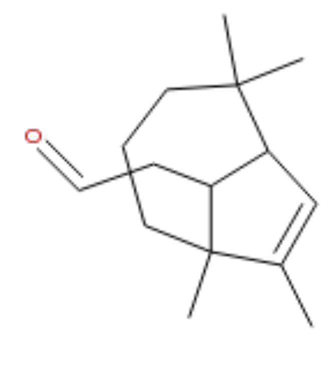

Cell ID	Cluster Center	Number of Compounds
322	0	8

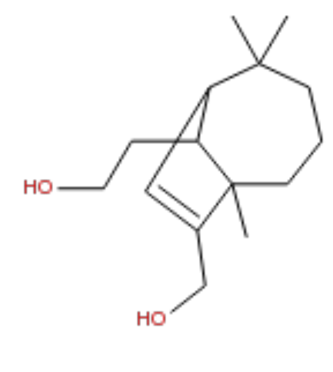

Cell ID	Cluster Center	Number of Compounds
322	0	8

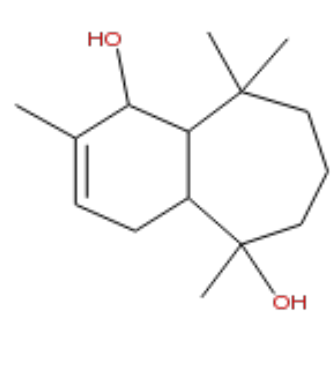

Cell ID	Cluster Center	Number of Compounds
322	0	8

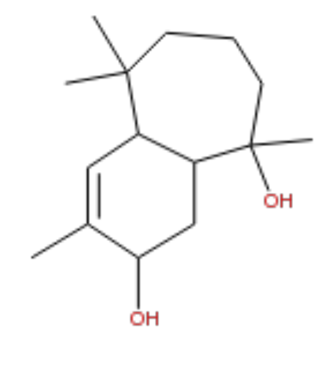

Cell ID	Cluster Center	Number of Compounds
322	0	8

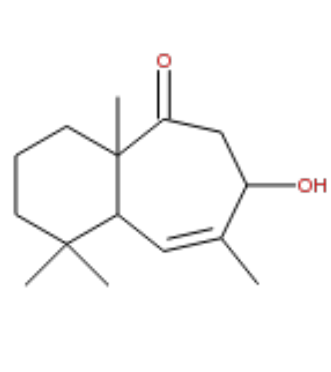

Cell ID	Cluster Center	Number of Compounds
322	0	8

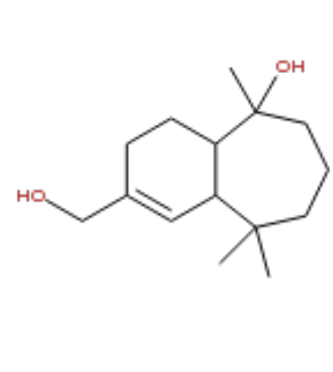

Cell ID	Cluster Center	Number of Compounds
322	0	8

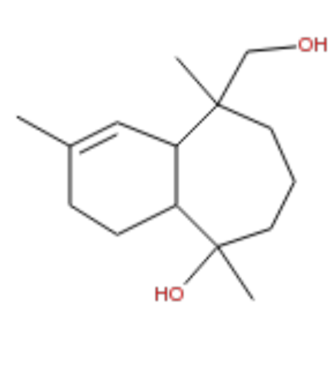

Cell ID	Cluster Center	Number of Compounds
322	0	8

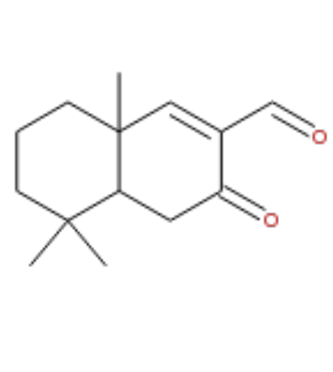

Cell ID	Cluster Center	Number of Compounds
323	1	6

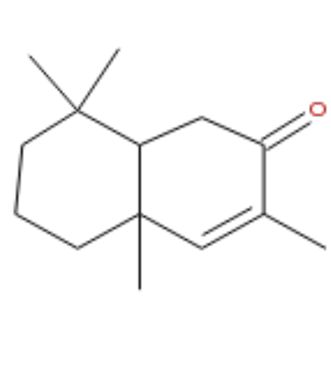

Cell ID	Cluster Center	Number of Compounds
323	0	6

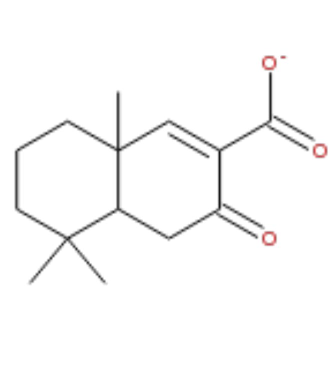

Cell ID	Cluster Center	Number of Compounds
323	0	6

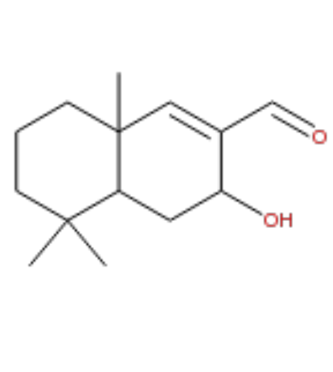

Cell ID	Cluster Center	Number of Compounds
323	0	6

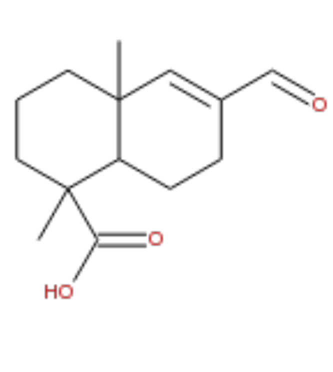

Cell ID	Cluster Center	Number of Compounds
323	0	6

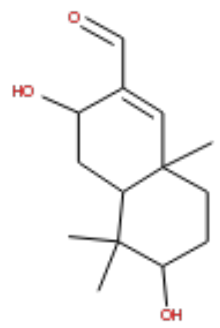

Cell ID	Cluster Center	Number of Compounds
323	0	6

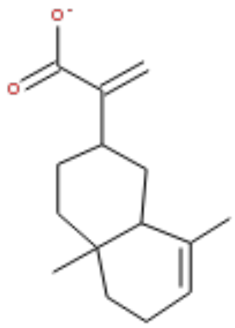

Cell ID	Cluster Center	Number of Compounds
324	1	8

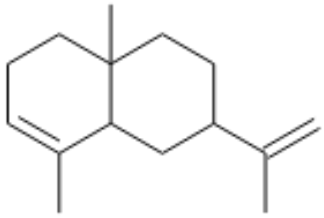

Cell ID	Cluster Center	Number of Compounds
324	0	8

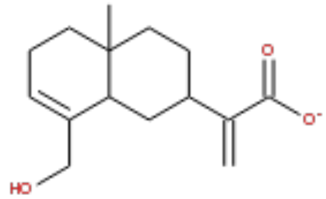

Cell ID	Cluster Center	Number of Compounds
324	0	8

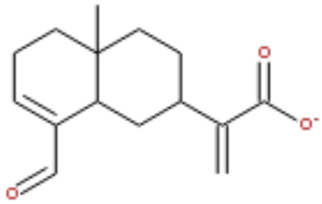

Cell ID	Cluster Center	Number of Compounds
324	0	8

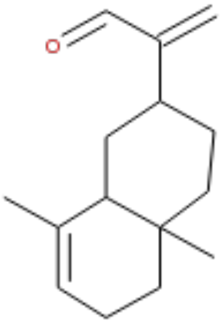

Cell ID	Cluster Center	Number of Compounds
324	0	8

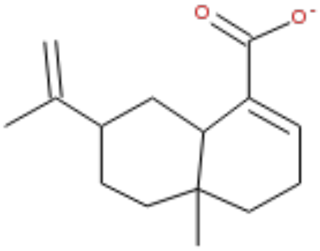

Cell ID	Cluster Center	Number of Compounds
324	0	8

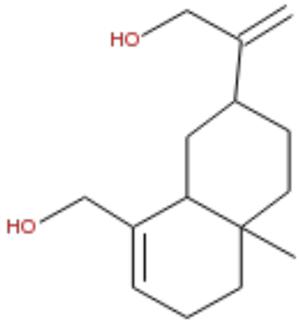

Cell ID	Cluster Center	Number of Compounds
324	0	8

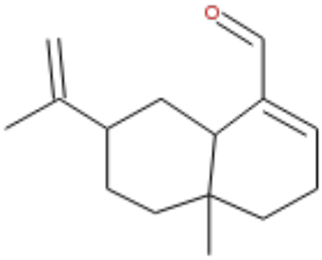

Cell ID	Cluster Center	Number of Compounds
324	0	8

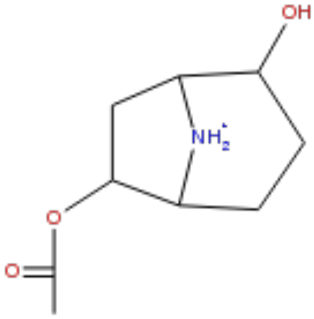

Cell ID	Cluster Center	Number of Compounds
325	1	10

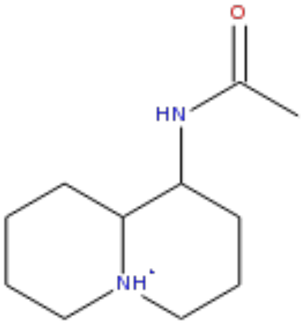

Cell ID	Cluster Center	Number of Compounds
325	0	10

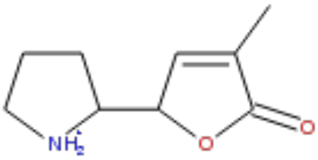

Cell ID	Cluster Center	Number of Compounds
325	0	10

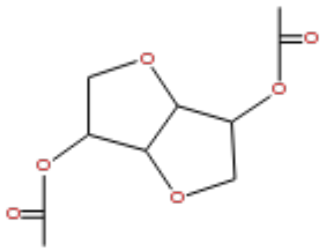

Cell ID	Cluster Center	Number of Compounds
325	0	10

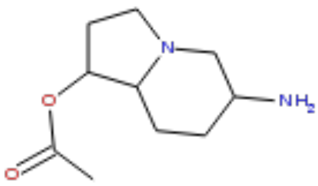

Cell ID	Cluster Center	Number of Compounds
325	0	10

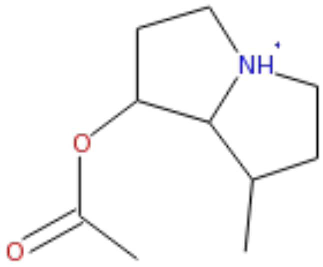

Cell ID	Cluster Center	Number of Compounds
325	0	10

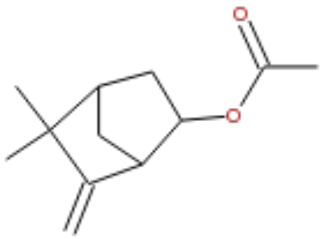

Cell ID	Cluster Center	Number of Compounds
325	0	10

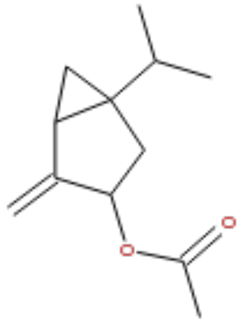

Cell ID	Cluster Center	Number of Compounds
325	0	10

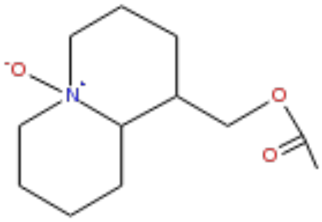

Cell ID	Cluster Center	Number of Compounds
325	0	10

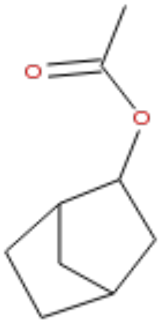

Cell ID	Cluster Center	Number of Compounds
325	0	10

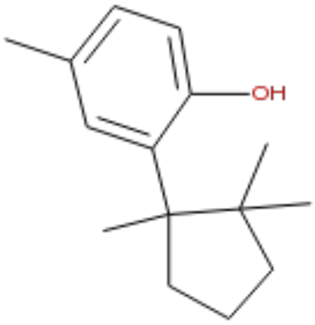

Cell ID	Cluster Center	Number of Compounds
326	1	23

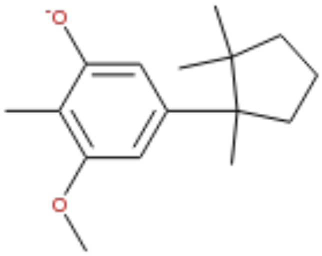

Cell ID	Cluster Center	Number of Compounds
326	0	23

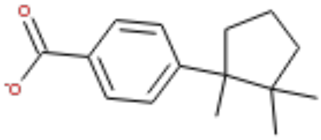

Cell ID	Cluster Center	Number of Compounds
326	0	23

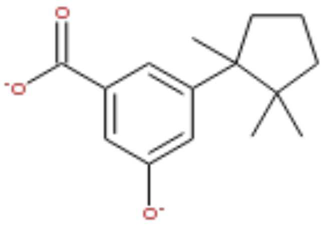

Cell ID	Cluster Center	Number of Compounds
326	0	23

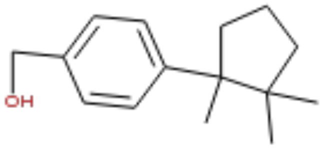

Cell ID	Cluster Center	Number of Compounds
326	0	23

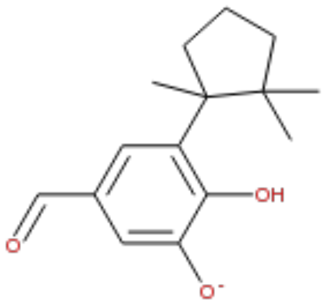

Cell ID	Cluster Center	Number of Compounds
326	0	23

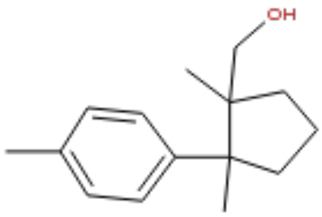

Cell ID	Cluster Center	Number of Compounds
326	0	23

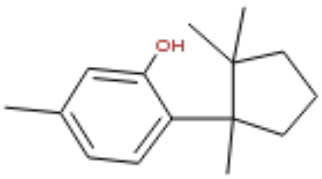

Cell ID	Cluster Center	Number of Compounds
326	0	23

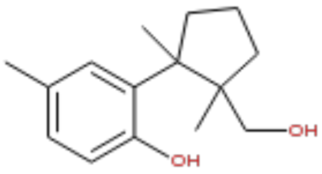

Cell ID	Cluster Center	Number of Compounds
326	0	23

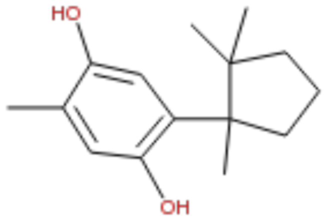

Cell ID	Cluster Center	Number of Compounds
326	0	23

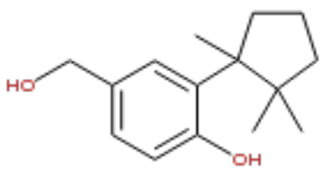

Cell ID	Cluster Center	Number of Compounds
326	0	23

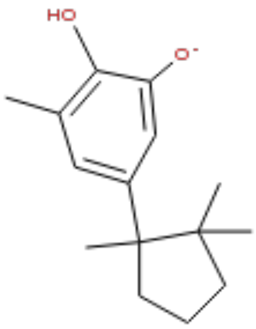

Cell ID	Cluster Center	Number of Compounds
326	0	23

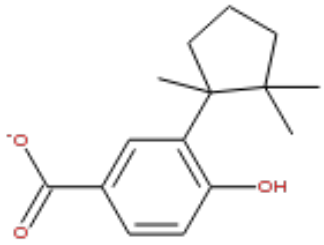

Cell ID	Cluster Center	Number of Compounds
326	0	23

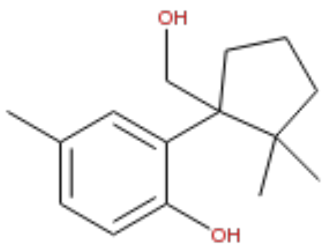

Cell ID	Cluster Center	Number of Compounds
326	0	23

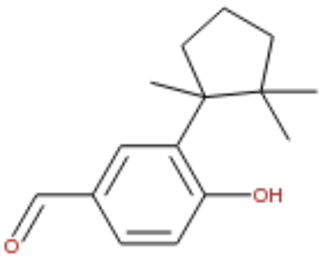

Cell ID	Cluster Center	Number of Compounds
326	0	23

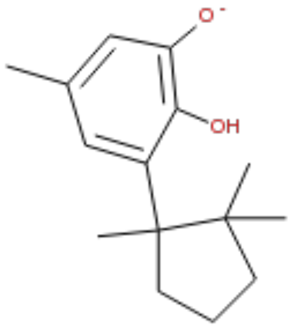

Cell ID	Cluster Center	Number of Compounds
326	0	23

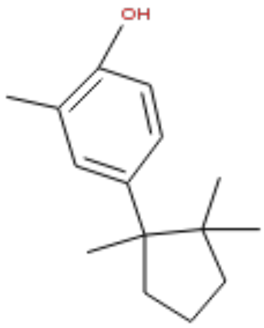

Cell ID	Cluster Center	Number of Compounds
326	0	23

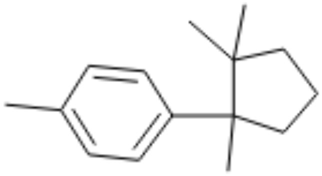

Cell ID	Cluster Center	Number of Compounds
326	0	23

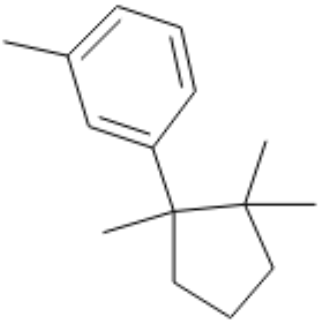

Cell ID	Cluster Center	Number of Compounds
326	0	23

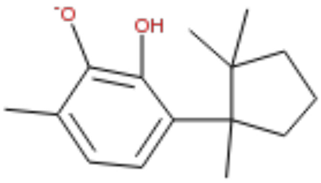

Cell ID	Cluster Center	Number of Compounds
326	0	23

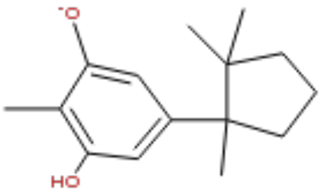

Cell ID	Cluster Center	Number of Compounds
326	0	23

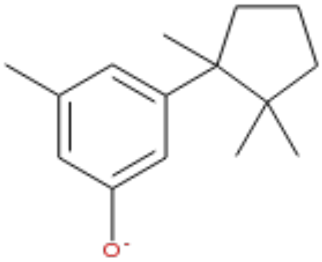

Cell ID	Cluster Center	Number of Compounds
326	0	23

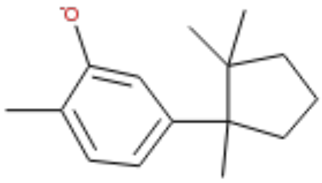

Cell ID	Cluster Center	Number of Compounds
326	0	23

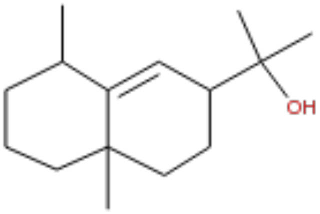

Cell ID	Cluster Center	Number of Compounds
327	1	7

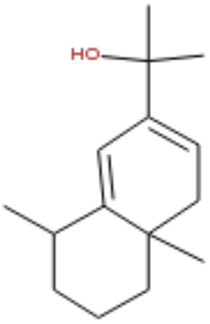

Cell ID	Cluster Center	Number of Compounds
327	0	7

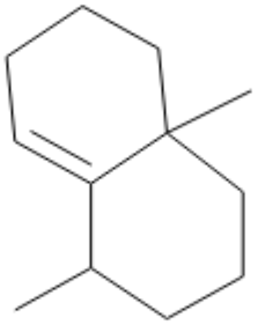

Cell ID	Cluster Center	Number of Compounds
327	0	7

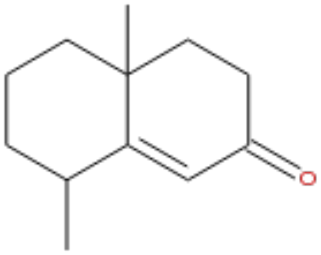

Cell ID	Cluster Center	Number of Compounds
327	0	7

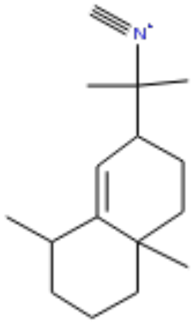

Cell ID	Cluster Center	Number of Compounds
327	0	7

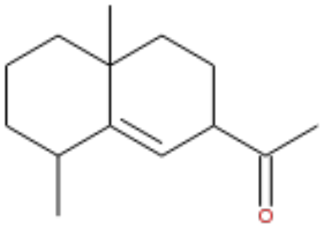

Cell ID	Cluster Center	Number of Compounds
327	0	7

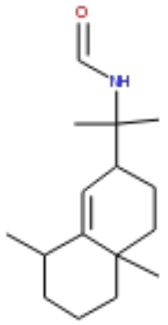

Cell ID	Cluster Center	Number of Compounds
327	0	7

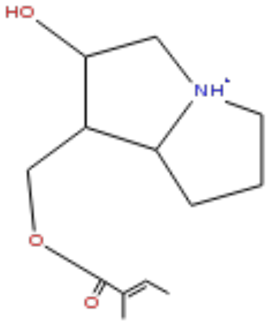

Cell ID	Cluster Center	Number of Compounds
328	1	5

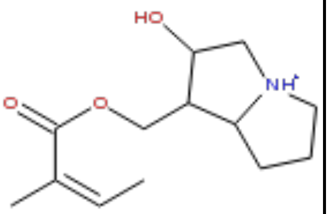

Cell ID	Cluster Center	Number of Compounds
328	0	5

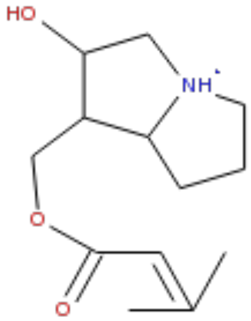

Cell ID	Cluster Center	Number of Compounds
328	0	5

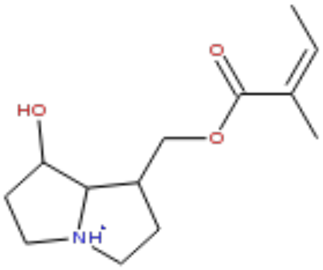

Cell ID	Cluster Center	Number of Compounds
328	0	5

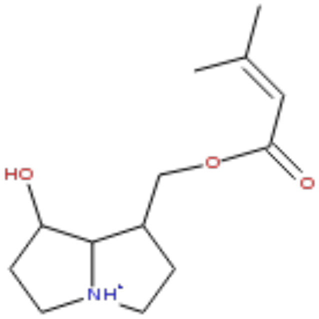

Cell ID	Cluster Center	Number of Compounds
328	0	5

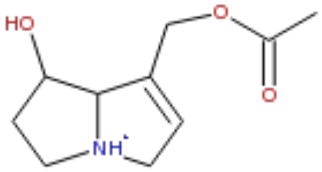

Cell ID	Cluster Center	Number of Compounds
329	1	8

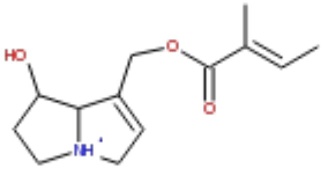

Cell ID	Cluster Center	Number of Compounds
329	0	8

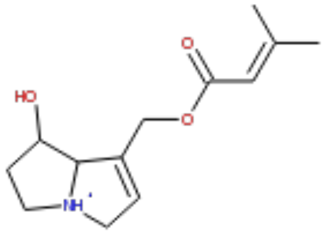

Cell ID	Cluster Center	Number of Compounds
329	0	8

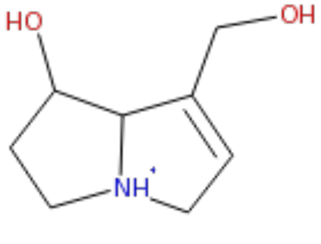

Cell ID	Cluster Center	Number of Compounds
329	0	8

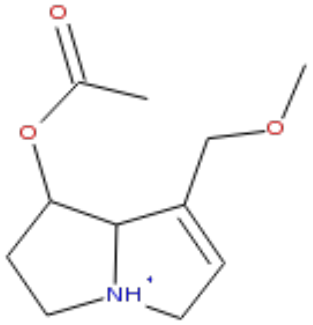

Cell ID	Cluster Center	Number of Compounds
329	0	8

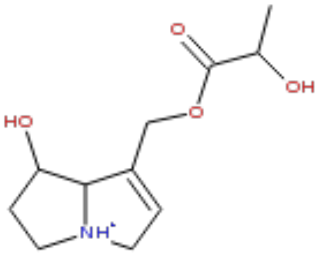

Cell ID	Cluster Center	Number of Compounds
329	0	8

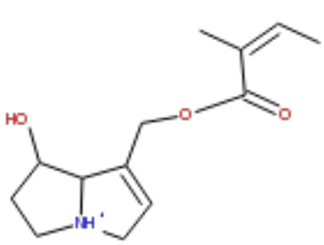

Cell ID	Cluster Center	Number of Compounds
329	0	8

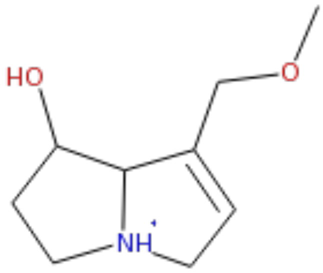

Cell ID	Cluster Center	Number of Compounds
329	0	8

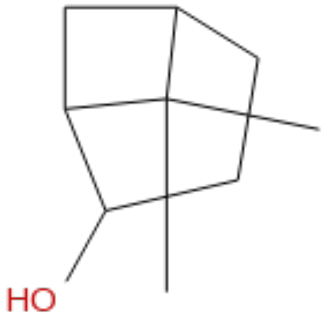

Cell ID	Cluster Center	Number of Compounds
330	1	52

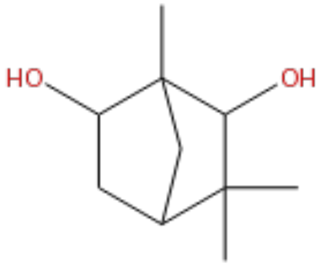

Cell ID	Cluster Center	Number of Compounds
330	0	52

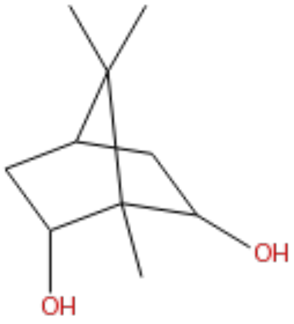

Cell ID	Cluster Center	Number of Compounds
330	0	52

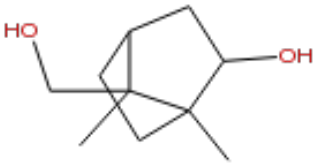

Cell ID	Cluster Center	Number of Compounds
330	0	52

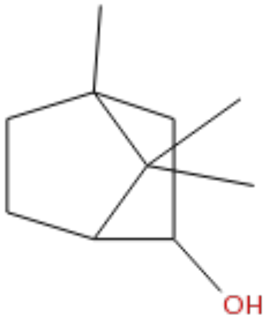

Cell ID	Cluster Center	Number of Compounds
330	0	52

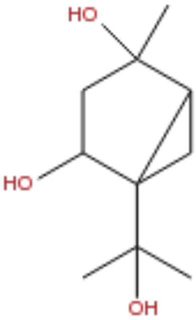

Cell ID	Cluster Center	Number of Compounds
330	0	52

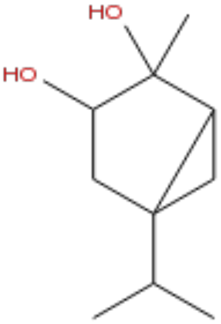

Cell ID	Cluster Center	Number of Compounds
330	0	52

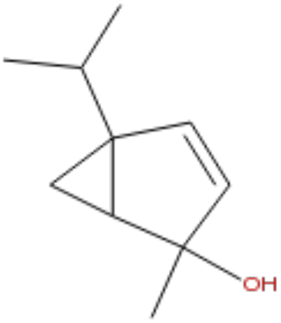

Cell ID	Cluster Center	Number of Compounds
330	0	52

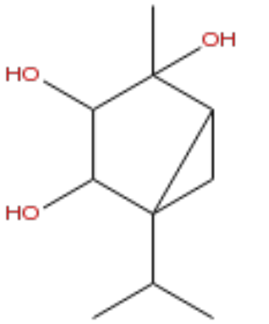

Cell ID	Cluster Center	Number of Compounds
330	0	52

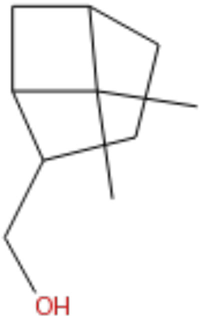

Cell ID	Cluster Center	Number of Compounds
330	0	52

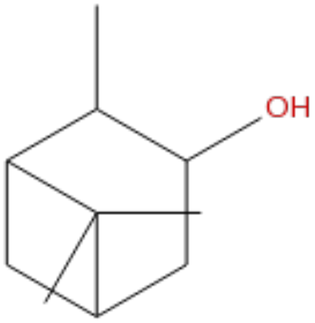

Cell ID	Cluster Center	Number of Compounds
330	0	52

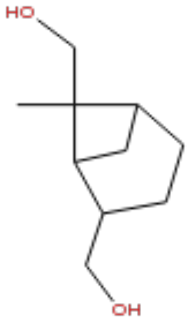

Cell ID	Cluster Center	Number of Compounds
330	0	52

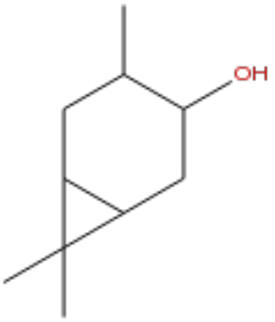

Cell ID	Cluster Center	Number of Compounds
330	0	52

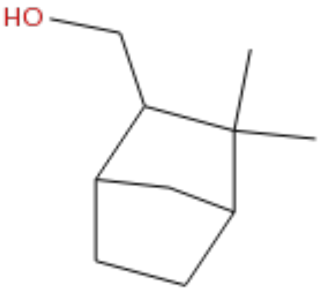

Cell ID	Cluster Center	Number of Compounds
330	0	52

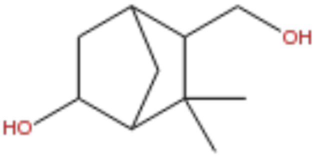

Cell ID	Cluster Center	Number of Compounds
330	0	52

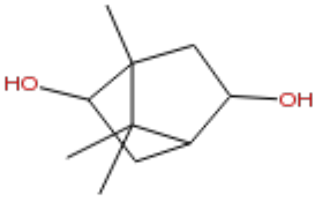

Cell ID	Cluster Center	Number of Compounds
330	0	52

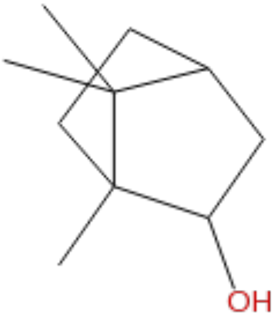

Cell ID	Cluster Center	Number of Compounds
330	0	52

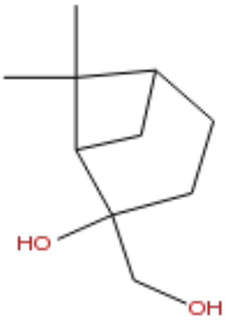

Cell ID	Cluster Center	Number of Compounds
330	0	52

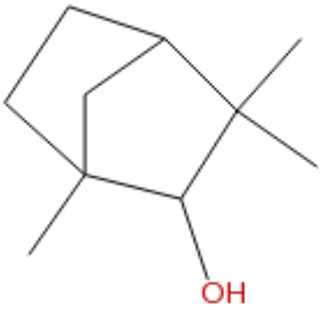

Cell ID	Cluster Center	Number of Compounds
330	0	52

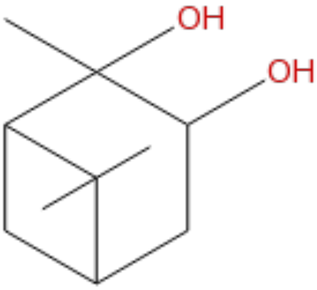

Cell ID	Cluster Center	Number of Compounds
330	0	52

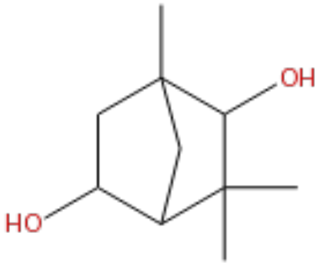

Cell ID	Cluster Center	Number of Compounds
330	0	52

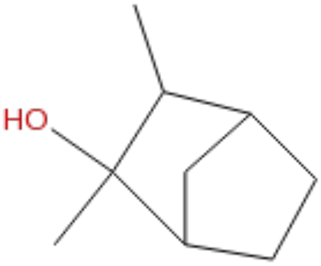

Cell ID	Cluster Center	Number of Compounds
330	0	52

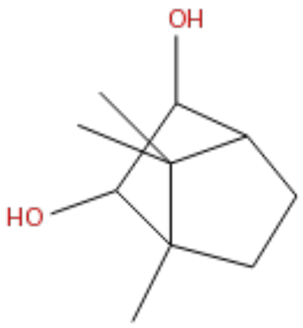

Cell ID	Cluster Center	Number of Compounds
330	0	52

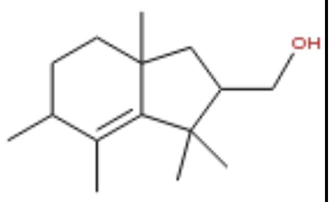

Cell ID	Cluster Center	Number of Compounds
330	0	52

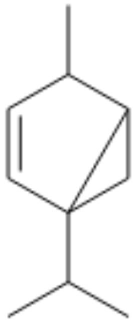

Cell ID	Cluster Center	Number of Compounds
330	0	52

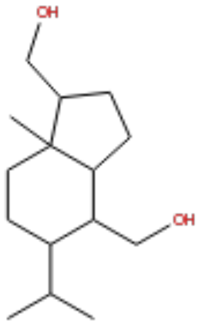

Cell ID	Cluster Center	Number of Compounds
330	0	52

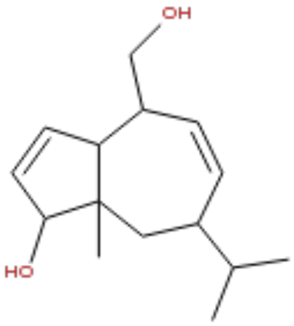

Cell ID	Cluster Center	Number of Compounds
330	0	52

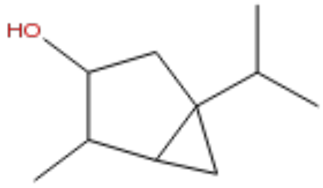

Cell ID	Cluster Center	Number of Compounds
330	0	52

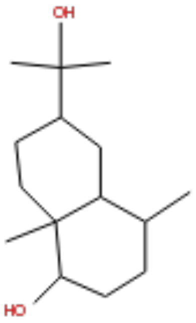

Cell ID	Cluster Center	Number of Compounds
330	0	52

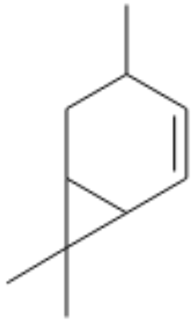

Cell ID	Cluster Center	Number of Compounds
330	0	52

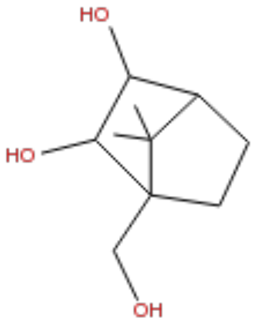

Cell ID	Cluster Center	Number of Compounds
330	0	52

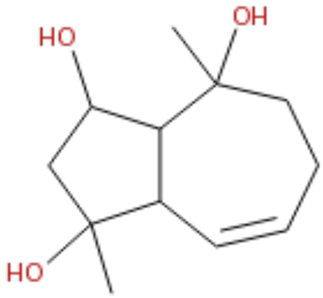

Cell ID	Cluster Center	Number of Compounds
330	0	52

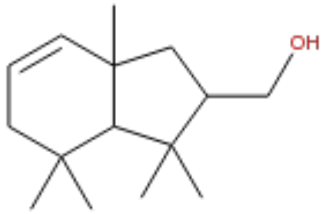

Cell ID	Cluster Center	Number of Compounds
330	0	52

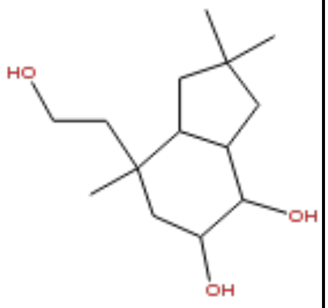

Cell ID	Cluster Center	Number of Compounds
330	0	52

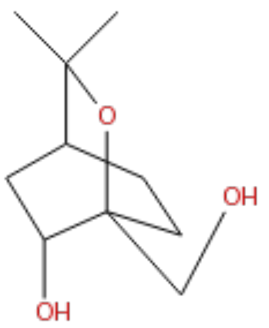

Cell ID	Cluster Center	Number of Compounds
330	0	52

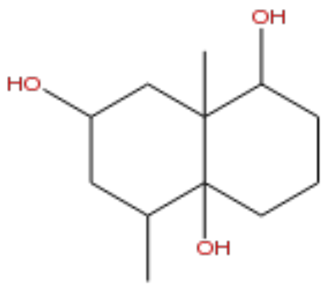

Cell ID	Cluster Center	Number of Compounds
330	0	52

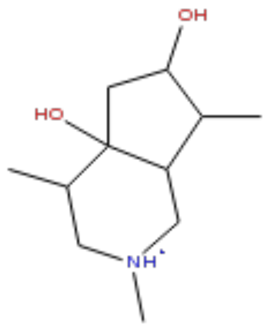

Cell ID	Cluster Center	Number of Compounds
330	0	52

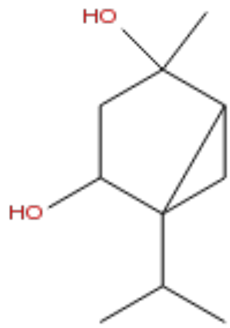

Cell ID	Cluster Center	Number of Compounds
330	0	52

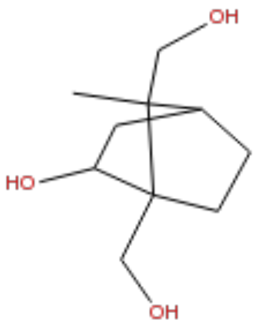

Cell ID	Cluster Center	Number of Compounds
330	0	52

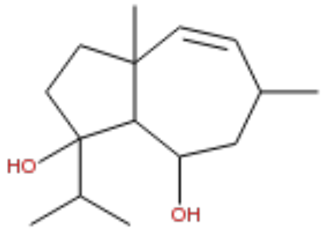

Cell ID	Cluster Center	Number of Compounds
330	0	52

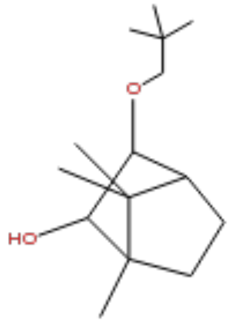

Cell ID	Cluster Center	Number of Compounds
330	0	52

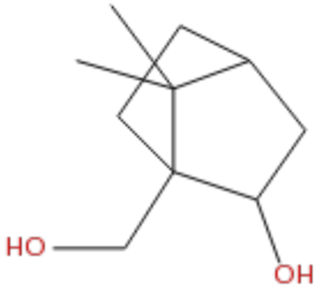

Cell ID	Cluster Center	Number of Compounds
330	0	52

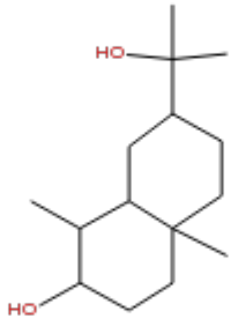

Cell ID	Cluster Center	Number of Compounds
330	0	52

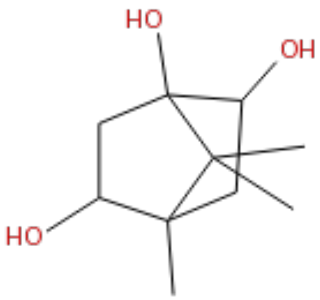

Cell ID	Cluster Center	Number of Compounds
330	0	52

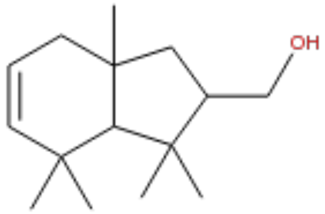

Cell ID	Cluster Center	Number of Compounds
330	0	52

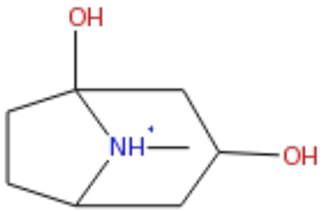

Cell ID	Cluster Center	Number of Compounds
330	0	52

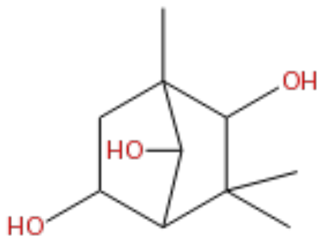

Cell ID	Cluster Center	Number of Compounds
330	0	52

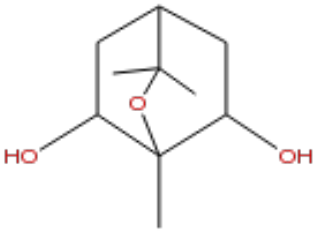

Cell ID	Cluster Center	Number of Compounds
330	0	52

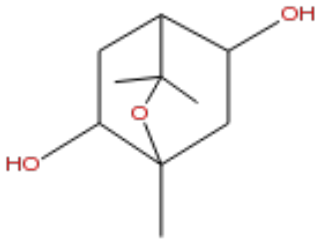

Cell ID	Cluster Center	Number of Compounds
330	0	52

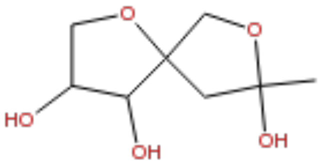

Cell ID	Cluster Center	Number of Compounds
330	0	52

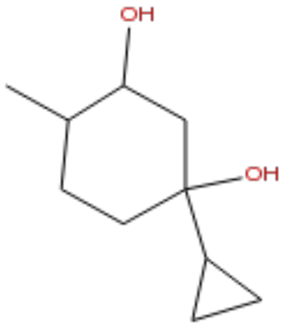

Cell ID	Cluster Center	Number of Compounds
330	0	52

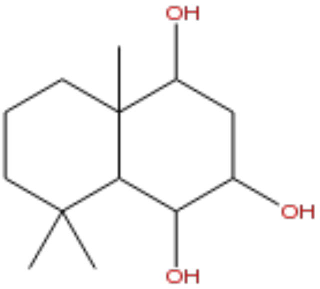

Cell ID	Cluster Center	Number of Compounds
330	0	52

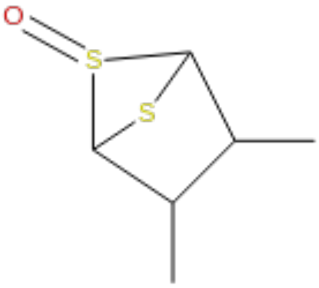

Cell ID	Cluster Center	Number of Compounds
332	1	4

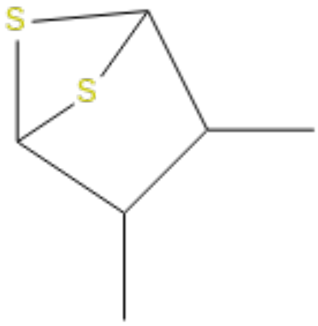

Cell ID	Cluster Center	Number of Compounds
332	0	4

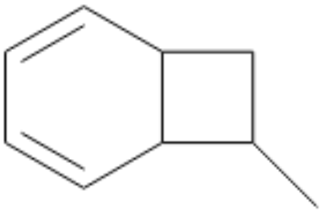

Cell ID	Cluster Center	Number of Compounds
332	0	4

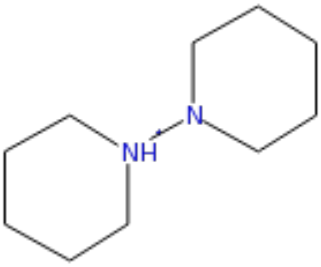

Cell ID	Cluster Center	Number of Compounds
332	0	4

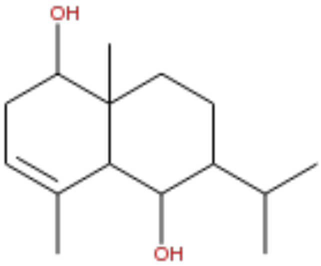

Cell ID	Cluster Center	Number of Compounds
333	1	2

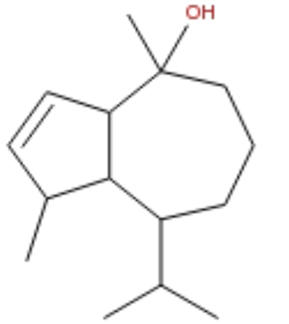

Cell ID	Cluster Center	Number of Compounds
333	0	2

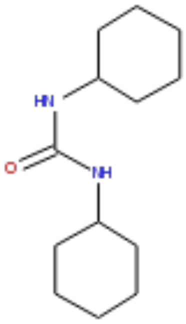

Cell ID	Cluster Center	Number of Compounds
335	1	1

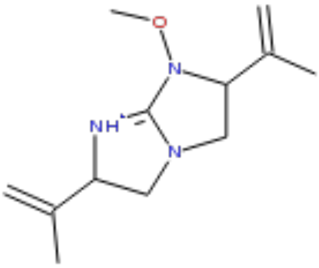

Cell ID	Cluster Center	Number of Compounds
336	1	3

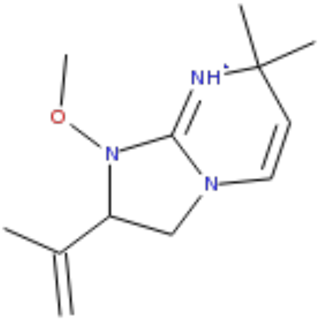

Cell ID	Cluster Center	Number of Compounds
336	0	3

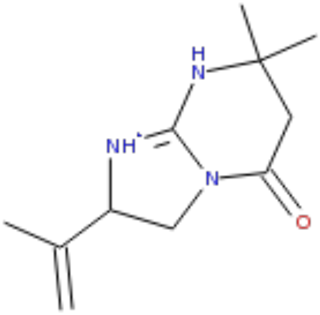

Cell ID	Cluster Center	Number of Compounds
336	0	3

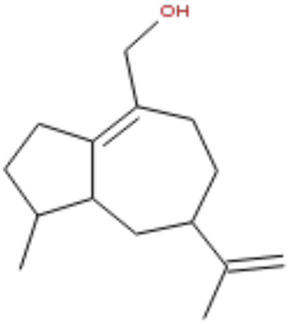

Cell ID	Cluster Center	Number of Compounds
337	1	8

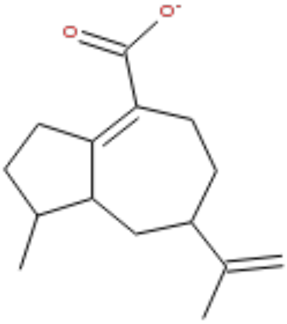

Cell ID	Cluster Center	Number of Compounds
337	0	8

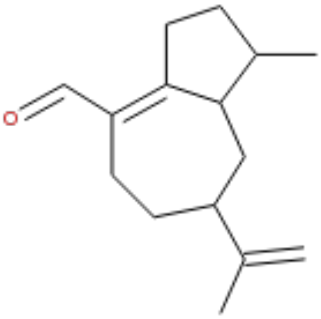

Cell ID	Cluster Center	Number of Compounds
337	0	8

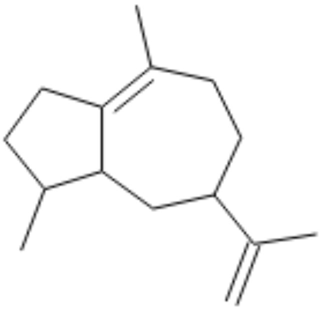

Cell ID	Cluster Center	Number of Compounds
337	0	8

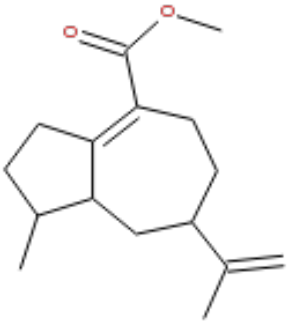

Cell ID	Cluster Center	Number of Compounds
337	0	8

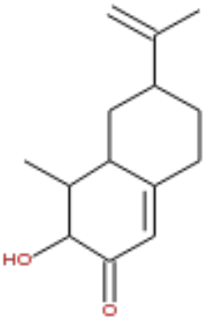

Cell ID	Cluster Center	Number of Compounds
337	0	8

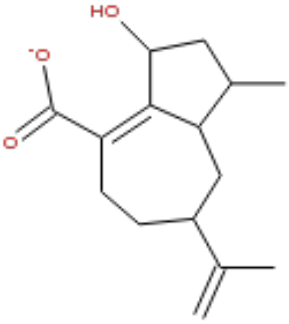

Cell ID	Cluster Center	Number of Compounds
337	0	8

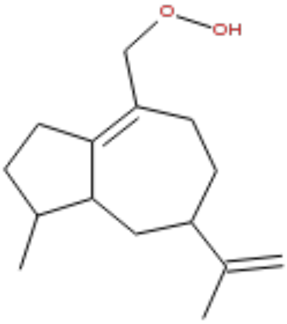

Cell ID	Cluster Center	Number of Compounds
337	0	8

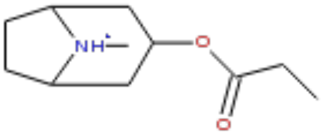

Cell ID	Cluster Center	Number of Compounds
338	1	8

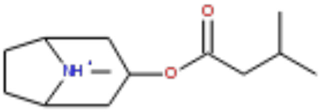

Cell ID	Cluster Center	Number of Compounds
338	0	8

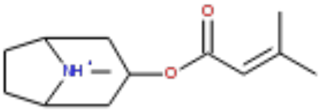

Cell ID	Cluster Center	Number of Compounds
338	0	8

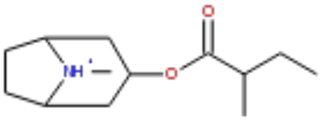

Cell ID	Cluster Center	Number of Compounds
338	0	8

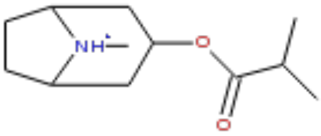

Cell ID	Cluster Center	Number of Compounds
338	0	8

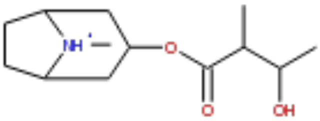

Cell ID	Cluster Center	Number of Compounds
338	0	8

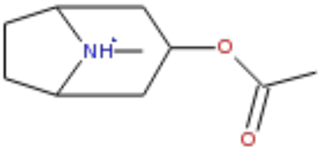

Cell ID	Cluster Center	Number of Compounds
338	0	8

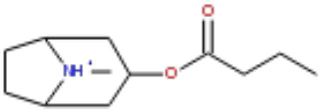

Cell ID	Cluster Center	Number of Compounds
338	0	8

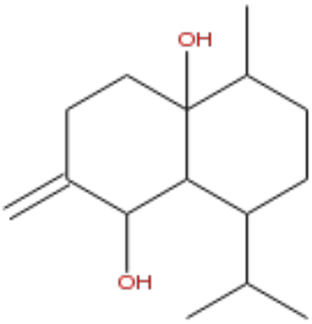

Cell ID	Cluster Center	Number of Compounds
339	1	2

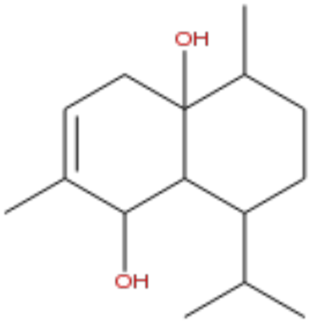

Cell ID	Cluster Center	Number of Compounds
339	0	2

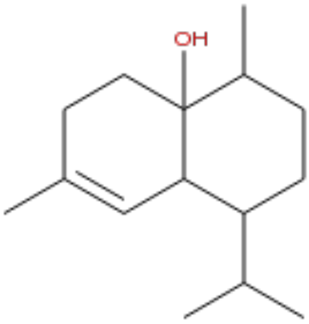

Cell ID	Cluster Center	Number of Compounds
341	1	11

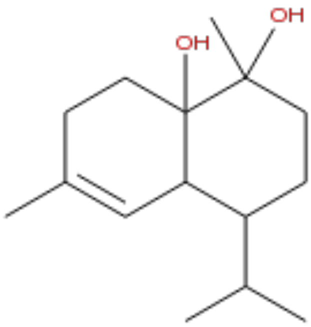

Cell ID	Cluster Center	Number of Compounds
341	0	11

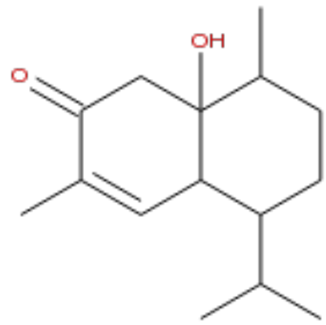

Cell ID	Cluster Center	Number of Compounds
341	0	11

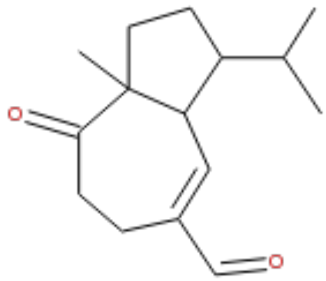

Cell ID	Cluster Center	Number of Compounds
341	0	11

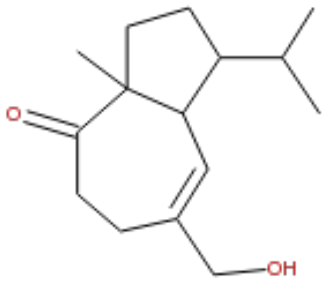

Cell ID	Cluster Center	Number of Compounds
341	0	11

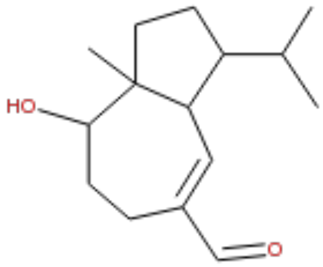

Cell ID	Cluster Center	Number of Compounds
341	0	11

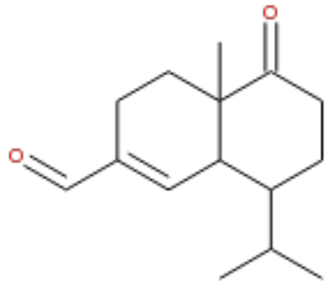

Cell ID	Cluster Center	Number of Compounds
341	0	11

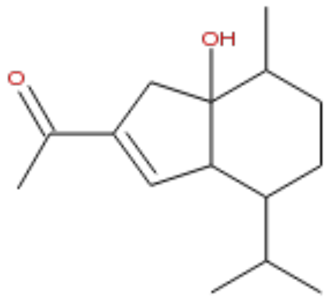

Cell ID	Cluster Center	Number of Compounds
341	0	11

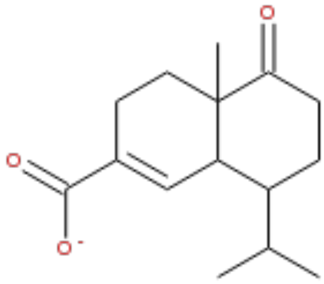

Cell ID	Cluster Center	Number of Compounds
341	0	11

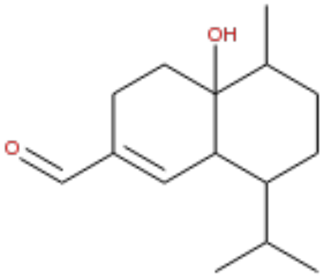

Cell ID	Cluster Center	Number of Compounds
341	0	11

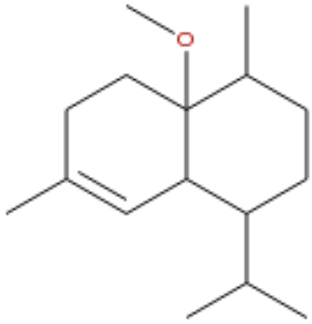

Cell ID	Cluster Center	Number of Compounds
341	0	11

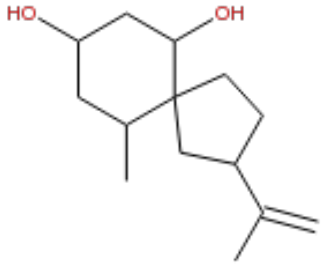

Cell ID	Cluster Center	Number of Compounds
342	1	12

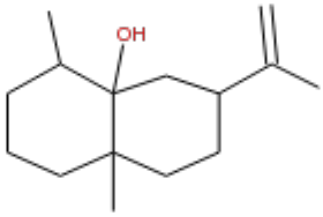

Cell ID	Cluster Center	Number of Compounds
342	0	12

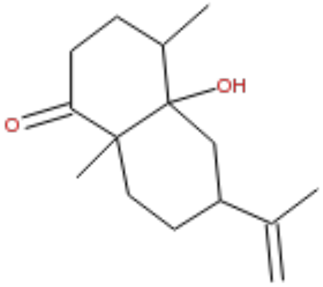

Cell ID	Cluster Center	Number of Compounds
342	0	12

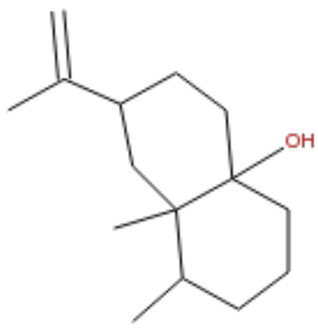

Cell ID	Cluster Center	Number of Compounds
342	0	12

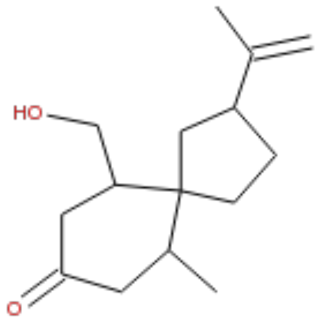

Cell ID	Cluster Center	Number of Compounds
342	0	12

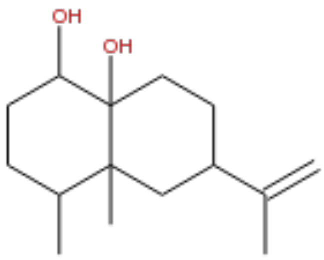

Cell ID	Cluster Center	Number of Compounds
342	0	12

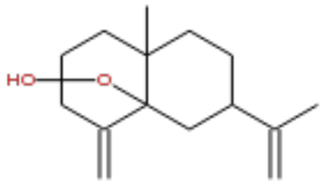

Cell ID	Cluster Center	Number of Compounds
342	0	12

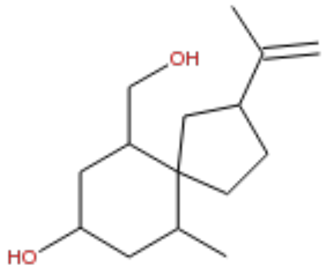

Cell ID	Cluster Center	Number of Compounds
342	0	12

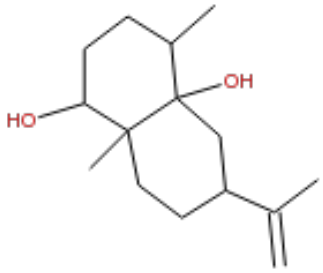

Cell ID	Cluster Center	Number of Compounds
342	0	12

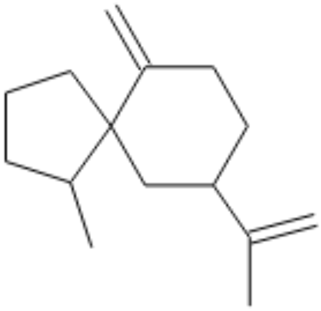

Cell ID	Cluster Center	Number of Compounds
342	0	12

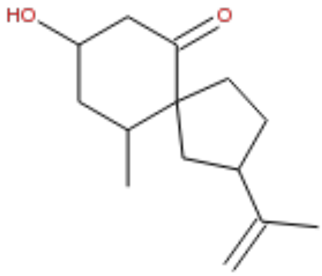

Cell ID	Cluster Center	Number of Compounds
342	0	12

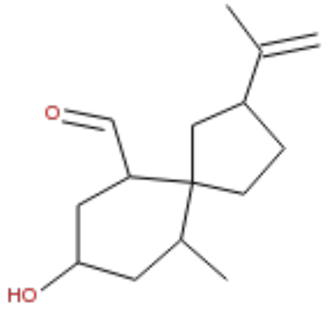

Cell ID	Cluster Center	Number of Compounds
342	0	12

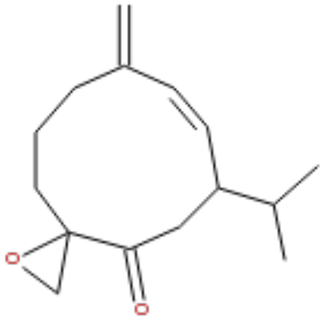

Cell ID	Cluster Center	Number of Compounds
344	1	2

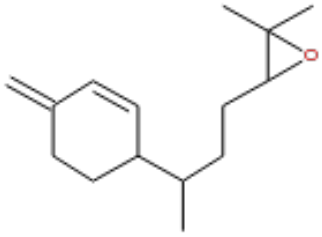

Cell ID	Cluster Center	Number of Compounds
344	0	2

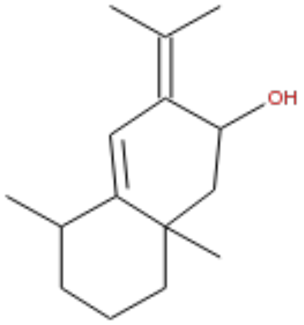

Cell ID	Cluster Center	Number of Compounds
345	1	3

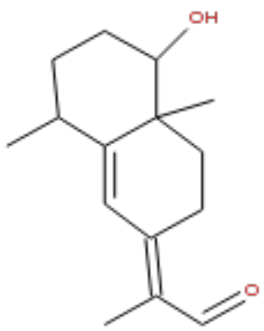

Cell ID	Cluster Center	Number of Compounds
345	0	3

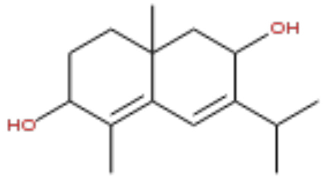

Cell ID	Cluster Center	Number of Compounds
345	0	3

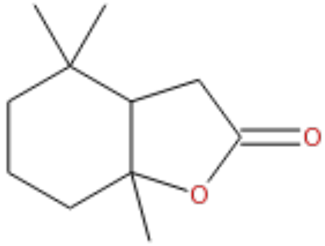

Cell ID	Cluster Center	Number of Compounds
347	1	15

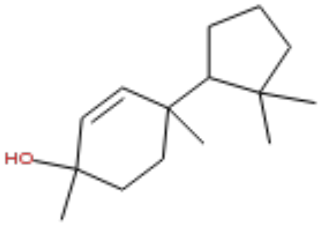

Cell ID	Cluster Center	Number of Compounds
347	0	15

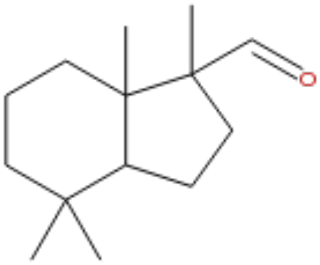

Cell ID	Cluster Center	Number of Compounds
347	0	15

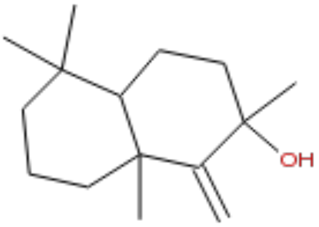

Cell ID	Cluster Center	Number of Compounds
347	0	15

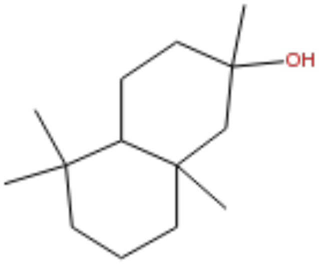

Cell ID	Cluster Center	Number of Compounds
347	0	15

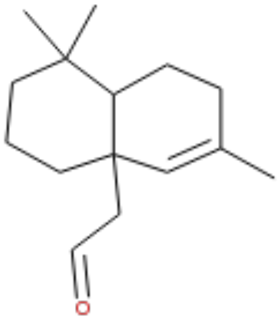

Cell ID	Cluster Center	Number of Compounds
347	0	15

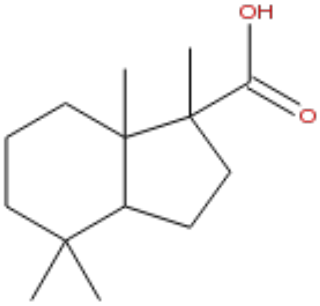

Cell ID	Cluster Center	Number of Compounds
347	0	15

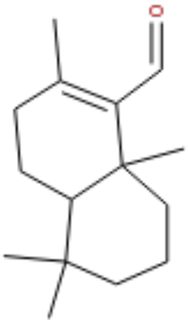

Cell ID	Cluster Center	Number of Compounds
347	0	15

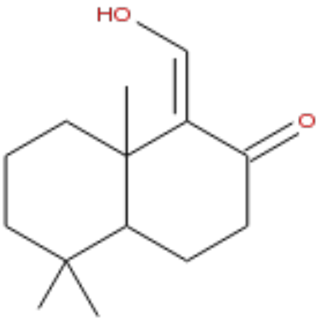

Cell ID	Cluster Center	Number of Compounds
347	0	15

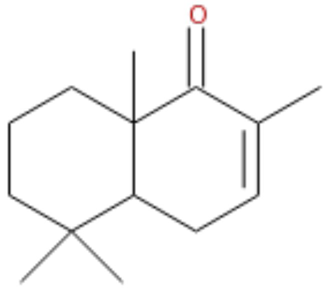

Cell ID	Cluster Center	Number of Compounds
347	0	15

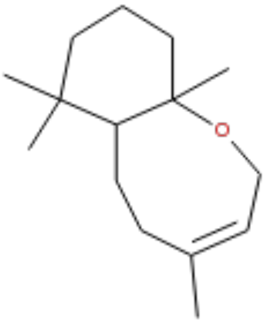

Cell ID	Cluster Center	Number of Compounds
347	0	15

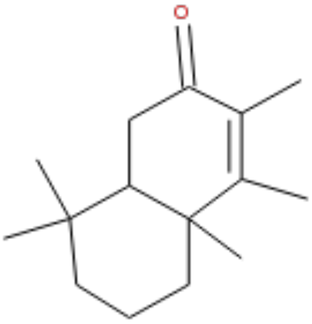

Cell ID	Cluster Center	Number of Compounds
347	0	15

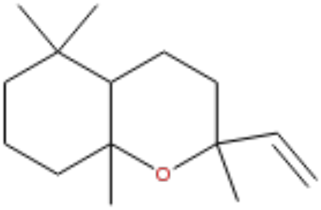

Cell ID	Cluster Center	Number of Compounds
347	0	15

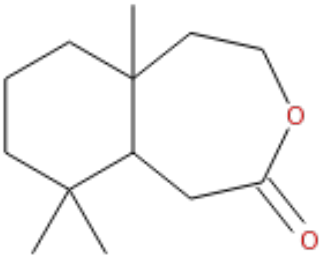

Cell ID	Cluster Center	Number of Compounds
347	0	15

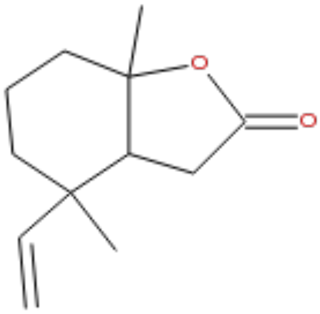

Cell ID	Cluster Center	Number of Compounds
347	0	15

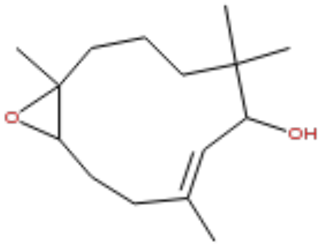

Cell ID	Cluster Center	Number of Compounds
348	1	1

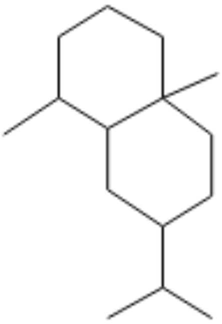

Cell ID	Cluster Center	Number of Compounds
349	1	1

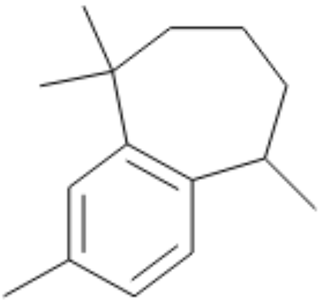

Cell ID	Cluster Center	Number of Compounds
351	1	9

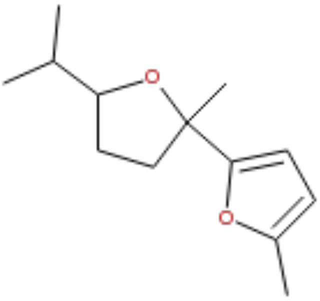

Cell ID	Cluster Center	Number of Compounds
351	0	9

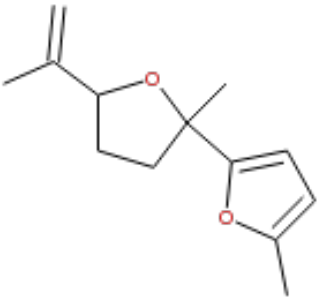

Cell ID	Cluster Center	Number of Compounds
351	0	9

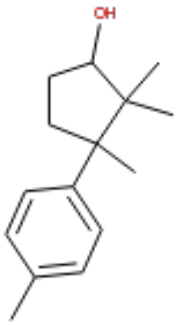

Cell ID	Cluster Center	Number of Compounds
351	0	9

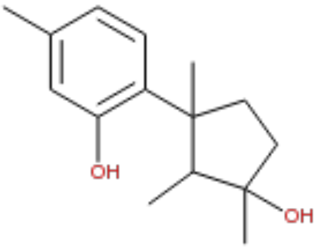

Cell ID	Cluster Center	Number of Compounds
351	0	9

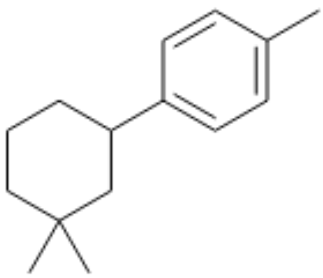

Cell ID	Cluster Center	Number of Compounds
351	0	9

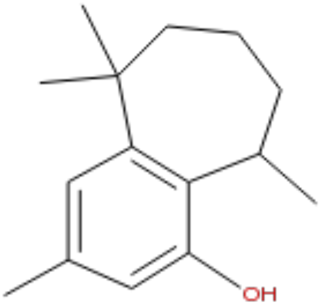

Cell ID	Cluster Center	Number of Compounds
351	0	9

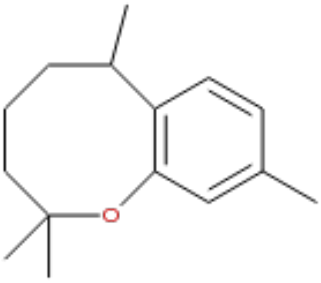

Cell ID	Cluster Center	Number of Compounds
351	0	9

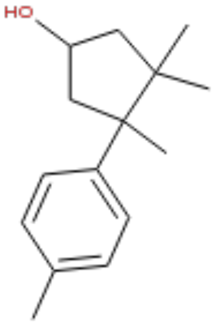

Cell ID	Cluster Center	Number of Compounds
351	0	9

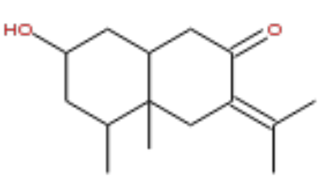

Cell ID	Cluster Center	Number of Compounds
353	1	6

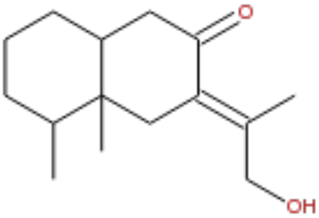

Cell ID	Cluster Center	Number of Compounds
353	0	6

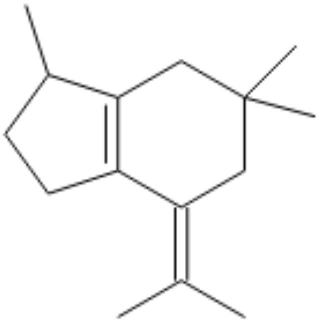

Cell ID	Cluster Center	Number of Compounds
353	0	6

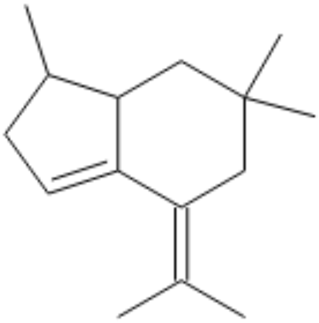

Cell ID	Cluster Center	Number of Compounds
353	0	6

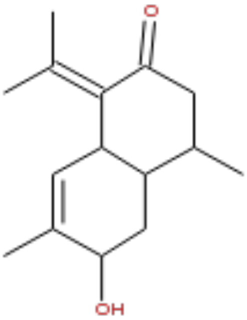

Cell ID	Cluster Center	Number of Compounds
353	0	6

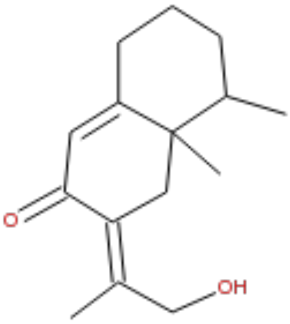

Cell ID	Cluster Center	Number of Compounds
353	0	6

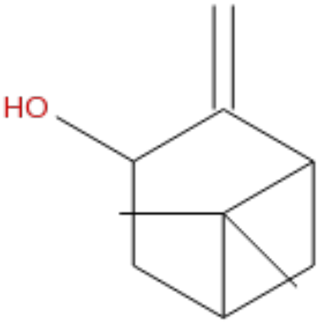

Cell ID	Cluster Center	Number of Compounds
354	1	16

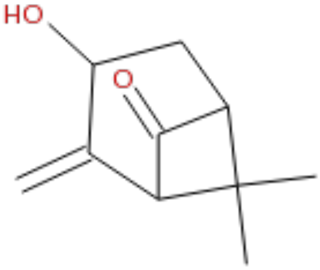

Cell ID	Cluster Center	Number of Compounds
354	0	16

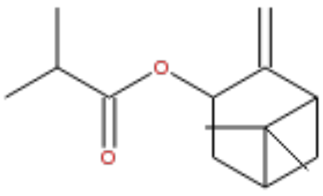

Cell ID	Cluster Center	Number of Compounds
354	0	16

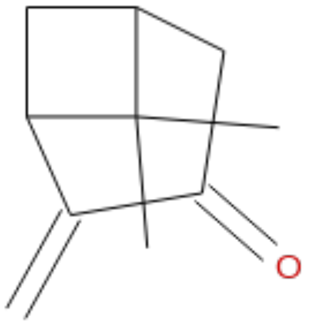

Cell ID	Cluster Center	Number of Compounds
354	0	16

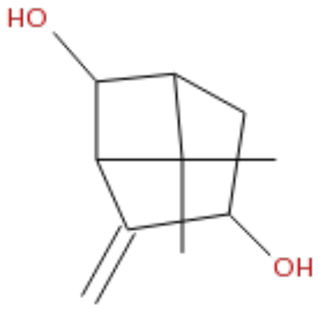

Cell ID	Cluster Center	Number of Compounds
354	0	16

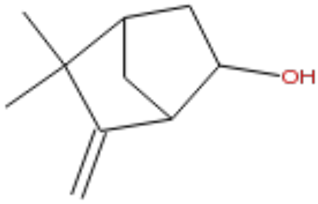

Cell ID	Cluster Center	Number of Compounds
354	0	16

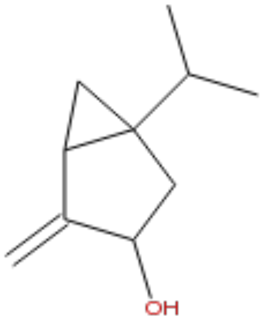

Cell ID	Cluster Center	Number of Compounds
354	0	16

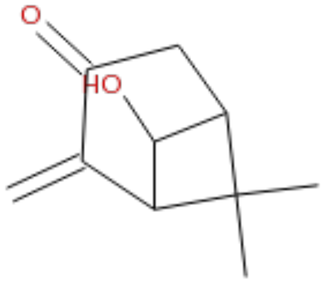

Cell ID	Cluster Center	Number of Compounds
354	0	16

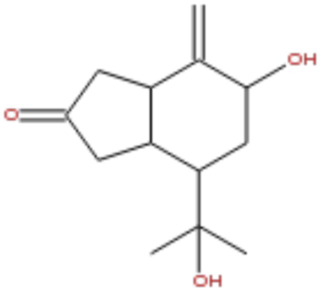

Cell ID	Cluster Center	Number of Compounds
354	0	16

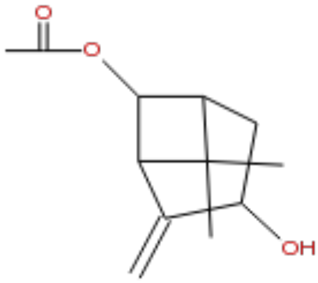

Cell ID	Cluster Center	Number of Compounds
354	0	16

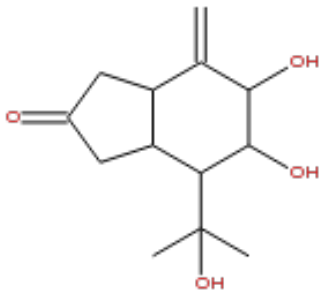

Cell ID	Cluster Center	Number of Compounds
354	0	16

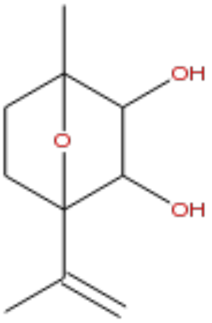

Cell ID	Cluster Center	Number of Compounds
354	0	16

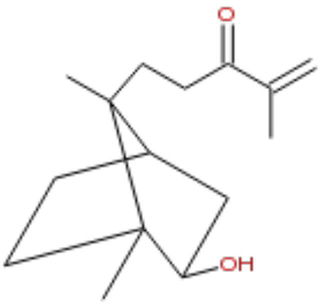

Cell ID	Cluster Center	Number of Compounds
354	0	16

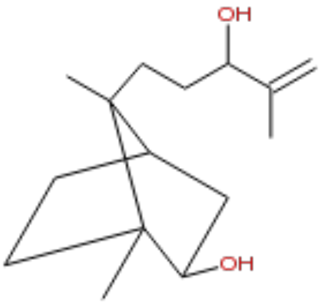

Cell ID	Cluster Center	Number of Compounds
354	0	16

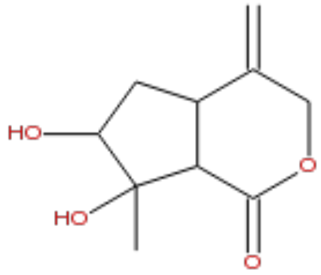

Cell ID	Cluster Center	Number of Compounds
354	0	16

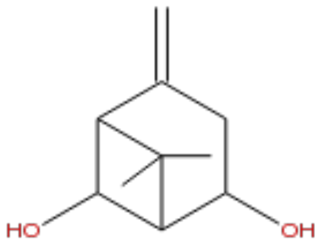

Cell ID	Cluster Center	Number of Compounds
354	0	16

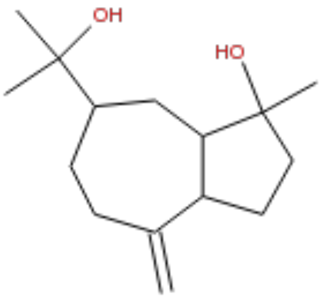

Cell ID	Cluster Center	Number of Compounds
356	1	9

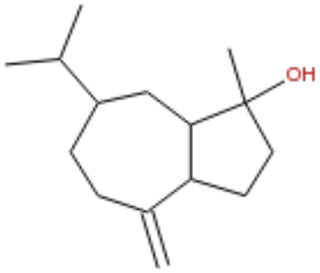

Cell ID	Cluster Center	Number of Compounds
356	0	9

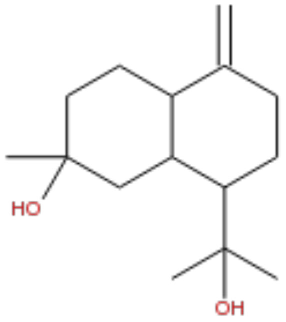

Cell ID	Cluster Center	Number of Compounds
356	0	9

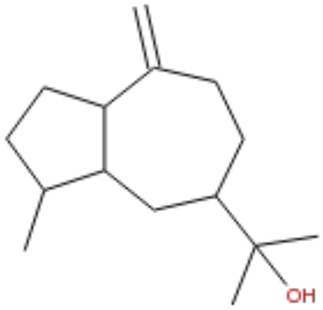

Cell ID	Cluster Center	Number of Compounds
356	0	9

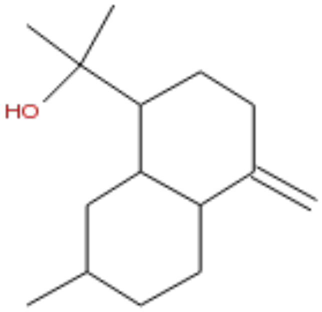

Cell ID	Cluster Center	Number of Compounds
356	0	9

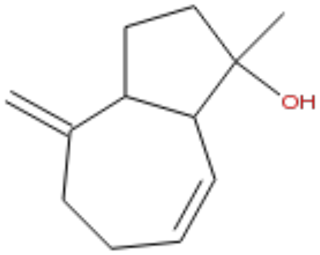

Cell ID	Cluster Center	Number of Compounds
356	0	9

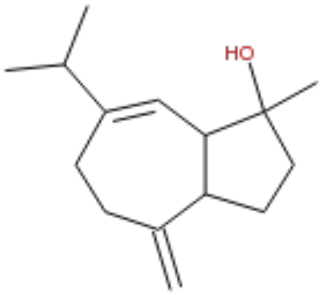

Cell ID	Cluster Center	Number of Compounds
356	0	9

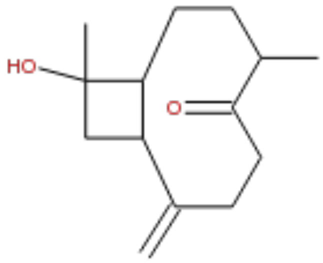

Cell ID	Cluster Center	Number of Compounds
356	0	9

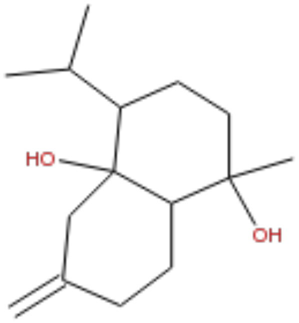

Cell ID	Cluster Center	Number of Compounds
356	0	9

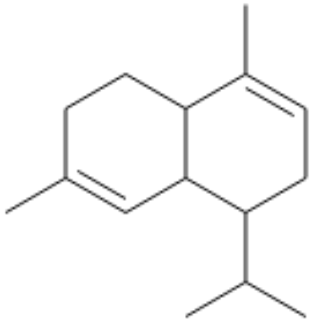

Cell ID	Cluster Center	Number of Compounds
357	1	11

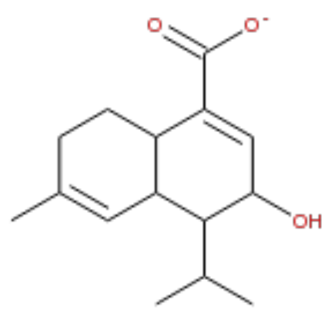

Cell ID	Cluster Center	Number of Compounds
357	0	11

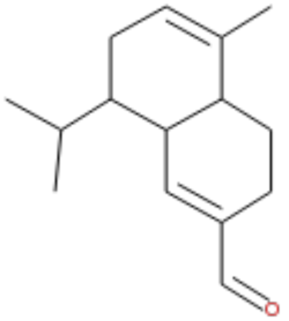

Cell ID	Cluster Center	Number of Compounds
357	0	11

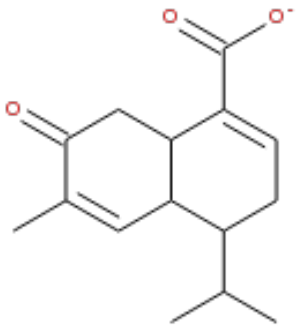

Cell ID	Cluster Center	Number of Compounds
357	0	11

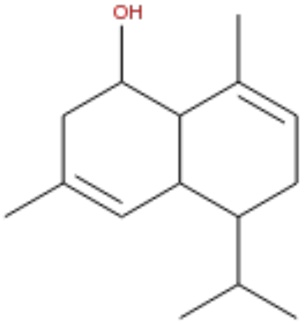

Cell ID	Cluster Center	Number of Compounds
357	0	11

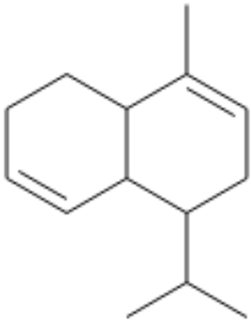

Cell ID	Cluster Center	Number of Compounds
357	0	11

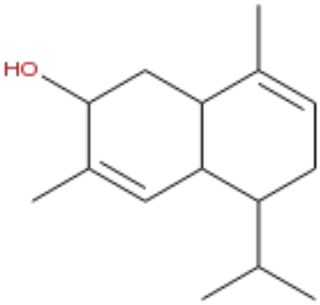

Cell ID	Cluster Center	Number of Compounds
357	0	11

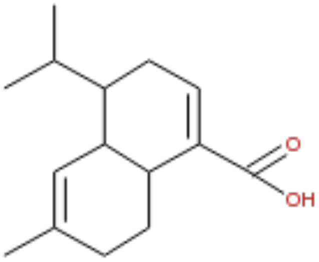

Cell ID	Cluster Center	Number of Compounds
357	0	11

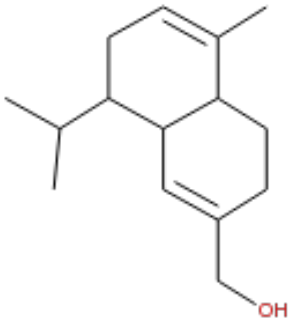

Cell ID	Cluster Center	Number of Compounds
357	0	11

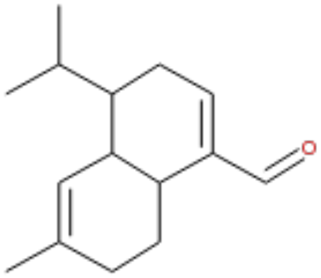

Cell ID	Cluster Center	Number of Compounds
357	0	11

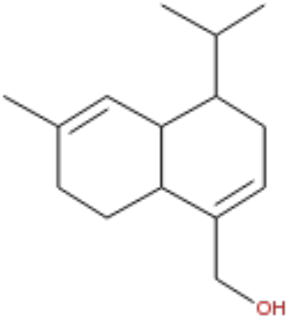

Cell ID	Cluster Center	Number of Compounds
357	0	11

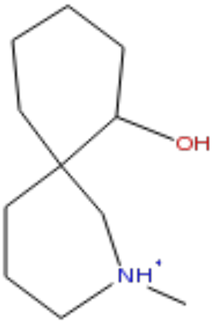

Cell ID	Cluster Center	Number of Compounds
358	1	5

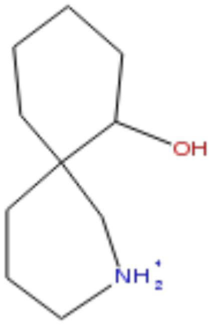

Cell ID	Cluster Center	Number of Compounds
358	0	5

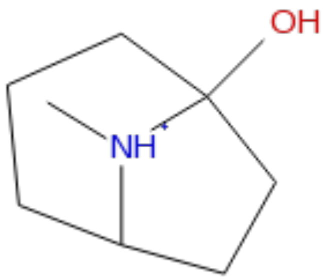

Cell ID	Cluster Center	Number of Compounds
358	0	5

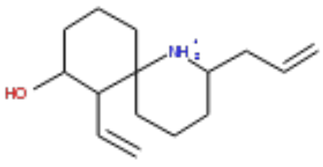

Cell ID	Cluster Center	Number of Compounds
358	0	5

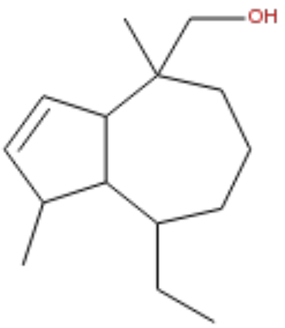

Cell ID	Cluster Center	Number of Compounds
358	0	5

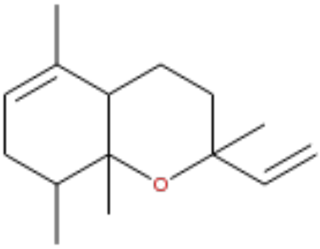

Cell ID	Cluster Center	Number of Compounds
359	1	9

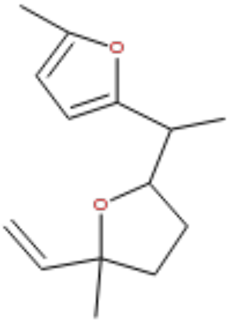

Cell ID	Cluster Center	Number of Compounds
359	0	9

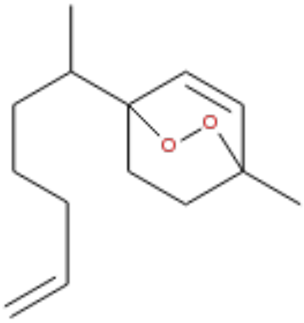

Cell ID	Cluster Center	Number of Compounds
359	0	9

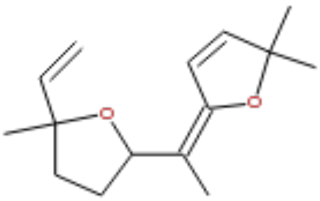

Cell ID	Cluster Center	Number of Compounds
359	0	9

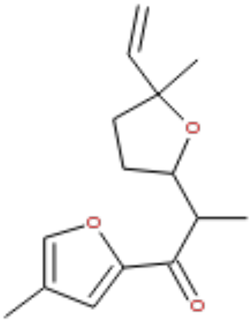

Cell ID	Cluster Center	Number of Compounds
359	0	9

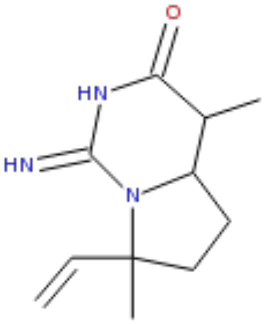

Cell ID	Cluster Center	Number of Compounds
359	0	9

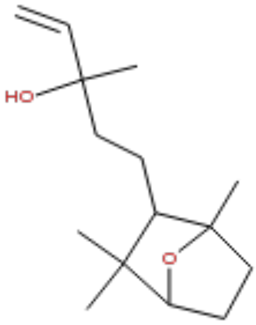

Cell ID	Cluster Center	Number of Compounds
359	0	9

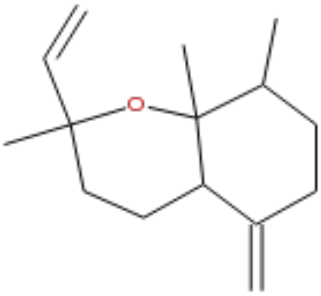

Cell ID	Cluster Center	Number of Compounds
359	0	9

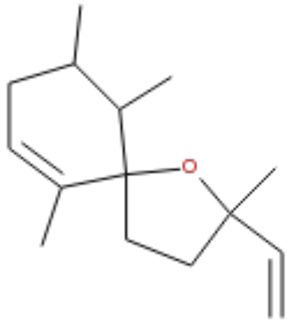

Cell ID	Cluster Center	Number of Compounds
359	0	9

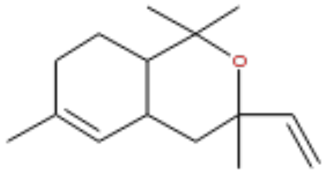

Cell ID	Cluster Center	Number of Compounds
360	1	1

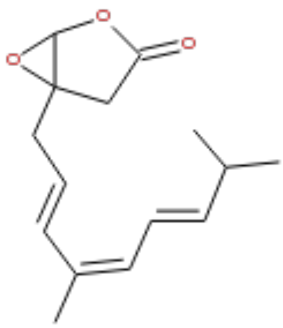

Cell ID	Cluster Center	Number of Compounds
361	1	2

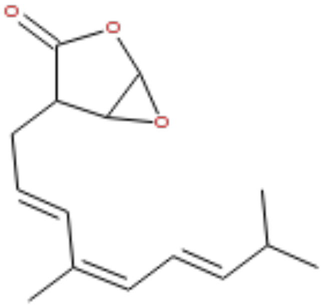

Cell ID	Cluster Center	Number of Compounds
361	0	2

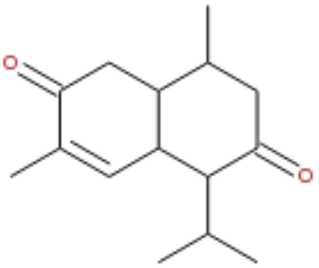

Cell ID	Cluster Center	Number of Compounds
362	1	8

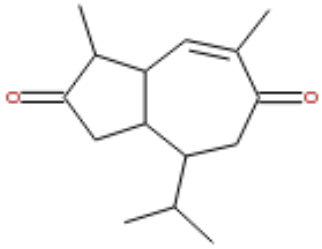

Cell ID	Cluster Center	Number of Compounds
362	0	8

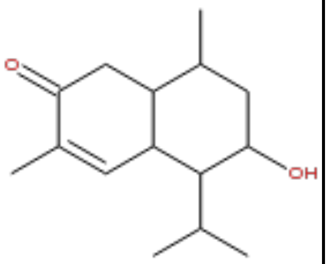

Cell ID	Cluster Center	Number of Compounds
362	0	8

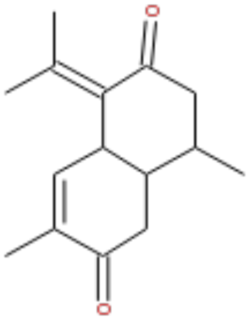

Cell ID	Cluster Center	Number of Compounds
362	0	8

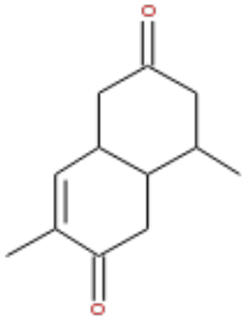

Cell ID	Cluster Center	Number of Compounds
362	0	8

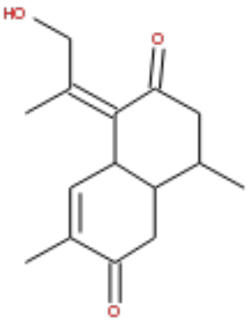

Cell ID	Cluster Center	Number of Compounds
362	0	8

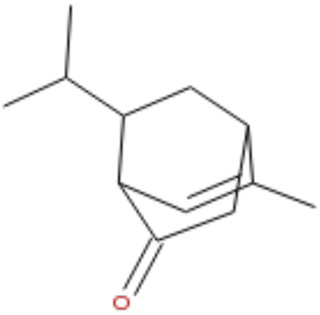

Cell ID	Cluster Center	Number of Compounds
362	0	8

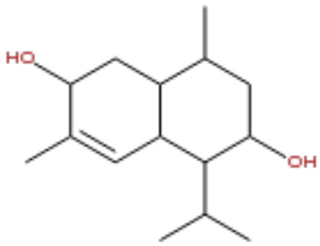

Cell ID	Cluster Center	Number of Compounds
362	0	8

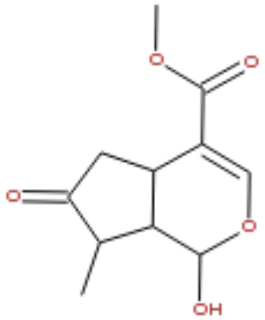

Cell ID	Cluster Center	Number of Compounds
364	1	13

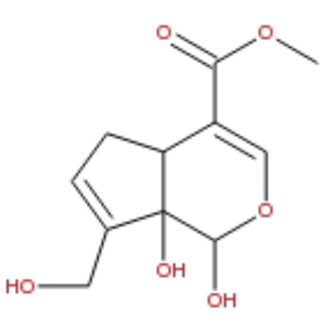

Cell ID	Cluster Center	Number of Compounds
364	0	13

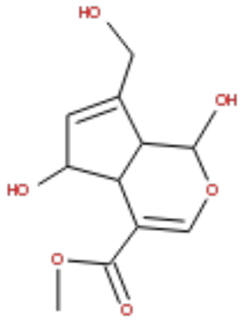

Cell ID	Cluster Center	Number of Compounds
364	0	13

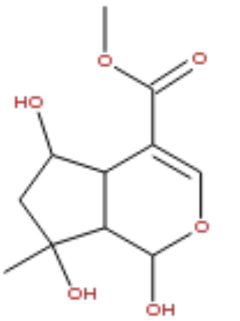

Cell ID	Cluster Center	Number of Compounds
364	0	13

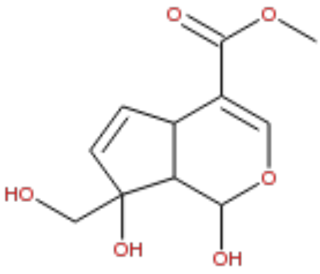

Cell ID	Cluster Center	Number of Compounds
364	0	13

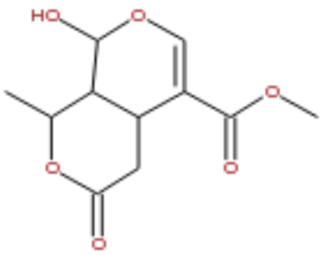

Cell ID	Cluster Center	Number of Compounds
364	0	13

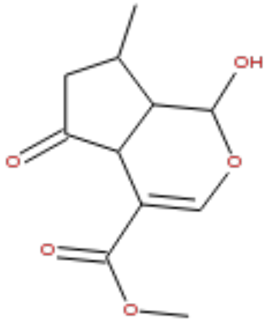

Cell ID	Cluster Center	Number of Compounds
364	0	13

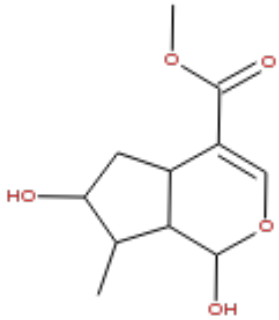

Cell ID	Cluster Center	Number of Compounds
364	0	13

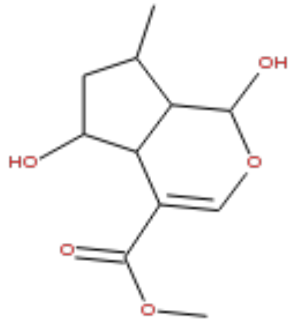

Cell ID	Cluster Center	Number of Compounds
364	0	13

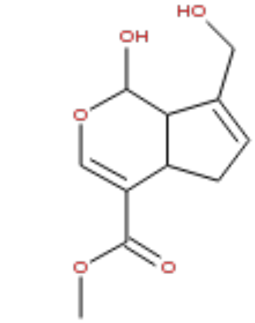

Cell ID	Cluster Center	Number of Compounds
364	0	13

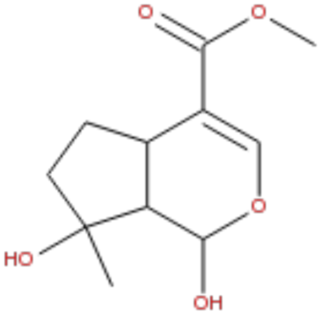

Cell ID	Cluster Center	Number of Compounds
364	0	13

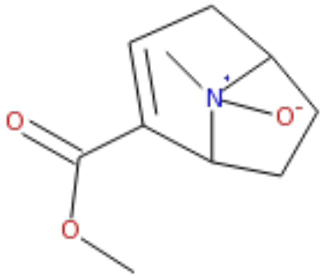

Cell ID	Cluster Center	Number of Compounds
364	0	13

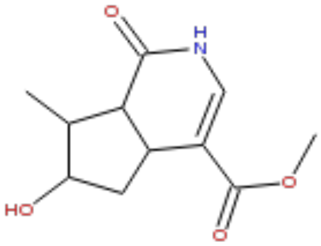

Cell ID	Cluster Center	Number of Compounds
364	0	13

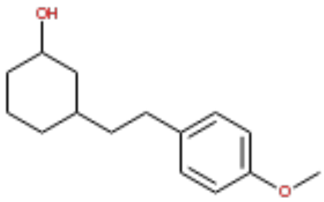

Cell ID	Cluster Center	Number of Compounds
365	1	3

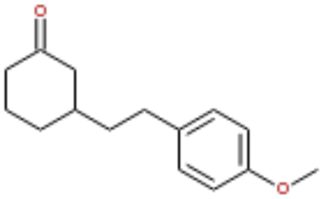

Cell ID	Cluster Center	Number of Compounds
365	0	3

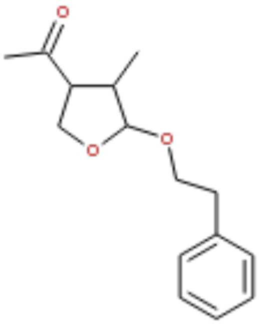

Cell ID	Cluster Center	Number of Compounds
365	0	3

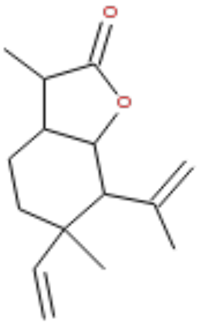

Cell ID	Cluster Center	Number of Compounds
366	1	1

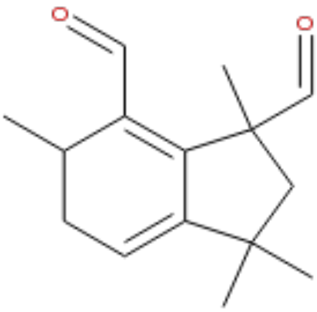

Cell ID	Cluster Center	Number of Compounds
367	1	1

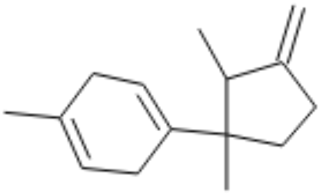

Cell ID	Cluster Center	Number of Compounds
369	1	3

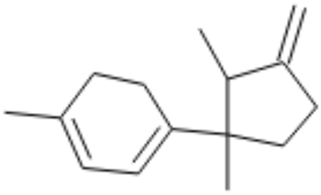

Cell ID	Cluster Center	Number of Compounds
369	0	3

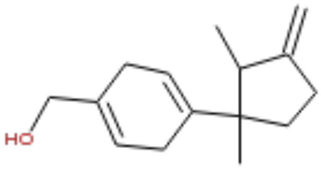

Cell ID	Cluster Center	Number of Compounds
369	0	3

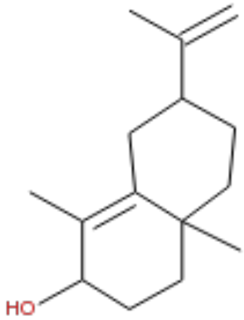

Cell ID	Cluster Center	Number of Compounds
370	1	4

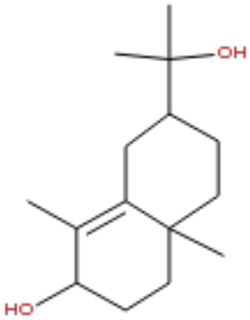

Cell ID	Cluster Center	Number of Compounds
370	0	4

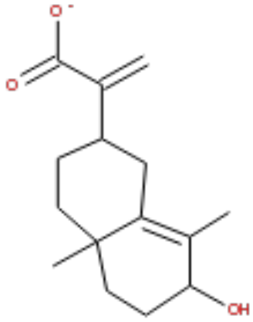

Cell ID	Cluster Center	Number of Compounds
370	0	4

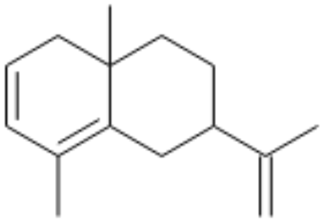

Cell ID	Cluster Center	Number of Compounds
370	0	4

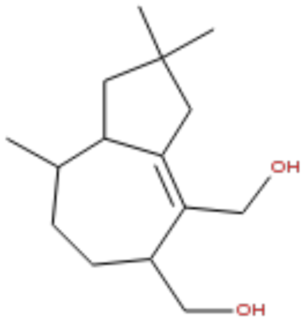

Cell ID	Cluster Center	Number of Compounds
371	1	1

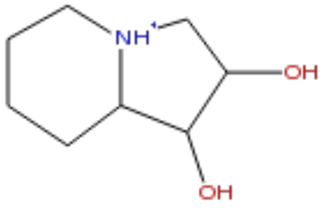

Cell ID	Cluster Center	Number of Compounds
375	1	42

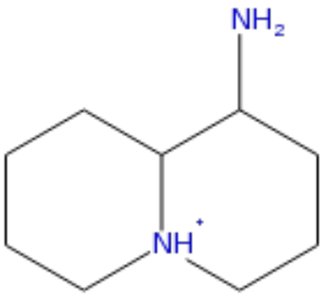

Cell ID	Cluster Center	Number of Compounds
375	0	42

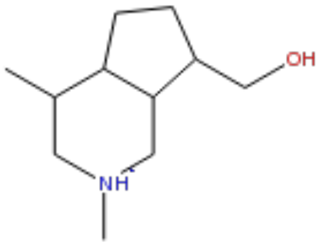

Cell ID	Cluster Center	Number of Compounds
375	0	42

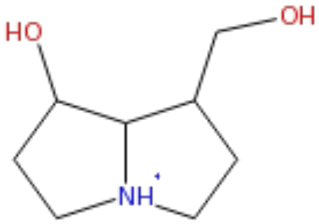

Cell ID	Cluster Center	Number of Compounds
375	0	42

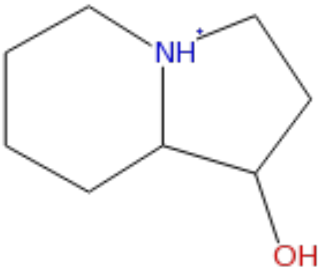

Cell ID	Cluster Center	Number of Compounds
375	0	42

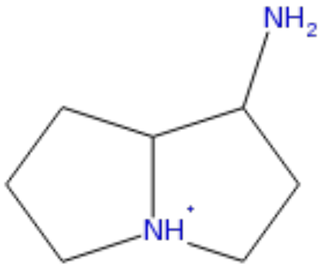

Cell ID	Cluster Center	Number of Compounds
375	0	42

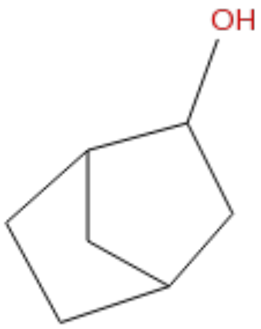

Cell ID	Cluster Center	Number of Compounds
375	0	42

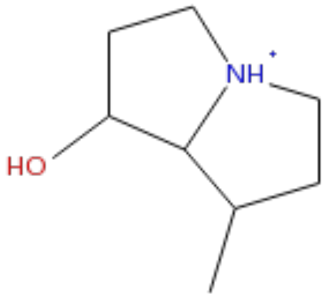

Cell ID	Cluster Center	Number of Compounds
375	0	42

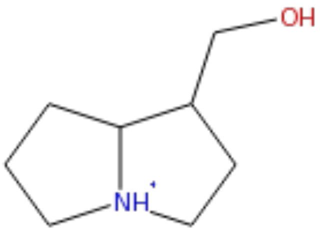

Cell ID	Cluster Center	Number of Compounds
375	0	42

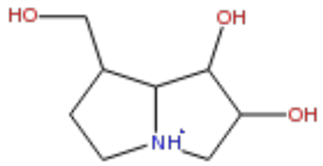

Cell ID	Cluster Center	Number of Compounds
375	0	42

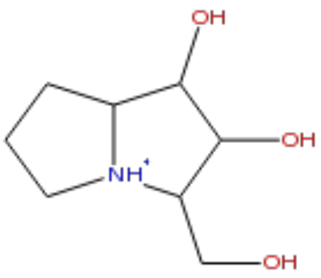

Cell ID	Cluster Center	Number of Compounds
375	0	42

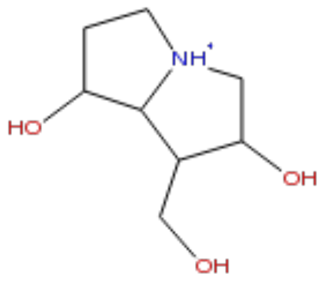

Cell ID	Cluster Center	Number of Compounds
375	0	42

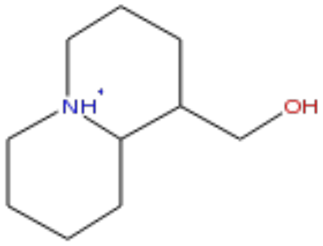

Cell ID	Cluster Center	Number of Compounds
375	0	42

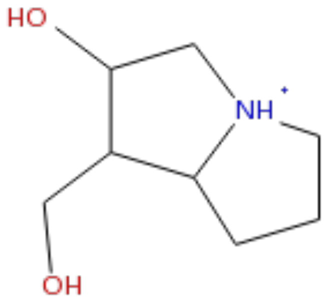

Cell ID	Cluster Center	Number of Compounds
375	0	42

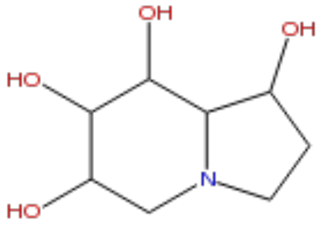

Cell ID	Cluster Center	Number of Compounds
375	0	42

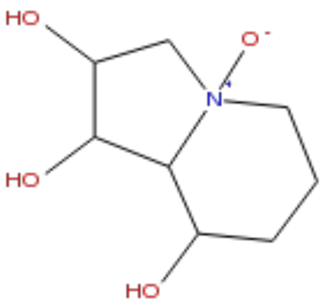

Cell ID	Cluster Center	Number of Compounds
375	0	42

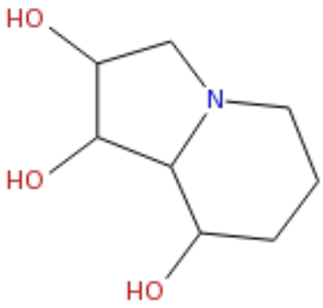

Cell ID	Cluster Center	Number of Compounds
375	0	42

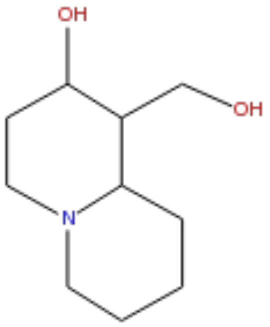

Cell ID	Cluster Center	Number of Compounds
375	0	42

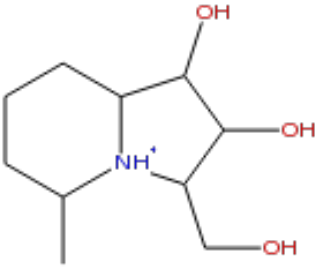

Cell ID	Cluster Center	Number of Compounds
375	0	42

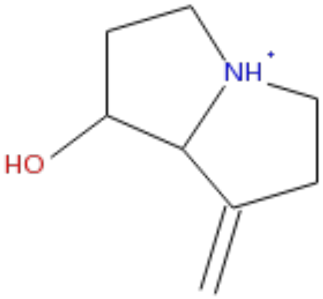

Cell ID	Cluster Center	Number of Compounds
375	0	42

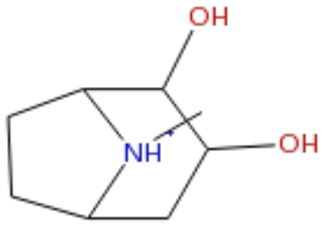

Cell ID	Cluster Center	Number of Compounds
375	0	42

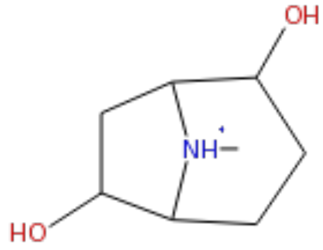

Cell ID	Cluster Center	Number of Compounds
375	0	42

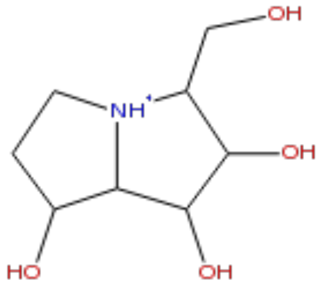

Cell ID	Cluster Center	Number of Compounds
375	0	42

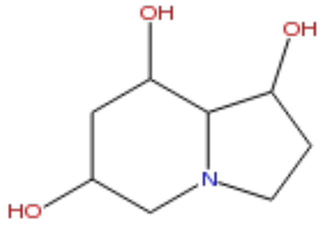

Cell ID	Cluster Center	Number of Compounds
375	0	42

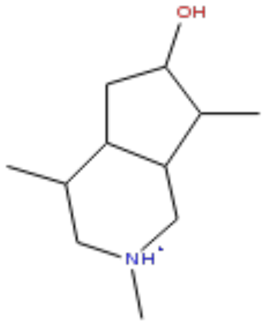

Cell ID	Cluster Center	Number of Compounds
375	0	42

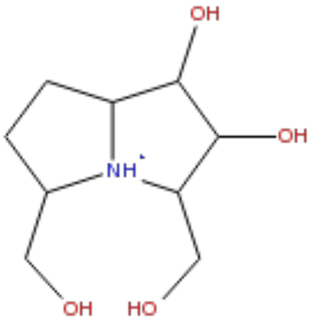

Cell ID	Cluster Center	Number of Compounds
375	0	42

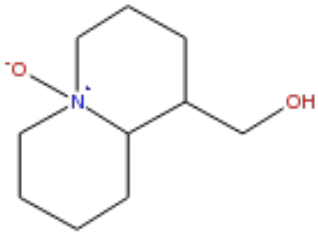

Cell ID	Cluster Center	Number of Compounds
375	0	42

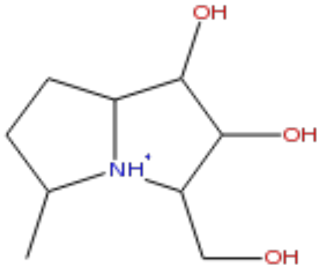

Cell ID	Cluster Center	Number of Compounds
375	0	42

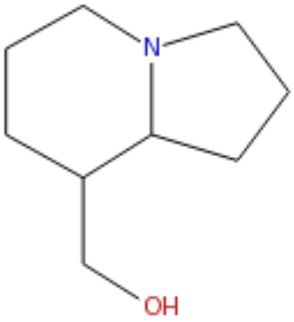

Cell ID	Cluster Center	Number of Compounds
375	0	42

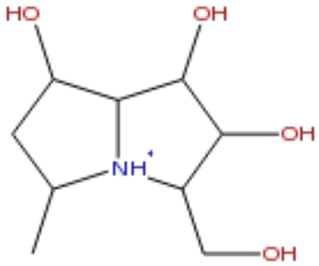

Cell ID	Cluster Center	Number of Compounds
375	0	42

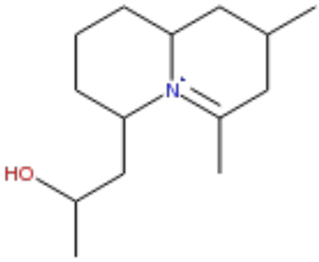

Cell ID	Cluster Center	Number of Compounds
375	0	42

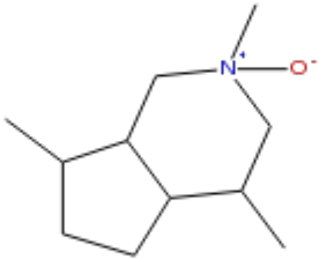

Cell ID	Cluster Center	Number of Compounds
375	0	42

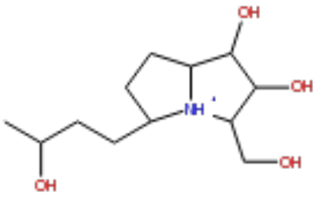

Cell ID	Cluster Center	Number of Compounds
375	0	42

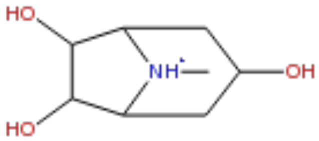

Cell ID	Cluster Center	Number of Compounds
375	0	42

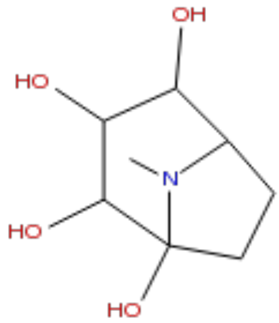

Cell ID	Cluster Center	Number of Compounds
375	0	42

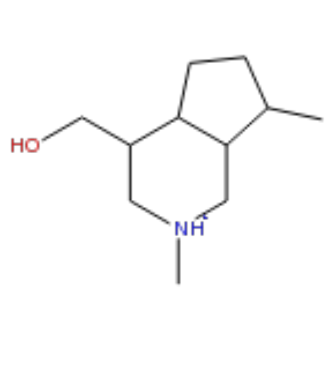

Cell ID	Cluster Center	Number of Compounds
375	0	42

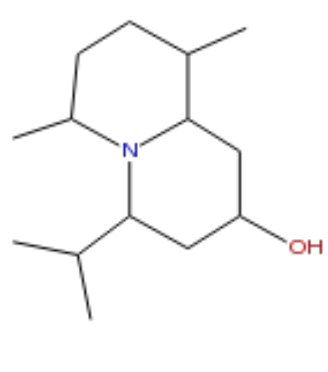

Cell ID	Cluster Center	Number of Compounds
375	0	42

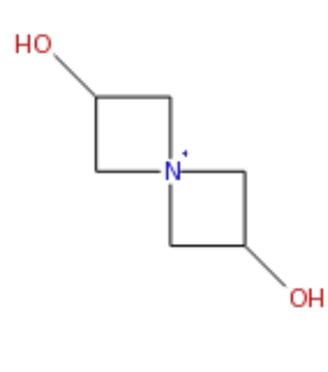

Cell ID	Cluster Center	Number of Compounds
375	0	42

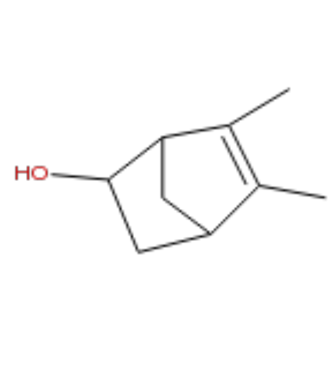

Cell ID	Cluster Center	Number of Compounds
375	0	42

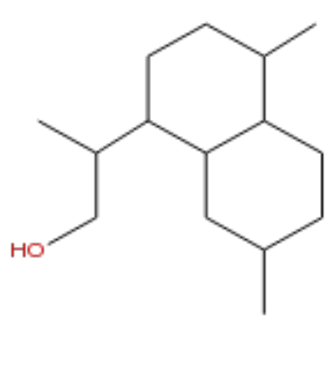

Cell ID	Cluster Center	Number of Compounds
375	0	42

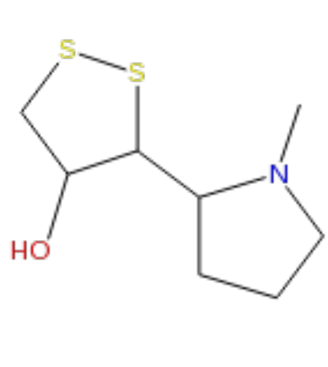

Cell ID	Cluster Center	Number of Compounds
375	0	42

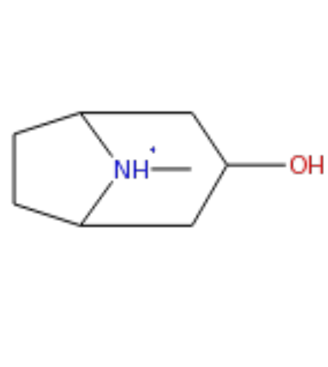

Cell ID	Cluster Center	Number of Compounds
375	0	42

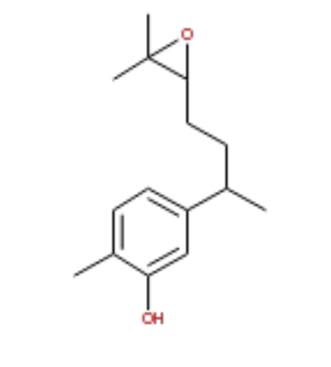

Cell ID	Cluster Center	Number of Compounds
376	1	1

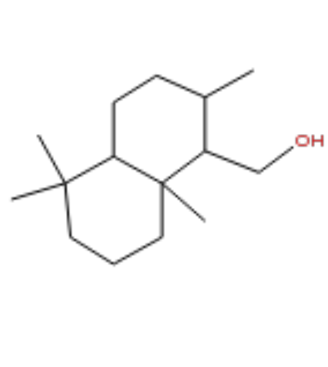

Cell ID	Cluster Center	Number of Compounds
378	1	13

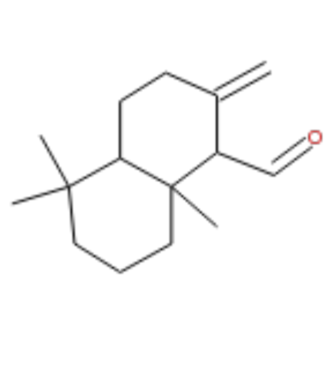

Cell ID	Cluster Center	Number of Compounds
378	0	13

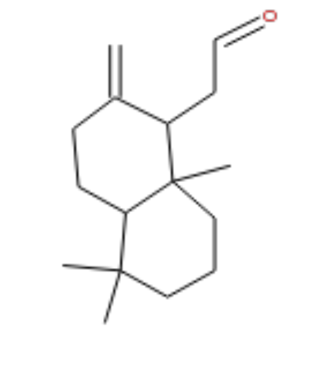

Cell ID	Cluster Center	Number of Compounds
378	0	13

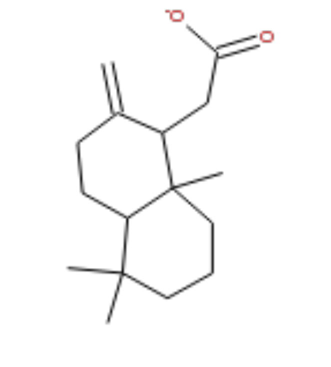

Cell ID	Cluster Center	Number of Compounds
378	0	13

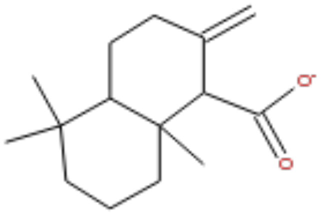

Cell ID	Cluster Center	Number of Compounds
378	0	13

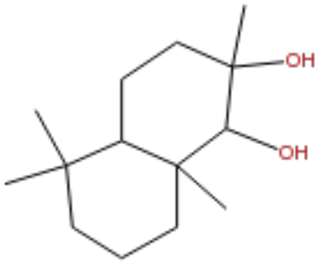

Cell ID	Cluster Center	Number of Compounds
378	0	13

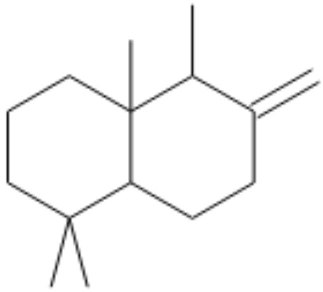

Cell ID	Cluster Center	Number of Compounds
378	0	13

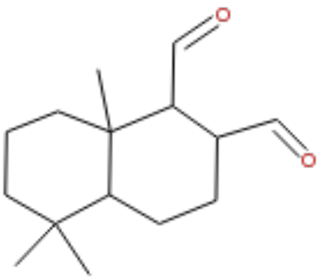

Cell ID	Cluster Center	Number of Compounds
378	0	13

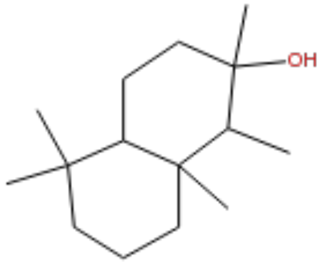

Cell ID	Cluster Center	Number of Compounds
378	0	13

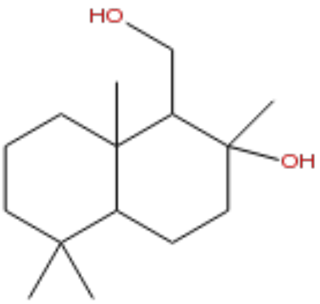

Cell ID	Cluster Center	Number of Compounds
378	0	13

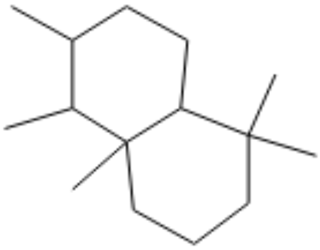

Cell ID	Cluster Center	Number of Compounds
378	0	13

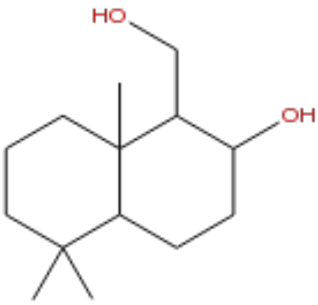

Cell ID	Cluster Center	Number of Compounds
378	0	13

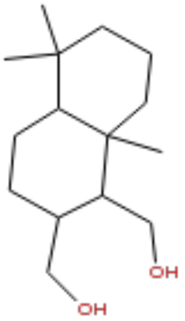

Cell ID	Cluster Center	Number of Compounds
378	0	13

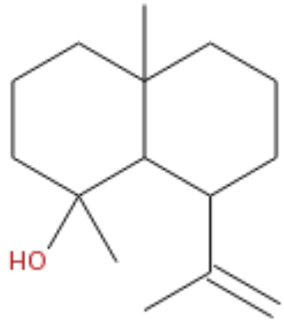

Cell ID	Cluster Center	Number of Compounds
380	1	6

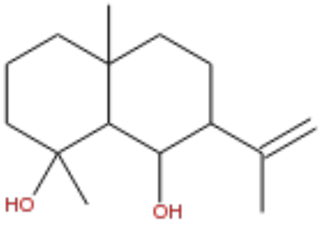

Cell ID	Cluster Center	Number of Compounds
380	0	6

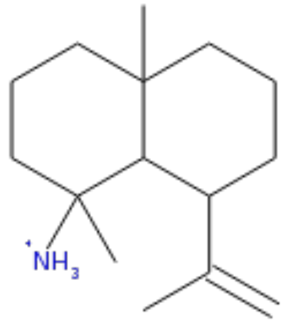

Cell ID	Cluster Center	Number of Compounds
380	0	6

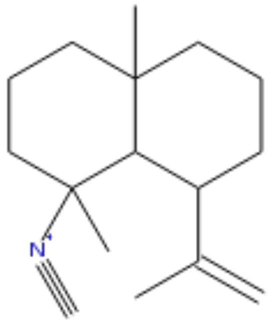

Cell ID	Cluster Center	Number of Compounds
380	0	6

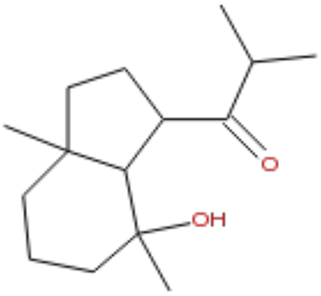

Cell ID	Cluster Center	Number of Compounds
380	0	6

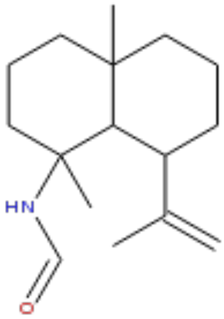

Cell ID	Cluster Center	Number of Compounds
380	0	6

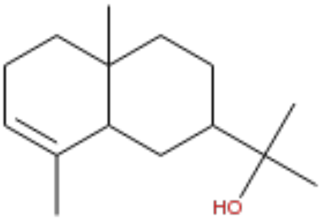

Cell ID	Cluster Center	Number of Compounds
381	1	12

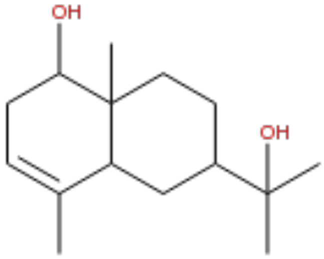

Cell ID	Cluster Center	Number of Compounds
381	0	12

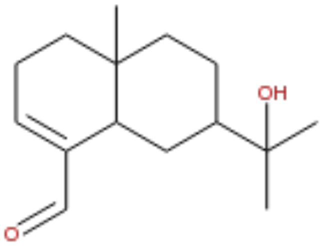

Cell ID	Cluster Center	Number of Compounds
381	0	12

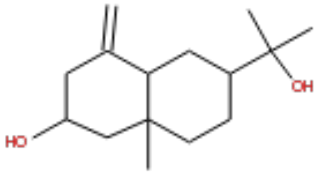

Cell ID	Cluster Center	Number of Compounds
381	0	12

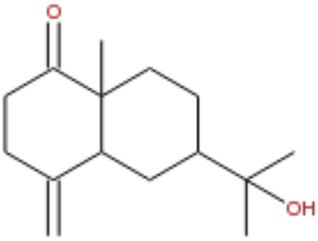

Cell ID	Cluster Center	Number of Compounds
381	0	12

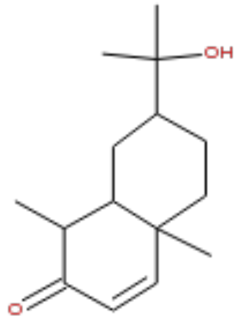

Cell ID	Cluster Center	Number of Compounds
381	0	12

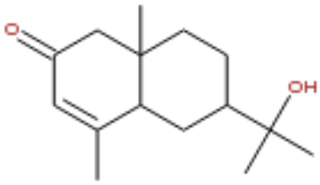

Cell ID	Cluster Center	Number of Compounds
381	0	12

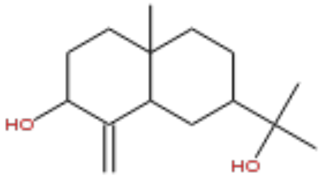

Cell ID	Cluster Center	Number of Compounds
381	0	12

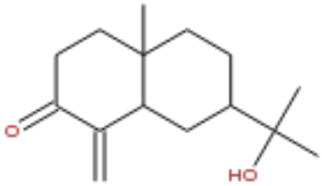

Cell ID	Cluster Center	Number of Compounds
381	0	12

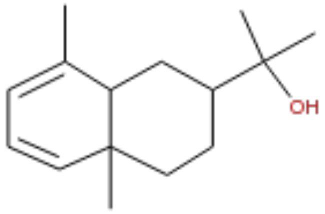

Cell ID	Cluster Center	Number of Compounds
381	0	12

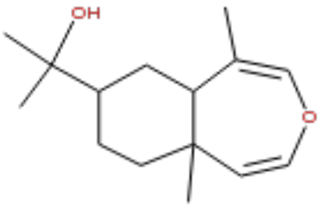

Cell ID	Cluster Center	Number of Compounds
381	0	12

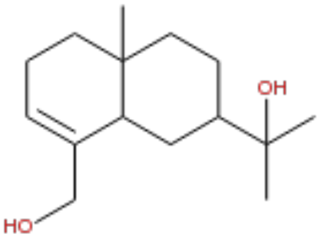

Cell ID	Cluster Center	Number of Compounds
381	0	12

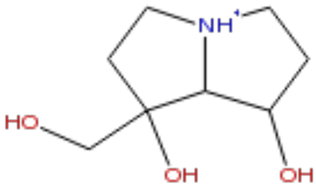

Cell ID	Cluster Center	Number of Compounds
382	1	19

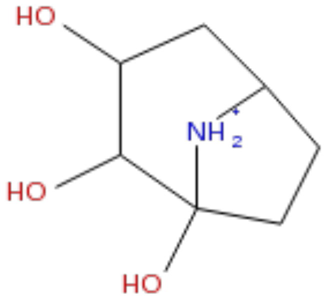

Cell ID	Cluster Center	Number of Compounds
382	0	19

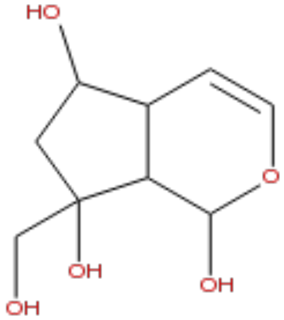

Cell ID	Cluster Center	Number of Compounds
382	0	19

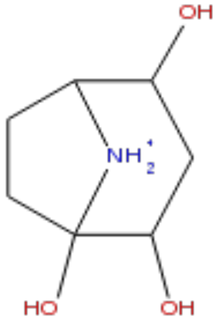

Cell ID	Cluster Center	Number of Compounds
382	0	19

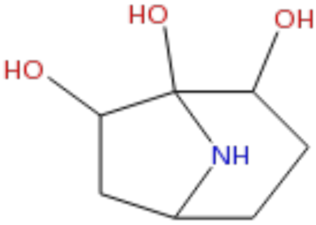

Cell ID	Cluster Center	Number of Compounds
382	0	19

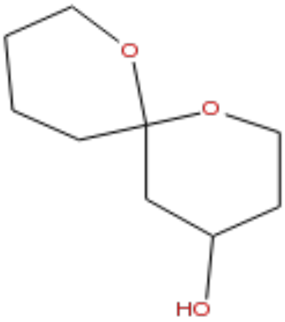

Cell ID	Cluster Center	Number of Compounds
382	0	19

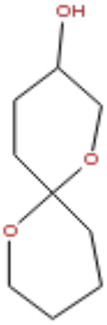

Cell ID	Cluster Center	Number of Compounds
382	0	19

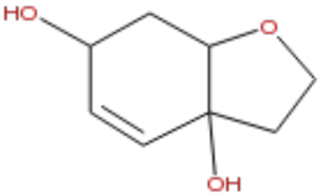

Cell ID	Cluster Center	Number of Compounds
382	0	19

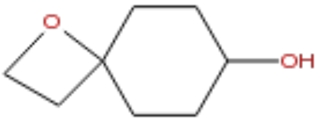

Cell ID	Cluster Center	Number of Compounds
382	0	19

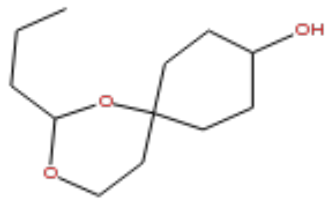

Cell ID	Cluster Center	Number of Compounds
382	0	19

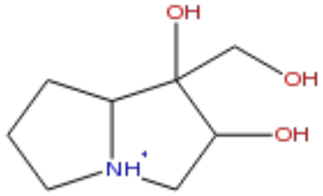

Cell ID	Cluster Center	Number of Compounds
382	0	19

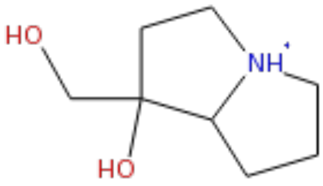

Cell ID	Cluster Center	Number of Compounds
382	0	19

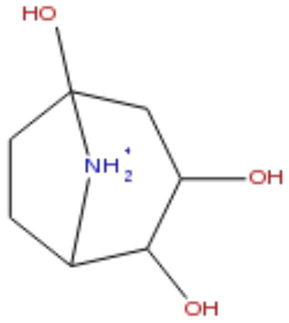

Cell ID	Cluster Center	Number of Compounds
382	0	19

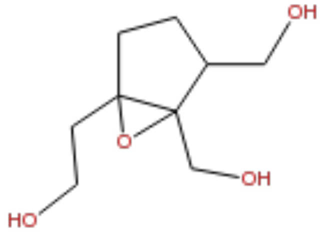

Cell ID	Cluster Center	Number of Compounds
382	0	19

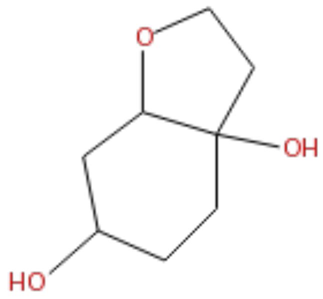

Cell ID	Cluster Center	Number of Compounds
382	0	19

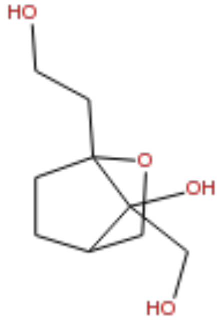

Cell ID	Cluster Center	Number of Compounds
382	0	19

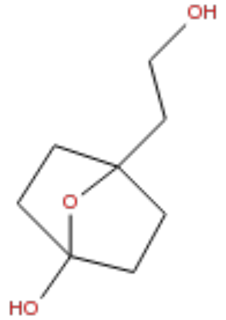

Cell ID	Cluster Center	Number of Compounds
382	0	19

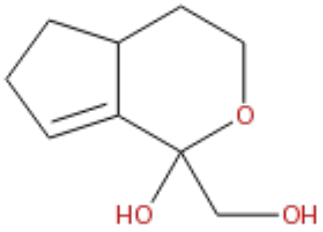

Cell ID	Cluster Center	Number of Compounds
382	0	19

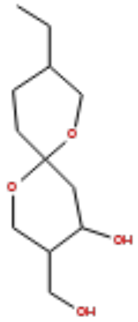

Cell ID	Cluster Center	Number of Compounds
382	0	19

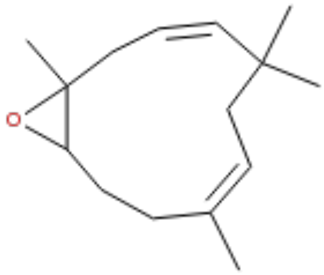

Cell ID	Cluster Center	Number of Compounds
384	1	4

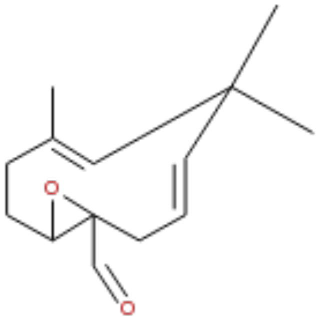

Cell ID	Cluster Center	Number of Compounds
384	0	4

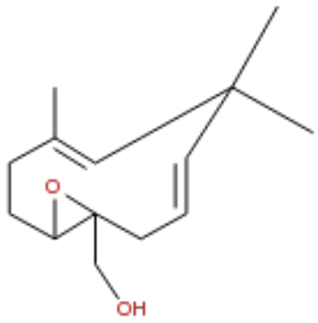

Cell ID	Cluster Center	Number of Compounds
384	0	4

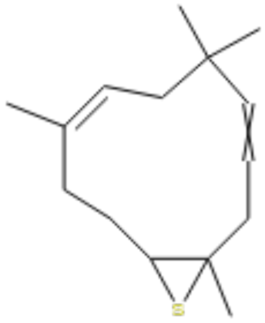

Cell ID	Cluster Center	Number of Compounds
384	0	4

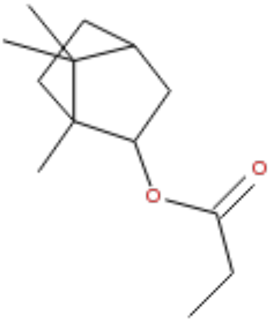

Cell ID	Cluster Center	Number of Compounds
385	1	8

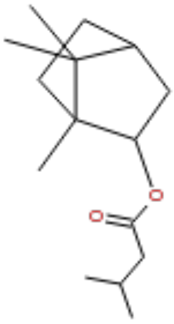

Cell ID	Cluster Center	Number of Compounds
385	0	8

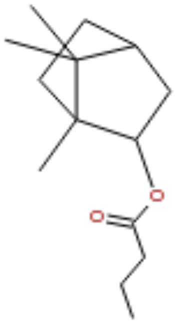

Cell ID	Cluster Center	Number of Compounds
385	0	8

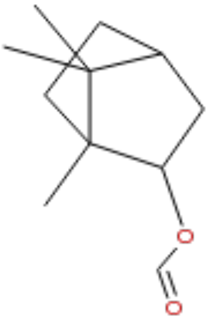

Cell ID	Cluster Center	Number of Compounds
385	0	8

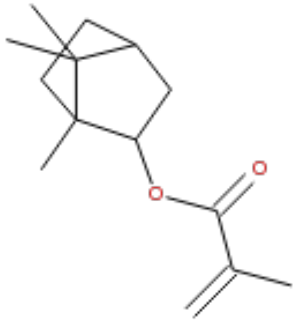

Cell ID	Cluster Center	Number of Compounds
385	0	8

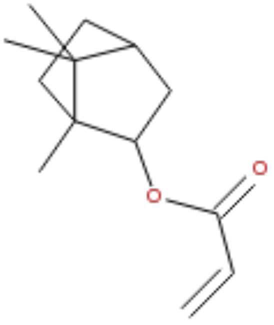

Cell ID	Cluster Center	Number of Compounds
385	0	8

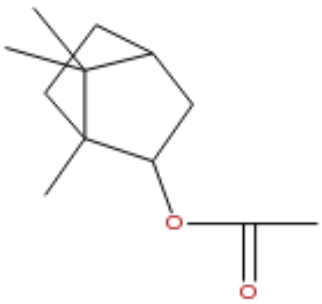

Cell ID	Cluster Center	Number of Compounds
385	0	8

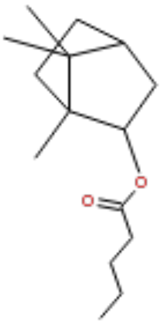

Cell ID	Cluster Center	Number of Compounds
385	0	8

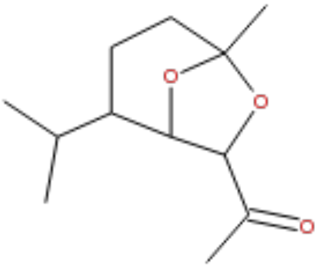

Cell ID	Cluster Center	Number of Compounds
386	1	14

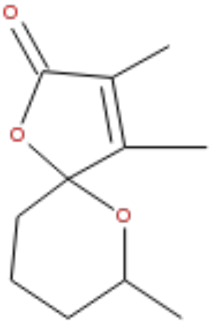

Cell ID	Cluster Center	Number of Compounds
386	0	14

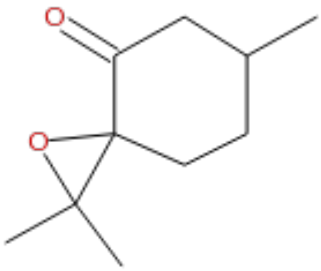

Cell ID	Cluster Center	Number of Compounds
386	0	14

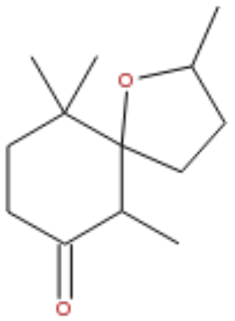

Cell ID	Cluster Center	Number of Compounds
386	0	14

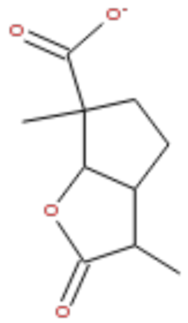

Cell ID	Cluster Center	Number of Compounds
386	0	14

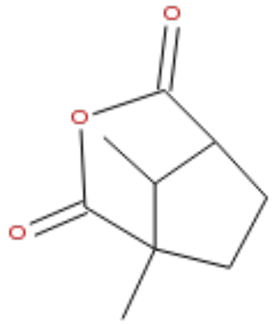

Cell ID	Cluster Center	Number of Compounds
386	0	14

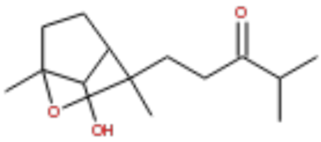

Cell ID	Cluster Center	Number of Compounds
386	0	14

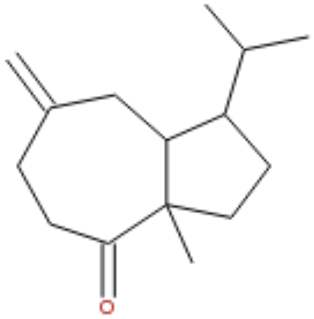

Cell ID	Cluster Center	Number of Compounds
386	0	14

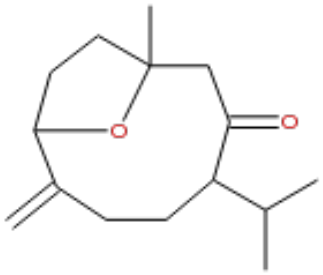

Cell ID	Cluster Center	Number of Compounds
386	0	14

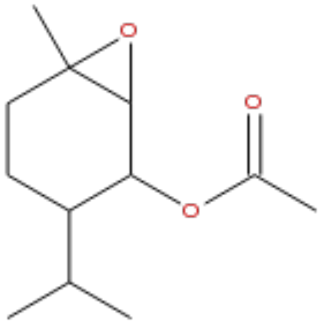

Cell ID	Cluster Center	Number of Compounds
386	0	14

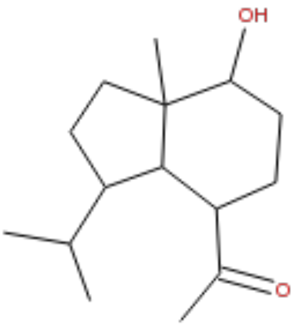

Cell ID	Cluster Center	Number of Compounds
386	0	14

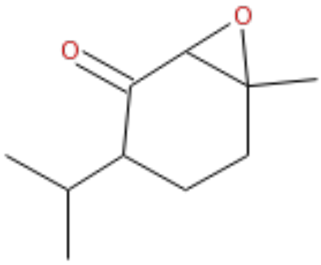

Cell ID	Cluster Center	Number of Compounds
386	0	14

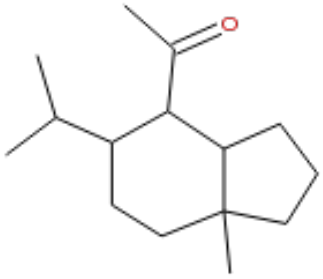

Cell ID	Cluster Center	Number of Compounds
386	0	14

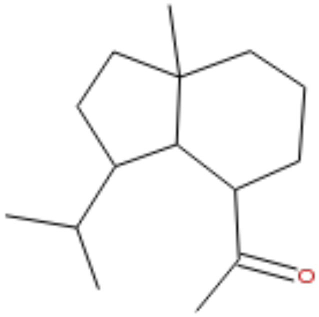

Cell ID	Cluster Center	Number of Compounds
386	0	14

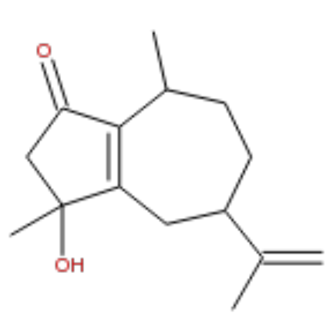

Cell ID	Cluster Center	Number of Compounds
387	1	2

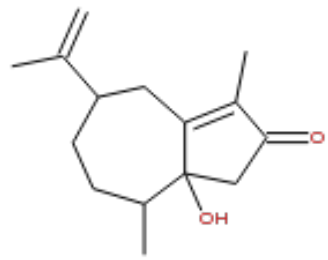

Cell ID	Cluster Center	Number of Compounds
387	0	2

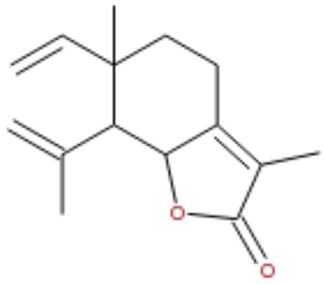

Cell ID	Cluster Center	Number of Compounds
388	1	2

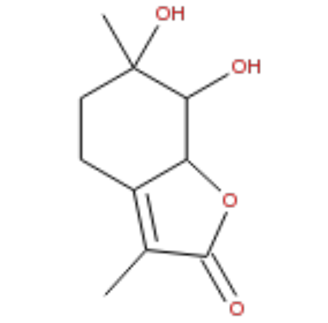

Cell ID	Cluster Center	Number of Compounds
388	0	2

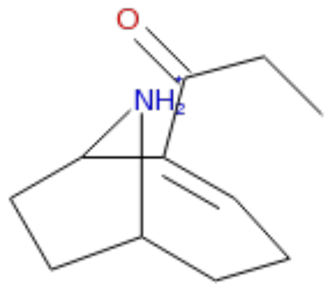

Cell ID	Cluster Center	Number of Compounds
389	1	4

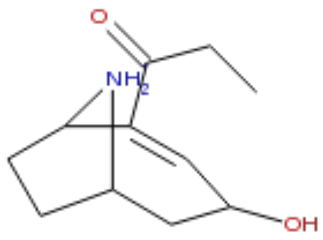

Cell ID	Cluster Center	Number of Compounds
389	0	4

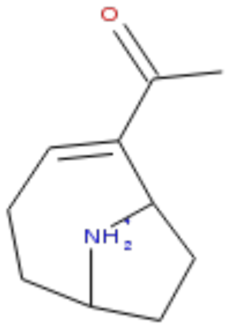

Cell ID	Cluster Center	Number of Compounds
389	0	4

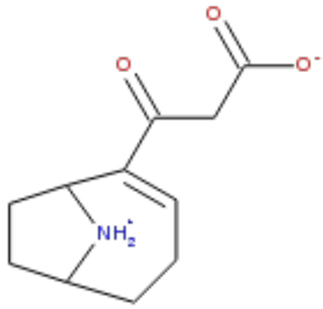

Cell ID	Cluster Center	Number of Compounds
389	0	4

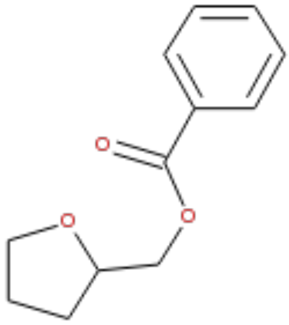

Cell ID	Cluster Center	Number of Compounds
390	1	9

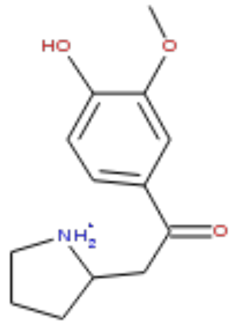

Cell ID	Cluster Center	Number of Compounds
390	0	9

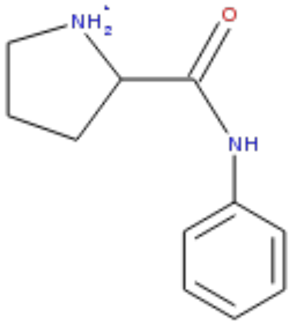

Cell ID	Cluster Center	Number of Compounds
390	0	9

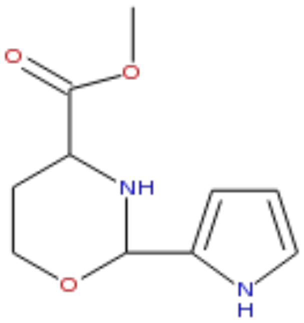

Cell ID	Cluster Center	Number of Compounds
390	0	9

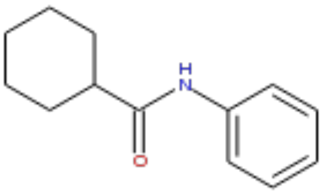

Cell ID	Cluster Center	Number of Compounds
390	0	9

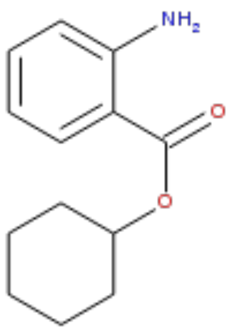

Cell ID	Cluster Center	Number of Compounds
390	0	9

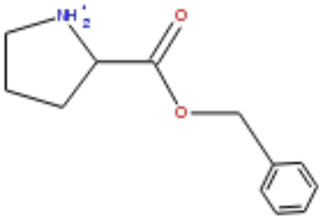

Cell ID	Cluster Center	Number of Compounds
390	0	9

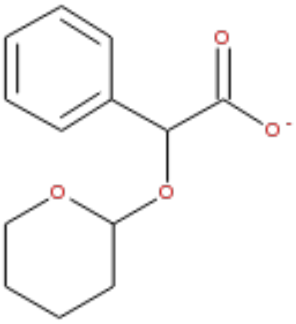

Cell ID	Cluster Center	Number of Compounds
390	0	9

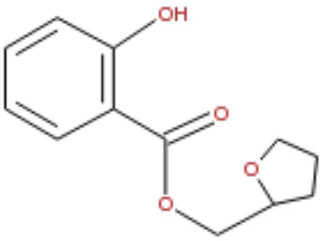

Cell ID	Cluster Center	Number of Compounds
390	0	9

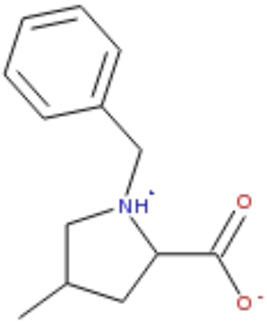

Cell ID	Cluster Center	Number of Compounds
391	1	13

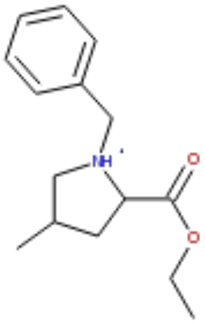

Cell ID	Cluster Center	Number of Compounds
391	0	13

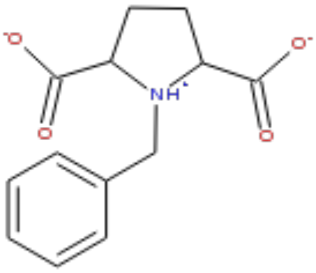

Cell ID	Cluster Center	Number of Compounds
391	0	13

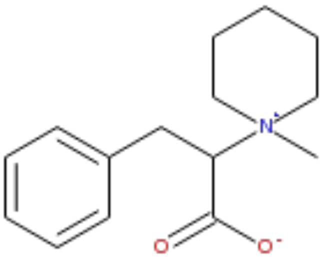

Cell ID	Cluster Center	Number of Compounds
391	0	13

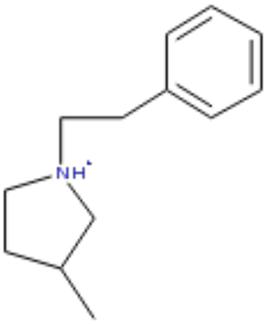

Cell ID	Cluster Center	Number of Compounds
391	0	13

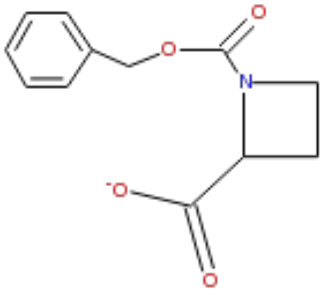

Cell ID	Cluster Center	Number of Compounds
391	0	13

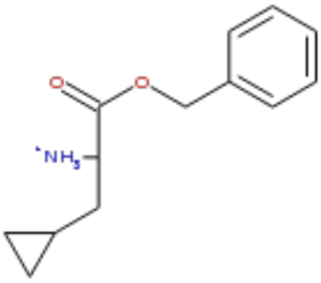

Cell ID	Cluster Center	Number of Compounds
391	0	13

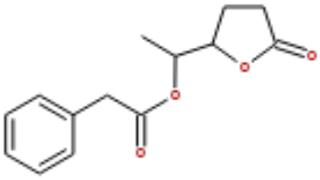

Cell ID	Cluster Center	Number of Compounds
391	0	13

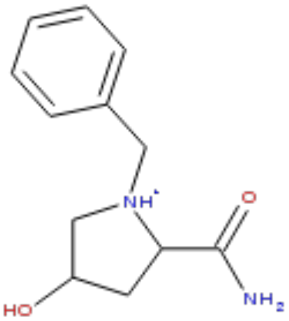

Cell ID	Cluster Center	Number of Compounds
391	0	13

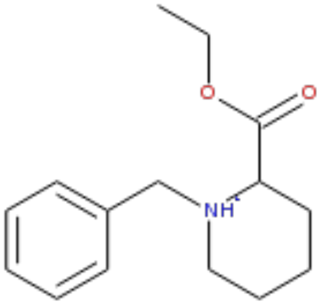

Cell ID	Cluster Center	Number of Compounds
391	0	13

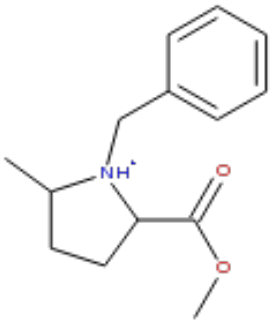

Cell ID	Cluster Center	Number of Compounds
391	0	13

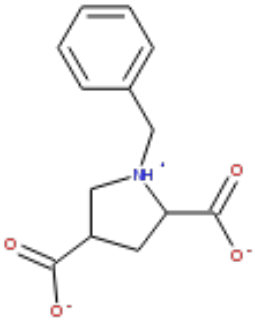

Cell ID	Cluster Center	Number of Compounds
391	0	13

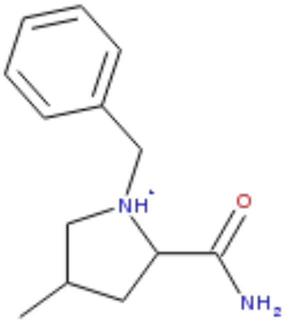

Cell ID	Cluster Center	Number of Compounds
391	0	13

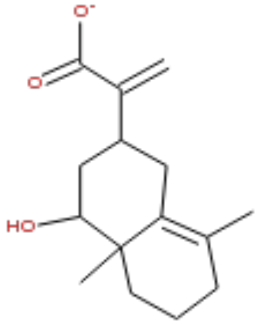

Cell ID	Cluster Center	Number of Compounds
392	1	6

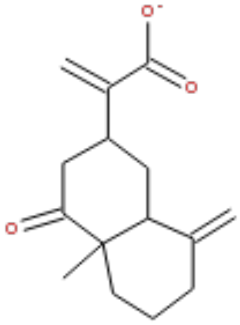

Cell ID	Cluster Center	Number of Compounds
392	0	6

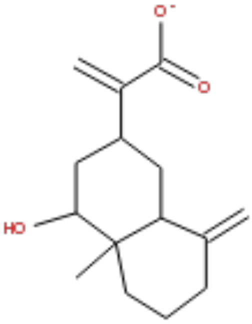

Cell ID	Cluster Center	Number of Compounds
392	0	6

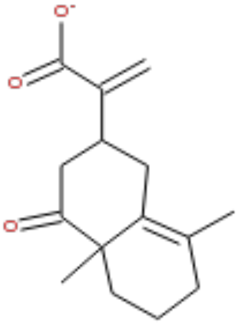

Cell ID	Cluster Center	Number of Compounds
392	0	6

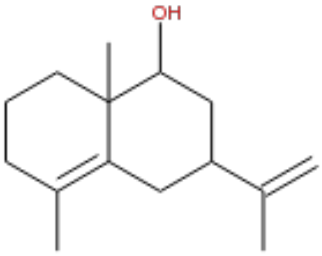

Cell ID	Cluster Center	Number of Compounds
392	0	6

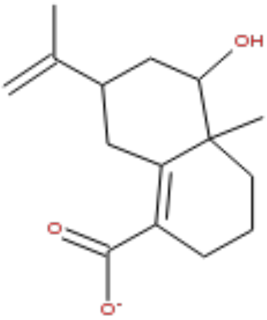

Cell ID	Cluster Center	Number of Compounds
392	0	6

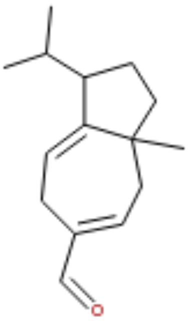

Cell ID	Cluster Center	Number of Compounds
393	1	3

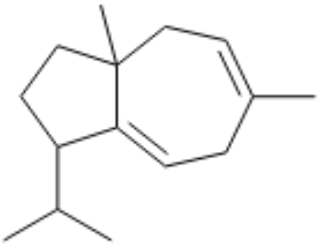

Cell ID	Cluster Center	Number of Compounds
393	0	3

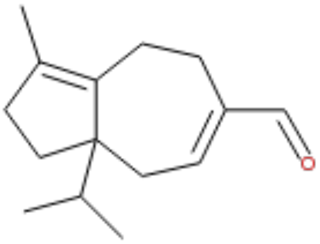

Cell ID	Cluster Center	Number of Compounds
393	0	3

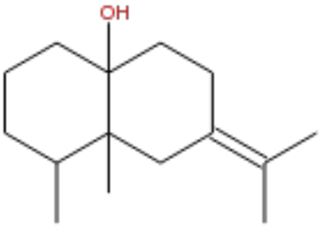

Cell ID	Cluster Center	Number of Compounds
394	1	3

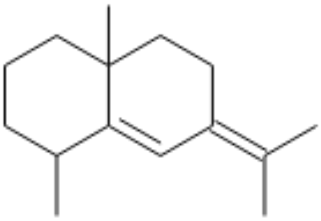

Cell ID	Cluster Center	Number of Compounds
394	0	3

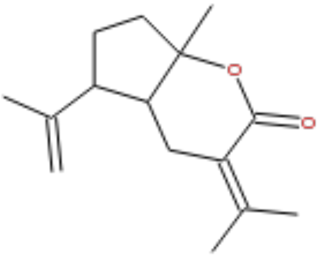

Cell ID	Cluster Center	Number of Compounds
394	0	3

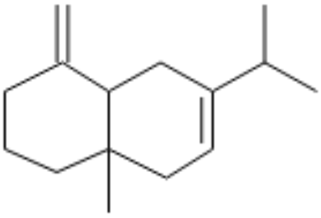

Cell ID	Cluster Center	Number of Compounds
395	1	5

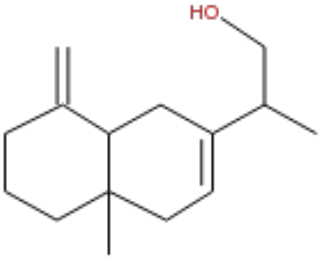

Cell ID	Cluster Center	Number of Compounds
395	0	5

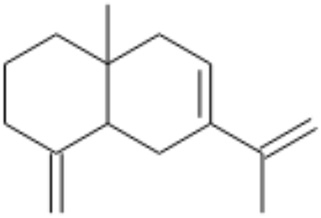

Cell ID	Cluster Center	Number of Compounds
395	0	5

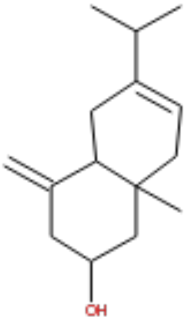

Cell ID	Cluster Center	Number of Compounds
395	0	5

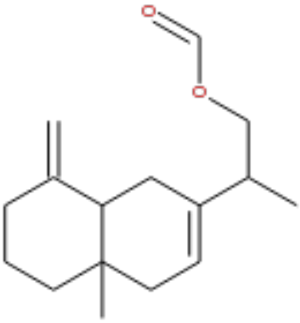

Cell ID	Cluster Center	Number of Compounds
395	0	5

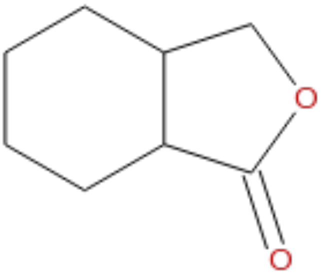

Cell ID	Cluster Center	Number of Compounds
396	1	23

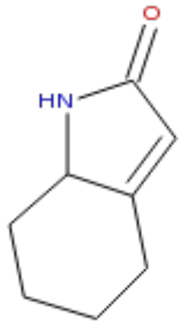

Cell ID	Cluster Center	Number of Compounds
396	0	23

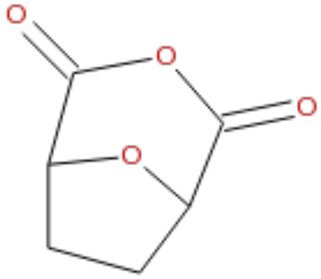

Cell ID	Cluster Center	Number of Compounds
396	0	23

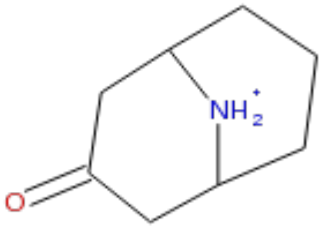

Cell ID	Cluster Center	Number of Compounds
396	0	23

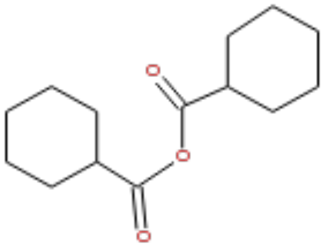

Cell ID	Cluster Center	Number of Compounds
396	0	23

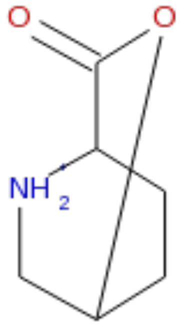

Cell ID	Cluster Center	Number of Compounds
396	0	23

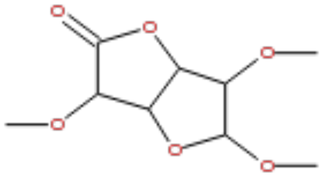

Cell ID	Cluster Center	Number of Compounds
396	0	23

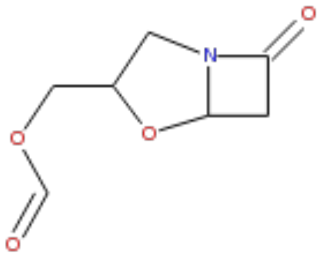

Cell ID	Cluster Center	Number of Compounds
396	0	23

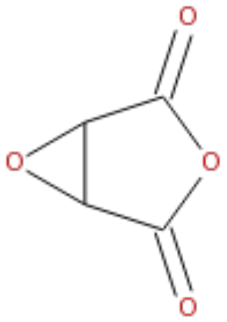

Cell ID	Cluster Center	Number of Compounds
396	0	23

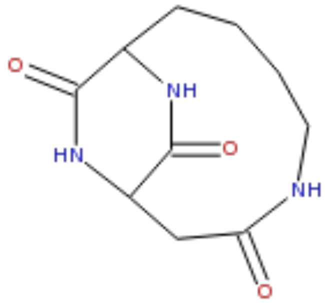

Cell ID	Cluster Center	Number of Compounds
396	0	23

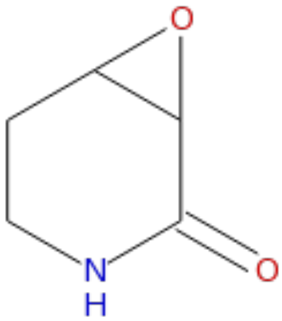

Cell ID	Cluster Center	Number of Compounds
396	0	23

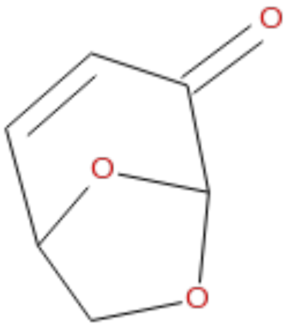

Cell ID	Cluster Center	Number of Compounds
396	0	23

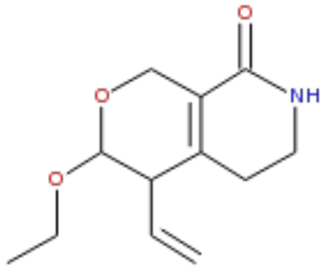

Cell ID	Cluster Center	Number of Compounds
396	0	23

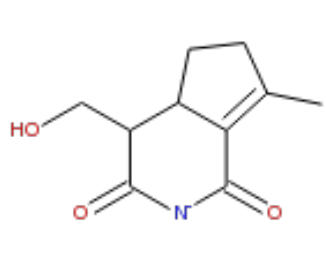

Cell ID	Cluster Center	Number of Compounds
396	0	23

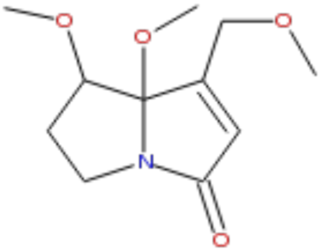

Cell ID	Cluster Center	Number of Compounds
396	0	23

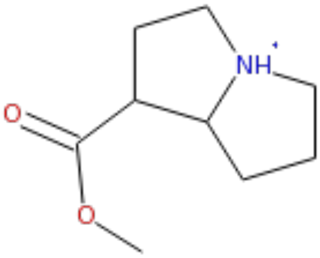

Cell ID	Cluster Center	Number of Compounds
396	0	23

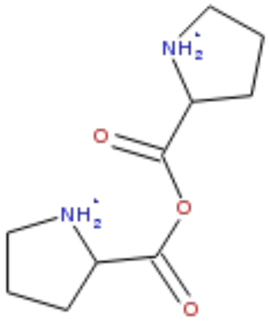

Cell ID	Cluster Center	Number of Compounds
396	0	23

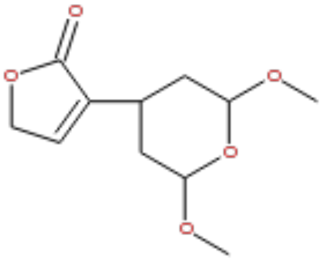

Cell ID	Cluster Center	Number of Compounds
396	0	23

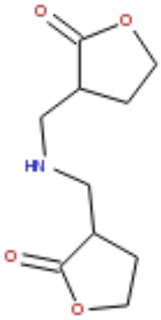

Cell ID	Cluster Center	Number of Compounds
396	0	23

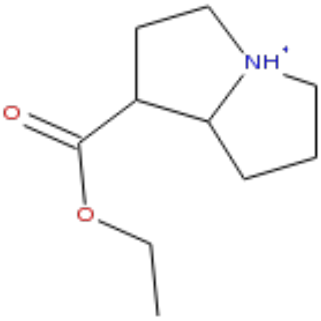

Cell ID	Cluster Center	Number of Compounds
396	0	23

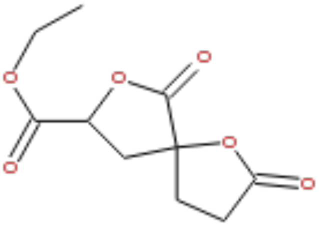

Cell ID	Cluster Center	Number of Compounds
396	0	23

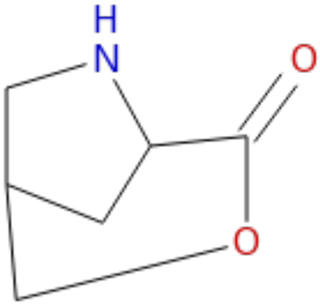

Cell ID	Cluster Center	Number of Compounds
396	0	23

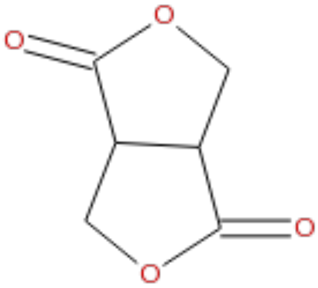

Cell ID	Cluster Center	Number of Compounds
396	0	23

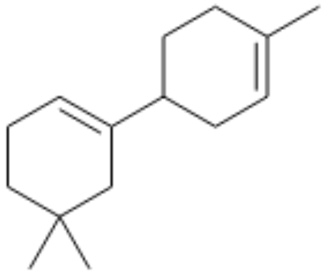

Cell ID	Cluster Center	Number of Compounds
397	1	1

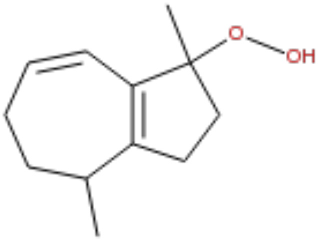

Cell ID	Cluster Center	Number of Compounds
398	1	1

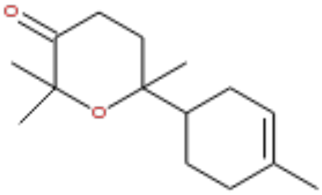

Cell ID	Cluster Center	Number of Compounds
400	1	9

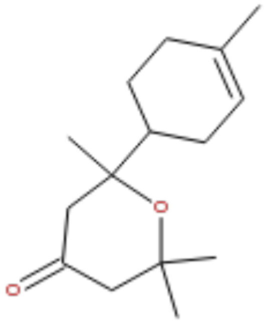

Cell ID	Cluster Center	Number of Compounds
400	0	9

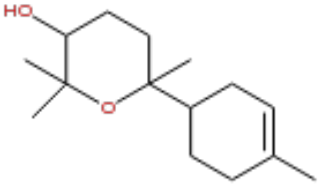

Cell ID	Cluster Center	Number of Compounds
400	0	9

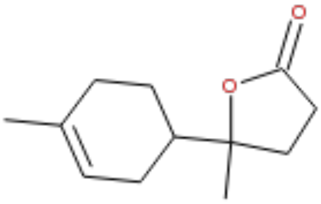

Cell ID	Cluster Center	Number of Compounds
400	0	9

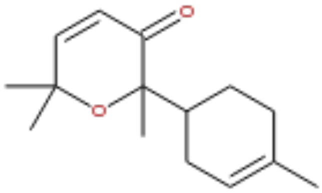

Cell ID	Cluster Center	Number of Compounds
400	0	9

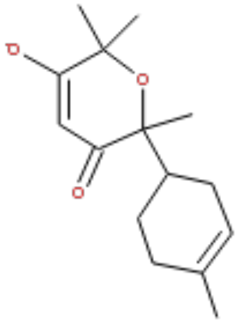

Cell ID	Cluster Center	Number of Compounds
400	0	9

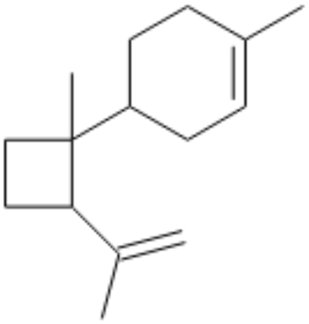

Cell ID	Cluster Center	Number of Compounds
400	0	9

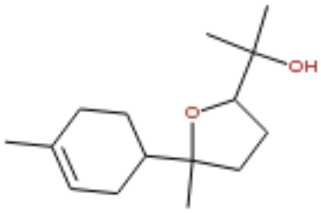

Cell ID	Cluster Center	Number of Compounds
400	0	9

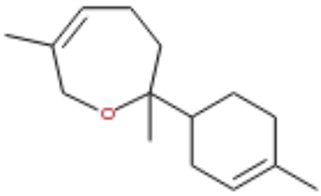

Cell ID	Cluster Center	Number of Compounds
400	0	9

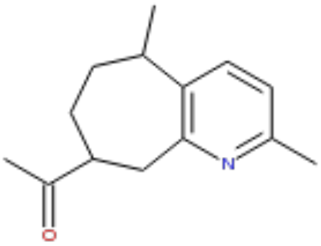

Cell ID	Cluster Center	Number of Compounds
401	1	5

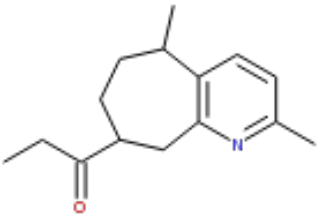

Cell ID	Cluster Center	Number of Compounds
401	0	5

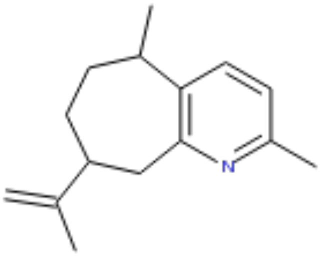

Cell ID	Cluster Center	Number of Compounds
401	0	5

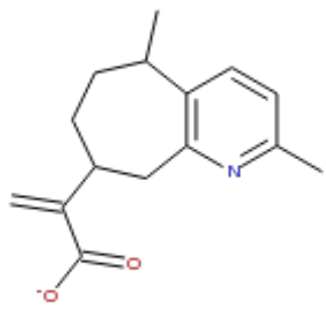

Cell ID	Cluster Center	Number of Compounds
401	0	5

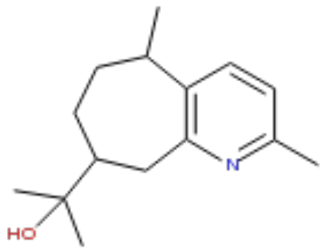

Cell ID	Cluster Center	Number of Compounds
401	0	5

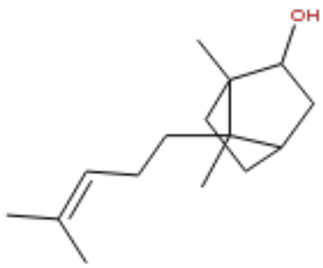

Cell ID	Cluster Center	Number of Compounds
404	1	10

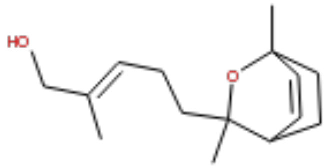

Cell ID	Cluster Center	Number of Compounds
404	0	10

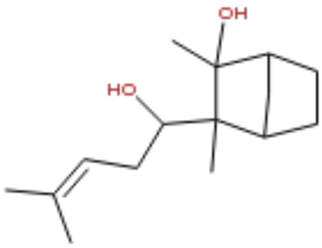

Cell ID	Cluster Center	Number of Compounds
404	0	10

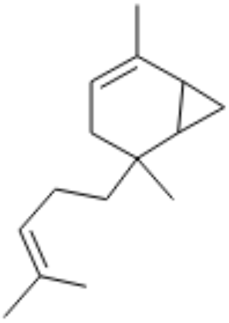

Cell ID	Cluster Center	Number of Compounds
404	0	10

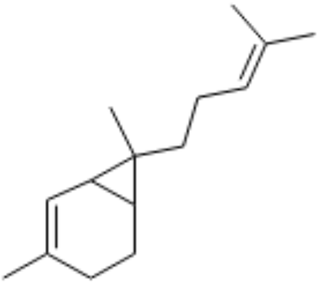

Cell ID	Cluster Center	Number of Compounds
404	0	10

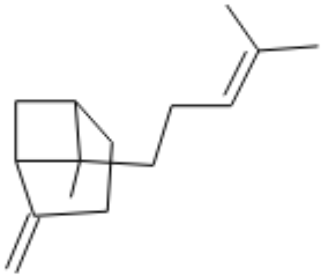

Cell ID	Cluster Center	Number of Compounds
404	0	10

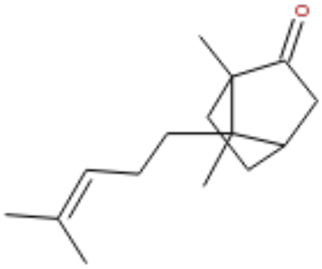

Cell ID	Cluster Center	Number of Compounds
404	0	10

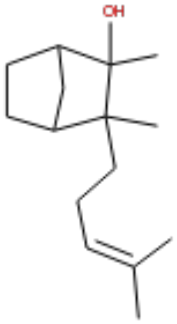

Cell ID	Cluster Center	Number of Compounds
404	0	10

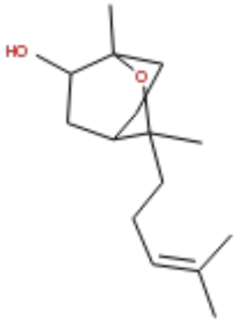

Cell ID	Cluster Center	Number of Compounds
404	0	10

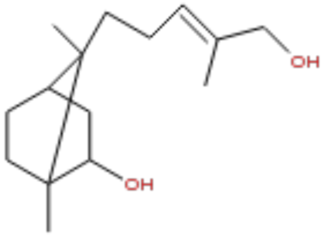

Cell ID	Cluster Center	Number of Compounds
404	0	10

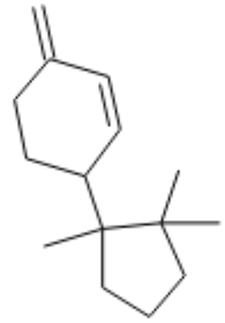

Cell ID	Cluster Center	Number of Compounds
406	1	7

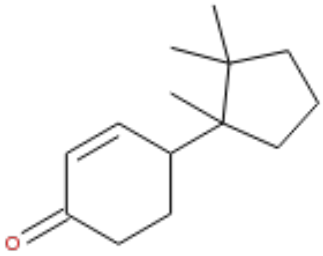

Cell ID	Cluster Center	Number of Compounds
406	0	7

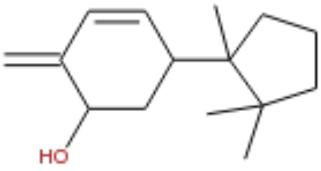

Cell ID	Cluster Center	Number of Compounds
406	0	7

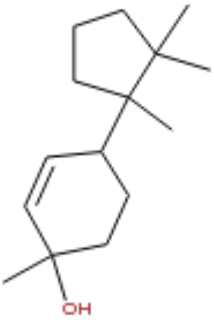

Cell ID	Cluster Center	Number of Compounds
406	0	7

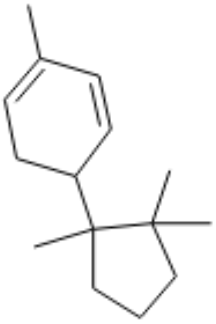

Cell ID	Cluster Center	Number of Compounds
406	0	7

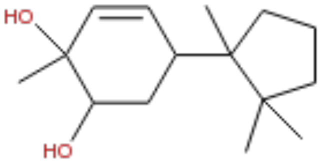

Cell ID	Cluster Center	Number of Compounds
406	0	7

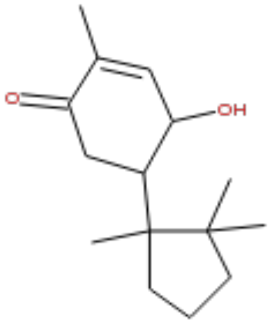

Cell ID	Cluster Center	Number of Compounds
406	0	7

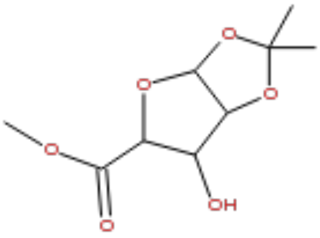

Cell ID	Cluster Center	Number of Compounds
407	1	9

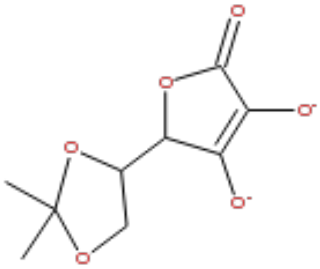

Cell ID	Cluster Center	Number of Compounds
407	0	9

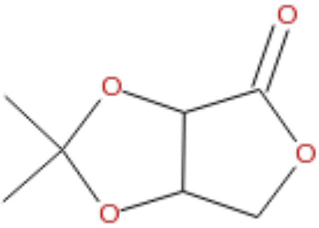

Cell ID	Cluster Center	Number of Compounds
407	0	9

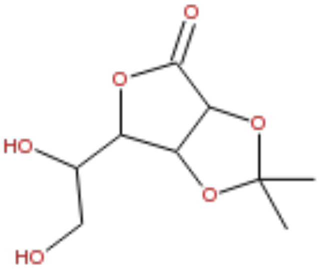

Cell ID	Cluster Center	Number of Compounds
407	0	9

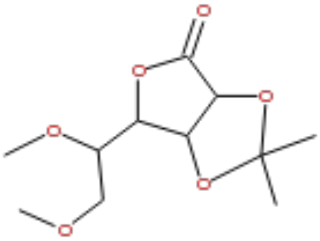

Cell ID	Cluster Center	Number of Compounds
407	0	9

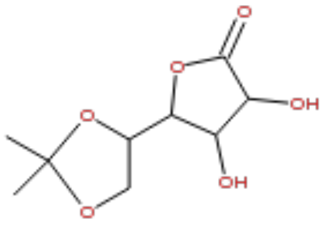

Cell ID	Cluster Center	Number of Compounds
407	0	9

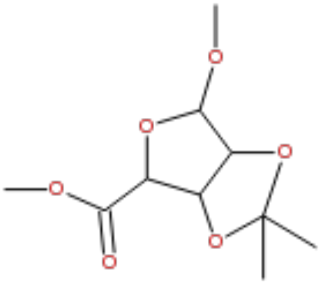

Cell ID	Cluster Center	Number of Compounds
407	0	9

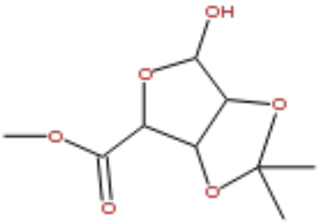

Cell ID	Cluster Center	Number of Compounds
407	0	9

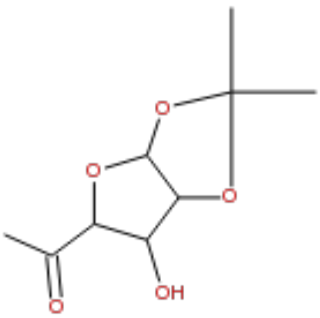

Cell ID	Cluster Center	Number of Compounds
407	0	9

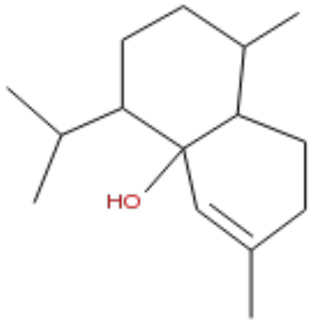

Cell ID	Cluster Center	Number of Compounds
408	1	4

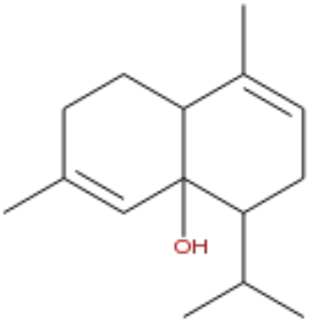

Cell ID	Cluster Center	Number of Compounds
408	0	4

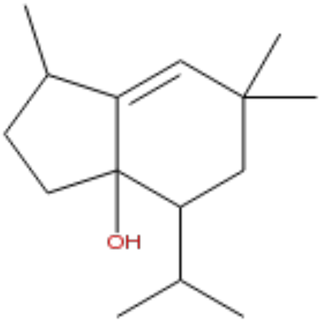

Cell ID	Cluster Center	Number of Compounds
408	0	4

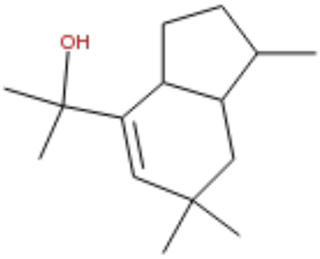

Cell ID	Cluster Center	Number of Compounds
408	0	4

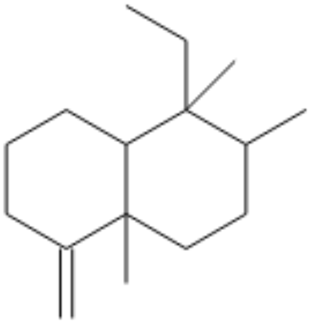

Cell ID	Cluster Center	Number of Compounds
409	1	1

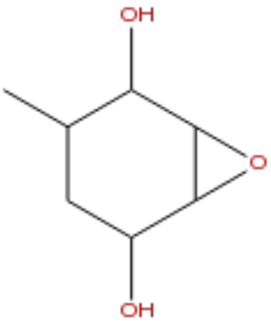

Cell ID	Cluster Center	Number of Compounds
412	1	3

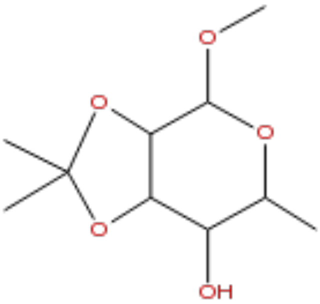

Cell ID	Cluster Center	Number of Compounds
412	0	3

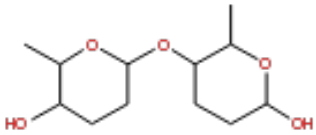

Cell ID	Cluster Center	Number of Compounds
412	0	3

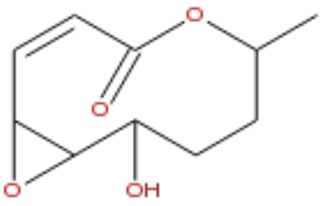

Cell ID	Cluster Center	Number of Compounds
413	1	1

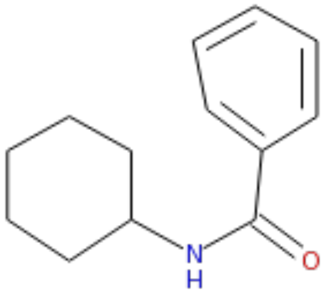

Cell ID	Cluster Center	Number of Compounds
415	1	3

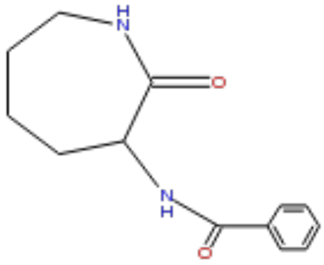

Cell ID	Cluster Center	Number of Compounds
415	0	3

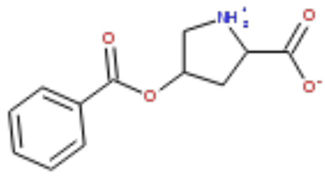

Cell ID	Cluster Center	Number of Compounds
415	0	3

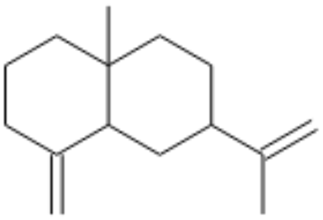

Cell ID	Cluster Center	Number of Compounds
419	1	18

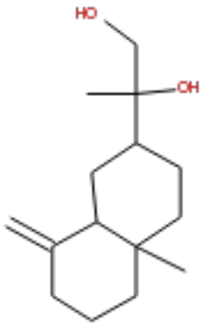

Cell ID	Cluster Center	Number of Compounds
419	0	18

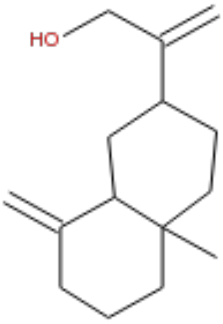

Cell ID	Cluster Center	Number of Compounds
419	0	18

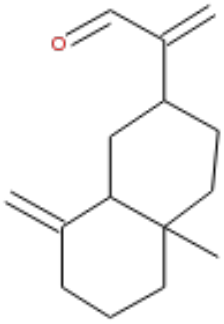

Cell ID	Cluster Center	Number of Compounds
419	0	18

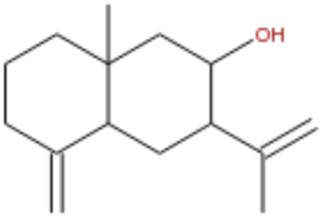

Cell ID	Cluster Center	Number of Compounds
419	0	18

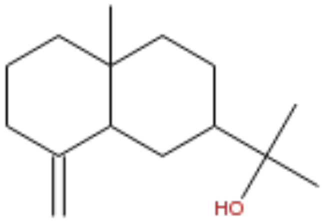

Cell ID	Cluster Center	Number of Compounds
419	0	18

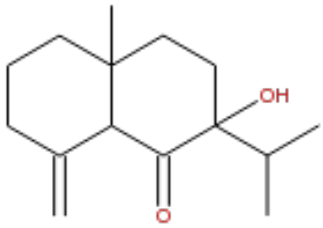

Cell ID	Cluster Center	Number of Compounds
419	0	18

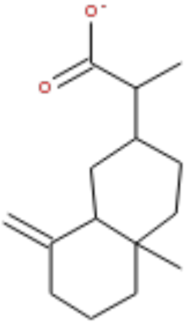

Cell ID	Cluster Center	Number of Compounds
419	0	18

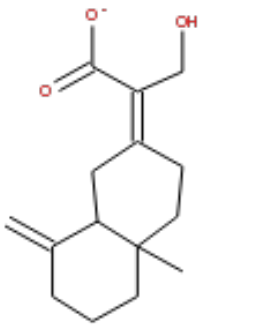

Cell ID	Cluster Center	Number of Compounds
419	0	18

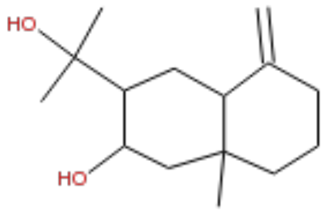

Cell ID	Cluster Center	Number of Compounds
419	0	18

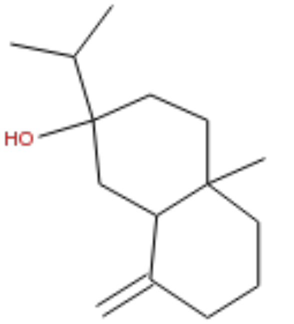

Cell ID	Cluster Center	Number of Compounds
419	0	18

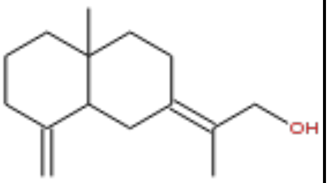

Cell ID	Cluster Center	Number of Compounds
419	0	18

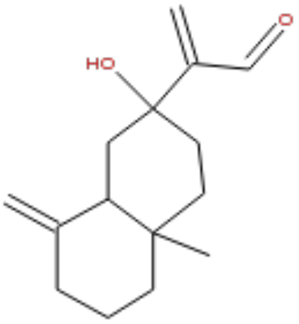

Cell ID	Cluster Center	Number of Compounds
419	0	18

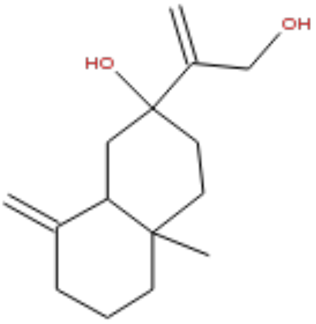

Cell ID	Cluster Center	Number of Compounds
419	0	18

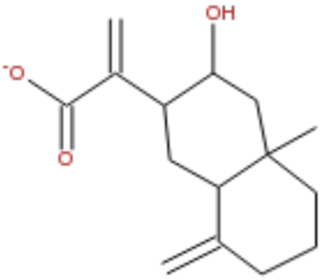

Cell ID	Cluster Center	Number of Compounds
419	0	18

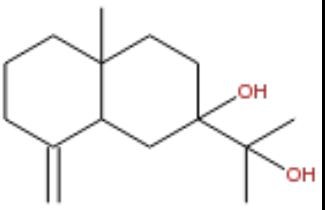

Cell ID	Cluster Center	Number of Compounds
419	0	18

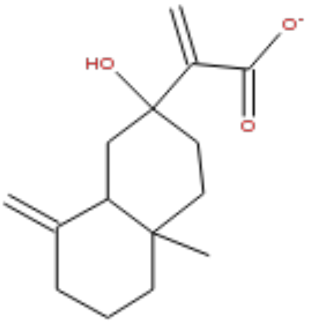

Cell ID	Cluster Center	Number of Compounds
419	0	18

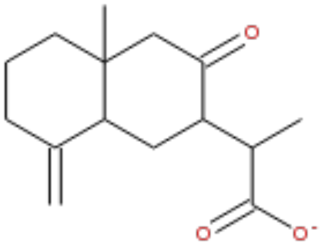

Cell ID	Cluster Center	Number of Compounds
419	0	18

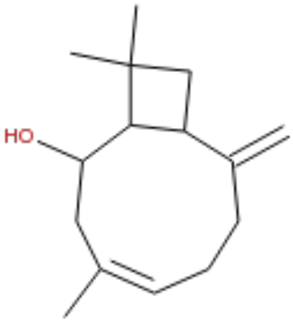

Cell ID	Cluster Center	Number of Compounds
420	1	5

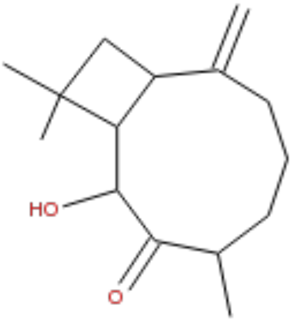

Cell ID	Cluster Center	Number of Compounds
420	0	5

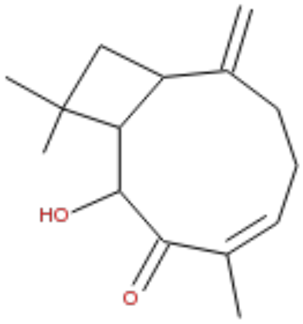

Cell ID	Cluster Center	Number of Compounds
420	0	5

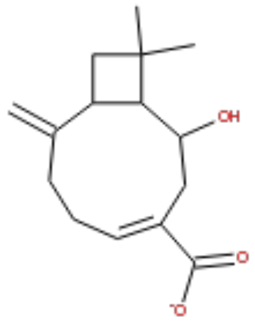

Cell ID	Cluster Center	Number of Compounds
420	0	5

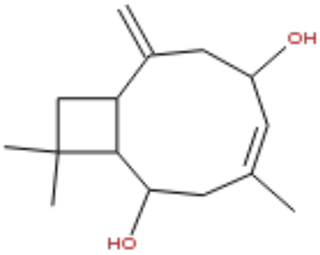

Cell ID	Cluster Center	Number of Compounds
420	0	5

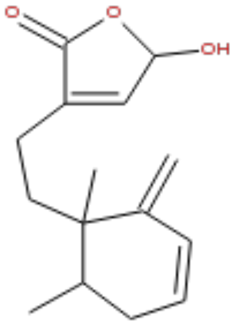

Cell ID	Cluster Center	Number of Compounds
422	1	4

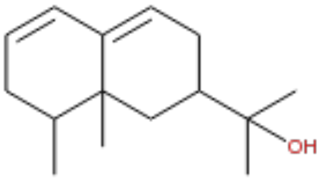

Cell ID	Cluster Center	Number of Compounds
422	0	4

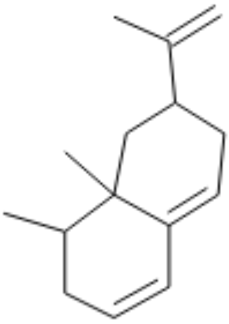

Cell ID	Cluster Center	Number of Compounds
422	0	4

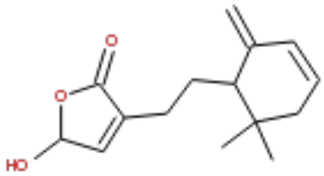

Cell ID	Cluster Center	Number of Compounds
422	0	4

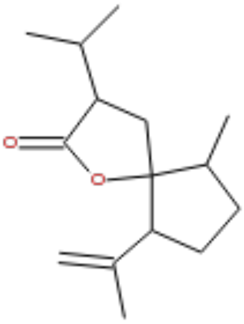

Cell ID	Cluster Center	Number of Compounds
426	1	2

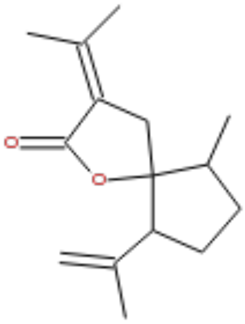

Cell ID	Cluster Center	Number of Compounds
426	0	2

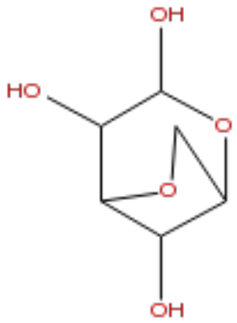

Cell ID	Cluster Center	Number of Compounds
427	1	18

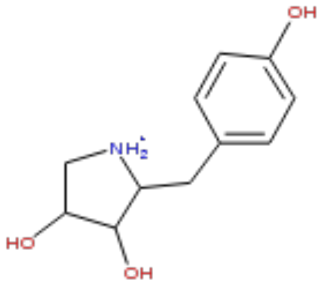

Cell ID	Cluster Center	Number of Compounds
427	0	18

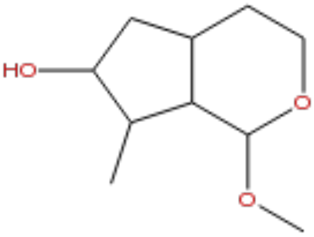

Cell ID	Cluster Center	Number of Compounds
427	0	18

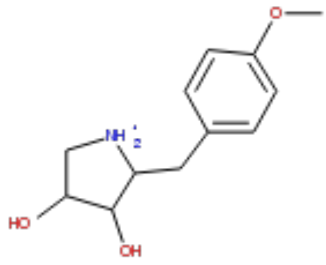

Cell ID	Cluster Center	Number of Compounds
427	0	18

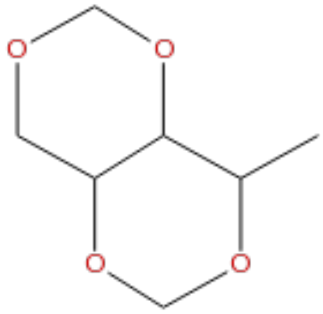

Cell ID	Cluster Center	Number of Compounds
427	0	18

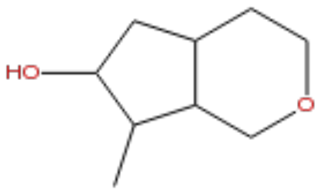

Cell ID	Cluster Center	Number of Compounds
427	0	18

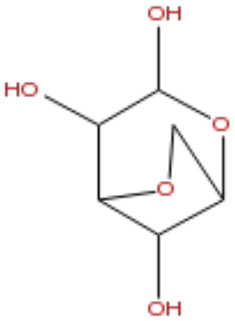

Cell ID	Cluster Center	Number of Compounds
427	0	18

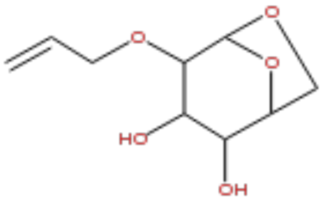

Cell ID	Cluster Center	Number of Compounds
427	0	18

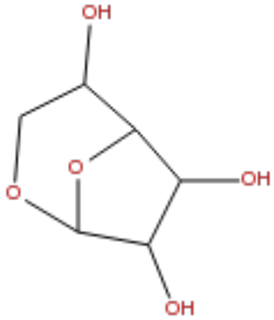

Cell ID	Cluster Center	Number of Compounds
427	0	18

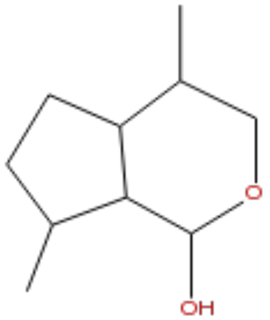

Cell ID	Cluster Center	Number of Compounds
427	0	18

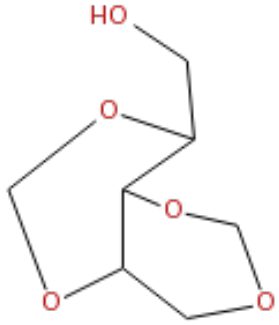

Cell ID	Cluster Center	Number of Compounds
427	0	18

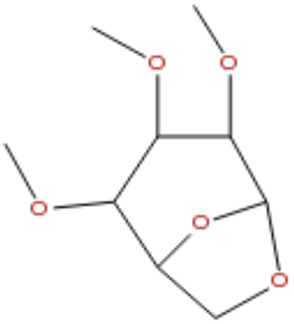

Cell ID	Cluster Center	Number of Compounds
427	0	18

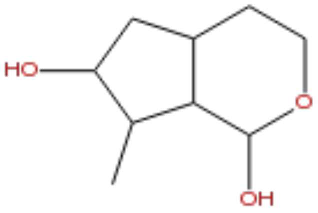

Cell ID	Cluster Center	Number of Compounds
427	0	18

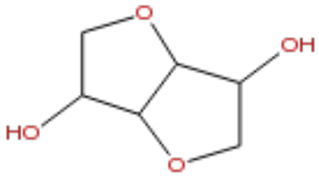

Cell ID	Cluster Center	Number of Compounds
427	0	18

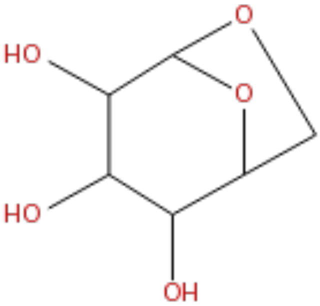

Cell ID	Cluster Center	Number of Compounds
427	0	18

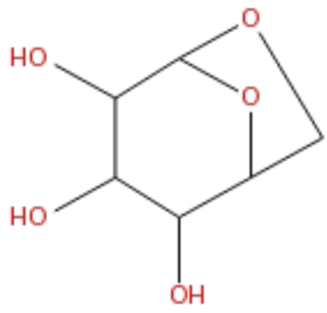

Cell ID	Cluster Center	Number of Compounds
427	0	18

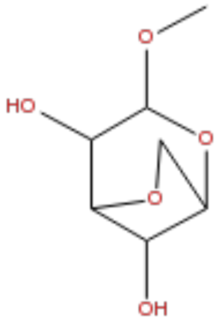

Cell ID	Cluster Center	Number of Compounds
427	0	18

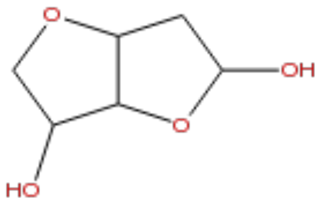

Cell ID	Cluster Center	Number of Compounds
427	0	18

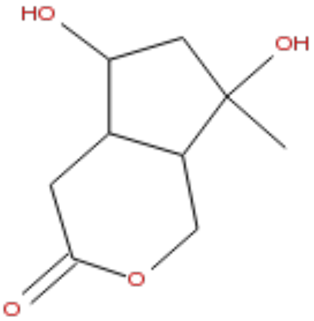

Cell ID	Cluster Center	Number of Compounds
429	1	34

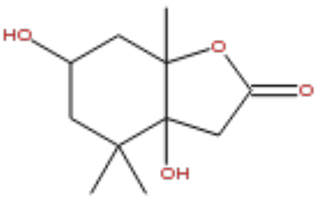

Cell ID	Cluster Center	Number of Compounds
429	0	34

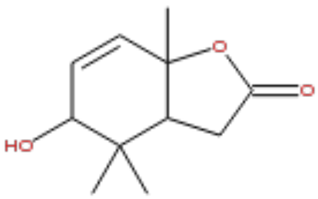

Cell ID	Cluster Center	Number of Compounds
429	0	34

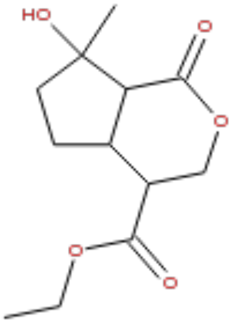

Cell ID	Cluster Center	Number of Compounds
429	0	34

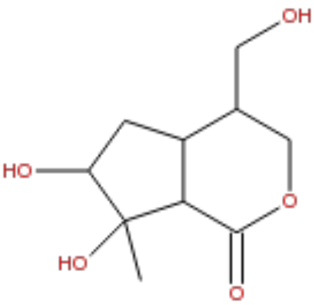

Cell ID	Cluster Center	Number of Compounds
429	0	34

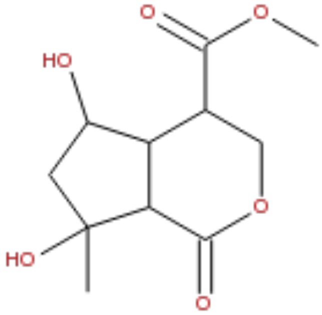

Cell ID	Cluster Center	Number of Compounds
429	0	34

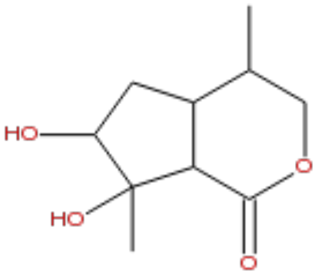

Cell ID	Cluster Center	Number of Compounds
429	0	34

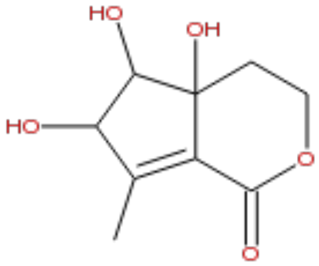

Cell ID	Cluster Center	Number of Compounds
429	0	34

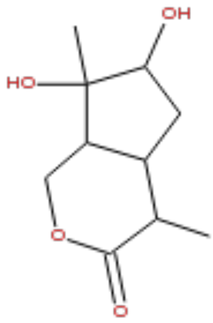

Cell ID	Cluster Center	Number of Compounds
429	0	34

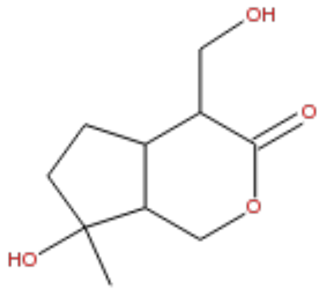

Cell ID	Cluster Center	Number of Compounds
429	0	34

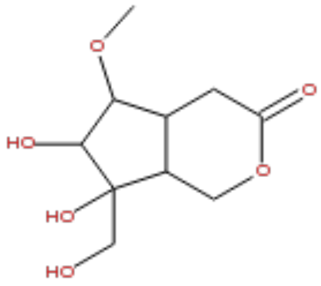

Cell ID	Cluster Center	Number of Compounds
429	0	34

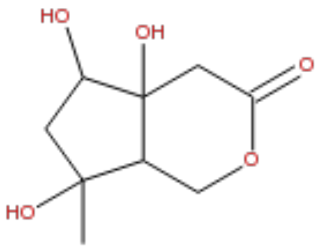

Cell ID	Cluster Center	Number of Compounds
429	0	34

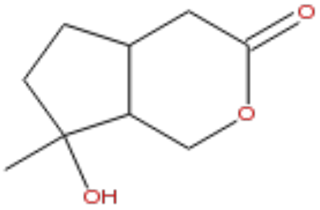

Cell ID	Cluster Center	Number of Compounds
429	0	34

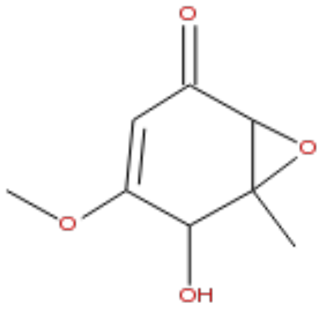

Cell ID	Cluster Center	Number of Compounds
429	0	34

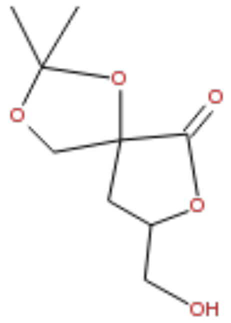

Cell ID	Cluster Center	Number of Compounds
429	0	34

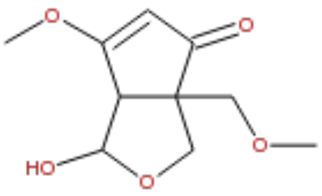

Cell ID	Cluster Center	Number of Compounds
429	0	34

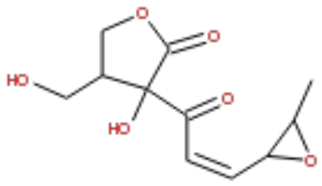

Cell ID	Cluster Center	Number of Compounds
429	0	34

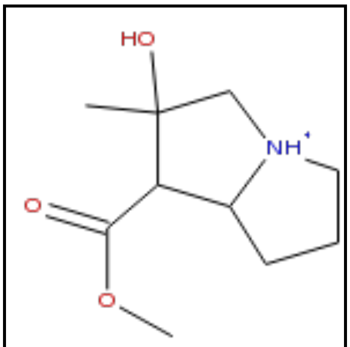

Cell ID	Cluster Center	Number of Compounds
429	0	34

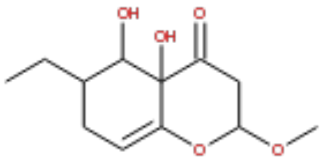

Cell ID	Cluster Center	Number of Compounds
429	0	34

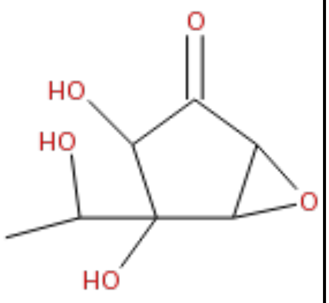

Cell ID	Cluster Center	Number of Compounds
429	0	34

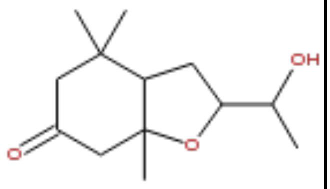

Cell ID	Cluster Center	Number of Compounds
429	0	34

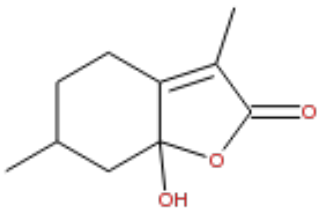

Cell ID	Cluster Center	Number of Compounds
429	0	34

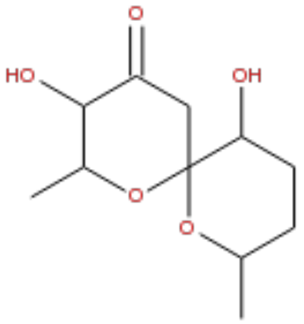

Cell ID	Cluster Center	Number of Compounds
429	0	34

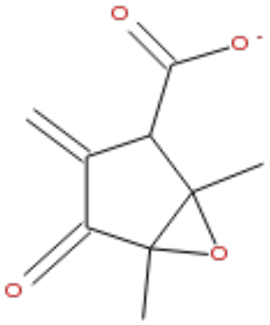

Cell ID	Cluster Center	Number of Compounds
429	0	34

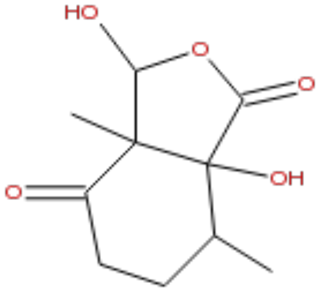

Cell ID	Cluster Center	Number of Compounds
429	0	34

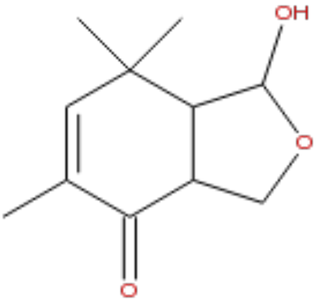

Cell ID	Cluster Center	Number of Compounds
429	0	34

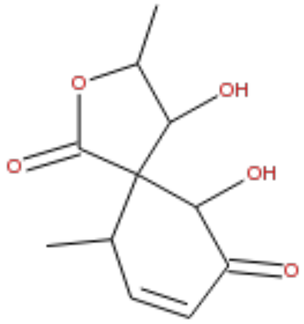

Cell ID	Cluster Center	Number of Compounds
429	0	34

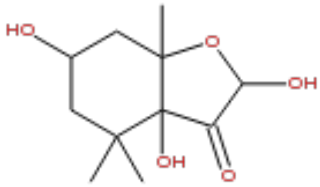

Cell ID	Cluster Center	Number of Compounds
429	0	34

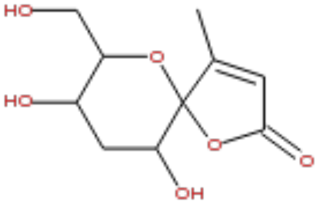

Cell ID	Cluster Center	Number of Compounds
429	0	34

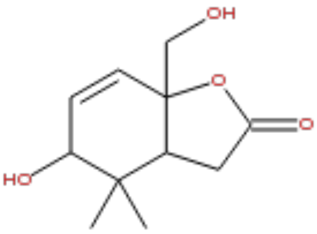

Cell ID	Cluster Center	Number of Compounds
429	0	34

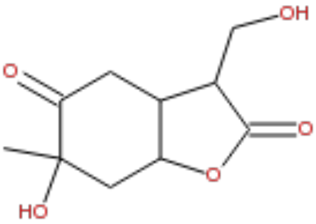

Cell ID	Cluster Center	Number of Compounds
429	0	34

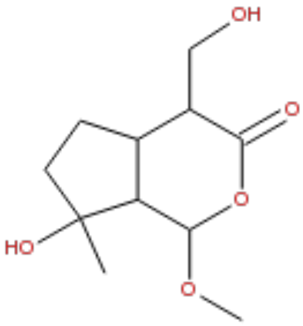

Cell ID	Cluster Center	Number of Compounds
429	0	34

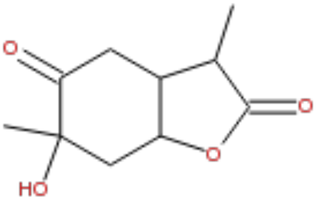

Cell ID	Cluster Center	Number of Compounds
429	0	34

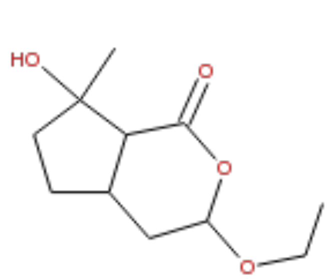

Cell ID	Cluster Center	Number of Compounds
429	0	34

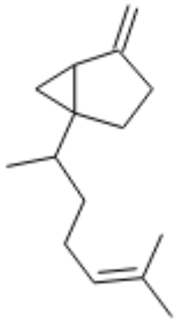

Cell ID	Cluster Center	Number of Compounds
430	1	5

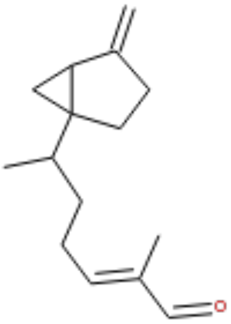

Cell ID	Cluster Center	Number of Compounds
430	0	5

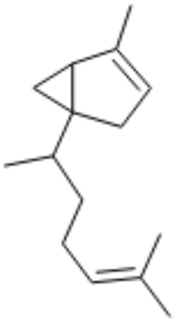

Cell ID	Cluster Center	Number of Compounds
430	0	5

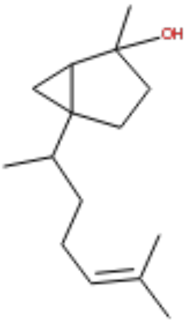

Cell ID	Cluster Center	Number of Compounds
430	0	5

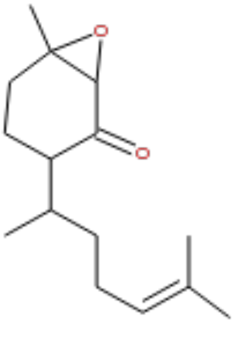

Cell ID	Cluster Center	Number of Compounds
430	0	5

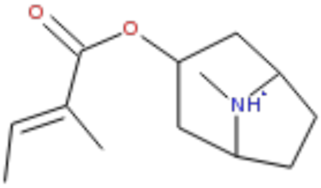

Cell ID	Cluster Center	Number of Compounds
432	1	5

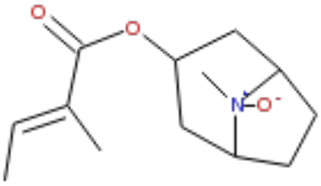

Cell ID	Cluster Center	Number of Compounds
432	0	5

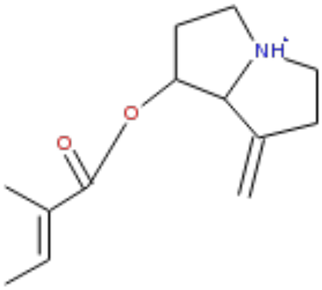

Cell ID	Cluster Center	Number of Compounds
432	0	5

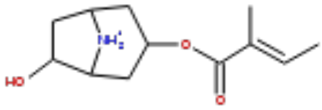

Cell ID	Cluster Center	Number of Compounds
432	0	5

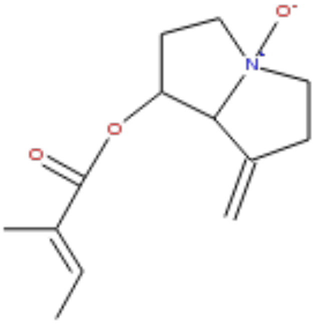

Cell ID	Cluster Center	Number of Compounds
432	0	5

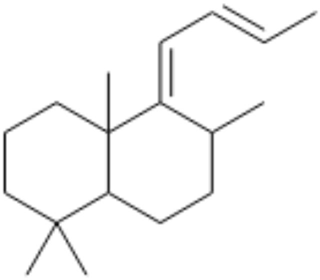

Cell ID	Cluster Center	Number of Compounds
433	1	1

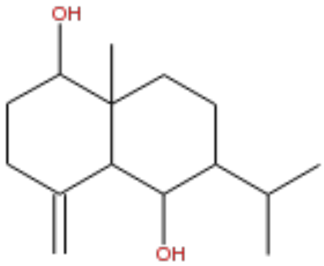

Cell ID	Cluster Center	Number of Compounds
434	1	13

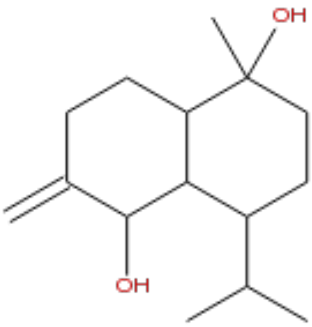

Cell ID	Cluster Center	Number of Compounds
434	0	13

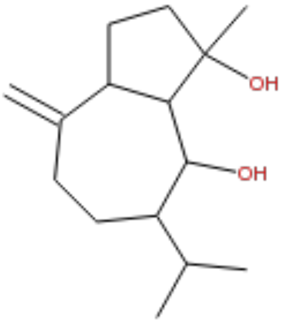

Cell ID	Cluster Center	Number of Compounds
434	0	13

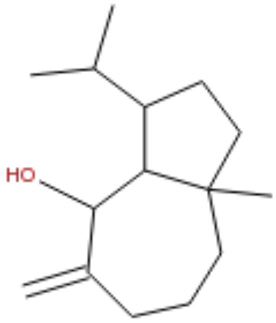

Cell ID	Cluster Center	Number of Compounds
434	0	13

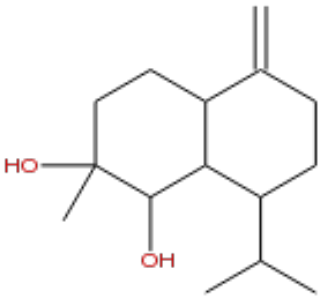

Cell ID	Cluster Center	Number of Compounds
434	0	13

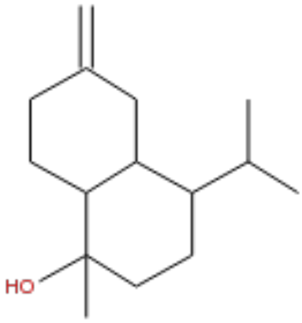

Cell ID	Cluster Center	Number of Compounds
434	0	13

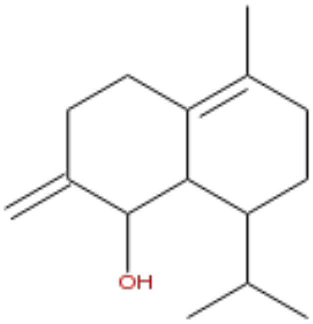

Cell ID	Cluster Center	Number of Compounds
434	0	13

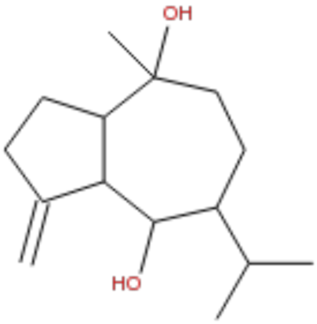

Cell ID	Cluster Center	Number of Compounds
434	0	13

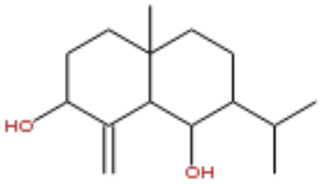

Cell ID	Cluster Center	Number of Compounds
434	0	13

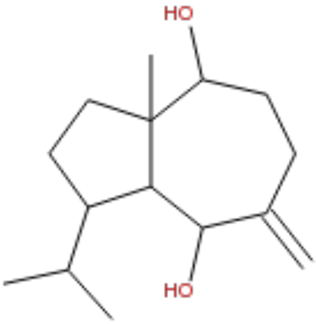

Cell ID	Cluster Center	Number of Compounds
434	0	13

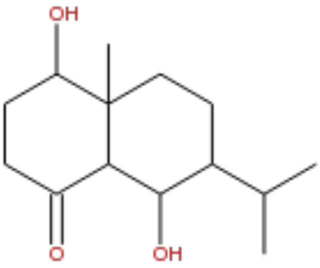

Cell ID	Cluster Center	Number of Compounds
434	0	13

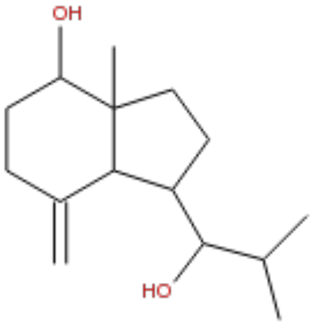

Cell ID	Cluster Center	Number of Compounds
434	0	13

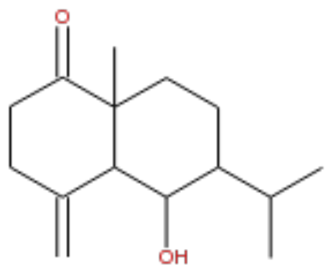

Cell ID	Cluster Center	Number of Compounds
434	0	13

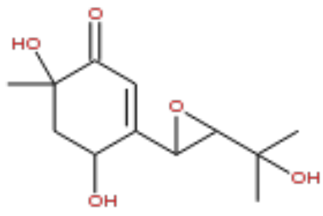

Cell ID	Cluster Center	Number of Compounds
436	1	2

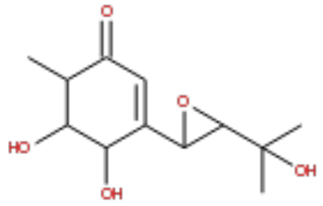

Cell ID	Cluster Center	Number of Compounds
436	0	2

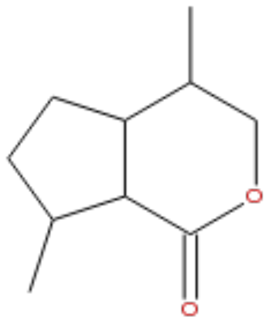

Cell ID	Cluster Center	Number of Compounds
437	1	27

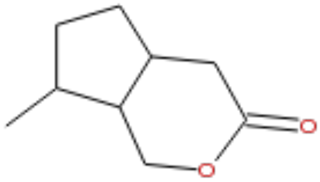

Cell ID	Cluster Center	Number of Compounds
437	0	27

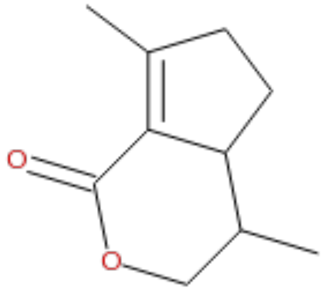

Cell ID	Cluster Center	Number of Compounds
437	0	27

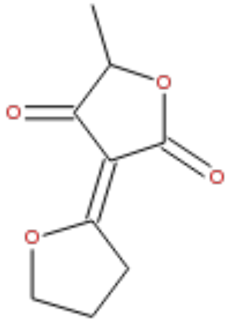

Cell ID	Cluster Center	Number of Compounds
437	0	27

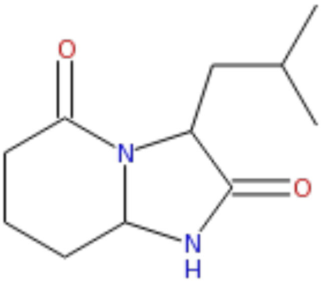

Cell ID	Cluster Center	Number of Compounds
437	0	27

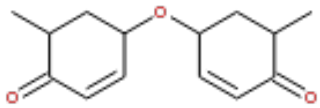

Cell ID	Cluster Center	Number of Compounds
437	0	27

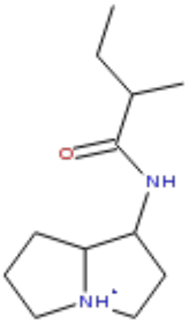

Cell ID	Cluster Center	Number of Compounds
437	0	27

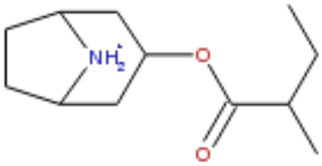

Cell ID	Cluster Center	Number of Compounds
437	0	27

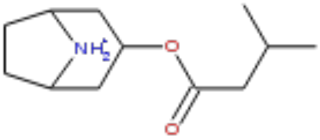

Cell ID	Cluster Center	Number of Compounds
437	0	27

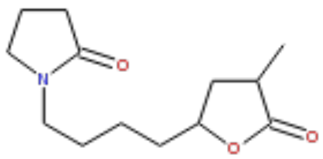

Cell ID	Cluster Center	Number of Compounds
437	0	27

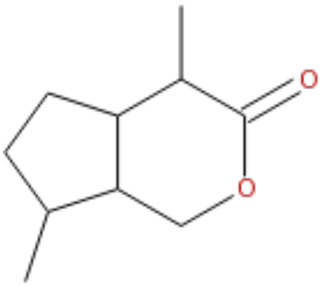

Cell ID	Cluster Center	Number of Compounds
437	0	27

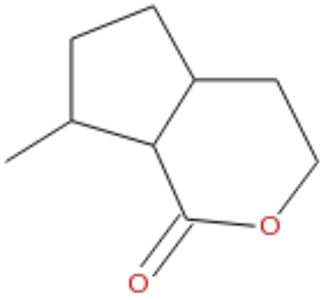

Cell ID	Cluster Center	Number of Compounds
437	0	27

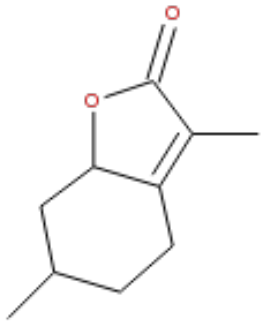

Cell ID	Cluster Center	Number of Compounds
437	0	27

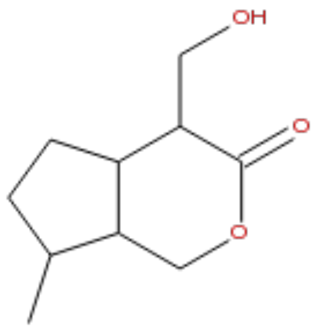

Cell ID	Cluster Center	Number of Compounds
437	0	27

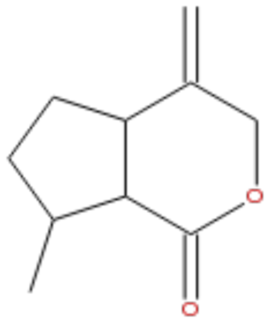

Cell ID	Cluster Center	Number of Compounds
437	0	27

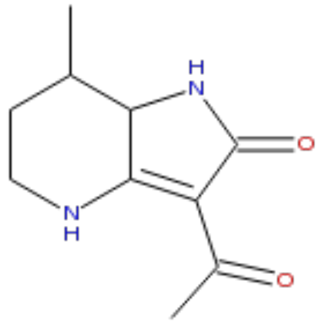

Cell ID	Cluster Center	Number of Compounds
437	0	27

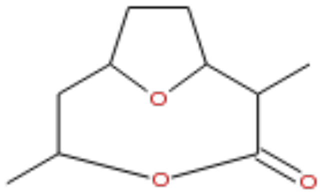

Cell ID	Cluster Center	Number of Compounds
437	0	27

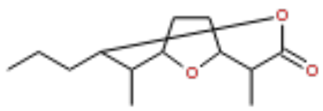

Cell ID	Cluster Center	Number of Compounds
437	0	27

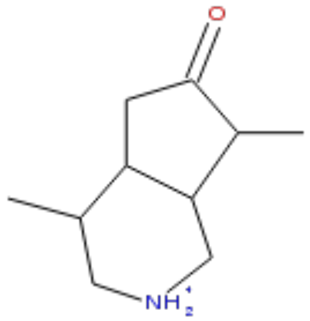

Cell ID	Cluster Center	Number of Compounds
437	0	27

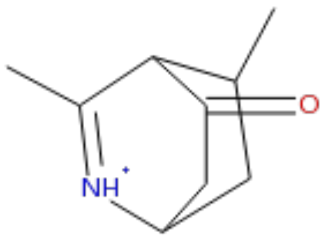

Cell ID	Cluster Center	Number of Compounds
437	0	27

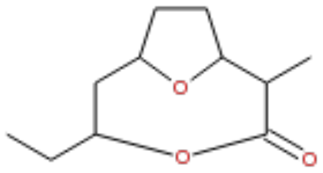

Cell ID	Cluster Center	Number of Compounds
437	0	27

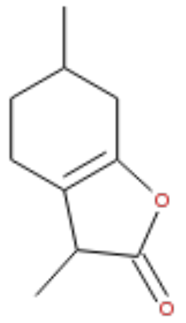

Cell ID	Cluster Center	Number of Compounds
437	0	27

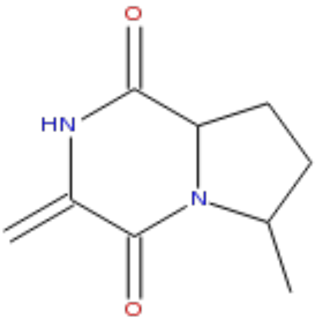

Cell ID	Cluster Center	Number of Compounds
437	0	27

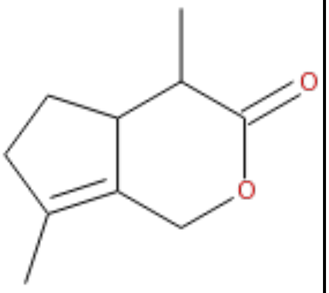

Cell ID	Cluster Center	Number of Compounds
437	0	27

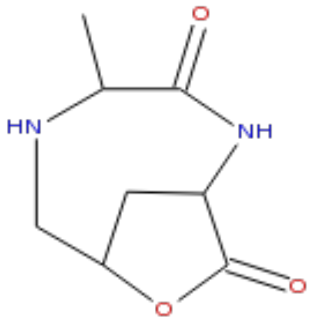

Cell ID	Cluster Center	Number of Compounds
437	0	27

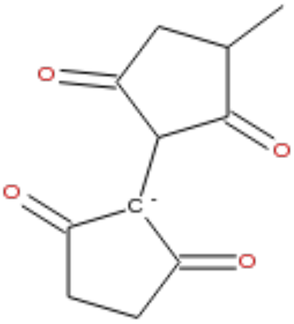

Cell ID	Cluster Center	Number of Compounds
437	0	27

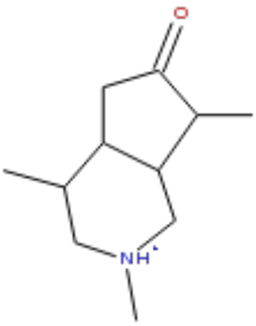

Cell ID	Cluster Center	Number of Compounds
437	0	27

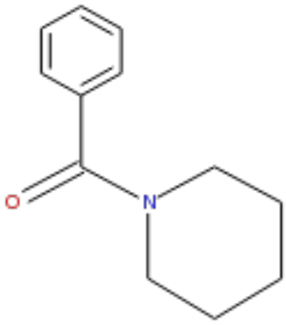

Cell ID	Cluster Center	Number of Compounds
440	1	7

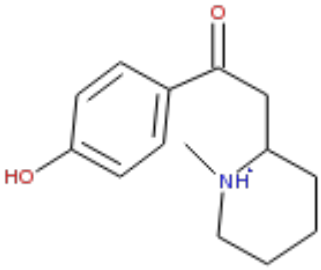

Cell ID	Cluster Center	Number of Compounds
440	0	7

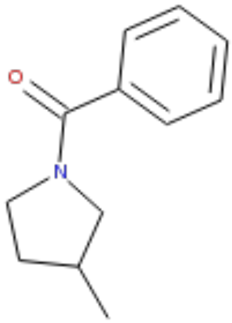

Cell ID	Cluster Center	Number of Compounds
440	0	7

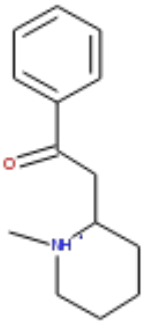

Cell ID	Cluster Center	Number of Compounds
440	0	7

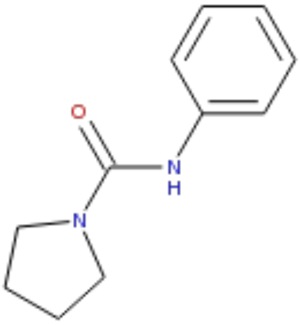

Cell ID	Cluster Center	Number of Compounds
440	0	7

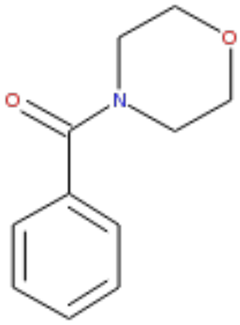

Cell ID	Cluster Center	Number of Compounds
440	0	7

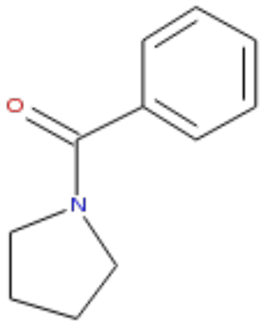

Cell ID	Cluster Center	Number of Compounds
440	0	7

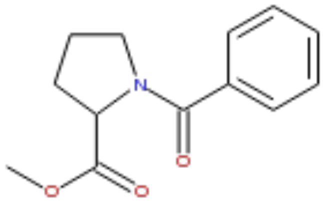

Cell ID	Cluster Center	Number of Compounds
441	1	5

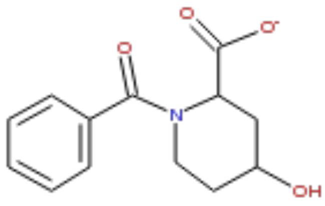

Cell ID	Cluster Center	Number of Compounds
441	0	5

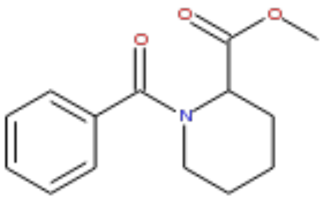

Cell ID	Cluster Center	Number of Compounds
441	0	5

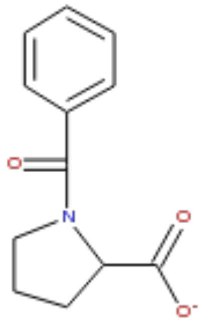

Cell ID	Cluster Center	Number of Compounds
441	0	5

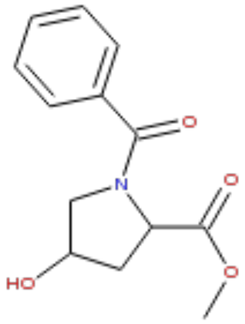

Cell ID	Cluster Center	Number of Compounds
441	0	5

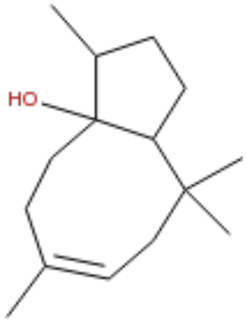

Cell ID	Cluster Center	Number of Compounds
443	1	25

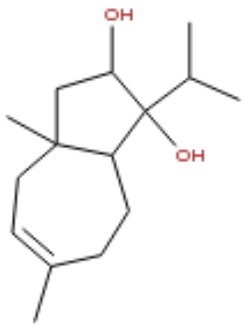

Cell ID	Cluster Center	Number of Compounds
443	0	25

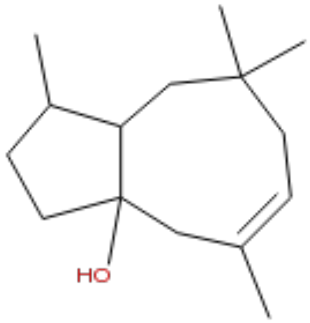

Cell ID	Cluster Center	Number of Compounds
443	0	25

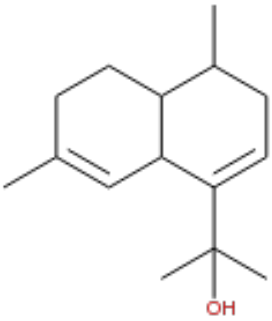

Cell ID	Cluster Center	Number of Compounds
443	0	25

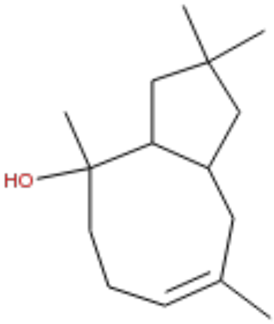

Cell ID	Cluster Center	Number of Compounds
443	0	25

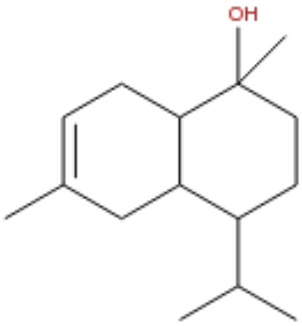

Cell ID	Cluster Center	Number of Compounds
443	0	25

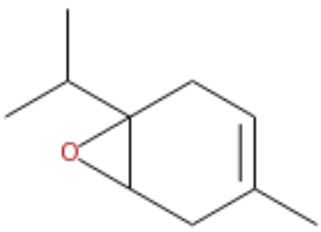

Cell ID	Cluster Center	Number of Compounds
443	0	25

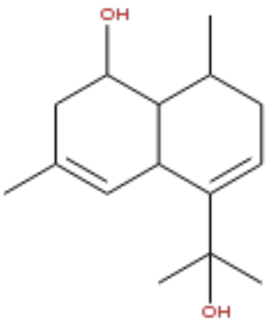

Cell ID	Cluster Center	Number of Compounds
443	0	25

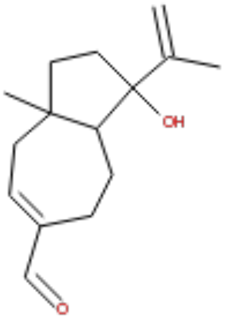

Cell ID	Cluster Center	Number of Compounds
443	0	25

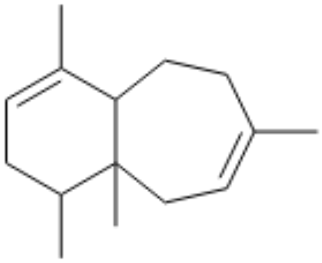

Cell ID	Cluster Center	Number of Compounds
443	0	25

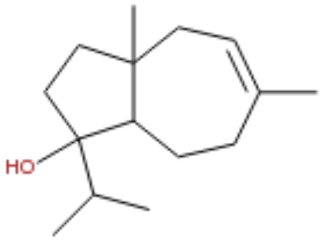

Cell ID	Cluster Center	Number of Compounds
443	0	25

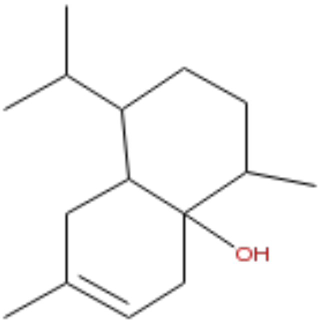

Cell ID	Cluster Center	Number of Compounds
443	0	25

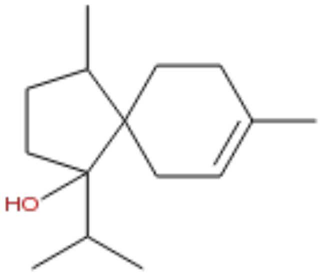

Cell ID	Cluster Center	Number of Compounds
443	0	25

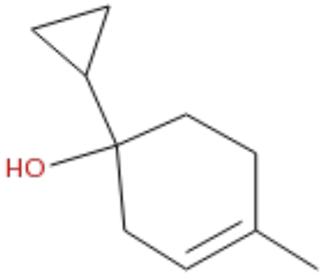

Cell ID	Cluster Center	Number of Compounds
443	0	25

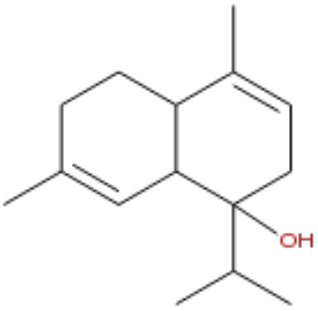

Cell ID	Cluster Center	Number of Compounds
443	0	25

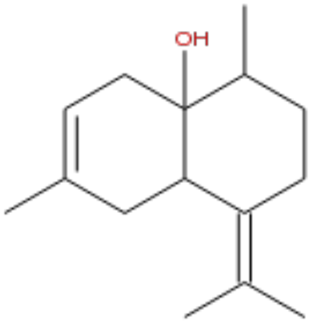

Cell ID	Cluster Center	Number of Compounds
443	0	25

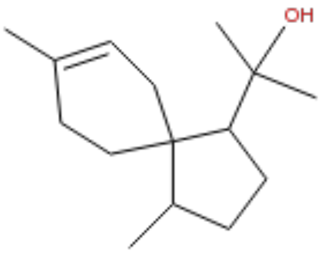

Cell ID	Cluster Center	Number of Compounds
443	0	25

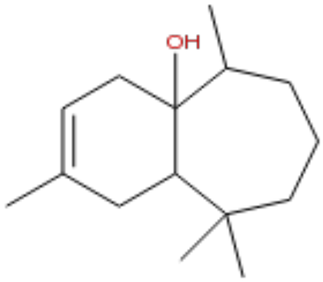

Cell ID	Cluster Center	Number of Compounds
443	0	25

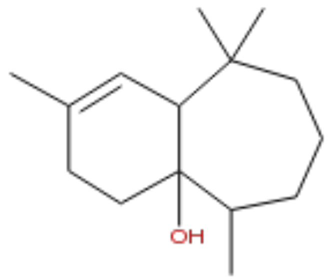

Cell ID	Cluster Center	Number of Compounds
443	0	25

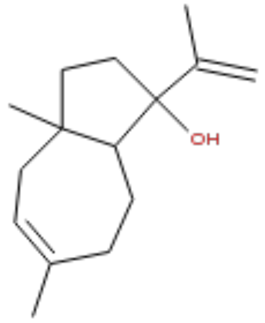

Cell ID	Cluster Center	Number of Compounds
443	0	25

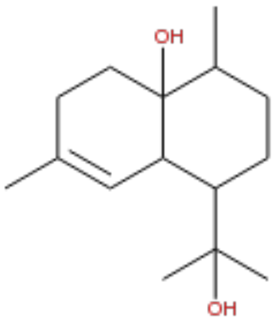

Cell ID	Cluster Center	Number of Compounds
443	0	25

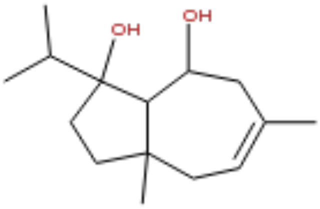

Cell ID	Cluster Center	Number of Compounds
443	0	25

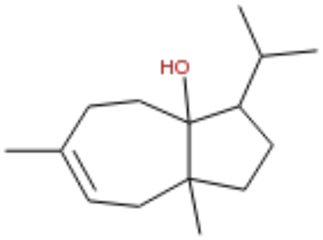

Cell ID	Cluster Center	Number of Compounds
443	0	25

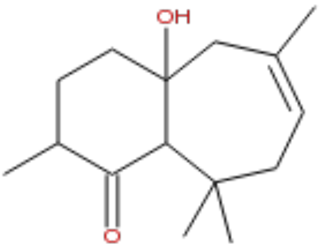

Cell ID	Cluster Center	Number of Compounds
443	0	25

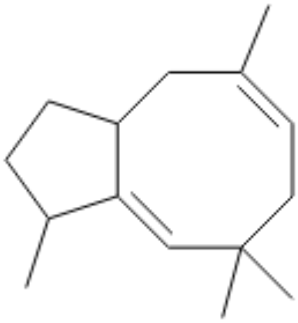

Cell ID	Cluster Center	Number of Compounds
443	0	25

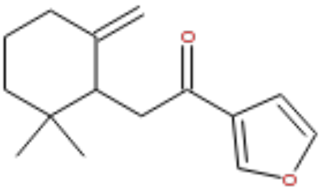

Cell ID	Cluster Center	Number of Compounds
444	1	1

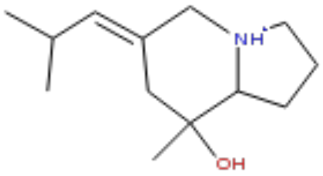

Cell ID	Cluster Center	Number of Compounds
445	1	5

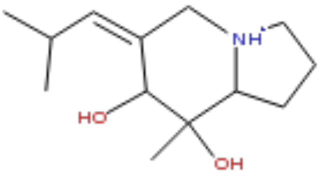

Cell ID	Cluster Center	Number of Compounds
445	0	5

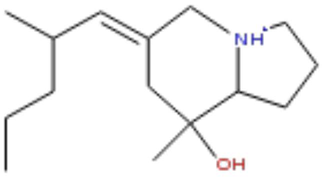

Cell ID	Cluster Center	Number of Compounds
445	0	5

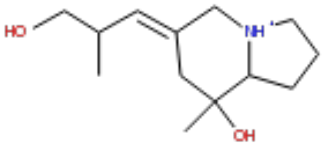

Cell ID	Cluster Center	Number of Compounds
445	0	5

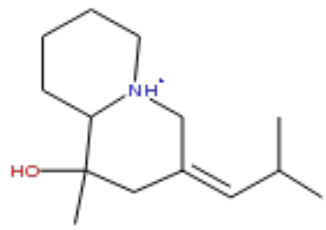

Cell ID	Cluster Center	Number of Compounds
445	0	5

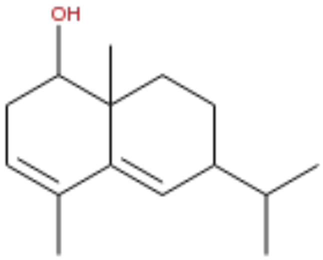

Cell ID	Cluster Center	Number of Compounds
446	1	9

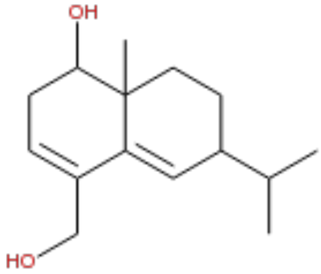

Cell ID	Cluster Center	Number of Compounds
446	0	9

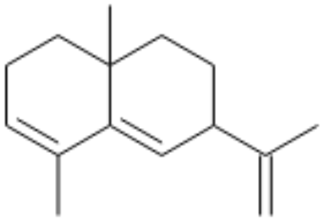

Cell ID	Cluster Center	Number of Compounds
446	0	9

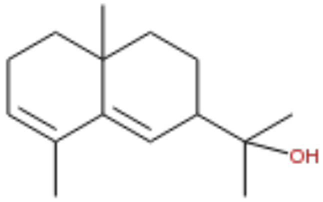

Cell ID	Cluster Center	Number of Compounds
446	0	9

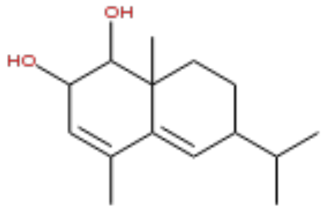

Cell ID	Cluster Center	Number of Compounds
446	0	9

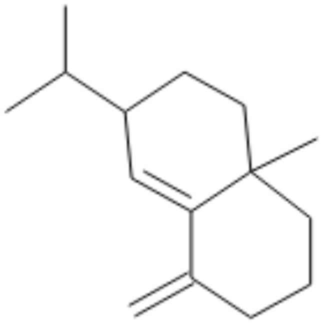

Cell ID	Cluster Center	Number of Compounds
446	0	9

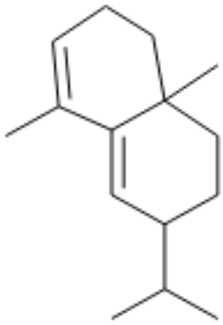

Cell ID	Cluster Center	Number of Compounds
446	0	9

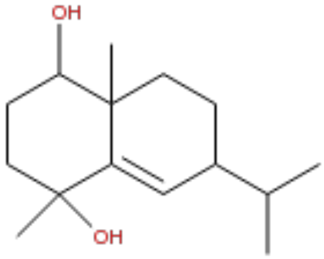

Cell ID	Cluster Center	Number of Compounds
446	0	9

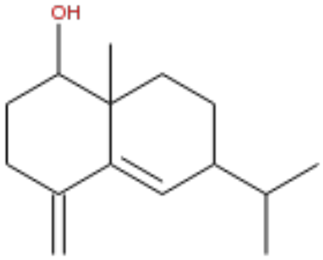

Cell ID	Cluster Center	Number of Compounds
446	0	9

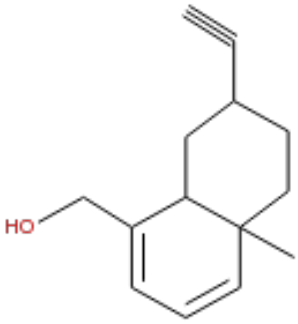

Cell ID	Cluster Center	Number of Compounds
447	1	7

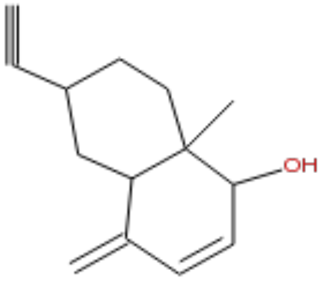

Cell ID	Cluster Center	Number of Compounds
447	0	7

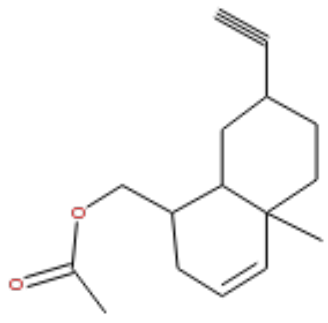

Cell ID	Cluster Center	Number of Compounds
447	0	7

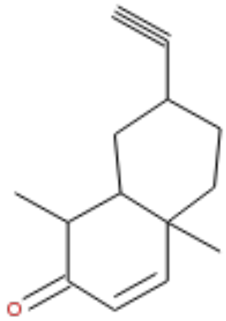

Cell ID	Cluster Center	Number of Compounds
447	0	7

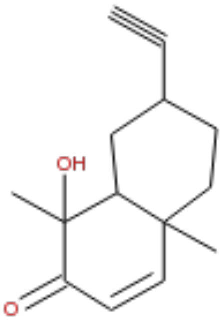

Cell ID	Cluster Center	Number of Compounds
447	0	7

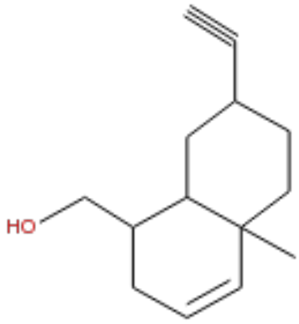

Cell ID	Cluster Center	Number of Compounds
447	0	7

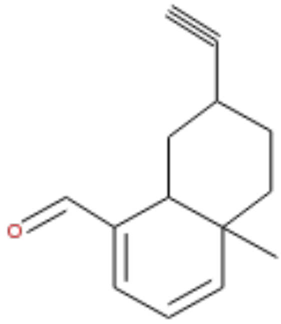

Cell ID	Cluster Center	Number of Compounds
447	0	7

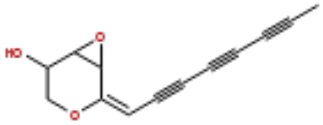

Cell ID	Cluster Center	Number of Compounds
449	1	3

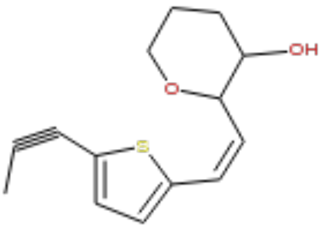

Cell ID	Cluster Center	Number of Compounds
449	0	3

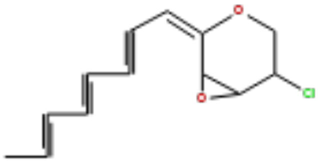

Cell ID	Cluster Center	Number of Compounds
449	0	3

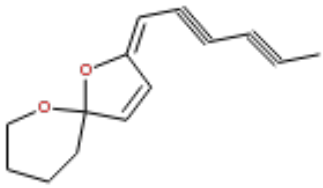

Cell ID	Cluster Center	Number of Compounds
450	1	4

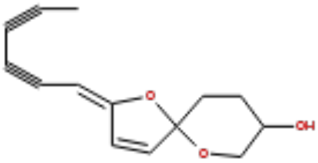

Cell ID	Cluster Center	Number of Compounds
450	0	4

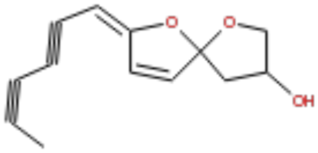

Cell ID	Cluster Center	Number of Compounds
450	0	4

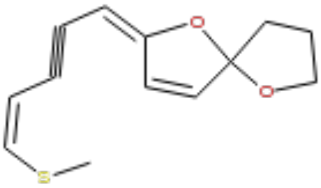

Cell ID	Cluster Center	Number of Compounds
450	0	4

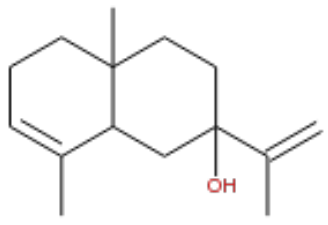

Cell ID	Cluster Center	Number of Compounds
451	1	15

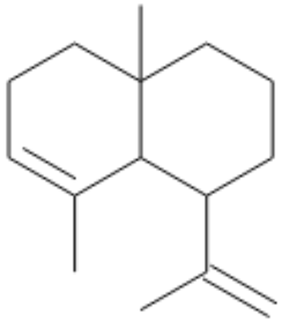

Cell ID	Cluster Center	Number of Compounds
451	0	15

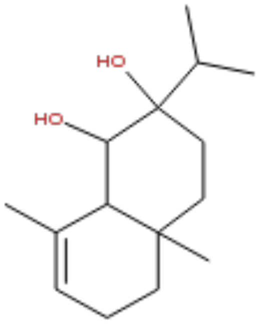

Cell ID	Cluster Center	Number of Compounds
451	0	15

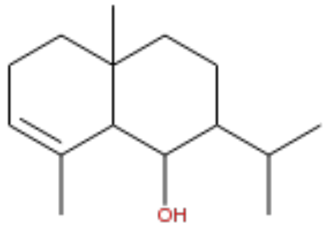

Cell ID	Cluster Center	Number of Compounds
451	0	15

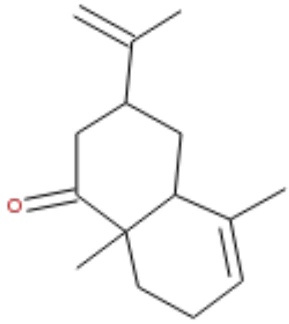

Cell ID	Cluster Center	Number of Compounds
451	0	15

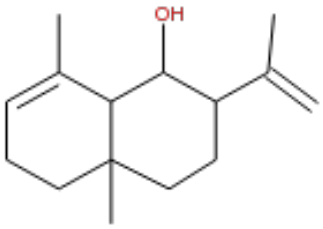

Cell ID	Cluster Center	Number of Compounds
451	0	15

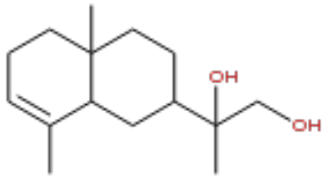

Cell ID	Cluster Center	Number of Compounds
451	0	15

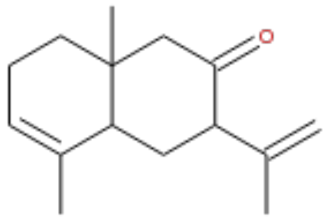

Cell ID	Cluster Center	Number of Compounds
451	0	15

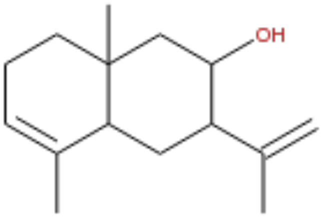

Cell ID	Cluster Center	Number of Compounds
451	0	15

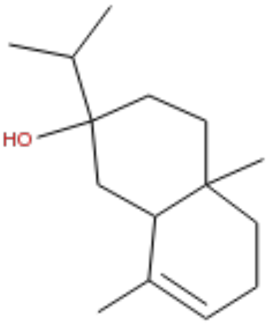

Cell ID	Cluster Center	Number of Compounds
451	0	15

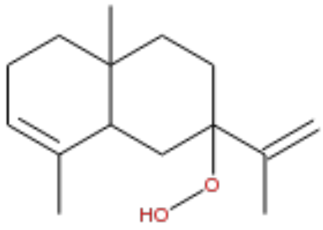

Cell ID	Cluster Center	Number of Compounds
451	0	15

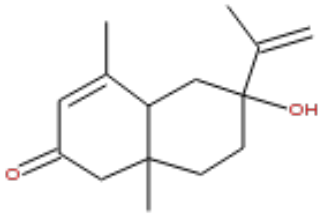

Cell ID	Cluster Center	Number of Compounds
451	0	15

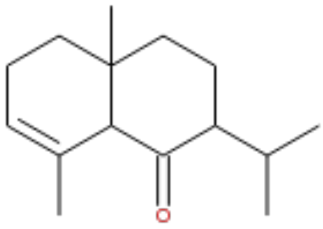

Cell ID	Cluster Center	Number of Compounds
451	0	15

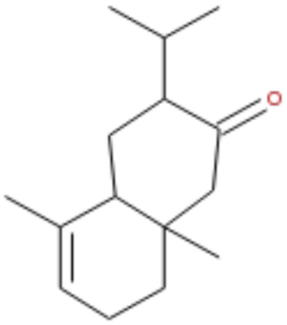

Cell ID	Cluster Center	Number of Compounds
451	0	15

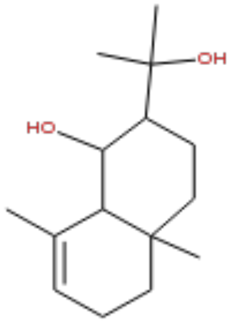

Cell ID	Cluster Center	Number of Compounds
451	0	15

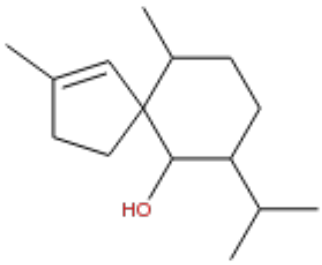

Cell ID	Cluster Center	Number of Compounds
452	1	8

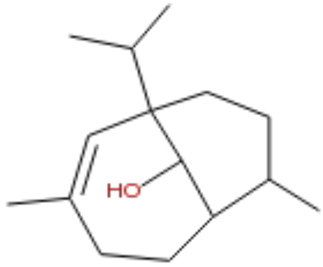

Cell ID	Cluster Center	Number of Compounds
452	0	8

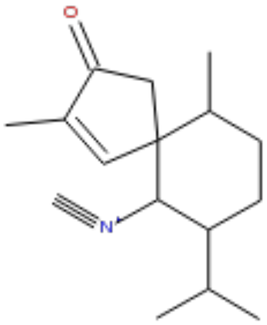

Cell ID	Cluster Center	Number of Compounds
452	0	8

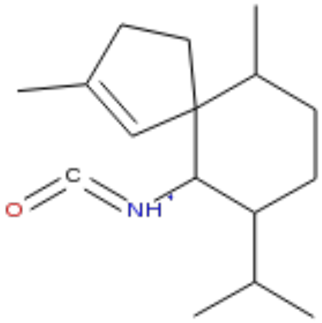

Cell ID	Cluster Center	Number of Compounds
452	0	8

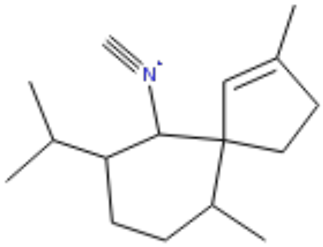

Cell ID	Cluster Center	Number of Compounds
452	0	8

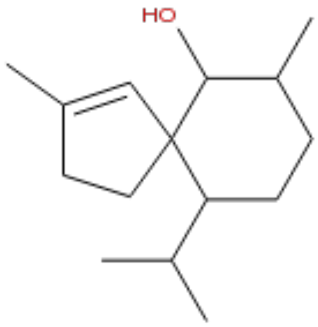

Cell ID	Cluster Center	Number of Compounds
452	0	8

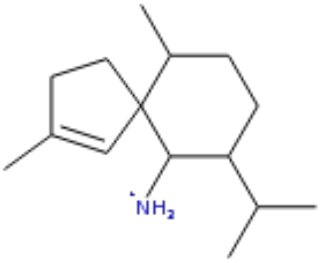

Cell ID	Cluster Center	Number of Compounds
452	0	8

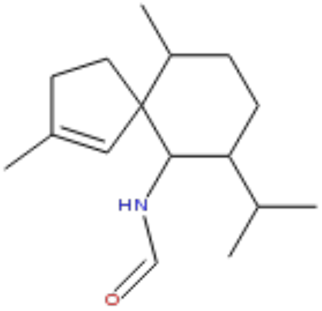

Cell ID	Cluster Center	Number of Compounds
452	0	8

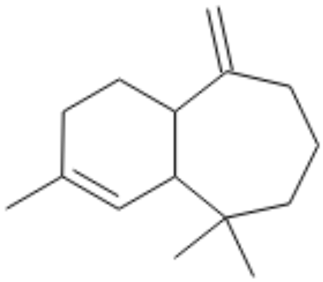

Cell ID	Cluster Center	Number of Compounds
453	1	5

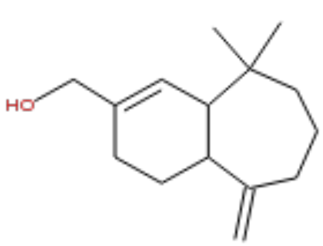

Cell ID	Cluster Center	Number of Compounds
453	0	5

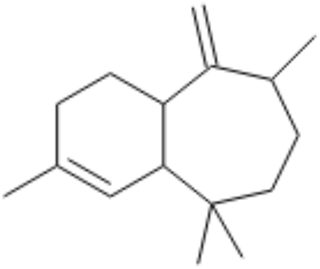

Cell ID	Cluster Center	Number of Compounds
453	0	5

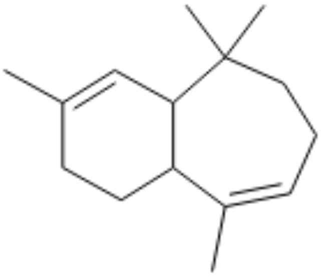

Cell ID	Cluster Center	Number of Compounds
453	0	5

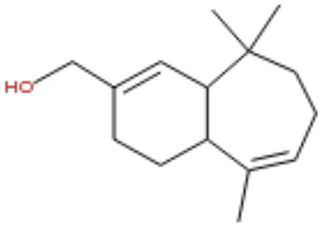

Cell ID	Cluster Center	Number of Compounds
453	0	5

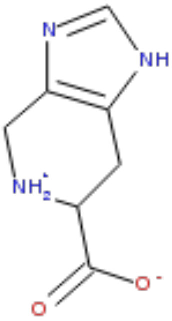

Cell ID	Cluster Center	Number of Compounds
454	1	14

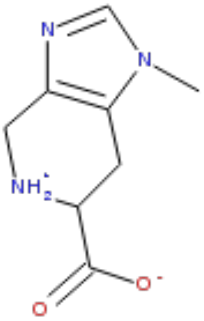

Cell ID	Cluster Center	Number of Compounds
454	0	14

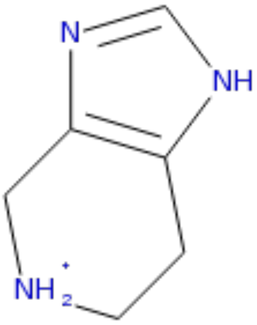

Cell ID	Cluster Center	Number of Compounds
454	0	14

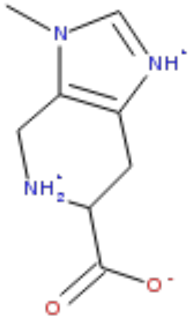

Cell ID	Cluster Center	Number of Compounds
454	0	14

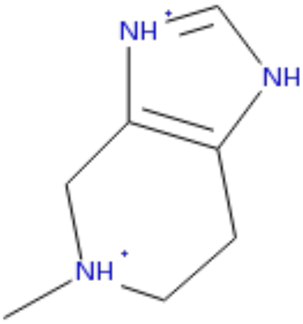

Cell ID	Cluster Center	Number of Compounds
454	0	14

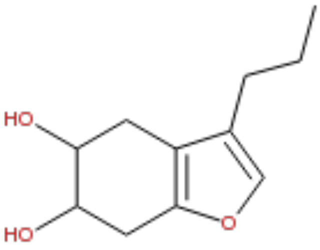

Cell ID	Cluster Center	Number of Compounds
454	0	14

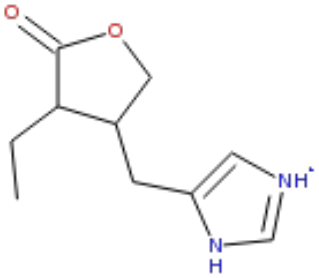

Cell ID	Cluster Center	Number of Compounds
454	0	14

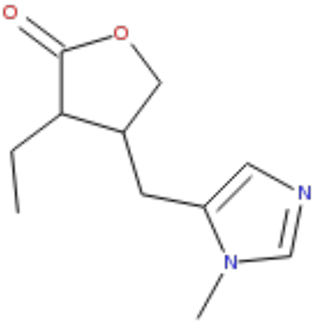

Cell ID	Cluster Center	Number of Compounds
454	0	14

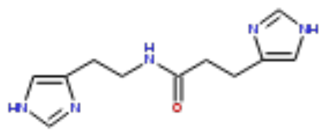

Cell ID	Cluster Center	Number of Compounds
454	0	14

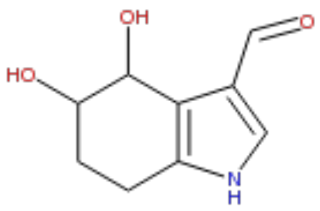

Cell ID	Cluster Center	Number of Compounds
454	0	14

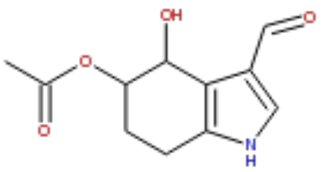

Cell ID	Cluster Center	Number of Compounds
454	0	14

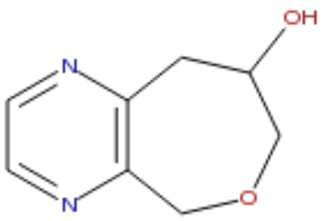

Cell ID	Cluster Center	Number of Compounds
454	0	14

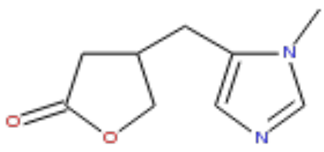

Cell ID	Cluster Center	Number of Compounds
454	0	14

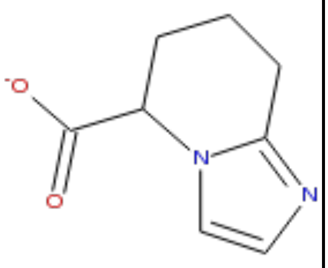

Cell ID	Cluster Center	Number of Compounds
454	0	14

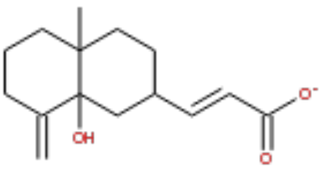

Cell ID	Cluster Center	Number of Compounds
455	1	1

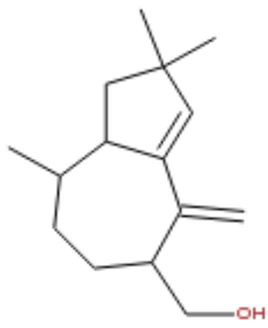

Cell ID	Cluster Center	Number of Compounds
456	1	1

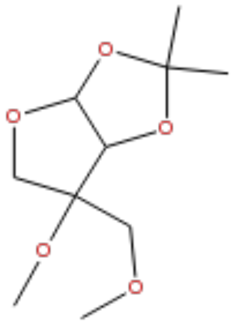

Cell ID	Cluster Center	Number of Compounds
457	1	5

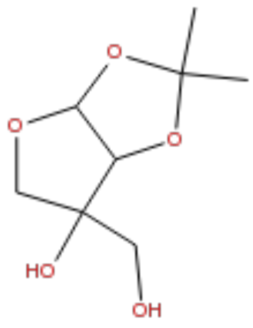

Cell ID	Cluster Center	Number of Compounds
457	0	5

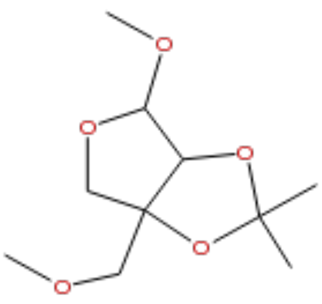

Cell ID	Cluster Center	Number of Compounds
457	0	5

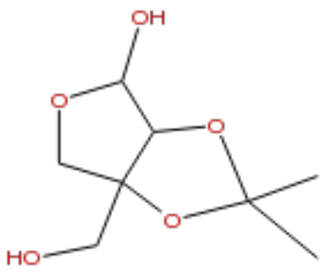

Cell ID	Cluster Center	Number of Compounds
457	0	5

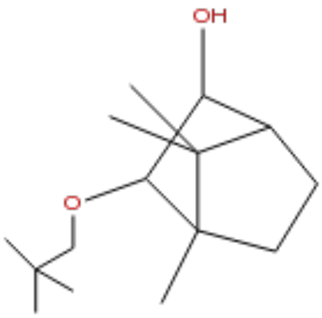

Cell ID	Cluster Center	Number of Compounds
457	0	5

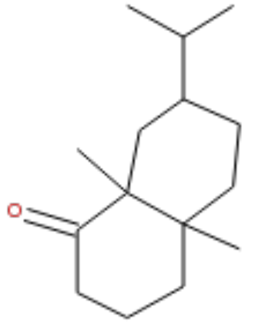

Cell ID	Cluster Center	Number of Compounds
458	1	10

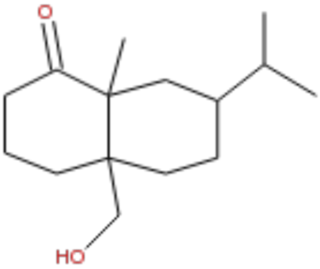

Cell ID	Cluster Center	Number of Compounds
458	0	10

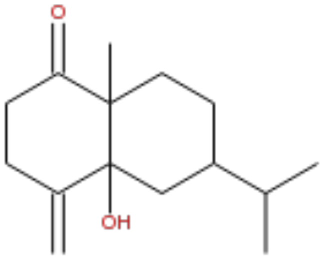

Cell ID	Cluster Center	Number of Compounds
458	0	10

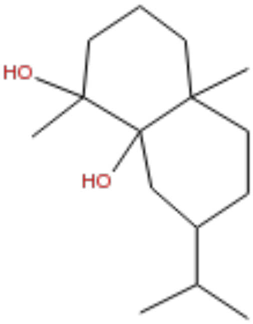

Cell ID	Cluster Center	Number of Compounds
458	0	10

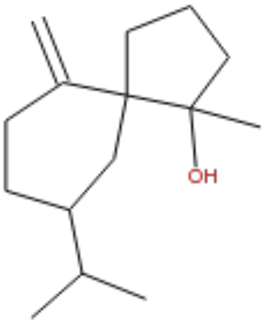

Cell ID	Cluster Center	Number of Compounds
458	0	10

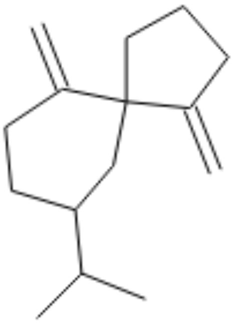

Cell ID	Cluster Center	Number of Compounds
458	0	10

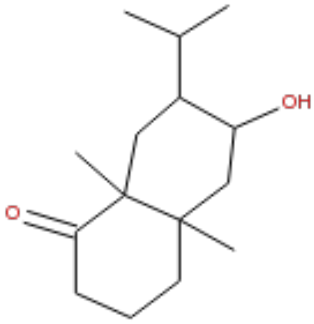

Cell ID	Cluster Center	Number of Compounds
458	0	10

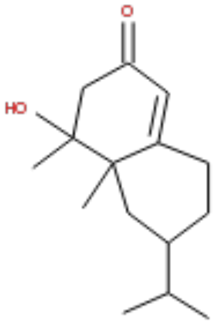

Cell ID	Cluster Center	Number of Compounds
458	0	10

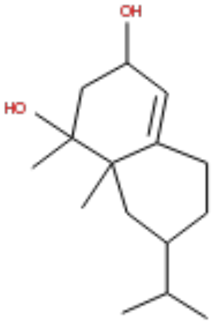

Cell ID	Cluster Center	Number of Compounds
458	0	10

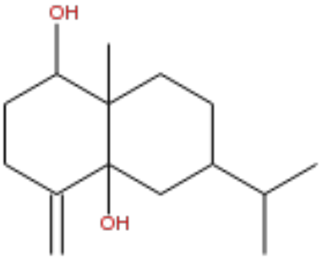

Cell ID	Cluster Center	Number of Compounds
458	0	10

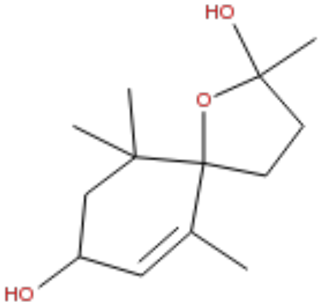

Cell ID	Cluster Center	Number of Compounds
459	1	1

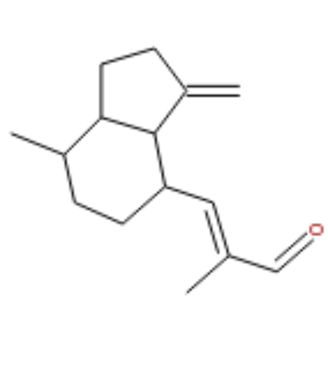

Cell ID	Cluster Center	Number of Compounds
462	1	5

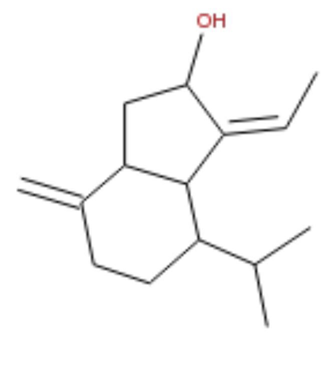

Cell ID	Cluster Center	Number of Compounds
462	0	5

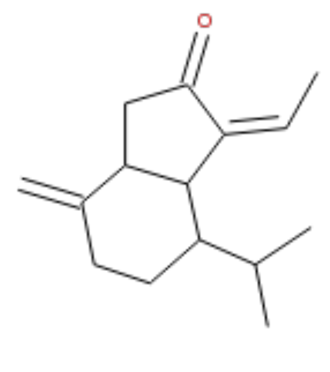

Cell ID	Cluster Center	Number of Compounds
462	0	5

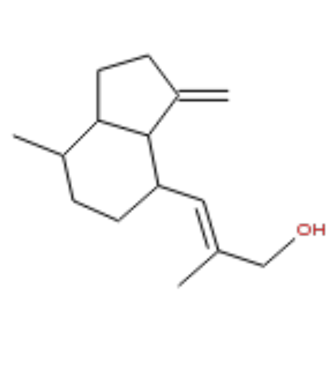

Cell ID	Cluster Center	Number of Compounds
462	0	5

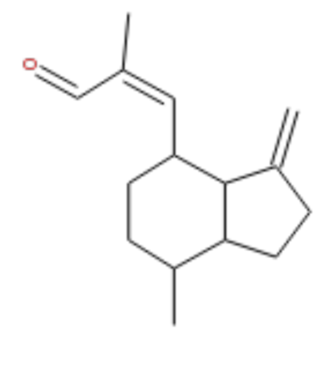

Cell ID	Cluster Center	Number of Compounds
462	0	5

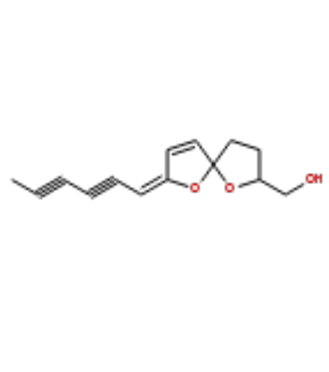

Cell ID	Cluster Center	Number of Compounds
463	1	4

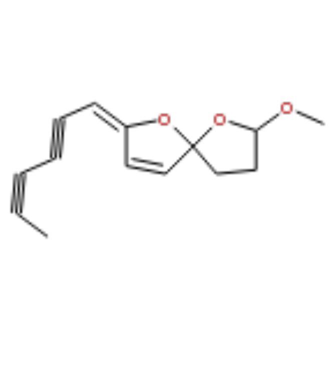

Cell ID	Cluster Center	Number of Compounds
463	0	4

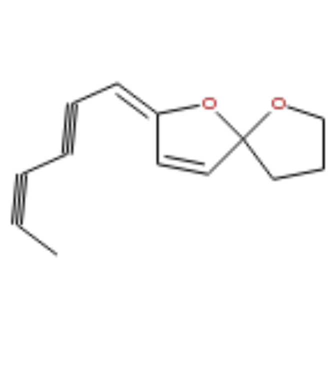

Cell ID	Cluster Center	Number of Compounds
463	0	4

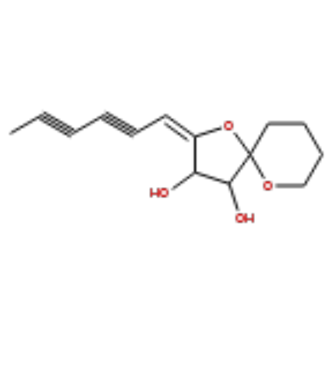

Cell ID	Cluster Center	Number of Compounds
463	0	4

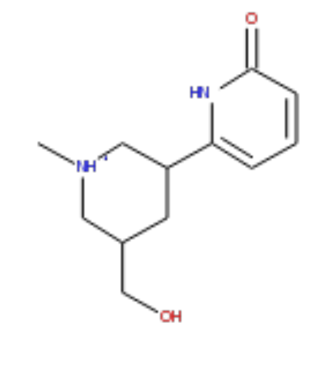

Cell ID	Cluster Center	Number of Compounds
464	1	2

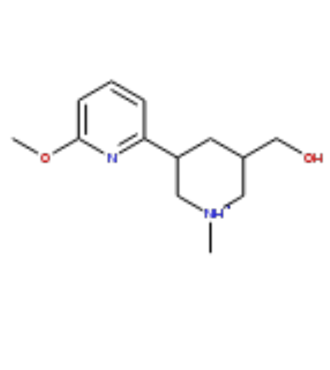

Cell ID	Cluster Center	Number of Compounds
464	0	2

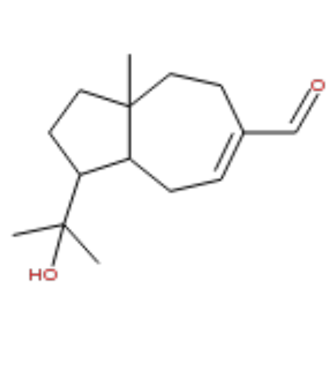

Cell ID	Cluster Center	Number of Compounds
467	1	4

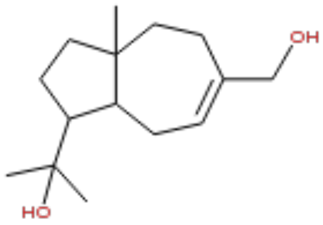

Cell ID	Cluster Center	Number of Compounds
467	0	4

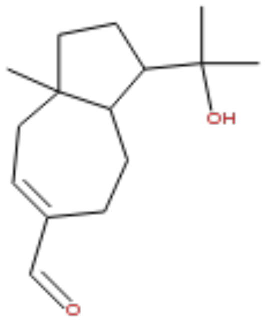

Cell ID	Cluster Center	Number of Compounds
467	0	4

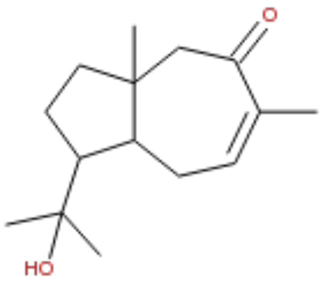

Cell ID	Cluster Center	Number of Compounds
467	0	4

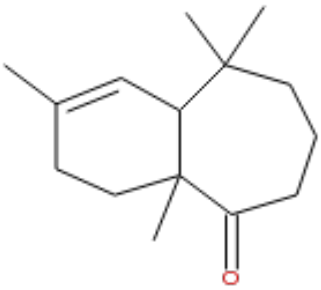

Cell ID	Cluster Center	Number of Compounds
469	1	3

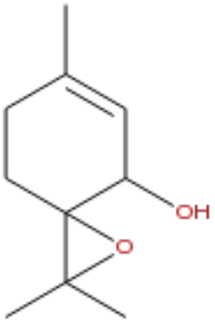

Cell ID	Cluster Center	Number of Compounds
469	0	3

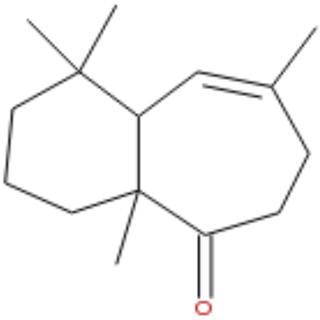

Cell ID	Cluster Center	Number of Compounds
469	0	3

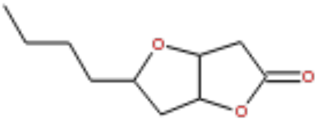

Cell ID	Cluster Center	Number of Compounds
471	1	11

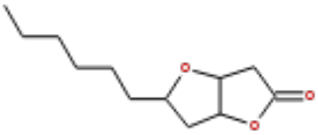

Cell ID	Cluster Center	Number of Compounds
471	0	11

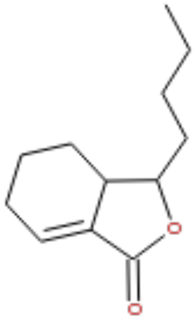

Cell ID	Cluster Center	Number of Compounds
471	0	11

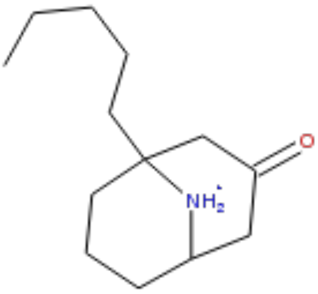

Cell ID	Cluster Center	Number of Compounds
471	0	11

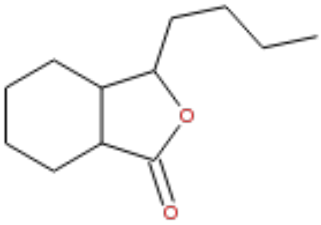

Cell ID	Cluster Center	Number of Compounds
471	0	11

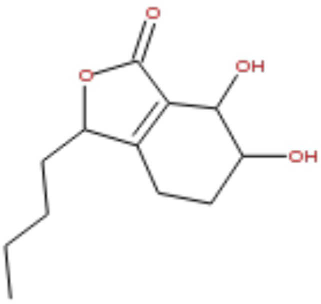

Cell ID	Cluster Center	Number of Compounds
471	0	11

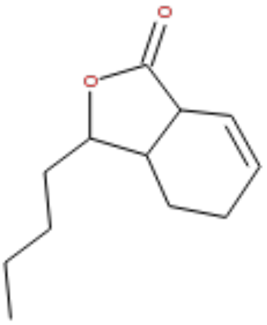

Cell ID	Cluster Center	Number of Compounds
471	0	11

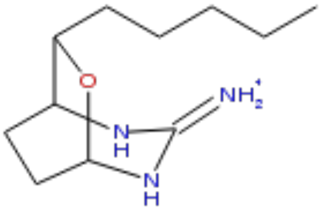

Cell ID	Cluster Center	Number of Compounds
471	0	11

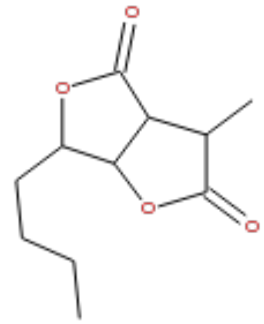

Cell ID	Cluster Center	Number of Compounds
471	0	11

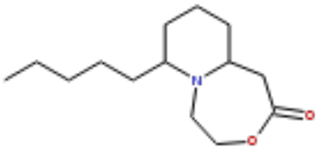

Cell ID	Cluster Center	Number of Compounds
471	0	11

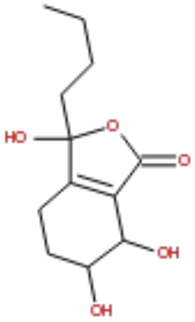

Cell ID	Cluster Center	Number of Compounds
471	0	11

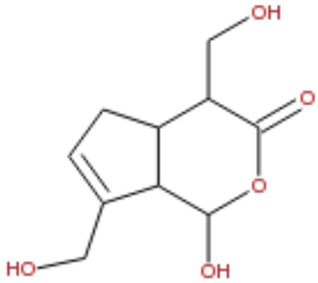

Cell ID	Cluster Center	Number of Compounds
472	1	33

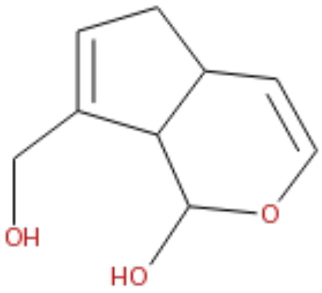

Cell ID	Cluster Center	Number of Compounds
472	0	33

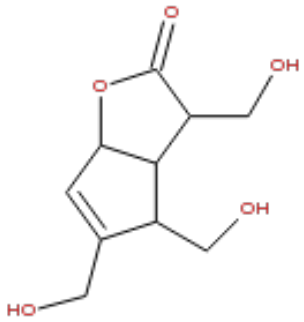

Cell ID	Cluster Center	Number of Compounds
472	0	33

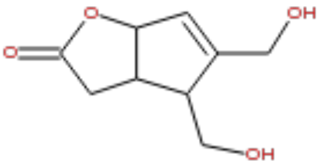

Cell ID	Cluster Center	Number of Compounds
472	0	33

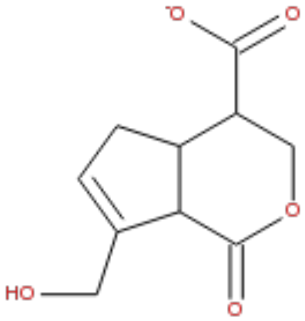

Cell ID	Cluster Center	Number of Compounds
472	0	33

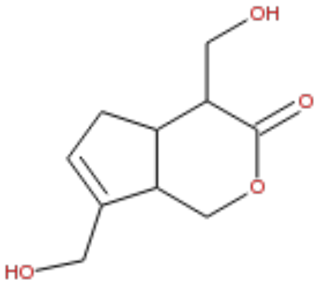

Cell ID	Cluster Center	Number of Compounds
472	0	33

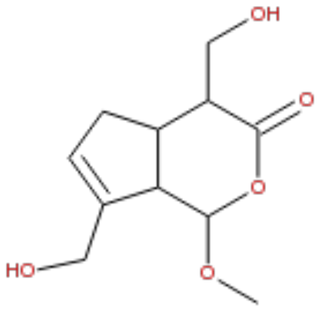

Cell ID	Cluster Center	Number of Compounds
472	0	33

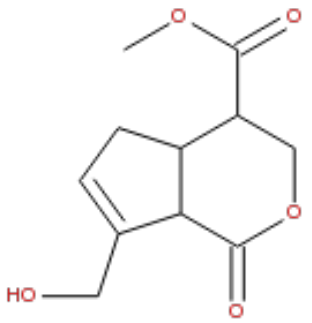

Cell ID	Cluster Center	Number of Compounds
472	0	33

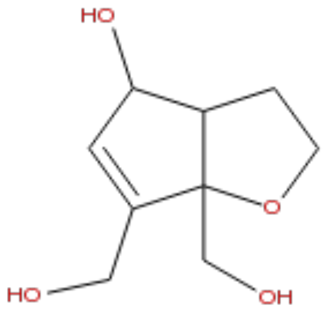

Cell ID	Cluster Center	Number of Compounds
472	0	33

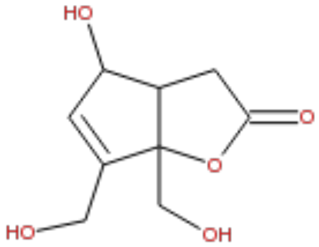

Cell ID	Cluster Center	Number of Compounds
472	0	33

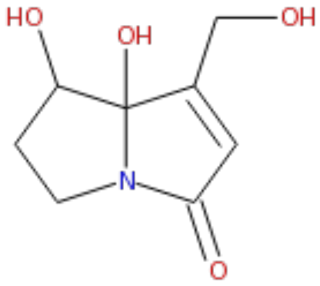

Cell ID	Cluster Center	Number of Compounds
472	0	33

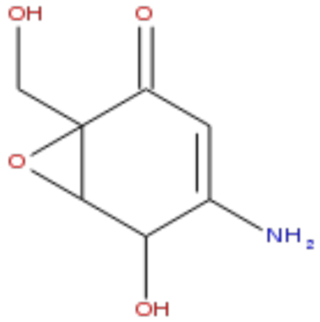

Cell ID	Cluster Center	Number of Compounds
472	0	33

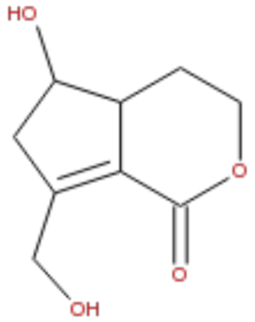

Cell ID	Cluster Center	Number of Compounds
472	0	33

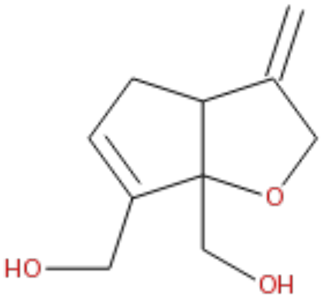

Cell ID	Cluster Center	Number of Compounds
472	0	33

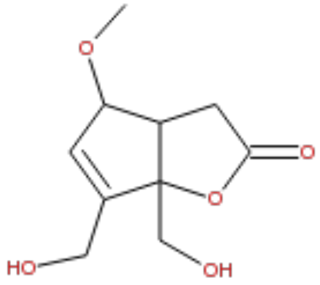

Cell ID	Cluster Center	Number of Compounds
472	0	33

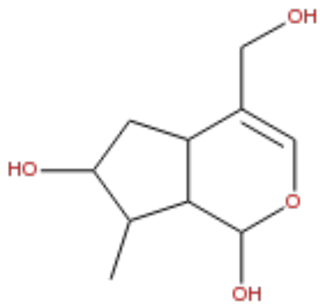

Cell ID	Cluster Center	Number of Compounds
472	0	33

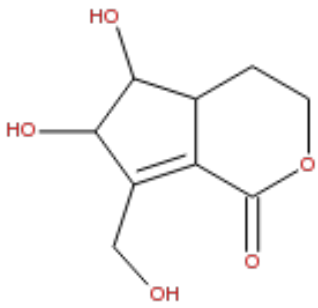

Cell ID	Cluster Center	Number of Compounds
472	0	33

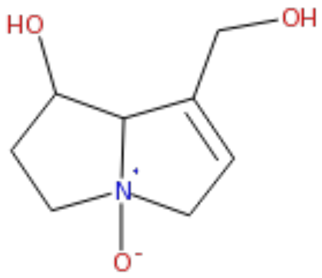

Cell ID	Cluster Center	Number of Compounds
472	0	33

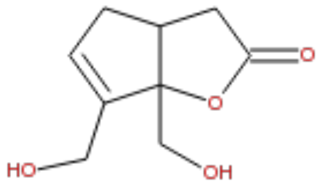

Cell ID	Cluster Center	Number of Compounds
472	0	33

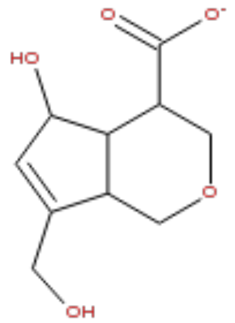

Cell ID	Cluster Center	Number of Compounds
472	0	33

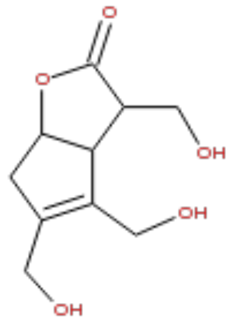

Cell ID	Cluster Center	Number of Compounds
472	0	33

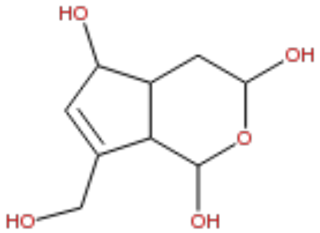

Cell ID	Cluster Center	Number of Compounds
472	0	33

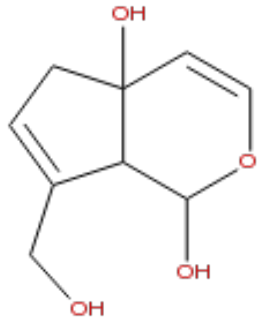

Cell ID	Cluster Center	Number of Compounds
472	0	33

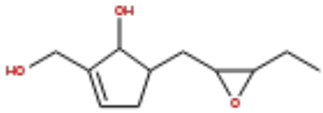

Cell ID	Cluster Center	Number of Compounds
472	0	33

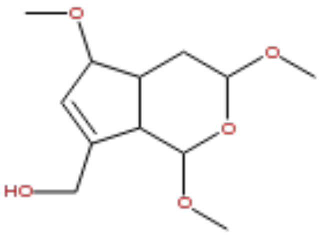

Cell ID	Cluster Center	Number of Compounds
472	0	33

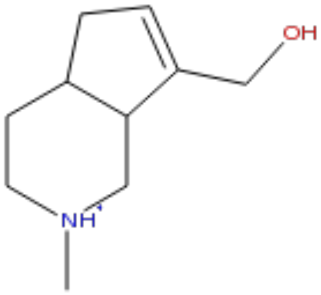

Cell ID	Cluster Center	Number of Compounds
472	0	33

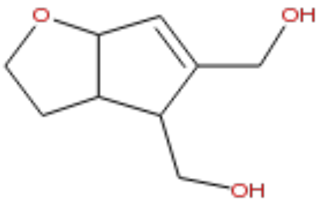

Cell ID	Cluster Center	Number of Compounds
472	0	33

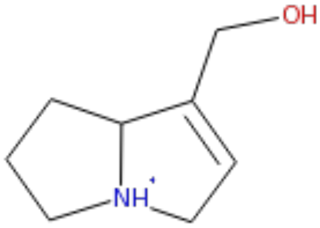

Cell ID	Cluster Center	Number of Compounds
472	0	33

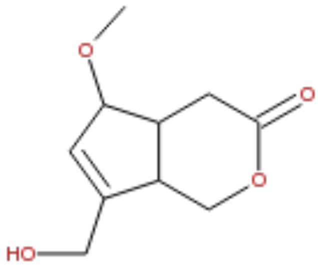

Cell ID	Cluster Center	Number of Compounds
472	0	33

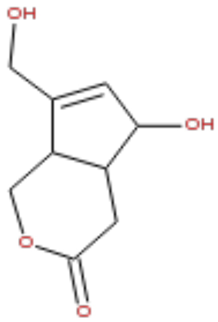

Cell ID	Cluster Center	Number of Compounds
472	0	33

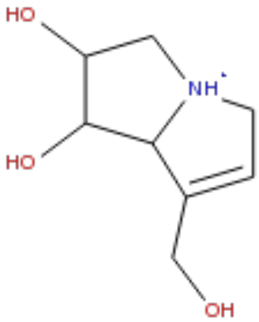

Cell ID	Cluster Center	Number of Compounds
472	0	33

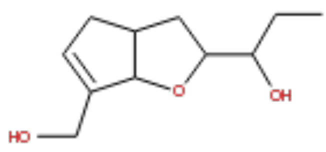

Cell ID	Cluster Center	Number of Compounds
472	0	33

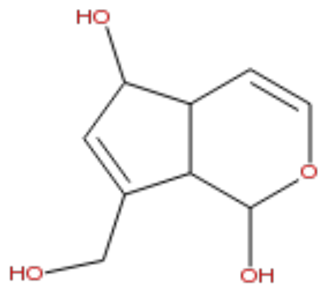

Cell ID	Cluster Center	Number of Compounds
472	0	33

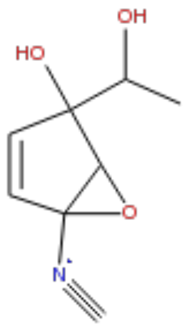

Cell ID	Cluster Center	Number of Compounds
474	1	3

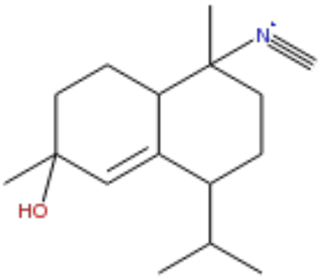

Cell ID	Cluster Center	Number of Compounds
474	0	3

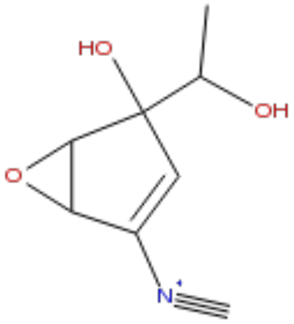

Cell ID	Cluster Center	Number of Compounds
474	0	3

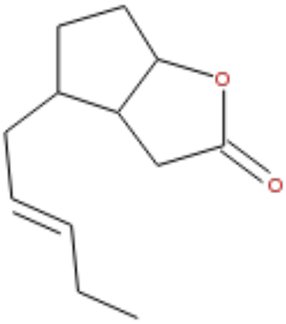

Cell ID	Cluster Center	Number of Compounds
475	1	1

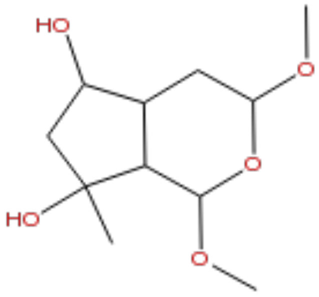

Cell ID	Cluster Center	Number of Compounds
476	1	1

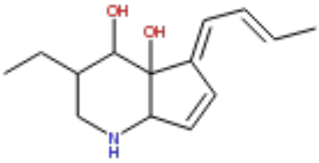

Cell ID	Cluster Center	Number of Compounds
477	1	2

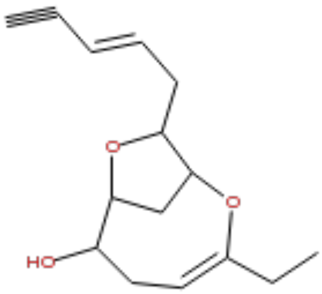

Cell ID	Cluster Center	Number of Compounds
477	0	2

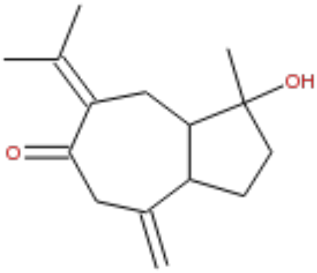

Cell ID	Cluster Center	Number of Compounds
478	1	2

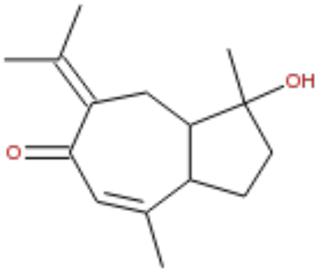

Cell ID	Cluster Center	Number of Compounds
478	0	2

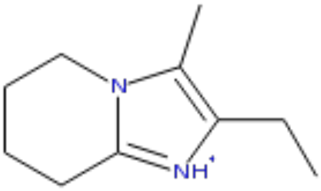

Cell ID	Cluster Center	Number of Compounds
479	1	4

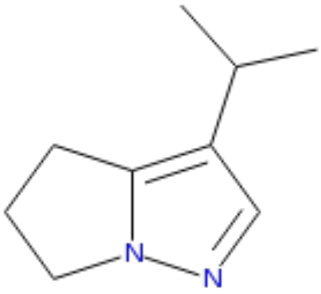

Cell ID	Cluster Center	Number of Compounds
479	0	4

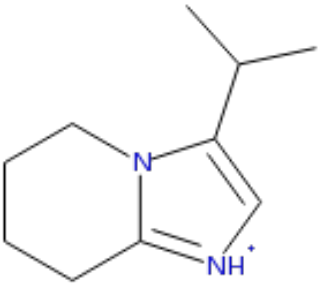

Cell ID	Cluster Center	Number of Compounds
479	0	4

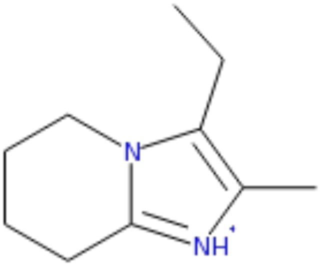

Cell ID	Cluster Center	Number of Compounds
479	0	4

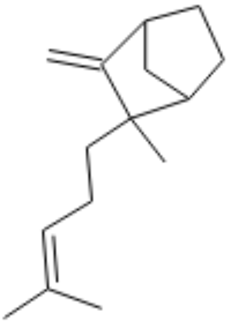

Cell ID	Cluster Center	Number of Compounds
480	1	7

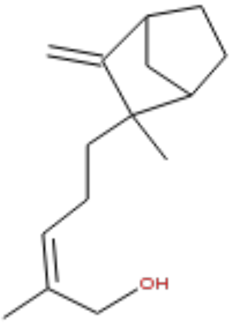

Cell ID	Cluster Center	Number of Compounds
480	0	7

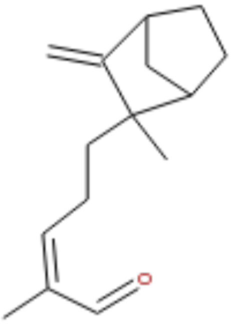

Cell ID	Cluster Center	Number of Compounds
480	0	7

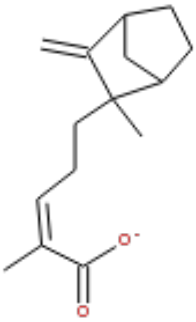

Cell ID	Cluster Center	Number of Compounds
480	0	7

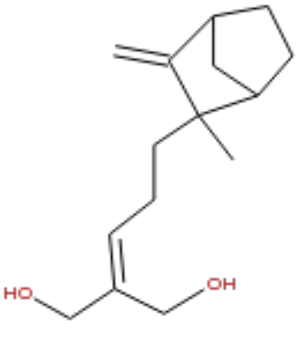

Cell ID	Cluster Center	Number of Compounds
480	0	7

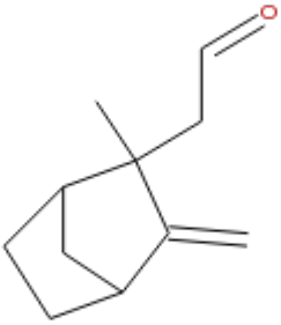

Cell ID	Cluster Center	Number of Compounds
480	0	7

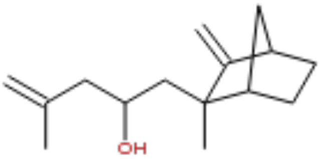

Cell ID	Cluster Center	Number of Compounds
480	0	7

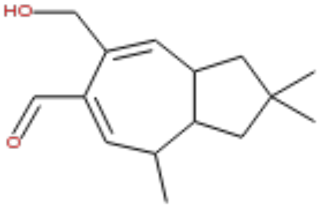

Cell ID	Cluster Center	Number of Compounds
481	1	5

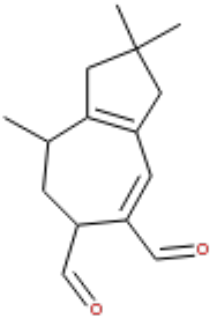

Cell ID	Cluster Center	Number of Compounds
481	0	5

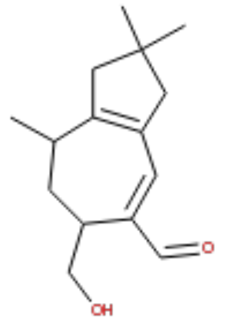

Cell ID	Cluster Center	Number of Compounds
481	0	5

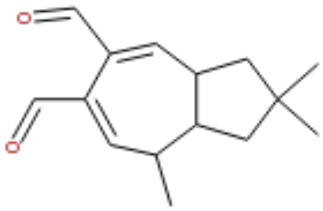

Cell ID	Cluster Center	Number of Compounds
481	0	5

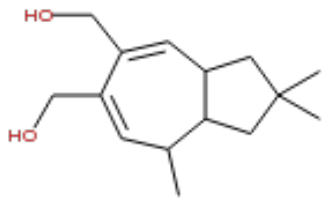

Cell ID	Cluster Center	Number of Compounds
481	0	5

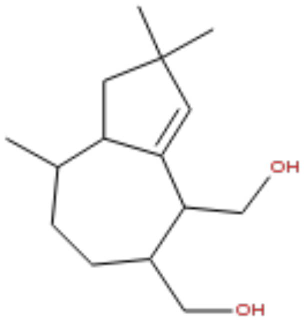

Cell ID	Cluster Center	Number of Compounds
482	1	2

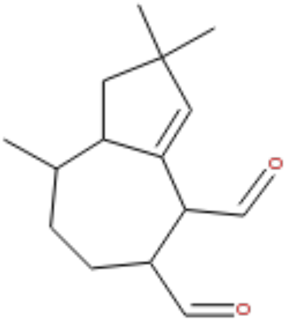

Cell ID	Cluster Center	Number of Compounds
482	0	2

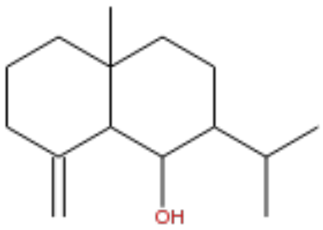

Cell ID	Cluster Center	Number of Compounds
483	1	18

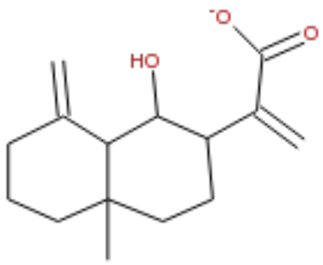

Cell ID	Cluster Center	Number of Compounds
483	0	18

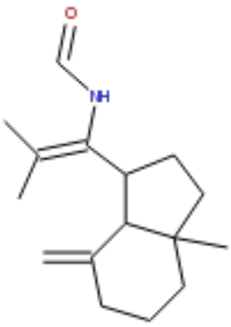

Cell ID	Cluster Center	Number of Compounds
483	0	18

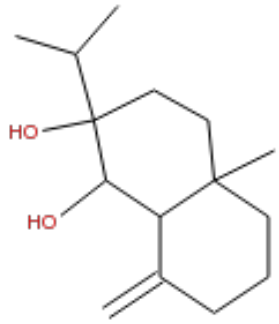

Cell ID	Cluster Center	Number of Compounds
483	0	18

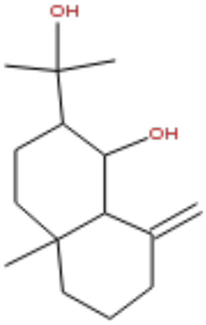

Cell ID	Cluster Center	Number of Compounds
483	0	18

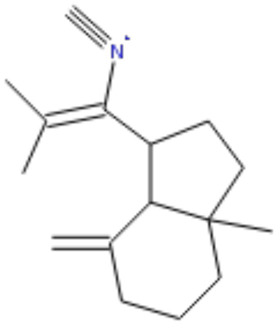

Cell ID	Cluster Center	Number of Compounds
483	0	18

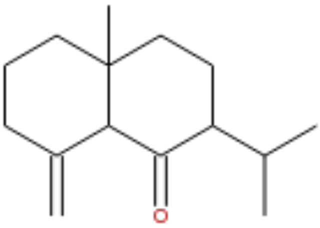

Cell ID	Cluster Center	Number of Compounds
483	0	18

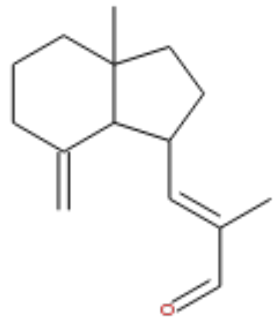

Cell ID	Cluster Center	Number of Compounds
483	0	18

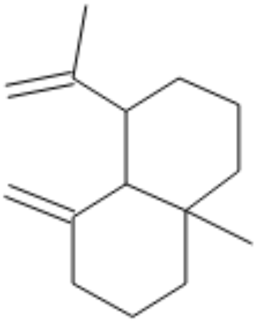

Cell ID	Cluster Center	Number of Compounds
483	0	18

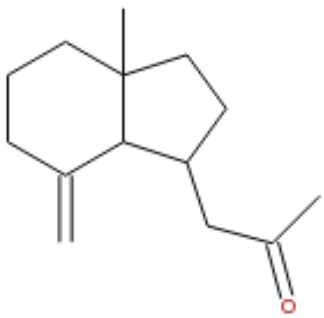

Cell ID	Cluster Center	Number of Compounds
483	0	18

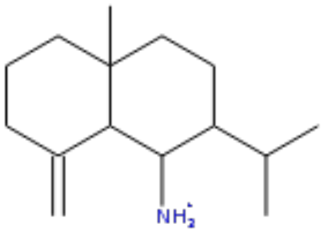

Cell ID	Cluster Center	Number of Compounds
483	0	18

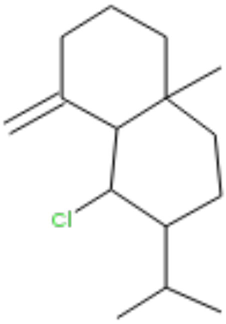

Cell ID	Cluster Center	Number of Compounds
483	0	18

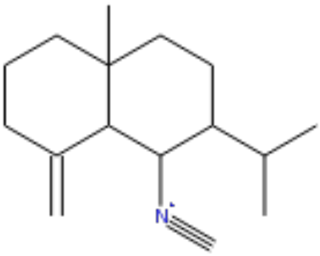

Cell ID	Cluster Center	Number of Compounds
483	0	18

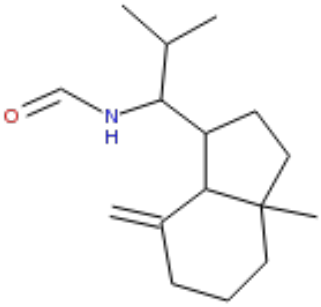

Cell ID	Cluster Center	Number of Compounds
483	0	18

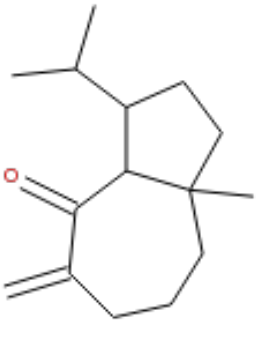

Cell ID	Cluster Center	Number of Compounds
483	0	18

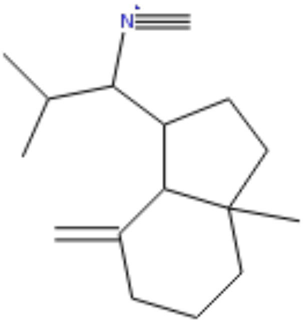

Cell ID	Cluster Center	Number of Compounds
483	0	18

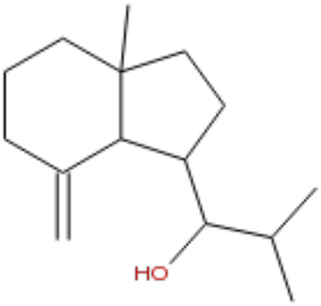

Cell ID	Cluster Center	Number of Compounds
483	0	18

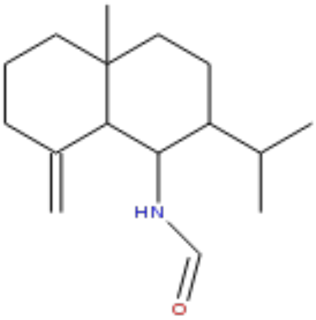

Cell ID	Cluster Center	Number of Compounds
483	0	18

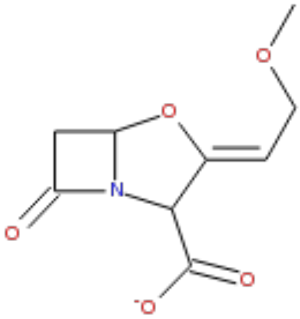

Cell ID	Cluster Center	Number of Compounds
486	1	5

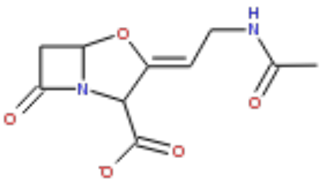

Cell ID	Cluster Center	Number of Compounds
486	0	5

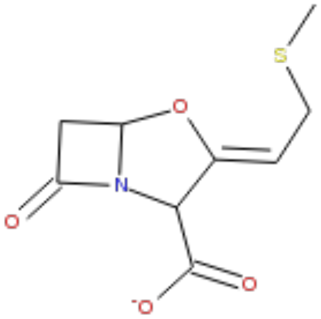

Cell ID	Cluster Center	Number of Compounds
486	0	5

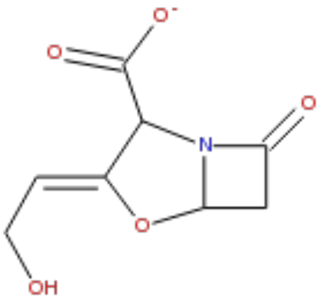

Cell ID	Cluster Center	Number of Compounds
486	0	5

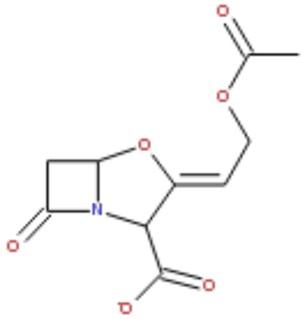

Cell ID	Cluster Center	Number of Compounds
486	0	5

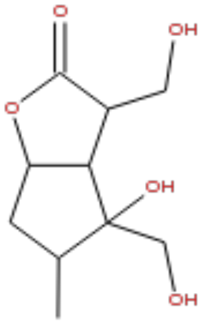

Cell ID	Cluster Center	Number of Compounds
487	1	1

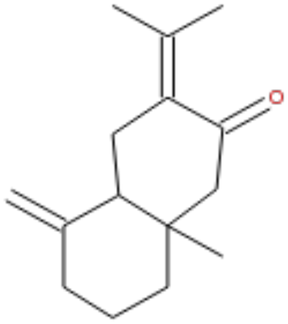

Cell ID	Cluster Center	Number of Compounds
489	1	19

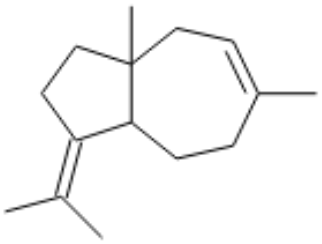

Cell ID	Cluster Center	Number of Compounds
489	0	19

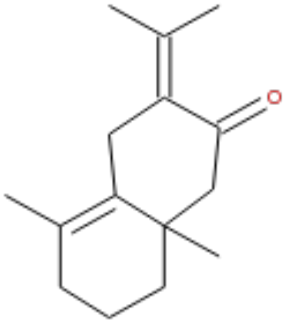

Cell ID	Cluster Center	Number of Compounds
489	0	19

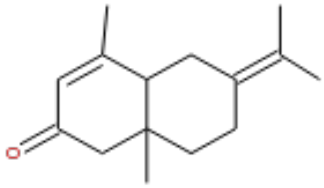

Cell ID	Cluster Center	Number of Compounds
489	0	19

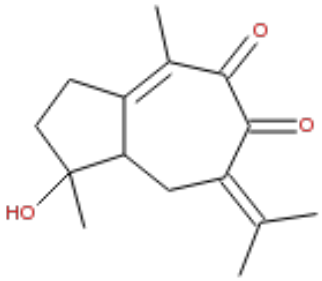

Cell ID	Cluster Center	Number of Compounds
489	0	19

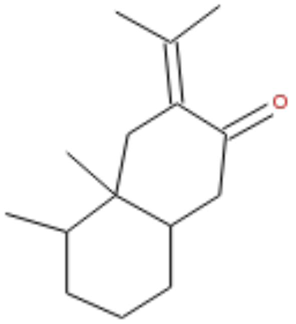

Cell ID	Cluster Center	Number of Compounds
489	0	19

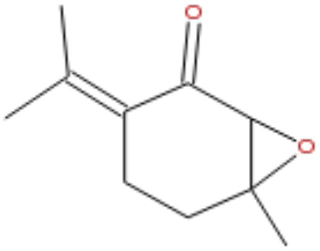

Cell ID	Cluster Center	Number of Compounds
489	0	19

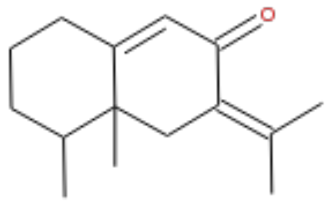

Cell ID	Cluster Center	Number of Compounds
489	0	19

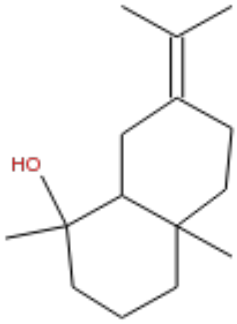

Cell ID	Cluster Center	Number of Compounds
489	0	19

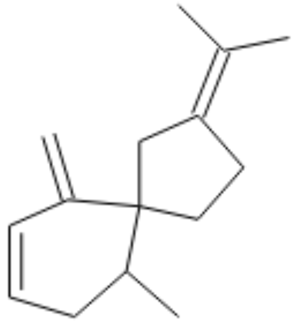

Cell ID	Cluster Center	Number of Compounds
489	0	19

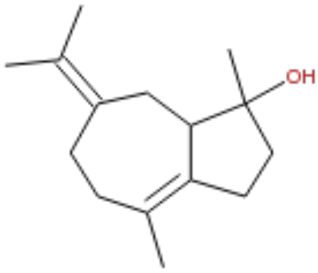

Cell ID	Cluster Center	Number of Compounds
489	0	19

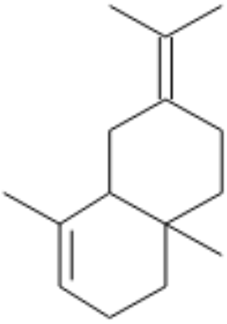

Cell ID	Cluster Center	Number of Compounds
489	0	19

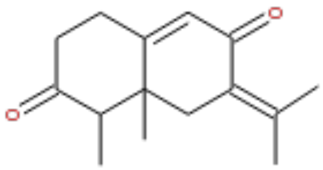

Cell ID	Cluster Center	Number of Compounds
489	0	19

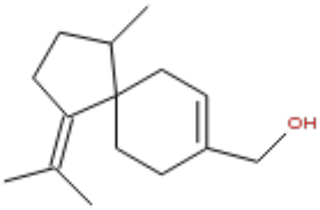

Cell ID	Cluster Center	Number of Compounds
489	0	19

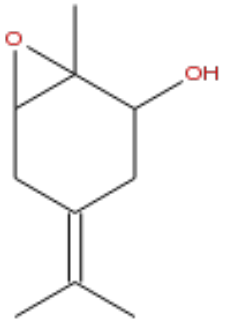

Cell ID	Cluster Center	Number of Compounds
489	0	19

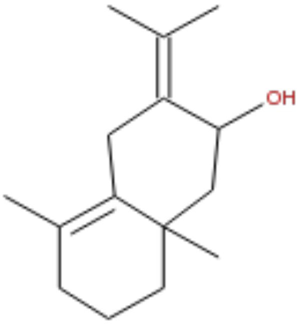

Cell ID	Cluster Center	Number of Compounds
489	0	19

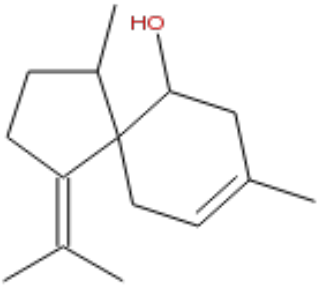

Cell ID	Cluster Center	Number of Compounds
489	0	19

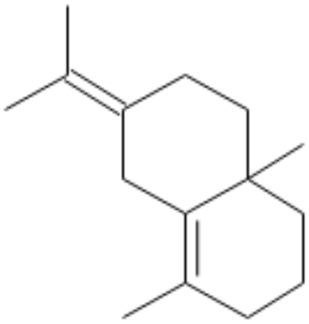

Cell ID	Cluster Center	Number of Compounds
489	0	19

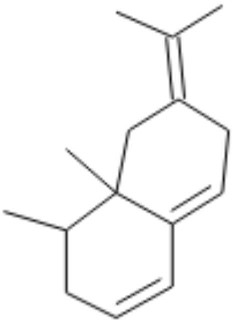

Cell ID	Cluster Center	Number of Compounds
489	0	19

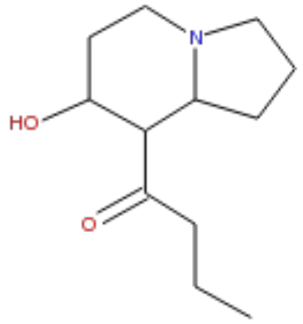

Cell ID	Cluster Center	Number of Compounds
490	1	1

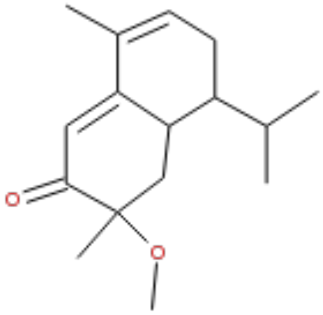

Cell ID	Cluster Center	Number of Compounds
491	1	3

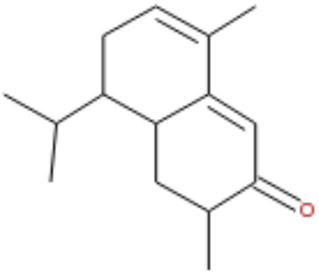

Cell ID	Cluster Center	Number of Compounds
491	0	3

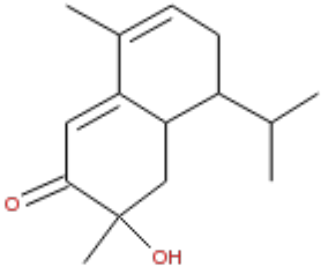

Cell ID	Cluster Center	Number of Compounds
491	0	3

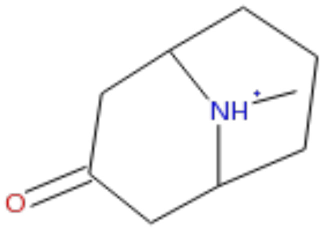

Cell ID	Cluster Center	Number of Compounds
492	1	20

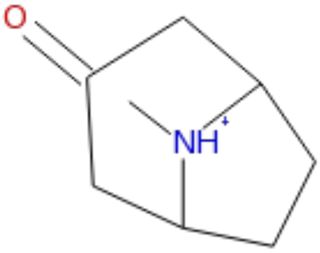

Cell ID	Cluster Center	Number of Compounds
492	0	20

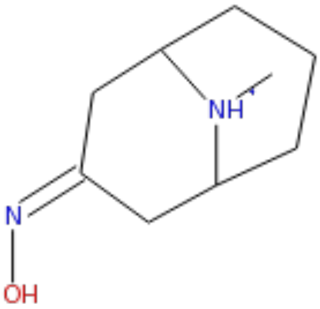

Cell ID	Cluster Center	Number of Compounds
492	0	20

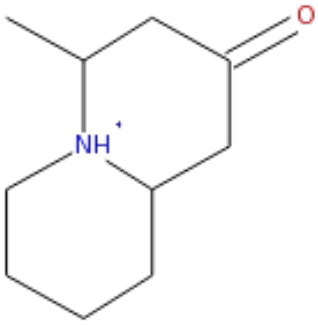

Cell ID	Cluster Center	Number of Compounds
492	0	20

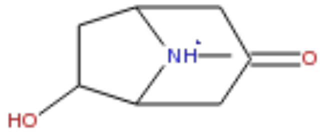

Cell ID	Cluster Center	Number of Compounds
492	0	20

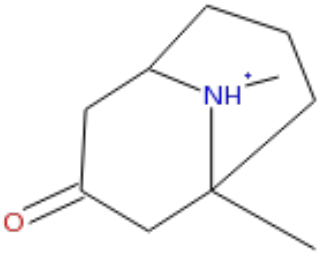

Cell ID	Cluster Center	Number of Compounds
492	0	20

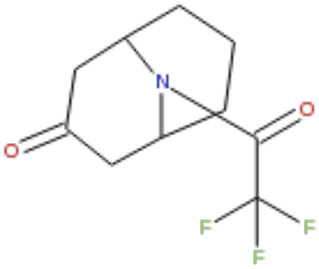

Cell ID	Cluster Center	Number of Compounds
492	0	20

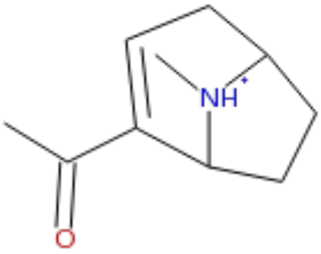

Cell ID	Cluster Center	Number of Compounds
492	0	20

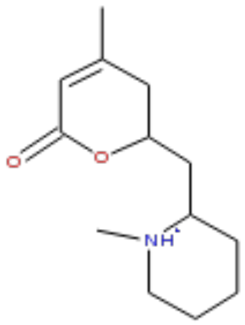

Cell ID	Cluster Center	Number of Compounds
492	0	20

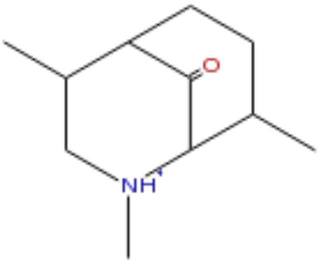

Cell ID	Cluster Center	Number of Compounds
492	0	20

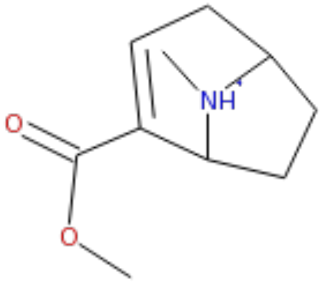

Cell ID	Cluster Center	Number of Compounds
492	0	20

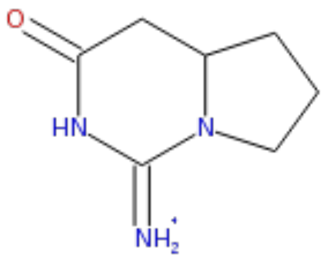

Cell ID	Cluster Center	Number of Compounds
492	0	20

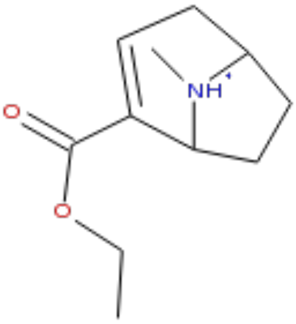

Cell ID	Cluster Center	Number of Compounds
492	0	20

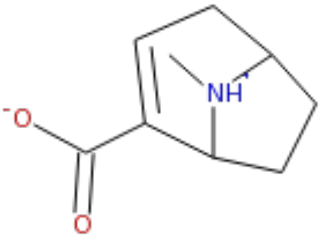

Cell ID	Cluster Center	Number of Compounds
492	0	20

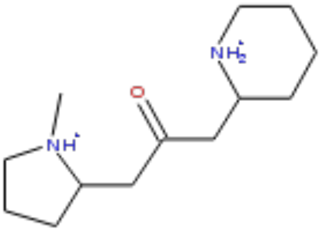

Cell ID	Cluster Center	Number of Compounds
492	0	20

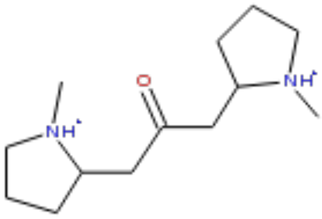

Cell ID	Cluster Center	Number of Compounds
492	0	20

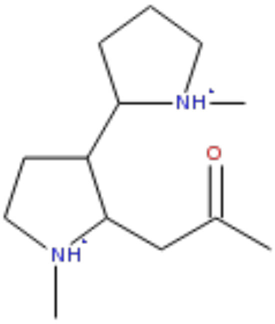

Cell ID	Cluster Center	Number of Compounds
492	0	20

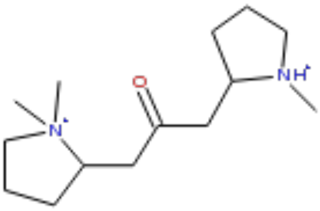

Cell ID	Cluster Center	Number of Compounds
492	0	20

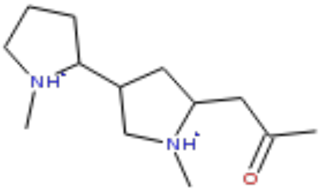

Cell ID	Cluster Center	Number of Compounds
492	0	20

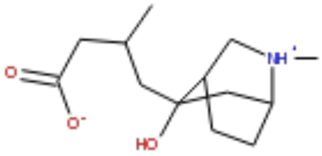

Cell ID	Cluster Center	Number of Compounds
492	0	20

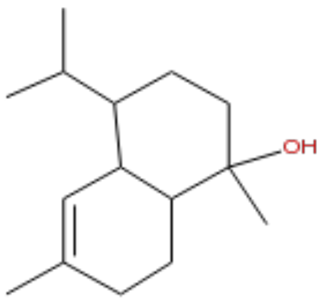

Cell ID	Cluster Center	Number of Compounds
493	1	12

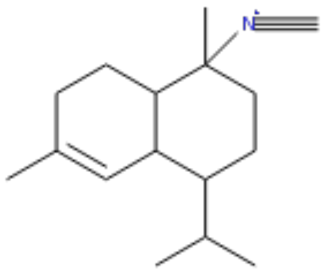

Cell ID	Cluster Center	Number of Compounds
493	0	12

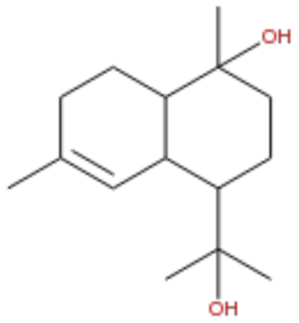

Cell ID	Cluster Center	Number of Compounds
493	0	12

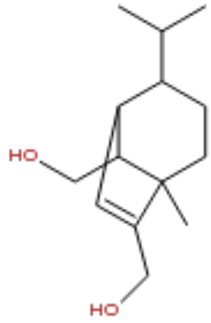

Cell ID	Cluster Center	Number of Compounds
493	0	12

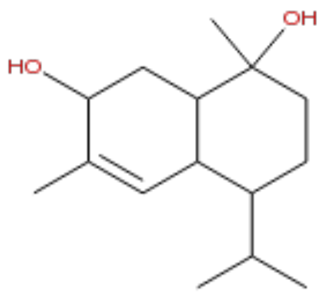

Cell ID	Cluster Center	Number of Compounds
493	0	12

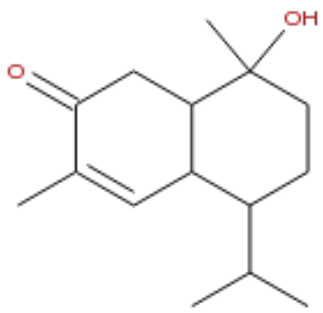

Cell ID	Cluster Center	Number of Compounds
493	0	12

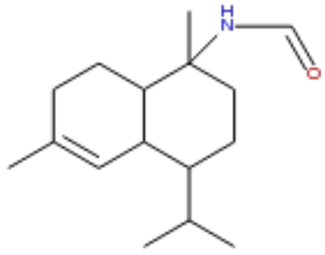

Cell ID	Cluster Center	Number of Compounds
493	0	12

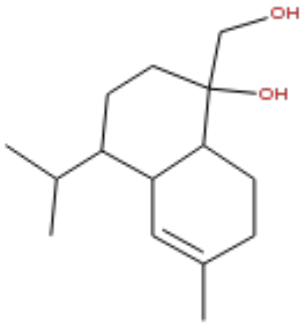

Cell ID	Cluster Center	Number of Compounds
493	0	12

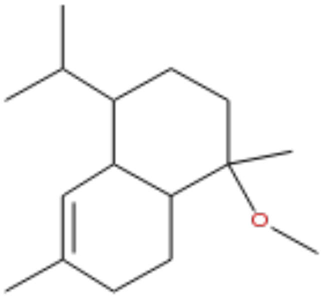

Cell ID	Cluster Center	Number of Compounds
493	0	12

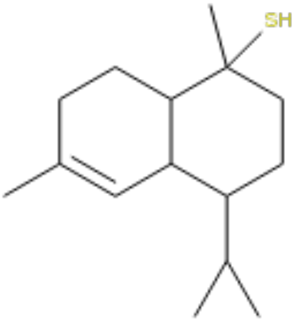

Cell ID	Cluster Center	Number of Compounds
493	0	12

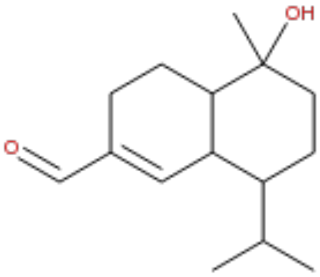

Cell ID	Cluster Center	Number of Compounds
493	0	12

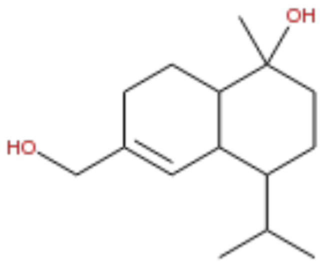

Cell ID	Cluster Center	Number of Compounds
493	0	12

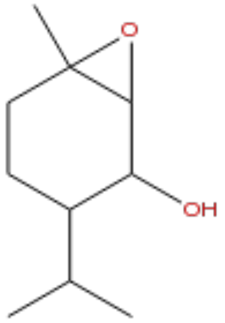

Cell ID	Cluster Center	Number of Compounds
495	1	12

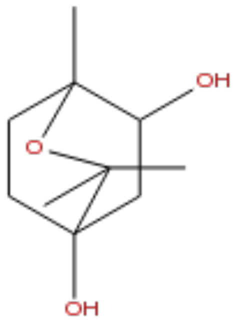

Cell ID	Cluster Center	Number of Compounds
495	0	12

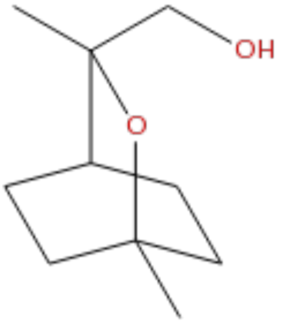

Cell ID	Cluster Center	Number of Compounds
495	0	12

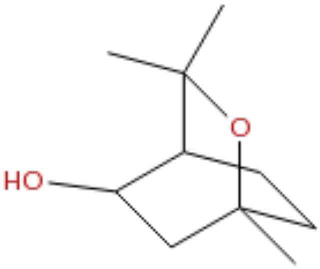

Cell ID	Cluster Center	Number of Compounds
495	0	12

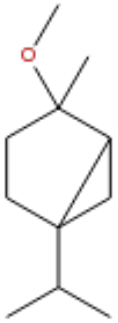

Cell ID	Cluster Center	Number of Compounds
495	0	12

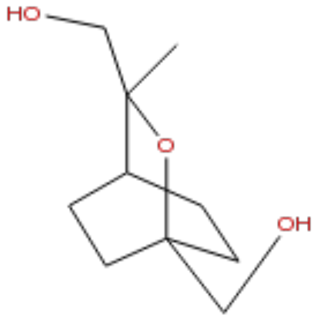

Cell ID	Cluster Center	Number of Compounds
495	0	12

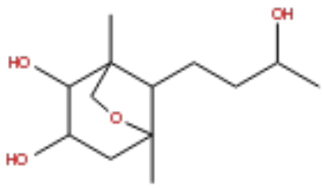

Cell ID	Cluster Center	Number of Compounds
495	0	12

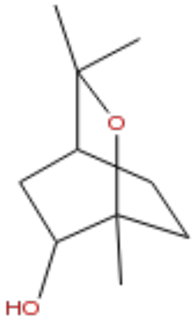

Cell ID	Cluster Center	Number of Compounds
495	0	12

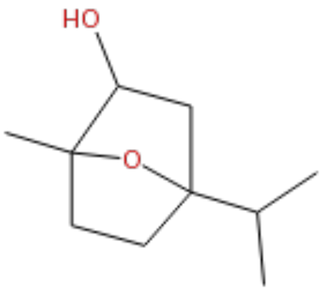

Cell ID	Cluster Center	Number of Compounds
495	0	12

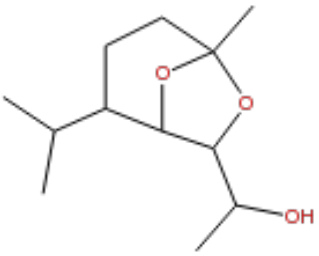

Cell ID	Cluster Center	Number of Compounds
495	0	12

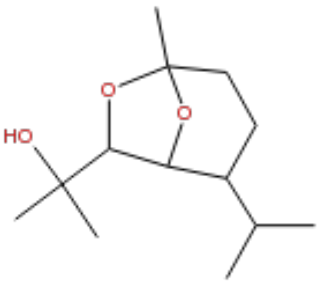

Cell ID	Cluster Center	Number of Compounds
495	0	12

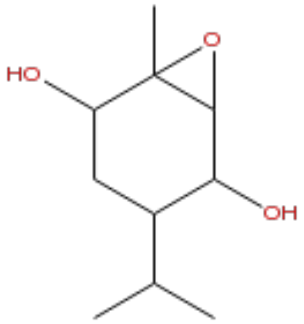

Cell ID	Cluster Center	Number of Compounds
495	0	12

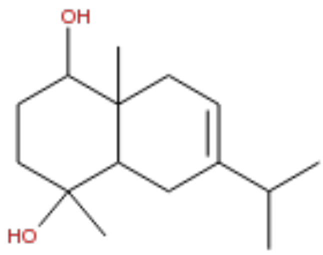

Cell ID	Cluster Center	Number of Compounds
497	1	9

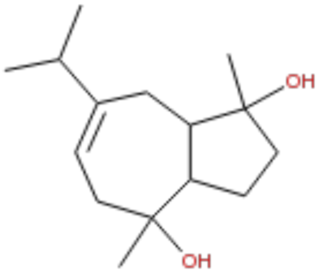

Cell ID	Cluster Center	Number of Compounds
497	0	9

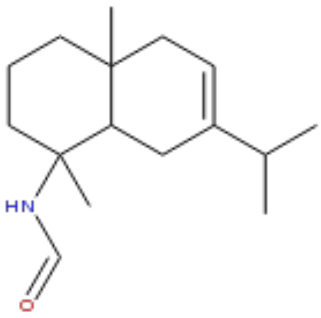

Cell ID	Cluster Center	Number of Compounds
497	0	9

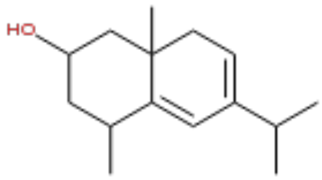

Cell ID	Cluster Center	Number of Compounds
497	0	9

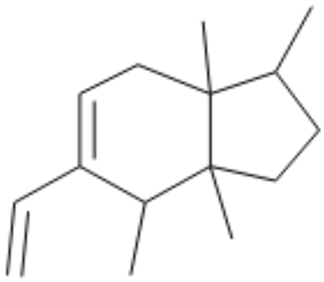

Cell ID	Cluster Center	Number of Compounds
497	0	9

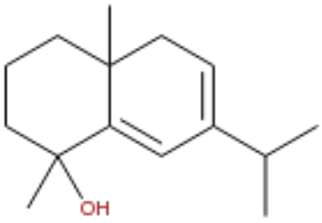

Cell ID	Cluster Center	Number of Compounds
497	0	9

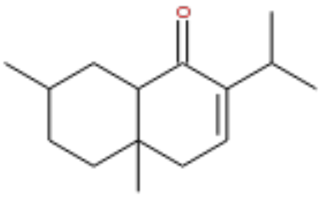

Cell ID	Cluster Center	Number of Compounds
497	0	9

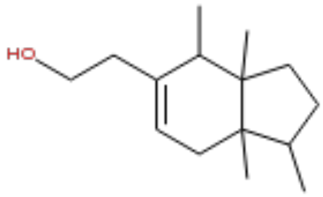

Cell ID	Cluster Center	Number of Compounds
497	0	9

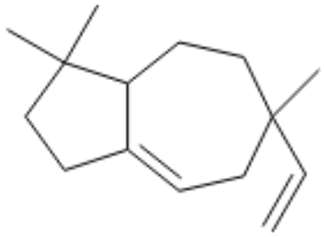

Cell ID	Cluster Center	Number of Compounds
497	0	9

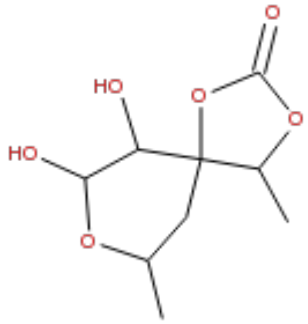

Cell ID	Cluster Center	Number of Compounds
498	1	9

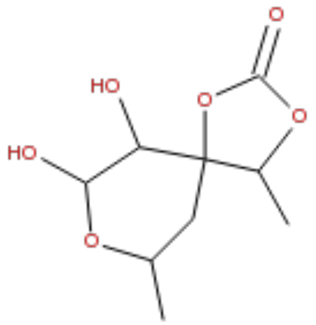

Cell ID	Cluster Center	Number of Compounds
498	0	9

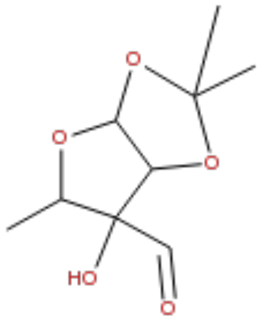

Cell ID	Cluster Center	Number of Compounds
498	0	9

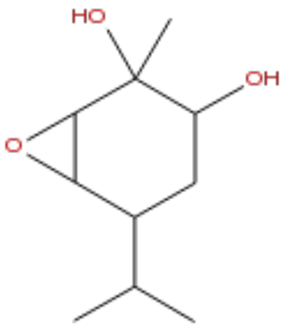

Cell ID	Cluster Center	Number of Compounds
498	0	9

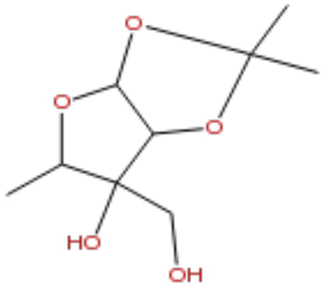

Cell ID	Cluster Center	Number of Compounds
498	0	9

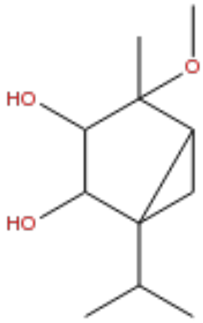

Cell ID	Cluster Center	Number of Compounds
498	0	9

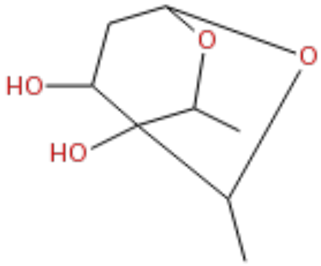

Cell ID	Cluster Center	Number of Compounds
498	0	9

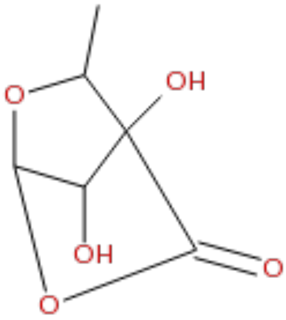

Cell ID	Cluster Center	Number of Compounds
498	0	9

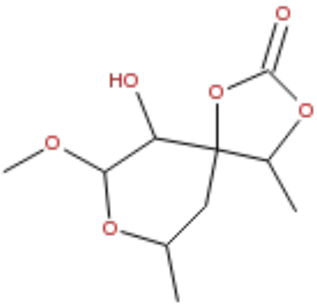

Cell ID	Cluster Center	Number of Compounds
498	0	9

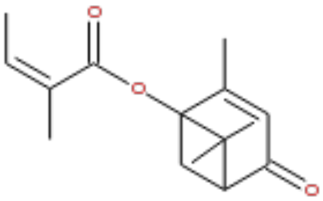

Cell ID	Cluster Center	Number of Compounds
499	1	2

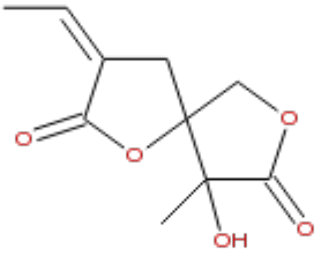

Cell ID	Cluster Center	Number of Compounds
499	0	2

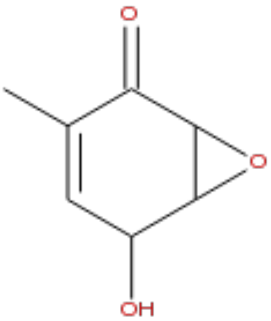

Cell ID	Cluster Center	Number of Compounds
500	1	37

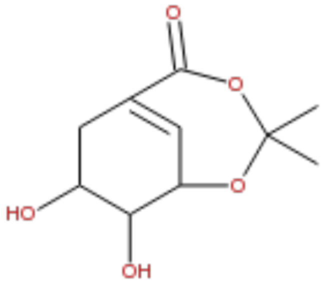

Cell ID	Cluster Center	Number of Compounds
500	0	37

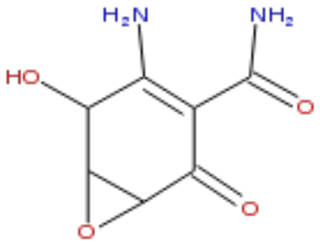

Cell ID	Cluster Center	Number of Compounds
500	0	37

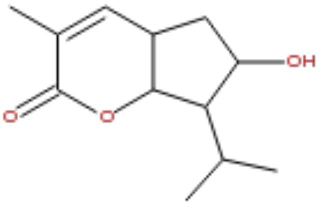

Cell ID	Cluster Center	Number of Compounds
500	0	37

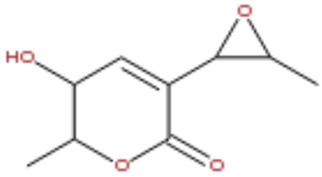

Cell ID	Cluster Center	Number of Compounds
500	0	37

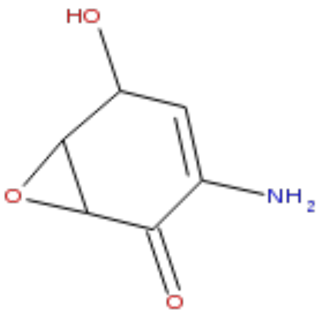

Cell ID	Cluster Center	Number of Compounds
500	0	37

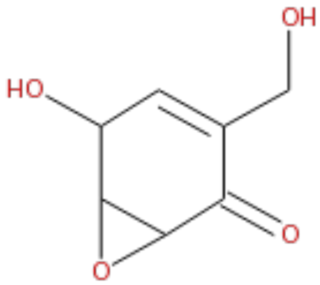

Cell ID	Cluster Center	Number of Compounds
500	0	37

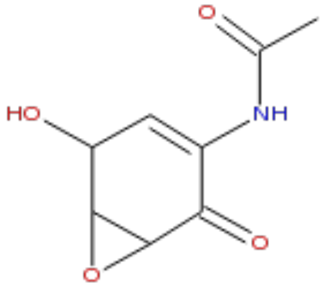

Cell ID	Cluster Center	Number of Compounds
500	0	37

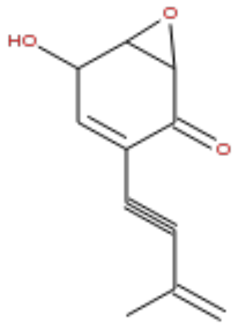

Cell ID	Cluster Center	Number of Compounds
500	0	37

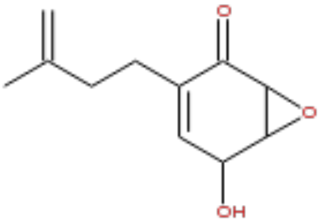

Cell ID	Cluster Center	Number of Compounds
500	0	37

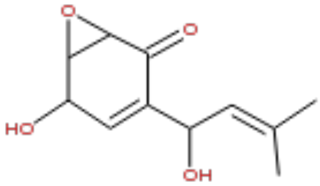

Cell ID	Cluster Center	Number of Compounds
500	0	37

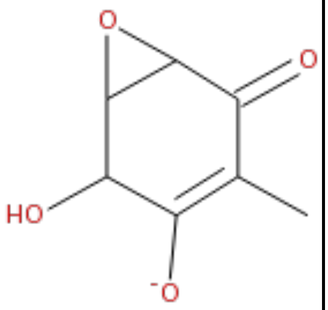

Cell ID	Cluster Center	Number of Compounds
500	0	37

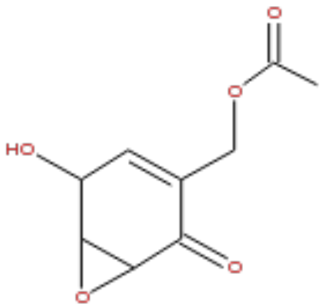

Cell ID	Cluster Center	Number of Compounds
500	0	37

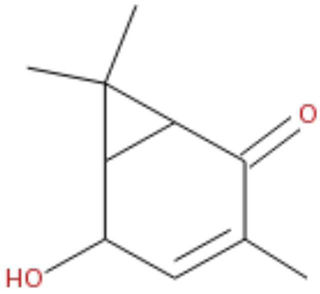

Cell ID	Cluster Center	Number of Compounds
500	0	37

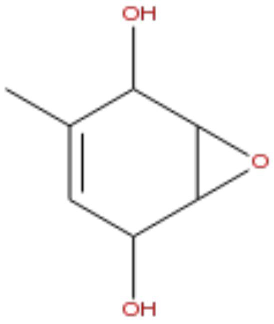

Cell ID	Cluster Center	Number of Compounds
500	0	37

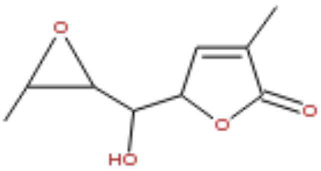

Cell ID	Cluster Center	Number of Compounds
500	0	37

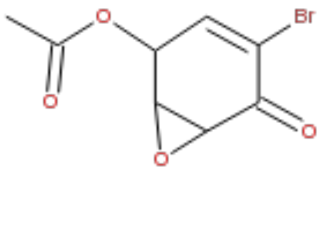

Cell ID	Cluster Center	Number of Compounds
500	0	37

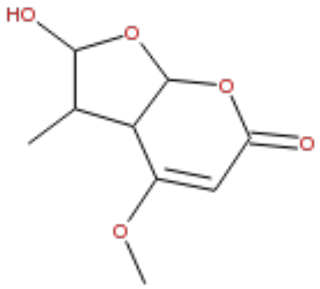

Cell ID	Cluster Center	Number of Compounds
500	0	37

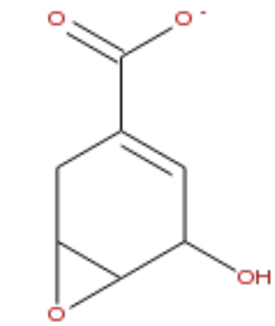

Cell ID	Cluster Center	Number of Compounds
500	0	37

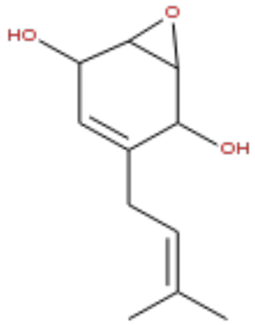

Cell ID	Cluster Center	Number of Compounds
500	0	37

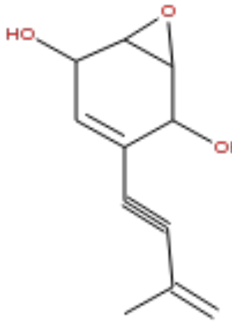

Cell ID	Cluster Center	Number of Compounds
500	0	37

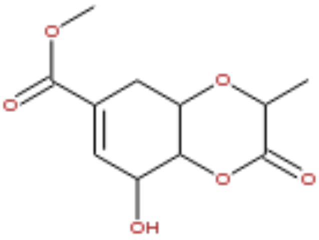

Cell ID	Cluster Center	Number of Compounds
500	0	37

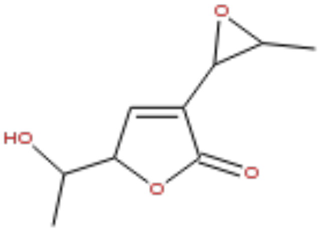

Cell ID	Cluster Center	Number of Compounds
500	0	37

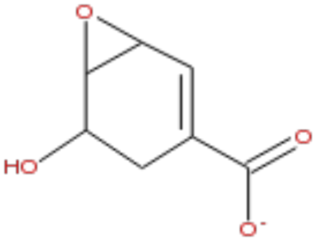

Cell ID	Cluster Center	Number of Compounds
500	0	37

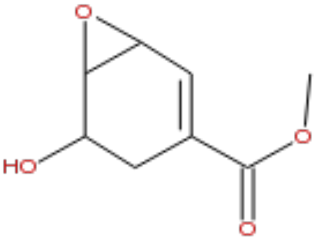

Cell ID	Cluster Center	Number of Compounds
500	0	37

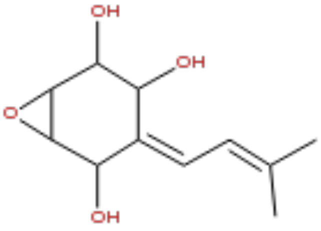

Cell ID	Cluster Center	Number of Compounds
500	0	37

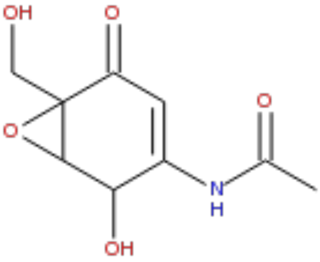

Cell ID	Cluster Center	Number of Compounds
500	0	37

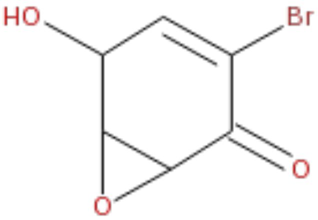

Cell ID	Cluster Center	Number of Compounds
500	0	37

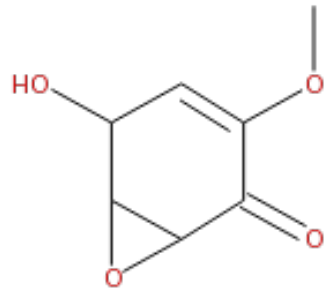

Cell ID	Cluster Center	Number of Compounds
500	0	37

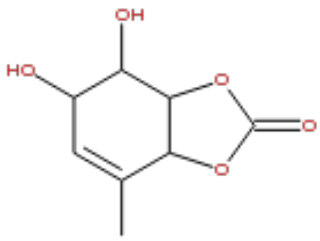

Cell ID	Cluster Center	Number of Compounds
500	0	37

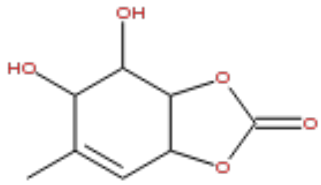

Cell ID	Cluster Center	Number of Compounds
500	0	37

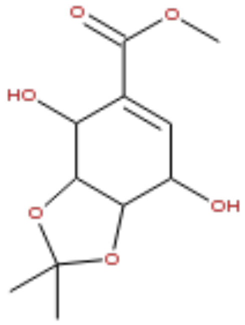

Cell ID	Cluster Center	Number of Compounds
500	0	37

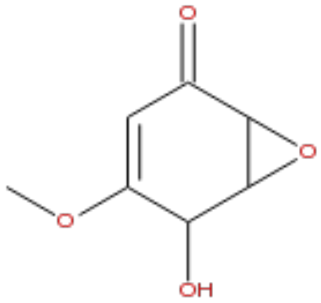

Cell ID	Cluster Center	Number of Compounds
500	0	37

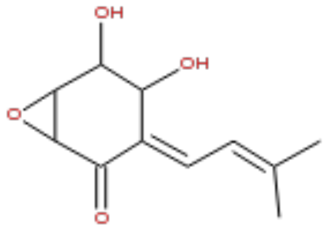

Cell ID	Cluster Center	Number of Compounds
500	0	37

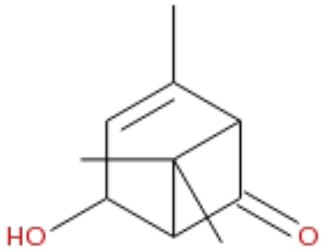

Cell ID	Cluster Center	Number of Compounds
500	0	37

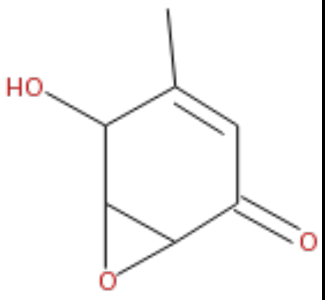

Cell ID	Cluster Center	Number of Compounds
500	0	37

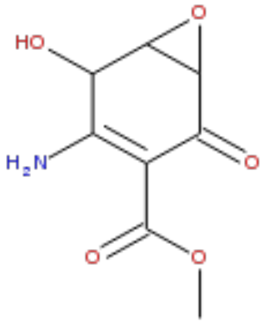

Cell ID	Cluster Center	Number of Compounds
500	0	37

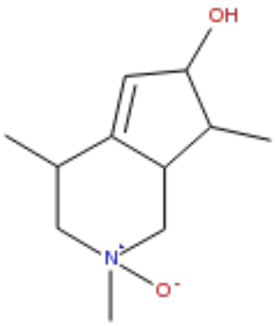

Cell ID	Cluster Center	Number of Compounds
502	1	2

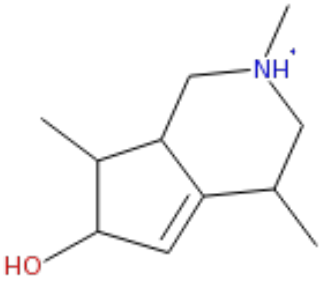

Cell ID	Cluster Center	Number of Compounds
502	0	2

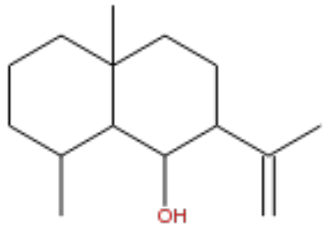

Cell ID	Cluster Center	Number of Compounds
504	1	6

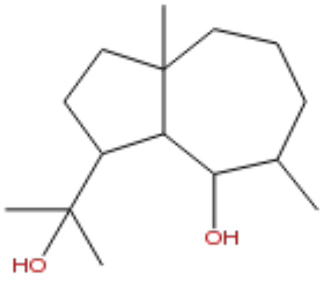

Cell ID	Cluster Center	Number of Compounds
504	0	6

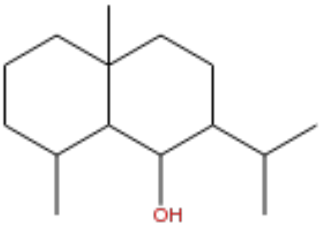

Cell ID	Cluster Center	Number of Compounds
504	0	6

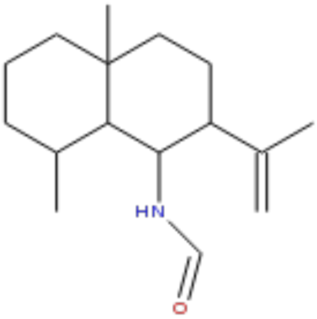

Cell ID	Cluster Center	Number of Compounds
504	0	6

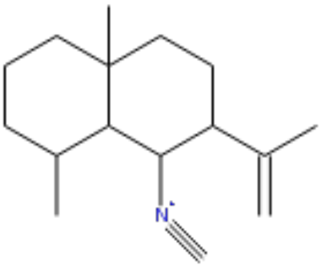

Cell ID	Cluster Center	Number of Compounds
504	0	6

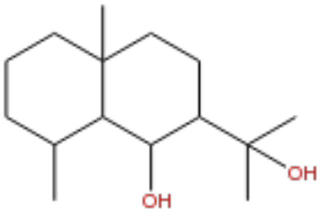

Cell ID	Cluster Center	Number of Compounds
504	0	6

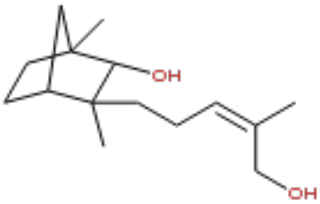

Cell ID	Cluster Center	Number of Compounds
505	1	4

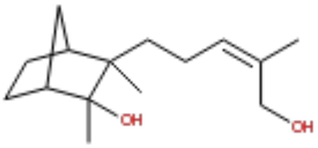

Cell ID	Cluster Center	Number of Compounds
505	0	4

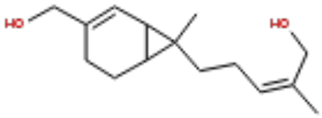

Cell ID	Cluster Center	Number of Compounds
505	0	4

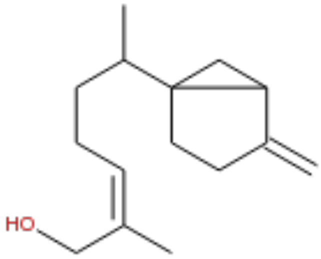

Cell ID	Cluster Center	Number of Compounds
505	0	4

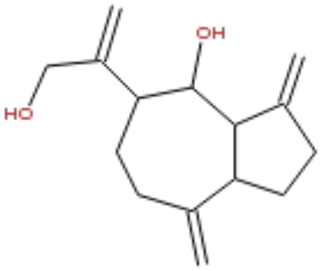

Cell ID	Cluster Center	Number of Compounds
506	1	1

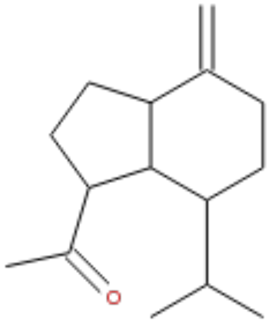

Cell ID	Cluster Center	Number of Compounds
509	1	14

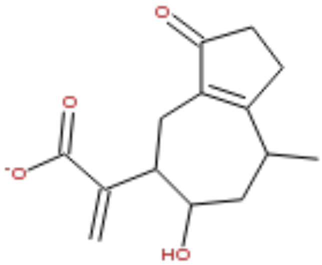

Cell ID	Cluster Center	Number of Compounds
509	0	14

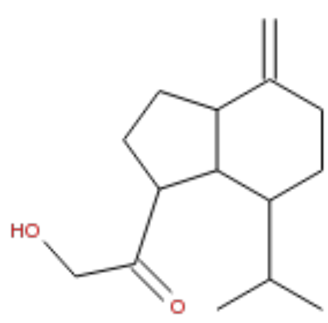

Cell ID	Cluster Center	Number of Compounds
509	0	14

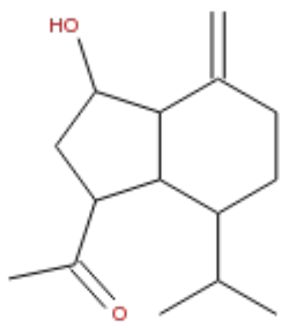

Cell ID	Cluster Center	Number of Compounds
509	0	14

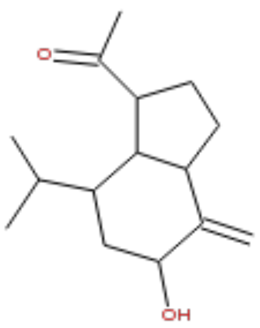

Cell ID	Cluster Center	Number of Compounds
509	0	14

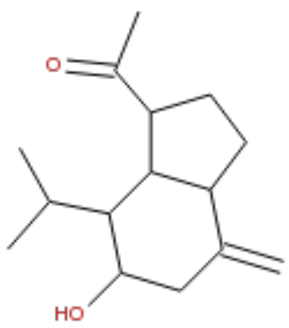

Cell ID	Cluster Center	Number of Compounds
509	0	14

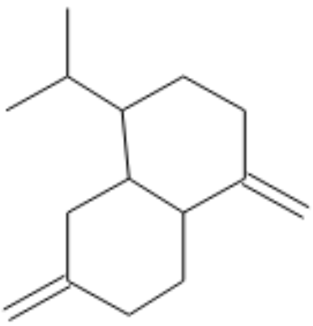

Cell ID	Cluster Center	Number of Compounds
509	0	14

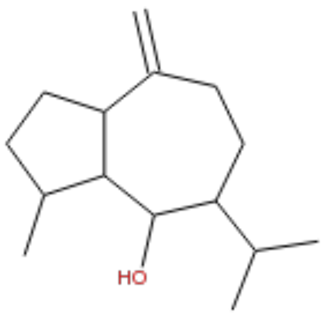

Cell ID	Cluster Center	Number of Compounds
509	0	14

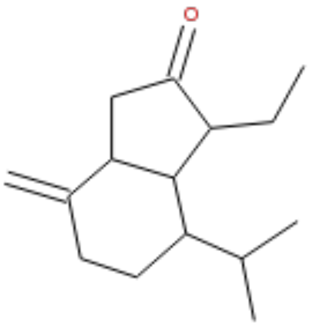

Cell ID	Cluster Center	Number of Compounds
509	0	14

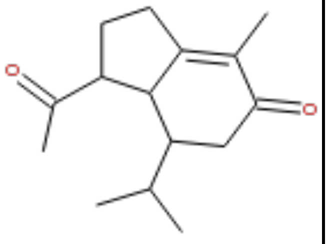

Cell ID	Cluster Center	Number of Compounds
509	0	14

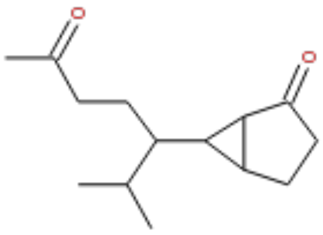

Cell ID	Cluster Center	Number of Compounds
509	0	14

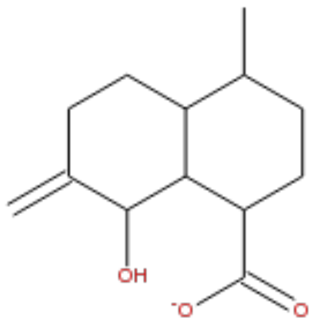

Cell ID	Cluster Center	Number of Compounds
509	0	14

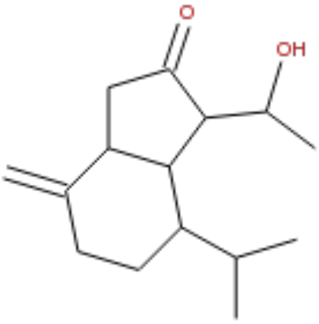

Cell ID	Cluster Center	Number of Compounds
509	0	14

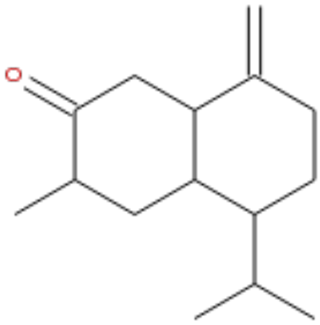

Cell ID	Cluster Center	Number of Compounds
509	0	14

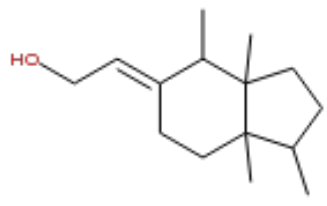

Cell ID	Cluster Center	Number of Compounds
510	1	3

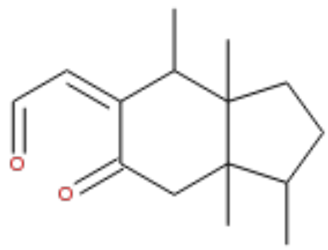

Cell ID	Cluster Center	Number of Compounds
510	0	3

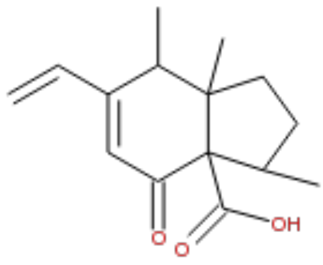

Cell ID	Cluster Center	Number of Compounds
510	0	3

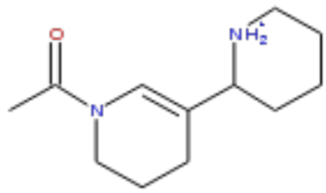

Cell ID	Cluster Center	Number of Compounds
511	1	6

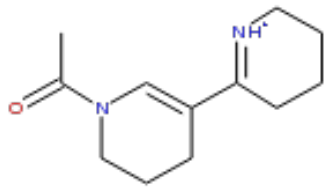

Cell ID	Cluster Center	Number of Compounds
511	0	6

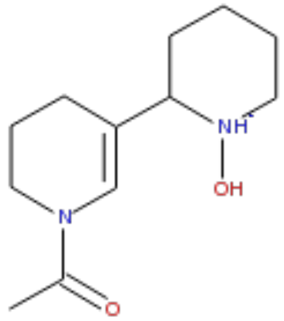

Cell ID	Cluster Center	Number of Compounds
511	0	6

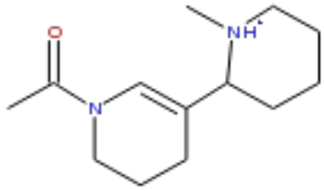

Cell ID	Cluster Center	Number of Compounds
511	0	6

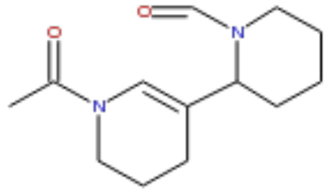

Cell ID	Cluster Center	Number of Compounds
511	0	6

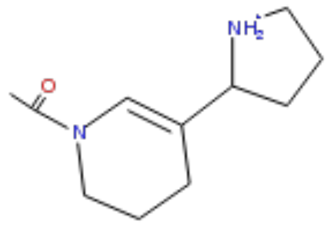

Cell ID	Cluster Center	Number of Compounds
511	0	6

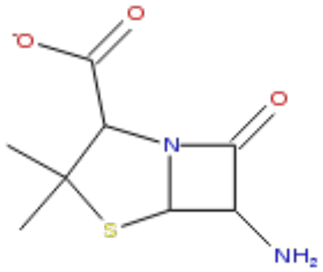

Cell ID	Cluster Center	Number of Compounds
512	1	5

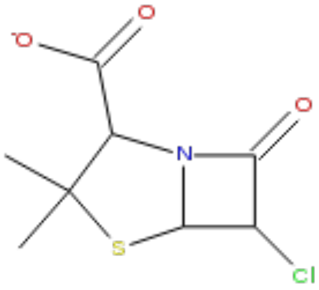

Cell ID	Cluster Center	Number of Compounds
512	0	5

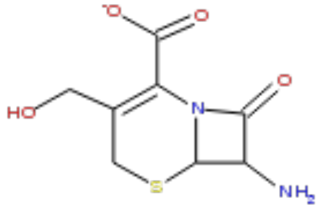

Cell ID	Cluster Center	Number of Compounds
512	0	5

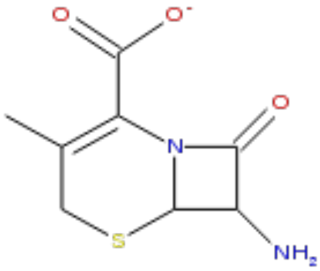

Cell ID	Cluster Center	Number of Compounds
512	0	5

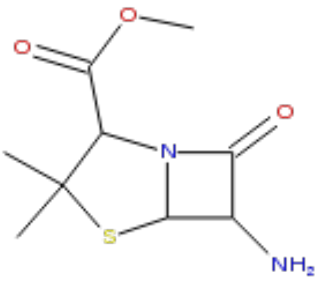

Cell ID	Cluster Center	Number of Compounds
512	0	5

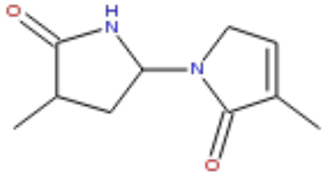

Cell ID	Cluster Center	Number of Compounds
515	1	5

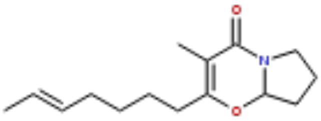

Cell ID	Cluster Center	Number of Compounds
515	0	5

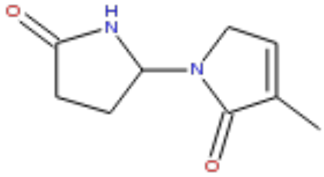

Cell ID	Cluster Center	Number of Compounds
515	0	5

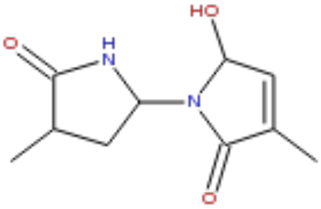

Cell ID	Cluster Center	Number of Compounds
515	0	5

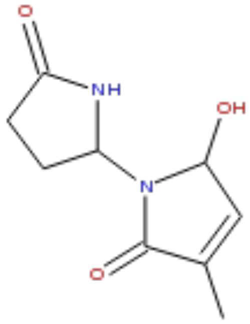

Cell ID	Cluster Center	Number of Compounds
515	0	5

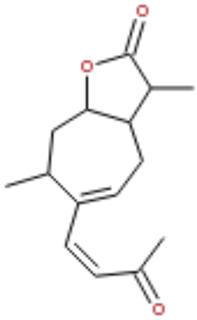

Cell ID	Cluster Center	Number of Compounds
516	1	1

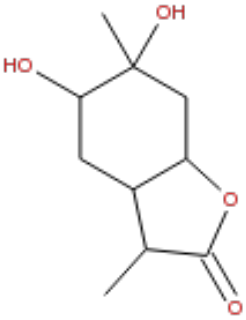

Cell ID	Cluster Center	Number of Compounds
517	1	2

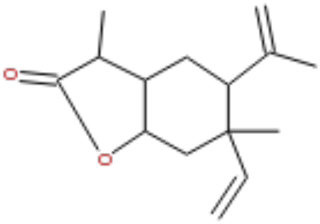

Cell ID	Cluster Center	Number of Compounds
517	0	2

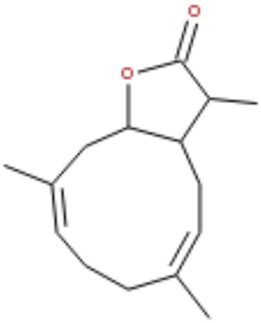

Cell ID	Cluster Center	Number of Compounds
518	1	2

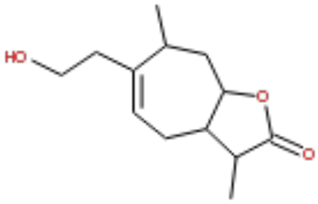

Cell ID	Cluster Center	Number of Compounds
518	0	2

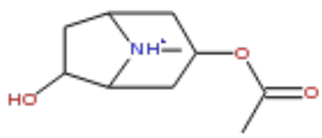

Cell ID	Cluster Center	Number of Compounds
520	1	8

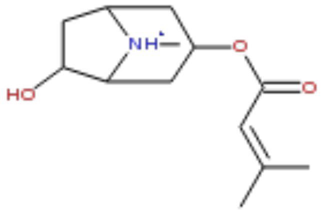

Cell ID	Cluster Center	Number of Compounds
520	0	8

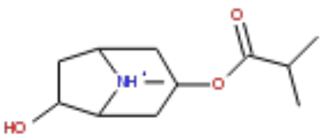

Cell ID	Cluster Center	Number of Compounds
520	0	8

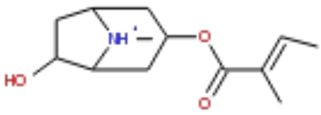

Cell ID	Cluster Center	Number of Compounds
520	0	8

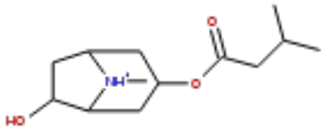

Cell ID	Cluster Center	Number of Compounds
520	0	8

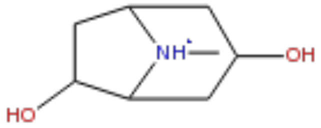

Cell ID	Cluster Center	Number of Compounds
520	0	8

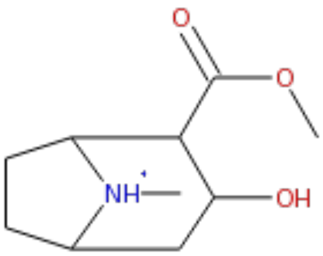

Cell ID	Cluster Center	Number of Compounds
520	0	8

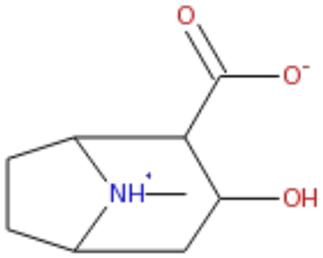

Cell ID	Cluster Center	Number of Compounds
520	0	8

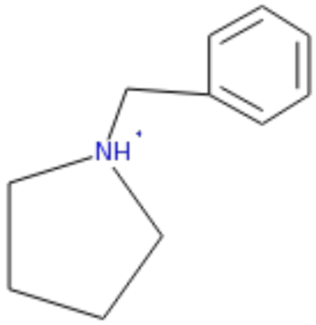

Cell ID	Cluster Center	Number of Compounds
521	1	3

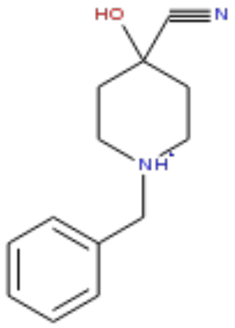

Cell ID	Cluster Center	Number of Compounds
521	0	3

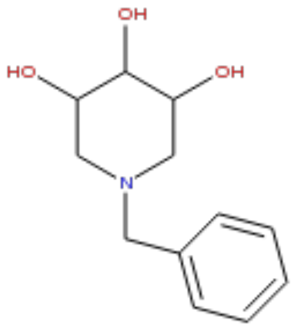

Cell ID	Cluster Center	Number of Compounds
521	0	3

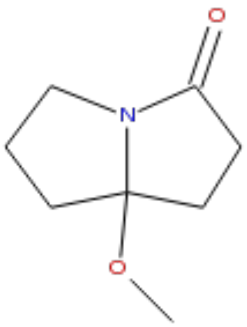

Cell ID	Cluster Center	Number of Compounds
525	1	17

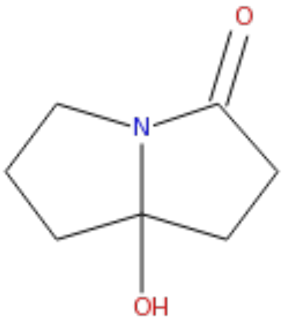

Cell ID	Cluster Center	Number of Compounds
525	0	17

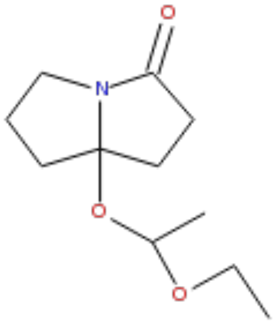

Cell ID	Cluster Center	Number of Compounds
525	0	17

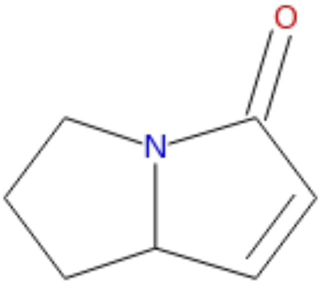

Cell ID	Cluster Center	Number of Compounds
525	0	17

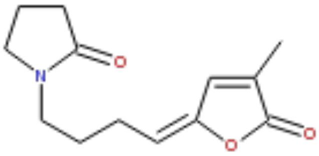

Cell ID	Cluster Center	Number of Compounds
525	0	17

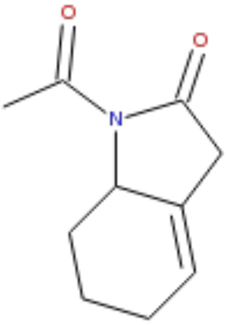

Cell ID	Cluster Center	Number of Compounds
525	0	17

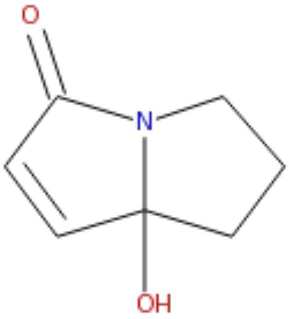

Cell ID	Cluster Center	Number of Compounds
525	0	17

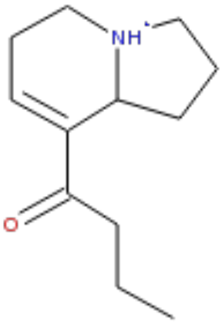

Cell ID	Cluster Center	Number of Compounds
525	0	17

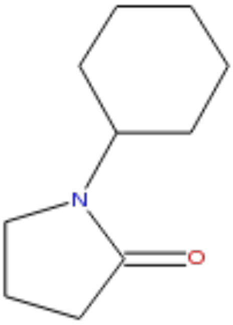

Cell ID	Cluster Center	Number of Compounds
525	0	17

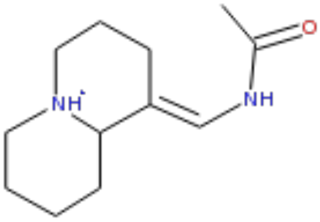

Cell ID	Cluster Center	Number of Compounds
525	0	17

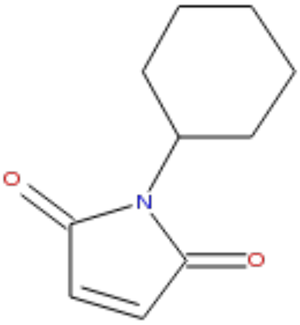

Cell ID	Cluster Center	Number of Compounds
525	0	17

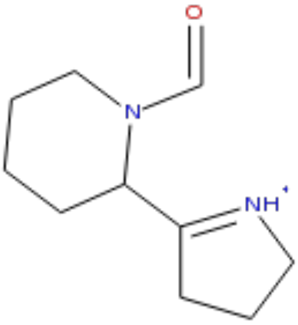

Cell ID	Cluster Center	Number of Compounds
525	0	17

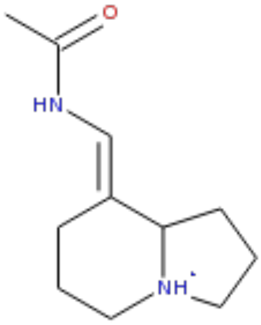

Cell ID	Cluster Center	Number of Compounds
525	0	17

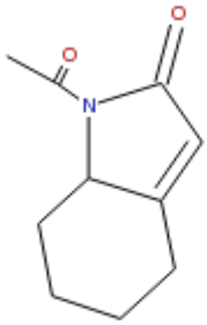

Cell ID	Cluster Center	Number of Compounds
525	0	17

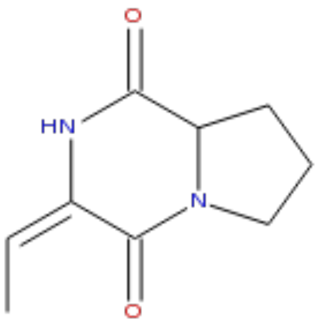

Cell ID	Cluster Center	Number of Compounds
525	0	17

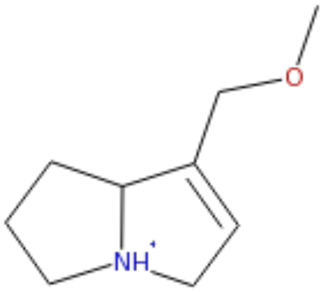

Cell ID	Cluster Center	Number of Compounds
525	0	17

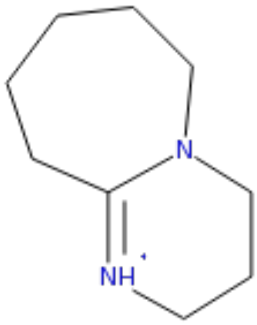

Cell ID	Cluster Center	Number of Compounds
525	0	17

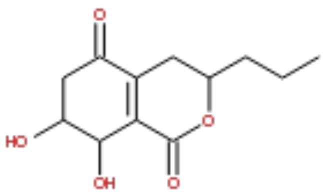

Cell ID	Cluster Center	Number of Compounds
526	1	1

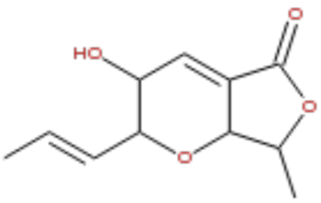

Cell ID	Cluster Center	Number of Compounds
528	1	6

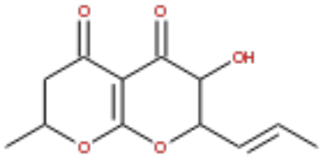

Cell ID	Cluster Center	Number of Compounds
528	0	6

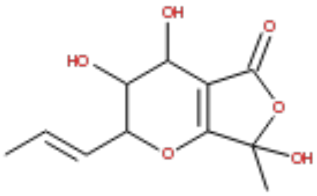

Cell ID	Cluster Center	Number of Compounds
528	0	6

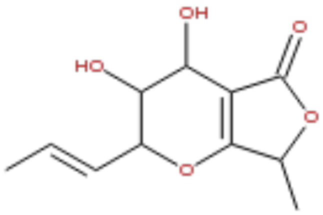

Cell ID	Cluster Center	Number of Compounds
528	0	6

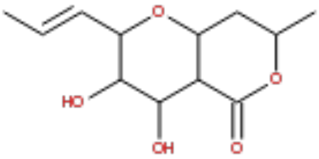

Cell ID	Cluster Center	Number of Compounds
528	0	6

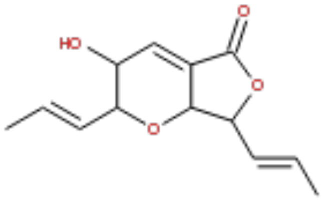

Cell ID	Cluster Center	Number of Compounds
528	0	6

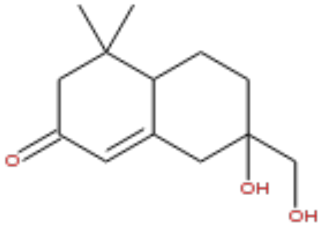

Cell ID	Cluster Center	Number of Compounds
529	1	2

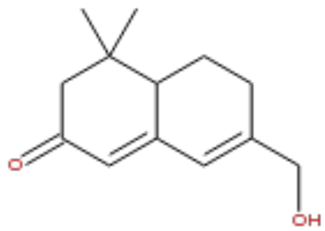

Cell ID	Cluster Center	Number of Compounds
529	0	2

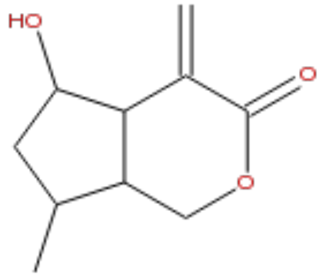

Cell ID	Cluster Center	Number of Compounds
533	1	4

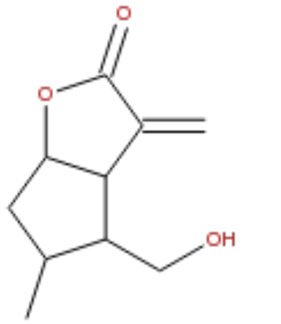

Cell ID	Cluster Center	Number of Compounds
533	0	4

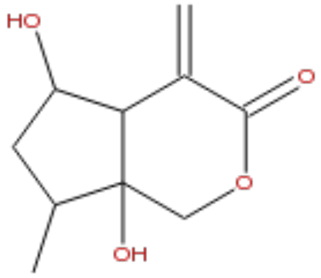

Cell ID	Cluster Center	Number of Compounds
533	0	4

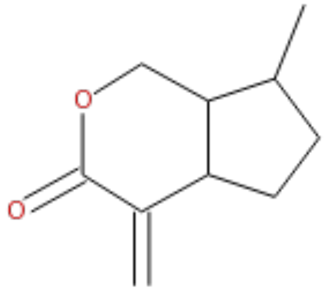

Cell ID	Cluster Center	Number of Compounds
533	0	4

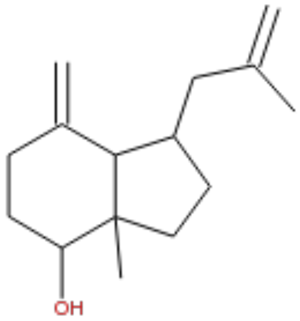

Cell ID	Cluster Center	Number of Compounds
536	1	14

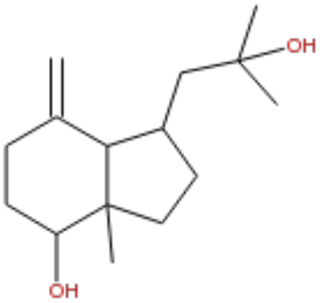

Cell ID	Cluster Center	Number of Compounds
536	0	14

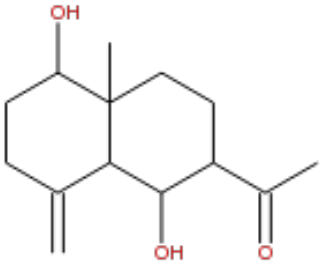

Cell ID	Cluster Center	Number of Compounds
536	0	14

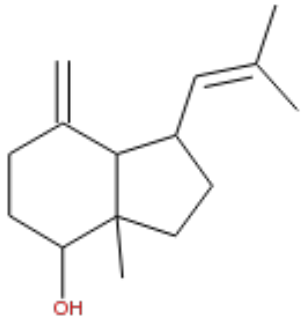

Cell ID	Cluster Center	Number of Compounds
536	0	14

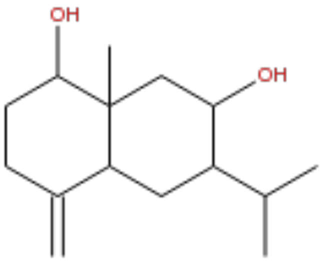

Cell ID	Cluster Center	Number of Compounds
536	0	14

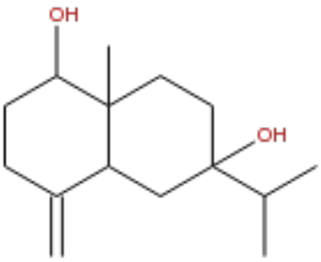

Cell ID	Cluster Center	Number of Compounds
536	0	14

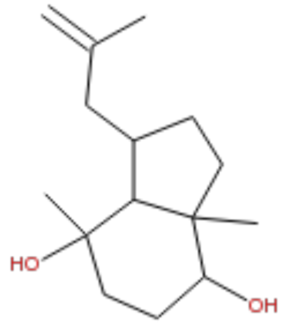

Cell ID	Cluster Center	Number of Compounds
536	0	14

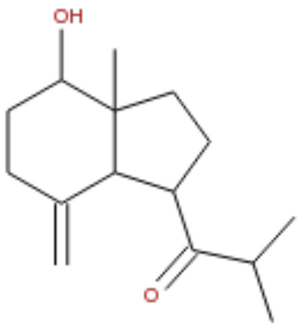

Cell ID	Cluster Center	Number of Compounds
536	0	14

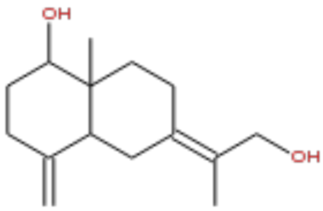

Cell ID	Cluster Center	Number of Compounds
536	0	14

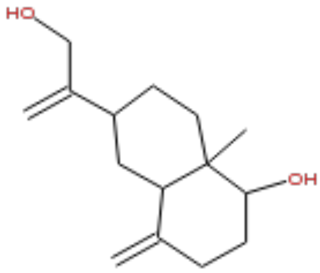

Cell ID	Cluster Center	Number of Compounds
536	0	14

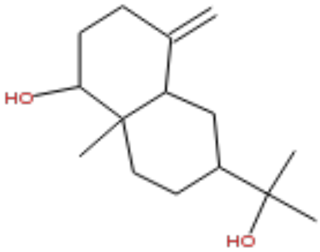

Cell ID	Cluster Center	Number of Compounds
536	0	14

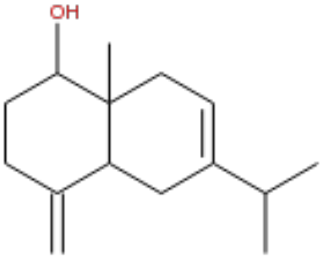

Cell ID	Cluster Center	Number of Compounds
536	0	14

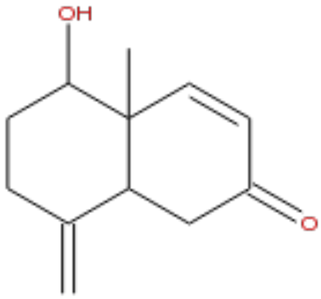

Cell ID	Cluster Center	Number of Compounds
536	0	14

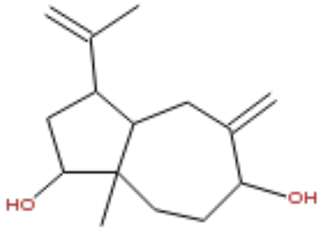

Cell ID	Cluster Center	Number of Compounds
536	0	14

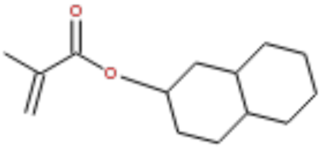

Cell ID	Cluster Center	Number of Compounds
539	1	1

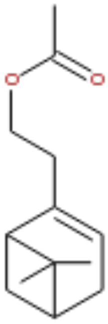

Cell ID	Cluster Center	Number of Compounds
540	1	10

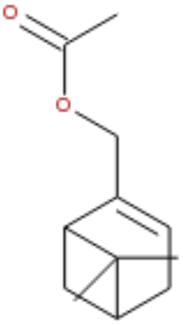

Cell ID	Cluster Center	Number of Compounds
540	0	10

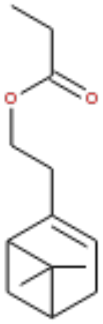

Cell ID	Cluster Center	Number of Compounds
540	0	10

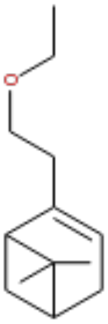

Cell ID	Cluster Center	Number of Compounds
540	0	10

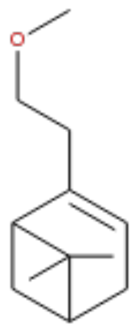

Cell ID	Cluster Center	Number of Compounds
540	0	10

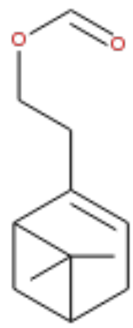

Cell ID	Cluster Center	Number of Compounds
540	0	10

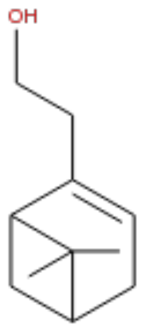

Cell ID	Cluster Center	Number of Compounds
540	0	10

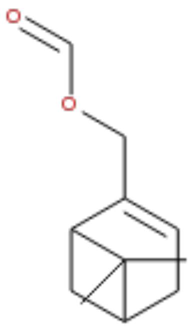

Cell ID	Cluster Center	Number of Compounds
540	0	10

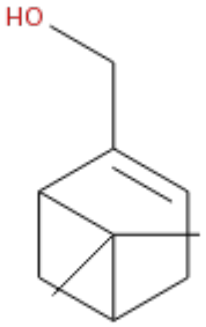

Cell ID	Cluster Center	Number of Compounds
540	0	10

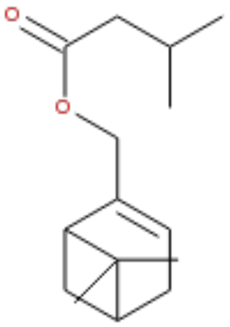

Cell ID	Cluster Center	Number of Compounds
540	0	10

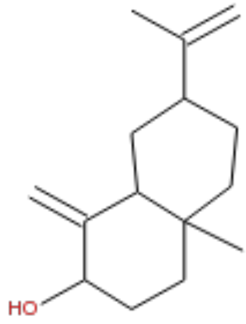

Cell ID	Cluster Center	Number of Compounds
542	1	16

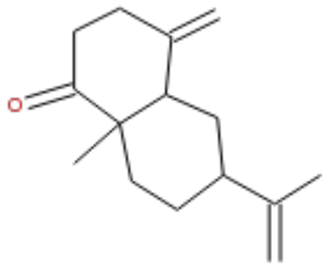

Cell ID	Cluster Center	Number of Compounds
542	0	16

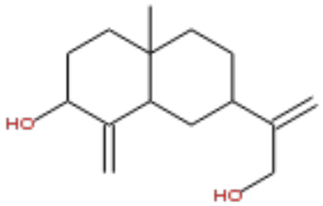

Cell ID	Cluster Center	Number of Compounds
542	0	16

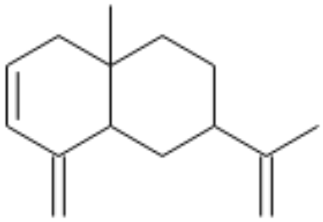

Cell ID	Cluster Center	Number of Compounds
542	0	16

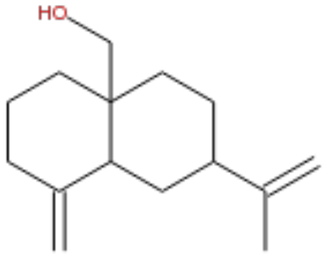

Cell ID	Cluster Center	Number of Compounds
542	0	16

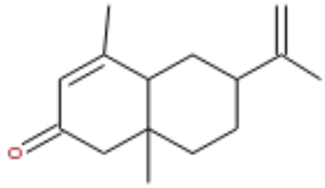

Cell ID	Cluster Center	Number of Compounds
542	0	16

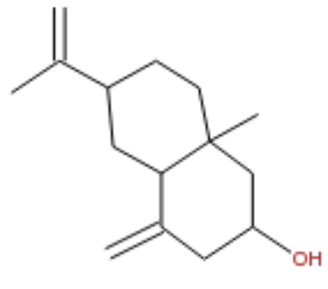

Cell ID	Cluster Center	Number of Compounds
542	0	16

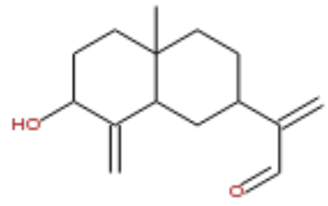

Cell ID	Cluster Center	Number of Compounds
542	0	16

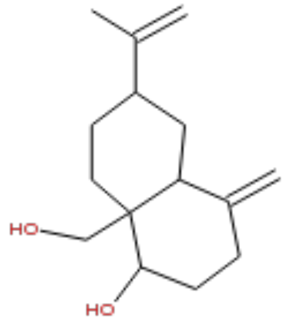

Cell ID	Cluster Center	Number of Compounds
542	0	16

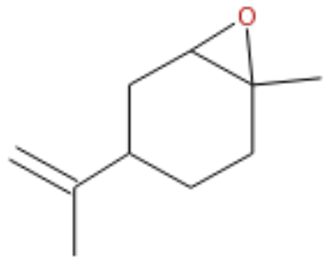

Cell ID	Cluster Center	Number of Compounds
542	0	16

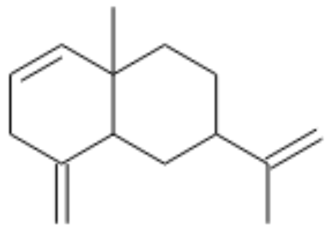

Cell ID	Cluster Center	Number of Compounds
542	0	16

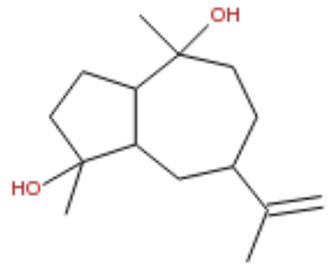

Cell ID	Cluster Center	Number of Compounds
542	0	16

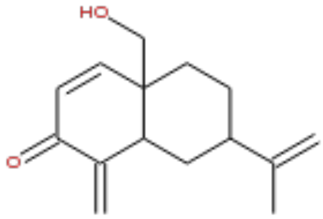

Cell ID	Cluster Center	Number of Compounds
542	0	16

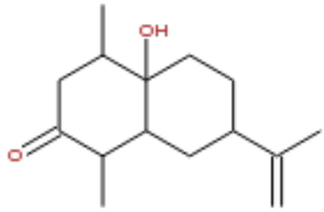

Cell ID	Cluster Center	Number of Compounds
542	0	16

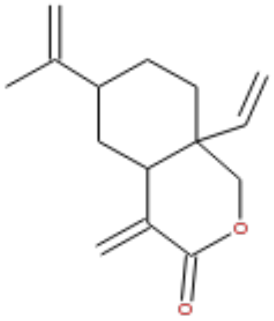

Cell ID	Cluster Center	Number of Compounds
542	0	16

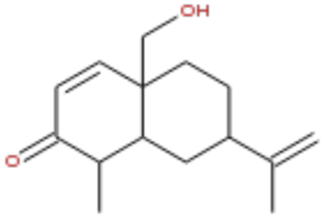

Cell ID	Cluster Center	Number of Compounds
542	0	16

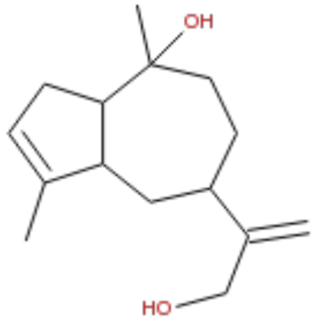

Cell ID	Cluster Center	Number of Compounds
543	1	11

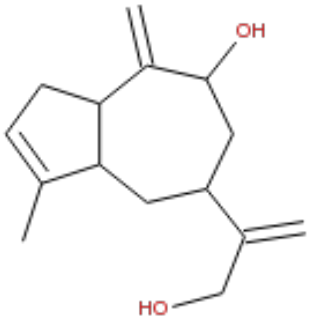

Cell ID	Cluster Center	Number of Compounds
543	0	11

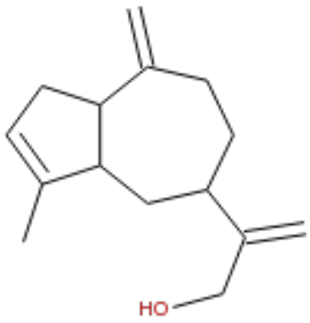

Cell ID	Cluster Center	Number of Compounds
543	0	11

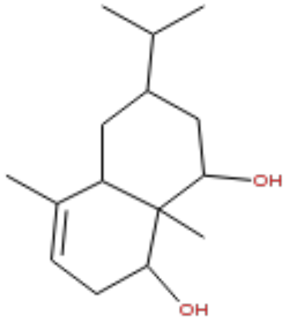

Cell ID	Cluster Center	Number of Compounds
543	0	11

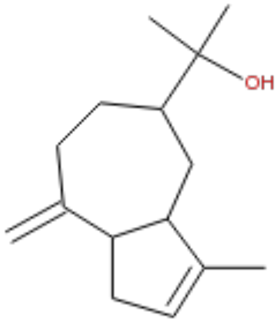

Cell ID	Cluster Center	Number of Compounds
543	0	11

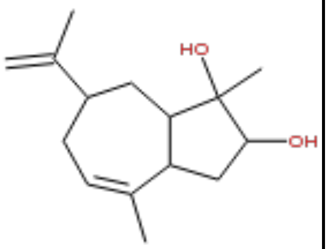

Cell ID	Cluster Center	Number of Compounds
543	0	11

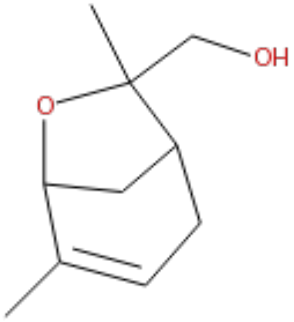

Cell ID	Cluster Center	Number of Compounds
543	0	11

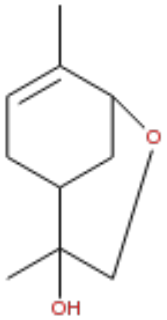

Cell ID	Cluster Center	Number of Compounds
543	0	11

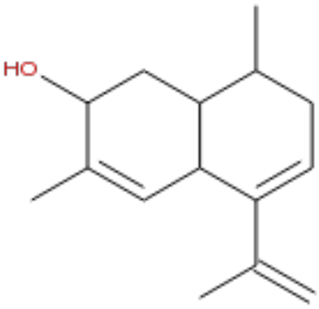

Cell ID	Cluster Center	Number of Compounds
543	0	11

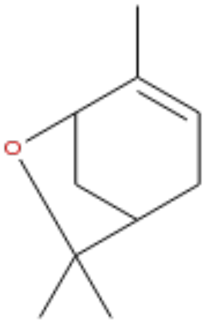

Cell ID	Cluster Center	Number of Compounds
543	0	11

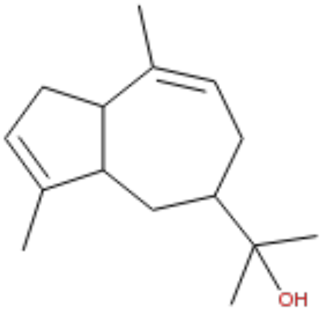

Cell ID	Cluster Center	Number of Compounds
543	0	11

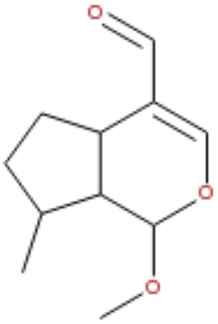

Cell ID	Cluster Center	Number of Compounds
545	1	11

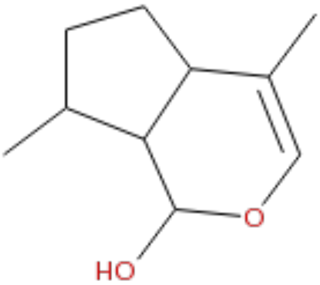

Cell ID	Cluster Center	Number of Compounds
545	0	11

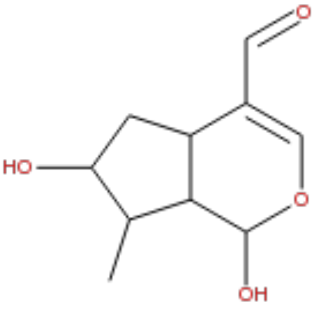

Cell ID	Cluster Center	Number of Compounds
545	0	11

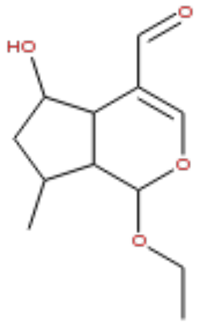

Cell ID	Cluster Center	Number of Compounds
545	0	11

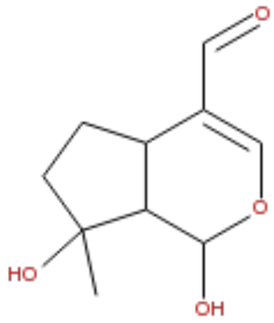

Cell ID	Cluster Center	Number of Compounds
545	0	11

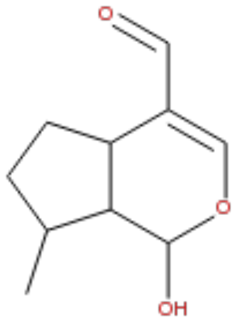

Cell ID	Cluster Center	Number of Compounds
545	0	11

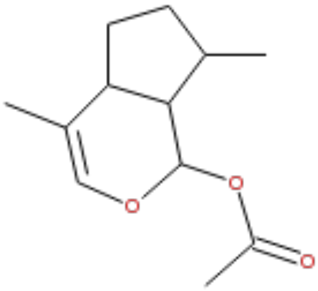

Cell ID	Cluster Center	Number of Compounds
545	0	11

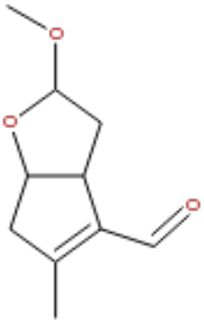

Cell ID	Cluster Center	Number of Compounds
545	0	11

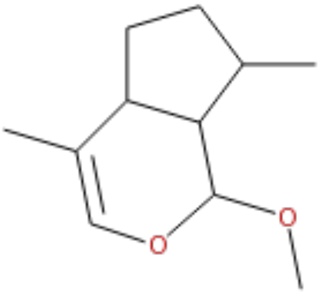

Cell ID	Cluster Center	Number of Compounds
545	0	11

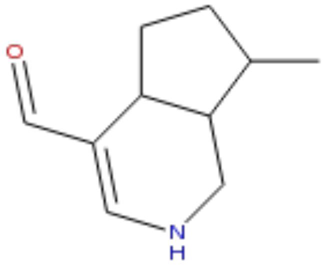

Cell ID	Cluster Center	Number of Compounds
545	0	11

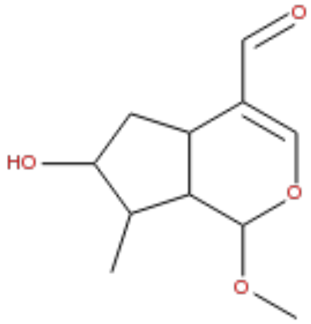

Cell ID	Cluster Center	Number of Compounds
545	0	11

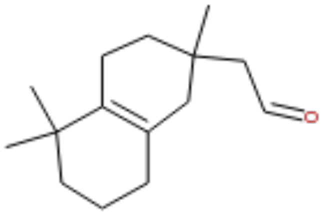

Cell ID	Cluster Center	Number of Compounds
547	1	2

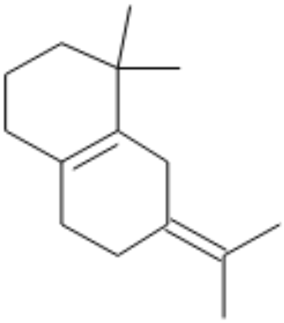

Cell ID	Cluster Center	Number of Compounds
547	0	2

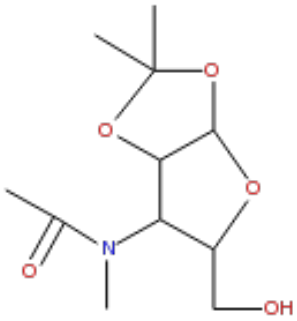

Cell ID	Cluster Center	Number of Compounds
548	1	1

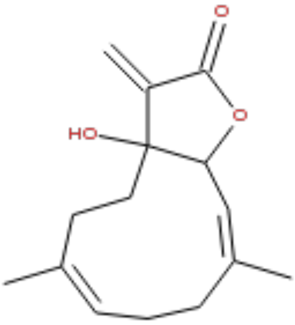

Cell ID	Cluster Center	Number of Compounds
549	1	1

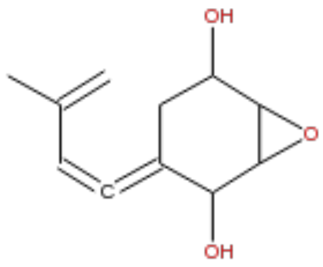

Cell ID	Cluster Center	Number of Compounds
550	1	1

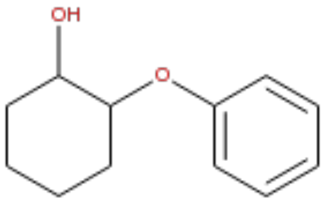

Cell ID	Cluster Center	Number of Compounds
551	1	3

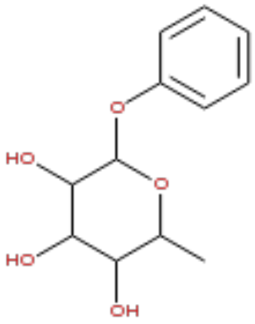

Cell ID	Cluster Center	Number of Compounds
551	0	3

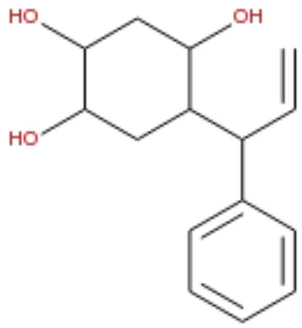

Cell ID	Cluster Center	Number of Compounds
551	0	3

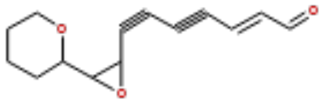

Cell ID	Cluster Center	Number of Compounds
552	1	2

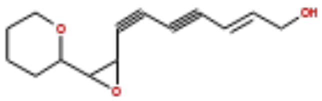

Cell ID	Cluster Center	Number of Compounds
552	0	2

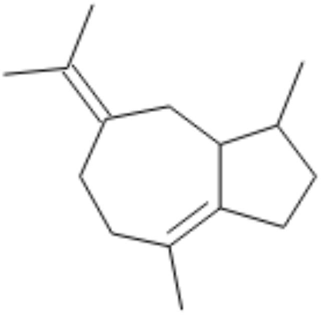

Cell ID	Cluster Center	Number of Compounds
554	1	7

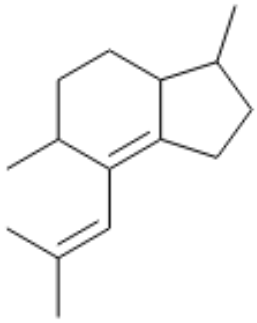

Cell ID	Cluster Center	Number of Compounds
554	0	7

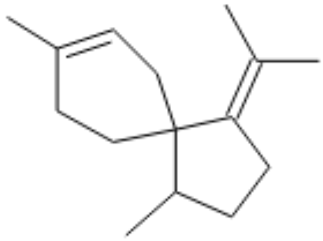

Cell ID	Cluster Center	Number of Compounds
554	0	7

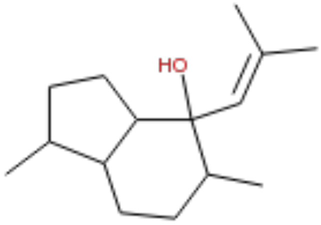

Cell ID	Cluster Center	Number of Compounds
554	0	7

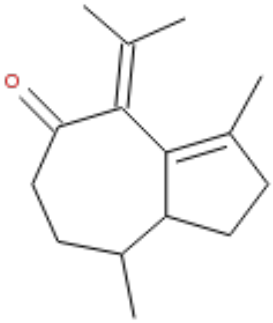

Cell ID	Cluster Center	Number of Compounds
554	0	7

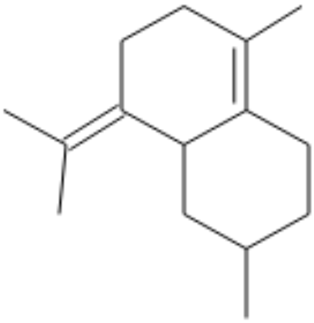

Cell ID	Cluster Center	Number of Compounds
554	0	7

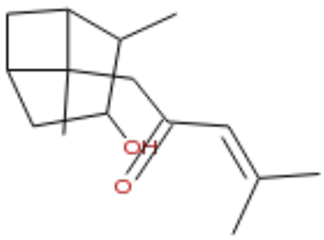

Cell ID	Cluster Center	Number of Compounds
554	0	7

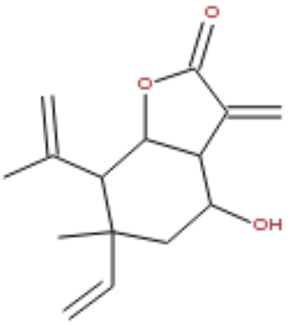

Cell ID	Cluster Center	Number of Compounds
557	1	1

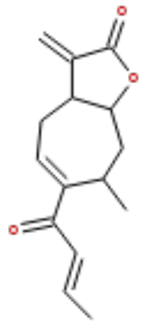

Cell ID	Cluster Center	Number of Compounds
563	1	6

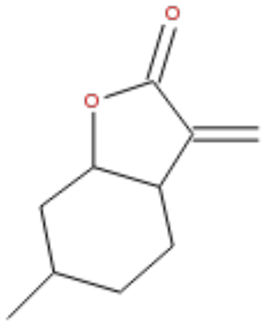

Cell ID	Cluster Center	Number of Compounds
563	0	6

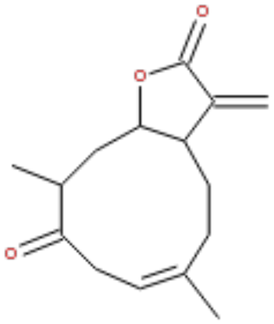

Cell ID	Cluster Center	Number of Compounds
563	0	6

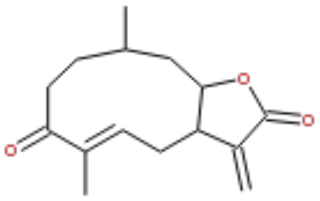

Cell ID	Cluster Center	Number of Compounds
563	0	6

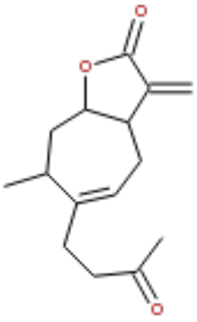

Cell ID	Cluster Center	Number of Compounds
563	0	6

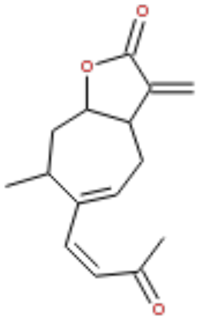

Cell ID	Cluster Center	Number of Compounds
563	0	6

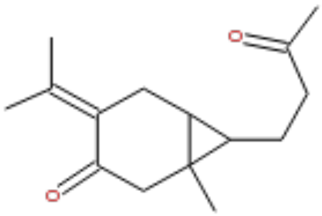

Cell ID	Cluster Center	Number of Compounds
564	1	4

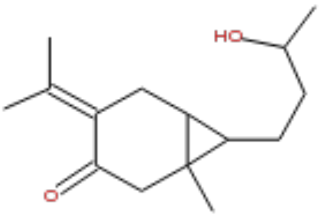

Cell ID	Cluster Center	Number of Compounds
564	0	4

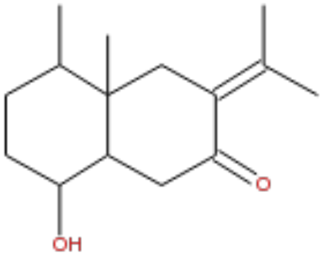

Cell ID	Cluster Center	Number of Compounds
564	0	4

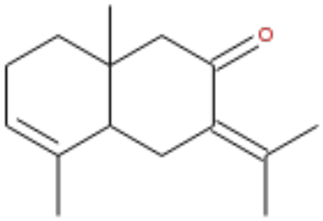

Cell ID	Cluster Center	Number of Compounds
564	0	4

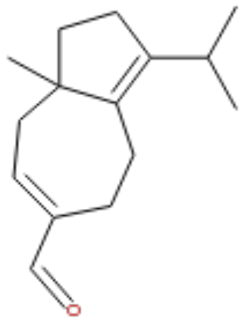

Cell ID	Cluster Center	Number of Compounds
565	1	4

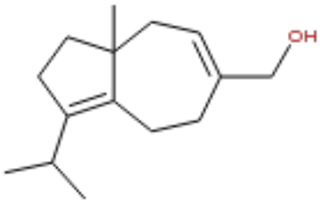

Cell ID	Cluster Center	Number of Compounds
565	0	4

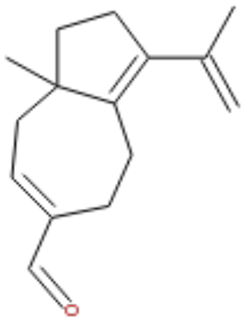

Cell ID	Cluster Center	Number of Compounds
565	0	4

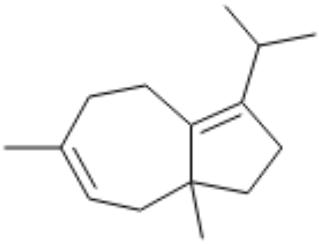

Cell ID	Cluster Center	Number of Compounds
565	0	4

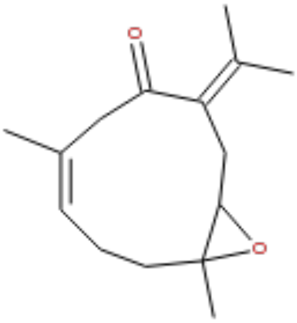

Cell ID	Cluster Center	Number of Compounds
566	1	2

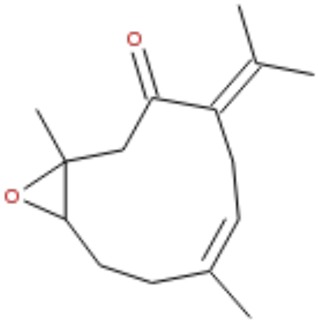

Cell ID	Cluster Center	Number of Compounds
566	0	2

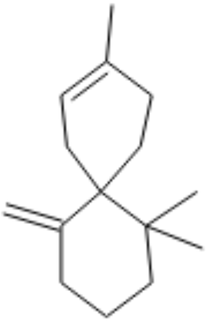

Cell ID	Cluster Center	Number of Compounds
567	1	21

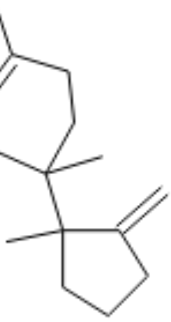

Cell ID	Cluster Center	Number of Compounds
567	0	21

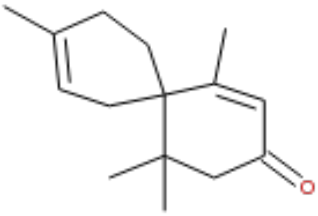

Cell ID	Cluster Center	Number of Compounds
567	0	21

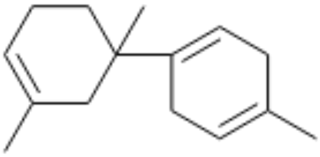

Cell ID	Cluster Center	Number of Compounds
567	0	21

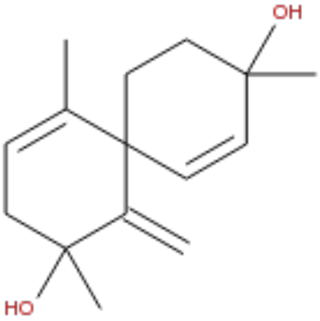

Cell ID	Cluster Center	Number of Compounds
567	0	21

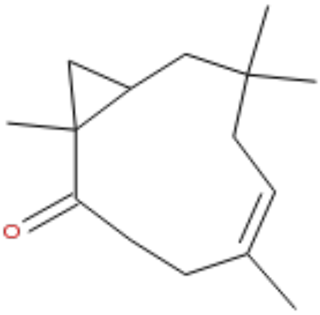

Cell ID	Cluster Center	Number of Compounds
567	0	21

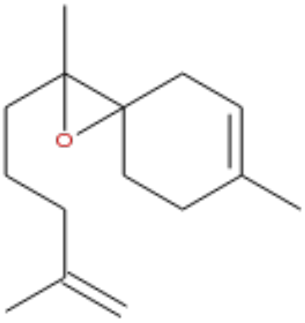

Cell ID	Cluster Center	Number of Compounds
567	0	21

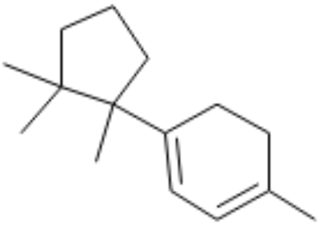

Cell ID	Cluster Center	Number of Compounds
567	0	21

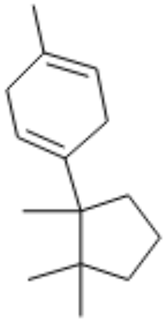

Cell ID	Cluster Center	Number of Compounds
567	0	21

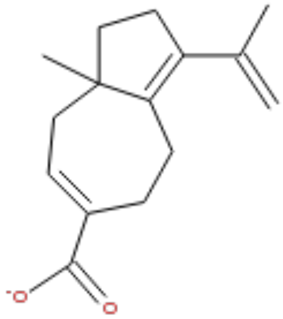

Cell ID	Cluster Center	Number of Compounds
567	0	21

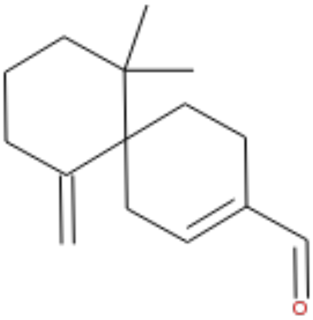

Cell ID	Cluster Center	Number of Compounds
567	0	21

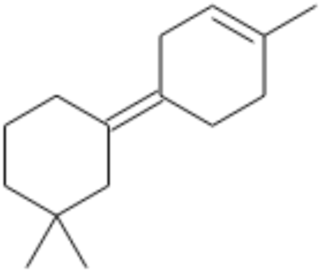

Cell ID	Cluster Center	Number of Compounds
567	0	21

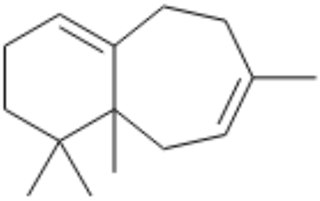

Cell ID	Cluster Center	Number of Compounds
567	0	21

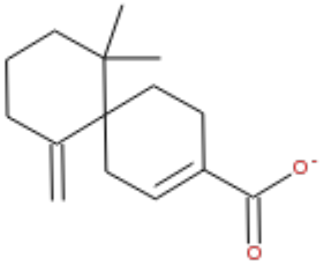

Cell ID	Cluster Center	Number of Compounds
567	0	21

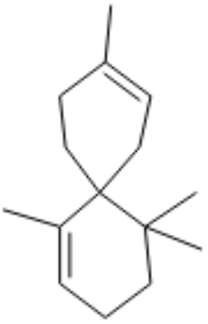

Cell ID	Cluster Center	Number of Compounds
567	0	21

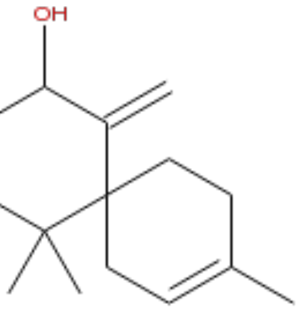

Cell ID	Cluster Center	Number of Compounds
567	0	21

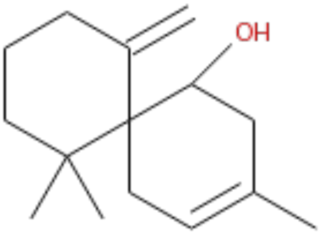

Cell ID	Cluster Center	Number of Compounds
567	0	21

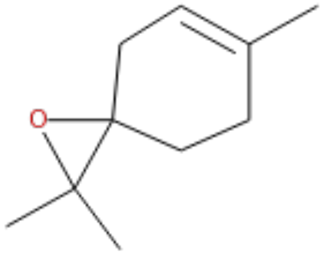

Cell ID	Cluster Center	Number of Compounds
567	0	21

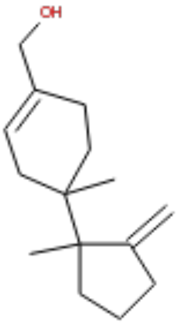

Cell ID	Cluster Center	Number of Compounds
567	0	21

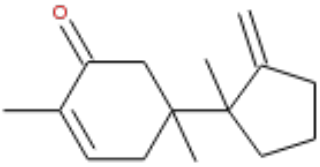

Cell ID	Cluster Center	Number of Compounds
567	0	21

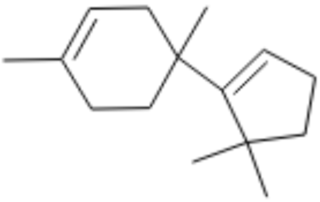

Cell ID	Cluster Center	Number of Compounds
567	0	21

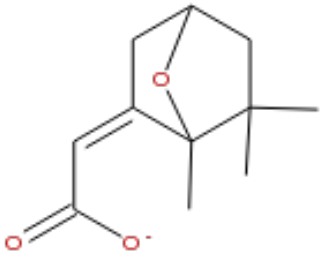

Cell ID	Cluster Center	Number of Compounds
568	1	2

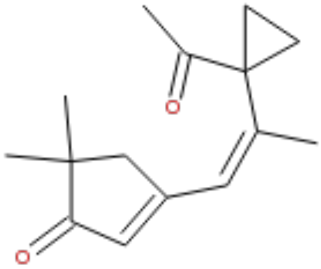

Cell ID	Cluster Center	Number of Compounds
568	0	2

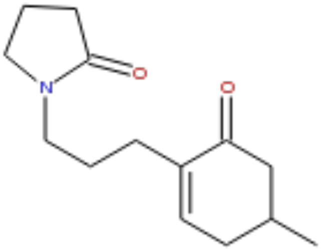

Cell ID	Cluster Center	Number of Compounds
570	1	1

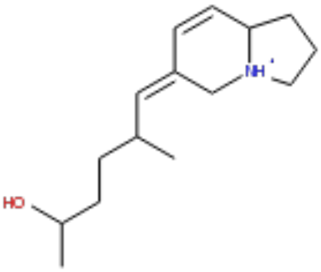

Cell ID	Cluster Center	Number of Compounds
571	1	3

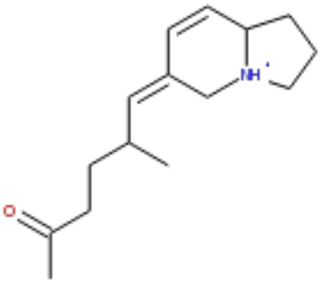

Cell ID	Cluster Center	Number of Compounds
571	0	3

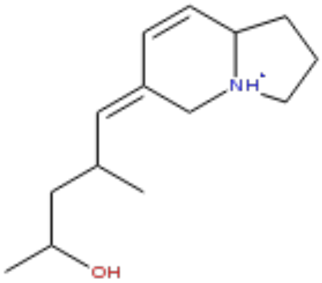

Cell ID	Cluster Center	Number of Compounds
571	0	3

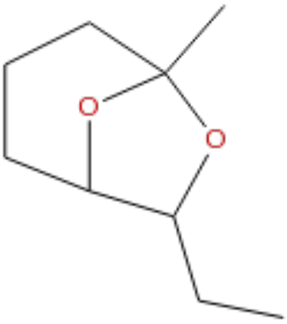

Cell ID	Cluster Center	Number of Compounds
572	1	26

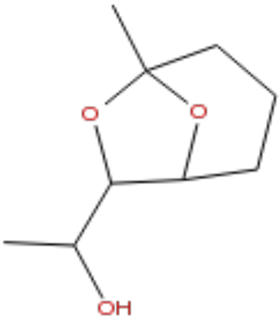

Cell ID	Cluster Center	Number of Compounds
572	0	26

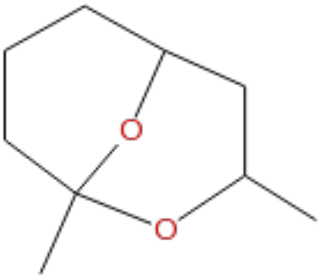

Cell ID	Cluster Center	Number of Compounds
572	0	26

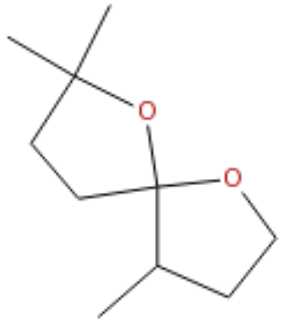

Cell ID	Cluster Center	Number of Compounds
572	0	26

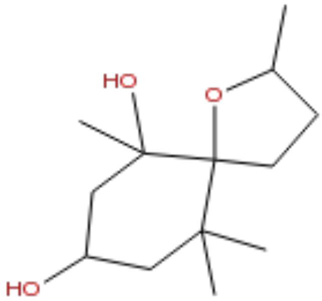

Cell ID	Cluster Center	Number of Compounds
572	0	26

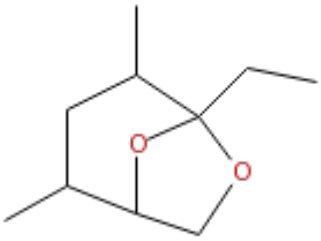

Cell ID	Cluster Center	Number of Compounds
572	0	26

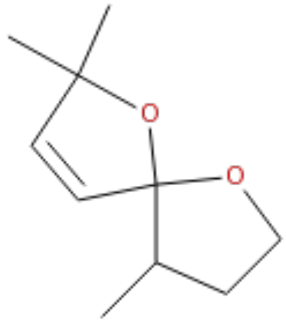

Cell ID	Cluster Center	Number of Compounds
572	0	26

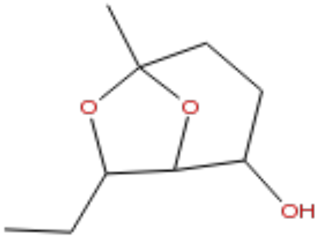

Cell ID	Cluster Center	Number of Compounds
572	0	26

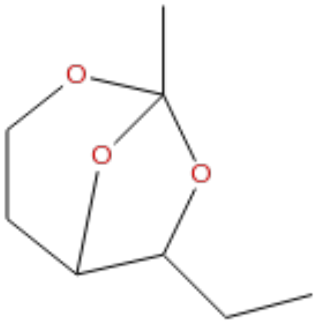

Cell ID	Cluster Center	Number of Compounds
572	0	26

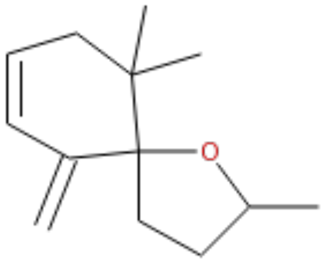

Cell ID	Cluster Center	Number of Compounds
572	0	26

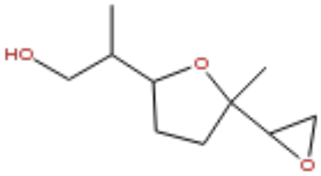

Cell ID	Cluster Center	Number of Compounds
572	0	26

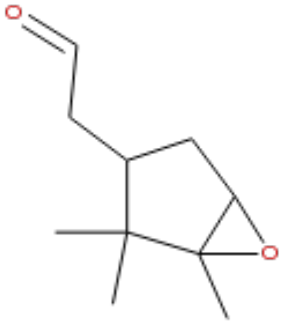

Cell ID	Cluster Center	Number of Compounds
572	0	26

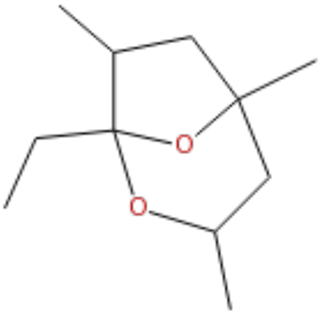

Cell ID	Cluster Center	Number of Compounds
572	0	26

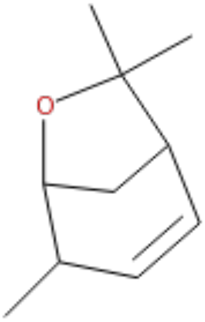

Cell ID	Cluster Center	Number of Compounds
572	0	26

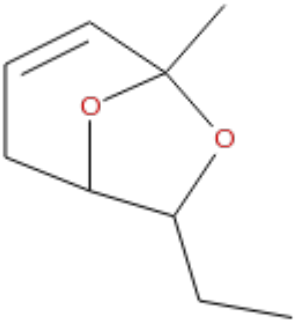

Cell ID	Cluster Center	Number of Compounds
572	0	26

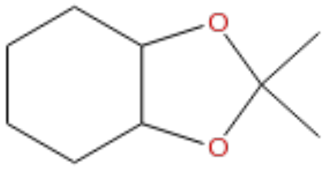

Cell ID	Cluster Center	Number of Compounds
572	0	26

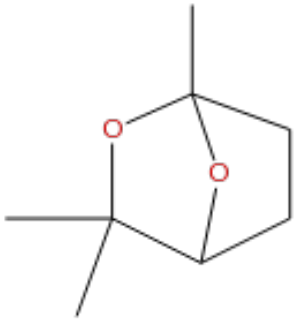

Cell ID	Cluster Center	Number of Compounds
572	0	26

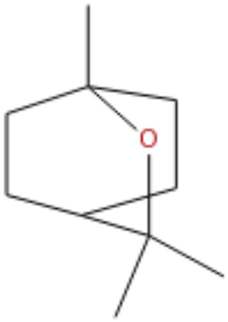

Cell ID	Cluster Center	Number of Compounds
572	0	26

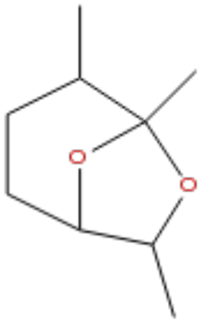

Cell ID	Cluster Center	Number of Compounds
572	0	26

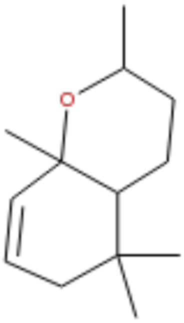

Cell ID	Cluster Center	Number of Compounds
572	0	26

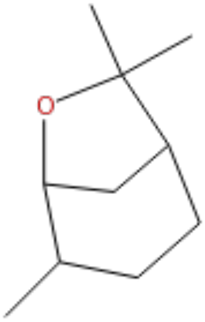

Cell ID	Cluster Center	Number of Compounds
572	0	26

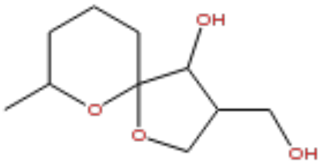

Cell ID	Cluster Center	Number of Compounds
572	0	26

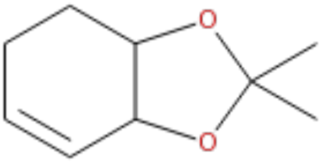

Cell ID	Cluster Center	Number of Compounds
572	0	26

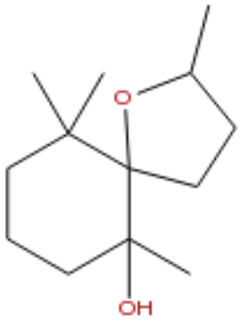

Cell ID	Cluster Center	Number of Compounds
572	0	26

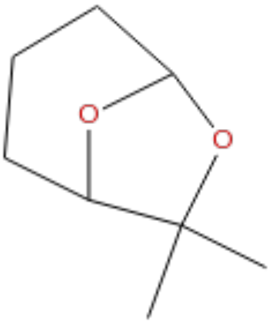

Cell ID	Cluster Center	Number of Compounds
572	0	26

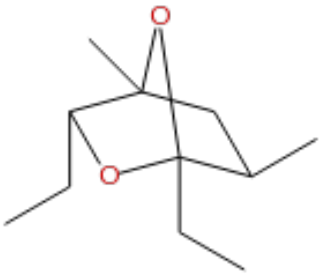

Cell ID	Cluster Center	Number of Compounds
572	0	26

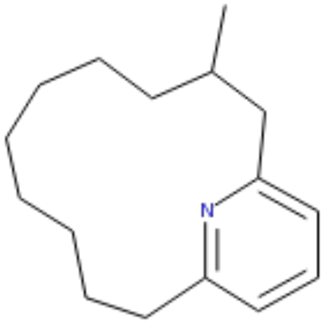

Cell ID	Cluster Center	Number of Compounds
573	1	3

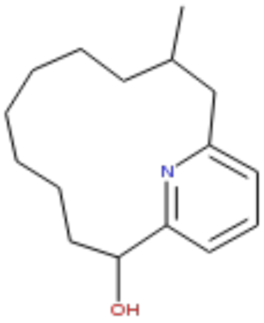

Cell ID	Cluster Center	Number of Compounds
573	0	3

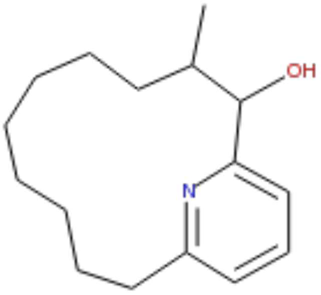

Cell ID	Cluster Center	Number of Compounds
573	0	3

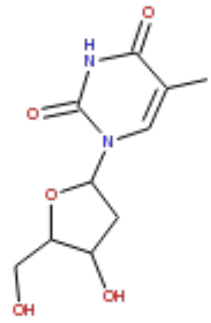

Cell ID	Cluster Center	Number of Compounds
574	1	8

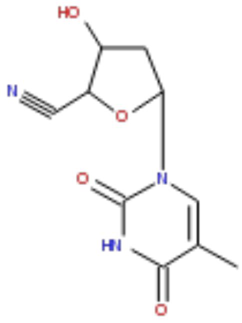

Cell ID	Cluster Center	Number of Compounds
574	0	8

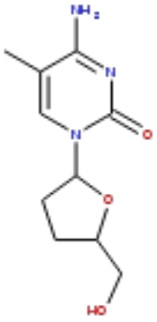

Cell ID	Cluster Center	Number of Compounds
574	0	8

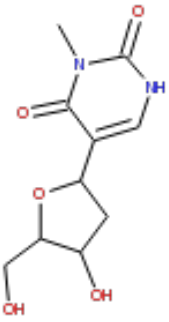

Cell ID	Cluster Center	Number of Compounds
574	0	8

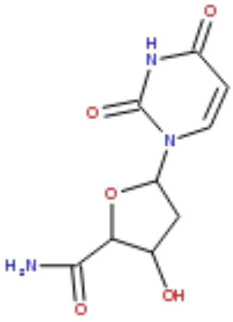

Cell ID	Cluster Center	Number of Compounds
574	0	8

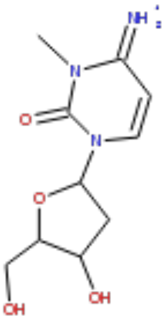

Cell ID	Cluster Center	Number of Compounds
574	0	8

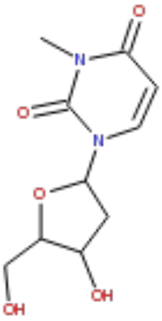

Cell ID	Cluster Center	Number of Compounds
574	0	8

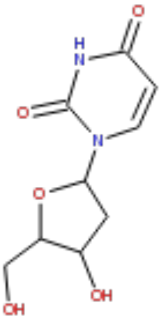

Cell ID	Cluster Center	Number of Compounds
574	0	8

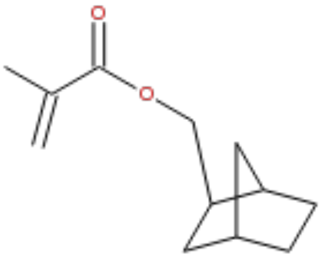

Cell ID	Cluster Center	Number of Compounds
576	1	1

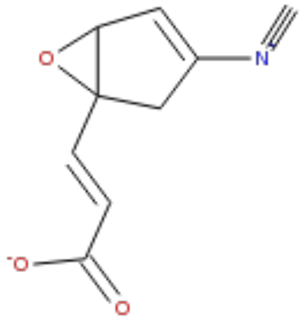

Cell ID	Cluster Center	Number of Compounds
577	1	3

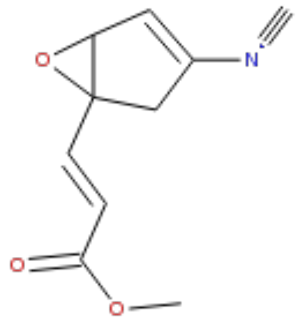

Cell ID	Cluster Center	Number of Compounds
577	0	3

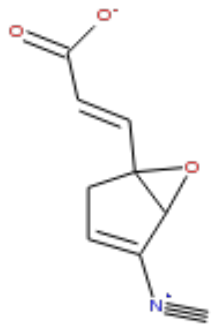

Cell ID	Cluster Center	Number of Compounds
577	0	3

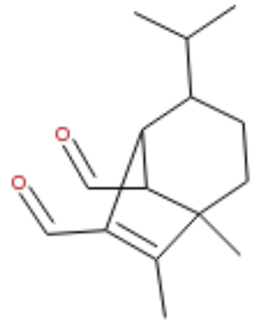

Cell ID	Cluster Center	Number of Compounds
578	1	5

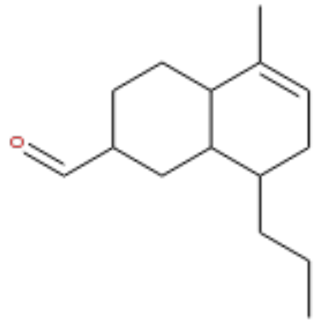

Cell ID	Cluster Center	Number of Compounds
578	0	5

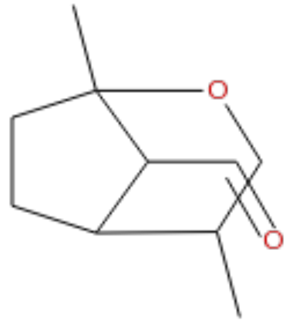

Cell ID	Cluster Center	Number of Compounds
578	0	5

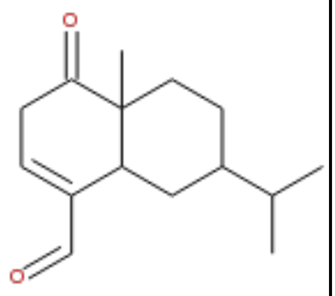

Cell ID	Cluster Center	Number of Compounds
578	0	5

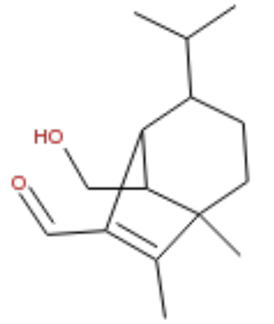

Cell ID	Cluster Center	Number of Compounds
578	0	5

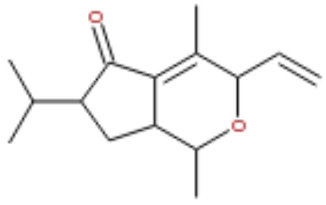

Cell ID	Cluster Center	Number of Compounds
579	1	4

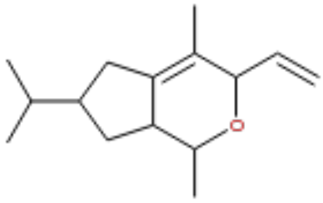

Cell ID	Cluster Center	Number of Compounds
579	0	4

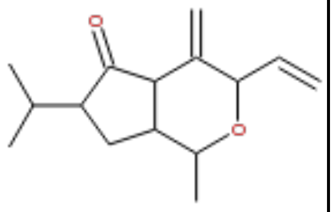

Cell ID	Cluster Center	Number of Compounds
579	0	4

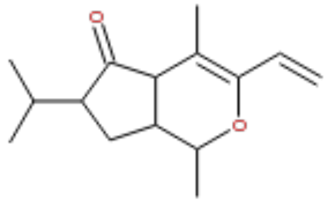

Cell ID	Cluster Center	Number of Compounds
579	0	4

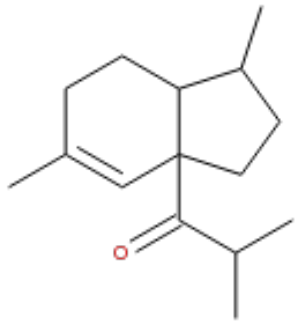

Cell ID	Cluster Center	Number of Compounds
580	1	1

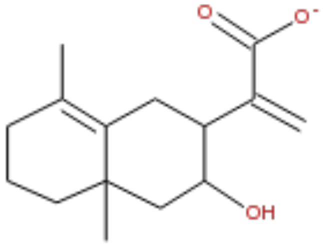

Cell ID	Cluster Center	Number of Compounds
583	1	2

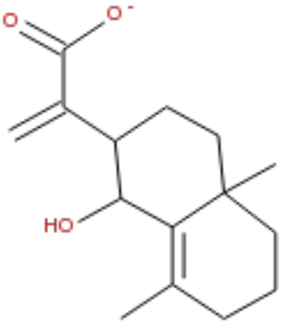

Cell ID	Cluster Center	Number of Compounds
583	0	2

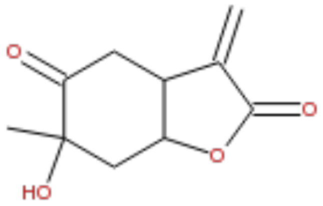

Cell ID	Cluster Center	Number of Compounds
584	1	1

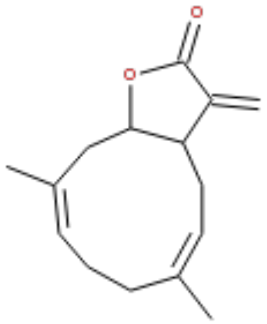

Cell ID	Cluster Center	Number of Compounds
588	1	8

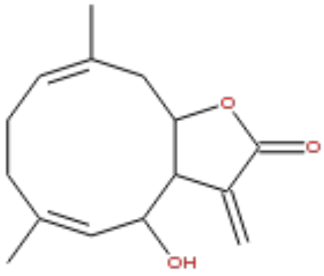

Cell ID	Cluster Center	Number of Compounds
588	0	8

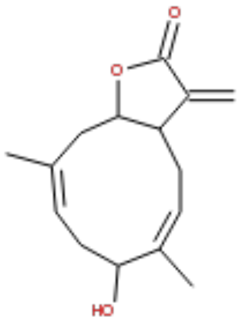

Cell ID	Cluster Center	Number of Compounds
588	0	8

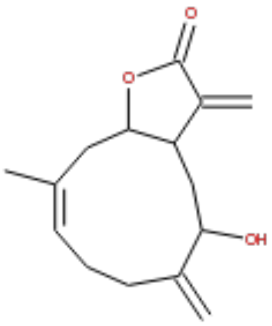

Cell ID	Cluster Center	Number of Compounds
588	0	8

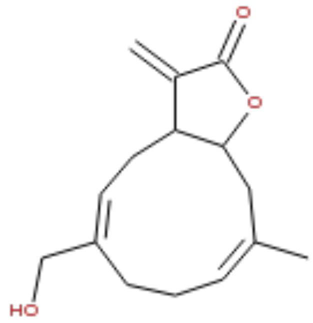

Cell ID	Cluster Center	Number of Compounds
588	0	8

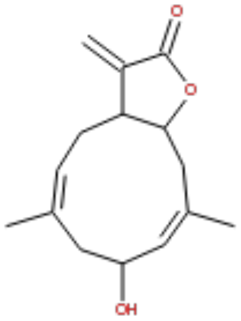

Cell ID	Cluster Center	Number of Compounds
588	0	8

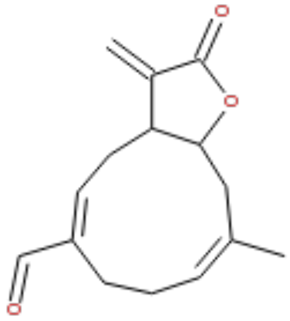

Cell ID	Cluster Center	Number of Compounds
588	0	8

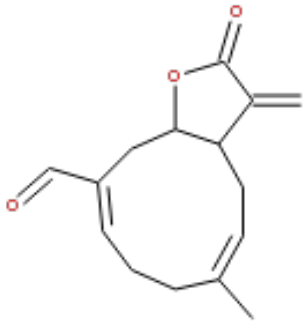

Cell ID	Cluster Center	Number of Compounds
588	0	8

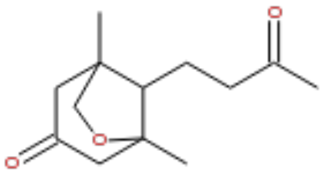

Cell ID	Cluster Center	Number of Compounds
589	1	4

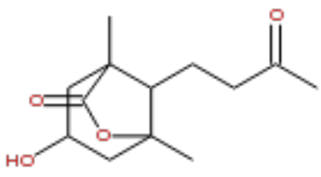

Cell ID	Cluster Center	Number of Compounds
589	0	4

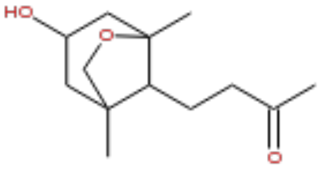

Cell ID	Cluster Center	Number of Compounds
589	0	4

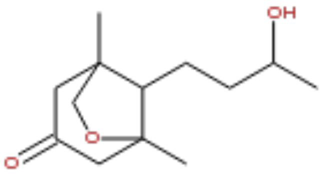

Cell ID	Cluster Center	Number of Compounds
589	0	4

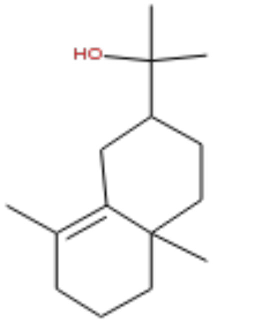

Cell ID	Cluster Center	Number of Compounds
590	1	17

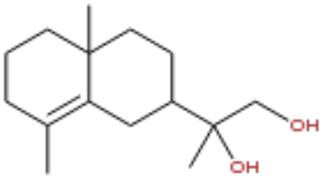

Cell ID	Cluster Center	Number of Compounds
590	0	17

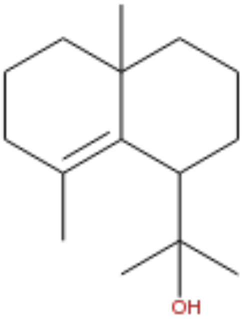

Cell ID	Cluster Center	Number of Compounds
590	0	17

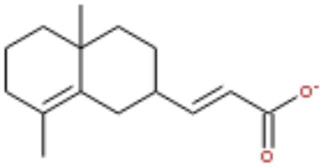

Cell ID	Cluster Center	Number of Compounds
590	0	17

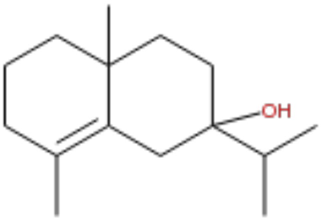

Cell ID	Cluster Center	Number of Compounds
590	0	17

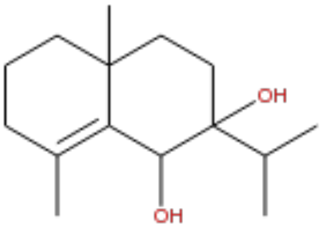

Cell ID	Cluster Center	Number of Compounds
590	0	17

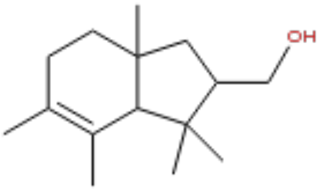

Cell ID	Cluster Center	Number of Compounds
590	0	17

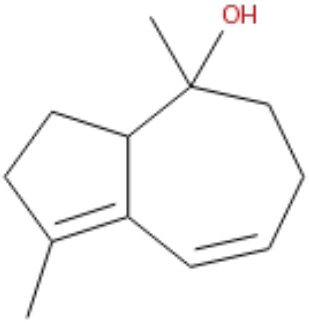

Cell ID	Cluster Center	Number of Compounds
590	0	17

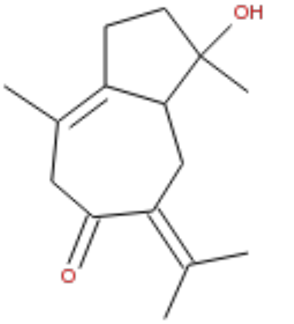

Cell ID	Cluster Center	Number of Compounds
590	0	17

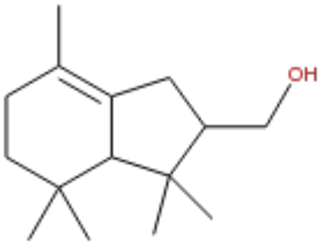

Cell ID	Cluster Center	Number of Compounds
590	0	17

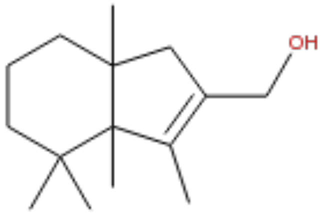

Cell ID	Cluster Center	Number of Compounds
590	0	17

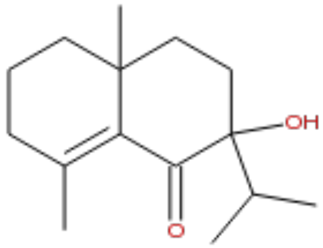

Cell ID	Cluster Center	Number of Compounds
590	0	17

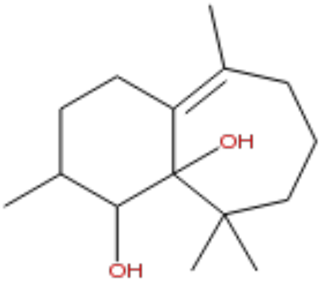

Cell ID	Cluster Center	Number of Compounds
590	0	17

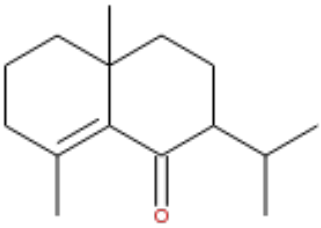

Cell ID	Cluster Center	Number of Compounds
590	0	17

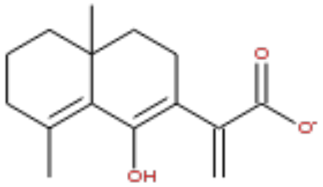

Cell ID	Cluster Center	Number of Compounds
590	0	17

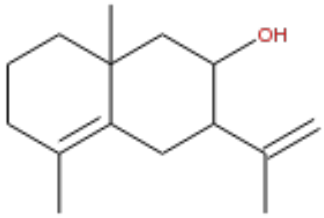

Cell ID	Cluster Center	Number of Compounds
590	0	17

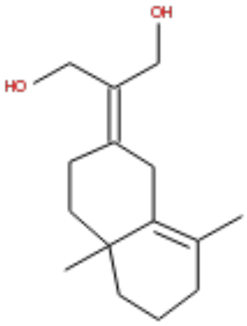

Cell ID	Cluster Center	Number of Compounds
590	0	17

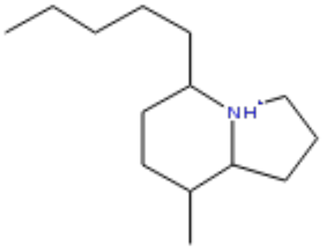

Cell ID	Cluster Center	Number of Compounds
592	1	7

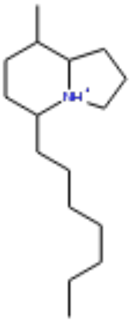

Cell ID	Cluster Center	Number of Compounds
592	0	7

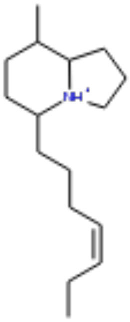

Cell ID	Cluster Center	Number of Compounds
592	0	7

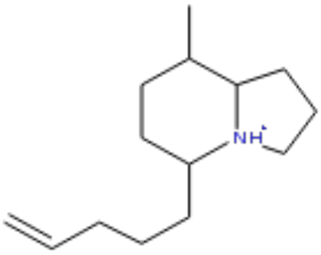

Cell ID	Cluster Center	Number of Compounds
592	0	7

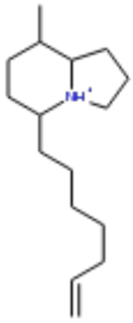

Cell ID	Cluster Center	Number of Compounds
592	0	7

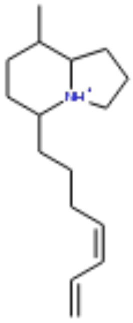

Cell ID	Cluster Center	Number of Compounds
592	0	7

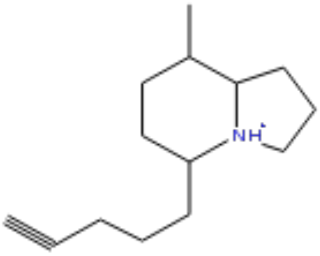

Cell ID	Cluster Center	Number of Compounds
592	0	7

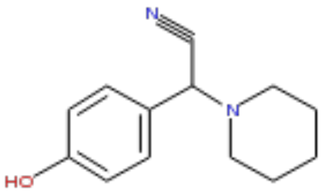

Cell ID	Cluster Center	Number of Compounds
593	1	5

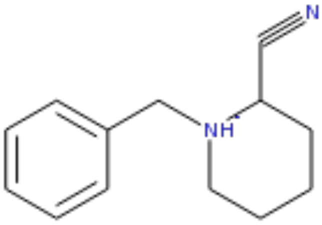

Cell ID	Cluster Center	Number of Compounds
593	0	5

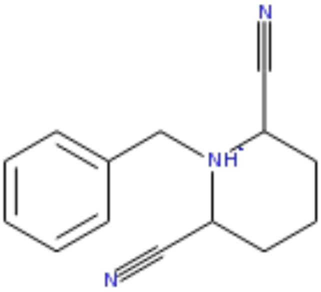

Cell ID	Cluster Center	Number of Compounds
593	0	5

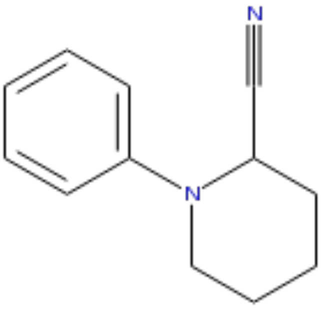

Cell ID	Cluster Center	Number of Compounds
593	0	5

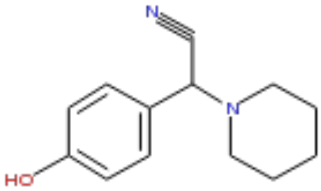

Cell ID	Cluster Center	Number of Compounds
593	0	5

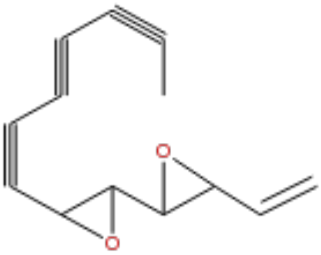

Cell ID	Cluster Center	Number of Compounds
594	1	1

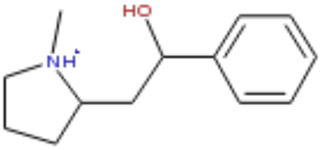

Cell ID	Cluster Center	Number of Compounds
597	1	7

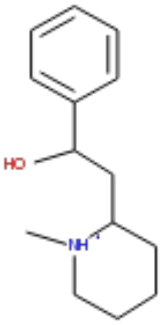

Cell ID	Cluster Center	Number of Compounds
597	0	7

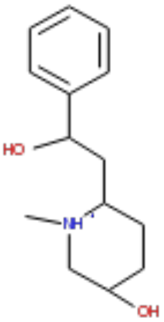

Cell ID	Cluster Center	Number of Compounds
597	0	7

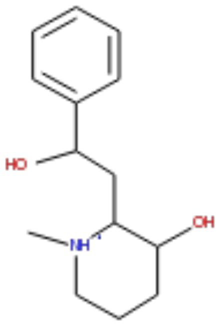

Cell ID	Cluster Center	Number of Compounds
597	0	7

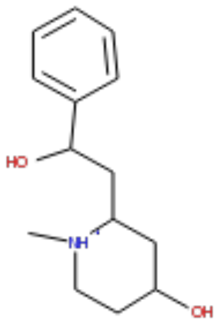

Cell ID	Cluster Center	Number of Compounds
597	0	7

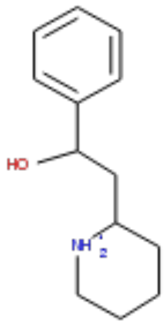

Cell ID	Cluster Center	Number of Compounds
597	0	7

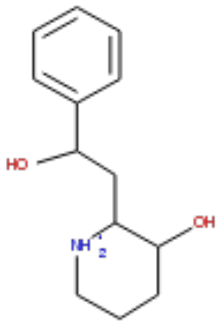

Cell ID	Cluster Center	Number of Compounds
597	0	7

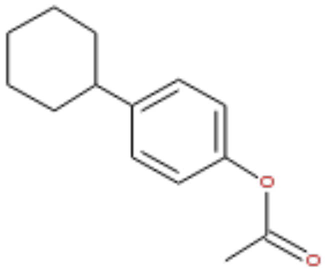

Cell ID	Cluster Center	Number of Compounds
598	1	6

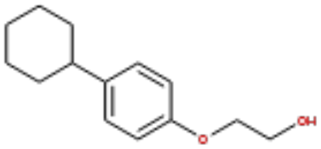

Cell ID	Cluster Center	Number of Compounds
598	0	6

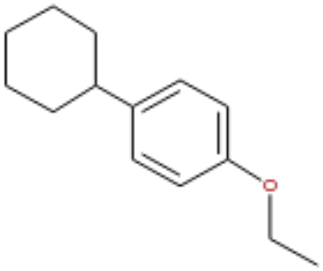

Cell ID	Cluster Center	Number of Compounds
598	0	6

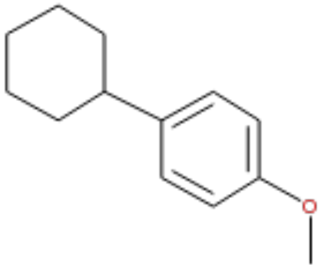

Cell ID	Cluster Center	Number of Compounds
598	0	6

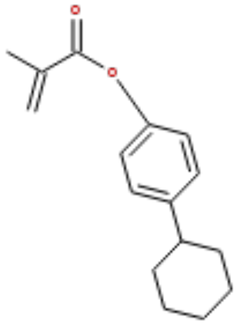

Cell ID	Cluster Center	Number of Compounds
598	0	6

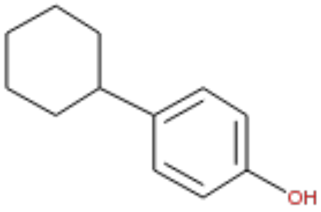

Cell ID	Cluster Center	Number of Compounds
598	0	6

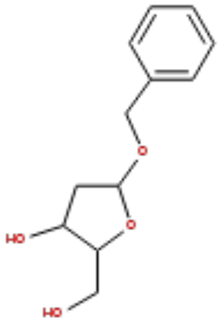

Cell ID	Cluster Center	Number of Compounds
599	1	5

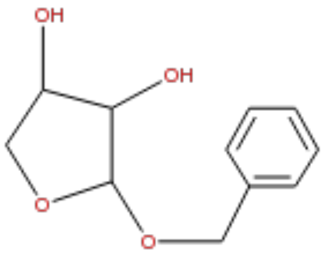

Cell ID	Cluster Center	Number of Compounds
599	0	5

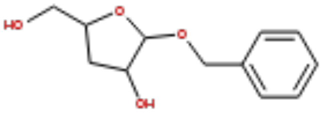

Cell ID	Cluster Center	Number of Compounds
599	0	5

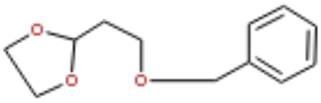

Cell ID	Cluster Center	Number of Compounds
599	0	5

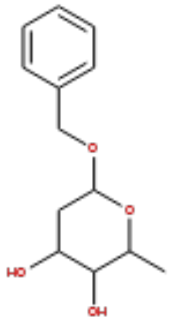

Cell ID	Cluster Center	Number of Compounds
599	0	5

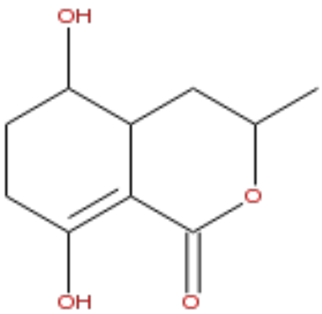

Cell ID	Cluster Center	Number of Compounds
600	1	5

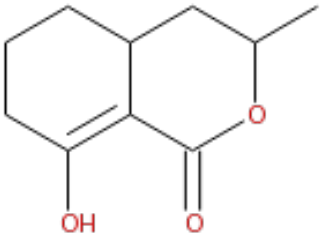

Cell ID	Cluster Center	Number of Compounds
600	0	5

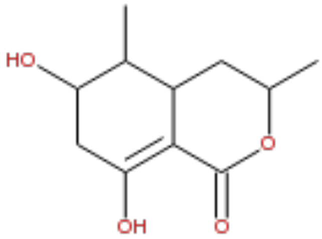

Cell ID	Cluster Center	Number of Compounds
600	0	5

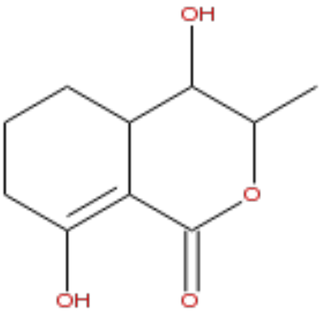

Cell ID	Cluster Center	Number of Compounds
600	0	5

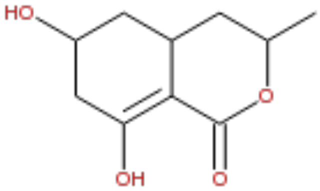

Cell ID	Cluster Center	Number of Compounds
600	0	5

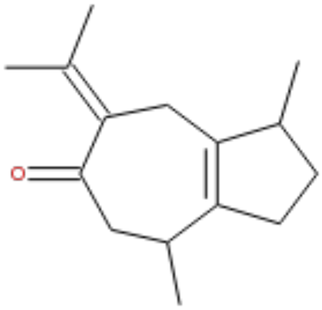

Cell ID	Cluster Center	Number of Compounds
602	1	3

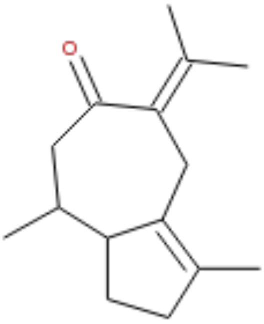

Cell ID	Cluster Center	Number of Compounds
602	0	3

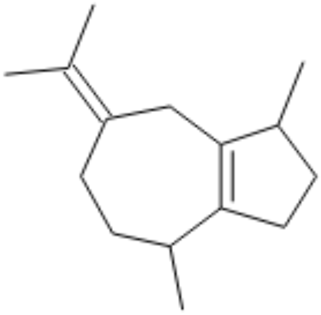

Cell ID	Cluster Center	Number of Compounds
602	0	3

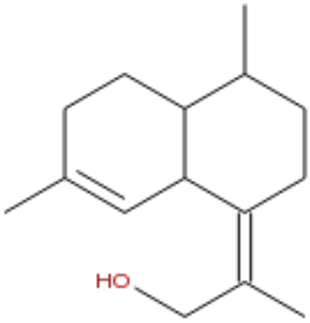

Cell ID	Cluster Center	Number of Compounds
603	1	5

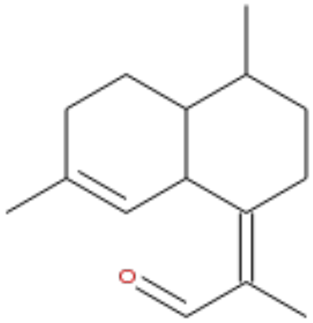

Cell ID	Cluster Center	Number of Compounds
603	0	5

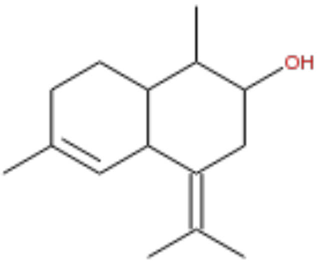

Cell ID	Cluster Center	Number of Compounds
603	0	5

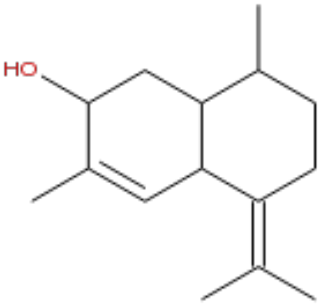

Cell ID	Cluster Center	Number of Compounds
603	0	5

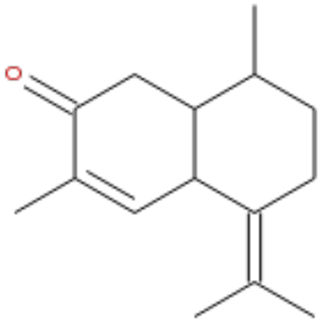

Cell ID	Cluster Center	Number of Compounds
603	0	5

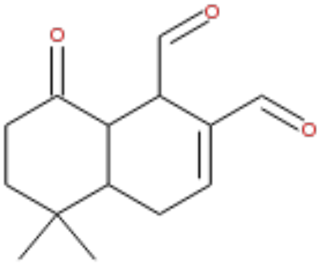

Cell ID	Cluster Center	Number of Compounds
605	1	3

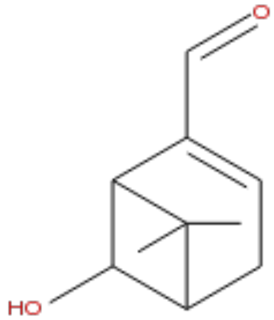

Cell ID	Cluster Center	Number of Compounds
605	0	3

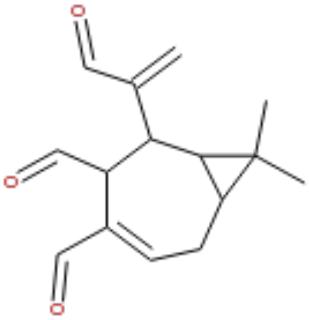

Cell ID	Cluster Center	Number of Compounds
605	0	3

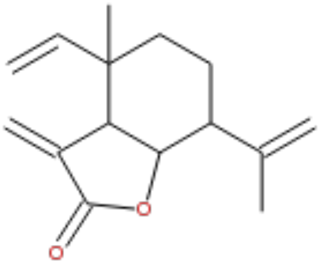

Cell ID	Cluster Center	Number of Compounds
607	1	2

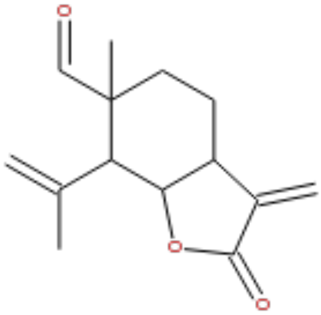

Cell ID	Cluster Center	Number of Compounds
607	0	2

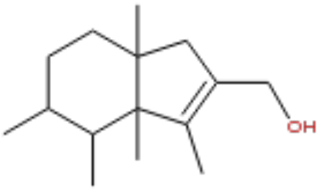

Cell ID	Cluster Center	Number of Compounds
608	1	4

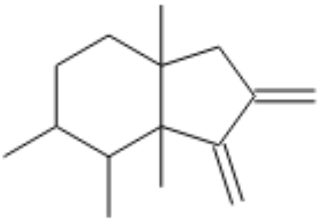

Cell ID	Cluster Center	Number of Compounds
608	0	4

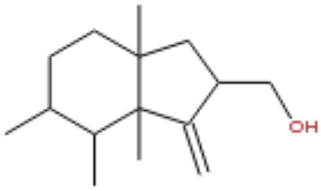

Cell ID	Cluster Center	Number of Compounds
608	0	4

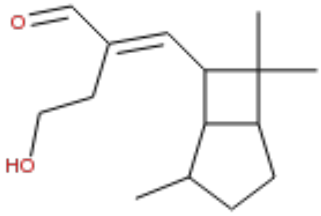

Cell ID	Cluster Center	Number of Compounds
608	0	4

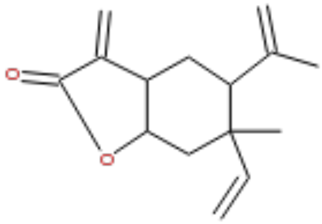

Cell ID	Cluster Center	Number of Compounds
611	1	2

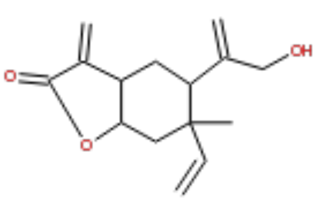

Cell ID	Cluster Center	Number of Compounds
611	0	2

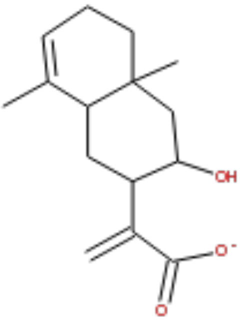

Cell ID	Cluster Center	Number of Compounds
614	1	1

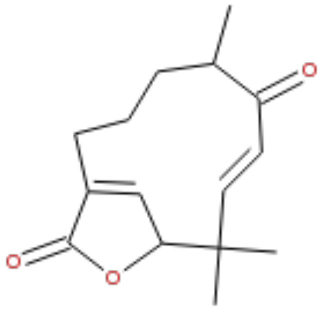

Cell ID	Cluster Center	Number of Compounds
615	1	1

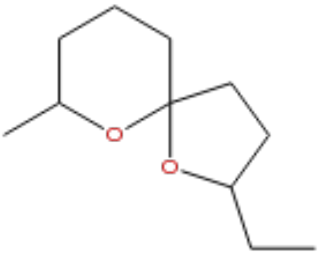

Cell ID	Cluster Center	Number of Compounds
616	1	28

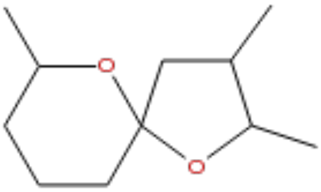

Cell ID	Cluster Center	Number of Compounds
616	0	28

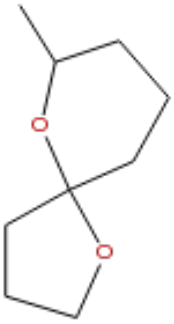

Cell ID	Cluster Center	Number of Compounds
616	0	28

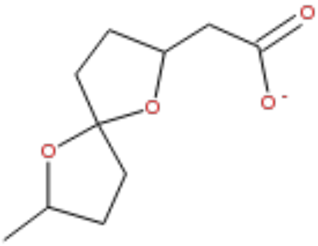

Cell ID	Cluster Center	Number of Compounds
616	0	28

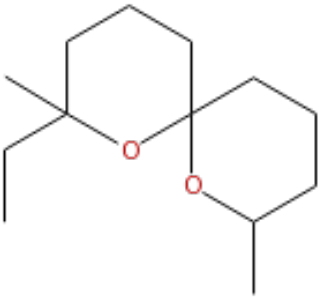

Cell ID	Cluster Center	Number of Compounds
616	0	28

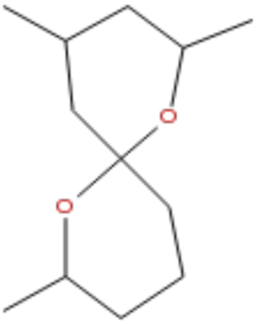

Cell ID	Cluster Center	Number of Compounds
616	0	28

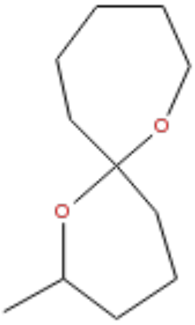

Cell ID	Cluster Center	Number of Compounds
616	0	28

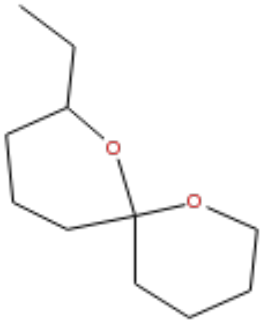

Cell ID	Cluster Center	Number of Compounds
616	0	28

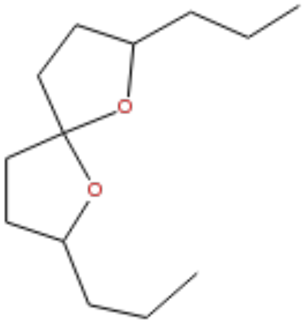

Cell ID	Cluster Center	Number of Compounds
616	0	28

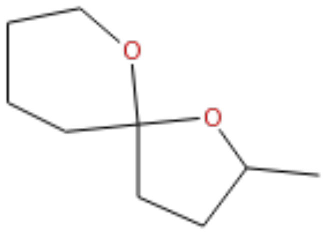

Cell ID	Cluster Center	Number of Compounds
616	0	28

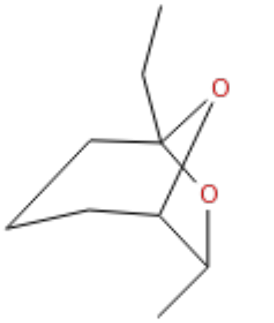

Cell ID	Cluster Center	Number of Compounds
616	0	28

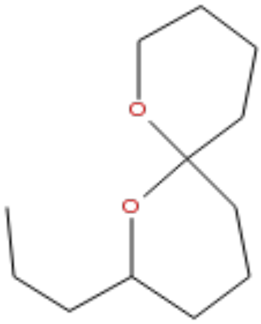

Cell ID	Cluster Center	Number of Compounds
616	0	28

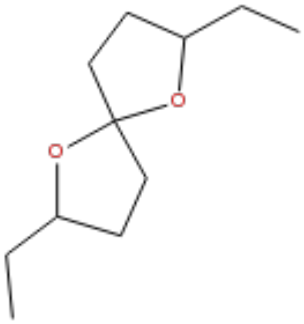

Cell ID	Cluster Center	Number of Compounds
616	0	28

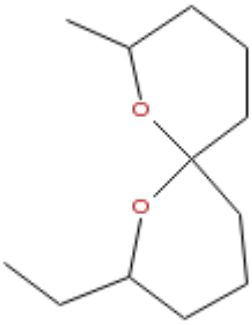

Cell ID	Cluster Center	Number of Compounds
616	0	28

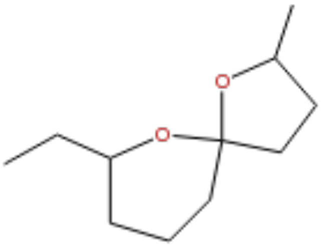

Cell ID	Cluster Center	Number of Compounds
616	0	28

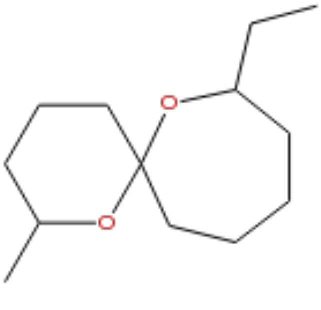

Cell ID	Cluster Center	Number of Compounds
616	0	28

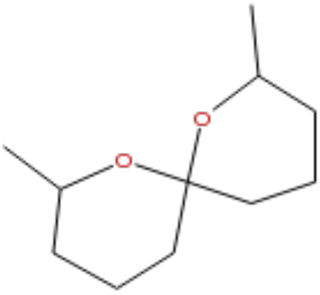

Cell ID	Cluster Center	Number of Compounds
616	0	28

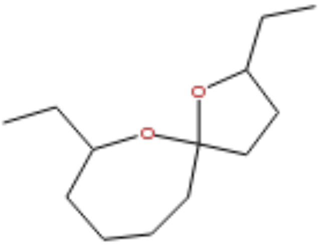

Cell ID	Cluster Center	Number of Compounds
616	0	28

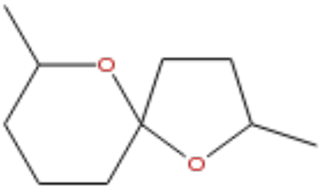

Cell ID	Cluster Center	Number of Compounds
616	0	28

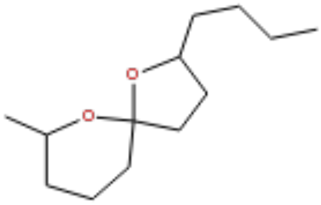

Cell ID	Cluster Center	Number of Compounds
616	0	28

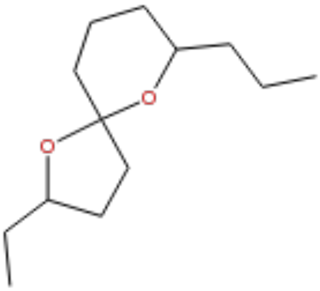

Cell ID	Cluster Center	Number of Compounds
616	0	28

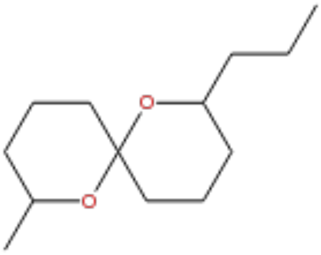

Cell ID	Cluster Center	Number of Compounds
616	0	28

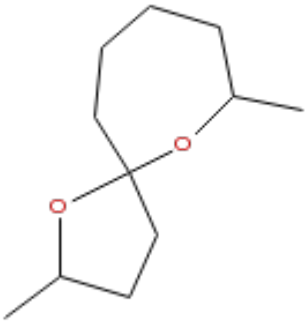

Cell ID	Cluster Center	Number of Compounds
616	0	28

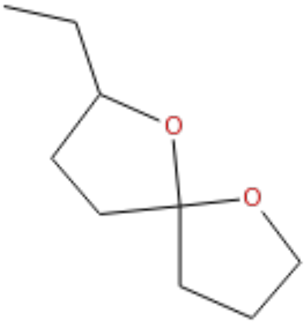

Cell ID	Cluster Center	Number of Compounds
616	0	28

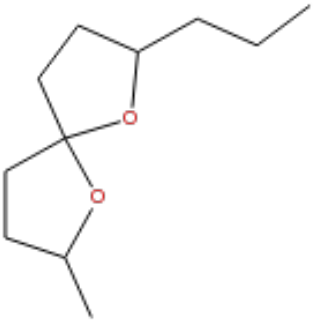

Cell ID	Cluster Center	Number of Compounds
616	0	28

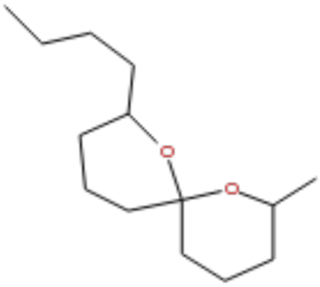

Cell ID	Cluster Center	Number of Compounds
616	0	28

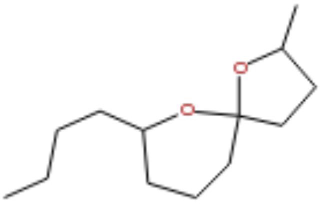

Cell ID	Cluster Center	Number of Compounds
616	0	28

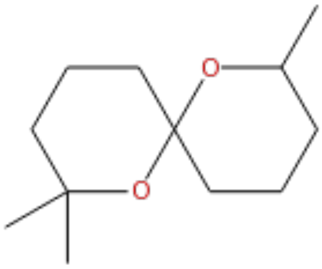

Cell ID	Cluster Center	Number of Compounds
616	0	28

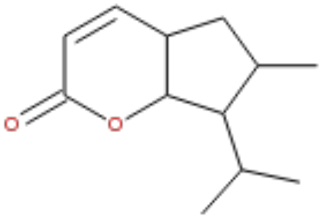

Cell ID	Cluster Center	Number of Compounds
617	1	2

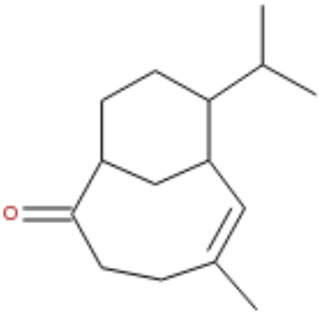

Cell ID	Cluster Center	Number of Compounds
617	0	2

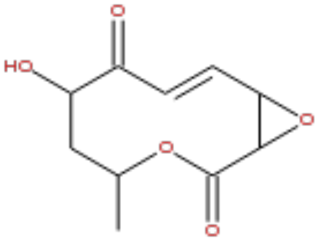

Cell ID	Cluster Center	Number of Compounds
618	1	3

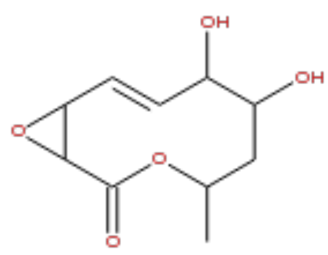

Cell ID	Cluster Center	Number of Compounds
618	0	3

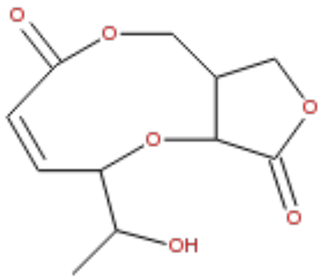

Cell ID	Cluster Center	Number of Compounds
618	0	3
