# Supplementary material for: Capturing Nature's Diversity
Source: PLoS One. 2015 Apr 22;10(4):e0120942. doi: 10.1371/journal.pone.0120942 (PMC4406718; doi:10.1371/journal.pone.0120942)
Supplement: S1 Fig — (PDF) [file pone.0120942.s004.pdf]

**S3 Figure. Self-organizing maps of the DNP and fragment-sized natural products using pharmacophore fingerprint.**

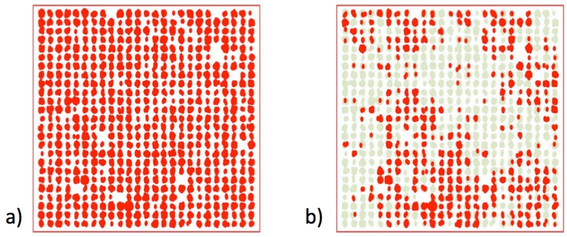


**Fig. A.** Distribution of compounds within the SOM trained using pharmacophore fingerprints of 20185 fragment-sized natural products. **(a)** 20185 fragment-sized natural products. **(b)** 7365 non-flat fragment-sized natural products (F$sp^3$* > 0.45). Each cell represent a cluster of fragments and the population of the cells is indicated by the size of the red spots.

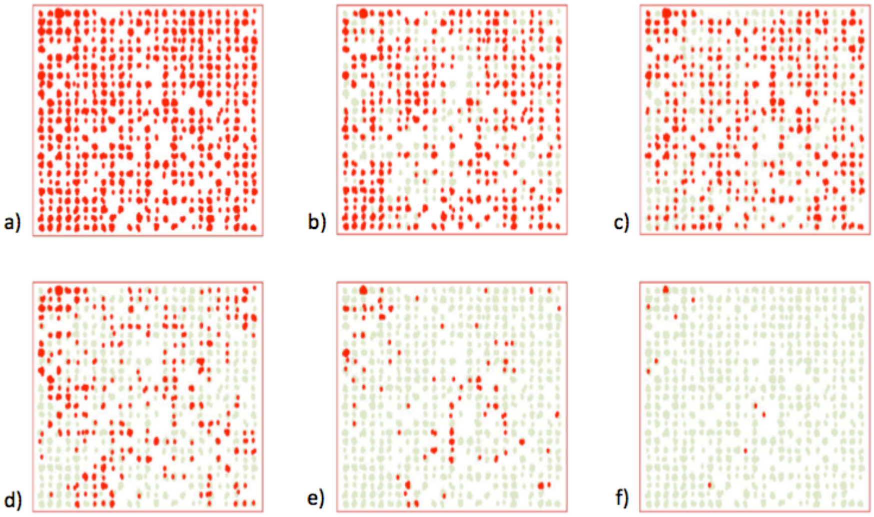


**Fig. B**. Distribution of compounds within the SOM trained using pharmacophore fingerprints of 7365 non-flat fragment-sized natural products. **(a)** 7365 non-flat fragment-sized natural products; **(b)** 1-ring molecules; 57 % coverage of non-flat fragments. **(c)** 2-ring molecules; 55 % coverage of non-flat fragments. **(d)** 3-ring molecules; 37 % coverage of non-flat fragments. **e)** 4-ring molecules; 13 % coverage of non-flat fragments. **(f)** 5-ring molecules; 2 % coverage of non-flat fragments. Each cell represent a cluster of fragments and the population of the cells is indicated by the size of the red spots.
